# Multiomics Integration Reveals Microbial Gene Interactions Shaping Host Responses in a DSS-Induced Colitis Mouse Model

**DOI:** 10.4014/jmb.2507.07010

**Published:** 2025-10-15

**Authors:** Hyun-Hee Hong, Seo-Yeong Lee, Da Hae Jang, Si-Eun Ju, Ji Eun Shim, Tae-Hwan Kim, Hyung-Sik Kang, Su-Man Kim

**Affiliations:** 1 School of Biological Sciences and Technology, Chonnam National University, Gwangju 61186, Republic of Korea; 2 Macrogen Inc., Seoul 06221, Republic of Korea; 3 College of Veterinary Medicine, Chungnam National University, Daejeon 34134, Republic of Korea; 4 Department of Biology Education, Chonnam National University, Gwangju 61186, Republic of Korea

**Keywords:** DSS-induced colitis model, inflammatory bowel disease, metatranscriptomics, Aminoacyl-tRNA synthetase (aaRS), sparse canonical correlation analysis, host-microbiome association

## Abstract

Inflammatory bowel disease (IBD) has been studied with a multi-omics approach to identify key contributors and unravel the biological complexity of its pathogenesis, aiding in the development of early diagnostic markers and therapeutic targets. The dextran sulfate sodium (DSS)-induced colitis mouse model, a widely used system for studying IBD, induces gut barrier disruption and proinflammatory responses, making it an ideal model for investigating host-microbiome interactions. This study emphasizes the intricate relationship between microbial transcriptomic changes and host immune responses, revealing regulation of microbial genes, particularly in metabolic pathways related to carbohydrate metabolism, nucleotide metabolism, and aminoacyl-tRNA biosynthesis under inflammatory conditions. We identified key hub microbes and microbial genes that are closely associated with host immunological pathways, with particular focus on microbial aminoacyl-tRNA synthetases (aaRSs), which play significant roles in immune cell activation and inflammatory pathways. These findings offer valuable insights into the microbial contributions to inflammation and immune modulation in IBD, highlighting the potential role of aaRSs in regulating immune responses beyond their traditional function in translation. This lays the foundation for future research into host-microbiome interactions in inflammatory diseases and the development of novel therapeutic strategies that target microbial aaRSs to manipulate immune response.

## Introduction

Inflammatory bowel disease (IBD), which includes Crohn’s disease (CD) and ulcerative colitis (UC), causes uncontrollable inflammation in the intestinal tract. Although the etiology of IBD remains unresolved, complex factors such as genetic susceptibility, immune disorders, environmental influences, and microbial dysbiosis have been proposed as potential causes of IBD [1-5]. In recent decades, IBD has been investigated from a multiomics perspective in the fields of genomics, proteomics, transcriptomics, and metagenomics to identify key players and to understand the biological complexity involved in IBD pathogenesis. These data have contributed to the development of early diagnostic markers and therapeutic targets [6-16]. In particular, recent studies have focused on an integrative approach to omics data, as this enables insights into the convergence of multiple factors [17-23].

The microbiome has been emphasized for its role in modulating the immune system, with microbial aaRSs being of particular interest due to their recently recognized immunomodulatory effects that extend beyond their canonical functions in protein synthesis. In a recent study, *Akkermansia muciniphila* threonyl-tRNA synthetases (TARS) were found to acquire additional domains that contribute to host anti-inflammatory responses [24]. Moreover, extracellular TARS has been implicated in the development of immune cells [25]. Evolutionarily, the acquisition of novel domains in microbial aaRSs may enable bacteria to gain new cellular functions. Similar to recent findings that human aaRSs possess non-canonical roles beyond protein synthesis [26, 27], microbial aaRSs may also have previously unrecognized functions affecting host immunity and disease. Despite their potential significance, microbial aaRSs remain largely unexplored, and understanding their roles could provide new insights into host immune regulation and the pathogenesis of IBD. Additionally, patients with IBD have been reported to exhibit gut microbiota dysbiosis [28-30], with microbes contributing to epithelial homeostasis through interactions with Toll-like receptors (TLRs) and the production of beneficial metabolites such as vitamins and short-chain fatty acids (SCFAs) [31-33]. Despite the growing evidence that the dynamics of specific microbial functions have a crucial role in IBD, microbial transcriptomic alterations in the IBD gut environment remain poorly understood [34]. Metatranscriptomic analyses could provide insights into active microbial populations and their functional roles, offering valuable information for understanding the gut environment in IBD patients [35, 36]. Metatranscriptomics is more robust than metagenomics for studying bacterial metabolism, although inconsistencies between microbial abundance and transcriptional activity have been reported [37, 38].

The dextran sulfate sodium (DSS)-induced colitis mouse model is a well-established model for IBD, as it leads to the collapse of gut barrier integrity and induces proinflammatory responses. Furthermore, the DSS mouse model is a suitable system for investigating host‒microbiome interactions, as DSS alone does not exert any significant effect on the gut microbiota in the absence of the host environment [39]. Based on this background, the DSS-induced colitis mouse model was employed to investigate multiomics networks, establishing host‒microbiome interaction networks based on microbial taxonomy and gene expression. The objective was to identify key microbial species and their genes, including aaRSs, that statistically associate with host immune responses, providing insights into potential therapeutic targets and offering a framework for future studies on IBD.

## Materials and Methods

### Induction of DSS-Induced Colitis in a Mouse Model

Fifteen C57BL/6J mice (male, 23.8 ± 1.2 g) were obtained from Damool Science (Republic of Korea) and were originally imported from The Jackson Laboratory. Following an acclimation period of 7 weeks at 22 ± 1°C under a 12-h light/dark cycle, with autoclaved drinking water and food provided ad libitum, the mice were randomly assigned to three experimental groups (*n* = 5 per group): control, DSS 3-day, and DSS 6-day. The 3- and 6-day DSS groups received 2% DSS solution in their drinking water with unrestricted access, whereas the control group was administered only drinking water for 6 days. The mice were sacrificed on Days 3 and 6, respectively. All experimental procedures were approved by the Institutional Animal Care and Use Committee of Chonnam National University, Korea (CNU IACUC-YB-2024-158). Body weight was monitored throughout the experimental period, and colon length was recorded at the time of sacrifice.

### Sample Collection and DNA/RNA Extraction

The colons were harvested from sacrificed mice, and fecal samples were isolated from the colon. The dissected colon samples were washed with phosphate-buffered saline (PBS). Colon and fecal samples for RNA extraction were preserved in RNAlater stabilization solution (Invitrogen, USA) after homogenization by vortexing, and fecal samples for amplicon sequencing were stored at −20°C until sequencing. DNA was extracted from the fecal samples using a DNeasy PowerSoil Pro Kit (QIAGEN, Germany) according to the manufacturer’s instructions. After the removal of RNAlater by centrifugation, RNA was extracted from both the colon and fecal samples using TRIzol (Invitrogen), followed by treatment with QIAzol Lysis Reagent (QIAGEN) and RNase-Free DNase I (QIAGEN).

### Library Preparation and Sequencing

For amplicon sequencing, the V3–V4 region of the 16S rRNA gene was amplified by PCR, and the amplicon library was constructed according to the Illumina 16S Metagenomic Sequencing Library Preparation guide (Illumina, USA). Sequencing was performed on the MiSeq platform (Illumina), generating 300 bp paired-end reads. For metatranscriptomic analysis, RNA libraries of mouse fecal samples were prepared using a TruSeq Stranded Total RNA Library Prep Gold Kit (Illumina), with rRNA depletion performed using an Illumina Ribo-Zero Plus rRNA Depletion Kit, following the TruSeq Stranded Total RNA with Illumina Ribo-Zero Plus rRNA Depletion Reference Guide. Sequencing was conducted on the Illumina NovaSeq platform (Illumina), which produced 150 bp paired-end reads. Colonic RNA was processed using a TruSeq Stranded Total RNA Library Prep Gold Kit according to the TruSeq Stranded Total RNA Reference Guide (1000000040499 v00). Sequencing was performed on the NovaSeq X platform, generating 100 bp paired-end reads. All the constructed libraries were quality-assessed using a TapeStation D1000 ScreenTape system (Agilent Technologies, Germany). Three samples (1_15, 2_15, 3_15) from each library were excluded from further analysis because of the presence of small dimers near the major peak in the metatranscriptomic library. The sequencing datasets used in this study are available in the National Center Biotechnology Information repository with accession number PRJNA1261586 (https://www.ncbi.nlm.nih.gov/bioproject/PRJNA1261586).

### 16S rRNA Amplicon Analysis

The raw data were processed using Cutadapt (v3.2) [40] to remove adapter sequences from both forward and reverse reads. Sequences lacking V3–V4 primer sequences were excluded from further analysis. Amplicon sequence variants (ASVs) analysis was performed using DADA2 (v1.18.0) [41]. The reads were filtered using the truncLen and maxEE parameters (-p-trunc-len-f: 250, -p-trunc-len-r: 200, --p-max-ee: 2) to remove low-quality bases. ASVs shorter than 350 bp were excluded to improve taxonomic accuracy. ASVs were inferred and subsequently normalized to the minimum read count across all samples. Taxonomic classification was performed using BLAST (v2.9.0) by aligning sequences to the NCBI 16S Microbial Database [42]. ASVs with query coverage or identity less than 85% were assigned to "unclassified". Alpha diversity was assessed using the Shannon, Chao1, and Gini‒Simpson indices via QIIME scripts [43]. Beta diversity was calculated using the Bray–Curtis index calculated in the vegan R package (v2.6.10) [44], and principal coordinate analysis (PCoA) was performed. The Wilcoxon rank-sum test was performed to evaluate differences between pairwise groups, with *p* values adjusted using the Benjamini–Hochberg correction. Adjusted *p* values < 0.05 were considered. A circular heatmap was constructed using the ggtree R package (v3.12.0) [45] to compare the abundance of statistically significant species.

### Metatranscriptome Analysis

The raw data were processed using KneadData (https://bitbucket.org/biobakery/kneaddata) with Trimmomatic (v0.33) [46] and Bowtie2 [47] to remove adapter and host-derived sequences based on the GRCm39 reference genome, respectively, and the bacterial transcript sequences were retained. Owing to the difficulty in performing taxonomic classification in the DSS group, Kraken2 [48] was employed to increase the accuracy of microbial taxonomy with the k2_standard database [49]. Abundance estimation was subsequently conducted using Bracken [50]. The resulting report was converted to MetaPhlAn format using the Bracken script (kreport2mpa.py) and then input into HUMAnN3 [51] with the --taxonomic-profile option. Gene abundance estimation was performed using HUMAnN3 with the ChocoPhlAn nucleotide and UniRef90 protein databases [52]. The UniRef90 annotations were then regrouped and mapped to EC numbers. Because the taxonomic and transcript abundances in several samples were remarkably low (2_9, 2_10, 2_11, 2_12, 2_13), particularly in samples of the DSS 6-day group, these samples were excluded from further statistical analysis. To normalize transcript abundance while accounting for taxonomic composition, taxon-specific scaling was applied, where raw read counts were adjusted for each organism [53]. The Wilcoxon rank-sum test with Benjamini-Hochberg correction was used to compare the control and DSS 3-day groups, with adjusted *p* values less than 0.05 considered statistically significant. The taxonomic abundance of transcripts was visualized as a stacked bar plot, with taxa comprising less than 0.03%relative abundance grouped as "other," using ggplot2 (v3.5.1) [54], except for the top 15 most abundant taxa. Functional analysis of differentially expressed genes (DEGs) was performed using Kyoto Encyclopedia of Genes and Genomes (KEGG) pathway enrichment via the Enrichr web tool [55].

### Host Transcriptome Analysis

Adapter sequences were trimmed using Trimmomatic (v0.33) [46] with the following parameters: LEADING:15, TRAILING:15, SLIDINGWINDOW:4:15, and MINLEN:36. Transcripts were assembled with Trinity (v2.8.5), and open reading frames were predicted using TransDecoder (http://transdecoder.github.io), followed by sequence analysis with BLAST (v2.10.0) [42] against the UniProt-SwissProt database [56]. To eliminate sequence redundancy, CD-HIT (v0.0.1) [57] was used to select the best representative transcripts, which were annotated and the longest. Abundance estimation was performed using Bowtie2 [47] with the RSEM [58]. The expression counts were normalized using the trimmed mean of M values (TMM) method, and the normalized abundances were statistically compared by pairwise comparison using the DESeq2 R package (v1.44.0); with significance defined as an adjusted *p*-value (Benjamini-Hochberg) < 0.05 [59]. Principal component analysis (PCA) was performed using normalized gene expression counts. Significant DEGs were clustered based on comparison logic using a Venn diagram and visualized with a clustered line graph. Six gene modules were identified based on expression patterns: genes upregulated on Day 3 (module 1), genes commonly upregulated across both DSS-treated groups (module 2), genes uniquely upregulated (module 3) on Day 6, genes downregulated on Day 3 (module 4), genes commonly downregulated (module 5), and genes uniquely downregulated (module 6) on Day 6. The functional analysis of these modules was performed using KEGG pathway enrichment through the Enrichr web tool [55]. P values were -log10 transformed and are shown in a bubble plot.

### Sparse Canonical Correlation Analysis

Sparse canonical correlation analysis (sCCA) was employed to analyze correlations and extract significantly interacting modules between omics datasets, specifically comparing the colonic transcriptome with the bacterial transcriptome and the colonic transcriptome with 16S rRNA amplicon data. This method assigns weights to variables using a penalty parameter to enhance interpretability, allowing it to identify sets of highly correlated variables. Unlike simple correlation methods, sCCA employs parameter learning to focus on the most meaningful associations and effectively captures complex relationships within the data. Therefore, sCCA is well-suited to identify complex interactions between different biological layers including microbial features and host transcriptomes. Differentially expressed or abundant variables were processed under the criterion of excluding variables where only one sample had values while the others did not. The amplicon data were transformed using the log2 transformation method to standardize the values, as many of the amplicon sequencing data values were close to zero. The analysis followed the methodology outlined by Priya *et al*. [60], which included stages of calculating the penalty parameter, running sCCA, performing significance tests, and extracting components from each module using the leave-one-out cross-validation (LOOCV) method. The best penalty values were selected by calculating the average correlation coefficients across all modules from the CCA results, using penalty values ranging from 0.02–0.5, creating 20 equally spaced values, and selecting the one with the highest value. *P* values were adjusted using the Benjamini‒Hochberg method, and modules with *p* values less than 0.05 were regarded as significant. The host genes in each module were functionally analyzed for KEGG pathways using Enrichr [55].

### Network Analysis

To construct interaction networks between variables in each group and dataset, Spearman correlation was performed on differentially expressed or abundant variables. To create concise networks, we applied the criterion that only variables with *p* values less than 0.05 and rho values greater than or equal to 0.5 were included in the analysis. The network was constructed using Cytoscape (v3.10.3) [61], and network properties were calculated. The edge values were calculated as the -log10 transformation of the *p* values. The network was visualized using the Prefuse force-directed layout based on *p* values. To explore clusters and assess modularity based on network properties, clusterMaker2 (v2.3.4) [62] was used with the 'community cluster (GLay)' option. Hub microbial features were identified based on high neighborhood connectivity within the network, as this metric reflects how well a node is connected to other highly connected nodes, indicating its potential importance in maintaining network structure and mediating interactions.

Associations between host genes and microbial aminoacyl-tRNA synthetases (aaRSs) were confirmed within the network of the control group, as most interactions became undetectable after DSS induction. Host gene functions were annotated using Gene Ontology (GO) biological process terms via the clusterProfiler R package [63]. Among these, GO terms involving fewer than 100 genes and exhibiting a high number of associations with bacterial aaRSs were visualized using a chord diagram in the circlize R package [64].

## Results

### Colon Shortening and Weight Loss Following DSS Treatment

**Fig. 1:**
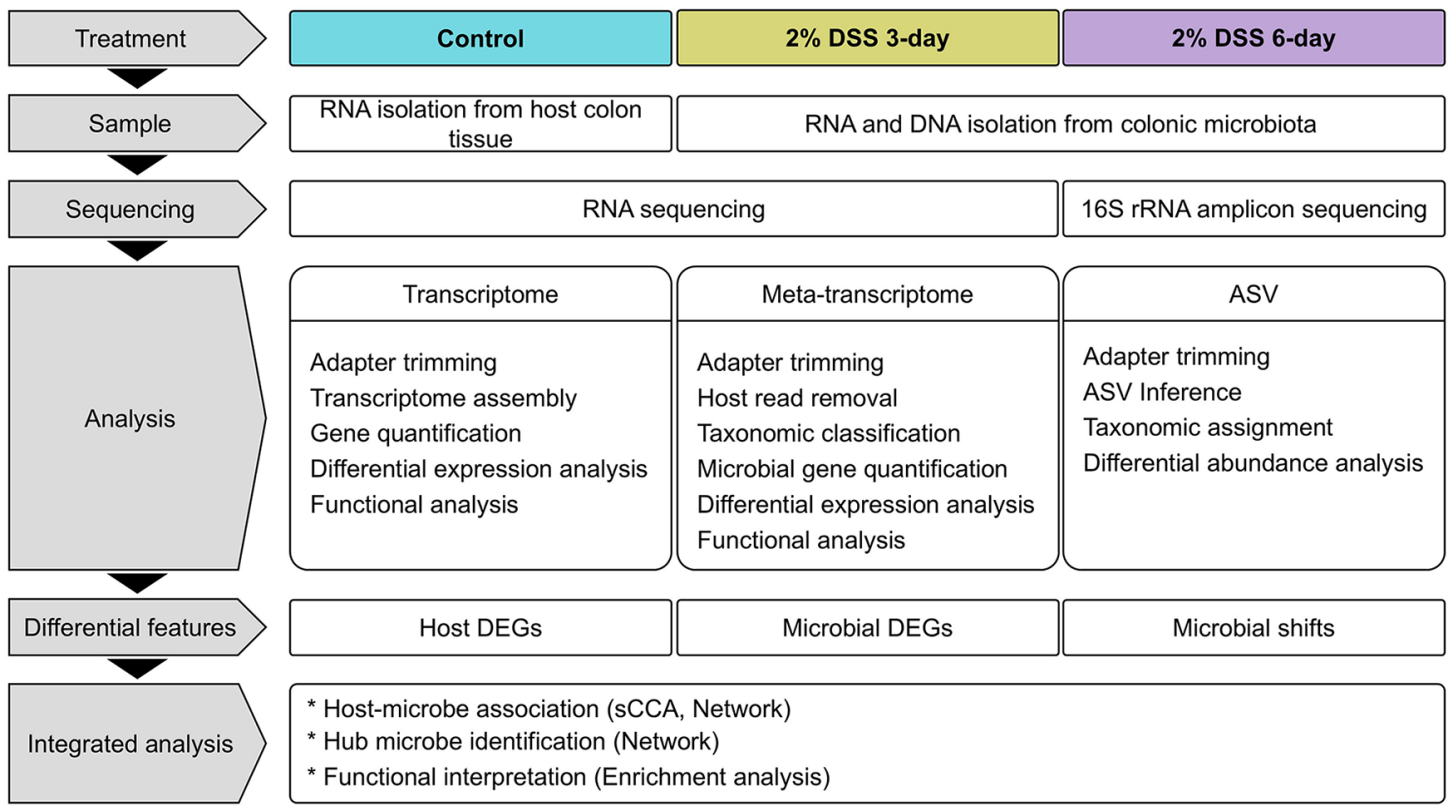
Schematic overview of the study. DSS-induced colitis model mice were sacrificed on Days 3 and 6. Colonic tissues and the colonic microbiota were collected, and RNA and DNA were extracted for next-generation sequencing. Omics analyses were performed on each dataset to identify differential features relative to the control group. Integrated analysis of host– microbe interactions was conducted using sparse canonical correlation analysis (sCCA) and network analysis. The biological functions of the associated host gene sets were assessed via enrichment analysis.

**Fig. 2:**
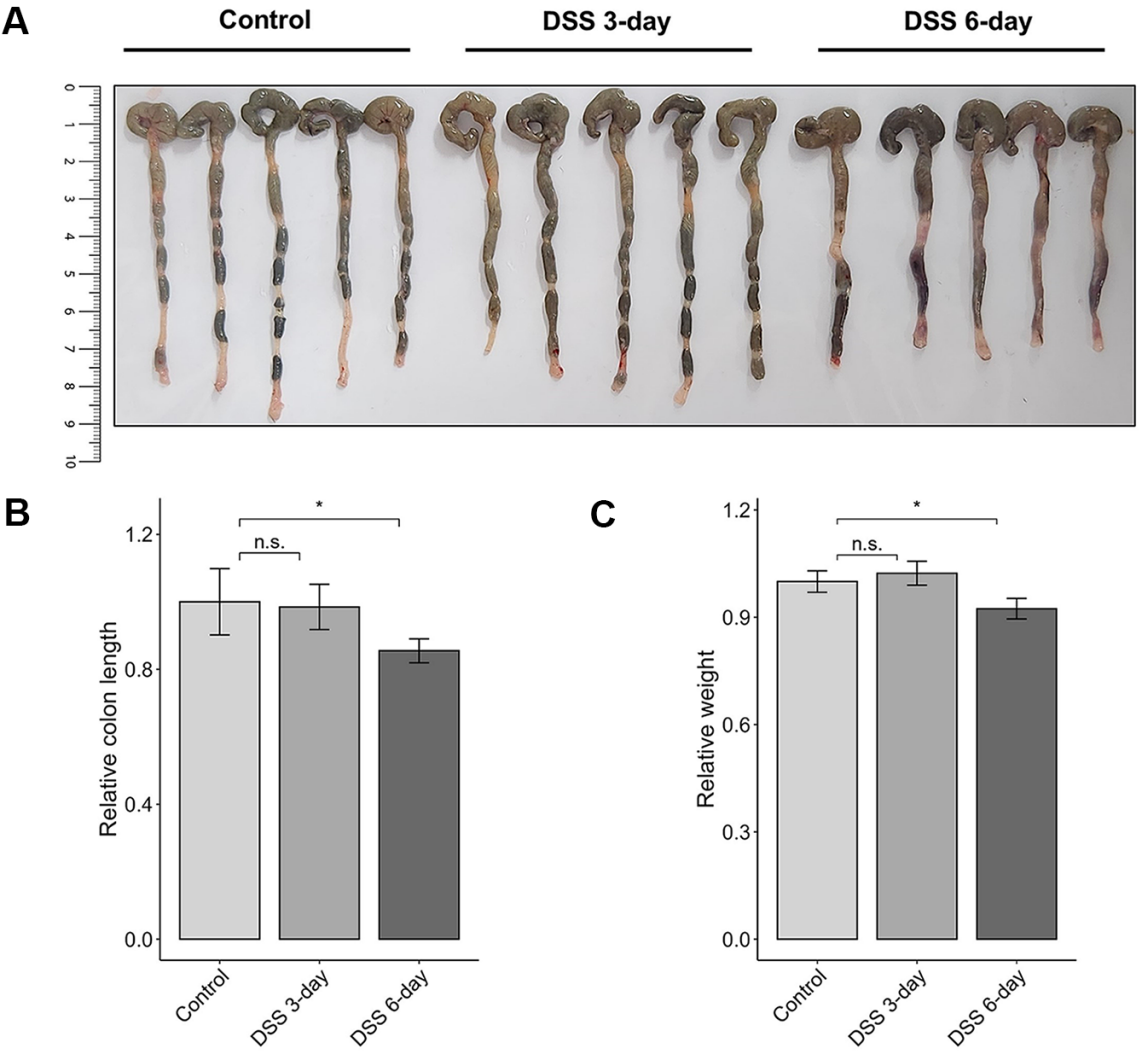
Effects of DSS treatment on colon length and body weight in mice. (**A**) Images of colons collected from control and DSS-treated mice. (**B**) Comparison of relative colon length and (**C**) relative body weight between groups. Statistical significance was determined using the Wilcoxon rank-sum test.

The overall experimental schema is illustrated in Fig. 1. Colon tissue and colonic microbiome samples were collected from mice induced with either water or 2% dextran sulfate sodium (DSS). Samples were obtained on Days 3 and 6 after induction. The mice began to exhibit mild signs of inflammation on Day 3, which progressed to severe inflammation by Day 6. In our experiments, DSS-induced mice presented typical IBD-like symptoms, including inflammation, weight loss and reduced colon length (Fig. 2) [65-67]. Although signs of mild inflammation were present, there were no significant differences in body weight or colon length compared with those of the control group on Day 3. However, both body weight and colon length were significantly reduced on Day 6, averaging 0.92 and 0.85, respectively, relative to those of the controls. These changes reflect substantial inflammation and tissue damage induced by DSS treatment.

### Microbial Community Dynamics in Response to DSS Treatment

**Fig. 3:**
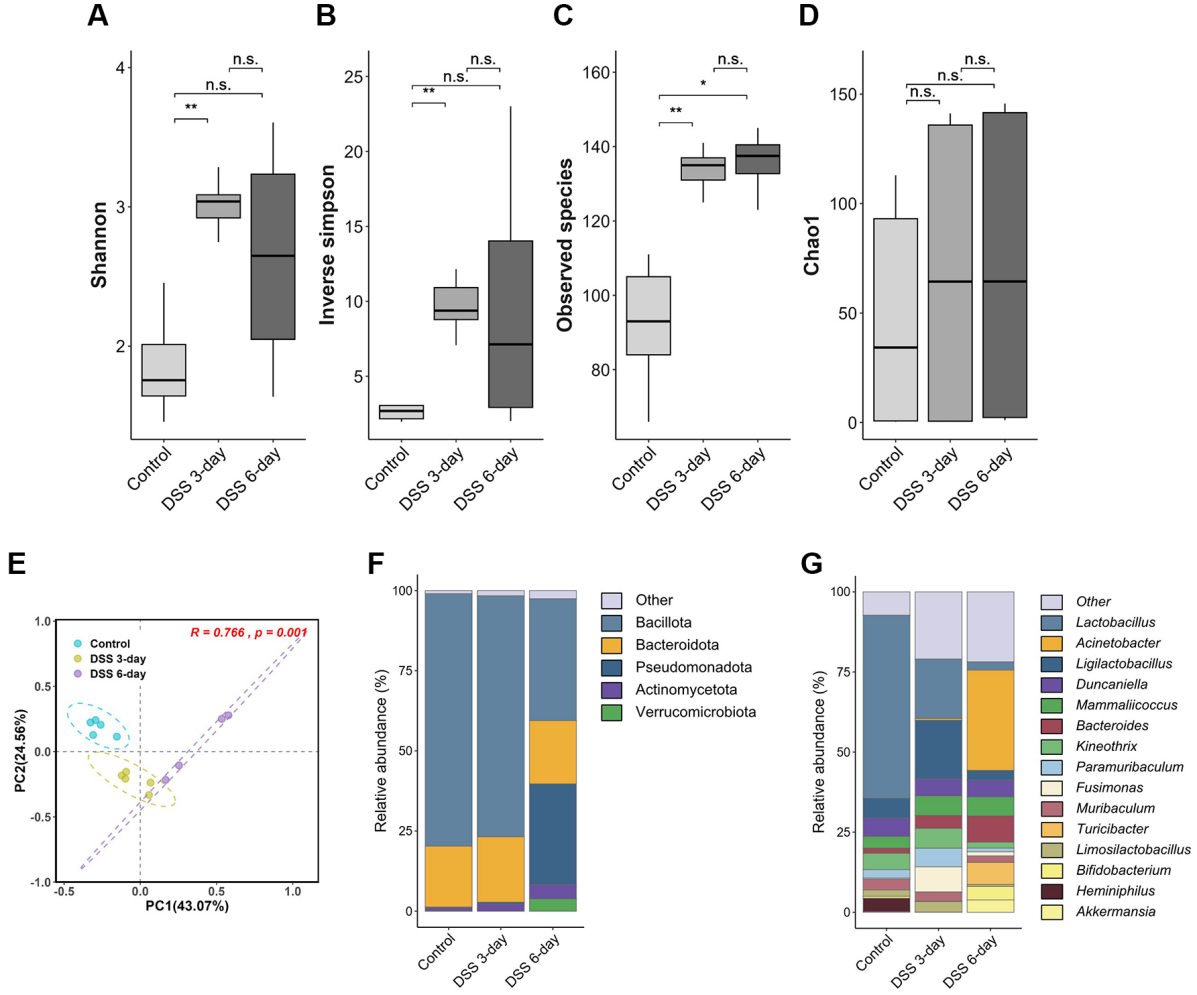
Changes in gut microbial diversity in response to DSS treatment. Comparisons of alpha diversity indices across groups: (**A**) Shannon diversity index, (**B**) Inverse Simpson index, (**C**) Observed species index, and (**D**) Chao1 index. (**E**) Beta diversity was assessed using principal coordinate analysis (PCoA) based on Bray–Curtis dissimilarity. Groupwise differences in sample distributions were evaluated using ANOSIM. Taxonomic abundances were compared at the (**F**) phylum and (**G**) genus levels. Taxa with relative abundances under 1% were grouped as “Other,” and the top 15 taxa are shown.

An average of 68 Mbp of raw sequencing data was generated, resulting in the construction of 37,264 amplicon sequence variants (ASVs) (Table S1). The number of detected microbial taxa was greater in the DSS-induced groups than in the control groups, with averages of 132.8 (*p* = 0.024) in the DSS 3-day group and 134.8 (*p* = 0.032) in the DSS 6-day group, whereas the control group had an average of 90.8 (Table S2). Alpha diversity analysis revealed significantly increased diversity in both DSS-treated groups (Fig. 3A-3D). In particular, the observed species index was markedly elevated, indicating that the increase in diversity was driven primarily by species richness. In contrast, the Chao1 index, which emphasizes rare taxa (*e.g.*, singletons and doubletons), did not show a similarly strong effect, suggesting that rare taxa were not a major contributor to the observed diversity. Beta diversity analysis via Bray‒Curtis dissimilarity demonstrated significant differences in microbial composition among the three groups (Fig. 3E; ANOSIM R = 0.766, *p* = 0.001). Pairwise comparisons confirmed significant differences between the control and DSS 3-day groups (Bray‒Curtis: R = 0.936, *p* = 0.008) and between the control and DSS 6-day groups (Bray‒Curtis: R = 0.819, *p* = 0.007).

**Fig. 4:**
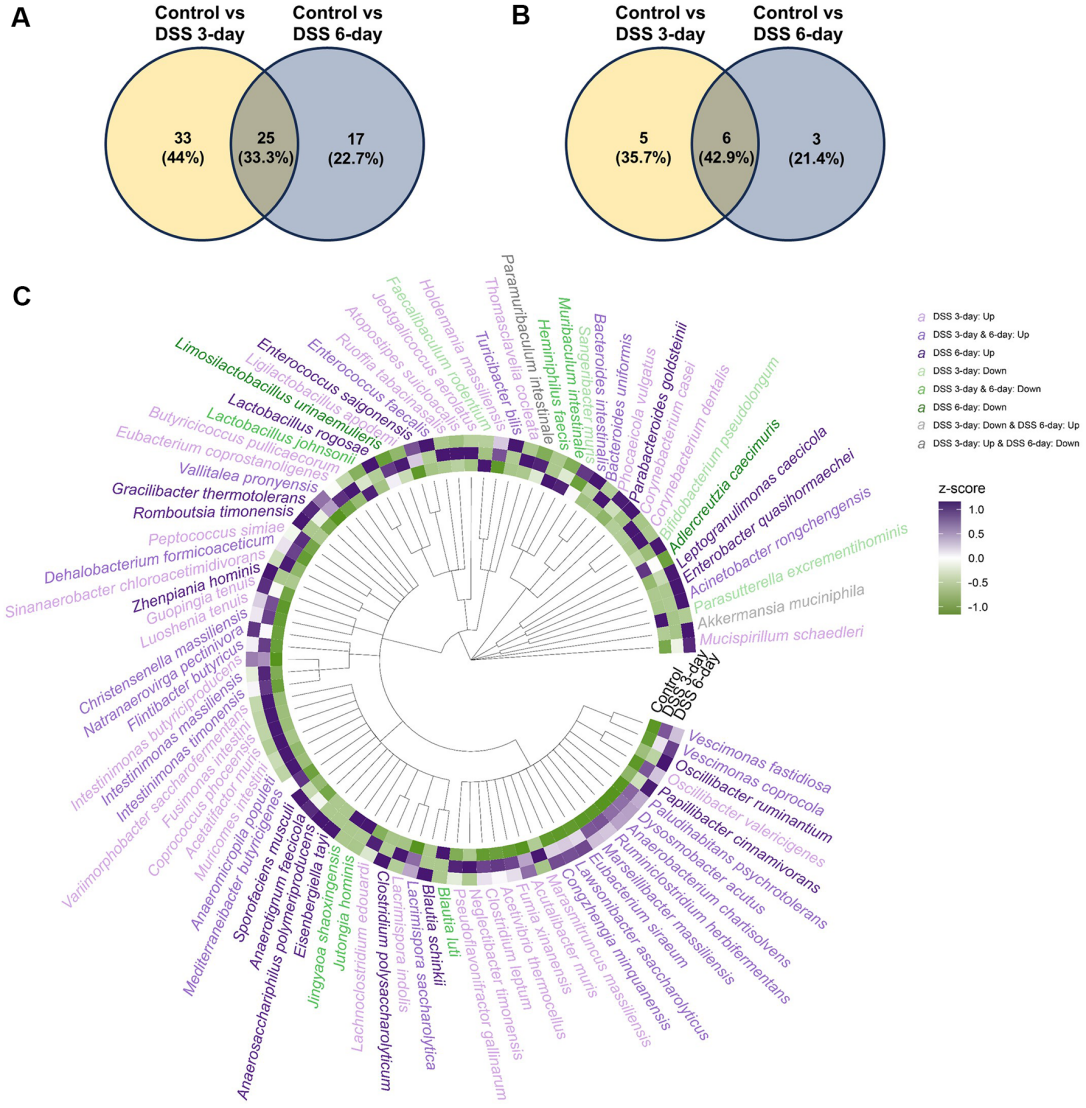
Identification of 87 differentially abundant microbes based on taxonomic profiles from 16S rRNA gene sequencing. (**A**) Venn diagram showing microbes with increased abundance in the 3- and 6-day DSS groups compared with the control group. (**B**) Venn diagram showing microbes with decreased abundance in the 3- and 6-day DSS groups compared with the control group. (**C**) Circular heatmap representing the relative abundances (z scores) of significantly altered microbes across the groups.

Taxonomic analysis and differential abundance testing revealed significant shifts at the phylum level. Notably, Pseudomonadota (*p* = 0.016, adjusted *p* = 0.024) and Verrucomicrobiota (p =0.032, adjusted *p* = 0.031) showed significantly increased relative abundance in the DSS 6-day group (Fig. 3F, Table S3). The abundance of Deferribacterota (p =0.036, adjusted *p* = 0.036) was transiently elevated on Day 3 but returned to control levels by Day 6. In contrast, the abundance of Bacillota (*p* = 0.016, adjusted *p* = 0.024) significantly decreased on Day 6. Fifty-eight genera were significantly different between the DSS 3-day group and the control group, and 37 genera were differentially abundant between the DSS 6-day group and the control group at the genus level (Fig. 3G, Table S3). *Acinetobacter* (*p* = 0.011, adjusted *p* = 0.033) and *Turicibacter* (*p* = 0.011, adjusted *p* = 0.033) abundances were notably increased, whereas *Lactobacillus* (*p* = 0.016, adjusted *p* = 0.033) and *Heminiphilus* (*p* = 0.015, adjusted *p* = 0.033) abundances progressively decreased following DSS induction. In total, 88 species presented differential abundances relative to those of the controls, with the majority increasing on Day 3. Among them, 25 taxa were consistently elevated, whereas six were consistently reduced across both DSS-treated groups (Fig. 4A and 4B). Interestingly, *Akkermansia muciniphila* was reduced on Day 3 (*p* = 0.025, adjusted *p* = 0.030) but exhibited a variable trend on Day 6. (*p* = 0.032, adjusted *p* = 0.044) Differentially abundant taxa did not show clear phylogenetic clustering (Fig. 4C), suggesting that the observed shifts were not restricted to specific microbial lineages.

### Changes in Microbial Gene Expression Following DSS Treatment

**Fig. 5:**
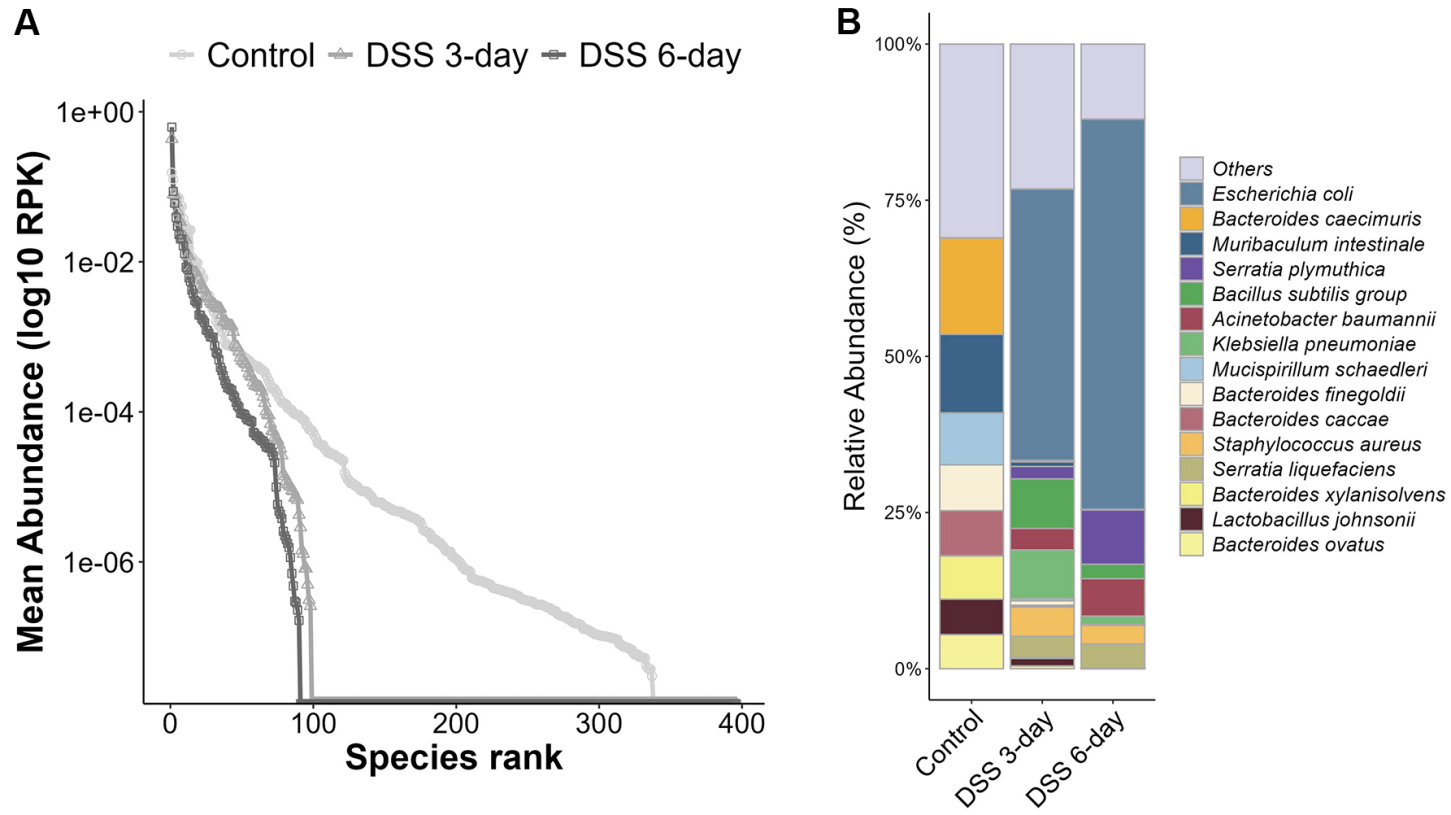
Microbial profiles detected from metatranscriptomic data. (**A**) Rank abundance plot showing average microbial expression levels across groups. (**B**) Taxonomic composition of microbes is represented as a stacked bar plot.

High-throughput sequencing generated an average of 13.3 Gbp of raw data, with 150 bp paired-end reads (Table S4). After adapter trimming and host sequence filtering, an average of 90.32% of the reads were retained in the control group, whereas only 39.85% and 35.9% of the reads remained in the 3- and 6-day DSS groups, respectively. Samples were assessed for RNA integrity using the DV200 index method, with all samples passing above 30%, indicating good quality (Table S4). Notably, despite identical experimental processing, a substantial proportion of sequences were classified as host-derived following DSS induction compared with the control group. Therefore, the low taxonomic assignments were not due to poor sample quality, but rather a high ratio of host sequences and the characteristic response to DSS treatment. This phenomenon was likely the result of the constant influx of immune cells into the gut environment caused by the disruption of tight junctions and increased intestinal permeability [68]. Taxonomic classification using Kraken2/Bracken revealed a markedly reduced number of microbial taxa in the DSS-treated groups, with particularly low classification rates in the DSS 6-day group. One sample (2_10) could not be assigned to any taxonomic group (Table S5). The low taxonomic classification results in the DSS-treated samples also impacted the downstream gene abundance estimation using HUMAnN3, leading to a substantial reduction in the number of microbial genes identified compared with those in the control group (Fig. 5A).

**Fig. 6:**
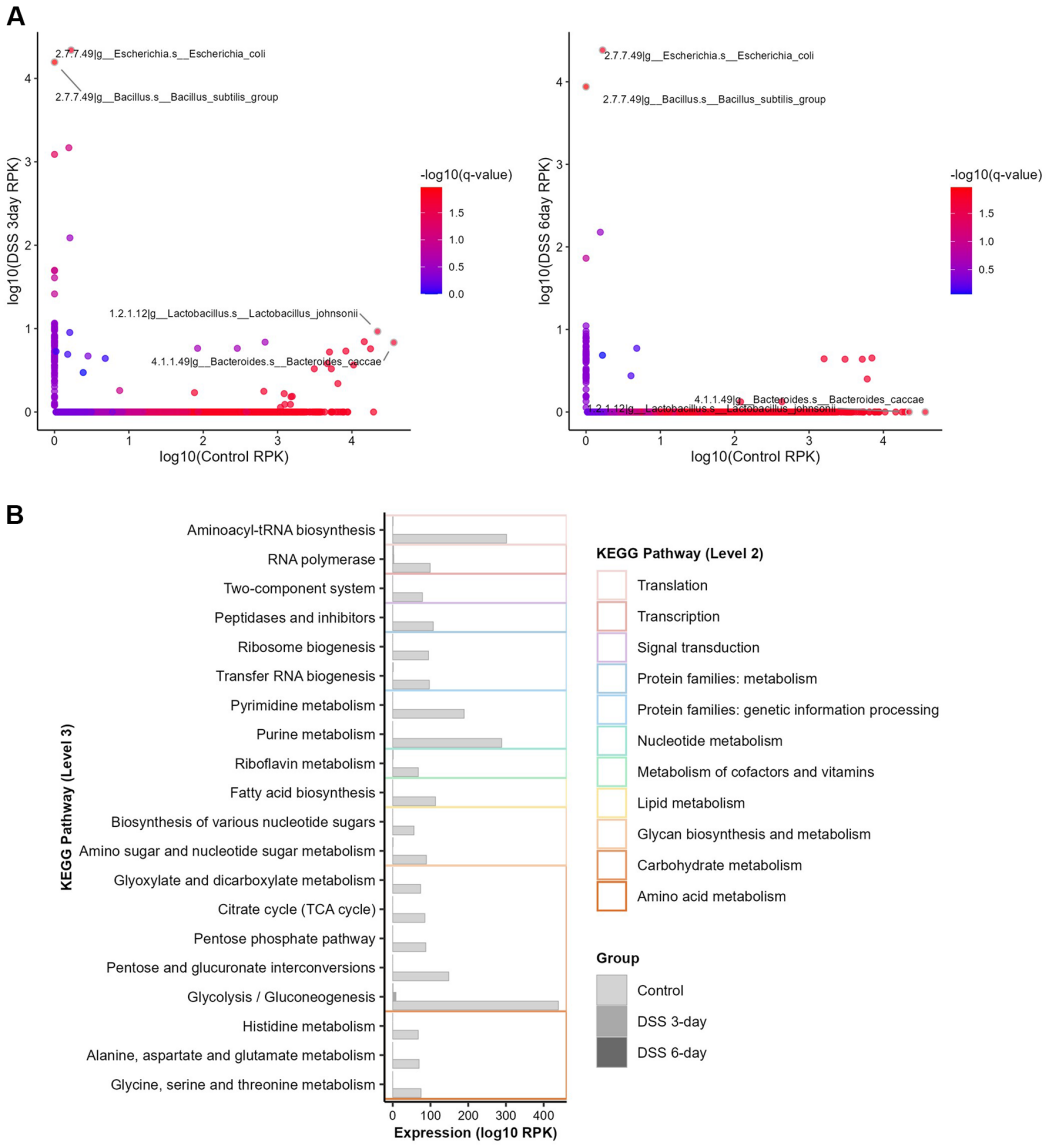
Differentially expressed microbial genes identified from metatranscriptomic data. (**A**) Microbial genes were differentially expressed between the control and DSS treated groups, with q values calculated using the Wilcoxon ranksum test with Benjamini-Hochberg FDR correction. Significantly differentially expressed genes are highlighted. (**B**) Total expression of microbial genes associated with each pathway, illustrating their biological functions based on KEGG pathway analysis.

To account for the taxonomic composition in microbial transcriptomic analyses conducted without a reference genome, taxon-specific scaling was applied to gene abundance (RPK) for normalization. Following DSS induction, transcript abundance exhibited widespread alterations across microbial taxa, with *Escherichia coli* being the most abundant species (Fig. 5B) and decrease of *Bacteroides* genus was observed. Our results showed that *E. coli* was the most transcriptionally abundant species, while its taxonomic abundance did not show significant changes. Lloyd-Price *et al*. reported that *E. coli* was significantly increased in IBD patients compared to non-IBD controls, and that *E. coli* was primarily involved in upregulating microbial enzymes under inflammatory conditions [17]. Alzahrani *et al*., also observed an increased abundance of *E. coli* and a decrease in the *Bacteroides* genus in the sigmoid colon microbiome of IBD patients [69]. A decrease in *Bacteroides* genus transcriptional activity was also notably observed in our results, which was consistent with the taxonomic decrease of *Bacteroides intestinalis* and *Bacteroides uniformis*. Statistical analysis revealed microbial transcripts that were significantly differentially expressed (Fig. 6A, Table S6). Differentially expressed genes (DEGs) were mapped to KEGG pathways to assess their potential functional contributions. A substantial proportion of these genes were associated with carbohydrate metabolism, nucleotide metabolism, and translation. Notably, the enriched pathways included glycolysis/gluconeogenesis, aminoacyl-tRNA biosynthesis, purine metabolism, and pyrimidine metabolism (Fig. 6B). These differentially expressed microbial transcripts were subsequently used for interaction analysis.

### Host Transcriptomic Responses to DSS-Induced Inflammation

**Fig. 7:**
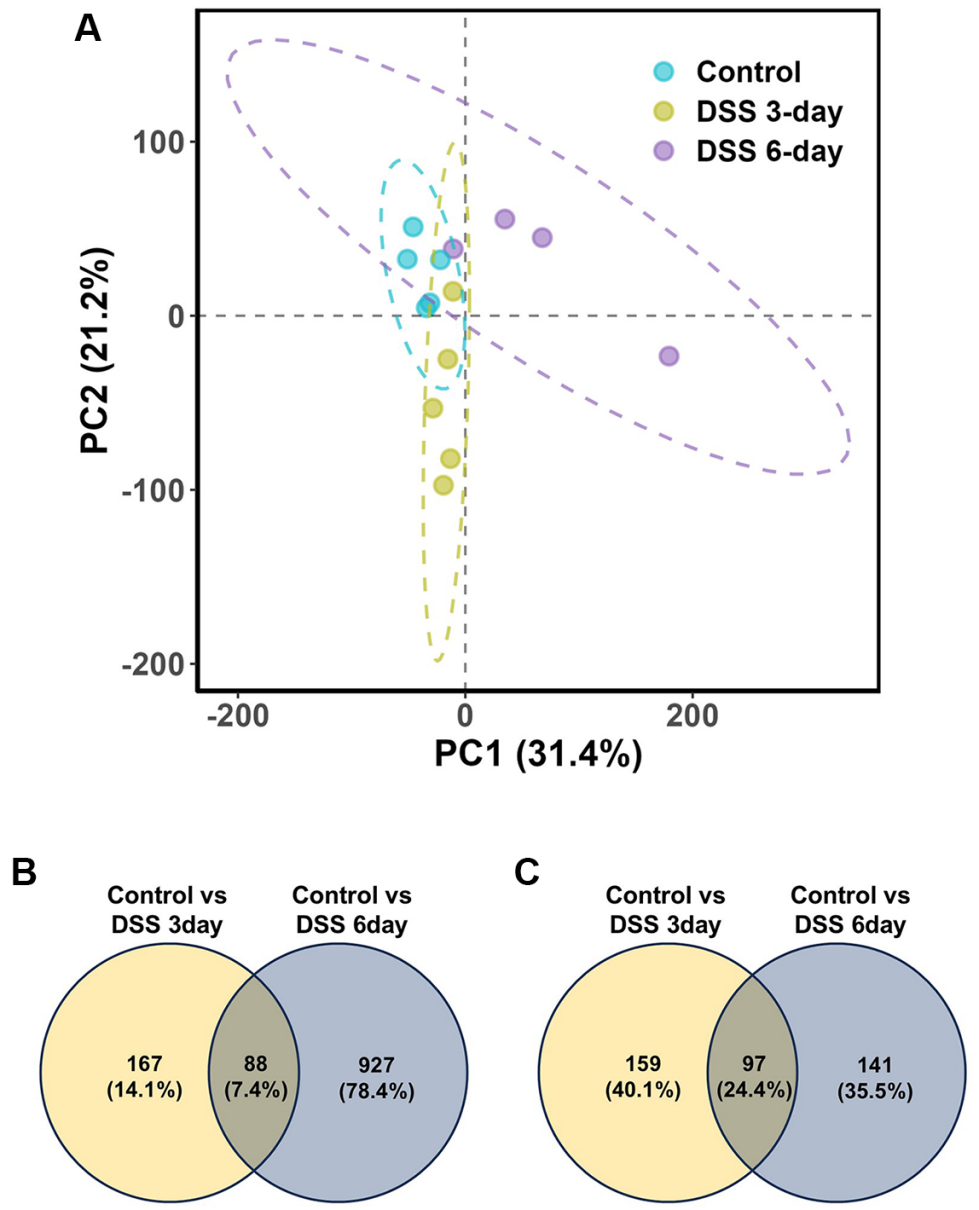
Differentially expressed genes (DEGs) identified via mouse host transcriptome analysis. (**A**) Principal component analysis (PCA) of overall gene expression profiles showing distinct clustering of DSS-treated samples compared with control samples, indicating altered host transcriptomic responses following DSS treatment. (**B**) Venn diagram comparing genes upregulated in the 3- and 6-day DSS groups relative to the control group. (**C**) Venn diagram comparing downregulated genes across the same groups.

On average, 6.1 Gbp of raw sequencing data were generated, and 957 Mbp of transcriptome data were successfully assembled (Tables S7 and S8). After removing redundant sequences and annotations, 11,518 transcripts were retained as representative. Across all samples, an average of 11,109 genes were expressed, with no significant difference in gene counts compared with controls (*p* = 0.095 for DSS 3-day; *p* = 0.556 for DSS 6-day). However, principal component analysis (PCA) revealed distinct clustering of the DSS-treated groups, indicating transcriptomic shifts following DSS induction (Fig. 7A).

**Fig. 8:**
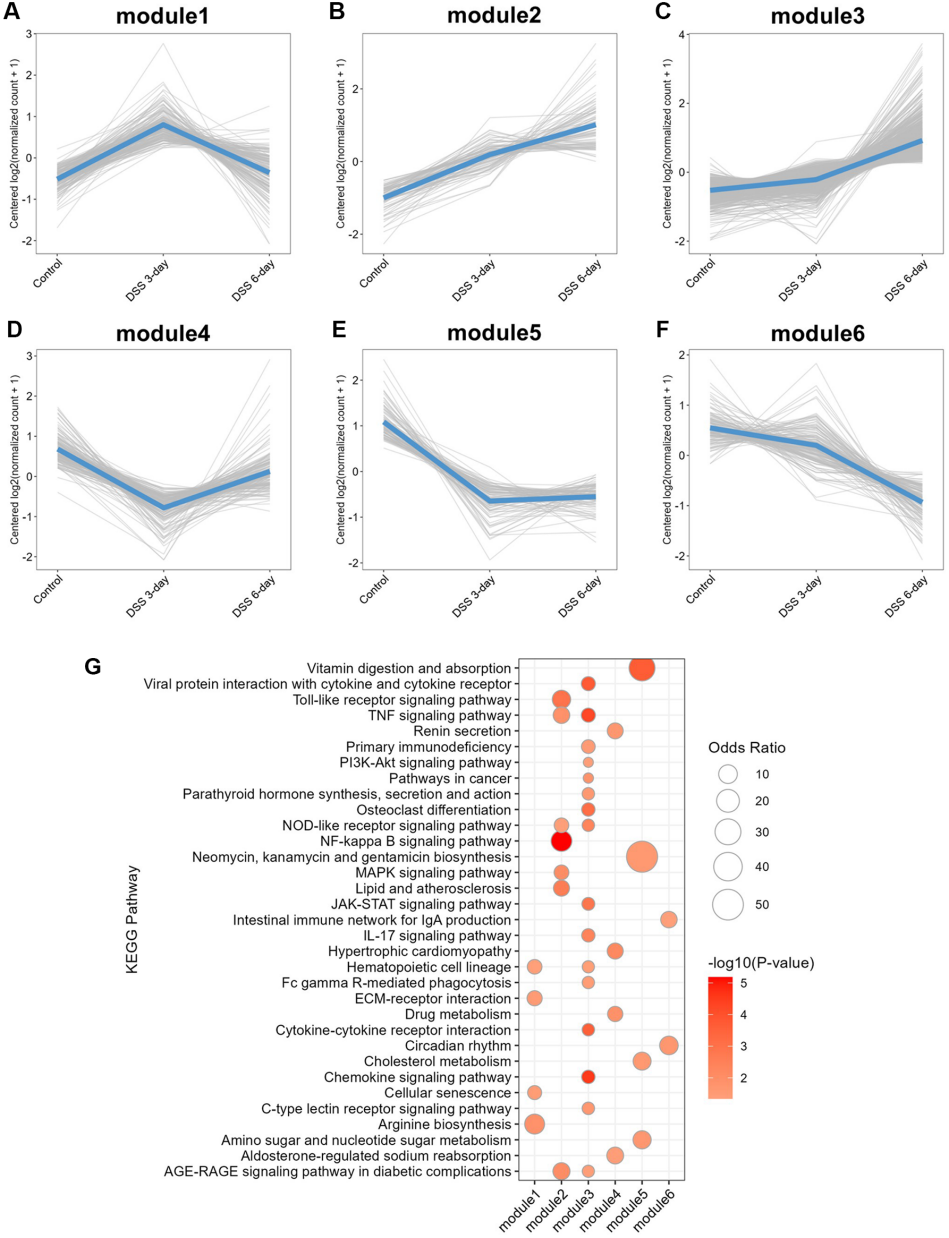
Functional analysis of DEGs from the host transcriptome. (**A**) Expression patterns of 1,571 DEGs grouped into six distinct modules based on their temporal dynamics across experimental conditions. (**B**) KEGG pathways enriched in each DEG module, highlighting key biological processes. Only pathways with an enrichment *p* value < 0.05 are shown.

To identify genes associated with DSS-induced changes, we compared the gene expression profiles of the 3- and 6-day DSS groups with those of the control group. A total of 1,571 DEGs were identified, which were categorized into six distinct expression patterns (Fig. 7B and 7C). Notably, many genes were markedly downregulated in the DSS 6-day group. To further investigate the biological significance of these patterns, DEGs were clustered into six gene modules based on their expression trends: (1) genes specifically upregulated at 3 days after DSS (module 1),(2) genes commonly upregulated in both DSS-treated groups (module 2), (3) genes upregulated exclusively at 6 days after DSS (module 3), (4) genes downregulated only at 3 days after DSS (module 4), (5) genes commonly downregulated across DSS-treated groups (module 5), and (6) genes downregulated exclusively at 6 days after DSS (module 6) (Fig. 8A-8F). All six modules were subjected to KEGG pathway enrichment analysis to assess their functional relevance (Fig. 8G). Notably, modules commonly or specifically responsive to DSS for 6 days (modules 2, 3, and 6) were enriched in immune-related pathways, including the Toll-like receptor signaling pathway, the TNF signaling pathway, the IL-17 signaling pathway, and the intestinal immune network for IgA production. Additionally, module 6 was enriched in circadian rhythm pathways. In contrast, the commonly downregulated module (module 5) was enriched in metabolic processes, such as amino sugar and nucleotide sugar metabolism and cholesterol metabolism regulation.

### Host‒Microbe Associations and Network Dynamics

**Fig. 9:**
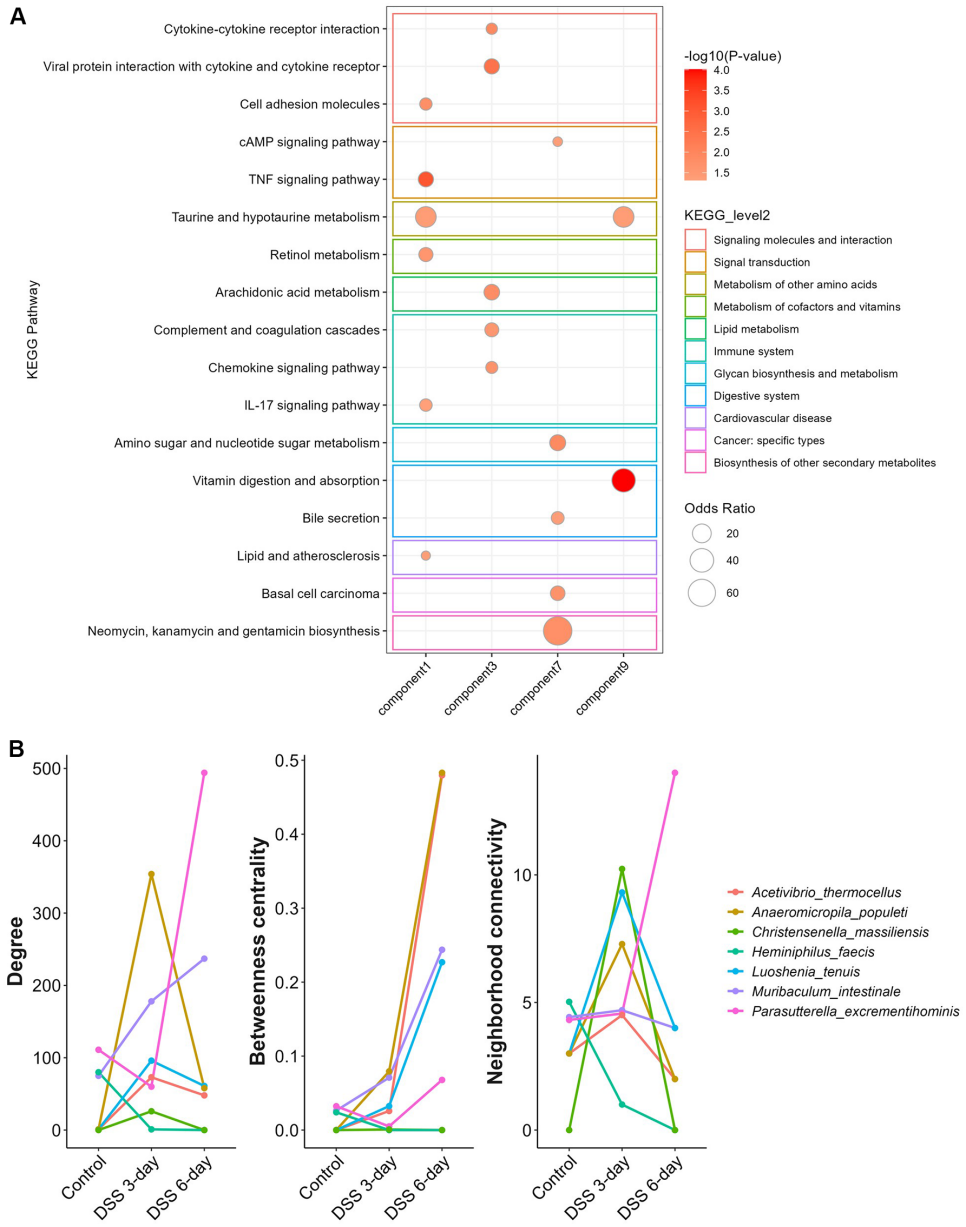
Host functional enrichment analysis of components identified by sCCA. (**A**) KEGG pathways enriched in host transcriptome features associated with microbial taxonomy components. Pathways with an enrichment *p* value < 0.05 are shown. These results provide insights into the host functions potentially interacting with microbes represented in each component. (**B**) Microbial taxa associated with sCCA components were evaluated for their network centrality using degree (number of direct connections), betweenness centrality (a measure of how often a node lies on the shortest path between other nodes, indicating its role as a central connector) and neighborhood connectivity (the average connectivity of a node’s neighbors, reflecting the extent to which connected nodes form local clusters). These indices highlight the potential importance of specific microbes as interaction hubs within host–microbe networks.

To explore host–microbe interactions following DSS induction, sparse canonical correlation analysis (sCCA) was performed using 88 differentially abundant microbial taxa and 1,253 host DEGs. Among the ten identified components, four showed statistically significant canonical correlations (Table S9). The associated microbes and their constituent host genes for each sCCA component are listed in Table S10. Component 1 included *Anaerosacchariphilus polymeriproducens* and *Enterobacter quasihormaechei*, whereas component 3 included *Parasutterella excrementihominis* and *Muribaculum intestinale*. Component 7 included *Christensenella massiliensis* and *Heminiphilus faecis*, and component 9 included *Acetivibrio thermocellus*, *Luoshenia tenuis*, and *Anaeromicropila populeti*. Pathway enrichment analysis of the host DEGs revealed that genes associated with components 1 and 3 were enriched primarily in immunological pathways (Fig. 9A). Specifically, component 1 was associated with TNF signaling, metabolism of amino acids and cofactors and vitamins, IL-17 signaling, and cell adhesion molecule pathways. Component 3 was significantly enriched in cytokine-related pathways and immune system, including viral protein interactions with cytokines and cytokine receptors, cytokine–cytokine receptor interactions, complement and coagulation cascades, chemokine signaling, and arachidonic acid metabolism. In contrast, component 7 was most enriched in the biosynthesis of other secondary metabolites, and component 9 was most enriched in digestive system and metabolism of other amino acids.

Network analysis further demonstrated extensive interactions between differentially abundant microbes and host DEGs. Microbial features robustly filtered by Spearman correlation are shown as network hubs. The number of edges and average node connectivity increased over time following DSS treatment, indicating increased interaction density and clustering within the host–microbe network (Table S12). The subnetwork modularity also shifted: eight distinct clusters were identified in the control group, five in the DSS 3-day group, and thirteen in the DSS 6-day group. These findings suggest that DSS-induced inflammation progressively restructures host–microbiome interactions, leading to more complex and concentrated interaction networks. Finally, seven hub microbes were identified based on network centrality measures, including degree centrality, betweenness centrality, and neighborhood connectivity (Fig. 9B). *A. thermocellus* and *A. populeti* from sCCA component 9 presented increased betweenness centrality on Day 6, suggesting that these taxa may serve as potential connectors or intermediaries within the host–microbiome interaction network, possibly linking otherwise unconnected host gene modules. *C. massiliensis* from sCCA component 7 presented a transient increase in neighborhood connectivity, indicating a potential role in local cluster formation. In contrast, *H. faecis* from sCCA component 7 presented reductions in degree and neighborhood connectivity following DSS induction, suggesting diminished interactions with host genes. *L. tenuis* from component 9 and *M. intestinale* from component 3 demonstrated increased betweenness centrality after DSS induction, supporting their potential roles as network connectors in inflammation-associated host responses. *P. excrementihominis* from component 3 formed new clusters and established associations with host genes exclusively after DSS induction, suggesting its context-specific relevance to disease progression.

### Correlations between Microbial Genes and Host Transcriptomic Profiles

Eight components were identified as being correlated with host and microbial features, among which six components exhibited statistically significant associations according to sCCA (Table S9). Pathway enrichment analysis revealed that components 5 and 6 were particularly enriched in signal transduction pathways related to immune functions (Table S11).

**Fig. 10:**
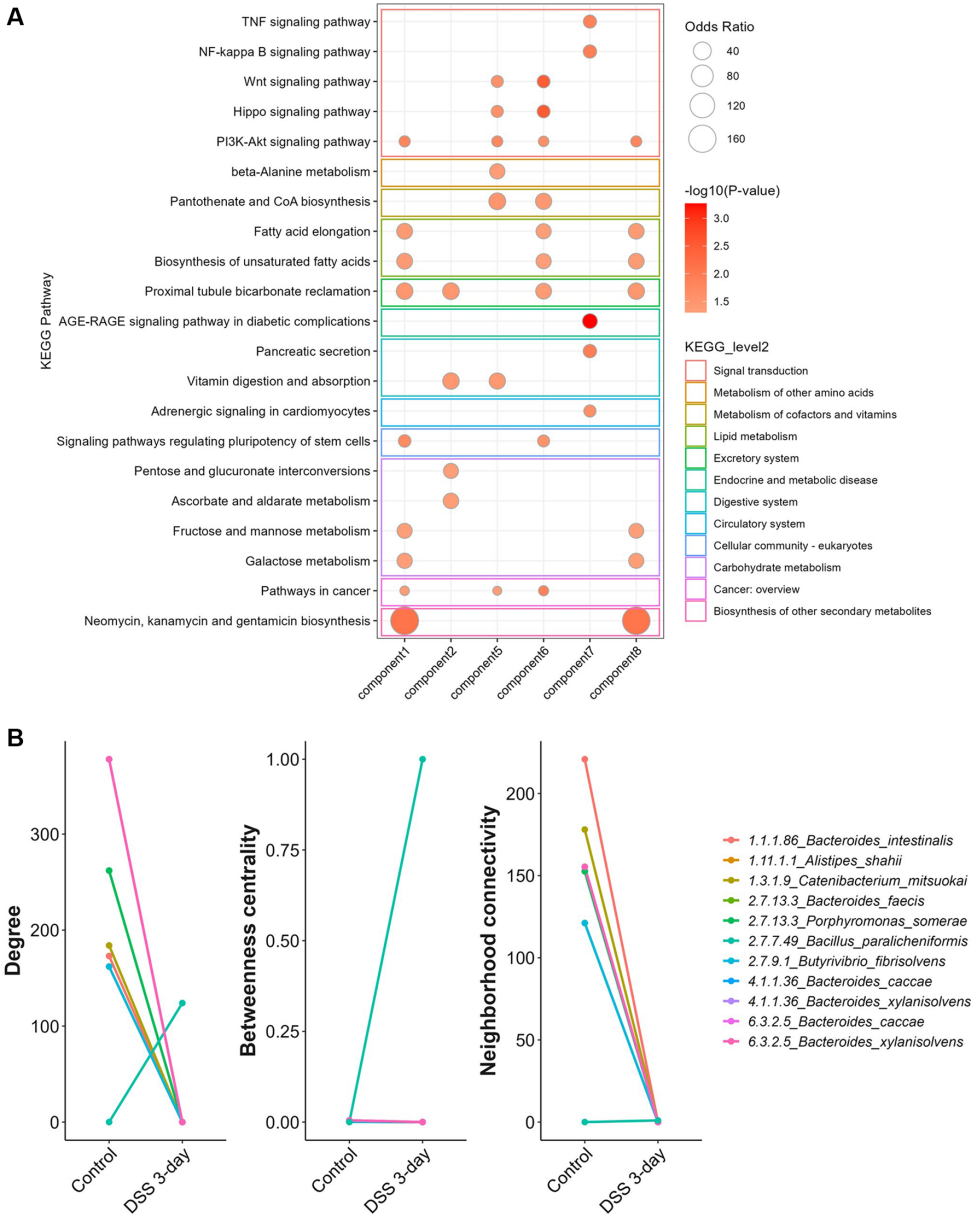
Network-based identification of microbial hubs that interact with host transcriptome features in sCCA components. (**A**) KEGG pathways enriched in host transcriptome features associated with microbial gene components. Pathways with an enrichment *p* value < 0.05 are shown. (**B**) Microbial genes from sCCA components were analyzed for their network centrality using degree, betweenness centrality,and neighborhood connectivity.

Components 1 and 2 included microbial genes involved in pantothenate and CoA biosynthesis (EC 4.1.1.36 and EC 6.3.2.5) from each *Bacteroides* genus. Although the microbial genes were the same, their contributions to host pathways differed (Fig. 10A). Component 5 contained NADH peroxidase (EC 1.11.1.1) from *Alistipes shahii* and pyruvate phosphate dikinase (EC 2.7.9.1) from *Butyrivibrio fibrisolvens*, whereas component 6 included histidine kinase (EC 2.7.13.3) from *Bacteroides faecis* and *Porphyromonas somerae*. These microbial genes were commonly associated with host pathways such as Wnt signaling, PI3K-Akt signaling, pantothenate and CoA biosynthesis, and the Hippo signaling pathway. Component 7 included reverse transcriptase (EC 2.7.7.49) from *Bacillus paralicheniformis* and *Klebsiella pneumoniae*, which were associated with TNF signaling, NF-κB signaling pathways, and endocrine and metabolic disease pathways known to be dysregulated in IBD patients [Tigas 2012]. Component 8 included genes related to pantothenate and CoA biosynthesis (EC 1.1.1.86) from *B. intestinalis* and fatty acid biosynthesis (EC 1.3.1.9) from *Catenibacterium mitsuokai*, which were associated with host lipid metabolism and the biosynthesis of secondary metabolites.

Network analysis revealed decreases in the number of interacting nodes, network density, and average number of neighbors in DSS-induced mice, indicating a weakening of microbial‒host gene interactions compared with those in the control group (Table S12). Among the hub microbes identified based on network properties, most microbial genes presented reduced centrality measures on Day 3. However, reverse transcriptase (EC 2.7.7.49) of *B. paralicheniformis* from component 7 showed the opposite trend, with increases in degree and betweenness centrality in the 3-day DSS samples, suggesting its potential role as an interaction hub in the altered network structure (Fig. 10B).

**Fig. 11:**
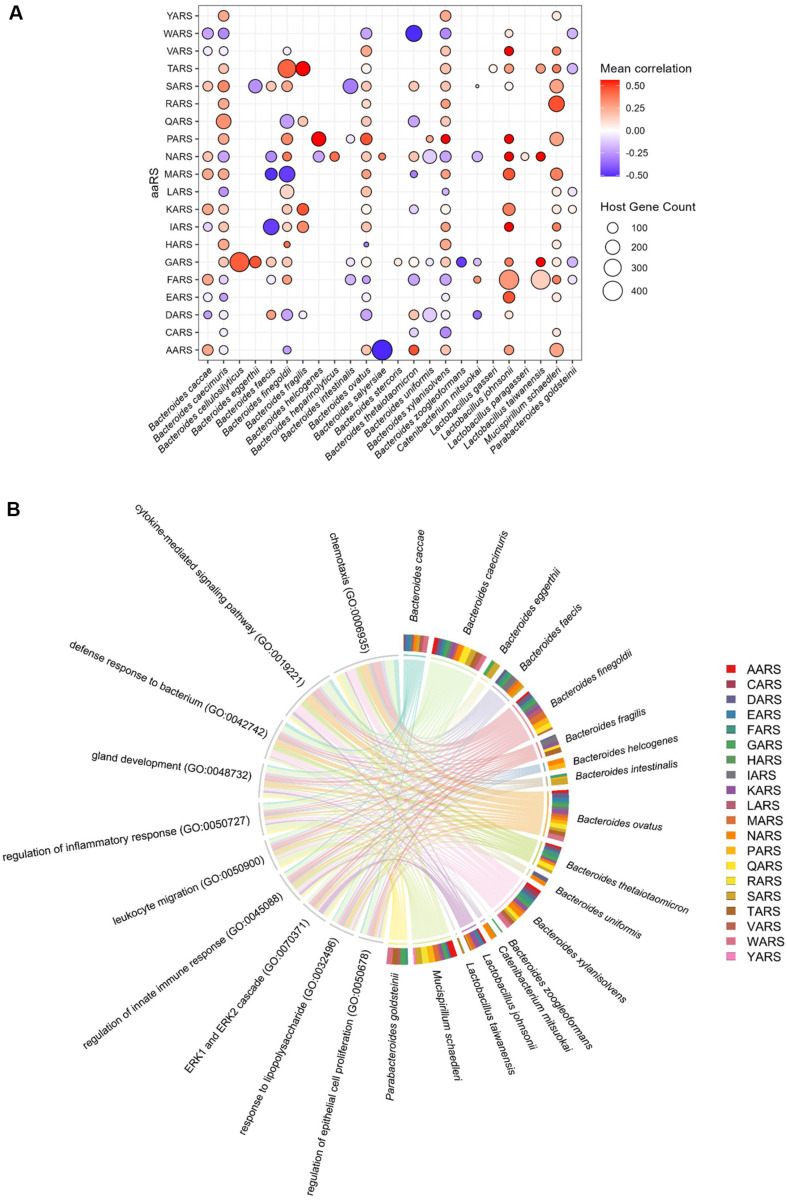
Associations between microbial aminoacyl-tRNA synthetases (aaRSs) and host genes. (**A**) aaRS genes from 24 microbial species associated with host genes are visualized along with their host gene counts and average correlation coefficients. (**B**) Gene Ontology (GO) biological process terms enriched in host genes linked to microbial aaRSs are shown. Each pie chart represents the relative aaRS gene contribution per microbe.

Bacterial aminoacyl-tRNA synthetases (aaRSs) have emerged as key bacterial components implicated in inflammatory processes and the modulation of the host immune system [24]. In our analysis, a substantial reduction in microbial genes associated with aminoacyl-tRNA biosynthesis was observed following DSS treatment, which is consistent with previous findings that aaRSs attenuate colitis. Given the marked decrease in bacterial gene expression in the DSS group, we specifically examined associations between aaRSs genes and host genes in control samples. A total of 173 aaRSs from 24 microbial species exhibited significant associations with host genes. Notably, *Bacteroides caecimuris*, *Bacteroides ovatus*, and *Bacteroides finegoldii* were the most prominent taxa, with the highest number of associations (Fig. 11A). The biological functions of the microbial aaRSs genes were further assessed (Fig. 11B). Network analysis revealed that aaRSs from 18 microbial species were involved in immunological processes, including chemotaxis (GO:0006935), cytokine-mediated signaling pathway (GO:0019221), defense response to bacterium (GO:0042742), regulation of the inflammatory response (GO:0050727), leukocyte migration (GO:0050900), regulation of the innate immune response (GO:0045088), and response to lipopolysaccharide (GO:0032496). In addition, microbial aaRSs genes were associated with the regulation of epithelial cell proliferation (GO:0050678), suggesting their potential involvement in epithelial defense mechanisms.

## Discussion

Timepoints were selected to represent intermediate (DSS 3-day) and confirmed inflammation stages (DSS 6-day). According to Park *et al*., mice treated with 2% DSS show elevated inflammatory biomarkers such as Hgb two days after DSS induction [70]. Early inflammatory timepoints reflect initial microbial and transcriptional changes. In our study, the DSS 3-day group showed mild inflammatory signs, including weight loss and reduced colon length, which are characteristic features of inflammation. Taxonomic assignment was more successful on Day 3, whereas metatranscriptomic data from Day 6 showed a marked decrease in detectable taxa. Consequently, most genes from the Day 6 dataset could not be reliably linked to bacterial information and were therefore excluded from subsequent integrative analyses.

In this study, we analyzed omics data from the colon and its contents. Kozik *et al*. and Abdel-Rahman *et al*., demonstrated the variability of bacterial diversity and disease susceptibility depending on the biopsy location (cecum, colonic mucus) or stool, both in inflammation mouse models and in IBD patients [71, 72]. Kozik *et al*., specifically highlighted that the colonic mucus microbiome was closely correlated with disease severity. While previous research has primarily focused on fecal samples in the DSS mouse model, Lloyd-Price *et al*., combined host transcriptome and metagenome data from human biopsies of IBD patients [17]. This study provides a unique approach to examining the colon microbiome in the DSS mouse model through the integration of three omics datasets, encompassing metagenome and host transcriptome data, to elucidate host–microbe interactions related to inflammatory gene responses. While it is well established that IBD patients exhibit a dysbiotic state and decreased gut diversity [17, 73], most of the preceding results have been primarily based on fecal samples from IBD patients. Recent studies have shown that stool and biopsy samples display different alpha diversity patterns, with stool showing a clear decrease in diversity, whereas biopsy samples exhibit variability in diversity [71]. In our study, the number of microbial taxa sampled from colonic contents was increased in the DSS-treated group, and alpha diversity indices, such as Shannon, inverse Simpson, and observed species, were higher in the DSS 6-day group compared to the control group. These findings are consistent with those of Park *et al*., who reported a slight but non-significant increase in diversity in the DSS group based on metagenomic samples collected from cecum tissue [70], and Alzahrani *et al*., who reported increased species richness in IBD patients from sigmoid colon tissue [69]. Moreover, microbial diversity can vary depending on sampling time points and DSS treatment protocol, reflecting differences in disease progression [70]. The microbial composition of the DSS-induced group was significantly altered, particularly on Day 6 postinduction compared with that of the control group. The increased abundances of Pseudomonadota, Verrucomicrobiota, and Deferribacterota in the DSS-treated group were consistent with previous findings [70]. Moreover, the differentially abundant taxa did not exhibit clear phylogenetic clustering, suggesting that microbial shifts may be shaped more by host-driven selection on microbial functional activities than by taxonomic identity. Among the significantly altered genera, *Turicibacter*, which is considerably more abundant in patients with ulcerative colitis (UC) and has been suggested as a potential biomarker [74], was detected. In addition, *Acinetobacter*, known to promote intestinal inflammation and reported to be elevated in patients with CD [75], was also present. Moreover, beneficial bacteria such as *Lactobacillus* and *Heminiphilus*, which contribute to epithelial barrier integrity and SCFA production [76, 77], were diminished. Microbiome studies from the DSS-induced colitis mouse model have yielded conflicting results due to variations in husbandry conditions, laboratory environments, and mouse characteristics. However, Khan *et al*. integrated metagenome data from different mouse types and identified common microbial taxa significantly associated with inflammation, such as an increase in Verrucomicrobiota, *Bifidobacterium*, *Akkermansia*, *Turicibacter*, and *Acinetobacter*, along with a decrease in *Lactobacillus* and *Adlercreutzia* [78]. These findings are consistent with our own metagenomic results.

To understand the contribution of microbial genes to the inflammatory environment in DSS-induced colitis, the DEGs of microbes and their potential roles were examined. Our results revealed that transcripts of *E. coli* and *Klebsiella pneumoniae*, microbes known to be prevalent in patients with UC and capable of inducing inflammation, were dominant in the DSS group [79-81]. Gene expression from the *Bacillus subtilis* group, which is known to increase tight junction protein expression and anti-inflammatory responses, was notably increased on Day 3 [82, 83]. In response to IBD, the phageome undergoes significant changes, with temperate phages replacing lytic phages and overrepresentation of microbiome-infecting phages [84, 85]. Reverse transcriptase (EC 2.7.7.49), a known anti-phage defense component [86], was upregulated in both *E. coli* and *B. subtilis*, suggesting a possible bacterial survival mechanism in response to host immune pressure. Among the microbial genes that were significantly downregulated following DSS induction, many were involved in carbohydrate metabolism, nucleotide metabolism, and aminoacyl-tRNA biosynthesis. These functional categories are fundamental to microbial growth and survival, and their suppression may reflect a shift in microbial activity under inflammatory conditions.

In the host gene analysis, prominent upregulation of colonic transcripts was observed over time. The upregulated modules in the DSS group (modules 2 and 3), identified via DEG analysis, were involved in immunological functions related to proinflammatory responses. Moreover, unusual features were observed in the downregulated modules: module 5 was involved in cholesterol metabolism, which is consistent with the observation of lower cholesterol levels in patients with IBD [87], whereas module 6 was associated with the circadian cycle, which aligns with previous studies indicating differential expression of circadian genes in patients with IBD [88].

With respect to the associations between taxonomic abundance and host genes, *Anaerosacchariphilus polymeriproducens* and *Enterobacter quasihormaechei* from sCCA component 1 were linked to host immunomodulatory pathways, including the TNF signaling, IL-17 signaling, and cell adhesion molecule pathways. Notably, *Enterobacter* bacteria have been implicated in the pathogenesis of IBD [89]. Both microbes also influence host retinol metabolism, as revealed by pathway enrichment analysis, which has been implicated in IBD pathophysiology through the retinoic acid-metabolizing enzyme CYP26B1, which is known to play a role in T-cell development [90]. While *E. quasihormaechei* exhibited weak correlations with individual host genes, *A. polymeriproducens* showed high centrality in the Day 6 network, suggesting that *E. quasihormaechei* may indirectly contribute to immunomodulation by interacting with highly connected microbial hubs. Similarly, *Parasutterella excrementihominis* and *Muribaculum intestinale* from component 3 were associated with multiple cytokine-related pathways, including interactions with cytokines and cytokine receptors, cytokine–cytokine receptor interactions, complement and coagulation cascades, chemokine signaling, and arachidonic acid metabolism. *Parasutterella* were identified as hub microbes based on their elevated centrality and neighborhood connectivity on Day 6, indicating an increased potential to influence host immune networks. *Parasutterella* has previously been associated with altered abundance in individuals with irritable bowel syndrome and chronic intestinal inflammation [91], whereas *Muribaculum* has been shown to induce the production of proinflammatory cytokines such as TNF-α, IL-6, and IL-23 through its metabolites [92]. These findings highlight that sCCA components can capture both direct gene correlations and network-mediated indirect interactions, emphasizing stage-specific host–microbe communication.

Integration of microbial functional genes with host pathways further demonstrated that NADH peroxidase from *Alistipes shahii*, pyruvate phosphate dikinase from *Butyrivibrio fibrisolvens*, and histidine kinases from *Bacteroides faecis* and *Porphyromonas somerae* were commonly associated with host immunological pathways, exhibiting high network connectivity. NADH peroxidase is known to play a crucial role in microbial oxidative stress management and biofilm formation [93, 94], while pyruvate phosphate dikinase catalyzes ATP conversion to AMP and PEP, linking energy metabolism to host oxidative stress [95-97]. Histidine phosphorylation has been predominantly observed in the intestinal epithelial cells of both patients with IBD and DSS-induced mice, suggesting its contribution in ulcerative colitis. The high centrality and connectivity of histidine kinases from *B. faecis* and *P. somerae* indicate that they may act as keystone nodes, coordinating interactions across multiple host immune pathways. *A. shahii* has been implicated in proinflammatory cytokine modulation [98], and *B. fibrisolvens* contributes to epithelial barrier integrity through butyrate production [99], while *B. faecis* has been reported to modulate regulatory T cells [100, 101]. In contrast, *P. somerae* has not been previously discussed in the context of IBD but has been identified as a biomarker in endometrial cancer [102], suggesting potential yet unexplored roles in intestinal inflammation. By integrating these two layers, we identified key hub microbes that may mediate host immune pathways, reflecting dynamic, stage-specific host–microbiome interactions. This approach underscores the importance of considering both direct correlations and network-mediated interactions to elucidate functional host–microbe relationships in the context of intestinal inflammation.

Moreover, microbial aaRSs have recently been shown to contribute to anti-inflammatory mechanisms through IL-10 production [24]. Because most interactions between bacterial and host genes were lost under the DSS-induced conditions in our analysis, we profiled the associations between aaRSs and host genes under healthy conditions. Most differentially expressed microbial aaRSs were associated with host immune pathways and the regulation of epithelial cell proliferation, a process crucial for epithelial homeostasis and involved in IBD pathogenesis [103], suggesting a potential role for microbial aaRSs in promoting mucosal repair and regeneration, although further experimental validation is needed to confirm these findings.

There were some integrative analyses employing metatranscriptome set in IBD and inflammation research. Schirmer *et al*., analyzed both metagenomes and metatranscriptomes in IBD patients, emphasizing that microbial gene expression dynamics, rather than simply genomic abundance, are key to understanding the mechanisms of the disease [37]. Their findings specifically highlight how disease-specific microbial characteristics, such as *Bacteroides vulgatus* and *Alistipes putredinis*, along with their transcriptional activity, vary between patients and are essential for interpreting the functional role of the microbiome in IBD. Lloyd-Price *et al*. analyzed a comprehensive and longitudinal omics dataset, including amplicon sequencing, metagenome, metatranscriptome, metabolome, host transcriptome, metaproteome, and virome, derived from stool, biopsy, and blood samples of IBD patients [17]. They demonstrated significant disruptions in the microbiome during disease activity, with alterations in microbial transcription and shifts in metabolite pools. Additionally, they established an integrative interaction network using a mixed-effects model, where *E. coli* accounted for a large fraction of upregulated enzymes. Arehart *et al*., integrated metagenome, metatranscriptome, metabolome, and virome data from IBD patients to develop a prediction model for IBD diagnosis [20]. In the DSS-induced colitis mouse model, Jovel *et al*., analyzed and compared the results of metagenome and metatranscriptome data, demonstrating the feasibility of using metagenomics to assess the metabolic responses of the microbiome under inflammatory conditions [38]. However, the importance of aaRSs in host-microbe interactions was not reported in previous studies. In this study, aminoacyl-tRNA biosynthesis was significantly decreased in microbial transcripts following DSS treatment. Furthermore, the association between aaRSs and host immune pathways in a healthy state represents a novel observation in the field. Microbes and microbial genes identified through our analysis may have preclinical potential as biomarkers for host inflammatory status, as targets for microbiome-based interventions, or as candidates for mechanistic studies to investigate host–microbe interactions. Furthermore, we identified putative microbial aaRSs significantly associated with host immune responses, and these novel candidates could serve as potential therapeutic targets for IBD. Our results revealed contrasting findings in that bacterial taxonomy was highly diverse, whereas bacterial transcripts were rarely expressed under DSS conditions. Further investigation is needed to elucidate this phenomenon; however, several possible explanations can be suggested. First, predominant microbes in a healthy state may decrease as the gut environment changes, whereas relatively low-abundance microbes may temporarily increase. Second, if microbes are in a dormant or dead state, gene expression would be minimal, and only surviving microbes would produce transcripts, whereas 16S rDNA could still be detected owing to its slow degradation. Third, host‒microbiome interactions play crucial roles in microbial activation [104, 105]. After DSS induction, these interactions were severely diminished, potentially leading to bacterial inactivation. This reduction in interaction may contribute to the observed decline in microbial transcript expression, despite the presence of diverse bacterial taxa. As observed in the network analysis, host‒microbiome interactions were concentrated on specific microbes, in contrast to the healthy condition. The gut environment in the DSS-induced colitis mouse model, which is maintained under sterile conditions, may be particularly vulnerable to DSS stimulation, which could further exacerbate microbial dysbiosis. Previous studies have shown that microbial transcriptional activity does not always align with metagenomic abundance, and metatranscriptomic analysis is better suited to capture temporal changes in the DSS-induced colitis mouse model [37, 38]. Therefore, it is not surprising that transcript activity is discordant with metagenome data.

In this study, multimodal aspects of IBD, encompassing microbial dynamics, genetic alterations in the microbiome, and corresponding host immune responses, were characterized using an omics approach. The integrative analysis provided insights into the complex networks between the host immune system and the gut microbiota in a DSS-induced colitis mouse model. As a result, host immune-related bacteria were identified, and gene-level host–microbe associations, including microbial aaRSs components, which are potentially crucial for immune modulation, were markedly diminished following DSS induction, as revealed by integrative analysis. These findings underscore the critical role of transcriptional activity of microbial genes in shaping host immunity and offer a valuable foundation for future IBD research focusing on host–microbiome interactions.

## Supplemental Materials

Supplementary data for this paper are available on-line only at http://jmb.or.kr.
