# Supplementary material for: Multiomics Integration Reveals Microbial Gene Interactions Shaping Host Responses in a DSS-Induced Colitis Mouse Model
Source: J Microbiol Biotechnol. 2025 Oct 15;35:e2507010. doi: 10.4014/jmb.2507.07010 (PMC12549230; doi:10.4014/jmb.2507.07010)
Supplement: Supplementary file 1 [file jmb-35-e2507010-supple.pdf]

Table S6. Differentially expressed microbial genes identified by the Wilcoxon rank-sum test.

Group	EC	Gene
Control vs DSS 3-day	2.7.7.49	RNA-directed DNA polymerase
Control vs DSS 3-day	2.7.7.49	RNA-directed DNA polymerase
Control vs DSS 3-day	2.7.7.6	DNA-directed RNA polymerase
Control vs DSS 3-day	4.1.1.49	Phosphoenolpyruvate carboxykinase (ATP)
Control vs DSS 3-day	2.7.7.6	DNA-directed RNA polymerase
Control vs DSS 3-day	4.1.1.11	Aspartate 1-decarboxylase
Control vs DSS 3-day	4.1.1.49	Phosphoenolpyruvate carboxykinase (ATP)
Control vs DSS 3-day	2.7.7.6	DNA-directed RNA polymerase
Control vs DSS 3-day	4.1.1.49	Phosphoenolpyruvate carboxykinase (ATP)
Control vs DSS 3-day	2.7.7.6	DNA-directed RNA polymerase
Control vs DSS 3-day	4.1.1.49	Phosphoenolpyruvate carboxykinase (ATP)
Control vs DSS 3-day	4.1.1.49	Phosphoenolpyruvate carboxykinase (ATP)
Control vs DSS 3-day	2.7.7.6	DNA-directed RNA polymerase
Control vs DSS 3-day	4.1.1.49	Phosphoenolpyruvate carboxykinase (ATP)
Control vs DSS 3-day	2.7.7.6	DNA-directed RNA polymerase
Control vs DSS 3-day	4.1.1.49	Phosphoenolpyruvate carboxykinase (ATP)
Control vs DSS 3-day	2.7.7.6	DNA-directed RNA polymerase
Control vs DSS 3-day	4.1.1.49	Phosphoenolpyruvate carboxykinase (ATP)
Control vs DSS 3-day	4.1.1.49	Phosphoenolpyruvate carboxykinase (ATP)
Control vs DSS 3-day	1.2.1.12	Glyceraldehyde-3-phosphate dehydrogenase (phosphorylating)
Control vs DSS 3-day	4.1.1.49	Phosphoenolpyruvate carboxykinase (ATP)
Control vs DSS 3-day	2.7.7.49	RNA-directed DNA polymerase
Control vs DSS 3-day	2.7.7.49	RNA-directed DNA polymerase
Control vs DSS 3-day	1.4.1.3	Glutamate dehydrogenase (NAD(P)(+))
Control vs DSS 3-day	3.6.5.n1	Elongation factor 4
Control vs DSS 3-day	6.3.5.1	NAD(+) synthase (glutamine-hydrolyzing)
Control vs DSS 3-day	4.1.1.36	Phosphopantothenoylcysteine decarboxylase
Control vs DSS 3-day	4.2.1.47	GDP-mannose 4,6-dehydratase
Control vs DSS 3-day	6.3.2.5	Phosphopantothenate--cysteine ligase
Control vs DSS 3-day	6.1.1.3	Threonine--tRNA ligase
Control vs DSS 3-day	1.6.5.3	NADH
Control vs DSS 3-day	1.6.99.5	Transferred entry
Control vs DSS 3-day	1.97.1.4	[Formate-C-acetyltransferase]-activating enzyme
Control vs DSS 3-day	2.1.1.163	Demethylmenaquinone methyltransferase
Control vs DSS 3-day	2.5.1.17	Cob(I)yrinic acid a,c-diamide adenosyltransferase
Control vs DSS 3-day	6.5.1.2	DNA ligase (NAD(+))
Control vs DSS 3-day	1.17.7.4	NO_NAME
Control vs DSS 3-day	2.7.8.13	Phospho-N-acetylmuramoyl-pentapeptide-transferase
Control vs DSS 3-day	6.1.1.7	Alanine--tRNA ligase
Control vs DSS 3-day	2.7.2.4	Aspartate kinase
Control vs DSS 3-day	2.1.1.199	16S rRNA (cytosine(1402)-N(4))-methyltransferase

Control vs DSS 3-day	4.1.1.36	Phosphopantothenoylcysteine decarboxylase
Control vs DSS 3-day	6.3.2.5	Phosphopantothenate--cysteine ligase
Control vs DSS 3-day	2.3.1.180	Beta-ketoacyl-[acyl-carrier-protein] synthase III
Control vs DSS 3-day	3.6.5.n1	Elongation factor 4
Control vs DSS 3-day	4.1.1.19	Arginine decarboxylase
Control vs DSS 3-day	2.7.2.4	Aspartate kinase
Control vs DSS 3-day	1.4.1.3	Glutamate dehydrogenase (NAD(P)(+))
Control vs DSS 3-day	1.97.1.4	[Formate-C-acetyltransferase]-activating enzyme
Control vs DSS 3-day	3.3.1.1	Adenosylhomocysteinase
Control vs DSS 3-day	3.5.1.10	Formyltetrahydrofolate deformylase
Control vs DSS 3-day	4.2.1.33	3-isopropylmalate dehydratase
Control vs DSS 3-day	4.2.1.8	Mannonate dehydratase
Control vs DSS 3-day	2.8.1.8	Lipoyl synthase
Control vs DSS 3-day	5.1.1.7	Diaminopimelate epimerase
Control vs DSS 3-day	1.11.1.15	Peroxiredoxin
Control vs DSS 3-day	1.4.1.16	Diaminopimelate dehydrogenase
Control vs DSS 3-day	2.5.1.72	Quinolinate synthase
Control vs DSS 3-day	1.1.1.205	IMP dehydrogenase
Control vs DSS 3-day	1.1.1.267	1-deoxy-D-xylulose-5-phosphate reductoisomerase
Control vs DSS 3-day	2.5.1.7	UDP-N-acetylglucosamine 1-carboxyvinyltransferase
Control vs DSS 3-day	3.4.21.107	Peptidase Do
Control vs DSS 3-day	5.3.1.14	L-rhamnose isomerase
Control vs DSS 3-day	2.2.1.6	Acetolactate synthase
Control vs DSS 3-day	2.5.1.15	Dihydropteroate synthase
Control vs DSS 3-day	2.7.8.13	Phospho-N-acetylmuramoyl-pentapeptide-transferase
Control vs DSS 3-day	3.6.3.31	Polyamine-transporting ATPase
Control vs DSS 3-day	5.1.3.3	Aldose 1-epimerase
Control vs DSS 3-day	6.1.1.21	Histidine--tRNA ligase
Control vs DSS 3-day	1.4.1.3	Glutamate dehydrogenase (NAD(P)(+))
Control vs DSS 3-day	2.4.2.14	Amidophosphoribosyltransferase
Control vs DSS 3-day	3.1.3.11	Fructose-bisphosphatase
Control vs DSS 3-day	3.4.21.105	Rhomboid protease
Control vs DSS 3-day	3.5.1.88	Peptide deformylase
Control vs DSS 3-day	3.6.4.13	RNA helicase
Control vs DSS 3-day	4.3.1.17	L-serine ammonia-lyase
Control vs DSS 3-day	4.2.1.2	Fumarate hydratase
Control vs DSS 3-day	4.2.1.46	dTDP-glucose 4,6-dehydratase
Control vs DSS 3-day	1.1.1.94	Glycerol-3-phosphate dehydrogenase (NAD(P)(+))
Control vs DSS 3-day	1.4.1.21	Aspartate dehydrogenase
Control vs DSS 3-day	2.3.1.29	Glycine C-acetyltransferase
Control vs DSS 3-day	2.7.4.25	(d)CMP kinase
Control vs DSS 3-day	3.2.2.n1	Cytokinin riboside 5'-monophosphate phosphoribohydrolase
Control vs DSS 3-day	3.5.1.10	Formyltetrahydrofolate deformylase

Control vs DSS 3-day	6.1.1.21	Histidine--tRNA ligase
Control vs DSS 3-day	6.1.1.22	Asparagine--tRNA ligase
Control vs DSS 3-day	3.6.5.n1	Elongation factor 4
Control vs DSS 3-day	2.7.1.33	Pantothenate kinase
Control vs DSS 3-day	5.3.1.14	L-rhamnose isomerase
Control vs DSS 3-day	1.2.1.11	Aspartate-semialdehyde dehydrogenase
Control vs DSS 3-day	6.1.1.15	Proline--tRNA ligase
Control vs DSS 3-day	6.3.5.3	Phosphoribosylformylglycinamidine synthase
Control vs DSS 3-day	1.1.1.267	1-deoxy-D-xylulose-5-phosphate reductoisomerase
Control vs DSS 3-day	2.8.1.8	Lipoyl synthase
Control vs DSS 3-day	4.2.1.10	3-dehydroquinate dehydratase
Control vs DSS 3-day	4.2.2.2	Pectate lyase
Control vs DSS 3-day	5.1.3.32	L-rhamnose mutarotase
Control vs DSS 3-day	1.1.1.100	3-oxoacyl-[acyl-carrier-protein] reductase
Control vs DSS 3-day	1.4.1.3	Glutamate dehydrogenase (NAD(P)(+))
Control vs DSS 3-day	6.1.1.11	Serine--tRNA ligase
Control vs DSS 3-day	1.1.1.157	3-hydroxybutyryl-CoA dehydrogenase
Control vs DSS 3-day	3.4.22.40	Bleomycin hydrolase
Control vs DSS 3-day	1.1.1.28	D-lactate dehydrogenase
Control vs DSS 3-day	3.1.13.1	Exoribonuclease II
Control vs DSS 3-day	2.1.1.72	Site-specific DNA-methyltransferase (adenine-specific)
Control vs DSS 3-day	2.1.3.3	Ornithine carbamoyltransferase
Control vs DSS 3-day	2.1.4.1	Glycine amidinotransferase
Control vs DSS 3-day	3.5.3.6	Arginine deiminase
Control vs DSS 3-day	3.1.21.3	Type I site-specific deoxyribonuclease
Control vs DSS 3-day	1.11.1.1	NADH peroxidase
Control vs DSS 3-day	1.3.5.1	Succinate dehydrogenase (quinone)
Control vs DSS 3-day	1.3.99.1	Deleted entry
Control vs DSS 3-day	2.7.7.6	DNA-directed RNA polymerase
Control vs DSS 3-day	2.7.9.1	Pyruvate, phosphate dikinase
Control vs DSS 3-day	3.4.11.18	Methionyl aminopeptidase
Control vs DSS 3-day	4.1.1.41	Methylmalonyl-CoA decarboxylase
Control vs DSS 3-day	4.1.1.49	Phosphoenolpyruvate carboxykinase (ATP)
Control vs DSS 3-day	4.1.99.1	Tryptophanase
Control vs DSS 3-day	4.1.99.2	Tyrosine phenol-lyase
Control vs DSS 3-day	5.4.99.2	Methylmalonyl-CoA mutase
Control vs DSS 3-day	6.4.1.3	Propionyl-CoA carboxylase
Control vs DSS 3-day	1.11.1.1	NADH peroxidase
Control vs DSS 3-day	1.3.5.1	Succinate dehydrogenase (quinone)
Control vs DSS 3-day	1.3.99.1	Deleted entry
Control vs DSS 3-day	2.7.13.3	Histidine kinase
Control vs DSS 3-day	2.7.7.6	DNA-directed RNA polymerase
Control vs DSS 3-day	2.7.9.1	Pyruvate, phosphate dikinase

Control vs DSS 3-day	4.1.1.41	Methylmalonyl-CoA decarboxylase
Control vs DSS 3-day	4.1.1.49	Phosphoenolpyruvate carboxykinase (ATP)
Control vs DSS 3-day	4.1.99.1	Tryptophanase
Control vs DSS 3-day	4.1.99.2	Tyrosine phenol-lyase
Control vs DSS 3-day	5.4.99.2	Methylmalonyl-CoA mutase
Control vs DSS 3-day	6.4.1.3	Propionyl-CoA carboxylase
Control vs DSS 3-day	1.11.1.1	NADH peroxidase
Control vs DSS 3-day	1.3.5.1	Succinate dehydrogenase (quinone)
Control vs DSS 3-day	1.3.99.1	Deleted entry
Control vs DSS 3-day	2.7.13.3	Histidine kinase
Control vs DSS 3-day	2.7.7.6	DNA-directed RNA polymerase
Control vs DSS 3-day	2.7.9.1	Pyruvate, phosphate dikinase
Control vs DSS 3-day	3.4.11.18	Methionyl aminopeptidase
Control vs DSS 3-day	4.1.1.41	Methylmalonyl-CoA decarboxylase
Control vs DSS 3-day	4.1.1.49	Phosphoenolpyruvate carboxykinase (ATP)
Control vs DSS 3-day	4.1.99.1	Tryptophanase
Control vs DSS 3-day	4.1.99.2	Tyrosine phenol-lyase
Control vs DSS 3-day	5.4.99.2	Methylmalonyl-CoA mutase
Control vs DSS 3-day	6.4.1.3	Propionyl-CoA carboxylase
Control vs DSS 3-day	1.1.1.205	IMP dehydrogenase
Control vs DSS 3-day	1.1.1.23	Histidinol dehydrogenase
Control vs DSS 3-day	1.1.1.262	4-hydroxythreonine-4-phosphate dehydrogenase
Control vs DSS 3-day	1.1.1.290	4-phosphoerythronate dehydrogenase
Control vs DSS 3-day	1.1.1.37	Malate dehydrogenase
Control vs DSS 3-day	1.1.1.86	Ketol-acid reductoisomerase (NADP(+))
Control vs DSS 3-day	1.1.1.94	Glycerol-3-phosphate dehydrogenase (NAD(P)(+))
Control vs DSS 3-day	1.1.1.95	Phosphoglycerate dehydrogenase
Control vs DSS 3-day	1.11.1.1	NADH peroxidase
Control vs DSS 3-day	1.11.1.15	Peroxiredoxin
Control vs DSS 3-day	1.15.1.1	Superoxide dismutase
Control vs DSS 3-day	1.17.4.1	Ribonucleoside-diphosphate reductase
Control vs DSS 3-day	1.17.4.2	Ribonucleoside-triphosphate reductase
Control vs DSS 3-day	1.3.1.14	Dihydroorotate dehydrogenase (NAD(+))
Control vs DSS 3-day	1.3.1.9	Enoyl-[acyl-carrier-protein] reductase (NADH)
Control vs DSS 3-day	1.4.1.2	Glutamate dehydrogenase
Control vs DSS 3-day	1.4.3.16	L-aspartate oxidase
Control vs DSS 3-day	1.5.1.5	Methylenetetrahydrofolate dehydrogenase (NADP(+))
Control vs DSS 3-day	1.6.5.11	NADH dehydrogenase (quinone)
Control vs DSS 3-day	1.6.5.3	NADH
Control vs DSS 3-day	1.7.1.13	PreQ(1) synthase
Control vs DSS 3-day	1.8.1.4	Dihydrolipoyl dehydrogenase
Control vs DSS 3-day	1.8.1.9	Thioredoxin-disulfide reductase
Control vs DSS 3-day	2.1.1.163	Demethylmenaquinone methyltransferase

Control vs DSS 3-day	2.1.1.191	23S rRNA (cytosine(1962)-C(5))-methyltransferase
Control vs DSS 3-day	2.1.1.192	23S rRNA (adenine(2503)-C(2))-methyltransferase
Control vs DSS 3-day	2.1.1.63	Methylated-DNA--[protein]-cysteine S-methyltransferase
Control vs DSS 3-day	2.1.1.72	Site-specific DNA-methyltransferase (adenine-specific)
Control vs DSS 3-day	2.1.2.1	Glycine hydroxymethyltransferase
Control vs DSS 3-day	2.1.2.3	Phosphoribosylaminoimidazolecarboxamide formyltransferase
Control vs DSS 3-day	2.1.3.2	Aspartate carbamoyltransferase
Control vs DSS 3-day	2.3.1.129	Acyl-[acyl-carrier-protein]--UDP-N-acetylglucosamine O-acyltransferase
Control vs DSS 3-day	2.3.1.179	Beta-ketoacyl-[acyl-carrier-protein] synthase II
Control vs DSS 3-day	2.3.1.180	Beta-ketoacyl-[acyl-carrier-protein] synthase III
Control vs DSS 3-day	2.3.1.29	Glycine C-acetyltransferase
Control vs DSS 3-day	2.3.1.47	8-amino-7-oxononanoate synthase
Control vs DSS 3-day	2.3.3.13	2-isopropylmalate synthase
Control vs DSS 3-day	2.4.1.21	Starch synthase
Control vs DSS 3-day	2.4.2.1	Purine-nucleoside phosphorylase
Control vs DSS 3-day	2.4.2.10	Orotate phosphoribosyltransferase
Control vs DSS 3-day	2.4.2.14	Amidophosphoribosyltransferase
Control vs DSS 3-day	2.4.2.17	ATP phosphoribosyltransferase
Control vs DSS 3-day	2.4.2.19	Nicotinate-nucleotide diphosphorylase (carboxylating)
Control vs DSS 3-day	2.5.1.78	6,7-dimethyl-8-ribityllumazine synthase
Control vs DSS 3-day	2.6.1.42	Branched-chain-amino-acid transaminase
Control vs DSS 3-day	2.6.1.52	Phosphoserine transaminase
Control vs DSS 3-day	2.6.1.83	LL-diaminopimelate aminotransferase
Control vs DSS 3-day	2.6.1.9	Histidinol-phosphate transaminase
Control vs DSS 3-day	2.6.99.2	Pyridoxine 5'-phosphate synthase
Control vs DSS 3-day	2.7.1.40	Pyruvate kinase
Control vs DSS 3-day	2.7.1.48	Uridine kinase
Control vs DSS 3-day	2.7.1.69	Protein-N(pi)-phosphohistidine--sugar phosphotransferase
Control vs DSS 3-day	2.7.1.90	Diphosphate--fructose-6-phosphate 1-phosphotransferase
Control vs DSS 3-day	2.7.13.3	Histidine kinase
Control vs DSS 3-day	2.7.2.1	Acetate kinase
Control vs DSS 3-day	2.7.2.3	Phosphoglycerate kinase
Control vs DSS 3-day	2.7.2.4	Aspartate kinase
Control vs DSS 3-day	2.7.4.6	Nucleoside-diphosphate kinase
Control vs DSS 3-day	2.7.6.1	Ribose-phosphate diphosphokinase
Control vs DSS 3-day	2.7.7.7	DNA-directed DNA polymerase
Control vs DSS 3-day	2.7.7.72	CCA tRNA nucleotidyltransferase
Control vs DSS 3-day	2.7.7.8	Polyribonucleotide nucleotidyltransferase
Control vs DSS 3-day	2.7.8.13	Phospho-N-acetylmuramoyl-pentapeptide-transferase
Control vs DSS 3-day	2.8.4.3	tRNA-2-methylthio-N(6)-dimethylallyladenosine synthase
Control vs DSS 3-day	3.1.1.29	Aminoacyl-tRNA hydrolase
Control vs DSS 3-day	3.1.1.96	D-aminoacyl-tRNA deacylase
Control vs DSS 3-day	3.1.21.3	Type I site-specific deoxyribonuclease

Control vs DSS 3-day	3.1.26.5	Ribonuclease P
Control vs DSS 3-day	3.1.3.11	Fructose-bisphosphatase
Control vs DSS 3-day	3.1.3.15	Histidinol-phosphatase
Control vs DSS 3-day	3.2.1.135	Neopullulanase
Control vs DSS 3-day	3.2.1.21	Beta-glucosidase
Control vs DSS 3-day	3.2.1.22	Alpha-galactosidase
Control vs DSS 3-day	3.2.1.23	Beta-galactosidase
Control vs DSS 3-day	3.2.1.51	Alpha-L-fucosidase
Control vs DSS 3-day	3.4.11.18	Methionyl aminopeptidase
Control vs DSS 3-day	3.4.11.9	Xaa-Pro aminopeptidase
Control vs DSS 3-day	3.4.13.18	Cytosol nonspecific dipeptidase
Control vs DSS 3-day	3.4.14.12	Xaa-Xaa-Pro tripeptidyl-peptidase
Control vs DSS 3-day	3.4.14.4	Dipeptidyl-peptidase III
Control vs DSS 3-day	3.4.21.53	Endopeptidase La
Control vs DSS 3-day	3.4.21.92	Endopeptidase Clp
Control vs DSS 3-day	3.5.1.108	UDP-3-O-acyl-N-acetylglucosamine deacetylase
Control vs DSS 3-day	3.5.1.28	N-acetylmuramoyl-L-alanine amidase
Control vs DSS 3-day	3.5.1.88	Peptide deformylase
Control vs DSS 3-day	3.5.4.10	IMP cyclohydrolase
Control vs DSS 3-day	3.5.4.16	GTP cyclohydrolase I
Control vs DSS 3-day	3.5.4.9	Methenyltetrahydrofolate cyclohydrolase
Control vs DSS 3-day	3.5.99.6	Glucosamine-6-phosphate deaminase
Control vs DSS 3-day	3.6.3.31	Polyamine-transporting ATPase
Control vs DSS 3-day	3.6.4.12	DNA helicase
Control vs DSS 3-day	4.1.1.19	Arginine decarboxylase
Control vs DSS 3-day	4.1.1.23	Orotidine-5'-phosphate decarboxylase
Control vs DSS 3-day	4.1.2.4	Deoxyribose-phosphate aldolase
Control vs DSS 3-day	4.1.3.36	1,4-dihydroxy-2-naphthoyl-CoA synthase
Control vs DSS 3-day	4.2.1.11	Phosphopyruvate hydratase
Control vs DSS 3-day	4.2.1.17	Enoyl-CoA hydratase
Control vs DSS 3-day	4.2.1.19	Imidazoleglycerol-phosphate dehydratase
Control vs DSS 3-day	4.2.1.2	Fumarate hydratase
Control vs DSS 3-day	4.2.1.59	3-hydroxyacyl-[acyl-carrier-protein] dehydratase
Control vs DSS 3-day	4.2.3.5	Chorismate synthase
Control vs DSS 3-day	4.3.3.7	4-hydroxy-tetrahydrodipicolinate synthase
Control vs DSS 3-day	5.1.1.7	Diaminopimelate epimerase
Control vs DSS 3-day	5.1.3.1	Ribulose-phosphate 3-epimerase
Control vs DSS 3-day	5.2.1.8	Peptidylprolyl isomerase
Control vs DSS 3-day	5.3.1.1	Triose-phosphate isomerase
Control vs DSS 3-day	5.3.1.12	Glucuronate isomerase
Control vs DSS 3-day	5.3.1.17	5-dehydro-4-deoxy-D-glucuronate isomerase
Control vs DSS 3-day	5.3.1.25	L-fucose isomerase
Control vs DSS 3-day	5.4.2.12	Phosphoglycerate mutase (2,3-diphosphoglycerate-independent)

Control vs DSS 3-day	5.4.2.2	Phosphoglucomutase (alpha-D-glucose-1,6-bisphosphate-dependent)
Control vs DSS 3-day	5.99.1.3	DNA topoisomerase (ATP-hydrolyzing)
Control vs DSS 3-day	6.1.1.10	Methionine--tRNA ligase
Control vs DSS 3-day	6.1.1.11	Serine--tRNA ligase
Control vs DSS 3-day	6.1.1.12	Aspartate--tRNA ligase
Control vs DSS 3-day	6.1.1.17	Glutamate--tRNA ligase
Control vs DSS 3-day	6.1.1.2	Tryptophan--tRNA ligase
Control vs DSS 3-day	6.1.1.20	Phenylalanine--tRNA ligase
Control vs DSS 3-day	6.1.1.22	Asparagine--tRNA ligase
Control vs DSS 3-day	6.1.1.5	Isoleucine--tRNA ligase
Control vs DSS 3-day	6.1.1.6	Lysine--tRNA ligase
Control vs DSS 3-day	6.1.1.7	Alanine--tRNA ligase
Control vs DSS 3-day	6.1.1.9	Valine--tRNA ligase
Control vs DSS 3-day	6.3.1.1	Aspartate--ammonia ligase
Control vs DSS 3-day	6.3.2.6	Phosphoribosylaminoimidazolesuccinocarboxamide synthase
Control vs DSS 3-day	6.3.2.9	UDP-N-acetylmuramoyl-L-alanine--D-glutamate ligase
Control vs DSS 3-day	6.3.4.14	Biotin carboxylase
Control vs DSS 3-day	6.3.5.1	NAD(+) synthase (glutamine-hydrolyzing)
Control vs DSS 3-day	6.3.5.3	Phosphoribosylformylglycinamidine synthase
Control vs DSS 3-day	6.3.5.5	Carbamoyl-phosphate synthase (glutamine-hydrolyzing)
Control vs DSS 3-day	6.4.1.2	Acetyl-CoA carboxylase
Control vs DSS 3-day	6.4.1.3	Propionyl-CoA carboxylase
Control vs DSS 3-day	7.1.2.2	NO_NAME
Control vs DSS 3-day	7.2.1.1	NO_NAME
Control vs DSS 3-day	1.1.1.100	3-oxoacyl-[acyl-carrier-protein] reductase
Control vs DSS 3-day	1.1.1.131	Mannuronate reductase
Control vs DSS 3-day	1.1.1.133	dTDP-4-dehydrorhamnose reductase
Control vs DSS 3-day	1.1.1.169	2-dehydropantoate 2-reductase
Control vs DSS 3-day	1.1.1.205	IMP dehydrogenase
Control vs DSS 3-day	1.1.1.22	UDP-glucose 6-dehydrogenase
Control vs DSS 3-day	1.1.1.23	Histidinol dehydrogenase
Control vs DSS 3-day	1.1.1.25	Shikimate dehydrogenase
Control vs DSS 3-day	1.1.1.262	4-hydroxythreonine-4-phosphate dehydrogenase
Control vs DSS 3-day	1.1.1.267	1-deoxy-D-xylulose-5-phosphate reductoisomerase
Control vs DSS 3-day	1.1.1.271	GDP-L-fucose synthase
Control vs DSS 3-day	1.1.1.290	4-phosphoerythronate dehydrogenase
Control vs DSS 3-day	1.1.1.37	Malate dehydrogenase
Control vs DSS 3-day	1.1.1.40	Malate dehydrogenase (oxaloacetate-decarboxylating) (NADP(+))
Control vs DSS 3-day	1.1.1.42	Isocitrate dehydrogenase (NADP(+))
Control vs DSS 3-day	1.1.1.44	Phosphogluconate dehydrogenase (NADP(+)-dependent, decarboxylating)
Control vs DSS 3-day	1.1.1.49	Glucose-6-phosphate dehydrogenase (NADP(+))
Control vs DSS 3-day	1.1.1.58	Tagaturonate reductase
Control vs DSS 3-day	1.1.1.85	3-isopropylmalate dehydrogenase

Control vs DSS 3-day	1.1.1.86	Ketol-acid reductoisomerase (NADP(+))
Control vs DSS 3-day	1.1.1.94	Glycerol-3-phosphate dehydrogenase (NAD(P)(+))
Control vs DSS 3-day	1.1.1.95	Phosphoglycerate dehydrogenase
Control vs DSS 3-day	1.11.1.1	NADH peroxidase
Control vs DSS 3-day	1.11.1.15	Peroxiredoxin
Control vs DSS 3-day	1.15.1.1	Superoxide dismutase
Control vs DSS 3-day	1.16.3.2	Bacterial non-heme ferritin
Control vs DSS 3-day	1.17.4.1	Ribonucleoside-diphosphate reductase
Control vs DSS 3-day	1.17.4.2	Ribonucleoside-triphosphate reductase
Control vs DSS 3-day	1.17.7.3	(E)-4-hydroxy-3-methylbut-2-enyl-diphosphate synthase (flavodoxin)
Control vs DSS 3-day	1.17.7.4	NO_NAME
Control vs DSS 3-day	1.2.1.11	Aspartate-semialdehyde dehydrogenase
Control vs DSS 3-day	1.2.1.38	N-acetyl-gamma-glutamyl-phosphate reductase
Control vs DSS 3-day	1.2.1.41	Glutamate-5-semialdehyde dehydrogenase
Control vs DSS 3-day	1.2.7.8	Indolepyruvate ferredoxin oxidoreductase
Control vs DSS 3-day	1.3.1.1	Dihydrouracil dehydrogenase (NAD(+))
Control vs DSS 3-day	1.3.1.14	Dihydroorotate dehydrogenase (NAD(+))
Control vs DSS 3-day	1.3.1.26	Transferred entry
Control vs DSS 3-day	1.3.1.9	Enoyl-[acyl-carrier-protein] reductase (NADH)
Control vs DSS 3-day	1.3.98.1	Dihydroorotate oxidase (fumarate)
Control vs DSS 3-day	1.3.99.5	3-oxo-5-alpha-steroid 4-dehydrogenase (acceptor)
Control vs DSS 3-day	1.4.1.13	Glutamate synthase (NADPH)
Control vs DSS 3-day	1.4.1.16	Diaminopimelate dehydrogenase
Control vs DSS 3-day	1.4.1.2	Glutamate dehydrogenase
Control vs DSS 3-day	1.4.1.3	Glutamate dehydrogenase (NAD(P)(+))
Control vs DSS 3-day	1.4.3.16	L-aspartate oxidase
Control vs DSS 3-day	1.4.4.2	Glycine dehydrogenase (aminomethyl-transferring)
Control vs DSS 3-day	1.5.1.2	Pyrroline-5-carboxylate reductase
Control vs DSS 3-day	1.5.1.20	Methylenetetrahydrofolate reductase (NAD(P)H)
Control vs DSS 3-day	1.5.1.3	Dihydrofolate reductase
Control vs DSS 3-day	1.5.1.39	FMN reductase (NAD(P)H)
Control vs DSS 3-day	1.5.1.5	Methylenetetrahydrofolate dehydrogenase (NADP(+))
Control vs DSS 3-day	1.6.5.11	NADH dehydrogenase (quinone)
Control vs DSS 3-day	1.6.5.3	NADH
Control vs DSS 3-day	1.6.99.1	NADPH dehydrogenase
Control vs DSS 3-day	1.6.99.5	Transferred entry
Control vs DSS 3-day	1.7.1.13	PreQ(1) synthase
Control vs DSS 3-day	1.8.1.9	Thioredoxin-disulfide reductase
Control vs DSS 3-day	1.97.1.4	[Formate-C-acetyltransferase]-activating enzyme
Control vs DSS 3-day	2.1.1.13	Methionine synthase
Control vs DSS 3-day	2.1.1.163	Demethylmenaquinone methyltransferase
Control vs DSS 3-day	2.1.1.182	16S rRNA (adenine(1518)-N(6)/adenine(1519)-N(6))-dimethyltransferase
Control vs DSS 3-day	2.1.1.185	23S rRNA (guanosine(2251)-2'-O)-methyltransferase

Control vs DSS 3-day	2.1.1.191	23S rRNA (cytosine(1962)-C(5))-methyltransferase
Control vs DSS 3-day	2.1.1.192	23S rRNA (adenine(2503)-C(2))-methyltransferase
Control vs DSS 3-day	2.1.1.193	16S rRNA (uracil(1498)-N(3))-methyltransferase
Control vs DSS 3-day	2.1.1.198	16S rRNA (cytidine(1402)-2'-O)-methyltransferase
Control vs DSS 3-day	2.1.1.199	16S rRNA (cytosine(1402)-N(4))-methyltransferase
Control vs DSS 3-day	2.1.1.228	tRNA (guanine(37)-N(1))-methyltransferase
Control vs DSS 3-day	2.1.1.33	tRNA (guanine(46)-N(7))-methyltransferase
Control vs DSS 3-day	2.1.1.45	Thymidylate synthase
Control vs DSS 3-day	2.1.1.63	Methylated-DNA--[protein]-cysteine S-methyltransferase
Control vs DSS 3-day	2.1.2.1	Glycine hydroxymethyltransferase
Control vs DSS 3-day	2.1.2.10	Aminomethyltransferase
Control vs DSS 3-day	2.1.2.11	3-methyl-2-oxobutanoate hydroxymethyltransferase
Control vs DSS 3-day	2.1.2.2	Phosphoribosylglycinamide formyltransferase
Control vs DSS 3-day	2.1.2.3	Phosphoribosylaminoimidazolecarboxamide formyltransferase
Control vs DSS 3-day	2.1.2.9	Methionyl-tRNA formyltransferase
Control vs DSS 3-day	2.1.3.2	Aspartate carbamoyltransferase
Control vs DSS 3-day	2.1.3.9	N-acetylornithine carbamoyltransferase
Control vs DSS 3-day	2.2.1.6	Acetolactate synthase
Control vs DSS 3-day	2.2.1.7	1-deoxy-D-xylulose-5-phosphate synthase
Control vs DSS 3-day	2.2.1.9	synthase
Control vs DSS 3-day	2.3.1.129	Acyl-[acyl-carrier-protein]--UDP-N-acetylglucosamine O-acyltransferase
Control vs DSS 3-day	2.3.1.179	Beta-ketoacyl-[acyl-carrier-protein] synthase II
Control vs DSS 3-day	2.3.1.180	Beta-ketoacyl-[acyl-carrier-protein] synthase III
Control vs DSS 3-day	2.3.1.181	Lipoyl(octanoyl) transferase
Control vs DSS 3-day	2.3.1.234	N(6)-L-threonylcarbamoyladenine synthase
Control vs DSS 3-day	2.3.1.29	Glycine C-acetyltransferase
Control vs DSS 3-day	2.3.1.31	Homoserine O-acetyltransferase
Control vs DSS 3-day	2.3.1.39	[Acyl-carrier-protein] S-malonyltransferase
Control vs DSS 3-day	2.3.1.47	8-amino-7-oxononanoate synthase
Control vs DSS 3-day	2.3.1.51	1-acylglycerol-3-phosphate O-acyltransferase
Control vs DSS 3-day	2.3.3.13	2-isopropylmalate synthase
Control vs DSS 3-day	2.4.1.18	1,4-alpha-glucan branching enzyme
Control vs DSS 3-day	2.4.1.182	Lipid-A-disaccharide synthase
Control vs DSS 3-day	2.4.1.21	Starch synthase
Control vs DSS 3-day	2.4.1.212	Hyaluronan synthase
Control vs DSS 3-day	2.4.1.320	1,4-beta-mannosyl-N-acetylglucosamine phosphorylase
Control vs DSS 3-day	2.4.1.54	Undecaprenyl-phosphate mannosyltransferase
Control vs DSS 3-day	2.4.2.1	Purine-nucleoside phosphorylase
Control vs DSS 3-day	2.4.2.10	Orotate phosphoribosyltransferase
Control vs DSS 3-day	2.4.2.14	Amidophosphoribosyltransferase
Control vs DSS 3-day	2.4.2.17	ATP phosphoribosyltransferase
Control vs DSS 3-day	2.4.2.18	Anthranilate phosphoribosyltransferase
Control vs DSS 3-day	2.4.2.19	Nicotinate-nucleotide diphosphorylase (carboxylating)

Control vs DSS 3-day	2.4.2.22	Xanthine phosphoribosyltransferase
Control vs DSS 3-day	2.4.2.29	tRNA-guanine(34) transglycosylase
Control vs DSS 3-day	2.4.2.53	Undecaprenyl-phosphate 4-deoxy-4-formamido-L-arabinose transferase
Control vs DSS 3-day	2.4.2.7	Adenine phosphoribosyltransferase
Control vs DSS 3-day	2.4.2.8	Hypoxanthine phosphoribosyltransferase
Control vs DSS 3-day	2.4.99.17	S-adenosylmethionine
Control vs DSS 3-day	2.5.1.15	Dihydropteroate synthase
Control vs DSS 3-day	2.5.1.17	Cob(I)yrinic acid a,c-diamide adenosyltransferase
Control vs DSS 3-day	2.5.1.19	3-phosphoshikimate 1-carboxyvinyltransferase
Control vs DSS 3-day	2.5.1.47	Cysteine synthase
Control vs DSS 3-day	2.5.1.55	3-deoxy-8-phosphooctulonate synthase
Control vs DSS 3-day	2.5.1.6	Methionine adenosyltransferase
Control vs DSS 3-day	2.5.1.7	UDP-N-acetylglucosamine 1-carboxyvinyltransferase
Control vs DSS 3-day	2.5.1.72	Quinolinate synthase
Control vs DSS 3-day	2.5.1.75	tRNA dimethylallyltransferase
Control vs DSS 3-day	2.5.1.78	6,7-dimethyl-8-ribityllumazine synthase
Control vs DSS 3-day	2.5.1.9	Riboflavin synthase
Control vs DSS 3-day	2.6.1.1	Aspartate transaminase
Control vs DSS 3-day	2.6.1.16	Glutamine--fructose-6-phosphate transaminase (isomerizing)
Control vs DSS 3-day	2.6.1.42	Branched-chain-amino-acid transaminase
Control vs DSS 3-day	2.6.1.52	Phosphoserine transaminase
Control vs DSS 3-day	2.6.1.83	LL-diaminopimelate aminotransferase
Control vs DSS 3-day	2.6.1.9	Histidinol-phosphate transaminase
Control vs DSS 3-day	2.6.99.2	Pyridoxine 5'-phosphate synthase
Control vs DSS 3-day	2.7.1.107	Diacylglycerol kinase (ATP)
Control vs DSS 3-day	2.7.1.11	6-phosphofructokinase
Control vs DSS 3-day	2.7.1.130	Tetraacyldisaccharide 4'-kinase
Control vs DSS 3-day	2.7.1.180	FAD
Control vs DSS 3-day	2.7.1.21	Thymidine kinase
Control vs DSS 3-day	2.7.1.25	Adenylyl-sulfate kinase
Control vs DSS 3-day	2.7.1.26	Riboflavin kinase
Control vs DSS 3-day	2.7.1.33	Pantothenate kinase
Control vs DSS 3-day	2.7.1.35	Pyridoxal kinase
Control vs DSS 3-day	2.7.1.40	Pyruvate kinase
Control vs DSS 3-day	2.7.1.48	Uridine kinase
Control vs DSS 3-day	2.7.1.69	Protein-N(pi)-phosphohistidine--sugar phosphotransferase
Control vs DSS 3-day	2.7.1.71	Shikimate kinase
Control vs DSS 3-day	2.7.1.90	Diphosphate--fructose-6-phosphate 1-phosphotransferase
Control vs DSS 3-day	2.7.13.3	Histidine kinase
Control vs DSS 3-day	2.7.2.1	Acetate kinase
Control vs DSS 3-day	2.7.2.11	Glutamate 5-kinase
Control vs DSS 3-day	2.7.2.3	Phosphoglycerate kinase
Control vs DSS 3-day	2.7.2.4	Aspartate kinase

Control vs DSS 3-day	2.7.2.8	Acetylglutamate kinase
Control vs DSS 3-day	2.7.4.16	Thiamine-phosphate kinase
Control vs DSS 3-day	2.7.4.22	UMP kinase
Control vs DSS 3-day	2.7.4.25	(d)CMP kinase
Control vs DSS 3-day	2.7.4.3	Adenylate kinase
Control vs DSS 3-day	2.7.4.8	Guanylate kinase
Control vs DSS 3-day	2.7.6.1	Ribose-phosphate diphosphokinase
Control vs DSS 3-day	2.7.6.3	2-amino-4-hydroxy-6-hydroxymethyldihydropteridine diphosphokinase
Control vs DSS 3-day	2.7.7.13	Mannose-1-phosphate guanylyltransferase
Control vs DSS 3-day	2.7.7.18	Nicotinate-nucleotide adenylyltransferase
Control vs DSS 3-day	2.7.7.2	FAD synthetase
Control vs DSS 3-day	2.7.7.24	Glucose-1-phosphate thymidylyltransferase
Control vs DSS 3-day	2.7.7.3	Pantetheine-phosphate adenylyltransferase
Control vs DSS 3-day	2.7.7.38	3-deoxy-manno-octulosonate cytidylyltransferase
Control vs DSS 3-day	2.7.7.4	Sulfate adenylyltransferase
Control vs DSS 3-day	2.7.7.41	Phosphatidate cytidylyltransferase
Control vs DSS 3-day	2.7.7.60	2-C-methyl-D-erythritol 4-phosphate cytidylyltransferase
Control vs DSS 3-day	2.7.7.7	DNA-directed DNA polymerase
Control vs DSS 3-day	2.7.7.72	CCA tRNA nucleotidyltransferase
Control vs DSS 3-day	2.7.7.8	Polyribonucleotide nucleotidyltransferase
Control vs DSS 3-day	2.7.7.85	Diadenylate cyclase
Control vs DSS 3-day	2.7.8.13	Phospho-N-acetylmuramoyl-pentapeptide-transferase
Control vs DSS 3-day	2.7.8.8	CDP-diacylglycerol--serine O-phosphatidyltransferase
Control vs DSS 3-day	2.7.9.1	Pyruvate, phosphate dikinase
Control vs DSS 3-day	2.8.1.7	Cysteine desulfurase
Control vs DSS 3-day	2.8.4.3	tRNA-2-methylthio-N(6)-dimethylallyladenosine synthase
Control vs DSS 3-day	2.8.4.4	[Ribosomal protein S12] (aspartate(89)-C(3))-methylthiotransferase
Control vs DSS 3-day	3.1.1.11	Pectinesterase
Control vs DSS 3-day	3.1.1.29	Aminoacyl-tRNA hydrolase
Control vs DSS 3-day	3.1.1.96	D-aminoacyl-tRNA deacylase
Control vs DSS 3-day	3.1.11.5	Exodeoxyribonuclease V
Control vs DSS 3-day	3.1.11.6	Exodeoxyribonuclease VII
Control vs DSS 3-day	3.1.13.1	Exoribonuclease II
Control vs DSS 3-day	3.1.21.2	Deoxyribonuclease IV
Control vs DSS 3-day	3.1.22.4	Crossover junction endodeoxyribonuclease
Control vs DSS 3-day	3.1.26.11	Ribonuclease Z
Control vs DSS 3-day	3.1.26.3	Ribonuclease III
Control vs DSS 3-day	3.1.26.4	Ribonuclease H
Control vs DSS 3-day	3.1.26.5	Ribonuclease P
Control vs DSS 3-day	3.1.3.11	Fructose-bisphosphatase
Control vs DSS 3-day	3.1.3.15	Histidinol-phosphatase
Control vs DSS 3-day	3.1.3.25	Inositol-phosphate phosphatase
Control vs DSS 3-day	3.1.3.45	3-deoxy-manno-octulosonate-8-phosphatase

Control vs DSS 3-day	3.1.3.5	5'-nucleotidase
Control vs DSS 3-day	3.2.1.131	Xylan alpha-1,2-glucuronosidase
Control vs DSS 3-day	3.2.1.135	Neopullulanase
Control vs DSS 3-day	3.2.1.165	Exo-1,4-beta-D-glucosaminidase
Control vs DSS 3-day	3.2.1.172	Unsaturated rhamnogalacturonyl hydrolase
Control vs DSS 3-day	3.2.1.21	Beta-glucosidase
Control vs DSS 3-day	3.2.1.22	Alpha-galactosidase
Control vs DSS 3-day	3.2.1.23	Beta-galactosidase
Control vs DSS 3-day	3.2.1.25	Beta-mannosidase
Control vs DSS 3-day	3.2.1.31	Beta-glucuronidase
Control vs DSS 3-day	3.2.1.37	Xylan 1,4-beta-xylosidase
Control vs DSS 3-day	3.2.1.55	Non-reducing end alpha-L-arabinofuranosidase
Control vs DSS 3-day	3.2.2.27	Uracil-DNA glycosylase
Control vs DSS 3-day	3.2.2.n1	Cytokinin riboside 5'-monophosphate phosphoribohydrolase
Control vs DSS 3-day	3.3.1.1	Adenosylhomocysteinase
Control vs DSS 3-day	3.4.11.18	Methionyl aminopeptidase
Control vs DSS 3-day	3.4.11.4	Tripeptide aminopeptidase
Control vs DSS 3-day	3.4.11.9	Xaa-Pro aminopeptidase
Control vs DSS 3-day	3.4.13.18	Cytosol nonspecific dipeptidase
Control vs DSS 3-day	3.4.13.20	Beta-Ala-His dipeptidase
Control vs DSS 3-day	3.4.14.12	Xaa-Xaa-Pro tripeptidyl-peptidase
Control vs DSS 3-day	3.4.14.4	Dipeptidyl-peptidase III
Control vs DSS 3-day	3.4.15.5	Peptidyl-dipeptidase Dcp
Control vs DSS 3-day	3.4.16.4	Serine-type D-Ala-D-Ala carboxypeptidase
Control vs DSS 3-day	3.4.21.102	C-terminal processing peptidase
Control vs DSS 3-day	3.4.21.105	Rhomboid protease
Control vs DSS 3-day	3.4.21.107	Peptidase Do
Control vs DSS 3-day	3.4.21.53	Endopeptidase La
Control vs DSS 3-day	3.4.21.89	Signal peptidase I
Control vs DSS 3-day	3.4.21.92	Endopeptidase Clp
Control vs DSS 3-day	3.4.23.36	Signal peptidase II
Control vs DSS 3-day	3.4.24.55	Pitrilysin
Control vs DSS 3-day	3.5.1.10	Formyltetrahydrofolate deformylase
Control vs DSS 3-day	3.5.1.100	(R)-amidase
Control vs DSS 3-day	3.5.1.104	Peptidoglycan-N-acetylglucosamine deacetylase
Control vs DSS 3-day	3.5.1.108	UDP-3-O-acyl-N-acetylglucosamine deacetylase
Control vs DSS 3-day	3.5.1.2	Glutaminase
Control vs DSS 3-day	3.5.1.25	N-acetylglucosamine-6-phosphate deacetylase
Control vs DSS 3-day	3.5.1.28	N-acetylmuramoyl-L-alanine amidase
Control vs DSS 3-day	3.5.1.88	Peptide deformylase
Control vs DSS 3-day	3.5.2.3	Dihydroorotase
Control vs DSS 3-day	3.5.2.6	Beta-lactamase
Control vs DSS 3-day	3.5.4.10	IMP cyclohydrolase

Control vs DSS 3-day	3.5.4.16	GTP cyclohydrolase I
Control vs DSS 3-day	3.5.4.19	Phosphoribosyl-AMP cyclohydrolase
Control vs DSS 3-day	3.5.4.25	GTP cyclohydrolase II
Control vs DSS 3-day	3.5.4.3	Guanine deaminase
Control vs DSS 3-day	3.5.4.5	Cytidine deaminase
Control vs DSS 3-day	3.5.4.9	Methenyltetrahydrofolate cyclohydrolase
Control vs DSS 3-day	3.5.99.6	Glucosamine-6-phosphate deaminase
Control vs DSS 3-day	3.6.1.23	dUTP diphosphatase
Control vs DSS 3-day	3.6.1.27	Undecaprenyl-diphosphate phosphatase
Control vs DSS 3-day	3.6.1.31	Phosphoribosyl-ATP diphosphatase
Control vs DSS 3-day	3.6.1.66	XTP/dITP diphosphatase
Control vs DSS 3-day	3.6.3.31	Polyamine-transporting ATPase
Control vs DSS 3-day	3.6.3.41	Heme-transporting ATPase
Control vs DSS 3-day	3.6.4.12	DNA helicase
Control vs DSS 3-day	3.6.4.13	RNA helicase
Control vs DSS 3-day	3.6.5.n1	Elongation factor 4
Control vs DSS 3-day	4.1.1.19	Arginine decarboxylase
Control vs DSS 3-day	4.1.1.20	Diaminopimelate decarboxylase
Control vs DSS 3-day	4.1.1.23	Orotidine-5'-phosphate decarboxylase
Control vs DSS 3-day	4.1.1.36	Phosphopantothenoylcysteine decarboxylase
Control vs DSS 3-day	4.1.1.65	Phosphatidylserine decarboxylase
Control vs DSS 3-day	4.1.2.19	Rhamnulose-1-phosphate aldolase
Control vs DSS 3-day	4.1.2.4	Deoxyribose-phosphate aldolase
Control vs DSS 3-day	4.1.2.50	6-carboxytetrahydropterin synthase
Control vs DSS 3-day	4.1.3.3	N-acetylneuraminate lyase
Control vs DSS 3-day	4.1.3.36	1,4-dihydroxy-2-naphthoyl-CoA synthase
Control vs DSS 3-day	4.1.99.12	3,4-dihydroxy-2-butanone-4-phosphate synthase
Control vs DSS 3-day	4.2.1.10	3-dehydroquinate dehydratase
Control vs DSS 3-day	4.2.1.11	Phosphopyruvate hydratase
Control vs DSS 3-day	4.2.1.136	ADP-dependent NAD(P)H-hydrate dehydratase
Control vs DSS 3-day	4.2.1.17	Enoyl-CoA hydratase
Control vs DSS 3-day	4.2.1.19	Imidazoleglycerol-phosphate dehydratase
Control vs DSS 3-day	4.2.1.2	Fumarate hydratase
Control vs DSS 3-day	4.2.1.20	Tryptophan synthase
Control vs DSS 3-day	4.2.1.3	Aconitate hydratase
Control vs DSS 3-day	4.2.1.33	3-isopropylmalate dehydratase
Control vs DSS 3-day	4.2.1.46	dTDP-glucose 4,6-dehydratase
Control vs DSS 3-day	4.2.1.59	3-hydroxyacyl-[acyl-carrier-protein] dehydratase
Control vs DSS 3-day	4.2.1.8	Mannonate dehydratase
Control vs DSS 3-day	4.2.1.9	Dihydroxy-acid dehydratase
Control vs DSS 3-day	4.2.2.2	Pectate lyase
Control vs DSS 3-day	4.2.3.1	Threonine synthase
Control vs DSS 3-day	4.2.3.3	Methylglyoxal synthase

Control vs DSS 3-day	4.2.3.4	3-dehydroquinate synthase
Control vs DSS 3-day	4.2.3.5	Chorismate synthase
Control vs DSS 3-day	4.2.99.18	DNA-(apurinic or apyrimidinic site) lyase
Control vs DSS 3-day	4.3.1.17	L-serine ammonia-lyase
Control vs DSS 3-day	4.3.2.1	Argininosuccinate lyase
Control vs DSS 3-day	4.3.2.10	NO_NAME
Control vs DSS 3-day	4.3.2.2	Adenylosuccinate lyase
Control vs DSS 3-day	4.3.3.7	4-hydroxy-tetrahydrodipicolinate synthase
Control vs DSS 3-day	4.3.99.3	7-carboxy-7-deazaguanine synthase
Control vs DSS 3-day	5.1.1.1	Alanine racemase
Control vs DSS 3-day	5.1.1.3	Glutamate racemase
Control vs DSS 3-day	5.1.3.13	dTDP-4-dehydrorhamnose 3,5-epimerase
Control vs DSS 3-day	5.1.3.3	Aldose 1-epimerase
Control vs DSS 3-day	5.1.3.32	L-rhamnose mutarotase
Control vs DSS 3-day	5.1.3.4	L-ribulose-5-phosphate 4-epimerase
Control vs DSS 3-day	5.1.99.6	NAD(P)H-hydrate epimerase
Control vs DSS 3-day	5.2.1.8	Peptidylprolyl isomerase
Control vs DSS 3-day	5.3.1.1	Triose-phosphate isomerase
Control vs DSS 3-day	5.3.1.12	Glucuronate isomerase
Control vs DSS 3-day	5.3.1.16	isomerase
Control vs DSS 3-day	5.3.1.17	5-dehydro-4-deoxy-D-glucuronate isomerase
Control vs DSS 3-day	5.3.1.4	L-arabinose isomerase
Control vs DSS 3-day	5.3.1.5	Xylose isomerase
Control vs DSS 3-day	5.3.1.9	Glucose-6-phosphate isomerase
Control vs DSS 3-day	5.3.3.2	Isopentenyl-diphosphate Delta-isomerase
Control vs DSS 3-day	5.4.2.12	Phosphoglycerate mutase (2,3-diphosphoglycerate-independent)
Control vs DSS 3-day	5.4.2.2	Phosphoglucomutase (alpha-D-glucose-1,6-bisphosphate-dependent)
Control vs DSS 3-day	5.4.2.6	Beta-phosphoglucomutase
Control vs DSS 3-day	5.4.4.2	Isochorismate synthase
Control vs DSS 3-day	5.4.99.12	tRNA pseudouridine(38-40) synthase
Control vs DSS 3-day	5.4.99.18	5-(carboxyamino)imidazole ribonucleotide mutase
Control vs DSS 3-day	5.4.99.25	tRNA pseudouridine(55) synthase
Control vs DSS 3-day	5.99.1.2	DNA topoisomerase
Control vs DSS 3-day	5.99.1.3	DNA topoisomerase (ATP-hydrolyzing)
Control vs DSS 3-day	6.1.1.1	Tyrosine--tRNA ligase
Control vs DSS 3-day	6.1.1.10	Methionine--tRNA ligase
Control vs DSS 3-day	6.1.1.11	Serine--tRNA ligase
Control vs DSS 3-day	6.1.1.12	Aspartate--tRNA ligase
Control vs DSS 3-day	6.1.1.14	Glycine--tRNA ligase
Control vs DSS 3-day	6.1.1.15	Proline--tRNA ligase
Control vs DSS 3-day	6.1.1.16	Cysteine--tRNA ligase
Control vs DSS 3-day	6.1.1.17	Glutamate--tRNA ligase
Control vs DSS 3-day	6.1.1.18	Glutamine--tRNA ligase

Control vs DSS 3-day	6.1.1.19	Arginine--tRNA ligase
Control vs DSS 3-day	6.1.1.2	Tryptophan--tRNA ligase
Control vs DSS 3-day	6.1.1.20	Phenylalanine--tRNA ligase
Control vs DSS 3-day	6.1.1.21	Histidine--tRNA ligase
Control vs DSS 3-day	6.1.1.22	Asparagine--tRNA ligase
Control vs DSS 3-day	6.1.1.3	Threonine--tRNA ligase
Control vs DSS 3-day	6.1.1.4	Leucine--tRNA ligase
Control vs DSS 3-day	6.1.1.5	Isoleucine--tRNA ligase
Control vs DSS 3-day	6.1.1.6	Lysine--tRNA ligase
Control vs DSS 3-day	6.1.1.7	Alanine--tRNA ligase
Control vs DSS 3-day	6.1.1.9	Valine--tRNA ligase
Control vs DSS 3-day	6.2.1.3	Long-chain-fatty-acid--CoA ligase
Control vs DSS 3-day	6.2.1.30	Phenylacetate--CoA ligase
Control vs DSS 3-day	6.3.1.1	Aspartate--ammonia ligase
Control vs DSS 3-day	6.3.1.2	Glutamate--ammonia ligase
Control vs DSS 3-day	6.3.2.1	Pantoate--beta-alanine ligase (AMP-forming)
Control vs DSS 3-day	6.3.2.10	UDP-N-acetylmuramoyl-tripeptide--D-alanyl-D-alanine ligase
Control vs DSS 3-day	6.3.2.13	UDP-N-acetylmuramoyl-L-alanyl-D-glutamate--2,6-diaminopimelate ligase
Control vs DSS 3-day	6.3.2.4	D-alanine--D-alanine ligase
Control vs DSS 3-day	6.3.2.5	Phosphopantothenate--cysteine ligase
Control vs DSS 3-day	6.3.2.6	Phosphoribosylaminoimidazolesuccinocarboxamide synthase
Control vs DSS 3-day	6.3.2.8	UDP-N-acetylmuramate--L-alanine ligase
Control vs DSS 3-day	6.3.2.9	UDP-N-acetylmuramoyl-L-alanine--D-glutamate ligase
Control vs DSS 3-day	6.3.3.2	5-formyltetrahydrofolate cyclo-ligase
Control vs DSS 3-day	6.3.3.3	Dethiobiotin synthase
Control vs DSS 3-day	6.3.4.13	Phosphoribosylamine--glycine ligase
Control vs DSS 3-day	6.3.4.14	Biotin carboxylase
Control vs DSS 3-day	6.3.4.19	tRNA(Ile)-lysidine synthetase
Control vs DSS 3-day	6.3.4.2	CTP synthase (glutamine hydrolyzing)
Control vs DSS 3-day	6.3.4.3	Formate--tetrahydrofolate ligase
Control vs DSS 3-day	6.3.4.4	Adenylosuccinate synthase
Control vs DSS 3-day	6.3.5.1	NAD(+) synthase (glutamine-hydrolyzing)
Control vs DSS 3-day	6.3.5.2	GMP synthase (glutamine-hydrolyzing)
Control vs DSS 3-day	6.3.5.3	Phosphoribosylformylglycinamidine synthase
Control vs DSS 3-day	6.3.5.5	Carbamoyl-phosphate synthase (glutamine-hydrolyzing)
Control vs DSS 3-day	6.4.1.2	Acetyl-CoA carboxylase
Control vs DSS 3-day	6.4.1.3	Propionyl-CoA carboxylase
Control vs DSS 3-day	6.5.1.2	DNA ligase (NAD(+))
Control vs DSS 3-day	7.1.2.2	NO_NAME
Control vs DSS 3-day	7.2.1.1	NO_NAME
Control vs DSS 3-day	1.1.1.37	Malate dehydrogenase
Control vs DSS 3-day	1.1.1.86	Ketol-acid reductoisomerase (NADP(+))
Control vs DSS 3-day	1.11.1.1	NADH peroxidase

Control vs DSS 3-day	2.1.2.3	Phosphoribosylaminoimidazolecarboxamide formyltransferase
Control vs DSS 3-day	2.3.1.39	[Acyl-carrier-protein] S-malonyltransferase
Control vs DSS 3-day	2.6.1.52	Phosphoserine transaminase
Control vs DSS 3-day	2.7.1.11	6-phosphofructokinase
Control vs DSS 3-day	2.7.13.3	Histidine kinase
Control vs DSS 3-day	2.7.2.3	Phosphoglycerate kinase
Control vs DSS 3-day	2.7.7.6	DNA-directed RNA polymerase
Control vs DSS 3-day	3.1.21.3	Type I site-specific deoxyribonuclease
Control vs DSS 3-day	3.4.11.18	Methionyl aminopeptidase
Control vs DSS 3-day	3.5.4.10	IMP cyclohydrolase
Control vs DSS 3-day	3.5.99.6	Glucosamine-6-phosphate deaminase
Control vs DSS 3-day	3.6.4.12	DNA helicase
Control vs DSS 3-day	4.1.1.49	Phosphoenolpyruvate carboxykinase (ATP)
Control vs DSS 3-day	5.3.1.1	Triose-phosphate isomerase
Control vs DSS 3-day	5.3.1.12	Glucuronate isomerase
Control vs DSS 3-day	5.4.2.12	Phosphoglycerate mutase (2,3-diphosphoglycerate-independent)
Control vs DSS 3-day	5.99.1.3	DNA topoisomerase (ATP-hydrolyzing)
Control vs DSS 3-day	6.1.1.14	Glycine--tRNA ligase
Control vs DSS 3-day	7.2.1.1	NO_NAME
Control vs DSS 3-day	1.1.1.205	IMP dehydrogenase
Control vs DSS 3-day	1.1.1.37	Malate dehydrogenase
Control vs DSS 3-day	1.11.1.1	NADH peroxidase
Control vs DSS 3-day	2.6.1.52	Phosphoserine transaminase
Control vs DSS 3-day	2.7.1.90	Diphosphate--fructose-6-phosphate 1-phosphotransferase
Control vs DSS 3-day	2.7.13.3	Histidine kinase
Control vs DSS 3-day	2.7.7.6	DNA-directed RNA polymerase
Control vs DSS 3-day	4.1.1.49	Phosphoenolpyruvate carboxykinase (ATP)
Control vs DSS 3-day	4.1.3.36	1,4-dihydroxy-2-naphthoyl-CoA synthase
Control vs DSS 3-day	4.2.1.2	Fumarate hydratase
Control vs DSS 3-day	5.3.1.1	Triose-phosphate isomerase
Control vs DSS 3-day	6.1.1.11	Serine--tRNA ligase
Control vs DSS 3-day	6.1.1.14	Glycine--tRNA ligase
Control vs DSS 3-day	7.2.1.1	NO_NAME
Control vs DSS 3-day	1.1.1.37	Malate dehydrogenase
Control vs DSS 3-day	1.1.1.58	Tagaturonate reductase
Control vs DSS 3-day	1.1.1.85	3-isopropylmalate dehydrogenase
Control vs DSS 3-day	1.1.1.86	Ketol-acid reductoisomerase (NADP(+))
Control vs DSS 3-day	1.1.1.95	Phosphoglycerate dehydrogenase
Control vs DSS 3-day	1.11.1.1	NADH peroxidase
Control vs DSS 3-day	1.15.1.1	Superoxide dismutase
Control vs DSS 3-day	1.2.1.11	Aspartate-semialdehyde dehydrogenase
Control vs DSS 3-day	1.3.1.14	Dihydroorotate dehydrogenase (NAD(+))
Control vs DSS 3-day	1.4.1.16	Diaminopimelate dehydrogenase

Control vs DSS 3-day	1.4.3.16	L-aspartate oxidase
Control vs DSS 3-day	1.6.5.11	NADH dehydrogenase (quinone)
Control vs DSS 3-day	1.6.5.3	NADH
Control vs DSS 3-day	1.6.99.5	Transferred entry
Control vs DSS 3-day	2.1.1.182	16S rRNA (adenine(1518)-N(6)/adenine(1519)-N(6))-dimethyltransferase
Control vs DSS 3-day	2.1.1.191	23S rRNA (cytosine(1962)-C(5))-methyltransferase
Control vs DSS 3-day	2.1.1.228	tRNA (guanine(37)-N(1))-methyltransferase
Control vs DSS 3-day	2.1.2.1	Glycine hydroxymethyltransferase
Control vs DSS 3-day	2.1.2.11	3-methyl-2-oxobutanoate hydroxymethyltransferase
Control vs DSS 3-day	2.1.3.2	Aspartate carbamoyltransferase
Control vs DSS 3-day	2.3.1.179	Beta-ketoacyl-[acyl-carrier-protein] synthase II
Control vs DSS 3-day	2.3.1.180	Beta-ketoacyl-[acyl-carrier-protein] synthase III
Control vs DSS 3-day	2.3.1.234	N(6)-L-threonylcarbamoyladenine synthase
Control vs DSS 3-day	2.3.1.47	8-amino-7-oxononanoate synthase
Control vs DSS 3-day	2.4.1.21	Starch synthase
Control vs DSS 3-day	2.4.1.320	1,4-beta-mannosyl-N-acetylglucosamine phosphorylase
Control vs DSS 3-day	2.4.2.1	Purine-nucleoside phosphorylase
Control vs DSS 3-day	2.4.2.10	Orotate phosphoribosyltransferase
Control vs DSS 3-day	2.4.2.29	tRNA-guanine(34) transglycosylase
Control vs DSS 3-day	2.4.99.17	S-adenosylmethionine
Control vs DSS 3-day	2.6.1.16	Glutamine--fructose-6-phosphate transaminase (isomerizing)
Control vs DSS 3-day	2.6.1.52	Phosphoserine transaminase
Control vs DSS 3-day	2.7.1.40	Pyruvate kinase
Control vs DSS 3-day	2.7.13.3	Histidine kinase
Control vs DSS 3-day	2.7.2.3	Phosphoglycerate kinase
Control vs DSS 3-day	2.7.6.1	Ribose-phosphate diphosphokinase
Control vs DSS 3-day	2.7.7.24	Glucose-1-phosphate thymidylyltransferase
Control vs DSS 3-day	2.7.7.7	DNA-directed DNA polymerase
Control vs DSS 3-day	2.7.7.8	Polyribonucleotide nucleotidyltransferase
Control vs DSS 3-day	2.7.8.13	Phospho-N-acetylmuramoyl-pentapeptide-transferase
Control vs DSS 3-day	3.1.3.11	Fructose-bisphosphatase
Control vs DSS 3-day	3.4.11.18	Methionyl aminopeptidase
Control vs DSS 3-day	3.4.11.9	Xaa-Pro aminopeptidase
Control vs DSS 3-day	3.4.21.92	Endopeptidase Clp
Control vs DSS 3-day	3.5.99.6	Glucosamine-6-phosphate deaminase
Control vs DSS 3-day	3.6.5.n1	Elongation factor 4
Control vs DSS 3-day	4.1.1.11	Aspartate 1-decarboxylase
Control vs DSS 3-day	4.1.1.19	Arginine decarboxylase
Control vs DSS 3-day	4.1.1.23	Orotidine-5'-phosphate decarboxylase
Control vs DSS 3-day	4.1.2.4	Deoxyribose-phosphate aldolase
Control vs DSS 3-day	4.2.1.33	3-isopropylmalate dehydratase
Control vs DSS 3-day	4.3.2.1	Argininosuccinate lyase
Control vs DSS 3-day	5.2.1.8	Peptidylprolyl isomerase

Control vs DSS 3-day	5.3.1.17	5-dehydro-4-deoxy-D-glucuronate isomerase
Control vs DSS 3-day	5.3.1.4	L-arabinose isomerase
Control vs DSS 3-day	5.3.1.9	Glucose-6-phosphate isomerase
Control vs DSS 3-day	5.4.2.12	Phosphoglycerate mutase (2,3-diphosphoglycerate-independent)
Control vs DSS 3-day	5.4.2.2	Phosphoglucomutase (alpha-D-glucose-1,6-bisphosphate-dependent)
Control vs DSS 3-day	5.99.1.3	DNA topoisomerase (ATP-hydrolyzing)
Control vs DSS 3-day	6.1.1.10	Methionine--tRNA ligase
Control vs DSS 3-day	6.1.1.11	Serine--tRNA ligase
Control vs DSS 3-day	6.1.1.12	Aspartate--tRNA ligase
Control vs DSS 3-day	6.1.1.14	Glycine--tRNA ligase
Control vs DSS 3-day	6.1.1.20	Phenylalanine--tRNA ligase
Control vs DSS 3-day	6.1.1.22	Asparagine--tRNA ligase
Control vs DSS 3-day	6.1.1.5	Isoleucine--tRNA ligase
Control vs DSS 3-day	6.3.2.6	Phosphoribosylaminoimidazolesuccinocarboxamide synthase
Control vs DSS 3-day	6.3.5.3	Phosphoribosylformylglycinamidine synthase
Control vs DSS 3-day	6.3.5.5	Carbamoyl-phosphate synthase (glutamine-hydrolyzing)
Control vs DSS 3-day	7.1.2.2	NO_NAME
Control vs DSS 3-day	7.2.1.1	NO_NAME
Control vs DSS 3-day	1.1.1.169	2-dehydropantoate 2-reductase
Control vs DSS 3-day	1.1.1.205	IMP dehydrogenase
Control vs DSS 3-day	1.1.1.22	UDP-glucose 6-dehydrogenase
Control vs DSS 3-day	1.1.1.25	Shikimate dehydrogenase
Control vs DSS 3-day	1.1.1.262	4-hydroxythreonine-4-phosphate dehydrogenase
Control vs DSS 3-day	1.1.1.271	GDP-L-fucose synthase
Control vs DSS 3-day	1.1.1.290	4-phosphoerythronate dehydrogenase
Control vs DSS 3-day	1.1.1.37	Malate dehydrogenase
Control vs DSS 3-day	1.1.1.86	Ketol-acid reductoisomerase (NADP(+))
Control vs DSS 3-day	1.1.1.95	Phosphoglycerate dehydrogenase
Control vs DSS 3-day	1.11.1.1	NADH peroxidase
Control vs DSS 3-day	1.11.1.15	Peroxiredoxin
Control vs DSS 3-day	1.15.1.1	Superoxide dismutase
Control vs DSS 3-day	1.17.4.1	Ribonucleoside-diphosphate reductase
Control vs DSS 3-day	1.17.4.2	Ribonucleoside-triphosphate reductase
Control vs DSS 3-day	1.2.1.11	Aspartate-semialdehyde dehydrogenase
Control vs DSS 3-day	1.3.1.1	Dihydrouracil dehydrogenase (NAD(+))
Control vs DSS 3-day	1.3.1.14	Dihydroorotate dehydrogenase (NAD(+))
Control vs DSS 3-day	1.3.1.9	Enoyl-[acyl-carrier-protein] reductase (NADH)
Control vs DSS 3-day	1.3.98.1	Dihydroorotate oxidase (fumarate)
Control vs DSS 3-day	1.4.1.13	Glutamate synthase (NADPH)
Control vs DSS 3-day	1.4.1.16	Diaminopimelate dehydrogenase
Control vs DSS 3-day	1.4.1.2	Glutamate dehydrogenase
Control vs DSS 3-day	1.4.1.3	Glutamate dehydrogenase (NAD(P)(+))
Control vs DSS 3-day	1.4.4.2	Glycine dehydrogenase (aminomethyl-transferring)

Control vs DSS 3-day	1.6.5.11	NADH dehydrogenase (quinone)
Control vs DSS 3-day	2.1.1.163	Demethylmenaquinone methyltransferase
Control vs DSS 3-day	2.1.1.182	16S rRNA (adenine(1518)-N(6)/adenine(1519)-N(6))-dimethyltransferase
Control vs DSS 3-day	2.1.1.192	23S rRNA (adenine(2503)-C(2))-methyltransferase
Control vs DSS 3-day	2.1.1.228	tRNA (guanine(37)-N(1))-methyltransferase
Control vs DSS 3-day	2.1.2.1	Glycine hydroxymethyltransferase
Control vs DSS 3-day	2.1.2.3	Phosphoribosylaminoimidazolecarboxamide formyltransferase
Control vs DSS 3-day	2.1.3.2	Aspartate carbamoyltransferase
Control vs DSS 3-day	2.1.3.9	N-acetylornithine carbamoyltransferase
Control vs DSS 3-day	2.2.1.7	1-deoxy-D-xylulose-5-phosphate synthase
Control vs DSS 3-day	2.3.1.129	Acyl-[acyl-carrier-protein]--UDP-N-acetylglucosamine O-acyltransferase
Control vs DSS 3-day	2.3.1.179	Beta-ketoacyl-[acyl-carrier-protein] synthase II
Control vs DSS 3-day	2.3.1.180	Beta-ketoacyl-[acyl-carrier-protein] synthase III
Control vs DSS 3-day	2.3.1.234	N(6)-L-threonylcarbamoyladenine synthase
Control vs DSS 3-day	2.3.1.47	8-amino-7-oxononanoate synthase
Control vs DSS 3-day	2.3.1.51	1-acylglycerol-3-phosphate O-acyltransferase
Control vs DSS 3-day	2.3.3.13	2-isopropylmalate synthase
Control vs DSS 3-day	2.4.1.18	1,4-alpha-glucan branching enzyme
Control vs DSS 3-day	2.4.1.182	Lipid-A-disaccharide synthase
Control vs DSS 3-day	2.4.1.21	Starch synthase
Control vs DSS 3-day	2.4.1.212	Hyaluronan synthase
Control vs DSS 3-day	2.4.2.10	Orotate phosphoribosyltransferase
Control vs DSS 3-day	2.4.2.14	Amidophosphoribosyltransferase
Control vs DSS 3-day	2.4.2.19	Nicotinate-nucleotide diphosphorylase (carboxylating)
Control vs DSS 3-day	2.5.1.6	Methionine adenosyltransferase
Control vs DSS 3-day	2.5.1.7	UDP-N-acetylglucosamine 1-carboxyvinyltransferase
Control vs DSS 3-day	2.5.1.75	tRNA dimethylallyltransferase
Control vs DSS 3-day	2.5.1.78	6,7-dimethyl-8-ribityllumazine synthase
Control vs DSS 3-day	2.6.1.16	Glutamine--fructose-6-phosphate transaminase (isomerizing)
Control vs DSS 3-day	2.6.1.42	Branched-chain-amino-acid transaminase
Control vs DSS 3-day	2.6.1.52	Phosphoserine transaminase
Control vs DSS 3-day	2.7.1.11	6-phosphofructokinase
Control vs DSS 3-day	2.7.1.40	Pyruvate kinase
Control vs DSS 3-day	2.7.1.69	Protein-N(pi)-phosphohistidine--sugar phosphotransferase
Control vs DSS 3-day	2.7.1.90	Diphosphate--fructose-6-phosphate 1-phosphotransferase
Control vs DSS 3-day	2.7.2.1	Acetate kinase
Control vs DSS 3-day	2.7.2.3	Phosphoglycerate kinase
Control vs DSS 3-day	2.7.2.4	Aspartate kinase
Control vs DSS 3-day	2.7.4.6	Nucleoside-diphosphate kinase
Control vs DSS 3-day	2.7.6.1	Ribose-phosphate diphosphokinase
Control vs DSS 3-day	2.7.7.7	DNA-directed DNA polymerase
Control vs DSS 3-day	2.7.7.8	Polyribonucleotide nucleotidyltransferase
Control vs DSS 3-day	2.8.1.7	Cysteine desulfurase

Control vs DSS 3-day	2.8.1.8	Lipoyl synthase
Control vs DSS 3-day	3.1.21.3	Type I site-specific deoxyribonuclease
Control vs DSS 3-day	3.1.26.5	Ribonuclease P
Control vs DSS 3-day	3.1.3.11	Fructose-bisphosphatase
Control vs DSS 3-day	3.1.4.46	Glycerophosphodiester phosphodiesterase
Control vs DSS 3-day	3.2.1.135	Neopullulanase
Control vs DSS 3-day	3.2.1.172	Unsaturated rhamnogalacturonyl hydrolase
Control vs DSS 3-day	3.2.1.22	Alpha-galactosidase
Control vs DSS 3-day	3.2.1.23	Beta-galactosidase
Control vs DSS 3-day	3.2.2.27	Uracil-DNA glycosylase
Control vs DSS 3-day	3.4.11.18	Methionyl aminopeptidase
Control vs DSS 3-day	3.4.13.18	Cytosol nonspecific dipeptidase
Control vs DSS 3-day	3.4.14.12	Xaa-Xaa-Pro tripeptidyl-peptidase
Control vs DSS 3-day	3.4.21.53	Endopeptidase La
Control vs DSS 3-day	3.4.21.89	Signal peptidase I
Control vs DSS 3-day	3.5.1.10	Formyltetrahydrofolate deformylase
Control vs DSS 3-day	3.5.1.108	UDP-3-O-acyl-N-acetylglucosamine deacetylase
Control vs DSS 3-day	3.5.1.25	N-acetylglucosamine-6-phosphate deacetylase
Control vs DSS 3-day	3.5.4.10	IMP cyclohydrolase
Control vs DSS 3-day	3.5.99.6	Glucosamine-6-phosphate deaminase
Control vs DSS 3-day	3.6.3.41	Heme-transporting ATPase
Control vs DSS 3-day	3.6.4.12	DNA helicase
Control vs DSS 3-day	3.6.4.13	RNA helicase
Control vs DSS 3-day	4.1.1.23	Orotidine-5'-phosphate decarboxylase
Control vs DSS 3-day	4.1.1.36	Phosphopantothenoylcysteine decarboxylase
Control vs DSS 3-day	4.1.3.36	1,4-dihydroxy-2-naphthoyl-CoA synthase
Control vs DSS 3-day	4.2.1.11	Phosphopyruvate hydratase
Control vs DSS 3-day	4.2.1.17	Enoyl-CoA hydratase
Control vs DSS 3-day	4.2.1.2	Fumarate hydratase
Control vs DSS 3-day	4.2.1.33	3-isopropylmalate dehydratase
Control vs DSS 3-day	4.2.1.47	GDP-mannose 4,6-dehydratase
Control vs DSS 3-day	4.2.1.59	3-hydroxyacyl-[acyl-carrier-protein] dehydratase
Control vs DSS 3-day	4.2.1.8	Mannonate dehydratase
Control vs DSS 3-day	4.2.2.2	Pectate lyase
Control vs DSS 3-day	4.2.3.3	Methylglyoxal synthase
Control vs DSS 3-day	4.3.1.17	L-serine ammonia-lyase
Control vs DSS 3-day	4.3.3.7	4-hydroxy-tetrahydrodipicolinate synthase
Control vs DSS 3-day	5.2.1.8	Peptidylprolyl isomerase
Control vs DSS 3-day	5.3.1.17	5-dehydro-4-deoxy-D-glucuronate isomerase
Control vs DSS 3-day	5.3.1.25	L-fucose isomerase
Control vs DSS 3-day	5.4.2.12	Phosphoglycerate mutase (2,3-diphosphoglycerate-independent)
Control vs DSS 3-day	5.4.2.2	Phosphoglucomutase (alpha-D-glucose-1,6-bisphosphate-dependent)
Control vs DSS 3-day	5.99.1.3	DNA topoisomerase (ATP-hydrolyzing)

Control vs DSS 3-day	6.1.1.10	Methionine--tRNA ligase
Control vs DSS 3-day	6.1.1.11	Serine--tRNA ligase
Control vs DSS 3-day	6.1.1.12	Aspartate--tRNA ligase
Control vs DSS 3-day	6.1.1.14	Glycine--tRNA ligase
Control vs DSS 3-day	6.1.1.15	Proline--tRNA ligase
Control vs DSS 3-day	6.1.1.18	Glutamine--tRNA ligase
Control vs DSS 3-day	6.1.1.20	Phenylalanine--tRNA ligase
Control vs DSS 3-day	6.1.1.22	Asparagine--tRNA ligase
Control vs DSS 3-day	6.1.1.4	Leucine--tRNA ligase
Control vs DSS 3-day	6.1.1.5	Isoleucine--tRNA ligase
Control vs DSS 3-day	6.1.1.6	Lysine--tRNA ligase
Control vs DSS 3-day	6.1.1.7	Alanine--tRNA ligase
Control vs DSS 3-day	6.1.1.9	Valine--tRNA ligase
Control vs DSS 3-day	6.3.2.4	D-alanine--D-alanine ligase
Control vs DSS 3-day	6.3.2.5	Phosphopantothenate--cysteine ligase
Control vs DSS 3-day	6.3.2.6	Phosphoribosylaminoimidazolesuccinocarboxamide synthase
Control vs DSS 3-day	6.3.2.9	UDP-N-acetylmuramoyl-L-alanine--D-glutamate ligase
Control vs DSS 3-day	6.3.4.13	Phosphoribosylamine--glycine ligase
Control vs DSS 3-day	6.3.4.3	Formate--tetrahydrofolate ligase
Control vs DSS 3-day	6.3.5.3	Phosphoribosylformylglycinamidine synthase
Control vs DSS 3-day	6.3.5.5	Carbamoyl-phosphate synthase (glutamine-hydrolyzing)
Control vs DSS 3-day	7.1.2.2	NO_NAME
Control vs DSS 3-day	7.2.1.1	NO_NAME
Control vs DSS 3-day	1.1.1.169	2-dehydropantoate 2-reductase
Control vs DSS 3-day	1.1.1.37	Malate dehydrogenase
Control vs DSS 3-day	1.1.1.42	Isocitrate dehydrogenase (NADP(+))
Control vs DSS 3-day	1.1.1.44	Phosphogluconate dehydrogenase (NADP(+)-dependent, decarboxylating)
Control vs DSS 3-day	1.1.1.58	Tagaturonate reductase
Control vs DSS 3-day	1.1.1.95	Phosphoglycerate dehydrogenase
Control vs DSS 3-day	1.1.9.1	Alcohol dehydrogenase (azurin)
Control vs DSS 3-day	1.11.1.1	NADH peroxidase
Control vs DSS 3-day	1.11.1.15	Peroxiredoxin
Control vs DSS 3-day	1.17.4.2	Ribonucleoside-triphosphate reductase
Control vs DSS 3-day	1.17.7.3	(E)-4-hydroxy-3-methylbut-2-enyl-diphosphate synthase (flavodoxin)
Control vs DSS 3-day	1.3.1.1	Dihydrouracil dehydrogenase (NAD(+))
Control vs DSS 3-day	1.3.98.1	Dihydroorotate oxidase (fumarate)
Control vs DSS 3-day	1.3.99.5	3-oxo-5-alpha-steroid 4-dehydrogenase (acceptor)
Control vs DSS 3-day	1.4.1.13	Glutamate synthase (NADPH)
Control vs DSS 3-day	1.4.1.16	Diaminopimelate dehydrogenase
Control vs DSS 3-day	1.4.1.2	Glutamate dehydrogenase
Control vs DSS 3-day	1.4.1.21	Aspartate dehydrogenase
Control vs DSS 3-day	1.4.3.5	Pyridoxal 5'-phosphate synthase
Control vs DSS 3-day	1.4.4.2	Glycine dehydrogenase (aminomethyl-transferring)

Control vs DSS 3-day	1.5.1.2	Pyrroline-5-carboxylate reductase
Control vs DSS 3-day	1.6.5.11	NADH dehydrogenase (quinone)
Control vs DSS 3-day	1.6.99.1	NADPH dehydrogenase
Control vs DSS 3-day	1.8.1.4	Dihydrolipoyl dehydrogenase
Control vs DSS 3-day	1.8.1.9	Thioredoxin-disulfide reductase
Control vs DSS 3-day	2.1.1.13	Methionine synthase
Control vs DSS 3-day	2.1.1.177	23S rRNA (pseudouridine(1915)-N(3))-methyltransferase
Control vs DSS 3-day	2.1.1.185	23S rRNA (guanosine(2251)-2'-O)-methyltransferase
Control vs DSS 3-day	2.1.1.192	23S rRNA (adenine(2503)-C(2))-methyltransferase
Control vs DSS 3-day	2.1.1.193	16S rRNA (uracil(1498)-N(3))-methyltransferase
Control vs DSS 3-day	2.1.1.37	DNA (cytosine-5-)-methyltransferase
Control vs DSS 3-day	2.1.1.63	Methylated-DNA--[protein]-cysteine S-methyltransferase
Control vs DSS 3-day	2.1.1.72	Site-specific DNA-methyltransferase (adenine-specific)
Control vs DSS 3-day	2.1.2.2	Phosphoribosylglycinamide formyltransferase
Control vs DSS 3-day	2.2.1.7	1-deoxy-D-xylulose-5-phosphate synthase
Control vs DSS 3-day	2.3.1.31	Homoserine O-acetyltransferase
Control vs DSS 3-day	2.3.1.47	8-amino-7-oxononanoate synthase
Control vs DSS 3-day	2.3.3.13	2-isopropylmalate synthase
Control vs DSS 3-day	2.4.1.21	Starch synthase
Control vs DSS 3-day	2.4.1.212	Hyaluronan synthase
Control vs DSS 3-day	2.4.1.320	1,4-beta-mannosyl-N-acetylglucosamine phosphorylase
Control vs DSS 3-day	2.5.1.47	Cysteine synthase
Control vs DSS 3-day	2.5.1.6	Methionine adenosyltransferase
Control vs DSS 3-day	2.5.1.72	Quinolinate synthase
Control vs DSS 3-day	2.5.1.75	tRNA dimethylallyltransferase
Control vs DSS 3-day	2.6.1.16	Glutamine--fructose-6-phosphate transaminase (isomerizing)
Control vs DSS 3-day	2.6.1.52	Phosphoserine transaminase
Control vs DSS 3-day	2.7.1.15	Ribokinase
Control vs DSS 3-day	2.7.1.26	Riboflavin kinase
Control vs DSS 3-day	2.7.1.33	Pantothenate kinase
Control vs DSS 3-day	2.7.1.35	Pyridoxal kinase
Control vs DSS 3-day	2.7.1.40	Pyruvate kinase
Control vs DSS 3-day	2.7.1.5	Rhamnulokinase
Control vs DSS 3-day	2.7.1.69	Protein-N(pi)-phosphohistidine--sugar phosphotransferase
Control vs DSS 3-day	2.7.1.71	Shikimate kinase
Control vs DSS 3-day	2.7.1.90	Diphosphate--fructose-6-phosphate 1-phosphotransferase
Control vs DSS 3-day	2.7.13.3	Histidine kinase
Control vs DSS 3-day	2.7.2.1	Acetate kinase
Control vs DSS 3-day	2.7.2.11	Glutamate 5-kinase
Control vs DSS 3-day	2.7.2.3	Phosphoglycerate kinase
Control vs DSS 3-day	2.7.4.25	(d)CMP kinase
Control vs DSS 3-day	2.7.7.13	Mannose-1-phosphate guanylyltransferase
Control vs DSS 3-day	2.7.7.2	FAD synthetase

Control vs DSS 3-day	2.7.7.49	RNA-directed DNA polymerase
Control vs DSS 3-day	2.7.7.6	DNA-directed RNA polymerase
Control vs DSS 3-day	2.7.7.7	DNA-directed DNA polymerase
Control vs DSS 3-day	2.7.7.8	Polyribonucleotide nucleotidyltransferase
Control vs DSS 3-day	2.8.1.8	Lipoyl synthase
Control vs DSS 3-day	3.1.1.11	Pectinesterase
Control vs DSS 3-day	3.1.1.29	Aminoacyl-tRNA hydrolase
Control vs DSS 3-day	3.1.11.2	Exodeoxyribonuclease III
Control vs DSS 3-day	3.1.11.6	Exodeoxyribonuclease VII
Control vs DSS 3-day	3.1.13.1	Exoribonuclease II
Control vs DSS 3-day	3.1.21.2	Deoxyribonuclease IV
Control vs DSS 3-day	3.1.21.3	Type I site-specific deoxyribonuclease
Control vs DSS 3-day	3.1.3.1	Alkaline phosphatase
Control vs DSS 3-day	3.1.3.25	Inositol-phosphate phosphatase
Control vs DSS 3-day	3.1.6.1	Arylsulfatase
Control vs DSS 3-day	3.2.1.131	Xylan alpha-1,2-glucuronosidase
Control vs DSS 3-day	3.2.1.172	Unsaturated rhamnogalacturonyl hydrolase
Control vs DSS 3-day	3.2.1.177	Alpha-D-xyloside xylohydrolase
Control vs DSS 3-day	3.2.1.21	Beta-glucosidase
Control vs DSS 3-day	3.2.1.22	Alpha-galactosidase
Control vs DSS 3-day	3.2.1.23	Beta-galactosidase
Control vs DSS 3-day	3.2.1.31	Beta-glucuronidase
Control vs DSS 3-day	3.2.1.37	Xylan 1,4-beta-xylosidase
Control vs DSS 3-day	3.2.1.51	Alpha-L-fucosidase
Control vs DSS 3-day	3.2.1.55	Non-reducing end alpha-L-arabinofuranosidase
Control vs DSS 3-day	3.2.1.8	Endo-1,4-beta-xylanase
Control vs DSS 3-day	3.2.1.82	Exo-poly-alpha-galacturonosidase
Control vs DSS 3-day	3.4.11.9	Xaa-Pro aminopeptidase
Control vs DSS 3-day	3.4.13.20	Beta-Ala-His dipeptidase
Control vs DSS 3-day	3.4.14.12	Xaa-Xaa-Pro tripeptidyl-peptidase
Control vs DSS 3-day	3.4.14.4	Dipeptidyl-peptidase III
Control vs DSS 3-day	3.4.15.5	Peptidyl-dipeptidase Dcp
Control vs DSS 3-day	3.4.16.4	Serine-type D-Ala-D-Ala carboxypeptidase
Control vs DSS 3-day	3.4.21.107	Peptidase Do
Control vs DSS 3-day	3.4.21.53	Endopeptidase La
Control vs DSS 3-day	3.4.21.89	Signal peptidase I
Control vs DSS 3-day	3.5.1.10	Formyltetrahydrofolate deformylase
Control vs DSS 3-day	3.5.1.100	(R)-amidase
Control vs DSS 3-day	3.5.1.104	Peptidoglycan-N-acetylglucosamine deacetylase
Control vs DSS 3-day	3.5.1.2	Glutaminase
Control vs DSS 3-day	3.5.1.25	N-acetylglucosamine-6-phosphate deacetylase
Control vs DSS 3-day	3.5.1.28	N-acetylmuramoyl-L-alanine amidase
Control vs DSS 3-day	3.5.2.3	Dihydroorotase

Control vs DSS 3-day	3.5.2.7	Imidazolonepropionase
Control vs DSS 3-day	3.5.4.16	GTP cyclohydrolase I
Control vs DSS 3-day	3.5.4.25	GTP cyclohydrolase II
Control vs DSS 3-day	3.5.99.6	Glucosamine-6-phosphate deaminase
Control vs DSS 3-day	3.6.3.54	Cu(+) exporting ATPase
Control vs DSS 3-day	3.6.4.12	DNA helicase
Control vs DSS 3-day	4.1.1.19	Arginine decarboxylase
Control vs DSS 3-day	4.1.1.20	Diaminopimelate decarboxylase
Control vs DSS 3-day	4.1.1.36	Phosphopantothenoylcysteine decarboxylase
Control vs DSS 3-day	4.1.1.65	Phosphatidylserine decarboxylase
Control vs DSS 3-day	4.1.2.19	Rhamnulose-1-phosphate aldolase
Control vs DSS 3-day	4.1.99.12	3,4-dihydroxy-2-butanone-4-phosphate synthase
Control vs DSS 3-day	4.2.1.10	3-dehydroquinate dehydratase
Control vs DSS 3-day	4.2.1.126	N-acetylmuramic acid 6-phosphate etherase
Control vs DSS 3-day	4.2.1.136	ADP-dependent NAD(P)H-hydrate dehydratase
Control vs DSS 3-day	4.2.1.2	Fumarate hydratase
Control vs DSS 3-day	4.2.1.3	Aconitate hydratase
Control vs DSS 3-day	4.2.1.33	3-isopropylmalate dehydratase
Control vs DSS 3-day	4.2.2.2	Pectate lyase
Control vs DSS 3-day	4.2.3.1	Threonine synthase
Control vs DSS 3-day	4.3.2.10	NO_NAME
Control vs DSS 3-day	5.1.3.1	Ribulose-phosphate 3-epimerase
Control vs DSS 3-day	5.1.3.3	Aldose 1-epimerase
Control vs DSS 3-day	5.1.99.6	NAD(P)H-hydrate epimerase
Control vs DSS 3-day	5.2.1.8	Peptidylprolyl isomerase
Control vs DSS 3-day	5.3.1.12	Glucuronate isomerase
Control vs DSS 3-day	5.3.1.14	L-rhamnose isomerase
Control vs DSS 3-day	5.3.1.16	isomerase
Control vs DSS 3-day	5.3.1.17	5-dehydro-4-deoxy-D-glucuronate isomerase
Control vs DSS 3-day	5.3.1.9	Glucose-6-phosphate isomerase
Control vs DSS 3-day	5.4.99.18	5-(carboxyamino)imidazole ribonucleotide mutase
Control vs DSS 3-day	5.4.99.25	tRNA pseudouridine(55) synthase
Control vs DSS 3-day	5.99.1.3	DNA topoisomerase (ATP-hydrolyzing)
Control vs DSS 3-day	6.1.1.12	Aspartate--tRNA ligase
Control vs DSS 3-day	6.1.1.18	Glutamine--tRNA ligase
Control vs DSS 3-day	6.1.1.3	Threonine--tRNA ligase
Control vs DSS 3-day	6.1.1.5	Isoleucine--tRNA ligase
Control vs DSS 3-day	6.1.1.6	Lysine--tRNA ligase
Control vs DSS 3-day	6.2.1.30	Phenylacetate--CoA ligase
Control vs DSS 3-day	6.3.1.2	Glutamate--ammonia ligase
Control vs DSS 3-day	6.3.2.5	Phosphopantothenate--cysteine ligase
Control vs DSS 3-day	6.3.4.13	Phosphoribosylamine--glycine ligase
Control vs DSS 3-day	6.3.4.2	CTP synthase (glutamine hydrolyzing)

Control vs DSS 3-day	6.3.5.5	Carbamoyl-phosphate synthase (glutamine-hydrolyzing)
Control vs DSS 3-day	7.1.2.2	NO_NAME
Control vs DSS 3-day	7.2.1.1	NO_NAME
Control vs DSS 3-day	2.7.7.6	DNA-directed RNA polymerase
Control vs DSS 3-day	1.1.1.37	Malate dehydrogenase
Control vs DSS 3-day	1.11.1.1	NADH peroxidase
Control vs DSS 3-day	1.4.1.2	Glutamate dehydrogenase
Control vs DSS 3-day	2.6.1.52	Phosphoserine transaminase
Control vs DSS 3-day	2.7.7.6	DNA-directed RNA polymerase
Control vs DSS 3-day	3.4.11.18	Methionyl aminopeptidase
Control vs DSS 3-day	4.1.1.49	Phosphoenolpyruvate carboxykinase (ATP)
Control vs DSS 3-day	4.3.2.1	Argininosuccinate lyase
Control vs DSS 3-day	5.3.1.5	Xylose isomerase
Control vs DSS 3-day	6.1.1.15	Proline--tRNA ligase
Control vs DSS 3-day	6.1.1.22	Asparagine--tRNA ligase
Control vs DSS 3-day	6.3.5.5	Carbamoyl-phosphate synthase (glutamine-hydrolyzing)
Control vs DSS 3-day	1.3.1.9	Enoyl-[acyl-carrier-protein] reductase (NADH)
Control vs DSS 3-day	2.7.7.6	DNA-directed RNA polymerase
Control vs DSS 3-day	4.3.2.1	Argininosuccinate lyase
Control vs DSS 3-day	5.3.1.12	Glucuronate isomerase
Control vs DSS 3-day	6.1.1.22	Asparagine--tRNA ligase
Control vs DSS 3-day	1.1.1.86	Ketol-acid reductoisomerase (NADP(+))
Control vs DSS 3-day	1.1.1.95	Phosphoglycerate dehydrogenase
Control vs DSS 3-day	1.11.1.1	NADH peroxidase
Control vs DSS 3-day	1.11.1.15	Peroxiredoxin
Control vs DSS 3-day	1.15.1.1	Superoxide dismutase
Control vs DSS 3-day	1.2.1.11	Aspartate-semialdehyde dehydrogenase
Control vs DSS 3-day	1.3.1.9	Enoyl-[acyl-carrier-protein] reductase (NADH)
Control vs DSS 3-day	1.4.1.16	Diaminopimelate dehydrogenase
Control vs DSS 3-day	2.1.1.72	Site-specific DNA-methyltransferase (adenine-specific)
Control vs DSS 3-day	2.1.2.1	Glycine hydroxymethyltransferase
Control vs DSS 3-day	2.1.2.3	Phosphoribosylaminoimidazolecarboxamide formyltransferase
Control vs DSS 3-day	2.3.1.179	Beta-ketoacyl-[acyl-carrier-protein] synthase II
Control vs DSS 3-day	2.3.1.180	Beta-ketoacyl-[acyl-carrier-protein] synthase III
Control vs DSS 3-day	2.3.1.47	8-amino-7-oxononanoate synthase
Control vs DSS 3-day	2.3.1.50	Serine C-palmitoyltransferase
Control vs DSS 3-day	2.4.2.29	tRNA-guanine(34) transglycosylase
Control vs DSS 3-day	2.4.99.17	S-adenosylmethionine
Control vs DSS 3-day	2.6.1.52	Phosphoserine transaminase
Control vs DSS 3-day	2.7.1.40	Pyruvate kinase
Control vs DSS 3-day	2.7.1.90	Diphosphate--fructose-6-phosphate 1-phosphotransferase
Control vs DSS 3-day	2.7.13.3	Histidine kinase
Control vs DSS 3-day	2.7.2.3	Phosphoglycerate kinase

Control vs DSS 3-day	2.7.2.4	Aspartate kinase
Control vs DSS 3-day	2.7.6.1	Ribose-phosphate diphosphokinase
Control vs DSS 3-day	2.7.7.6	DNA-directed RNA polymerase
Control vs DSS 3-day	2.7.7.8	Polyribonucleotide nucleotidyltransferase
Control vs DSS 3-day	3.1.21.3	Type I site-specific deoxyribonuclease
Control vs DSS 3-day	3.4.11.18	Methionyl aminopeptidase
Control vs DSS 3-day	3.5.1.108	UDP-3-O-acyl-N-acetylglucosamine deacetylase
Control vs DSS 3-day	3.5.4.10	IMP cyclohydrolase
Control vs DSS 3-day	3.5.99.6	Glucosamine-6-phosphate deaminase
Control vs DSS 3-day	3.6.4.12	DNA helicase
Control vs DSS 3-day	4.1.1.15	Glutamate decarboxylase
Control vs DSS 3-day	4.1.1.23	Orotidine-5'-phosphate decarboxylase
Control vs DSS 3-day	4.1.1.49	Phosphoenolpyruvate carboxykinase (ATP)
Control vs DSS 3-day	4.2.1.11	Phosphopyruvate hydratase
Control vs DSS 3-day	4.2.1.59	3-hydroxyacyl-[acyl-carrier-protein] dehydratase
Control vs DSS 3-day	5.2.1.8	Peptidylprolyl isomerase
Control vs DSS 3-day	5.3.1.1	Triose-phosphate isomerase
Control vs DSS 3-day	5.3.1.12	Glucuronate isomerase
Control vs DSS 3-day	5.3.1.9	Glucose-6-phosphate isomerase
Control vs DSS 3-day	5.4.2.12	Phosphoglycerate mutase (2,3-diphosphoglycerate-independent)
Control vs DSS 3-day	5.99.1.3	DNA topoisomerase (ATP-hydrolyzing)
Control vs DSS 3-day	6.1.1.11	Serine--tRNA ligase
Control vs DSS 3-day	6.1.1.14	Glycine--tRNA ligase
Control vs DSS 3-day	6.1.1.15	Proline--tRNA ligase
Control vs DSS 3-day	6.1.1.20	Phenylalanine--tRNA ligase
Control vs DSS 3-day	6.3.5.3	Phosphoribosylformylglycinamidine synthase
Control vs DSS 3-day	6.3.5.5	Carbamoyl-phosphate synthase (glutamine-hydrolyzing)
Control vs DSS 3-day	7.1.2.2	NO_NAME
Control vs DSS 3-day	7.2.1.1	NO_NAME
Control vs DSS 3-day	1.1.1.100	3-oxoacyl-[acyl-carrier-protein] reductase
Control vs DSS 3-day	1.1.1.37	Malate dehydrogenase
Control vs DSS 3-day	2.7.1.90	Diphosphate--fructose-6-phosphate 1-phosphotransferase
Control vs DSS 3-day	2.7.7.6	DNA-directed RNA polymerase
Control vs DSS 3-day	2.7.7.8	Polyribonucleotide nucleotidyltransferase
Control vs DSS 3-day	3.4.11.18	Methionyl aminopeptidase
Control vs DSS 3-day	3.5.99.6	Glucosamine-6-phosphate deaminase
Control vs DSS 3-day	5.99.1.3	DNA topoisomerase (ATP-hydrolyzing)
Control vs DSS 3-day	1.1.1.100	3-oxoacyl-[acyl-carrier-protein] reductase
Control vs DSS 3-day	1.1.1.122	D-threo-aldose 1-dehydrogenase
Control vs DSS 3-day	1.1.1.169	2-dehydropantoate 2-reductase
Control vs DSS 3-day	1.1.1.205	IMP dehydrogenase
Control vs DSS 3-day	1.1.1.22	UDP-glucose 6-dehydrogenase
Control vs DSS 3-day	1.1.1.267	1-deoxy-D-xylulose-5-phosphate reductoisomerase

Control vs DSS 3-day	1.1.1.290	4-phosphoerythronate dehydrogenase
Control vs DSS 3-day	1.1.1.37	Malate dehydrogenase
Control vs DSS 3-day	1.1.1.42	Isocitrate dehydrogenase (NADP(+))
Control vs DSS 3-day	1.1.1.44	Phosphogluconate dehydrogenase (NADP(+)-dependent, decarboxylating)
Control vs DSS 3-day	1.1.1.58	Tagaturonate reductase
Control vs DSS 3-day	1.1.1.85	3-isopropylmalate dehydrogenase
Control vs DSS 3-day	1.1.1.86	Ketol-acid reductoisomerase (NADP(+))
Control vs DSS 3-day	1.1.1.95	Phosphoglycerate dehydrogenase
Control vs DSS 3-day	1.11.1.1	NADH peroxidase
Control vs DSS 3-day	1.15.1.1	Superoxide dismutase
Control vs DSS 3-day	1.16.3.2	Bacterial non-heme ferritin
Control vs DSS 3-day	1.17.4.1	Ribonucleoside-diphosphate reductase
Control vs DSS 3-day	1.17.4.2	Ribonucleoside-triphosphate reductase
Control vs DSS 3-day	1.2.1.11	Aspartate-semialdehyde dehydrogenase
Control vs DSS 3-day	1.2.1.38	N-acetyl-gamma-glutamyl-phosphate reductase
Control vs DSS 3-day	1.2.7.8	Indolepyruvate ferredoxin oxidoreductase
Control vs DSS 3-day	1.3.1.1	Dihydrouracil dehydrogenase (NAD(+))
Control vs DSS 3-day	1.3.1.14	Dihydroorotate dehydrogenase (NAD(+))
Control vs DSS 3-day	1.3.1.26	Transferred entry
Control vs DSS 3-day	1.3.1.9	Enoyl-[acyl-carrier-protein] reductase (NADH)
Control vs DSS 3-day	1.3.98.1	Dihydroorotate oxidase (fumarate)
Control vs DSS 3-day	1.3.99.5	3-oxo-5-alpha-steroid 4-dehydrogenase (acceptor)
Control vs DSS 3-day	1.4.1.2	Glutamate dehydrogenase
Control vs DSS 3-day	1.4.1.3	Glutamate dehydrogenase (NAD(P)(+))
Control vs DSS 3-day	1.4.3.16	L-aspartate oxidase
Control vs DSS 3-day	1.4.3.5	Pyridoxal 5'-phosphate synthase
Control vs DSS 3-day	1.5.1.5	Methylenetetrahydrofolate dehydrogenase (NADP(+))
Control vs DSS 3-day	1.6.5.11	NADH dehydrogenase (quinone)
Control vs DSS 3-day	1.6.5.3	NADH
Control vs DSS 3-day	1.6.99.1	NADPH dehydrogenase
Control vs DSS 3-day	1.8.1.4	Dihydrolipoyl dehydrogenase
Control vs DSS 3-day	1.8.1.9	Thioredoxin-disulfide reductase
Control vs DSS 3-day	2.1.1.13	Methionine synthase
Control vs DSS 3-day	2.1.1.163	Demethylmenaquinone methyltransferase
Control vs DSS 3-day	2.1.1.182	16S rRNA (adenine(1518)-N(6)/adenine(1519)-N(6))-dimethyltransferase
Control vs DSS 3-day	2.1.1.185	23S rRNA (guanosine(2251)-2'-O)-methyltransferase
Control vs DSS 3-day	2.1.1.191	23S rRNA (cytosine(1962)-C(5))-methyltransferase
Control vs DSS 3-day	2.1.1.192	23S rRNA (adenine(2503)-C(2))-methyltransferase
Control vs DSS 3-day	2.1.1.193	16S rRNA (uracil(1498)-N(3))-methyltransferase
Control vs DSS 3-day	2.1.1.198	16S rRNA (cytidine(1402)-2'-O)-methyltransferase
Control vs DSS 3-day	2.1.1.199	16S rRNA (cytosine(1402)-N(4))-methyltransferase
Control vs DSS 3-day	2.1.1.228	tRNA (guanine(37)-N(1))-methyltransferase
Control vs DSS 3-day	2.1.1.37	DNA (cytosine-5-)-methyltransferase

Control vs DSS 3-day	2.1.2.1	Glycine hydroxymethyltransferase
Control vs DSS 3-day	2.1.2.10	Aminomethyltransferase
Control vs DSS 3-day	2.1.2.2	Phosphoribosylglycinamide formyltransferase
Control vs DSS 3-day	2.1.2.3	Phosphoribosylaminoimidazolecarboxamide formyltransferase
Control vs DSS 3-day	2.1.3.2	Aspartate carbamoyltransferase
Control vs DSS 3-day	2.1.3.9	N-acetylornithine carbamoyltransferase
Control vs DSS 3-day	2.2.1.6	Acetolactate synthase
Control vs DSS 3-day	2.2.1.7	1-deoxy-D-xylulose-5-phosphate synthase
Control vs DSS 3-day	2.3.1.129	Acyl-[acyl-carrier-protein]--UDP-N-acetylglucosamine O-acyltransferase
Control vs DSS 3-day	2.3.1.179	Beta-ketoacyl-[acyl-carrier-protein] synthase II
Control vs DSS 3-day	2.3.1.180	Beta-ketoacyl-[acyl-carrier-protein] synthase III
Control vs DSS 3-day	2.3.1.234	N(6)-L-threonylcarbamoyladenine synthase
Control vs DSS 3-day	2.3.1.31	Homoserine O-acetyltransferase
Control vs DSS 3-day	2.3.1.39	[Acyl-carrier-protein] S-malonyltransferase
Control vs DSS 3-day	2.3.1.47	8-amino-7-oxononanoate synthase
Control vs DSS 3-day	2.3.1.51	1-acylglycerol-3-phosphate O-acyltransferase
Control vs DSS 3-day	2.3.3.13	2-isopropylmalate synthase
Control vs DSS 3-day	2.4.1.18	1,4-alpha-glucan branching enzyme
Control vs DSS 3-day	2.4.1.21	Starch synthase
Control vs DSS 3-day	2.4.1.212	Hyaluronan synthase
Control vs DSS 3-day	2.4.1.320	1,4-beta-mannosyl-N-acetylglucosamine phosphorylase
Control vs DSS 3-day	2.4.2.10	Orotate phosphoribosyltransferase
Control vs DSS 3-day	2.4.2.14	Amidophosphoribosyltransferase
Control vs DSS 3-day	2.4.2.19	Nicotinate-nucleotide diphosphorylase (carboxylating)
Control vs DSS 3-day	2.4.2.29	tRNA-guanine(34) transglycosylase
Control vs DSS 3-day	2.4.2.8	Hypoxanthine phosphoribosyltransferase
Control vs DSS 3-day	2.4.99.17	S-adenosylmethionine
Control vs DSS 3-day	2.5.1.17	Cob(I)yrinic acid a,c-diamide adenosyltransferase
Control vs DSS 3-day	2.5.1.19	3-phosphoshikimate 1-carboxyvinyltransferase
Control vs DSS 3-day	2.5.1.47	Cysteine synthase
Control vs DSS 3-day	2.5.1.7	UDP-N-acetylglucosamine 1-carboxyvinyltransferase
Control vs DSS 3-day	2.5.1.72	Quinolinate synthase
Control vs DSS 3-day	2.5.1.78	6,7-dimethyl-8-ribityllumazine synthase
Control vs DSS 3-day	2.6.1.42	Branched-chain-amino-acid transaminase
Control vs DSS 3-day	2.6.1.52	Phosphoserine transaminase
Control vs DSS 3-day	2.6.1.83	LL-diaminopimelate aminotransferase
Control vs DSS 3-day	2.7.1.11	6-phosphofructokinase
Control vs DSS 3-day	2.7.1.15	Ribokinase
Control vs DSS 3-day	2.7.1.21	Thymidine kinase
Control vs DSS 3-day	2.7.1.35	Pyridoxal kinase
Control vs DSS 3-day	2.7.1.40	Pyruvate kinase
Control vs DSS 3-day	2.7.1.48	Uridine kinase
Control vs DSS 3-day	2.7.1.5	Rhamnulokinase

Control vs DSS 3-day	2.7.1.69	Protein-N(pi)-phosphohistidine--sugar phosphotransferase
Control vs DSS 3-day	2.7.1.71	Shikimate kinase
Control vs DSS 3-day	2.7.1.90	Diphosphate--fructose-6-phosphate 1-phosphotransferase
Control vs DSS 3-day	2.7.13.3	Histidine kinase
Control vs DSS 3-day	2.7.2.1	Acetate kinase
Control vs DSS 3-day	2.7.2.3	Phosphoglycerate kinase
Control vs DSS 3-day	2.7.2.4	Aspartate kinase
Control vs DSS 3-day	2.7.4.3	Adenylate kinase
Control vs DSS 3-day	2.7.4.6	Nucleoside-diphosphate kinase
Control vs DSS 3-day	2.7.6.1	Ribose-phosphate diphosphokinase
Control vs DSS 3-day	2.7.7.13	Mannose-1-phosphate guanylyltransferase
Control vs DSS 3-day	2.7.7.7	DNA-directed DNA polymerase
Control vs DSS 3-day	2.7.7.72	CCA tRNA nucleotidyltransferase
Control vs DSS 3-day	2.7.7.8	Polyribonucleotide nucleotidyltransferase
Control vs DSS 3-day	2.7.7.85	Diadenylate cyclase
Control vs DSS 3-day	2.7.8.8	CDP-diacylglycerol--serine O-phosphatidyltransferase
Control vs DSS 3-day	2.8.4.3	tRNA-2-methylthio-N(6)-dimethylallyladenosine synthase
Control vs DSS 3-day	3.1.1.11	Pectinesterase
Control vs DSS 3-day	3.1.1.31	6-phosphogluconolactonase
Control vs DSS 3-day	3.1.13.1	Exoribonuclease II
Control vs DSS 3-day	3.1.21.3	Type I site-specific deoxyribonuclease
Control vs DSS 3-day	3.1.3.1	Alkaline phosphatase
Control vs DSS 3-day	3.1.3.11	Fructose-bisphosphatase
Control vs DSS 3-day	3.1.3.25	Inositol-phosphate phosphatase
Control vs DSS 3-day	3.1.3.5	5'-nucleotidase
Control vs DSS 3-day	3.1.6.1	Arylsulfatase
Control vs DSS 3-day	3.2.1.172	Unsaturated rhamnogalacturonyl hydrolase
Control vs DSS 3-day	3.2.1.177	Alpha-D-xyloside xylohydrolase
Control vs DSS 3-day	3.2.1.21	Beta-glucosidase
Control vs DSS 3-day	3.2.1.22	Alpha-galactosidase
Control vs DSS 3-day	3.2.1.23	Beta-galactosidase
Control vs DSS 3-day	3.2.1.37	Xylan 1,4-beta-xylosidase
Control vs DSS 3-day	3.2.1.55	Non-reducing end alpha-L-arabinofuranosidase
Control vs DSS 3-day	3.2.1.8	Endo-1,4-beta-xylanase
Control vs DSS 3-day	3.2.2.27	Uracil-DNA glycosylase
Control vs DSS 3-day	3.4.11.18	Methionyl aminopeptidase
Control vs DSS 3-day	3.4.11.9	Xaa-Pro aminopeptidase
Control vs DSS 3-day	3.4.13.18	Cytosol nonspecific dipeptidase
Control vs DSS 3-day	3.4.13.20	Beta-Ala-His dipeptidase
Control vs DSS 3-day	3.4.14.12	Xaa-Xaa-Pro tripeptidyl-peptidase
Control vs DSS 3-day	3.4.16.4	Serine-type D-Ala-D-Ala carboxypeptidase
Control vs DSS 3-day	3.4.21.105	Rhomboid protease
Control vs DSS 3-day	3.4.21.107	Peptidase Do

Control vs DSS 3-day	3.4.21.53	Endopeptidase La
Control vs DSS 3-day	3.4.21.89	Signal peptidase I
Control vs DSS 3-day	3.4.21.92	Endopeptidase Clp
Control vs DSS 3-day	3.4.23.36	Signal peptidase II
Control vs DSS 3-day	3.5.1.100	(R)-amidase
Control vs DSS 3-day	3.5.1.108	UDP-3-O-acyl-N-acetylglucosamine deacetylase
Control vs DSS 3-day	3.5.1.28	N-acetylmuramoyl-L-alanine amidase
Control vs DSS 3-day	3.5.4.10	IMP cyclohydrolase
Control vs DSS 3-day	3.5.4.16	GTP cyclohydrolase I
Control vs DSS 3-day	3.5.4.9	Methenyltetrahydrofolate cyclohydrolase
Control vs DSS 3-day	3.5.99.6	Glucosamine-6-phosphate deaminase
Control vs DSS 3-day	3.6.3.31	Polyamine-transporting ATPase
Control vs DSS 3-day	3.6.3.41	Heme-transporting ATPase
Control vs DSS 3-day	3.6.3.44	Xenobiotic-transporting ATPase
Control vs DSS 3-day	3.6.3.54	Cu(+) exporting ATPase
Control vs DSS 3-day	3.6.4.12	DNA helicase
Control vs DSS 3-day	4.1.1.19	Arginine decarboxylase
Control vs DSS 3-day	4.1.1.20	Diaminopimelate decarboxylase
Control vs DSS 3-day	4.1.1.23	Orotidine-5'-phosphate decarboxylase
Control vs DSS 3-day	4.1.1.36	Phosphopantothenoylcysteine decarboxylase
Control vs DSS 3-day	4.1.1.65	Phosphatidylserine decarboxylase
Control vs DSS 3-day	4.1.2.50	6-carboxytetrahydropterin synthase
Control vs DSS 3-day	4.1.3.36	1,4-dihydroxy-2-naphthoyl-CoA synthase
Control vs DSS 3-day	4.2.1.10	3-dehydroquinate dehydratase
Control vs DSS 3-day	4.2.1.11	Phosphopyruvate hydratase
Control vs DSS 3-day	4.2.1.17	Enoyl-CoA hydratase
Control vs DSS 3-day	4.2.1.2	Fumarate hydratase
Control vs DSS 3-day	4.2.1.3	Aconitate hydratase
Control vs DSS 3-day	4.2.1.33	3-isopropylmalate dehydratase
Control vs DSS 3-day	4.2.1.46	dTDP-glucose 4,6-dehydratase
Control vs DSS 3-day	4.2.1.47	GDP-mannose 4,6-dehydratase
Control vs DSS 3-day	4.2.1.59	3-hydroxyacyl-[acyl-carrier-protein] dehydratase
Control vs DSS 3-day	4.2.1.8	Mannonate dehydratase
Control vs DSS 3-day	4.2.1.9	Dihydroxy-acid dehydratase
Control vs DSS 3-day	4.2.3.4	3-dehydroquinate synthase
Control vs DSS 3-day	4.2.3.5	Chorismate synthase
Control vs DSS 3-day	4.3.1.3	Histidine ammonia-lyase
Control vs DSS 3-day	4.3.2.1	Argininosuccinate lyase
Control vs DSS 3-day	4.3.2.2	Adenylosuccinate lyase
Control vs DSS 3-day	4.3.3.7	4-hydroxy-tetrahydrodipicolinate synthase
Control vs DSS 3-day	5.1.1.1	Alanine racemase
Control vs DSS 3-day	5.1.3.3	Aldose 1-epimerase
Control vs DSS 3-day	5.1.3.4	L-ribulose-5-phosphate 4-epimerase

Control vs DSS 3-day	5.2.1.8	Peptidylprolyl isomerase
Control vs DSS 3-day	5.3.1.1	Triose-phosphate isomerase
Control vs DSS 3-day	5.3.1.12	Glucuronate isomerase
Control vs DSS 3-day	5.3.1.14	L-rhamnose isomerase
Control vs DSS 3-day	5.3.1.17	5-dehydro-4-deoxy-D-glucuronate isomerase
Control vs DSS 3-day	5.3.1.25	L-fucose isomerase
Control vs DSS 3-day	5.3.1.4	L-arabinose isomerase
Control vs DSS 3-day	5.3.1.5	Xylose isomerase
Control vs DSS 3-day	5.3.3.2	Isopentenyl-diphosphate Delta-isomerase
Control vs DSS 3-day	5.4.2.12	Phosphoglycerate mutase (2,3-diphosphoglycerate-independent)
Control vs DSS 3-day	5.4.2.2	Phosphoglucomutase (alpha-D-glucose-1,6-bisphosphate-dependent)
Control vs DSS 3-day	5.99.1.2	DNA topoisomerase
Control vs DSS 3-day	5.99.1.3	DNA topoisomerase (ATP-hydrolyzing)
Control vs DSS 3-day	6.1.1.10	Methionine--tRNA ligase
Control vs DSS 3-day	6.1.1.11	Serine--tRNA ligase
Control vs DSS 3-day	6.1.1.12	Aspartate--tRNA ligase
Control vs DSS 3-day	6.1.1.14	Glycine--tRNA ligase
Control vs DSS 3-day	6.1.1.15	Proline--tRNA ligase
Control vs DSS 3-day	6.1.1.17	Glutamate--tRNA ligase
Control vs DSS 3-day	6.1.1.18	Glutamine--tRNA ligase
Control vs DSS 3-day	6.1.1.19	Arginine--tRNA ligase
Control vs DSS 3-day	6.1.1.2	Tryptophan--tRNA ligase
Control vs DSS 3-day	6.1.1.20	Phenylalanine--tRNA ligase
Control vs DSS 3-day	6.1.1.22	Asparagine--tRNA ligase
Control vs DSS 3-day	6.1.1.3	Threonine--tRNA ligase
Control vs DSS 3-day	6.1.1.4	Leucine--tRNA ligase
Control vs DSS 3-day	6.1.1.5	Isoleucine--tRNA ligase
Control vs DSS 3-day	6.1.1.6	Lysine--tRNA ligase
Control vs DSS 3-day	6.1.1.7	Alanine--tRNA ligase
Control vs DSS 3-day	6.1.1.9	Valine--tRNA ligase
Control vs DSS 3-day	6.2.1.30	Phenylacetate--CoA ligase
Control vs DSS 3-day	6.3.1.1	Aspartate--ammonia ligase
Control vs DSS 3-day	6.3.2.13	UDP-N-acetylmuramoyl-L-alanyl-D-glutamate--2,6-diaminopimelate ligase
Control vs DSS 3-day	6.3.2.4	D-alanine--D-alanine ligase
Control vs DSS 3-day	6.3.2.5	Phosphopantothenate--cysteine ligase
Control vs DSS 3-day	6.3.2.6	Phosphoribosylaminoimidazolesuccinocarboxamide synthase
Control vs DSS 3-day	6.3.2.9	UDP-N-acetylmuramoyl-L-alanine--D-glutamate ligase
Control vs DSS 3-day	6.3.4.13	Phosphoribosylamine--glycine ligase
Control vs DSS 3-day	6.3.4.14	Biotin carboxylase
Control vs DSS 3-day	6.3.4.2	CTP synthase (glutamine hydrolyzing)
Control vs DSS 3-day	6.3.5.2	GMP synthase (glutamine-hydrolyzing)
Control vs DSS 3-day	6.3.5.3	Phosphoribosylformylglycinamidine synthase
Control vs DSS 3-day	6.3.5.5	Carbamoyl-phosphate synthase (glutamine-hydrolyzing)

Control vs DSS 3-day	6.4.1.2	Acetyl-CoA carboxylase
Control vs DSS 3-day	6.5.1.2	DNA ligase (NAD(+))
Control vs DSS 3-day	7.1.2.2	NO_NAME
Control vs DSS 3-day	7.2.1.1	NO_NAME
Control vs DSS 3-day	1.11.1.1	NADH peroxidase
Control vs DSS 3-day	1.2.1.11	Aspartate-semialdehyde dehydrogenase
Control vs DSS 3-day	1.2.1.12	Glyceraldehyde-3-phosphate dehydrogenase (phosphorylating)
Control vs DSS 3-day	2.7.1.90	Diphosphate--fructose-6-phosphate 1-phosphotransferase
Control vs DSS 3-day	2.7.13.3	Histidine kinase
Control vs DSS 3-day	2.7.7.6	DNA-directed RNA polymerase
Control vs DSS 3-day	2.7.7.8	Polyribonucleotide nucleotidyltransferase
Control vs DSS 3-day	5.3.1.1	Triose-phosphate isomerase
Control vs DSS 3-day	5.99.1.3	DNA topoisomerase (ATP-hydrolyzing)
Control vs DSS 3-day	1.1.1.37	Malate dehydrogenase
Control vs DSS 3-day	1.11.1.1	NADH peroxidase
Control vs DSS 3-day	1.3.1.9	Enoyl-[acyl-carrier-protein] reductase (NADH)
Control vs DSS 3-day	2.1.1.72	Site-specific DNA-methyltransferase (adenine-specific)
Control vs DSS 3-day	2.7.1.90	Diphosphate--fructose-6-phosphate 1-phosphotransferase
Control vs DSS 3-day	2.7.13.3	Histidine kinase
Control vs DSS 3-day	2.7.7.6	DNA-directed RNA polymerase
Control vs DSS 3-day	2.7.7.8	Polyribonucleotide nucleotidyltransferase
Control vs DSS 3-day	3.1.21.3	Type I site-specific deoxyribonuclease
Control vs DSS 3-day	3.6.4.12	DNA helicase
Control vs DSS 3-day	4.1.1.49	Phosphoenolpyruvate carboxykinase (ATP)
Control vs DSS 3-day	5.3.1.17	5-dehydro-4-deoxy-D-glucuronate isomerase
Control vs DSS 3-day	5.4.2.12	Phosphoglycerate mutase (2,3-diphosphoglycerate-independent)
Control vs DSS 3-day	5.99.1.3	DNA topoisomerase (ATP-hydrolyzing)
Control vs DSS 3-day	6.1.1.14	Glycine--tRNA ligase
Control vs DSS 3-day	6.3.5.5	Carbamoyl-phosphate synthase (glutamine-hydrolyzing)
Control vs DSS 3-day	1.1.1.205	IMP dehydrogenase
Control vs DSS 3-day	1.1.1.37	Malate dehydrogenase
Control vs DSS 3-day	1.1.1.58	Tagaturonate reductase
Control vs DSS 3-day	1.1.1.86	Ketol-acid reductoisomerase (NADP(+))
Control vs DSS 3-day	1.11.1.1	NADH peroxidase
Control vs DSS 3-day	1.11.1.15	Peroxiredoxin
Control vs DSS 3-day	1.17.4.1	Ribonucleoside-diphosphate reductase
Control vs DSS 3-day	1.17.4.2	Ribonucleoside-triphosphate reductase
Control vs DSS 3-day	1.2.7.8	Indolepyruvate ferredoxin oxidoreductase
Control vs DSS 3-day	1.3.1.14	Dihydroorotate dehydrogenase (NAD(+))
Control vs DSS 3-day	1.4.1.16	Diaminopimelate dehydrogenase
Control vs DSS 3-day	1.4.1.2	Glutamate dehydrogenase
Control vs DSS 3-day	1.4.3.16	L-aspartate oxidase
Control vs DSS 3-day	1.5.1.5	Methylenetetrahydrofolate dehydrogenase (NADP(+))

Control vs DSS 3-day	1.8.1.9	Thioredoxin-disulfide reductase
Control vs DSS 3-day	2.1.1.182	16S rRNA (adenine(1518)-N(6)/adenine(1519)-N(6))-dimethyltransferase
Control vs DSS 3-day	2.1.1.192	23S rRNA (adenine(2503)-C(2))-methyltransferase
Control vs DSS 3-day	2.1.1.37	DNA (cytosine-5-)-methyltransferase
Control vs DSS 3-day	2.1.1.72	Site-specific DNA-methyltransferase (adenine-specific)
Control vs DSS 3-day	2.1.2.1	Glycine hydroxymethyltransferase
Control vs DSS 3-day	2.1.2.11	3-methyl-2-oxobutanoate hydroxymethyltransferase
Control vs DSS 3-day	2.1.2.3	Phosphoribosylaminoimidazolecarboxamide formyltransferase
Control vs DSS 3-day	2.1.3.2	Aspartate carbamoyltransferase
Control vs DSS 3-day	2.3.1.179	Beta-ketoacyl-[acyl-carrier-protein] synthase II
Control vs DSS 3-day	2.3.1.180	Beta-ketoacyl-[acyl-carrier-protein] synthase III
Control vs DSS 3-day	2.3.1.234	N(6)-L-threonylcarbamoyladenine synthase
Control vs DSS 3-day	2.3.3.13	2-isopropylmalate synthase
Control vs DSS 3-day	2.4.1.18	1,4-alpha-glucan branching enzyme
Control vs DSS 3-day	2.4.1.21	Starch synthase
Control vs DSS 3-day	2.4.1.320	1,4-beta-mannosyl-N-acetylglucosamine phosphorylase
Control vs DSS 3-day	2.4.2.29	tRNA-guanine(34) transglycosylase
Control vs DSS 3-day	2.5.1.7	UDP-N-acetylglucosamine 1-carboxyvinyltransferase
Control vs DSS 3-day	2.5.1.78	6,7-dimethyl-8-ribityllumazine synthase
Control vs DSS 3-day	2.6.1.16	Glutamine--fructose-6-phosphate transaminase (isomerizing)
Control vs DSS 3-day	2.6.1.52	Phosphoserine transaminase
Control vs DSS 3-day	2.7.1.40	Pyruvate kinase
Control vs DSS 3-day	2.7.1.90	Diphosphate--fructose-6-phosphate 1-phosphotransferase
Control vs DSS 3-day	2.7.13.3	Histidine kinase
Control vs DSS 3-day	2.7.2.1	Acetate kinase
Control vs DSS 3-day	2.7.2.3	Phosphoglycerate kinase
Control vs DSS 3-day	2.7.6.1	Ribose-phosphate diphosphokinase
Control vs DSS 3-day	2.7.7.33	Glucose-1-phosphate cytidylyltransferase
Control vs DSS 3-day	2.7.7.7	DNA-directed DNA polymerase
Control vs DSS 3-day	2.7.7.71	D-glycero-alpha-D-manno-heptose 1-phosphate guanylyltransferase
Control vs DSS 3-day	2.7.7.8	Polyribonucleotide nucleotidyltransferase
Control vs DSS 3-day	2.7.8.13	Phospho-N-acetylmuramoyl-pentapeptide-transferase
Control vs DSS 3-day	2.8.4.3	tRNA-2-methylthio-N(6)-dimethylallyladenosine synthase
Control vs DSS 3-day	3.2.1.1	Alpha-amylase
Control vs DSS 3-day	3.2.1.14	Chitinase
Control vs DSS 3-day	3.4.11.18	Methionyl aminopeptidase
Control vs DSS 3-day	3.4.11.9	Xaa-Pro aminopeptidase
Control vs DSS 3-day	3.4.13.18	Cytosol nonspecific dipeptidase
Control vs DSS 3-day	3.4.21.53	Endopeptidase La
Control vs DSS 3-day	3.4.21.92	Endopeptidase Clp
Control vs DSS 3-day	3.5.1.108	UDP-3-O-acyl-N-acetylglucosamine deacetylase
Control vs DSS 3-day	3.5.4.10	IMP cyclohydrolase
Control vs DSS 3-day	3.5.4.16	GTP cyclohydrolase I

Control vs DSS 3-day	3.5.4.9	Methenyltetrahydrofolate cyclohydrolase
Control vs DSS 3-day	3.6.3.31	Polyamine-transporting ATPase
Control vs DSS 3-day	3.6.4.12	DNA helicase
Control vs DSS 3-day	4.1.1.15	Glutamate decarboxylase
Control vs DSS 3-day	4.1.1.19	Arginine decarboxylase
Control vs DSS 3-day	4.1.2.4	Deoxyribose-phosphate aldolase
Control vs DSS 3-day	4.2.1.59	3-hydroxyacyl-[acyl-carrier-protein] dehydratase
Control vs DSS 3-day	4.3.1.17	L-serine ammonia-lyase
Control vs DSS 3-day	5.2.1.8	Peptidylprolyl isomerase
Control vs DSS 3-day	5.3.1.1	Triose-phosphate isomerase
Control vs DSS 3-day	5.3.1.12	Glucuronate isomerase
Control vs DSS 3-day	5.3.1.17	5-dehydro-4-deoxy-D-glucuronate isomerase
Control vs DSS 3-day	5.3.1.25	L-fucose isomerase
Control vs DSS 3-day	5.3.1.4	L-arabinose isomerase
Control vs DSS 3-day	5.4.2.12	Phosphoglycerate mutase (2,3-diphosphoglycerate-independent)
Control vs DSS 3-day	5.4.2.2	Phosphoglucomutase (alpha-D-glucose-1,6-bisphosphate-dependent)
Control vs DSS 3-day	5.99.1.3	DNA topoisomerase (ATP-hydrolyzing)
Control vs DSS 3-day	6.1.1.10	Methionine--tRNA ligase
Control vs DSS 3-day	6.1.1.11	Serine--tRNA ligase
Control vs DSS 3-day	6.1.1.12	Aspartate--tRNA ligase
Control vs DSS 3-day	6.1.1.14	Glycine--tRNA ligase
Control vs DSS 3-day	6.1.1.16	Cysteine--tRNA ligase
Control vs DSS 3-day	6.1.1.18	Glutamine--tRNA ligase
Control vs DSS 3-day	6.1.1.2	Tryptophan--tRNA ligase
Control vs DSS 3-day	6.1.1.20	Phenylalanine--tRNA ligase
Control vs DSS 3-day	6.1.1.22	Asparagine--tRNA ligase
Control vs DSS 3-day	6.1.1.6	Lysine--tRNA ligase
Control vs DSS 3-day	6.1.1.7	Alanine--tRNA ligase
Control vs DSS 3-day	6.3.2.6	Phosphoribosylaminoimidazolesuccinocarboxamide synthase
Control vs DSS 3-day	6.3.4.13	Phosphoribosylamine--glycine ligase
Control vs DSS 3-day	6.3.5.3	Phosphoribosylformylglycinamidine synthase
Control vs DSS 3-day	6.3.5.5	Carbamoyl-phosphate synthase (glutamine-hydrolyzing)
Control vs DSS 3-day	6.5.1.2	DNA ligase (NAD(+))
Control vs DSS 3-day	7.1.2.2	NO_NAME
Control vs DSS 3-day	7.2.1.1	NO_NAME
Control vs DSS 3-day	1.1.1.37	Malate dehydrogenase
Control vs DSS 3-day	1.1.1.86	Ketol-acid reductoisomerase (NADP(+))
Control vs DSS 3-day	1.1.1.95	Phosphoglycerate dehydrogenase
Control vs DSS 3-day	1.11.1.1	NADH peroxidase
Control vs DSS 3-day	1.11.1.15	Peroxiredoxin
Control vs DSS 3-day	1.15.1.1	Superoxide dismutase
Control vs DSS 3-day	1.16.3.2	Bacterial non-heme ferritin
Control vs DSS 3-day	1.4.1.2	Glutamate dehydrogenase

Control vs DSS 3-day	1.4.1.3	Glutamate dehydrogenase (NAD(P)(+))
Control vs DSS 3-day	2.1.1.72	Site-specific DNA-methyltransferase (adenine-specific)
Control vs DSS 3-day	2.1.2.1	Glycine hydroxymethyltransferase
Control vs DSS 3-day	2.1.2.3	Phosphoribosylaminoimidazolecarboxamide formyltransferase
Control vs DSS 3-day	2.3.1.179	Beta-ketoacyl-[acyl-carrier-protein] synthase II
Control vs DSS 3-day	2.3.1.180	Beta-ketoacyl-[acyl-carrier-protein] synthase III
Control vs DSS 3-day	2.3.1.51	1-acylglycerol-3-phosphate O-acyltransferase
Control vs DSS 3-day	2.4.2.10	Orotate phosphoribosyltransferase
Control vs DSS 3-day	2.6.1.16	Glutamine--fructose-6-phosphate transaminase (isomerizing)
Control vs DSS 3-day	2.6.1.52	Phosphoserine transaminase
Control vs DSS 3-day	2.7.1.11	6-phosphofructokinase
Control vs DSS 3-day	2.7.13.3	Histidine kinase
Control vs DSS 3-day	2.7.2.3	Phosphoglycerate kinase
Control vs DSS 3-day	2.7.6.1	Ribose-phosphate diphosphokinase
Control vs DSS 3-day	2.7.7.49	RNA-directed DNA polymerase
Control vs DSS 3-day	2.7.7.6	DNA-directed RNA polymerase
Control vs DSS 3-day	2.7.7.8	Polyribonucleotide nucleotidyltransferase
Control vs DSS 3-day	2.7.8.13	Phospho-N-acetylmuramoyl-pentapeptide-transferase
Control vs DSS 3-day	3.1.21.3	Type I site-specific deoxyribonuclease
Control vs DSS 3-day	3.1.3.25	Inositol-phosphate phosphatase
Control vs DSS 3-day	3.4.11.18	Methionyl aminopeptidase
Control vs DSS 3-day	3.5.4.10	IMP cyclohydrolase
Control vs DSS 3-day	3.5.99.6	Glucosamine-6-phosphate deaminase
Control vs DSS 3-day	3.6.3.44	Xenobiotic-transporting ATPase
Control vs DSS 3-day	3.6.4.12	DNA helicase
Control vs DSS 3-day	4.1.1.23	Orotidine-5'-phosphate decarboxylase
Control vs DSS 3-day	4.1.1.49	Phosphoenolpyruvate carboxykinase (ATP)
Control vs DSS 3-day	4.2.1.11	Phosphopyruvate hydratase
Control vs DSS 3-day	4.2.1.2	Fumarate hydratase
Control vs DSS 3-day	5.1.3.3	Aldose 1-epimerase
Control vs DSS 3-day	5.2.1.8	Peptidylprolyl isomerase
Control vs DSS 3-day	5.3.1.5	Xylose isomerase
Control vs DSS 3-day	5.99.1.3	DNA topoisomerase (ATP-hydrolyzing)
Control vs DSS 3-day	6.1.1.12	Aspartate--tRNA ligase
Control vs DSS 3-day	6.1.1.14	Glycine--tRNA ligase
Control vs DSS 3-day	6.1.1.22	Asparagine--tRNA ligase
Control vs DSS 3-day	6.3.4.2	CTP synthase (glutamine hydrolyzing)
Control vs DSS 3-day	6.3.4.4	Adenylosuccinate synthase
Control vs DSS 3-day	6.3.5.5	Carbamoyl-phosphate synthase (glutamine-hydrolyzing)
Control vs DSS 3-day	7.1.2.2	NO_NAME
Control vs DSS 3-day	7.2.1.1	NO_NAME
Control vs DSS 3-day	1.1.1.100	3-oxoacyl-[acyl-carrier-protein] reductase
Control vs DSS 3-day	1.1.1.122	D-threo-aldose 1-dehydrogenase

Control vs DSS 3-day	1.1.1.133	dTDP-4-dehydrorhamnose reductase
Control vs DSS 3-day	1.1.1.169	2-dehydropantoate 2-reductase
Control vs DSS 3-day	1.1.1.205	IMP dehydrogenase
Control vs DSS 3-day	1.1.1.22	UDP-glucose 6-dehydrogenase
Control vs DSS 3-day	1.1.1.262	4-hydroxythreonine-4-phosphate dehydrogenase
Control vs DSS 3-day	1.1.1.271	GDP-L-fucose synthase
Control vs DSS 3-day	1.1.1.290	4-phosphoerythronate dehydrogenase
Control vs DSS 3-day	1.1.1.37	Malate dehydrogenase
Control vs DSS 3-day	1.1.1.40	Malate dehydrogenase (oxaloacetate-decarboxylating) (NADP(+))
Control vs DSS 3-day	1.1.1.42	Isocitrate dehydrogenase (NADP(+))
Control vs DSS 3-day	1.1.1.44	Phosphogluconate dehydrogenase (NADP(+)-dependent, decarboxylating)
Control vs DSS 3-day	1.1.1.49	Glucose-6-phosphate dehydrogenase (NADP(+))
Control vs DSS 3-day	1.1.1.58	Tagaturonate reductase
Control vs DSS 3-day	1.1.1.85	3-isopropylmalate dehydrogenase
Control vs DSS 3-day	1.1.1.86	Ketol-acid reductoisomerase (NADP(+))
Control vs DSS 3-day	1.1.1.94	Glycerol-3-phosphate dehydrogenase (NAD(P)(+))
Control vs DSS 3-day	1.1.1.95	Phosphoglycerate dehydrogenase
Control vs DSS 3-day	1.11.1.1	NADH peroxidase
Control vs DSS 3-day	1.11.1.15	Peroxiredoxin
Control vs DSS 3-day	1.15.1.1	Superoxide dismutase
Control vs DSS 3-day	1.16.3.2	Bacterial non-heme ferritin
Control vs DSS 3-day	1.17.4.1	Ribonucleoside-diphosphate reductase
Control vs DSS 3-day	1.17.4.2	Ribonucleoside-triphosphate reductase
Control vs DSS 3-day	1.17.7.3	(E)-4-hydroxy-3-methylbut-2-enyl-diphosphate synthase (flavodoxin)
Control vs DSS 3-day	1.2.1.11	Aspartate-semialdehyde dehydrogenase
Control vs DSS 3-day	1.2.1.38	N-acetyl-gamma-glutamyl-phosphate reductase
Control vs DSS 3-day	1.2.1.41	Glutamate-5-semialdehyde dehydrogenase
Control vs DSS 3-day	1.2.7.8	Indolepyruvate ferredoxin oxidoreductase
Control vs DSS 3-day	1.3.1.1	Dihydrouracil dehydrogenase (NAD(+))
Control vs DSS 3-day	1.3.1.14	Dihydroorotate dehydrogenase (NAD(+))
Control vs DSS 3-day	1.3.1.26	Transferred entry
Control vs DSS 3-day	1.3.1.9	Enoyl-[acyl-carrier-protein] reductase (NADH)
Control vs DSS 3-day	1.3.98.1	Dihydroorotate oxidase (fumarate)
Control vs DSS 3-day	1.3.99.5	3-oxo-5-alpha-steroid 4-dehydrogenase (acceptor)
Control vs DSS 3-day	1.4.1.13	Glutamate synthase (NADPH)
Control vs DSS 3-day	1.4.1.16	Diaminopimelate dehydrogenase
Control vs DSS 3-day	1.4.1.2	Glutamate dehydrogenase
Control vs DSS 3-day	1.4.1.21	Aspartate dehydrogenase
Control vs DSS 3-day	1.4.1.3	Glutamate dehydrogenase (NAD(P)(+))
Control vs DSS 3-day	1.4.3.16	L-aspartate oxidase
Control vs DSS 3-day	1.4.3.5	Pyridoxal 5'-phosphate synthase
Control vs DSS 3-day	1.4.4.2	Glycine dehydrogenase (aminomethyl-transferring)
Control vs DSS 3-day	1.5.1.2	Pyrroline-5-carboxylate reductase

Control vs DSS 3-day	1.5.1.20	Methylenetetrahydrofolate reductase (NAD(P)H)
Control vs DSS 3-day	1.5.1.3	Dihydrofolate reductase
Control vs DSS 3-day	1.5.1.39	FMN reductase (NAD(P)H)
Control vs DSS 3-day	1.5.1.5	Methylenetetrahydrofolate dehydrogenase (NADP(+))
Control vs DSS 3-day	1.6.5.11	NADH dehydrogenase (quinone)
Control vs DSS 3-day	1.6.5.3	NADH
Control vs DSS 3-day	1.6.99.1	NADPH dehydrogenase
Control vs DSS 3-day	1.7.1.13	PreQ(1) synthase
Control vs DSS 3-day	1.8.1.4	Dihydrolipoyl dehydrogenase
Control vs DSS 3-day	1.8.1.9	Thioredoxin-disulfide reductase
Control vs DSS 3-day	1.97.1.4	[Formate-C-acetyltransferase]-activating enzyme
Control vs DSS 3-day	2.1.1.13	Methionine synthase
Control vs DSS 3-day	2.1.1.163	Demethylmenaquinone methyltransferase
Control vs DSS 3-day	2.1.1.182	16S rRNA (adenine(1518)-N(6)/adenine(1519)-N(6))-dimethyltransferase
Control vs DSS 3-day	2.1.1.191	23S rRNA (cytosine(1962)-C(5))-methyltransferase
Control vs DSS 3-day	2.1.1.192	23S rRNA (adenine(2503)-C(2))-methyltransferase
Control vs DSS 3-day	2.1.1.193	16S rRNA (uracil(1498)-N(3))-methyltransferase
Control vs DSS 3-day	2.1.1.228	tRNA (guanine(37)-N(1))-methyltransferase
Control vs DSS 3-day	2.1.1.33	tRNA (guanine(46)-N(7))-methyltransferase
Control vs DSS 3-day	2.1.1.45	Thymidylate synthase
Control vs DSS 3-day	2.1.1.63	Methylated-DNA--[protein]-cysteine S-methyltransferase
Control vs DSS 3-day	2.1.1.72	Site-specific DNA-methyltransferase (adenine-specific)
Control vs DSS 3-day	2.1.2.1	Glycine hydroxymethyltransferase
Control vs DSS 3-day	2.1.2.3	Phosphoribosylaminoimidazolecarboxamide formyltransferase
Control vs DSS 3-day	2.1.2.9	Methionyl-tRNA formyltransferase
Control vs DSS 3-day	2.1.3.2	Aspartate carbamoyltransferase
Control vs DSS 3-day	2.1.3.9	N-acetylornithine carbamoyltransferase
Control vs DSS 3-day	2.2.1.1	Transketolase
Control vs DSS 3-day	2.2.1.6	Acetolactate synthase
Control vs DSS 3-day	2.2.1.7	1-deoxy-D-xylulose-5-phosphate synthase
Control vs DSS 3-day	2.2.1.9	synthase
Control vs DSS 3-day	2.3.1.129	Acyl-[acyl-carrier-protein]--UDP-N-acetylglucosamine O-acyltransferase
Control vs DSS 3-day	2.3.1.179	Beta-ketoacyl-[acyl-carrier-protein] synthase II
Control vs DSS 3-day	2.3.1.180	Beta-ketoacyl-[acyl-carrier-protein] synthase III
Control vs DSS 3-day	2.3.1.234	N(6)-L-threonylcarbamoyladenine synthase
Control vs DSS 3-day	2.3.1.29	Glycine C-acetyltransferase
Control vs DSS 3-day	2.3.1.31	Homoserine O-acetyltransferase
Control vs DSS 3-day	2.3.1.39	[Acyl-carrier-protein] S-malonyltransferase
Control vs DSS 3-day	2.3.1.47	8-amino-7-oxononanoate synthase
Control vs DSS 3-day	2.3.1.51	1-acylglycerol-3-phosphate O-acyltransferase
Control vs DSS 3-day	2.3.3.13	2-isopropylmalate synthase
Control vs DSS 3-day	2.4.1.18	1,4-alpha-glucan branching enzyme
Control vs DSS 3-day	2.4.1.21	Starch synthase

Control vs DSS 3-day	2.4.1.212	Hyaluronan synthase
Control vs DSS 3-day	2.4.1.320	1,4-beta-mannosyl-N-acetylglucosamine phosphorylase
Control vs DSS 3-day	2.4.2.1	Purine-nucleoside phosphorylase
Control vs DSS 3-day	2.4.2.10	Orotate phosphoribosyltransferase
Control vs DSS 3-day	2.4.2.14	Amidophosphoribosyltransferase
Control vs DSS 3-day	2.4.2.17	ATP phosphoribosyltransferase
Control vs DSS 3-day	2.4.2.19	Nicotinate-nucleotide diphosphorylase (carboxylating)
Control vs DSS 3-day	2.4.2.29	tRNA-guanine(34) transglycosylase
Control vs DSS 3-day	2.4.2.8	Hypoxanthine phosphoribosyltransferase
Control vs DSS 3-day	2.4.99.17	S-adenosylmethionine
Control vs DSS 3-day	2.5.1.15	Dihydropteroate synthase
Control vs DSS 3-day	2.5.1.17	Cob(I)yrinic acid a,c-diamide adenosyltransferase
Control vs DSS 3-day	2.5.1.19	3-phosphoshikimate 1-carboxyvinyltransferase
Control vs DSS 3-day	2.5.1.47	Cysteine synthase
Control vs DSS 3-day	2.5.1.6	Methionine adenosyltransferase
Control vs DSS 3-day	2.5.1.7	UDP-N-acetylglucosamine 1-carboxyvinyltransferase
Control vs DSS 3-day	2.5.1.72	Quinolinate synthase
Control vs DSS 3-day	2.5.1.75	tRNA dimethylallyltransferase
Control vs DSS 3-day	2.5.1.78	6,7-dimethyl-8-ribityllumazine synthase
Control vs DSS 3-day	2.6.1.1	Aspartate transaminase
Control vs DSS 3-day	2.6.1.16	Glutamine--fructose-6-phosphate transaminase (isomerizing)
Control vs DSS 3-day	2.6.1.42	Branched-chain-amino-acid transaminase
Control vs DSS 3-day	2.6.1.52	Phosphoserine transaminase
Control vs DSS 3-day	2.6.1.83	LL-diaminopimelate aminotransferase
Control vs DSS 3-day	2.6.1.9	Histidinol-phosphate transaminase
Control vs DSS 3-day	2.7.1.11	6-phosphofructokinase
Control vs DSS 3-day	2.7.1.130	Tetraacyldisaccharide 4'-kinase
Control vs DSS 3-day	2.7.1.15	Ribokinase
Control vs DSS 3-day	2.7.1.180	FAD
Control vs DSS 3-day	2.7.1.26	Riboflavin kinase
Control vs DSS 3-day	2.7.1.33	Pantothenate kinase
Control vs DSS 3-day	2.7.1.35	Pyridoxal kinase
Control vs DSS 3-day	2.7.1.40	Pyruvate kinase
Control vs DSS 3-day	2.7.1.48	Uridine kinase
Control vs DSS 3-day	2.7.1.5	Rhamnulokinase
Control vs DSS 3-day	2.7.1.69	Protein-N(pi)-phosphohistidine--sugar phosphotransferase
Control vs DSS 3-day	2.7.1.90	Diphosphate--fructose-6-phosphate 1-phosphotransferase
Control vs DSS 3-day	2.7.13.3	Histidine kinase
Control vs DSS 3-day	2.7.2.1	Acetate kinase
Control vs DSS 3-day	2.7.2.11	Glutamate 5-kinase
Control vs DSS 3-day	2.7.2.3	Phosphoglycerate kinase
Control vs DSS 3-day	2.7.2.4	Aspartate kinase
Control vs DSS 3-day	2.7.4.16	Thiamine-phosphate kinase

Control vs DSS 3-day	2.7.4.25	(d)CMP kinase
Control vs DSS 3-day	2.7.4.3	Adenylate kinase
Control vs DSS 3-day	2.7.4.6	Nucleoside-diphosphate kinase
Control vs DSS 3-day	2.7.6.1	Ribose-phosphate diphosphokinase
Control vs DSS 3-day	2.7.7.13	Mannose-1-phosphate guanylyltransferase
Control vs DSS 3-day	2.7.7.18	Nicotinate-nucleotide adenylyltransferase
Control vs DSS 3-day	2.7.7.2	FAD synthetase
Control vs DSS 3-day	2.7.7.24	Glucose-1-phosphate thymidylyltransferase
Control vs DSS 3-day	2.7.7.3	Pantetheine-phosphate adenylyltransferase
Control vs DSS 3-day	2.7.7.4	Sulfate adenylyltransferase
Control vs DSS 3-day	2.7.7.7	DNA-directed DNA polymerase
Control vs DSS 3-day	2.7.7.72	CCA tRNA nucleotidyltransferase
Control vs DSS 3-day	2.7.7.8	Polyribonucleotide nucleotidyltransferase
Control vs DSS 3-day	2.7.7.87	L-threonylcarbamoyladenylate synthase
Control vs DSS 3-day	2.7.8.8	CDP-diacylglycerol--serine O-phosphatidyltransferase
Control vs DSS 3-day	2.7.9.1	Pyruvate, phosphate dikinase
Control vs DSS 3-day	2.8.1.7	Cysteine desulfurase
Control vs DSS 3-day	2.8.4.3	tRNA-2-methylthio-N(6)-dimethylallyladenosine synthase
Control vs DSS 3-day	2.8.4.4	[Ribosomal protein S12] (aspartate(89)-C(3))-methylthiotransferase
Control vs DSS 3-day	3.1.1.11	Pectinesterase
Control vs DSS 3-day	3.1.1.29	Aminoacyl-tRNA hydrolase
Control vs DSS 3-day	3.1.11.2	Exodeoxyribonuclease III
Control vs DSS 3-day	3.1.11.6	Exodeoxyribonuclease VII
Control vs DSS 3-day	3.1.13.1	Exoribonuclease II
Control vs DSS 3-day	3.1.21.2	Deoxyribonuclease IV
Control vs DSS 3-day	3.1.21.3	Type I site-specific deoxyribonuclease
Control vs DSS 3-day	3.1.22.4	Crossover junction endodeoxyribonuclease
Control vs DSS 3-day	3.1.3.11	Fructose-bisphosphatase
Control vs DSS 3-day	3.1.3.15	Histidinol-phosphatase
Control vs DSS 3-day	3.1.3.25	Inositol-phosphate phosphatase
Control vs DSS 3-day	3.1.3.45	3-deoxy-manno-octulosonate-8-phosphatase
Control vs DSS 3-day	3.1.3.5	5'-nucleotidase
Control vs DSS 3-day	3.1.4.46	Glycerophosphodiester phosphodiesterase
Control vs DSS 3-day	3.1.6.1	Arylsulfatase
Control vs DSS 3-day	3.2.1.131	Xylan alpha-1,2-glucuronosidase
Control vs DSS 3-day	3.2.1.135	Neopullulanase
Control vs DSS 3-day	3.2.1.165	Exo-1,4-beta-D-glucosaminidase
Control vs DSS 3-day	3.2.1.172	Unsaturated rhamnogalacturonyl hydrolase
Control vs DSS 3-day	3.2.1.177	Alpha-D-xyloside xylohydrolase
Control vs DSS 3-day	3.2.1.21	Beta-glucosidase
Control vs DSS 3-day	3.2.1.22	Alpha-galactosidase
Control vs DSS 3-day	3.2.1.23	Beta-galactosidase
Control vs DSS 3-day	3.2.1.25	Beta-mannosidase

Control vs DSS 3-day	3.2.1.31	Beta-glucuronidase
Control vs DSS 3-day	3.2.1.37	Xylan 1,4-beta-xylosidase
Control vs DSS 3-day	3.2.1.51	Alpha-L-fucosidase
Control vs DSS 3-day	3.2.1.52	Beta-N-acetylhexosaminidase
Control vs DSS 3-day	3.2.1.55	Non-reducing end alpha-L-arabinofuranosidase
Control vs DSS 3-day	3.2.1.8	Endo-1,4-beta-xylanase
Control vs DSS 3-day	3.2.1.82	Exo-poly-alpha-galacturonosidase
Control vs DSS 3-day	3.2.2.27	Uracil-DNA glycosylase
Control vs DSS 3-day	3.2.2.n1	Cytokinin riboside 5'-monophosphate phosphoribohydrolase
Control vs DSS 3-day	3.4.11.18	Methionyl aminopeptidase
Control vs DSS 3-day	3.4.11.4	Tripeptide aminopeptidase
Control vs DSS 3-day	3.4.13.18	Cytosol nonspecific dipeptidase
Control vs DSS 3-day	3.4.13.20	Beta-Ala-His dipeptidase
Control vs DSS 3-day	3.4.14.12	Xaa-Xaa-Pro tripeptidyl-peptidase
Control vs DSS 3-day	3.4.14.4	Dipeptidyl-peptidase III
Control vs DSS 3-day	3.4.15.5	Peptidyl-dipeptidase Dcp
Control vs DSS 3-day	3.4.16.4	Serine-type D-Ala-D-Ala carboxypeptidase
Control vs DSS 3-day	3.4.21.102	C-terminal processing peptidase
Control vs DSS 3-day	3.4.21.105	Rhomboid protease
Control vs DSS 3-day	3.4.21.107	Peptidase Do
Control vs DSS 3-day	3.4.21.53	Endopeptidase La
Control vs DSS 3-day	3.4.21.89	Signal peptidase I
Control vs DSS 3-day	3.4.21.92	Endopeptidase Clp
Control vs DSS 3-day	3.4.23.36	Signal peptidase II
Control vs DSS 3-day	3.4.24.55	Pitrilysin
Control vs DSS 3-day	3.5.1.10	Formyltetrahydrofolate deformylase
Control vs DSS 3-day	3.5.1.100	(R)-amidase
Control vs DSS 3-day	3.5.1.104	Peptidoglycan-N-acetylglucosamine deacetylase
Control vs DSS 3-day	3.5.1.108	UDP-3-O-acyl-N-acetylglucosamine deacetylase
Control vs DSS 3-day	3.5.1.2	Glutaminase
Control vs DSS 3-day	3.5.1.25	N-acetylglucosamine-6-phosphate deacetylase
Control vs DSS 3-day	3.5.1.28	N-acetylmuramoyl-L-alanine amidase
Control vs DSS 3-day	3.5.1.88	Peptide deformylase
Control vs DSS 3-day	3.5.2.3	Dihydroorotase
Control vs DSS 3-day	3.5.2.7	Imidazolonepropionase
Control vs DSS 3-day	3.5.4.10	IMP cyclohydrolase
Control vs DSS 3-day	3.5.4.16	GTP cyclohydrolase I
Control vs DSS 3-day	3.5.4.19	Phosphoribosyl-AMP cyclohydrolase
Control vs DSS 3-day	3.5.4.25	GTP cyclohydrolase II
Control vs DSS 3-day	3.5.4.33	tRNA(adenine(34)) deaminase
Control vs DSS 3-day	3.5.4.9	Methenyltetrahydrofolate cyclohydrolase
Control vs DSS 3-day	3.5.99.6	Glucosamine-6-phosphate deaminase
Control vs DSS 3-day	3.6.1.27	Undecaprenyl-diphosphate phosphatase

Control vs DSS 3-day	3.6.1.31	Phosphoribosyl-ATP diphosphatase
Control vs DSS 3-day	3.6.1.66	XTP/dITP diphosphatase
Control vs DSS 3-day	3.6.3.31	Polyamine-transporting ATPase
Control vs DSS 3-day	3.6.3.41	Heme-transporting ATPase
Control vs DSS 3-day	3.6.3.44	Xenobiotic-transporting ATPase
Control vs DSS 3-day	3.6.3.54	Cu(+) exporting ATPase
Control vs DSS 3-day	3.6.4.12	DNA helicase
Control vs DSS 3-day	3.6.5.n1	Elongation factor 4
Control vs DSS 3-day	4.1.1.12	Aspartate 4-decarboxylase
Control vs DSS 3-day	4.1.1.15	Glutamate decarboxylase
Control vs DSS 3-day	4.1.1.19	Arginine decarboxylase
Control vs DSS 3-day	4.1.1.20	Diaminopimelate decarboxylase
Control vs DSS 3-day	4.1.1.23	Orotidine-5'-phosphate decarboxylase
Control vs DSS 3-day	4.1.1.65	Phosphatidylserine decarboxylase
Control vs DSS 3-day	4.1.2.19	Rhamnulose-1-phosphate aldolase
Control vs DSS 3-day	4.1.2.4	Deoxyribose-phosphate aldolase
Control vs DSS 3-day	4.1.2.50	6-carboxytetrahydropterin synthase
Control vs DSS 3-day	4.1.3.36	1,4-dihydroxy-2-naphthoyl-CoA synthase
Control vs DSS 3-day	4.1.99.12	3,4-dihydroxy-2-butanone-4-phosphate synthase
Control vs DSS 3-day	4.2.1.11	Phosphopyruvate hydratase
Control vs DSS 3-day	4.2.1.126	N-acetylmuramic acid 6-phosphate etherase
Control vs DSS 3-day	4.2.1.17	Enoyl-CoA hydratase
Control vs DSS 3-day	4.2.1.19	Imidazoleglycerol-phosphate dehydratase
Control vs DSS 3-day	4.2.1.2	Fumarate hydratase
Control vs DSS 3-day	4.2.1.20	Tryptophan synthase
Control vs DSS 3-day	4.2.1.3	Aconitate hydratase
Control vs DSS 3-day	4.2.1.33	3-isopropylmalate dehydratase
Control vs DSS 3-day	4.2.1.46	dTDP-glucose 4,6-dehydratase
Control vs DSS 3-day	4.2.1.47	GDP-mannose 4,6-dehydratase
Control vs DSS 3-day	4.2.1.59	3-hydroxyacyl-[acyl-carrier-protein] dehydratase
Control vs DSS 3-day	4.2.1.8	Mannonate dehydratase
Control vs DSS 3-day	4.2.1.9	Dihydroxy-acid dehydratase
Control vs DSS 3-day	4.2.3.1	Threonine synthase
Control vs DSS 3-day	4.2.3.4	3-dehydroquinate synthase
Control vs DSS 3-day	4.2.3.5	Chorismate synthase
Control vs DSS 3-day	4.3.1.17	L-serine ammonia-lyase
Control vs DSS 3-day	4.3.1.3	Histidine ammonia-lyase
Control vs DSS 3-day	4.3.1.4	Formimidoyltetrahydrofolate cyclodeaminase
Control vs DSS 3-day	4.3.2.1	Argininosuccinate lyase
Control vs DSS 3-day	4.3.2.10	NO_NAME
Control vs DSS 3-day	4.3.2.2	Adenylosuccinate lyase
Control vs DSS 3-day	4.3.3.7	4-hydroxy-tetrahydrodipicolinate synthase
Control vs DSS 3-day	5.1.1.1	Alanine racemase

Control vs DSS 3-day	5.1.1.7	Diaminopimelate epimerase
Control vs DSS 3-day	5.1.3.1	Ribulose-phosphate 3-epimerase
Control vs DSS 3-day	5.1.3.3	Aldose 1-epimerase
Control vs DSS 3-day	5.1.3.4	L-ribulose-5-phosphate 4-epimerase
Control vs DSS 3-day	5.2.1.8	Peptidylprolyl isomerase
Control vs DSS 3-day	5.3.1.1	Triose-phosphate isomerase
Control vs DSS 3-day	5.3.1.12	Glucuronate isomerase
Control vs DSS 3-day	5.3.1.14	L-rhamnose isomerase
Control vs DSS 3-day	5.3.1.16	isomerase
Control vs DSS 3-day	5.3.1.17	5-dehydro-4-deoxy-D-glucuronate isomerase
Control vs DSS 3-day	5.3.1.25	L-fucose isomerase
Control vs DSS 3-day	5.3.1.4	L-arabinose isomerase
Control vs DSS 3-day	5.3.1.5	Xylose isomerase
Control vs DSS 3-day	5.3.1.9	Glucose-6-phosphate isomerase
Control vs DSS 3-day	5.3.3.2	Isopentenyl-diphosphate Delta-isomerase
Control vs DSS 3-day	5.4.2.10	Phosphoglucosamine mutase
Control vs DSS 3-day	5.4.2.12	Phosphoglycerate mutase (2,3-diphosphoglycerate-independent)
Control vs DSS 3-day	5.4.2.2	Phosphoglucomutase (alpha-D-glucose-1,6-bisphosphate-dependent)
Control vs DSS 3-day	5.4.2.8	Phosphomannomutase
Control vs DSS 3-day	5.4.99.18	5-(carboxyamino)imidazole ribonucleotide mutase
Control vs DSS 3-day	5.4.99.25	tRNA pseudouridine(55) synthase
Control vs DSS 3-day	5.99.1.2	DNA topoisomerase
Control vs DSS 3-day	5.99.1.3	DNA topoisomerase (ATP-hydrolyzing)
Control vs DSS 3-day	6.1.1.1	Tyrosine--tRNA ligase
Control vs DSS 3-day	6.1.1.10	Methionine--tRNA ligase
Control vs DSS 3-day	6.1.1.11	Serine--tRNA ligase
Control vs DSS 3-day	6.1.1.12	Aspartate--tRNA ligase
Control vs DSS 3-day	6.1.1.14	Glycine--tRNA ligase
Control vs DSS 3-day	6.1.1.15	Proline--tRNA ligase
Control vs DSS 3-day	6.1.1.16	Cysteine--tRNA ligase
Control vs DSS 3-day	6.1.1.17	Glutamate--tRNA ligase
Control vs DSS 3-day	6.1.1.18	Glutamine--tRNA ligase
Control vs DSS 3-day	6.1.1.19	Arginine--tRNA ligase
Control vs DSS 3-day	6.1.1.2	Tryptophan--tRNA ligase
Control vs DSS 3-day	6.1.1.20	Phenylalanine--tRNA ligase
Control vs DSS 3-day	6.1.1.21	Histidine--tRNA ligase
Control vs DSS 3-day	6.1.1.22	Asparagine--tRNA ligase
Control vs DSS 3-day	6.1.1.3	Threonine--tRNA ligase
Control vs DSS 3-day	6.1.1.4	Leucine--tRNA ligase
Control vs DSS 3-day	6.1.1.5	Isoleucine--tRNA ligase
Control vs DSS 3-day	6.1.1.6	Lysine--tRNA ligase
Control vs DSS 3-day	6.1.1.7	Alanine--tRNA ligase
Control vs DSS 3-day	6.1.1.9	Valine--tRNA ligase

Control vs DSS 3-day	6.2.1.30	Phenylacetate--CoA ligase
Control vs DSS 3-day	6.3.1.1	Aspartate--ammonia ligase
Control vs DSS 3-day	6.3.1.2	Glutamate--ammonia ligase
Control vs DSS 3-day	6.3.2.13	UDP-N-acetylmuramoyl-L-alanyl-D-glutamate--2,6-diaminopimelate ligase
Control vs DSS 3-day	6.3.2.4	D-alanine--D-alanine ligase
Control vs DSS 3-day	6.3.2.6	Phosphoribosylaminoimidazolesuccinocarboxamide synthase
Control vs DSS 3-day	6.3.2.8	UDP-N-acetylmuramate--L-alanine ligase
Control vs DSS 3-day	6.3.2.9	UDP-N-acetylmuramoyl-L-alanine--D-glutamate ligase
Control vs DSS 3-day	6.3.3.3	Dethiobiotin synthase
Control vs DSS 3-day	6.3.4.13	Phosphoribosylamine--glycine ligase
Control vs DSS 3-day	6.3.4.14	Biotin carboxylase
Control vs DSS 3-day	6.3.4.19	tRNA(Ile)-lysidine synthetase
Control vs DSS 3-day	6.3.4.2	CTP synthase (glutamine hydrolyzing)
Control vs DSS 3-day	6.3.4.3	Formate--tetrahydrofolate ligase
Control vs DSS 3-day	6.3.4.4	Adenylosuccinate synthase
Control vs DSS 3-day	6.3.5.2	GMP synthase (glutamine-hydrolyzing)
Control vs DSS 3-day	6.3.5.3	Phosphoribosylformylglycinamidine synthase
Control vs DSS 3-day	6.3.5.5	Carbamoyl-phosphate synthase (glutamine-hydrolyzing)
Control vs DSS 3-day	6.4.1.2	Acetyl-CoA carboxylase
Control vs DSS 3-day	6.4.1.3	Propionyl-CoA carboxylase
Control vs DSS 3-day	6.5.1.2	DNA ligase (NAD(+))
Control vs DSS 3-day	7.1.2.2	NO_NAME
Control vs DSS 3-day	7.2.1.1	NO_NAME
Control vs DSS 3-day	1.3.1.9	Enoyl-[acyl-carrier-protein] reductase (NADH)
Control vs DSS 3-day	1.4.1.2	Glutamate dehydrogenase
Control vs DSS 3-day	2.3.1.180	Beta-ketoacyl-[acyl-carrier-protein] synthase III
Control vs DSS 3-day	2.7.7.6	DNA-directed RNA polymerase
Control vs DSS 3-day	4.1.1.49	Phosphoenolpyruvate carboxykinase (ATP)
Control vs DSS 3-day	4.3.2.1	Argininosuccinate lyase
Control vs DSS 3-day	6.1.1.14	Glycine--tRNA ligase
Control vs DSS 3-day	1.7.99.1	Hydroxylamine reductase
Control vs DSS 3-day	2.7.13.3	Histidine kinase
Control vs DSS 3-day	4.1.1.49	Phosphoenolpyruvate carboxykinase (ATP)
Control vs DSS 3-day	2.7.6.1	Ribose-phosphate diphosphokinase
Control vs DSS 3-day	2.7.9.1	Pyruvate, phosphate dikinase
Control vs DSS 3-day	4.1.1.49	Phosphoenolpyruvate carboxykinase (ATP)
Control vs DSS 3-day	1.11.1.1	NADH peroxidase
Control vs DSS 3-day	2.7.13.3	Histidine kinase
Control vs DSS 3-day	2.7.9.1	Pyruvate, phosphate dikinase
Control vs DSS 3-day	2.7.9.1	Pyruvate, phosphate dikinase
Control vs DSS 3-day	2.7.9.1	Pyruvate, phosphate dikinase
Control vs DSS 3-day	2.7.9.1	Pyruvate, phosphate dikinase
Control vs DSS 3-day	1.1.1.205	IMP dehydrogenase

Control vs DSS 3-day	1.1.1.37	Malate dehydrogenase
Control vs DSS 3-day	1.1.1.86	Ketol-acid reductoisomerase (NADP(+))
Control vs DSS 3-day	1.1.1.95	Phosphoglycerate dehydrogenase
Control vs DSS 3-day	1.11.1.1	NADH peroxidase
Control vs DSS 3-day	1.11.1.15	Peroxiredoxin
Control vs DSS 3-day	1.16.3.2	Bacterial non-heme ferritin
Control vs DSS 3-day	1.3.1.9	Enoyl-[acyl-carrier-protein] reductase (NADH)
Control vs DSS 3-day	1.4.1.2	Glutamate dehydrogenase
Control vs DSS 3-day	2.1.2.3	Phosphoribosylaminoimidazolecarboxamide formyltransferase
Control vs DSS 3-day	2.3.1.179	Beta-ketoacyl-[acyl-carrier-protein] synthase II
Control vs DSS 3-day	2.6.1.52	Phosphoserine transaminase
Control vs DSS 3-day	2.7.1.90	Diphosphate--fructose-6-phosphate 1-phosphotransferase
Control vs DSS 3-day	2.7.13.3	Histidine kinase
Control vs DSS 3-day	2.7.2.3	Phosphoglycerate kinase
Control vs DSS 3-day	2.7.6.1	Ribose-phosphate diphosphokinase
Control vs DSS 3-day	2.7.7.6	DNA-directed RNA polymerase
Control vs DSS 3-day	2.7.7.8	Polyribonucleotide nucleotidyltransferase
Control vs DSS 3-day	3.4.11.18	Methionyl aminopeptidase
Control vs DSS 3-day	3.5.1.108	UDP-3-O-acyl-N-acetylglucosamine deacetylase
Control vs DSS 3-day	3.5.4.10	IMP cyclohydrolase
Control vs DSS 3-day	3.5.99.6	Glucosamine-6-phosphate deaminase
Control vs DSS 3-day	4.1.1.15	Glutamate decarboxylase
Control vs DSS 3-day	4.2.1.11	Phosphopyruvate hydratase
Control vs DSS 3-day	4.2.1.59	3-hydroxyacyl-[acyl-carrier-protein] dehydratase
Control vs DSS 3-day	5.1.3.3	Aldose 1-epimerase
Control vs DSS 3-day	5.2.1.8	Peptidylprolyl isomerase
Control vs DSS 3-day	5.3.1.5	Xylose isomerase
Control vs DSS 3-day	5.4.2.12	Phosphoglycerate mutase (2,3-diphosphoglycerate-independent)
Control vs DSS 3-day	5.99.1.3	DNA topoisomerase (ATP-hydrolyzing)
Control vs DSS 3-day	6.1.1.12	Aspartate--tRNA ligase
Control vs DSS 3-day	6.1.1.14	Glycine--tRNA ligase
Control vs DSS 3-day	6.1.1.20	Phenylalanine--tRNA ligase
Control vs DSS 3-day	6.1.1.22	Asparagine--tRNA ligase
Control vs DSS 3-day	6.3.5.3	Phosphoribosylformylglycinamidine synthase
Control vs DSS 3-day	6.3.5.5	Carbamoyl-phosphate synthase (glutamine-hydrolyzing)
Control vs DSS 3-day	7.1.2.2	NO_NAME
Control vs DSS 3-day	7.2.1.1	NO_NAME
Control vs DSS 3-day	2.1.1.72	Site-specific DNA-methyltransferase (adenine-specific)
Control vs DSS 3-day	2.7.13.3	Histidine kinase
Control vs DSS 3-day	3.6.4.12	DNA helicase
Control vs DSS 3-day	3.6.3.20	Glycerol-3-phosphate-transporting ATPase
Control vs DSS 3-day	2.7.9.1	Pyruvate, phosphate dikinase
Control vs DSS 3-day	1.8.99.5	Dissimilatory sulfite reductase

Control vs DSS 3-day	4.1.1.49	Phosphoenolpyruvate carboxykinase (ATP)
Control vs DSS 3-day	1.11.1.1	NADH peroxidase
Control vs DSS 3-day	3.6.3.20	Glycerol-3-phosphate-transporting ATPase
Control vs DSS 3-day	2.7.9.1	Pyruvate, phosphate dikinase
Control vs DSS 3-day	4.1.1.49	Phosphoenolpyruvate carboxykinase (ATP)
Control vs DSS 3-day	2.3.1.8	Phosphate acetyltransferase
Control vs DSS 3-day	2.7.1.23	NAD(+) kinase
Control vs DSS 3-day	2.7.1.40	Pyruvate kinase
Control vs DSS 3-day	2.7.1.69	Protein-N(pi)-phosphohistidine--sugar phosphotransferase
Control vs DSS 3-day	2.7.7.6	DNA-directed RNA polymerase
Control vs DSS 3-day	2.8.4.4	[Ribosomal protein S12] (aspartate(89)-C(3))-methylthiotransferase
Control vs DSS 3-day	3.5.99.6	Glucosamine-6-phosphate deaminase
Control vs DSS 3-day	4.2.1.11	Phosphopyruvate hydratase
Control vs DSS 3-day	5.1.3.2	UDP-glucose 4-epimerase
Control vs DSS 3-day	5.2.1.8	Peptidylprolyl isomerase
Control vs DSS 3-day	1.11.1.1	NADH peroxidase
Control vs DSS 3-day	4.2.1.11	Phosphopyruvate hydratase
Control vs DSS 3-day	4.1.2.9	Phosphoketolase
Control vs DSS 3-day	4.2.1.11	Phosphopyruvate hydratase
Control vs DSS 3-day	1.1.1.86	Ketol-acid reductoisomerase (NADP(+))
Control vs DSS 3-day	2.7.7.6	DNA-directed RNA polymerase
Control vs DSS 3-day	3.6.3.14	H(+)-transporting two-sector ATPase
Control vs DSS 3-day	4.1.1.49	Phosphoenolpyruvate carboxykinase (ATP)
Control vs DSS 3-day	4.1.99.17	Phosphomethylpyrimidine synthase
Control vs DSS 3-day	5.3.1.6	Ribose-5-phosphate isomerase
Control vs DSS 3-day	1.11.1.15	Peroxiredoxin
Control vs DSS 3-day	1.11.1.15	Peroxiredoxin
Control vs DSS 3-day	1.1.1.27	L-lactate dehydrogenase
Control vs DSS 3-day	1.2.1.12	Glyceraldehyde-3-phosphate dehydrogenase (phosphorylating)
Control vs DSS 3-day	2.7.1.40	Pyruvate kinase
Control vs DSS 3-day	2.7.1.69	Protein-N(pi)-phosphohistidine--sugar phosphotransferase
Control vs DSS 3-day	2.7.2.3	Phosphoglycerate kinase
Control vs DSS 3-day	2.7.6.1	Ribose-phosphate diphosphokinase
Control vs DSS 3-day	2.7.7.6	DNA-directed RNA polymerase
Control vs DSS 3-day	3.2.1.122	Maltose-6'-phosphate glucosidase
Control vs DSS 3-day	4.1.2.40	Tagatose-bisphosphate aldolase
Control vs DSS 3-day	4.2.1.11	Phosphopyruvate hydratase
Control vs DSS 3-day	5.4.2.10	Phosphoglucosamine mutase
Control vs DSS 3-day	5.4.2.11	Phosphoglycerate mutase (2,3-diphosphoglycerate-dependent)
Control vs DSS 3-day	6.1.1.3	Threonine--tRNA ligase
Control vs DSS 3-day	6.3.1.2	Glutamate--ammonia ligase
Control vs DSS 3-day	4.3.2.2	Adenylosuccinate lyase
Control vs DSS 3-day	6.3.5.2	GMP synthase (glutamine-hydrolyzing)

Control vs DSS 3-day	1.1.1.100	3-oxoacyl-[acyl-carrier-protein] reductase
Control vs DSS 3-day	1.1.1.133	dTDP-4-dehydrorhamnose reductase
Control vs DSS 3-day	1.1.1.218	Morphine 6-dehydrogenase
Control vs DSS 3-day	1.1.1.27	L-lactate dehydrogenase
Control vs DSS 3-day	1.1.1.28	D-lactate dehydrogenase
Control vs DSS 3-day	1.1.1.44	Phosphogluconate dehydrogenase (NADP(+)-dependent, decarboxylating)
Control vs DSS 3-day	1.1.1.49	Glucose-6-phosphate dehydrogenase (NADP(+))
Control vs DSS 3-day	1.1.1.88	Hydroxymethylglutaryl-CoA reductase
Control vs DSS 3-day	1.1.1.94	Glycerol-3-phosphate dehydrogenase (NAD(P)(+))
Control vs DSS 3-day	1.11.1.15	Peroxiredoxin
Control vs DSS 3-day	1.18.1.2	Ferredoxin--NADP(+) reductase
Control vs DSS 3-day	1.2.3.3	Pyruvate oxidase
Control vs DSS 3-day	1.3.1.98	UDP-N-acetylmuramate dehydrogenase
Control vs DSS 3-day	1.5.1.36	Flavin reductase (NADH)
Control vs DSS 3-day	1.7.1.7	GMP reductase
Control vs DSS 3-day	1.8.1.7	Glutathione-disulfide reductase
Control vs DSS 3-day	1.8.1.9	Thioredoxin-disulfide reductase
Control vs DSS 3-day	1.8.4.12	Peptide-methionine (R)-S-oxide reductase
Control vs DSS 3-day	2.1.1.14	5-methyltetrahydropteroyltriglutamate--homocysteine S-methyltransferase
Control vs DSS 3-day	2.1.1.163	Demethylmenaquinone methyltransferase
Control vs DSS 3-day	2.1.1.182	16S rRNA (adenine(1518)-N(6)/adenine(1519)-N(6))-dimethyltransferase
Control vs DSS 3-day	2.1.1.199	16S rRNA (cytosine(1402)-N(4))-methyltransferase
Control vs DSS 3-day	2.1.1.207	tRNA (cytidine(34)-2'-O)-methyltransferase
Control vs DSS 3-day	2.1.1.33	tRNA (guanine(46)-N(7))-methyltransferase
Control vs DSS 3-day	2.1.1.45	Thymidylate synthase
Control vs DSS 3-day	2.1.1.74	(FADH(2)-oxidizing)
Control vs DSS 3-day	2.1.2.1	Glycine hydroxymethyltransferase
Control vs DSS 3-day	2.3.1.157	Glucosamine-1-phosphate N-acetyltransferase
Control vs DSS 3-day	2.3.1.8	Phosphate acetyltransferase
Control vs DSS 3-day	2.3.1.81	Aminoglycoside N(3')-acetyltransferase
Control vs DSS 3-day	2.3.2.3	Lysyltransferase
Control vs DSS 3-day	2.4.1.227	acetylglucosaminyltransferase
Control vs DSS 3-day	2.4.2.6	Nucleoside deoxyribosyltransferase
Control vs DSS 3-day	2.4.2.8	Hypoxanthine phosphoribosyltransferase
Control vs DSS 3-day	2.4.2.9	Uracil phosphoribosyltransferase
Control vs DSS 3-day	2.5.1.145	NO_NAME
Control vs DSS 3-day	2.5.1.30	Heptaprenyl diphosphate synthase
Control vs DSS 3-day	2.5.1.6	Methionine adenosyltransferase
Control vs DSS 3-day	2.5.1.7	UDP-N-acetylglucosamine 1-carboxyvinyltransferase
Control vs DSS 3-day	2.6.1.16	Glutamine--fructose-6-phosphate transaminase (isomerizing)
Control vs DSS 3-day	2.7.1.11	6-phosphofructokinase
Control vs DSS 3-day	2.7.1.113	Deoxyguanosine kinase
Control vs DSS 3-day	2.7.1.144	Tagatose-6-phosphate kinase

Control vs DSS 3-day	2.7.1.15	Ribokinase
Control vs DSS 3-day	2.7.1.180	FAD
Control vs DSS 3-day	2.7.1.24	Dephospho-CoA kinase
Control vs DSS 3-day	2.7.1.26	Riboflavin kinase
Control vs DSS 3-day	2.7.1.33	Pantothenate kinase
Control vs DSS 3-day	2.7.1.40	Pyruvate kinase
Control vs DSS 3-day	2.7.1.6	Galactokinase
Control vs DSS 3-day	2.7.1.69	Protein-N(pi)-phosphohistidine--sugar phosphotransferase
Control vs DSS 3-day	2.7.1.76	Deoxyadenosine kinase
Control vs DSS 3-day	2.7.11.1	Non-specific serine/threonine protein kinase
Control vs DSS 3-day	2.7.11.32	[Pyruvate, phosphate dikinase] kinase
Control vs DSS 3-day	2.7.13.3	Histidine kinase
Control vs DSS 3-day	2.7.2.1	Acetate kinase
Control vs DSS 3-day	2.7.2.3	Phosphoglycerate kinase
Control vs DSS 3-day	2.7.3.9	Phosphoenolpyruvate--protein phosphotransferase
Control vs DSS 3-day	2.7.4.22	UMP kinase
Control vs DSS 3-day	2.7.4.25	(d)CMP kinase
Control vs DSS 3-day	2.7.4.27	([Pyruvate, phosphate dikinase] phosphate) phosphotransferase
Control vs DSS 3-day	2.7.4.3	Adenylate kinase
Control vs DSS 3-day	2.7.4.8	Guanylate kinase
Control vs DSS 3-day	2.7.6.1	Ribose-phosphate diphosphokinase
Control vs DSS 3-day	2.7.7.12	UDP-glucose--hexose-1-phosphate uridylyltransferase
Control vs DSS 3-day	2.7.7.18	Nicotinate-nucleotide adenylyltransferase
Control vs DSS 3-day	2.7.7.2	FAD synthetase
Control vs DSS 3-day	2.7.7.23	UDP-N-acetylglucosamine diphosphorylase
Control vs DSS 3-day	2.7.7.41	Phosphatidate cytidylyltransferase
Control vs DSS 3-day	2.7.7.6	DNA-directed RNA polymerase
Control vs DSS 3-day	2.7.7.7	DNA-directed DNA polymerase
Control vs DSS 3-day	2.7.7.72	CCA tRNA nucleotidyltransferase
Control vs DSS 3-day	2.7.7.85	Diadenylate cyclase
Control vs DSS 3-day	2.7.7.9	UTP--glucose-1-phosphate uridylyltransferase
Control vs DSS 3-day	2.7.8.13	Phospho-N-acetylmuramoyl-pentapeptide-transferase
Control vs DSS 3-day	2.7.8.5	CDP-diacylglycerol--glycerol-3-phosphate 3-phosphatidyltransferase
Control vs DSS 3-day	2.7.8.7	Holo-[acyl-carrier-protein] synthase
Control vs DSS 3-day	2.8.1.13	tRNA-uridine 2-sulfurtransferase
Control vs DSS 3-day	2.8.1.4	tRNA sulfurtransferase
Control vs DSS 3-day	2.8.1.7	Cysteine desulfurase
Control vs DSS 3-day	2.8.3.16	Formyl-CoA transferase
Control vs DSS 3-day	3.1.1.96	D-aminoacyl-tRNA deacylase
Control vs DSS 3-day	3.1.11.2	Exodeoxyribonuclease III
Control vs DSS 3-day	3.1.13.1	Exoribonuclease II
Control vs DSS 3-day	3.1.26.11	Ribonuclease Z
Control vs DSS 3-day	3.1.26.4	Ribonuclease H

Control vs DSS 3-day	3.1.26.5	Ribonuclease P
Control vs DSS 3-day	3.1.26.8	Ribonuclease M5
Control vs DSS 3-day	3.1.3.16	Protein-serine/threonine phosphatase
Control vs DSS 3-day	3.1.3.23	Sugar-phosphatase
Control vs DSS 3-day	3.1.3.48	Protein-tyrosine-phosphatase
Control vs DSS 3-day	3.1.3.5	5'-nucleotidase
Control vs DSS 3-day	3.1.3.73	Adenosylcobalamin/alpha-ribazole phosphatase
Control vs DSS 3-day	3.1.5.1	dGTPase
Control vs DSS 3-day	3.2.1.10	Oligo-1,6-glucosidase
Control vs DSS 3-day	3.2.1.122	Maltose-6'-phosphate glucosidase
Control vs DSS 3-day	3.2.1.135	Neopullulanase
Control vs DSS 3-day	3.2.1.17	Lysozyme
Control vs DSS 3-day	3.2.1.20	Alpha-glucosidase
Control vs DSS 3-day	3.2.1.23	Beta-galactosidase
Control vs DSS 3-day	3.2.1.26	Beta-fructofuranosidase
Control vs DSS 3-day	3.2.1.86	6-phospho-beta-glucosidase
Control vs DSS 3-day	3.2.2.1	Purine nucleosidase
Control vs DSS 3-day	3.2.2.23	DNA-formamidopyrimidine glycosylase
Control vs DSS 3-day	3.2.2.27	Uracil-DNA glycosylase
Control vs DSS 3-day	3.2.2.9	Adenosylhomocysteine nucleosidase
Control vs DSS 3-day	3.4.11.18	Methionyl aminopeptidase
Control vs DSS 3-day	3.4.11.4	Tripeptide aminopeptidase
Control vs DSS 3-day	3.4.11.5	Prolyl aminopeptidase
Control vs DSS 3-day	3.4.13.3	Transferred entry
Control vs DSS 3-day	3.4.13.9	Xaa-Pro dipeptidase
Control vs DSS 3-day	3.4.14.11	Xaa-Pro dipeptidyl-peptidase
Control vs DSS 3-day	3.4.19.3	Pyroglutamyl-peptidase I
Control vs DSS 3-day	3.4.21.102	C-terminal processing peptidase
Control vs DSS 3-day	3.4.21.88	Repressor LexA
Control vs DSS 3-day	3.4.21.89	Signal peptidase I
Control vs DSS 3-day	3.4.21.92	Endopeptidase Clp
Control vs DSS 3-day	3.4.22.40	Bleomycin hydrolase
Control vs DSS 3-day	3.4.23.36	Signal peptidase II
Control vs DSS 3-day	3.4.25.2	HslU--HslV peptidase
Control vs DSS 3-day	3.5.1.2	Glutaminase
Control vs DSS 3-day	3.5.1.24	Choloylglycine hydrolase
Control vs DSS 3-day	3.5.1.88	Peptide deformylase
Control vs DSS 3-day	3.5.99.6	Glucosamine-6-phosphate deaminase
Control vs DSS 3-day	3.6.1.1	Inorganic diphosphatase
Control vs DSS 3-day	3.6.1.66	XTP/dITP diphosphatase
Control vs DSS 3-day	3.6.3.3	Cadmium-exporting ATPase
Control vs DSS 3-day	3.6.4.12	DNA helicase
Control vs DSS 3-day	3.6.5.3	Protein-synthesizing GTPase

Control vs DSS 3-day	3.6.5.n1	Elongation factor 4
Control vs DSS 3-day	4.1.1.31	Phosphoenolpyruvate carboxylase
Control vs DSS 3-day	4.1.1.5	Acetolactate decarboxylase
Control vs DSS 3-day	4.1.2.4	Deoxyribose-phosphate aldolase
Control vs DSS 3-day	4.1.2.40	Tagatose-bisphosphate aldolase
Control vs DSS 3-day	4.2.1.11	Phosphopyruvate hydratase
Control vs DSS 3-day	4.2.1.2	Fumarate hydratase
Control vs DSS 3-day	4.2.99.18	DNA-(apurinic or apyrimidinic site) lyase
Control vs DSS 3-day	4.3.2.2	Adenylosuccinate lyase
Control vs DSS 3-day	4.4.1.21	S-ribosylhomocysteine lyase
Control vs DSS 3-day	5.1.1.1	Alanine racemase
Control vs DSS 3-day	5.1.1.13	Aspartate racemase
Control vs DSS 3-day	5.1.3.1	Ribulose-phosphate 3-epimerase
Control vs DSS 3-day	5.2.1.8	Peptidylprolyl isomerase
Control vs DSS 3-day	5.3.1.1	Triose-phosphate isomerase
Control vs DSS 3-day	5.3.1.26	Galactose-6-phosphate isomerase
Control vs DSS 3-day	5.3.1.6	Ribose-5-phosphate isomerase
Control vs DSS 3-day	5.3.1.8	Mannose-6-phosphate isomerase
Control vs DSS 3-day	5.3.1.9	Glucose-6-phosphate isomerase
Control vs DSS 3-day	5.3.3.2	Isopentenyl-diphosphate Delta-isomerase
Control vs DSS 3-day	5.4.2.10	Phosphoglucosamine mutase
Control vs DSS 3-day	5.4.2.11	Phosphoglycerate mutase (2,3-diphosphoglycerate-dependent)
Control vs DSS 3-day	5.4.2.2	Phosphoglucomutase (alpha-D-glucose-1,6-bisphosphate-dependent)
Control vs DSS 3-day	5.4.2.6	Beta-phosphoglucomutase
Control vs DSS 3-day	5.4.99.12	tRNA pseudouridine(38-40) synthase
Control vs DSS 3-day	5.99.1.3	DNA topoisomerase (ATP-hydrolyzing)
Control vs DSS 3-day	6.1.1.10	Methionine--tRNA ligase
Control vs DSS 3-day	6.1.1.11	Serine--tRNA ligase
Control vs DSS 3-day	6.1.1.12	Aspartate--tRNA ligase
Control vs DSS 3-day	6.1.1.14	Glycine--tRNA ligase
Control vs DSS 3-day	6.1.1.15	Proline--tRNA ligase
Control vs DSS 3-day	6.1.1.17	Glutamate--tRNA ligase
Control vs DSS 3-day	6.1.1.2	Tryptophan--tRNA ligase
Control vs DSS 3-day	6.1.1.20	Phenylalanine--tRNA ligase
Control vs DSS 3-day	6.1.1.22	Asparagine--tRNA ligase
Control vs DSS 3-day	6.1.1.3	Threonine--tRNA ligase
Control vs DSS 3-day	6.1.1.5	Isoleucine--tRNA ligase
Control vs DSS 3-day	6.1.1.6	Lysine--tRNA ligase
Control vs DSS 3-day	6.1.1.7	Alanine--tRNA ligase
Control vs DSS 3-day	6.1.1.9	Valine--tRNA ligase
Control vs DSS 3-day	6.3.1.2	Glutamate--ammonia ligase
Control vs DSS 3-day	6.3.1.5	NAD(+) synthase
Control vs DSS 3-day	6.3.2.10	UDP-N-acetylmuramoyl-tripeptide--D-alanyl-D-alanine ligase

Control vs DSS 3-day	6.3.2.4	D-alanine--D-alanine ligase
Control vs DSS 3-day	6.3.2.9	UDP-N-acetylmuramoyl-L-alanine--D-glutamate ligase
Control vs DSS 3-day	6.3.4.2	CTP synthase (glutamine hydrolyzing)
Control vs DSS 3-day	6.3.4.21	Nicotinate phosphoribosyltransferase
Control vs DSS 3-day	6.3.4.4	Adenylosuccinate synthase
Control vs DSS 3-day	6.3.5.2	GMP synthase (glutamine-hydrolyzing)
Control vs DSS 3-day	6.3.5.4	Asparagine synthase (glutamine-hydrolyzing)
Control vs DSS 3-day	6.3.5.5	Carbamoyl-phosphate synthase (glutamine-hydrolyzing)
Control vs DSS 3-day	6.3.5.7	Glutaminyl-tRNA synthase (glutamine-hydrolyzing)
Control vs DSS 3-day	6.5.1.2	DNA ligase (NAD(+))
Control vs DSS 3-day	7.1.2.2	NO_NAME
Control vs DSS 3-day	7.3.2.1	NO_NAME
Control vs DSS 3-day	1.2.1.12	Glyceraldehyde-3-phosphate dehydrogenase (phosphorylating)
Control vs DSS 3-day	2.7.2.3	Phosphoglycerate kinase
Control vs DSS 3-day	2.7.7.6	DNA-directed RNA polymerase
Control vs DSS 3-day	3.2.1.122	Maltose-6'-phosphate glucosidase
Control vs DSS 3-day	3.4.19.3	Pyroglutamyl-peptidase I
Control vs DSS 3-day	4.1.2.40	Tagatose-bisphosphate aldolase
Control vs DSS 3-day	4.2.1.11	Phosphopyruvate hydratase
Control vs DSS 3-day	5.4.2.11	Phosphoglycerate mutase (2,3-diphosphoglycerate-dependent)
Control vs DSS 3-day	6.1.1.22	Asparagine--tRNA ligase
Control vs DSS 3-day	6.3.1.2	Glutamate--ammonia ligase
Control vs DSS 3-day	1.1.1.133	dTDP-4-dehydrorhamnose reductase
Control vs DSS 3-day	1.1.1.27	L-lactate dehydrogenase
Control vs DSS 3-day	1.2.1.12	Glyceraldehyde-3-phosphate dehydrogenase (phosphorylating)
Control vs DSS 3-day	1.7.1.7	GMP reductase
Control vs DSS 3-day	2.5.1.30	Heptaprenyl diphosphate synthase
Control vs DSS 3-day	2.7.1.144	Tagatose-6-phosphate kinase
Control vs DSS 3-day	2.7.1.26	Riboflavin kinase
Control vs DSS 3-day	2.7.1.40	Pyruvate kinase
Control vs DSS 3-day	2.7.1.6	Galactokinase
Control vs DSS 3-day	2.7.1.69	Protein-N(pi)-phosphohistidine--sugar phosphotransferase
Control vs DSS 3-day	2.7.10.1	Receptor protein-tyrosine kinase
Control vs DSS 3-day	2.7.13.3	Histidine kinase
Control vs DSS 3-day	2.7.3.9	Phosphoenolpyruvate--protein phosphotransferase
Control vs DSS 3-day	2.7.6.1	Ribose-phosphate diphosphokinase
Control vs DSS 3-day	2.7.7.12	UDP-glucose--hexose-1-phosphate uridylyltransferase
Control vs DSS 3-day	2.7.7.2	FAD synthetase
Control vs DSS 3-day	2.7.7.6	DNA-directed RNA polymerase
Control vs DSS 3-day	2.7.7.7	DNA-directed DNA polymerase
Control vs DSS 3-day	3.1.3.1	Alkaline phosphatase
Control vs DSS 3-day	3.1.3.23	Sugar-phosphatase
Control vs DSS 3-day	3.1.3.48	Protein-tyrosine-phosphatase

Control vs DSS 3-day	3.1.3.5	5'-nucleotidase
Control vs DSS 3-day	3.2.1.10	Oligo-1,6-glucosidase
Control vs DSS 3-day	3.2.1.135	Neopullulanase
Control vs DSS 3-day	3.2.1.20	Alpha-glucosidase
Control vs DSS 3-day	3.2.1.23	Beta-galactosidase
Control vs DSS 3-day	3.2.1.86	6-phospho-beta-glucosidase
Control vs DSS 3-day	3.4.14.11	Xaa-Pro dipeptidyl-peptidase
Control vs DSS 3-day	3.4.22.40	Bleomycin hydrolase
Control vs DSS 3-day	3.5.1.2	Glutaminase
Control vs DSS 3-day	3.6.4.12	DNA helicase
Control vs DSS 3-day	4.2.1.11	Phosphopyruvate hydratase
Control vs DSS 3-day	5.2.1.8	Peptidylprolyl isomerase
Control vs DSS 3-day	5.4.2.11	Phosphoglycerate mutase (2,3-diphosphoglycerate-dependent)
Control vs DSS 3-day	6.1.1.14	Glycine--tRNA ligase
Control vs DSS 3-day	6.1.1.20	Phenylalanine--tRNA ligase
Control vs DSS 3-day	6.1.1.22	Asparagine--tRNA ligase
Control vs DSS 3-day	6.1.1.3	Threonine--tRNA ligase
Control vs DSS 3-day	6.3.4.21	Nicotinate phosphoribosyltransferase
Control vs DSS 3-day	6.3.5.4	Asparagine synthase (glutamine-hydrolyzing)
Control vs DSS 3-day	6.3.5.5	Carbamoyl-phosphate synthase (glutamine-hydrolyzing)
Control vs DSS 3-day	7.1.2.2	NO_NAME
Control vs DSS 3-day	4.2.1.11	Phosphopyruvate hydratase
Control vs DSS 3-day	4.1.1.49	Phosphoenolpyruvate carboxykinase (ATP)
Control vs DSS 3-day	1.1.1.133	dTDP-4-dehydrorhamnose reductase
Control vs DSS 3-day	1.1.1.193	5-amino-6-(5-phosphoribosylamino)uracil reductase
Control vs DSS 3-day	1.1.1.205	IMP dehydrogenase
Control vs DSS 3-day	1.1.1.23	Histidinol dehydrogenase
Control vs DSS 3-day	1.1.1.25	Shikimate dehydrogenase
Control vs DSS 3-day	1.1.1.267	1-deoxy-D-xylulose-5-phosphate reductoisomerase
Control vs DSS 3-day	1.1.1.3	Homoserine dehydrogenase
Control vs DSS 3-day	1.1.1.37	Malate dehydrogenase
Control vs DSS 3-day	1.1.1.42	Isocitrate dehydrogenase (NADP(+))
Control vs DSS 3-day	1.1.1.85	3-isopropylmalate dehydrogenase
Control vs DSS 3-day	1.1.1.86	Ketol-acid reductoisomerase (NADP(+))
Control vs DSS 3-day	1.1.1.94	Glycerol-3-phosphate dehydrogenase (NAD(P)(+))
Control vs DSS 3-day	1.1.1.95	Phosphoglycerate dehydrogenase
Control vs DSS 3-day	1.11.1.15	Peroxiredoxin
Control vs DSS 3-day	1.11.1.6	Catalase
Control vs DSS 3-day	1.17.1.8	4-hydroxy-tetrahydrodipicolinate reductase
Control vs DSS 3-day	1.17.4.1	Ribonucleoside-diphosphate reductase
Control vs DSS 3-day	1.17.7.3	(E)-4-hydroxy-3-methylbut-2-enyl-diphosphate synthase (flavodoxin)
Control vs DSS 3-day	1.17.7.4	NO_NAME
Control vs DSS 3-day	1.2.1.11	Aspartate-semialdehyde dehydrogenase

Control vs DSS 3-day	1.2.1.38	N-acetyl-gamma-glutamyl-phosphate reductase
Control vs DSS 3-day	1.2.1.70	Glutamyl-tRNA reductase
Control vs DSS 3-day	1.21.98.1	Cyclic dehypoxanthinyl futalosine synthase
Control vs DSS 3-day	1.3.1.9	Enoyl-[acyl-carrier-protein] reductase (NADH)
Control vs DSS 3-day	1.3.3.4	Protoporphyrinogen oxidase
Control vs DSS 3-day	1.3.5.1	Succinate dehydrogenase (quinone)
Control vs DSS 3-day	1.4.1.16	Diaminopimelate dehydrogenase
Control vs DSS 3-day	1.5.1.2	Pyrroline-5-carboxylate reductase
Control vs DSS 3-day	1.5.1.20	Methylenetetrahydrofolate reductase (NAD(P)H)
Control vs DSS 3-day	1.5.1.5	Methylenetetrahydrofolate dehydrogenase (NADP(+))
Control vs DSS 3-day	1.7.2.2	Nitrite reductase (cytochrome; ammonia-forming)
Control vs DSS 3-day	1.8.1.9	Thioredoxin-disulfide reductase
Control vs DSS 3-day	1.8.4.11	Peptide-methionine (S)-S-oxide reductase
Control vs DSS 3-day	1.8.4.12	Peptide-methionine (R)-S-oxide reductase
Control vs DSS 3-day	2.1.1.107	Uroporphyrinogen-III C-methyltransferase
Control vs DSS 3-day	2.1.1.13	Methionine synthase
Control vs DSS 3-day	2.1.1.148	Thymidylate synthase (FAD)
Control vs DSS 3-day	2.1.1.163	Demethylmenaquinone methyltransferase
Control vs DSS 3-day	2.1.1.177	23S rRNA (pseudouridine(1915)-N(3))-methyltransferase
Control vs DSS 3-day	2.1.1.182	16S rRNA (adenine(1518)-N(6)/adenine(1519)-N(6))-dimethyltransferase
Control vs DSS 3-day	2.1.1.192	23S rRNA (adenine(2503)-C(2))-methyltransferase
Control vs DSS 3-day	2.1.1.193	16S rRNA (uracil(1498)-N(3))-methyltransferase
Control vs DSS 3-day	2.1.1.195	Cobalt-precorrin-5B (C(1))-methyltransferase
Control vs DSS 3-day	2.1.1.198	16S rRNA (cytidine(1402)-2'-O)-methyltransferase
Control vs DSS 3-day	2.1.1.199	16S rRNA (cytosine(1402)-N(4))-methyltransferase
Control vs DSS 3-day	2.1.1.220	tRNA (adenine(58)-N(1))-methyltransferase
Control vs DSS 3-day	2.1.1.228	tRNA (guanine(37)-N(1))-methyltransferase
Control vs DSS 3-day	2.1.1.33	tRNA (guanine(46)-N(7))-methyltransferase
Control vs DSS 3-day	2.1.1.37	DNA (cytosine-5-)-methyltransferase
Control vs DSS 3-day	2.1.1.72	Site-specific DNA-methyltransferase (adenine-specific)
Control vs DSS 3-day	2.1.1.77	Protein-L-isoaspartate(D-aspartate) O-methyltransferase
Control vs DSS 3-day	2.1.2.1	Glycine hydroxymethyltransferase
Control vs DSS 3-day	2.1.2.11	3-methyl-2-oxobutanoate hydroxymethyltransferase
Control vs DSS 3-day	2.1.2.2	Phosphoribosylglycinamide formyltransferase
Control vs DSS 3-day	2.1.2.3	Phosphoribosylaminoimidazolecarboxamide formyltransferase
Control vs DSS 3-day	2.1.2.9	Methionyl-tRNA formyltransferase
Control vs DSS 3-day	2.1.3.2	Aspartate carbamoyltransferase
Control vs DSS 3-day	2.1.3.3	Ornithine carbamoyltransferase
Control vs DSS 3-day	2.2.1.6	Acetolactate synthase
Control vs DSS 3-day	2.2.1.7	1-deoxy-D-xylulose-5-phosphate synthase
Control vs DSS 3-day	2.3.1.1	Amino-acid N-acetyltransferase
Control vs DSS 3-day	2.3.1.157	Glucosamine-1-phosphate N-acetyltransferase
Control vs DSS 3-day	2.3.1.179	Beta-ketoacyl-[acyl-carrier-protein] synthase II

Control vs DSS 3-day	2.3.1.234	N(6)-L-threonylcarbamoyladenine synthase
Control vs DSS 3-day	2.3.1.266	NO_NAME
Control vs DSS 3-day	2.3.1.31	Homoserine O-acetyltransferase
Control vs DSS 3-day	2.3.1.35	Glutamate N-acetyltransferase
Control vs DSS 3-day	2.3.1.39	[Acyl-carrier-protein] S-malonyltransferase
Control vs DSS 3-day	2.3.1.51	1-acylglycerol-3-phosphate O-acyltransferase
Control vs DSS 3-day	2.3.1.n2	Phosphate acyltransferase
Control vs DSS 3-day	2.3.1.n3	Glycerol-3-phosphate acyltransferase (acyl-phosphate transferring)
Control vs DSS 3-day	2.3.3.13	2-isopropylmalate synthase
Control vs DSS 3-day	2.4.1.182	Lipid-A-disaccharide synthase
Control vs DSS 3-day	2.4.1.21	Starch synthase
Control vs DSS 3-day	2.4.1.227	acetylglucosaminyltransferase
Control vs DSS 3-day	2.4.2.10	Orotate phosphoribosyltransferase
Control vs DSS 3-day	2.4.2.14	Amidophosphoribosyltransferase
Control vs DSS 3-day	2.4.2.17	ATP phosphoribosyltransferase
Control vs DSS 3-day	2.4.2.21	Nicotinate-nucleotide--dimethylbenzimidazole phosphoribosyltransferase
Control vs DSS 3-day	2.4.2.29	tRNA-guanine(34) transglycosylase
Control vs DSS 3-day	2.4.2.9	Uracil phosphoribosyltransferase
Control vs DSS 3-day	2.4.99.17	S-adenosylmethionine
Control vs DSS 3-day	2.5.1.129	NO_NAME
Control vs DSS 3-day	2.5.1.19	3-phosphoshikimate 1-carboxyvinyltransferase
Control vs DSS 3-day	2.5.1.55	3-deoxy-8-phosphooctulonate synthase
Control vs DSS 3-day	2.5.1.6	Methionine adenosyltransferase
Control vs DSS 3-day	2.5.1.61	Hydroxymethylbilane synthase
Control vs DSS 3-day	2.5.1.7	UDP-N-acetylglucosamine 1-carboxyvinyltransferase
Control vs DSS 3-day	2.5.1.75	tRNA dimethylallyltransferase
Control vs DSS 3-day	2.5.1.78	6,7-dimethyl-8-ribityllumazine synthase
Control vs DSS 3-day	2.6.1.16	Glutamine--fructose-6-phosphate transaminase (isomerizing)
Control vs DSS 3-day	2.6.1.42	Branched-chain-amino-acid transaminase
Control vs DSS 3-day	2.6.1.62	Adenosylmethionine--8-amino-7-oxononanoate transaminase
Control vs DSS 3-day	2.6.1.9	Histidinol-phosphate transaminase
Control vs DSS 3-day	2.6.99.2	Pyridoxine 5'-phosphate synthase
Control vs DSS 3-day	2.7.1.11	6-phosphofructokinase
Control vs DSS 3-day	2.7.1.130	Tetraacyldisaccharide 4'-kinase
Control vs DSS 3-day	2.7.1.167	D-glycero-beta-D-manno-heptose-7-phosphate kinase
Control vs DSS 3-day	2.7.1.23	NAD(+) kinase
Control vs DSS 3-day	2.7.1.24	Dephospho-CoA kinase
Control vs DSS 3-day	2.7.1.26	Riboflavin kinase
Control vs DSS 3-day	2.7.1.30	Glycerol kinase
Control vs DSS 3-day	2.7.1.33	Pantothenate kinase
Control vs DSS 3-day	2.7.1.40	Pyruvate kinase
Control vs DSS 3-day	2.7.13.3	Histidine kinase
Control vs DSS 3-day	2.7.2.1	Acetate kinase

Control vs DSS 3-day	2.7.2.3	Phosphoglycerate kinase
Control vs DSS 3-day	2.7.2.4	Aspartate kinase
Control vs DSS 3-day	2.7.2.8	Acetylglutamate kinase
Control vs DSS 3-day	2.7.3.9	Phosphoenolpyruvate--protein phosphotransferase
Control vs DSS 3-day	2.7.4.1	Polyphosphate kinase
Control vs DSS 3-day	2.7.4.22	UMP kinase
Control vs DSS 3-day	2.7.4.25	(d)CMP kinase
Control vs DSS 3-day	2.7.4.3	Adenylate kinase
Control vs DSS 3-day	2.7.4.6	Nucleoside-diphosphate kinase
Control vs DSS 3-day	2.7.4.9	dTMP kinase
Control vs DSS 3-day	2.7.6.1	Ribose-phosphate diphosphokinase
Control vs DSS 3-day	2.7.7.2	FAD synthetase
Control vs DSS 3-day	2.7.7.23	UDP-N-acetylglucosamine diphosphorylase
Control vs DSS 3-day	2.7.7.24	Glucose-1-phosphate thymidylyltransferase
Control vs DSS 3-day	2.7.7.3	Pantetheine-phosphate adenylyltransferase
Control vs DSS 3-day	2.7.7.38	3-deoxy-manno-octulosonate cytidylyltransferase
Control vs DSS 3-day	2.7.7.41	Phosphatidate cytidylyltransferase
Control vs DSS 3-day	2.7.7.6	DNA-directed RNA polymerase
Control vs DSS 3-day	2.7.7.60	2-C-methyl-D-erythritol 4-phosphate cytidylyltransferase
Control vs DSS 3-day	2.7.7.7	DNA-directed DNA polymerase
Control vs DSS 3-day	2.7.7.70	D-glycero-beta-D-manno-heptose 1-phosphate adenylyltransferase
Control vs DSS 3-day	2.7.7.77	Molybdenum cofactor guanylyltransferase
Control vs DSS 3-day	2.7.7.8	Polyribonucleotide nucleotidyltransferase
Control vs DSS 3-day	2.7.7.85	Diadenylate cyclase
Control vs DSS 3-day	2.7.7.9	UTP--glucose-1-phosphate uridylyltransferase
Control vs DSS 3-day	2.7.8.13	Phospho-N-acetylmuramoyl-pentapeptide-transferase
Control vs DSS 3-day	2.7.8.26	Adenosylcobinamide-GDP ribazoletransferase
Control vs DSS 3-day	2.7.8.7	Holo-[acyl-carrier-protein] synthase
Control vs DSS 3-day	2.8.1.10	Thiazole synthase
Control vs DSS 3-day	2.8.1.6	Biotin synthase
Control vs DSS 3-day	2.8.4.3	tRNA-2-methylthio-N(6)-dimethylallyladenosine synthase
Control vs DSS 3-day	2.8.4.4	[Ribosomal protein S12] (aspartate(89)-C(3))-methylthiotransferase
Control vs DSS 3-day	3.1.1.29	Aminoacyl-tRNA hydrolase
Control vs DSS 3-day	3.1.1.61	Protein-glutamate methylesterase
Control vs DSS 3-day	3.1.1.96	D-aminoacyl-tRNA deacylase
Control vs DSS 3-day	3.1.11.6	Exodeoxyribonuclease VII
Control vs DSS 3-day	3.1.13.1	Exoribonuclease II
Control vs DSS 3-day	3.1.21.2	Deoxyribonuclease IV
Control vs DSS 3-day	3.1.26.3	Ribonuclease III
Control vs DSS 3-day	3.1.26.4	Ribonuclease H
Control vs DSS 3-day	3.1.26.5	Ribonuclease P
Control vs DSS 3-day	3.1.3.25	Inositol-phosphate phosphatase
Control vs DSS 3-day	3.1.3.5	5'-nucleotidase

Control vs DSS 3-day	3.1.4.58	NO_NAME
Control vs DSS 3-day	3.2.2.n1	Cytokinin riboside 5'-monophosphate phosphoribohydrolase
Control vs DSS 3-day	3.3.1.1	Adenosylhomocysteinase
Control vs DSS 3-day	3.4.11.18	Methionyl aminopeptidase
Control vs DSS 3-day	3.4.21.53	Endopeptidase La
Control vs DSS 3-day	3.4.21.88	Repressor LexA
Control vs DSS 3-day	3.4.21.89	Signal peptidase I
Control vs DSS 3-day	3.4.21.92	Endopeptidase Clp
Control vs DSS 3-day	3.4.23.36	Signal peptidase II
Control vs DSS 3-day	3.4.25.2	HslU--HslV peptidase
Control vs DSS 3-day	3.5.1.108	UDP-3-O-acyl-N-acetylglucosamine deacetylase
Control vs DSS 3-day	3.5.1.2	Glutaminase
Control vs DSS 3-day	3.5.1.44	Protein-glutamine glutaminase
Control vs DSS 3-day	3.5.1.88	Peptide deformylase
Control vs DSS 3-day	3.5.2.3	Dihydroorotase
Control vs DSS 3-day	3.5.4.10	IMP cyclohydrolase
Control vs DSS 3-day	3.5.4.16	GTP cyclohydrolase I
Control vs DSS 3-day	3.5.4.19	Phosphoribosyl-AMP cyclohydrolase
Control vs DSS 3-day	3.5.4.25	GTP cyclohydrolase II
Control vs DSS 3-day	3.5.4.26	Diaminohydroxyphosphoribosylaminopyrimidine deaminase
Control vs DSS 3-day	3.5.4.33	tRNA(adenine(34)) deaminase
Control vs DSS 3-day	3.5.4.9	Methenyltetrahydrofolate cyclohydrolase
Control vs DSS 3-day	3.6.1.23	dUTP diphosphatase
Control vs DSS 3-day	3.6.1.27	Undecaprenyl-diphosphate phosphatase
Control vs DSS 3-day	3.6.1.7	Acylphosphatase
Control vs DSS 3-day	3.6.3.31	Polyamine-transporting ATPase
Control vs DSS 3-day	3.6.4.12	DNA helicase
Control vs DSS 3-day	3.6.5.n1	Elongation factor 4
Control vs DSS 3-day	4.1.1.11	Aspartate 1-decarboxylase
Control vs DSS 3-day	4.1.1.23	Orotidine-5'-phosphate decarboxylase
Control vs DSS 3-day	4.1.1.36	Phosphopantothenoylcysteine decarboxylase
Control vs DSS 3-day	4.1.1.37	Uroporphyrinogen decarboxylase
Control vs DSS 3-day	4.1.1.49	Phosphoenolpyruvate carboxykinase (ATP)
Control vs DSS 3-day	4.1.1.65	Phosphatidylserine decarboxylase
Control vs DSS 3-day	4.1.99.12	3,4-dihydroxy-2-butanone-4-phosphate synthase
Control vs DSS 3-day	4.1.99.22	NO_NAME
Control vs DSS 3-day	4.2.1.10	3-dehydroquinate dehydratase
Control vs DSS 3-day	4.2.1.11	Phosphopyruvate hydratase
Control vs DSS 3-day	4.2.1.136	ADP-dependent NAD(P)H-hydrate dehydratase
Control vs DSS 3-day	4.2.1.151	Chorismate dehydratase
Control vs DSS 3-day	4.2.1.19	Imidazoleglycerol-phosphate dehydratase
Control vs DSS 3-day	4.2.1.20	Tryptophan synthase
Control vs DSS 3-day	4.2.1.24	Porphobilinogen synthase

Control vs DSS 3-day	4.2.1.33	3-isopropylmalate dehydratase
Control vs DSS 3-day	4.2.1.46	dTDP-glucose 4,6-dehydratase
Control vs DSS 3-day	4.2.1.59	3-hydroxyacyl-[acyl-carrier-protein] dehydratase
Control vs DSS 3-day	4.2.1.9	Dihydroxy-acid dehydratase
Control vs DSS 3-day	4.2.3.3	Methylglyoxal synthase
Control vs DSS 3-day	4.2.3.4	3-dehydroquinate synthase
Control vs DSS 3-day	4.2.3.5	Chorismate synthase
Control vs DSS 3-day	4.2.99.18	DNA-(apurinic or apyrimidinic site) lyase
Control vs DSS 3-day	4.3.1.1	Aspartate ammonia-lyase
Control vs DSS 3-day	4.3.2.1	Argininosuccinate lyase
Control vs DSS 3-day	4.3.2.10	NO_NAME
Control vs DSS 3-day	4.3.2.2	Adenylosuccinate lyase
Control vs DSS 3-day	4.3.3.7	4-hydroxy-tetrahydrodipicolinate synthase
Control vs DSS 3-day	4.6.1.12	2-C-methyl-D-erythritol 2,4-cyclodiphosphate synthase
Control vs DSS 3-day	4.6.1.17	NO_NAME
Control vs DSS 3-day	4.99.1.1	Ferrochelatase
Control vs DSS 3-day	5.1.1.1	Alanine racemase
Control vs DSS 3-day	5.1.1.3	Glutamate racemase
Control vs DSS 3-day	5.1.1.7	Diaminopimelate epimerase
Control vs DSS 3-day	5.1.3.1	Ribulose-phosphate 3-epimerase
Control vs DSS 3-day	5.1.3.13	dTDP-4-dehydrorhamnose 3,5-epimerase
Control vs DSS 3-day	5.1.3.20	ADP-glyceromanno-heptose 6-epimerase
Control vs DSS 3-day	5.1.99.6	NAD(P)H-hydrate epimerase
Control vs DSS 3-day	5.2.1.8	Peptidylprolyl isomerase
Control vs DSS 3-day	5.3.1.1	Triose-phosphate isomerase
Control vs DSS 3-day	5.3.1.16	isomerase
Control vs DSS 3-day	5.3.1.23	S-methyl-5-thioribose-1-phosphate isomerase
Control vs DSS 3-day	5.3.1.28	D-sedoheptulose 7-phosphate isomerase
Control vs DSS 3-day	5.3.1.9	Glucose-6-phosphate isomerase
Control vs DSS 3-day	5.4.2.10	Phosphoglucosamine mutase
Control vs DSS 3-day	5.4.2.12	Phosphoglycerate mutase (2,3-diphosphoglycerate-independent)
Control vs DSS 3-day	5.4.3.8	Glutamate-1-semialdehyde 2,1-aminomutase
Control vs DSS 3-day	5.4.99.12	tRNA pseudouridine(38-40) synthase
Control vs DSS 3-day	5.4.99.18	5-(carboxyamino)imidazole ribonucleotide mutase
Control vs DSS 3-day	5.4.99.25	tRNA pseudouridine(55) synthase
Control vs DSS 3-day	5.99.1.2	DNA topoisomerase
Control vs DSS 3-day	5.99.1.3	DNA topoisomerase (ATP-hydrolyzing)
Control vs DSS 3-day	6.1.1.1	Tyrosine--tRNA ligase
Control vs DSS 3-day	6.1.1.10	Methionine--tRNA ligase
Control vs DSS 3-day	6.1.1.11	Serine--tRNA ligase
Control vs DSS 3-day	6.1.1.14	Glycine--tRNA ligase
Control vs DSS 3-day	6.1.1.15	Proline--tRNA ligase
Control vs DSS 3-day	6.1.1.16	Cysteine--tRNA ligase

Control vs DSS 3-day	6.1.1.17	Glutamate--tRNA ligase
Control vs DSS 3-day	6.1.1.19	Arginine--tRNA ligase
Control vs DSS 3-day	6.1.1.20	Phenylalanine--tRNA ligase
Control vs DSS 3-day	6.1.1.21	Histidine--tRNA ligase
Control vs DSS 3-day	6.1.1.23	Aspartate--tRNA(Asn) ligase
Control vs DSS 3-day	6.1.1.3	Threonine--tRNA ligase
Control vs DSS 3-day	6.1.1.4	Leucine--tRNA ligase
Control vs DSS 3-day	6.1.1.5	Isoleucine--tRNA ligase
Control vs DSS 3-day	6.1.1.6	Lysine--tRNA ligase
Control vs DSS 3-day	6.1.1.7	Alanine--tRNA ligase
Control vs DSS 3-day	6.1.1.9	Valine--tRNA ligase
Control vs DSS 3-day	6.3.1.5	NAD(+) synthase
Control vs DSS 3-day	6.3.2.1	Pantoate--beta-alanine ligase (AMP-forming)
Control vs DSS 3-day	6.3.2.13	UDP-N-acetylmuramoyl-L-alanyl-D-glutamate--2,6-diaminopimelate ligase
Control vs DSS 3-day	6.3.2.4	D-alanine--D-alanine ligase
Control vs DSS 3-day	6.3.2.5	Phosphopantothenate--cysteine ligase
Control vs DSS 3-day	6.3.2.6	Phosphoribosylaminoimidazolesuccinocarboxamide synthase
Control vs DSS 3-day	6.3.2.8	UDP-N-acetylmuramate--L-alanine ligase
Control vs DSS 3-day	6.3.2.9	UDP-N-acetylmuramoyl-L-alanine--D-glutamate ligase
Control vs DSS 3-day	6.3.3.1	Phosphoribosylformylglycinamidine cyclo-ligase
Control vs DSS 3-day	6.3.3.2	5-formyltetrahydrofolate cyclo-ligase
Control vs DSS 3-day	6.3.3.3	Dethiobiotin synthase
Control vs DSS 3-day	6.3.4.13	Phosphoribosylamine--glycine ligase
Control vs DSS 3-day	6.3.4.15	Biotin--[acetyl-CoA-carboxylase] ligase
Control vs DSS 3-day	6.3.4.19	tRNA(Ile)-lysidine synthetase
Control vs DSS 3-day	6.3.4.2	CTP synthase (glutamine hydrolyzing)
Control vs DSS 3-day	6.3.4.20	7-cyano-7-deazaguanine synthase
Control vs DSS 3-day	6.3.4.21	Nicotinate phosphoribosyltransferase
Control vs DSS 3-day	6.3.4.4	Adenylosuccinate synthase
Control vs DSS 3-day	6.3.4.5	Argininosuccinate synthase
Control vs DSS 3-day	6.3.5.2	GMP synthase (glutamine-hydrolyzing)
Control vs DSS 3-day	6.3.5.5	Carbamoyl-phosphate synthase (glutamine-hydrolyzing)
Control vs DSS 3-day	6.3.5.7	Glutaminyl-tRNA synthase (glutamine-hydrolyzing)
Control vs DSS 3-day	6.5.1.2	DNA ligase (NAD(+))
Control vs DSS 3-day	7.1.2.2	NO_NAME
Control vs DSS 3-day	1.7.99.1	Hydroxylamine reductase
Control vs DSS 3-day	2.1.1.37	DNA (cytosine-5-)-methyltransferase
Control vs DSS 3-day	4.1.1.49	Phosphoenolpyruvate carboxykinase (ATP)
Control vs DSS 3-day	4.2.1.11	Phosphopyruvate hydratase
Control vs DSS 3-day	5.3.1.5	Xylose isomerase
Control vs DSS 3-day	2.7.13.3	Histidine kinase
Control vs DSS 3-day	3.1.21.3	Type I site-specific deoxyribonuclease
Control vs DSS 3-day	2.7.13.3	Histidine kinase

Control vs DSS 3-day	1.1.1.25	Shikimate dehydrogenase
Control vs DSS 3-day	1.1.1.37	Malate dehydrogenase
Control vs DSS 3-day	1.1.1.91	Aryl-alcohol dehydrogenase (NADP(+))
Control vs DSS 3-day	2.1.1.72	Site-specific DNA-methyltransferase (adenine-specific)
Control vs DSS 3-day	2.3.1.51	1-acylglycerol-3-phosphate O-acyltransferase
Control vs DSS 3-day	2.6.1.52	Phosphoserine transaminase
Control vs DSS 3-day	2.7.13.3	Histidine kinase
Control vs DSS 3-day	3.1.21.3	Type I site-specific deoxyribonuclease
Control vs DSS 3-day	3.1.3.23	Sugar-phosphatase
Control vs DSS 3-day	3.1.3.25	Inositol-phosphate phosphatase
Control vs DSS 3-day	3.4.21.92	Endopeptidase Clp
Control vs DSS 3-day	3.6.4.12	DNA helicase
Control vs DSS 3-day	4.1.99.12	3,4-dihydroxy-2-butanone-4-phosphate synthase
Control vs DSS 3-day	1.1.1.37	Malate dehydrogenase
Control vs DSS 3-day	1.1.1.86	Ketol-acid reductoisomerase (NADP(+))
Control vs DSS 3-day	1.1.1.95	Phosphoglycerate dehydrogenase
Control vs DSS 3-day	1.11.1.15	Peroxiredoxin
Control vs DSS 3-day	1.11.1.6	Catalase
Control vs DSS 3-day	1.15.1.1	Superoxide dismutase
Control vs DSS 3-day	1.16.3.2	Bacterial non-heme ferritin
Control vs DSS 3-day	1.17.7.4	NO_NAME
Control vs DSS 3-day	1.2.1.12	Glyceraldehyde-3-phosphate dehydrogenase (phosphorylating)
Control vs DSS 3-day	1.3.1.9	Enoyl-[acyl-carrier-protein] reductase (NADH)
Control vs DSS 3-day	1.3.5.1	Succinate dehydrogenase (quinone)
Control vs DSS 3-day	1.3.8.1	Short-chain acyl-CoA dehydrogenase
Control vs DSS 3-day	1.5.1.3	Dihydrofolate reductase
Control vs DSS 3-day	1.5.1.5	Methylenetetrahydrofolate dehydrogenase (NADP(+))
Control vs DSS 3-day	2.1.2.1	Glycine hydroxymethyltransferase
Control vs DSS 3-day	2.3.1.129	Acyl-[acyl-carrier-protein]--UDP-N-acetylglucosamine O-acyltransferase
Control vs DSS 3-day	2.3.1.179	Beta-ketoacyl-[acyl-carrier-protein] synthase II
Control vs DSS 3-day	2.3.1.29	Glycine C-acetyltransferase
Control vs DSS 3-day	2.4.2.10	Orotate phosphoribosyltransferase
Control vs DSS 3-day	2.5.1.78	6,7-dimethyl-8-ribityllumazine synthase
Control vs DSS 3-day	2.6.1.52	Phosphoserine transaminase
Control vs DSS 3-day	2.7.1.2	Glucokinase
Control vs DSS 3-day	2.7.1.33	Pantothenate kinase
Control vs DSS 3-day	2.7.1.40	Pyruvate kinase
Control vs DSS 3-day	2.7.1.90	Diphosphate--fructose-6-phosphate 1-phosphotransferase
Control vs DSS 3-day	2.7.13.3	Histidine kinase
Control vs DSS 3-day	2.7.2.3	Phosphoglycerate kinase
Control vs DSS 3-day	2.7.4.8	Guanylate kinase
Control vs DSS 3-day	2.7.7.4	Sulfate adenylyltransferase
Control vs DSS 3-day	2.7.7.49	RNA-directed DNA polymerase

Control vs DSS 3-day	2.7.7.6	DNA-directed RNA polymerase
Control vs DSS 3-day	2.7.7.8	Polyribonucleotide nucleotidyltransferase
Control vs DSS 3-day	3.1.21.3	Type I site-specific deoxyribonuclease
Control vs DSS 3-day	3.1.3.25	Inositol-phosphate phosphatase
Control vs DSS 3-day	3.2.1.23	Beta-galactosidase
Control vs DSS 3-day	3.4.11.18	Methionyl aminopeptidase
Control vs DSS 3-day	3.4.21.92	Endopeptidase Clp
Control vs DSS 3-day	3.5.1.108	UDP-3-O-acyl-N-acetylglucosamine deacetylase
Control vs DSS 3-day	3.5.1.25	N-acetylglucosamine-6-phosphate deacetylase
Control vs DSS 3-day	3.5.4.9	Methenyltetrahydrofolate cyclohydrolase
Control vs DSS 3-day	3.5.99.6	Glucosamine-6-phosphate deaminase
Control vs DSS 3-day	4.2.1.11	Phosphopyruvate hydratase
Control vs DSS 3-day	4.2.1.2	Fumarate hydratase
Control vs DSS 3-day	4.2.1.59	3-hydroxyacyl-[acyl-carrier-protein] dehydratase
Control vs DSS 3-day	4.2.1.8	Mannonate dehydratase
Control vs DSS 3-day	5.1.3.3	Aldose 1-epimerase
Control vs DSS 3-day	5.2.1.8	Peptidylprolyl isomerase
Control vs DSS 3-day	5.3.1.1	Triose-phosphate isomerase
Control vs DSS 3-day	5.3.1.9	Glucose-6-phosphate isomerase
Control vs DSS 3-day	5.4.2.12	Phosphoglycerate mutase (2,3-diphosphoglycerate-independent)
Control vs DSS 3-day	5.99.1.2	DNA topoisomerase
Control vs DSS 3-day	5.99.1.3	DNA topoisomerase (ATP-hydrolyzing)
Control vs DSS 3-day	6.1.1.14	Glycine--tRNA ligase
Control vs DSS 3-day	6.1.1.2	Tryptophan--tRNA ligase
Control vs DSS 3-day	6.1.1.20	Phenylalanine--tRNA ligase
Control vs DSS 3-day	6.1.1.3	Threonine--tRNA ligase
Control vs DSS 3-day	6.1.1.4	Leucine--tRNA ligase
Control vs DSS 3-day	6.1.1.6	Lysine--tRNA ligase
Control vs DSS 3-day	6.3.2.6	Phosphoribosylaminoimidazolesuccinocarboxamide synthase
Control vs DSS 3-day	6.3.4.4	Adenylosuccinate synthase
Control vs DSS 3-day	6.3.5.2	GMP synthase (glutamine-hydrolyzing)
Control vs DSS 3-day	7.2.1.1	NO_NAME
Control vs DSS 3-day	1.1.1.37	Malate dehydrogenase
Control vs DSS 3-day	2.1.1.72	Site-specific DNA-methyltransferase (adenine-specific)
Control vs DSS 3-day	2.7.7.6	DNA-directed RNA polymerase
Control vs DSS 3-day	4.1.1.49	Phosphoenolpyruvate carboxykinase (ATP)
Control vs DSS 3-day	5.4.2.12	Phosphoglycerate mutase (2,3-diphosphoglycerate-independent)
Control vs DSS 3-day	1.1.1.37	Malate dehydrogenase
Control vs DSS 3-day	1.3.8.1	Short-chain acyl-CoA dehydrogenase
Control vs DSS 3-day	2.7.13.3	Histidine kinase
Control vs DSS 3-day	2.7.7.6	DNA-directed RNA polymerase
Control vs DSS 3-day	4.1.1.49	Phosphoenolpyruvate carboxykinase (ATP)
Control vs DSS 3-day	2.7.7.6	DNA-directed RNA polymerase

Control vs DSS 3-day	2.7.7.6	DNA-directed RNA polymerase
Control vs DSS 3-day	3.6.4.12	DNA helicase
Control vs DSS 3-day	3.1.21.3	Type I site-specific deoxyribonuclease
Control vs DSS 3-day	2.7.13.3	Histidine kinase
Control vs DSS 3-day	2.7.13.3	Histidine kinase
Control vs DSS 3-day	2.7.7.6	DNA-directed RNA polymerase
Control vs DSS 3-day	2.7.13.3	Histidine kinase
Control vs DSS 3-day	2.1.1.37	DNA (cytosine-5-)-methyltransferase
Control vs DSS 3-day	2.1.1.37	DNA (cytosine-5-)-methyltransferase
Control vs DSS 3-day	2.7.13.3	Histidine kinase
Control vs DSS 3-day	2.7.13.3	Histidine kinase
Control vs DSS 3-day	2.7.7.6	DNA-directed RNA polymerase
Control vs DSS 3-day	3.1.21.3	Type I site-specific deoxyribonuclease
Control vs DSS 3-day	2.7.13.3	Histidine kinase
Control vs DSS 3-day	2.7.7.6	DNA-directed RNA polymerase
Control vs DSS 3-day	2.7.7.6	DNA-directed RNA polymerase
Control vs DSS 3-day	2.7.7.6	DNA-directed RNA polymerase
Control vs DSS 3-day	2.1.1.37	DNA (cytosine-5-)-methyltransferase
Control vs DSS 3-day	2.7.9.1	Pyruvate, phosphate dikinase
Control vs DSS 3-day	2.7.9.1	Pyruvate, phosphate dikinase
Control vs DSS 3-day	4.1.1.49	Phosphoenolpyruvate carboxykinase (ATP)
Control vs DSS 3-day	2.7.9.1	Pyruvate, phosphate dikinase
Control vs DSS 3-day	3.6.3.20	Glycerol-3-phosphate-transporting ATPase
Control vs DSS 3-day	4.1.1.49	Phosphoenolpyruvate carboxykinase (ATP)
Control vs DSS 3-day	1.8.99.5	Dissimilatory sulfite reductase
Control vs DSS 3-day	2.3.1.181	Lipoyl(octanoyl) transferase
Control vs DSS 3-day	2.3.1.234	N(6)-L-threonylcarbamoyladenine synthase
Control vs DSS 3-day	2.1.2.9	Methionyl-tRNA formyltransferase
Control vs DSS 3-day	2.3.1.79	Maltose O-acetyltransferase
Control vs DSS 3-day	3.5.1.10	Formyltetrahydrofolate deformylase
Control vs DSS 3-day	2.6.99.2	Pyridoxine 5'-phosphate synthase
Control vs DSS 3-day	3.5.1.10	Formyltetrahydrofolate deformylase
Control vs DSS 3-day	2.2.1.6	Acetolactate synthase
Control vs DSS 3-day	6.1.1.7	Alanine--tRNA ligase
Control vs DSS 3-day	3.4.11.5	Prolyl aminopeptidase
Control vs DSS 3-day	3.4.19.3	Pyroglutamyl-peptidase I
Control vs DSS 3-day	1.1.1.44	Phosphogluconate dehydrogenase (NADP(+)-dependent, decarboxylating)
Control vs DSS 3-day	2.3.1.29	Glycine C-acetyltransferase
Control vs DSS 3-day	2.3.1.29	Glycine C-acetyltransferase
Control vs DSS 3-day	3.2.2.27	Uracil-DNA glycosylase
Control vs DSS 3-day	4.2.1.46	dTDP-glucose 4,6-dehydratase
Control vs DSS 3-day	5.1.3.3	Aldose 1-epimerase
Control vs DSS 3-day	1.8.1.4	Dihydrolipoyl dehydrogenase

Control vs DSS 3-day	2.3.1.179	Beta-ketoacyl-[acyl-carrier-protein] synthase II
Control vs DSS 3-day	6.1.1.4	Leucine--tRNA ligase
Control vs DSS 3-day	1.8.1.9	Thioredoxin-disulfide reductase
Control vs DSS 3-day	4.2.1.11	Phosphopyruvate hydratase
Control vs DSS 3-day	3.2.1.37	Xylan 1,4-beta-xylosidase
Control vs DSS 3-day	3.5.4.16	GTP cyclohydrolase I
Control vs DSS 3-day	2.1.1.198	16S rRNA (cytidine(1402)-2'-O)-methyltransferase
Control vs DSS 3-day	2.3.1.28	Chloramphenicol O-acetyltransferase
Control vs DSS 3-day	2.7.4.1	Polyphosphate kinase
Control vs DSS 3-day	2.7.7.60	2-C-methyl-D-erythritol 4-phosphate cytidylyltransferase
Control vs DSS 3-day	3.2.1.165	Exo-1,4-beta-D-glucosaminidase
Control vs DSS 3-day	3.2.1.25	Beta-mannosidase
Control vs DSS 3-day	5.99.1.3	DNA topoisomerase (ATP-hydrolyzing)
Control vs DSS 3-day	1.4.1.2	Glutamate dehydrogenase
Control vs DSS 3-day	2.3.1.129	Acyl-[acyl-carrier-protein]--UDP-N-acetylglucosamine O-acyltransferase
Control vs DSS 3-day	2.7.7.4	Sulfate adenylyltransferase
Control vs DSS 3-day	5.3.1.25	L-fucose isomerase
Control vs DSS 3-day	2.3.1.181	Lipoyl(octanoyl) transferase
Control vs DSS 3-day	5.3.1.9	Glucose-6-phosphate isomerase
Control vs DSS 3-day	2.4.2.10	Orotate phosphoribosyltransferase
Control vs DSS 3-day	2.7.2.4	Aspartate kinase
Control vs DSS 3-day	6.3.2.8	UDP-N-acetylmuramate--L-alanine ligase
Control vs DSS 3-day	2.5.1.78	6,7-dimethyl-8-ribityllumazine synthase
Control vs DSS 3-day	3.1.4.46	Glycerophosphodiester phosphodiesterase
Control vs DSS 3-day	1.1.1.28	D-lactate dehydrogenase
Control vs DSS 3-day	2.1.1.177	23S rRNA (pseudouridine(1915)-N(3))-methyltransferase
Control vs DSS 3-day	2.1.2.10	Aminomethyltransferase
Control vs DSS 3-day	2.7.1.71	Shikimate kinase
Control vs DSS 3-day	2.7.7.85	Diadenylate cyclase
Control vs DSS 3-day	2.1.2.1	Glycine hydroxymethyltransferase
Control vs DSS 3-day	1.1.1.271	GDP-L-fucose synthase
Control vs DSS 3-day	1.1.1.94	Glycerol-3-phosphate dehydrogenase (NAD(P)(+))
Control vs DSS 3-day	1.2.1.11	Aspartate-semialdehyde dehydrogenase
Control vs DSS 3-day	4.1.2.4	Deoxyribose-phosphate aldolase
Control vs DSS 3-day	5.3.1.12	Glucuronate isomerase
Control vs DSS 3-day	6.3.1.1	Aspartate--ammonia ligase
Control vs DSS 3-day	1.17.1.2	4-hydroxy-3-methylbut-2-enyl diphosphate reductase
Control vs DSS 3-day	2.4.1.1	Glycogen phosphorylase
Control vs DSS 3-day	4.2.1.136	ADP-dependent NAD(P)H-hydrate dehydratase
Control vs DSS 3-day	1.3.1.76	Precorrin-2 dehydrogenase
Control vs DSS 3-day	2.7.4.22	UMP kinase
Control vs DSS 3-day	5.4.2.11	Phosphoglycerate mutase (2,3-diphosphoglycerate-dependent)
Control vs DSS 3-day	1.1.1.40	Malate dehydrogenase (oxaloacetate-decarboxylating) (NADP(+))

Control vs DSS 3-day	2.7.13.3	Histidine kinase
Control vs DSS 3-day	1.16.3.2	Bacterial non-heme ferritin
Control vs DSS 3-day	2.7.7.8	Polyribonucleotide nucleotidyltransferase
Control vs DSS 3-day	3.6.5.n1	Elongation factor 4
Control vs DSS 3-day	6.1.1.3	Threonine--tRNA ligase
Control vs DSS 3-day	2.7.7.8	Polyribonucleotide nucleotidyltransferase
Control vs DSS 3-day	3.4.11.18	Methionyl aminopeptidase
Control vs DSS 3-day	3.6.5.n1	Elongation factor 4
Control vs DSS 3-day	6.1.1.3	Threonine--tRNA ligase
Control vs DSS 3-day	2.7.7.8	Polyribonucleotide nucleotidyltransferase
Control vs DSS 3-day	3.6.5.n1	Elongation factor 4
Control vs DSS 3-day	6.1.1.3	Threonine--tRNA ligase
Control vs DSS 3-day	2.7.13.3	Histidine kinase
Control vs DSS 3-day	1.1.1.58	Tagaturonate reductase
Control vs DSS 3-day	1.2.1.11	Aspartate-semialdehyde dehydrogenase
Control vs DSS 3-day	1.2.1.38	N-acetyl-gamma-glutamyl-phosphate reductase
Control vs DSS 3-day	1.3.1.1	Dihydrouracil dehydrogenase (NAD(+))
Control vs DSS 3-day	1.3.98.1	Dihydroorotate oxidase (fumarate)
Control vs DSS 3-day	1.4.1.16	Diaminopimelate dehydrogenase
Control vs DSS 3-day	2.1.3.9	N-acetylornithine carbamoyltransferase
Control vs DSS 3-day	2.4.1.18	1,4-alpha-glucan branching enzyme
Control vs DSS 3-day	2.4.2.18	Anthranilate phosphoribosyltransferase
Control vs DSS 3-day	2.5.1.75	tRNA dimethylallyltransferase
Control vs DSS 3-day	2.6.1.1	Aspartate transaminase
Control vs DSS 3-day	2.8.1.7	Cysteine desulfurase
Control vs DSS 3-day	3.1.1.11	Pectinesterase
Control vs DSS 3-day	3.1.11.5	Exodeoxyribonuclease V
Control vs DSS 3-day	3.5.1.2	Glutaminase
Control vs DSS 3-day	4.1.1.15	Glutamate decarboxylase
Control vs DSS 3-day	6.1.1.18	Glutamine--tRNA ligase
Control vs DSS 3-day	6.1.1.4	Leucine--tRNA ligase
Control vs DSS 3-day	6.2.1.30	Phenylacetate--CoA ligase
Control vs DSS 3-day	6.3.2.4	D-alanine--D-alanine ligase
Control vs DSS 3-day	6.5.1.2	DNA ligase (NAD(+))
Control vs DSS 3-day	1.1.1.193	5-amino-6-(5-phosphoribosylamino)uracil reductase
Control vs DSS 3-day	1.3.1.98	UDP-N-acetylmuramate dehydrogenase
Control vs DSS 3-day	2.1.1.197	Malonyl-[acyl-carrier protein] O-methyltransferase
Control vs DSS 3-day	2.3.1.28	Chloramphenicol O-acetyltransferase
Control vs DSS 3-day	2.4.1.227	acetylglucosaminyltransferase
Control vs DSS 3-day	2.6.1.62	Adenosylmethionine--8-amino-7-oxononanoate transaminase
Control vs DSS 3-day	2.6.1.85	Aminodeoxychorismate synthase
Control vs DSS 3-day	2.7.1.148	4-(cytidine 5'-diphospho)-2-C-methyl-D-erythritol kinase
Control vs DSS 3-day	2.7.4.1	Polyphosphate kinase

Control vs DSS 3-day	2.8.1.13	tRNA-uridine 2-sulfurtransferase
Control vs DSS 3-day	2.8.1.6	Biotin synthase
Control vs DSS 3-day	3.1.21.4	Type II site-specific deoxyribonuclease
Control vs DSS 3-day	3.1.3.7	3'(2'),5'-bisphosphate nucleotidase
Control vs DSS 3-day	3.1.4.46	Glycerophosphodiester phosphodiesterase
Control vs DSS 3-day	3.1.6.1	Arylsulfatase
Control vs DSS 3-day	3.2.1.41	Pullulanase
Control vs DSS 3-day	3.2.1.8	Endo-1,4-beta-xylanase
Control vs DSS 3-day	3.4.17.13	Muramoyltetrapeptide carboxypeptidase
Control vs DSS 3-day	3.5.4.26	Diaminohydroxyphosphoribosylaminopyrimidine deaminase
Control vs DSS 3-day	4.1.2.25	Dihydroneopterin aldolase
Control vs DSS 3-day	4.1.3.38	Aminodeoxychorismate lyase
Control vs DSS 3-day	4.6.1.12	2-C-methyl-D-erythritol 2,4-cyclodiphosphate synthase
Control vs DSS 3-day	5.3.1.24	Phosphoribosylanthranilate isomerase
Control vs DSS 3-day	1.16.3.2	Bacterial non-heme ferritin
Control vs DSS 3-day	2.3.1.79	Maltose O-acetyltransferase
Control vs DSS 3-day	3.5.1.2	Glutaminase
Control vs DSS 3-day	4.1.1.15	Glutamate decarboxylase
Control vs DSS 3-day	4.1.1.19	Arginine decarboxylase
Control vs DSS 3-day	5.2.1.8	Peptidylprolyl isomerase
Control vs DSS 3-day	5.3.1.5	Xylose isomerase
Control vs DSS 3-day	6.1.1.22	Asparagine--tRNA ligase
Control vs DSS 3-day	6.1.1.3	Threonine--tRNA ligase
Control vs DSS 3-day	6.3.4.4	Adenylosuccinate synthase
Control vs DSS 3-day	6.3.5.5	Carbamoyl-phosphate synthase (glutamine-hydrolyzing)
Control vs DSS 3-day	1.1.1.86	Ketol-acid reductoisomerase (NADP(+))
Control vs DSS 3-day	1.1.1.95	Phosphoglycerate dehydrogenase
Control vs DSS 3-day	1.17.4.1	Ribonucleoside-diphosphate reductase
Control vs DSS 3-day	1.17.4.2	Ribonucleoside-triphosphate reductase
Control vs DSS 3-day	1.2.7.8	Indolepyruvate ferredoxin oxidoreductase
Control vs DSS 3-day	1.4.1.2	Glutamate dehydrogenase
Control vs DSS 3-day	1.4.1.3	Glutamate dehydrogenase (NAD(P)(+))
Control vs DSS 3-day	2.3.1.181	Lipoyl(octanoyl) transferase
Control vs DSS 3-day	2.7.1.33	Pantothenate kinase
Control vs DSS 3-day	2.7.2.1	Acetate kinase
Control vs DSS 3-day	2.8.4.4	[Ribosomal protein S12] (aspartate(89)-C(3))-methylthiotransferase
Control vs DSS 3-day	3.4.11.4	Tripeptide aminopeptidase
Control vs DSS 3-day	3.4.13.20	Beta-Ala-His dipeptidase
Control vs DSS 3-day	3.6.4.12	DNA helicase
Control vs DSS 3-day	5.3.1.1	Triose-phosphate isomerase
Control vs DSS 3-day	6.1.1.16	Cysteine--tRNA ligase
Control vs DSS 3-day	6.1.1.3	Threonine--tRNA ligase
Control vs DSS 3-day	1.1.1.28	D-lactate dehydrogenase

Control vs DSS 3-day	1.1.1.58	Tagaturonate reductase
Control vs DSS 3-day	1.2.7.8	Indolepyruvate ferredoxin oxidoreductase
Control vs DSS 3-day	1.8.1.9	Thioredoxin-disulfide reductase
Control vs DSS 3-day	2.1.1.37	DNA (cytosine-5-)-methyltransferase
Control vs DSS 3-day	2.1.2.9	Methionyl-tRNA formyltransferase
Control vs DSS 3-day	2.4.2.17	ATP phosphoribosyltransferase
Control vs DSS 3-day	2.5.1.47	Cysteine synthase
Control vs DSS 3-day	2.6.1.83	LL-diaminopimelate aminotransferase
Control vs DSS 3-day	2.7.7.72	CCA tRNA nucleotidyltransferase
Control vs DSS 3-day	3.1.22.4	Crossover junction endodeoxyribonuclease
Control vs DSS 3-day	3.2.2.n1	Cytokinin riboside 5'-monophosphate phosphoribohydrolase
Control vs DSS 3-day	3.4.11.4	Tripeptide aminopeptidase
Control vs DSS 3-day	4.1.1.65	Phosphatidylserine decarboxylase
Control vs DSS 3-day	4.2.1.10	3-dehydroquinate dehydratase
Control vs DSS 3-day	4.2.1.3	Aconitate hydratase
Control vs DSS 3-day	4.2.1.9	Dihydroxy-acid dehydratase
Control vs DSS 3-day	6.1.1.17	Glutamate--tRNA ligase
Control vs DSS 3-day	6.5.1.2	DNA ligase (NAD(+))
Control vs DSS 3-day	1.1.1.133	dTDP-4-dehydrorhamnose reductase
Control vs DSS 3-day	1.1.1.205	IMP dehydrogenase
Control vs DSS 3-day	1.1.1.22	UDP-glucose 6-dehydrogenase
Control vs DSS 3-day	1.1.1.25	Shikimate dehydrogenase
Control vs DSS 3-day	1.1.1.262	4-hydroxythreonine-4-phosphate dehydrogenase
Control vs DSS 3-day	1.5.1.20	Methylenetetrahydrofolate reductase (NAD(P)H)
Control vs DSS 3-day	2.2.1.6	Acetolactate synthase
Control vs DSS 3-day	2.4.1.182	Lipid-A-disaccharide synthase
Control vs DSS 3-day	2.5.1.15	Dihydropteroate synthase
Control vs DSS 3-day	2.7.1.180	FAD
Control vs DSS 3-day	2.7.7.63	Lipoate--protein ligase
Control vs DSS 3-day	2.7.7.85	Diadenylate cyclase
Control vs DSS 3-day	3.1.26.11	Ribonuclease Z
Control vs DSS 3-day	3.1.26.4	Ribonuclease H
Control vs DSS 3-day	3.6.1.27	Undecaprenyl-diphosphate phosphatase
Control vs DSS 3-day	4.2.1.46	dTDP-glucose 4,6-dehydratase
Control vs DSS 3-day	4.3.1.4	Formimidoyltetrahydrofolate cyclodeaminase
Control vs DSS 3-day	5.99.1.2	DNA topoisomerase
Control vs DSS 3-day	6.1.1.15	Proline--tRNA ligase
Control vs DSS 3-day	6.1.1.20	Phenylalanine--tRNA ligase
Control vs DSS 3-day	6.1.1.22	Asparagine--tRNA ligase
Control vs DSS 3-day	6.3.3.3	Dethiobiotin synthase
Control vs DSS 3-day	5.4.2.12	Phosphoglycerate mutase (2,3-diphosphoglycerate-independent)
Control vs DSS 3-day	6.1.1.14	Glycine--tRNA ligase
Control vs DSS 3-day	1.1.1.37	Malate dehydrogenase

Control vs DSS 3-day	6.1.1.14	Glycine--tRNA ligase
Control vs DSS 3-day	1.1.1.205	IMP dehydrogenase
Control vs DSS 3-day	1.16.3.2	Bacterial non-heme ferritin
Control vs DSS 3-day	1.17.4.1	Ribonucleoside-diphosphate reductase
Control vs DSS 3-day	1.17.7.4	NO_NAME
Control vs DSS 3-day	1.2.1.38	N-acetyl-gamma-glutamyl-phosphate reductase
Control vs DSS 3-day	1.4.1.3	Glutamate dehydrogenase (NAD(P)(+))
Control vs DSS 3-day	2.1.1.198	16S rRNA (cytidine(1402)-2'-O)-methyltransferase
Control vs DSS 3-day	2.1.1.45	Thymidylate synthase
Control vs DSS 3-day	2.1.3.2	Aspartate carbamoyltransferase
Control vs DSS 3-day	2.3.1.79	Maltose O-acetyltransferase
Control vs DSS 3-day	2.4.1.1	Glycogen phosphorylase
Control vs DSS 3-day	2.4.2.1	Purine-nucleoside phosphorylase
Control vs DSS 3-day	2.4.2.10	Orotate phosphoribosyltransferase
Control vs DSS 3-day	2.7.1.11	6-phosphofructokinase
Control vs DSS 3-day	2.7.4.25	(d)CMP kinase
Control vs DSS 3-day	2.7.9.1	Pyruvate, phosphate dikinase
Control vs DSS 3-day	3.4.21.92	Endopeptidase Clp
Control vs DSS 3-day	4.1.1.19	Arginine decarboxylase
Control vs DSS 3-day	4.2.1.20	Tryptophan synthase
Control vs DSS 3-day	4.3.2.2	Adenylosuccinate lyase
Control vs DSS 3-day	5.3.1.17	5-dehydro-4-deoxy-D-glucuronate isomerase
Control vs DSS 3-day	5.4.2.11	Phosphoglycerate mutase (2,3-diphosphoglycerate-dependent)
Control vs DSS 3-day	6.1.1.1	Tyrosine--tRNA ligase
Control vs DSS 3-day	6.1.1.10	Methionine--tRNA ligase
Control vs DSS 3-day	6.1.1.12	Aspartate--tRNA ligase
Control vs DSS 3-day	6.1.1.19	Arginine--tRNA ligase
Control vs DSS 3-day	6.1.1.22	Asparagine--tRNA ligase
Control vs DSS 3-day	6.1.1.3	Threonine--tRNA ligase
Control vs DSS 3-day	6.2.1.30	Phenylacetate--CoA ligase
Control vs DSS 3-day	6.3.4.2	CTP synthase (glutamine hydrolyzing)
Control vs DSS 3-day	6.3.4.4	Adenylosuccinate synthase
Control vs DSS 3-day	6.3.5.2	GMP synthase (glutamine-hydrolyzing)
Control vs DSS 3-day	1.11.1.1	NADH peroxidase
Control vs DSS 3-day	2.7.13.3	Histidine kinase
Control vs DSS 3-day	4.1.1.49	Phosphoenolpyruvate carboxykinase (ATP)
Control vs DSS 3-day	1.4.4.2	Glycine dehydrogenase (aminomethyl-transferring)
Control vs DSS 3-day	1.6.99.5	Transferred entry
Control vs DSS 3-day	2.1.2.11	3-methyl-2-oxobutanoate hydroxymethyltransferase
Control vs DSS 3-day	2.6.1.16	Glutamine--fructose-6-phosphate transaminase (isomerizing)
Control vs DSS 3-day	2.6.99.2	Pyridoxine 5'-phosphate synthase
Control vs DSS 3-day	2.7.1.33	Pantothenate kinase
Control vs DSS 3-day	2.7.7.63	Lipoate--protein ligase

Control vs DSS 3-day	3.6.4.13	RNA helicase
Control vs DSS 3-day	4.1.1.15	Glutamate decarboxylase
Control vs DSS 3-day	4.3.1.4	Formimidoyltetrahydrofolate cyclodeaminase
Control vs DSS 3-day	6.1.1.1	Tyrosine--tRNA ligase
Control vs DSS 3-day	1.1.1.37	Malate dehydrogenase
Control vs DSS 3-day	1.17.4.1	Ribonucleoside-diphosphate reductase
Control vs DSS 3-day	1.4.1.3	Glutamate dehydrogenase (NAD(P)(+))
Control vs DSS 3-day	3.1.21.3	Type I site-specific deoxyribonuclease
Control vs DSS 3-day	3.6.4.12	DNA helicase
Control vs DSS 3-day	4.1.1.49	Phosphoenolpyruvate carboxykinase (ATP)
Control vs DSS 3-day	5.3.1.12	Glucuronate isomerase
Control vs DSS 3-day	2.7.1.11	6-phosphofructokinase
Control vs DSS 3-day	6.1.1.3	Threonine--tRNA ligase
Control vs DSS 3-day	1.4.1.3	Glutamate dehydrogenase (NAD(P)(+))
Control vs DSS 3-day	1.6.5.3	NADH
Control vs DSS 3-day	2.3.1.47	8-amino-7-oxononanoate synthase
Control vs DSS 3-day	2.4.2.14	Amidophosphoribosyltransferase
Control vs DSS 3-day	2.4.2.19	Nicotinate-nucleotide diphosphorylase (carboxylating)
Control vs DSS 3-day	3.1.21.3	Type I site-specific deoxyribonuclease
Control vs DSS 3-day	3.4.11.4	Tripeptide aminopeptidase
Control vs DSS 3-day	3.4.21.107	Peptidase Do
Control vs DSS 3-day	3.4.21.89	Signal peptidase I
Control vs DSS 3-day	4.1.3.36	1,4-dihydroxy-2-naphthoyl-CoA synthase
Control vs DSS 3-day	4.2.1.11	Phosphopyruvate hydratase
Control vs DSS 3-day	4.2.1.17	Enoyl-CoA hydratase
Control vs DSS 3-day	4.2.3.5	Chorismate synthase
Control vs DSS 3-day	5.4.99.18	5-(carboxyamino)imidazole ribonucleotide mutase
Control vs DSS 3-day	6.1.1.4	Leucine--tRNA ligase
Control vs DSS 3-day	6.1.1.5	Isoleucine--tRNA ligase
Control vs DSS 3-day	6.3.4.2	CTP synthase (glutamine hydrolyzing)
Control vs DSS 3-day	1.1.1.100	3-oxoacyl-[acyl-carrier-protein] reductase
Control vs DSS 3-day	1.1.1.271	GDP-L-fucose synthase
Control vs DSS 3-day	1.1.1.3	Homoserine dehydrogenase
Control vs DSS 3-day	1.3.1.9	Enoyl-[acyl-carrier-protein] reductase (NADH)
Control vs DSS 3-day	2.1.1.37	DNA (cytosine-5-)-methyltransferase
Control vs DSS 3-day	2.3.1.47	8-amino-7-oxononanoate synthase
Control vs DSS 3-day	2.3.1.50	Serine C-palmitoyltransferase
Control vs DSS 3-day	2.4.1.1	Glycogen phosphorylase
Control vs DSS 3-day	2.4.1.18	1,4-alpha-glucan branching enzyme
Control vs DSS 3-day	2.5.1.17	Cob(I)yrinic acid a,c-diamide adenosyltransferase
Control vs DSS 3-day	2.6.1.42	Branched-chain-amino-acid transaminase
Control vs DSS 3-day	2.7.1.90	Diphosphate--fructose-6-phosphate 1-phosphotransferase
Control vs DSS 3-day	3.4.22.8	Clostripain

Control vs DSS 3-day	3.5.1.108	UDP-3-O-acyl-N-acetylglucosamine deacetylase
Control vs DSS 3-day	3.5.4.25	GTP cyclohydrolase II
Control vs DSS 3-day	3.6.5.n1	Elongation factor 4
Control vs DSS 3-day	4.1.1.15	Glutamate decarboxylase
Control vs DSS 3-day	4.1.2.4	Deoxyribose-phosphate aldolase
Control vs DSS 3-day	4.1.99.12	3,4-dihydroxy-2-butanone-4-phosphate synthase
Control vs DSS 3-day	4.2.99.18	DNA-(apurinic or apyrimidinic site) lyase
Control vs DSS 3-day	4.3.2.1	Argininosuccinate lyase
Control vs DSS 3-day	5.3.1.1	Triose-phosphate isomerase
Control vs DSS 3-day	5.3.1.12	Glucuronate isomerase
Control vs DSS 3-day	5.4.2.12	Phosphoglycerate mutase (2,3-diphosphoglycerate-independent)
Control vs DSS 3-day	6.1.1.20	Phenylalanine--tRNA ligase
Control vs DSS 3-day	6.1.1.3	Threonine--tRNA ligase
Control vs DSS 3-day	6.3.2.6	Phosphoribosylaminoimidazolesuccinocarboxamide synthase
Control vs DSS 3-day	2.1.1.198	16S rRNA (cytidine(1402)-2'-O)-methyltransferase
Control vs DSS 3-day	2.1.2.2	Phosphoribosylglycinamide formyltransferase
Control vs DSS 3-day	2.3.1.28	Chloramphenicol O-acetyltransferase
Control vs DSS 3-day	2.5.1.3	Thiamine-phosphate diphosphorylase
Control vs DSS 3-day	2.6.1.62	Adenosylmethionine--8-amino-7-oxononanoate transaminase
Control vs DSS 3-day	2.7.1.148	4-(cytidine 5'-diphospho)-2-C-methyl-D-erythritol kinase
Control vs DSS 3-day	2.7.1.21	Thymidine kinase
Control vs DSS 3-day	2.7.4.1	Polyphosphate kinase
Control vs DSS 3-day	2.7.4.8	Guanylate kinase
Control vs DSS 3-day	2.7.6.2	Thiamine diphosphokinase
Control vs DSS 3-day	2.7.6.3	2-amino-4-hydroxy-6-hydroxymethyldihydropteridine diphosphokinase
Control vs DSS 3-day	2.7.8.13	Phospho-N-acetylmuramoyl-pentapeptide-transferase
Control vs DSS 3-day	2.8.1.6	Biotin synthase
Control vs DSS 3-day	3.1.1.31	6-phosphogluconolactonase
Control vs DSS 3-day	3.1.1.96	D-aminoacyl-tRNA deacylase
Control vs DSS 3-day	3.1.26.11	Ribonuclease Z
Control vs DSS 3-day	3.1.26.4	Ribonuclease H
Control vs DSS 3-day	3.1.3.1	Alkaline phosphatase
Control vs DSS 3-day	3.3.1.1	Adenosylhomocysteinase
Control vs DSS 3-day	3.4.17.13	Muramoyltetrapeptide carboxypeptidase
Control vs DSS 3-day	3.6.4.13	RNA helicase
Control vs DSS 3-day	4.2.1.136	ADP-dependent NAD(P)H-hydrate dehydratase
Control vs DSS 3-day	4.2.3.3	Methylglyoxal synthase
Control vs DSS 3-day	5.1.3.13	dTDP-4-dehydrorhamnose 3,5-epimerase
Control vs DSS 3-day	5.1.99.6	NAD(P)H-hydrate epimerase
Control vs DSS 3-day	6.3.2.1	Pantoate--beta-alanine ligase (AMP-forming)
Control vs DSS 3-day	1.11.1.1	NADH peroxidase
Control vs DSS 3-day	2.7.9.1	Pyruvate, phosphate dikinase
Control vs DSS 3-day	2.7.13.3	Histidine kinase

Control vs DSS 3-day	2.7.9.1	Pyruvate, phosphate dikinase
Control vs DSS 3-day	4.1.1.49	Phosphoenolpyruvate carboxykinase (ATP)
Control vs DSS 3-day	2.1.1.198	16S rRNA (cytidine(1402)-2'-O)-methyltransferase
Control vs DSS 3-day	2.4.1.1	Glycogen phosphorylase
Control vs DSS 3-day	2.4.1.18	1,4-alpha-glucan branching enzyme
Control vs DSS 3-day	2.4.2.10	Orotate phosphoribosyltransferase
Control vs DSS 3-day	2.4.2.29	tRNA-guanine(34) transglycosylase
Control vs DSS 3-day	2.5.1.17	Cob(I)yrinic acid a,c-diamide adenosyltransferase
Control vs DSS 3-day	2.7.1.11	6-phosphofructokinase
Control vs DSS 3-day	2.7.1.40	Pyruvate kinase
Control vs DSS 3-day	2.7.9.1	Pyruvate, phosphate dikinase
Control vs DSS 3-day	3.5.1.2	Glutaminase
Control vs DSS 3-day	3.5.1.24	Choloylglycine hydrolase
Control vs DSS 3-day	4.2.99.18	DNA-(apurinic or apyrimidinic site) lyase
Control vs DSS 3-day	5.3.1.1	Triose-phosphate isomerase
Control vs DSS 3-day	5.3.1.9	Glucose-6-phosphate isomerase
Control vs DSS 3-day	6.3.2.6	Phosphoribosylaminoimidazolesuccinocarboxamide synthase
Control vs DSS 3-day	6.3.4.4	Adenylosuccinate synthase
Control vs DSS 3-day	2.7.13.3	Histidine kinase
Control vs DSS 3-day	3.1.21.3	Type I site-specific deoxyribonuclease
Control vs DSS 3-day	2.7.9.1	Pyruvate, phosphate dikinase
Control vs DSS 3-day	4.1.1.49	Phosphoenolpyruvate carboxykinase (ATP)
Control vs DSS 3-day	2.7.13.3	Histidine kinase
Control vs DSS 3-day	2.7.9.1	Pyruvate, phosphate dikinase
Control vs DSS 3-day	2.7.13.3	Histidine kinase
Control vs DSS 3-day	2.7.9.1	Pyruvate, phosphate dikinase
Control vs DSS 3-day	2.7.13.3	Histidine kinase
Control vs DSS 3-day	2.4.1.1	Glycogen phosphorylase
Control vs DSS 3-day	2.7.6.1	Ribose-phosphate diphosphokinase
Control vs DSS 3-day	3.5.1.88	Peptide deformylase
Control vs DSS 3-day	4.2.1.46	dTDP-glucose 4,6-dehydratase
Control vs DSS 3-day	5.1.3.1	Ribulose-phosphate 3-epimerase
Control vs DSS 3-day	5.1.3.13	dTDP-4-dehydrorhamnose 3,5-epimerase
Control vs DSS 3-day	5.3.1.8	Mannose-6-phosphate isomerase
Control vs DSS 3-day	5.4.2.12	Phosphoglycerate mutase (2,3-diphosphoglycerate-independent)
Control vs DSS 3-day	5.99.1.3	DNA topoisomerase (ATP-hydrolyzing)
Control vs DSS 3-day	6.1.1.2	Tryptophan--tRNA ligase
Control vs DSS 3-day	2.1.1.72	Site-specific DNA-methyltransferase (adenine-specific)
Control vs DSS 3-day	3.1.21.3	Type I site-specific deoxyribonuclease
Control vs DSS 3-day	2.7.1.130	Tetraacyldisaccharide 4'-kinase
Control vs DSS 3-day	2.7.1.40	Pyruvate kinase
Control vs DSS 3-day	2.7.13.3	Histidine kinase
Control vs DSS 3-day	2.8.1.13	tRNA-uridine 2-sulfurtransferase

Control vs DSS 3-day	2.7.2.1	Acetate kinase
Control vs DSS 3-day	3.2.1.86	6-phospho-beta-glucosidase
Control vs DSS 3-day	7.1.2.2	NO_NAME
Control vs DSS 3-day	1.2.1.11	Aspartate-semialdehyde dehydrogenase
Control vs DSS 3-day	1.5.1.3	Dihydrofolate reductase
Control vs DSS 3-day	1.5.1.5	Methylenetetrahydrofolate dehydrogenase (NADP(+))
Control vs DSS 3-day	1.8.4.11	Peptide-methionine (S)-S-oxide reductase
Control vs DSS 3-day	2.1.1.198	16S rRNA (cytidine(1402)-2'-O)-methyltransferase
Control vs DSS 3-day	2.1.2.9	Methionyl-tRNA formyltransferase
Control vs DSS 3-day	2.2.1.7	1-deoxy-D-xylulose-5-phosphate synthase
Control vs DSS 3-day	2.3.1.234	N(6)-L-threonylcarbamoyladenine synthase
Control vs DSS 3-day	2.3.1.274	NO_NAME
Control vs DSS 3-day	2.3.1.9	Acetyl-CoA C-acetyltransferase
Control vs DSS 3-day	2.4.2.22	Xanthine phosphoribosyltransferase
Control vs DSS 3-day	2.4.2.7	Adenine phosphoribosyltransferase
Control vs DSS 3-day	2.5.1.10	(2E,6E)-farnesyl diphosphate synthase
Control vs DSS 3-day	2.7.1.23	NAD(+) kinase
Control vs DSS 3-day	2.7.6.2	Thiamine diphosphokinase
Control vs DSS 3-day	2.7.6.5	GTP diphosphokinase
Control vs DSS 3-day	2.7.7.24	Glucose-1-phosphate thymidylyltransferase
Control vs DSS 3-day	3.1.11.6	Exodeoxyribonuclease VII
Control vs DSS 3-day	3.1.22.4	Crossover junction endodeoxyribonuclease
Control vs DSS 3-day	3.1.26.3	Ribonuclease III
Control vs DSS 3-day	3.1.3.1	Alkaline phosphatase
Control vs DSS 3-day	3.1.4.46	Glycerophosphodiester phosphodiesterase
Control vs DSS 3-day	3.2.1.70	Glucan 1,6-alpha-glucosidase
Control vs DSS 3-day	3.2.1.85	6-phospho-beta-galactosidase
Control vs DSS 3-day	3.2.2.20	DNA-3-methyladenine glycosylase I
Control vs DSS 3-day	3.5.4.9	Methenyltetrahydrofolate cyclohydrolase
Control vs DSS 3-day	3.6.1.13	ADP-ribose diphosphatase
Control vs DSS 3-day	3.6.1.7	Acylphosphatase
Control vs DSS 3-day	4.1.1.33	Diphosphomevalonate decarboxylase
Control vs DSS 3-day	4.1.1.36	Phosphopantothenoylcysteine decarboxylase
Control vs DSS 3-day	4.1.1.8	Oxalyl-CoA decarboxylase
Control vs DSS 3-day	4.2.1.46	dTDP-glucose 4,6-dehydratase
Control vs DSS 3-day	5.1.1.3	Glutamate racemase
Control vs DSS 3-day	5.1.1.7	Diaminopimelate epimerase
Control vs DSS 3-day	5.1.3.13	dTDP-4-dehydrorhamnose 3,5-epimerase
Control vs DSS 3-day	5.4.99.62	D-ribose pyranase
Control vs DSS 3-day	5.99.1.2	DNA topoisomerase
Control vs DSS 3-day	6.1.1.21	Histidine--tRNA ligase
Control vs DSS 3-day	6.3.2.5	Phosphopantothenate--cysteine ligase
Control vs DSS 3-day	6.3.2.8	UDP-N-acetylmuramate--L-alanine ligase

Control vs DSS 3-day	6.3.3.2	5-formyltetrahydrofolate cyclo-ligase
Control vs DSS 3-day	6.3.4.19	tRNA(Ile)-lysidine synthetase
Control vs DSS 3-day	6.3.4.3	Formate--tetrahydrofolate ligase
Control vs DSS 3-day	7.3.2.2	NO_NAME
Control vs DSS 3-day	3.1.13.1	Exoribonuclease II
Control vs DSS 3-day	2.2.1.7	1-deoxy-D-xylulose-5-phosphate synthase
Control vs DSS 3-day	3.2.1.26	Beta-fructofuranosidase
Control vs DSS 3-day	3.4.11.5	Prolyl aminopeptidase
Control vs DSS 3-day	4.2.1.136	ADP-dependent NAD(P)H-hydrate dehydratase
Control vs DSS 3-day	4.2.1.46	dTDP-glucose 4,6-dehydratase
Control vs DSS 3-day	5.4.2.6	Beta-phosphoglucomutase
Control vs DSS 3-day	6.1.1.7	Alanine--tRNA ligase
Control vs DSS 3-day	4.1.1.49	Phosphoenolpyruvate carboxykinase (ATP)
Control vs DSS 3-day	1.17.99.6	Epoxyqueuosine reductase
Control vs DSS 3-day	1.2.1.41	Glutamate-5-semialdehyde dehydrogenase
Control vs DSS 3-day	2.1.1.166	23S rRNA (uridine(2552)-2'-O)-methyltransferase
Control vs DSS 3-day	2.1.1.207	tRNA (cytidine(34)-2'-O)-methyltransferase
Control vs DSS 3-day	2.1.1.74	(FADH(2)-oxidizing)
Control vs DSS 3-day	2.3.1.30	Serine O-acetyltransferase
Control vs DSS 3-day	2.4.2.8	Hypoxanthine phosphoribosyltransferase
Control vs DSS 3-day	2.5.1.3	Thiamine-phosphate diphosphorylase
Control vs DSS 3-day	2.7.1.148	4-(cytidine 5'-diphospho)-2-C-methyl-D-erythritol kinase
Control vs DSS 3-day	2.7.1.180	FAD
Control vs DSS 3-day	2.7.1.71	Shikimate kinase
Control vs DSS 3-day	2.7.2.11	Glutamate 5-kinase
Control vs DSS 3-day	2.7.4.16	Thiamine-phosphate kinase
Control vs DSS 3-day	2.7.4.8	Guanylate kinase
Control vs DSS 3-day	3.1.21.3	Type I site-specific deoxyribonuclease
Control vs DSS 3-day	3.1.22.4	Crossover junction endodeoxyribonuclease
Control vs DSS 3-day	3.6.1.31	Phosphoribosyl-ATP diphosphatase
Control vs DSS 3-day	1.1.1.100	3-oxoacyl-[acyl-carrier-protein] reductase
Control vs DSS 3-day	1.1.1.205	IMP dehydrogenase
Control vs DSS 3-day	1.1.1.22	UDP-glucose 6-dehydrogenase
Control vs DSS 3-day	1.16.3.1	Ferroxidase
Control vs DSS 3-day	1.2.1.11	Aspartate-semialdehyde dehydrogenase
Control vs DSS 3-day	1.8.1.4	Dihydrolipoyl dehydrogenase
Control vs DSS 3-day	2.1.1.163	Demethylmenaquinone methyltransferase
Control vs DSS 3-day	2.1.1.45	Thymidylate synthase
Control vs DSS 3-day	2.1.2.10	Aminomethyltransferase
Control vs DSS 3-day	2.1.2.3	Phosphoribosylaminoimidazolecarboxamide formyltransferase
Control vs DSS 3-day	2.1.3.2	Aspartate carbamoyltransferase
Control vs DSS 3-day	2.3.1.180	Beta-ketoacyl-[acyl-carrier-protein] synthase III
Control vs DSS 3-day	2.4.2.19	Nicotinate-nucleotide diphosphorylase (carboxylating)

Control vs DSS 3-day	2.4.2.8	Hypoxanthine phosphoribosyltransferase
Control vs DSS 3-day	2.4.99.17	S-adenosylmethionine
Control vs DSS 3-day	2.5.1.6	Methionine adenosyltransferase
Control vs DSS 3-day	2.5.1.9	Riboflavin synthase
Control vs DSS 3-day	2.7.1.11	6-phosphofructokinase
Control vs DSS 3-day	2.7.2.4	Aspartate kinase
Control vs DSS 3-day	2.7.4.16	Thiamine-phosphate kinase
Control vs DSS 3-day	2.7.4.3	Adenylate kinase
Control vs DSS 3-day	2.7.9.1	Pyruvate, phosphate dikinase
Control vs DSS 3-day	3.1.26.5	Ribonuclease P
Control vs DSS 3-day	3.1.3.7	3'(2'),5'-bisphosphate nucleotidase
Control vs DSS 3-day	3.3.1.1	Adenosylhomocysteinase
Control vs DSS 3-day	3.5.4.10	IMP cyclohydrolase
Control vs DSS 3-day	4.1.1.23	Orotidine-5'-phosphate decarboxylase
Control vs DSS 3-day	4.2.1.10	3-dehydroquinate dehydratase
Control vs DSS 3-day	4.3.2.2	Adenylosuccinate lyase
Control vs DSS 3-day	4.3.3.7	4-hydroxy-tetrahydrodipicolinate synthase
Control vs DSS 3-day	5.1.3.2	UDP-glucose 4-epimerase
Control vs DSS 3-day	5.1.99.1	Methylmalonyl-CoA epimerase
Control vs DSS 3-day	5.3.1.5	Xylose isomerase
Control vs DSS 3-day	5.4.99.18	5-(carboxyamino)imidazole ribonucleotide mutase
Control vs DSS 3-day	6.1.1.1	Tyrosine--tRNA ligase
Control vs DSS 3-day	6.1.1.15	Proline--tRNA ligase
Control vs DSS 3-day	6.1.1.19	Arginine--tRNA ligase
Control vs DSS 3-day	6.1.1.22	Asparagine--tRNA ligase
Control vs DSS 3-day	6.3.2.4	D-alanine--D-alanine ligase
Control vs DSS 3-day	1.2.1.12	Glyceraldehyde-3-phosphate dehydrogenase (phosphorylating)
Control vs DSS 3-day	3.1.21.3	Type I site-specific deoxyribonuclease
Control vs DSS 3-day	6.1.1.3	Threonine--tRNA ligase
Control vs DSS 3-day	2.4.1.1	Glycogen phosphorylase
Control vs DSS 3-day	2.7.6.1	Ribose-phosphate diphosphokinase
Control vs DSS 3-day	2.7.7.6	DNA-directed RNA polymerase
Control vs DSS 3-day	2.7.7.6	DNA-directed RNA polymerase
Control vs DSS 3-day	2.7.7.6	DNA-directed RNA polymerase
Control vs DSS 3-day	2.7.13.3	Histidine kinase
Control vs DSS 3-day	2.7.9.1	Pyruvate, phosphate dikinase
Control vs DSS 6-day	1.1.1.40	Malate dehydrogenase (oxaloacetate-decarboxylating) (NADP(+))
Control vs DSS 6-day	2.1.1.72	Site-specific DNA-methyltransferase (adenine-specific)
Control vs DSS 6-day	2.7.13.3	Histidine kinase
Control vs DSS 6-day	2.1.3.3	Ornithine carbamoyltransferase
Control vs DSS 6-day	2.1.4.1	Glycine amidinotransferase
Control vs DSS 6-day	3.5.3.6	Arginine deiminase
Control vs DSS 6-day	1.16.3.2	Bacterial non-heme ferritin

Control vs DSS 6-day	3.1.21.3	Type I site-specific deoxyribonuclease
Control vs DSS 6-day	1.11.1.1	NADH peroxidase
Control vs DSS 6-day	1.3.5.1	Succinate dehydrogenase (quinone)
Control vs DSS 6-day	1.3.99.1	Deleted entry
Control vs DSS 6-day	2.3.1.29	Glycine C-acetyltransferase
Control vs DSS 6-day	2.7.7.6	DNA-directed RNA polymerase
Control vs DSS 6-day	2.7.7.8	Polyribonucleotide nucleotidyltransferase
Control vs DSS 6-day	2.7.9.1	Pyruvate, phosphate dikinase
Control vs DSS 6-day	3.4.11.18	Methionyl aminopeptidase
Control vs DSS 6-day	3.6.5.n1	Elongation factor 4
Control vs DSS 6-day	4.1.1.41	Methylmalonyl-CoA decarboxylase
Control vs DSS 6-day	4.1.1.49	Phosphoenolpyruvate carboxykinase (ATP)
Control vs DSS 6-day	4.1.99.1	Tryptophanase
Control vs DSS 6-day	4.1.99.2	Tyrosine phenol-lyase
Control vs DSS 6-day	5.4.99.2	Methylmalonyl-CoA mutase
Control vs DSS 6-day	6.1.1.3	Threonine--tRNA ligase
Control vs DSS 6-day	6.4.1.3	Propionyl-CoA carboxylase
Control vs DSS 6-day	1.11.1.1	NADH peroxidase
Control vs DSS 6-day	1.3.5.1	Succinate dehydrogenase (quinone)
Control vs DSS 6-day	1.3.99.1	Deleted entry
Control vs DSS 6-day	2.3.1.29	Glycine C-acetyltransferase
Control vs DSS 6-day	2.7.13.3	Histidine kinase
Control vs DSS 6-day	2.7.7.6	DNA-directed RNA polymerase
Control vs DSS 6-day	2.7.7.8	Polyribonucleotide nucleotidyltransferase
Control vs DSS 6-day	2.7.9.1	Pyruvate, phosphate dikinase
Control vs DSS 6-day	3.4.11.18	Methionyl aminopeptidase
Control vs DSS 6-day	3.6.5.n1	Elongation factor 4
Control vs DSS 6-day	4.1.1.41	Methylmalonyl-CoA decarboxylase
Control vs DSS 6-day	4.1.1.49	Phosphoenolpyruvate carboxykinase (ATP)
Control vs DSS 6-day	4.1.99.1	Tryptophanase
Control vs DSS 6-day	4.1.99.2	Tyrosine phenol-lyase
Control vs DSS 6-day	5.4.99.2	Methylmalonyl-CoA mutase
Control vs DSS 6-day	6.1.1.3	Threonine--tRNA ligase
Control vs DSS 6-day	6.4.1.3	Propionyl-CoA carboxylase
Control vs DSS 6-day	1.11.1.1	NADH peroxidase
Control vs DSS 6-day	1.3.5.1	Succinate dehydrogenase (quinone)
Control vs DSS 6-day	1.3.99.1	Deleted entry
Control vs DSS 6-day	2.7.13.3	Histidine kinase
Control vs DSS 6-day	2.7.7.6	DNA-directed RNA polymerase
Control vs DSS 6-day	2.7.7.8	Polyribonucleotide nucleotidyltransferase
Control vs DSS 6-day	2.7.9.1	Pyruvate, phosphate dikinase
Control vs DSS 6-day	3.4.11.18	Methionyl aminopeptidase
Control vs DSS 6-day	3.6.5.n1	Elongation factor 4

Control vs DSS 6-day	4.1.1.41	Methylmalonyl-CoA decarboxylase
Control vs DSS 6-day	4.1.1.49	Phosphoenolpyruvate carboxykinase (ATP)
Control vs DSS 6-day	4.1.99.1	Tryptophanase
Control vs DSS 6-day	4.1.99.2	Tyrosine phenol-lyase
Control vs DSS 6-day	5.4.99.2	Methylmalonyl-CoA mutase
Control vs DSS 6-day	6.1.1.3	Threonine--tRNA ligase
Control vs DSS 6-day	6.4.1.3	Propionyl-CoA carboxylase
Control vs DSS 6-day	2.7.13.3	Histidine kinase
Control vs DSS 6-day	2.7.7.49	RNA-directed DNA polymerase
Control vs DSS 6-day	1.1.1.205	IMP dehydrogenase
Control vs DSS 6-day	1.1.1.23	Histidinol dehydrogenase
Control vs DSS 6-day	1.1.1.262	4-hydroxythreonine-4-phosphate dehydrogenase
Control vs DSS 6-day	1.1.1.290	4-phosphoerythronate dehydrogenase
Control vs DSS 6-day	1.1.1.37	Malate dehydrogenase
Control vs DSS 6-day	1.1.1.58	Tagaturonate reductase
Control vs DSS 6-day	1.1.1.86	Ketol-acid reductoisomerase (NADP(+))
Control vs DSS 6-day	1.1.1.94	Glycerol-3-phosphate dehydrogenase (NAD(P)(+))
Control vs DSS 6-day	1.1.1.95	Phosphoglycerate dehydrogenase
Control vs DSS 6-day	1.11.1.1	NADH peroxidase
Control vs DSS 6-day	1.11.1.15	Peroxiredoxin
Control vs DSS 6-day	1.15.1.1	Superoxide dismutase
Control vs DSS 6-day	1.17.4.1	Ribonucleoside-diphosphate reductase
Control vs DSS 6-day	1.17.4.2	Ribonucleoside-triphosphate reductase
Control vs DSS 6-day	1.2.1.11	Aspartate-semialdehyde dehydrogenase
Control vs DSS 6-day	1.2.1.38	N-acetyl-gamma-glutamyl-phosphate reductase
Control vs DSS 6-day	1.3.1.1	Dihydrouracil dehydrogenase (NAD(+))
Control vs DSS 6-day	1.3.1.14	Dihydroorotate dehydrogenase (NAD(+))
Control vs DSS 6-day	1.3.1.9	Enoyl-[acyl-carrier-protein] reductase (NADH)
Control vs DSS 6-day	1.3.98.1	Dihydroorotate oxidase (fumarate)
Control vs DSS 6-day	1.4.1.16	Diaminopimelate dehydrogenase
Control vs DSS 6-day	1.4.1.2	Glutamate dehydrogenase
Control vs DSS 6-day	1.4.1.3	Glutamate dehydrogenase (NAD(P)(+))
Control vs DSS 6-day	1.4.3.16	L-aspartate oxidase
Control vs DSS 6-day	1.5.1.5	Methylenetetrahydrofolate dehydrogenase (NADP(+))
Control vs DSS 6-day	1.6.5.11	NADH dehydrogenase (quinone)
Control vs DSS 6-day	1.6.5.3	NADH
Control vs DSS 6-day	1.7.1.13	PreQ(1) synthase
Control vs DSS 6-day	1.8.1.4	Dihydrolipoyl dehydrogenase
Control vs DSS 6-day	1.8.1.9	Thioredoxin-disulfide reductase
Control vs DSS 6-day	1.97.1.4	[Formate-C-acetyltransferase]-activating enzyme
Control vs DSS 6-day	2.1.1.163	Demethylmenaquinone methyltransferase
Control vs DSS 6-day	2.1.1.191	23S rRNA (cytosine(1962)-C(5))-methyltransferase
Control vs DSS 6-day	2.1.1.192	23S rRNA (adenine(2503)-C(2))-methyltransferase

Control vs DSS 6-day	2.1.1.63	Methylated-DNA--[protein]-cysteine S-methyltransferase
Control vs DSS 6-day	2.1.1.72	Site-specific DNA-methyltransferase (adenine-specific)
Control vs DSS 6-day	2.1.2.1	Glycine hydroxymethyltransferase
Control vs DSS 6-day	2.1.2.3	Phosphoribosylaminoimidazolecarboxamide formyltransferase
Control vs DSS 6-day	2.1.3.2	Aspartate carbamoyltransferase
Control vs DSS 6-day	2.1.3.9	N-acetylornithine carbamoyltransferase
Control vs DSS 6-day	2.3.1.129	Acyl-[acyl-carrier-protein]--UDP-N-acetylglucosamine O-acyltransferase
Control vs DSS 6-day	2.3.1.179	Beta-ketoacyl-[acyl-carrier-protein] synthase II
Control vs DSS 6-day	2.3.1.180	Beta-ketoacyl-[acyl-carrier-protein] synthase III
Control vs DSS 6-day	2.3.1.181	Lipoyl(octanoyl) transferase
Control vs DSS 6-day	2.3.1.29	Glycine C-acetyltransferase
Control vs DSS 6-day	2.3.1.47	8-amino-7-oxononanoate synthase
Control vs DSS 6-day	2.3.3.13	2-isopropylmalate synthase
Control vs DSS 6-day	2.4.1.18	1,4-alpha-glucan branching enzyme
Control vs DSS 6-day	2.4.1.21	Starch synthase
Control vs DSS 6-day	2.4.2.1	Purine-nucleoside phosphorylase
Control vs DSS 6-day	2.4.2.10	Orotate phosphoribosyltransferase
Control vs DSS 6-day	2.4.2.14	Amidophosphoribosyltransferase
Control vs DSS 6-day	2.4.2.17	ATP phosphoribosyltransferase
Control vs DSS 6-day	2.4.2.18	Anthranilate phosphoribosyltransferase
Control vs DSS 6-day	2.4.2.19	Nicotinate-nucleotide diphosphorylase (carboxylating)
Control vs DSS 6-day	2.5.1.75	tRNA dimethylallyltransferase
Control vs DSS 6-day	2.5.1.78	6,7-dimethyl-8-ribityllumazine synthase
Control vs DSS 6-day	2.6.1.1	Aspartate transaminase
Control vs DSS 6-day	2.6.1.42	Branched-chain-amino-acid transaminase
Control vs DSS 6-day	2.6.1.52	Phosphoserine transaminase
Control vs DSS 6-day	2.6.1.83	LL-diaminopimelate aminotransferase
Control vs DSS 6-day	2.6.1.9	Histidinol-phosphate transaminase
Control vs DSS 6-day	2.6.99.2	Pyridoxine 5'-phosphate synthase
Control vs DSS 6-day	2.7.1.40	Pyruvate kinase
Control vs DSS 6-day	2.7.1.48	Uridine kinase
Control vs DSS 6-day	2.7.1.69	Protein-N(pi)-phosphohistidine--sugar phosphotransferase
Control vs DSS 6-day	2.7.1.90	Diphosphate--fructose-6-phosphate 1-phosphotransferase
Control vs DSS 6-day	2.7.13.3	Histidine kinase
Control vs DSS 6-day	2.7.2.1	Acetate kinase
Control vs DSS 6-day	2.7.2.3	Phosphoglycerate kinase
Control vs DSS 6-day	2.7.2.4	Aspartate kinase
Control vs DSS 6-day	2.7.4.6	Nucleoside-diphosphate kinase
Control vs DSS 6-day	2.7.6.1	Ribose-phosphate diphosphokinase
Control vs DSS 6-day	2.7.7.6	DNA-directed RNA polymerase
Control vs DSS 6-day	2.7.7.7	DNA-directed DNA polymerase
Control vs DSS 6-day	2.7.7.72	CCA tRNA nucleotidyltransferase
Control vs DSS 6-day	2.7.7.8	Polyribonucleotide nucleotidyltransferase

Control vs DSS 6-day	2.7.8.13	Phospho-N-acetylmuramoyl-pentapeptide-transferase
Control vs DSS 6-day	2.8.1.7	Cysteine desulfurase
Control vs DSS 6-day	2.8.4.3	tRNA-2-methylthio-N(6)-dimethylallyladenosine synthase
Control vs DSS 6-day	3.1.1.11	Pectinesterase
Control vs DSS 6-day	3.1.1.29	Aminoacyl-tRNA hydrolase
Control vs DSS 6-day	3.1.1.96	D-aminoacyl-tRNA deacylase
Control vs DSS 6-day	3.1.11.5	Exodeoxyribonuclease V
Control vs DSS 6-day	3.1.21.3	Type I site-specific deoxyribonuclease
Control vs DSS 6-day	3.1.26.5	Ribonuclease P
Control vs DSS 6-day	3.1.3.11	Fructose-bisphosphatase
Control vs DSS 6-day	3.1.3.15	Histidinol-phosphatase
Control vs DSS 6-day	3.2.1.135	Neopullulanase
Control vs DSS 6-day	3.2.1.21	Beta-glucosidase
Control vs DSS 6-day	3.2.1.22	Alpha-galactosidase
Control vs DSS 6-day	3.2.1.23	Beta-galactosidase
Control vs DSS 6-day	3.2.1.51	Alpha-L-fucosidase
Control vs DSS 6-day	3.2.2.27	Uracil-DNA glycosylase
Control vs DSS 6-day	3.3.1.1	Adenosylhomocysteinase
Control vs DSS 6-day	3.4.11.18	Methionyl aminopeptidase
Control vs DSS 6-day	3.4.11.9	Xaa-Pro aminopeptidase
Control vs DSS 6-day	3.4.13.18	Cytosol nonspecific dipeptidase
Control vs DSS 6-day	3.4.14.12	Xaa-Xaa-Pro tripeptidyl-peptidase
Control vs DSS 6-day	3.4.14.4	Dipeptidyl-peptidase III
Control vs DSS 6-day	3.4.21.53	Endopeptidase La
Control vs DSS 6-day	3.4.21.92	Endopeptidase Clp
Control vs DSS 6-day	3.5.1.10	Formyltetrahydrofolate deformylase
Control vs DSS 6-day	3.5.1.108	UDP-3-O-acyl-N-acetylglucosamine deacetylase
Control vs DSS 6-day	3.5.1.2	Glutaminase
Control vs DSS 6-day	3.5.1.28	N-acetylmuramoyl-L-alanine amidase
Control vs DSS 6-day	3.5.1.88	Peptide deformylase
Control vs DSS 6-day	3.5.4.10	IMP cyclohydrolase
Control vs DSS 6-day	3.5.4.16	GTP cyclohydrolase I
Control vs DSS 6-day	3.5.4.9	Methenyltetrahydrofolate cyclohydrolase
Control vs DSS 6-day	3.5.99.6	Glucosamine-6-phosphate deaminase
Control vs DSS 6-day	3.6.3.31	Polyamine-transporting ATPase
Control vs DSS 6-day	3.6.4.12	DNA helicase
Control vs DSS 6-day	3.6.5.n1	Elongation factor 4
Control vs DSS 6-day	4.1.1.15	Glutamate decarboxylase
Control vs DSS 6-day	4.1.1.19	Arginine decarboxylase
Control vs DSS 6-day	4.1.1.23	Orotidine-5'-phosphate decarboxylase
Control vs DSS 6-day	4.1.1.36	Phosphopantothenoylcysteine decarboxylase
Control vs DSS 6-day	4.1.1.49	Phosphoenolpyruvate carboxykinase (ATP)
Control vs DSS 6-day	4.1.2.4	Deoxyribose-phosphate aldolase

Control vs DSS 6-day	4.1.3.36	1,4-dihydroxy-2-naphthoyl-CoA synthase
Control vs DSS 6-day	4.2.1.11	Phosphopyruvate hydratase
Control vs DSS 6-day	4.2.1.17	Enoyl-CoA hydratase
Control vs DSS 6-day	4.2.1.19	Imidazoleglycerol-phosphate dehydratase
Control vs DSS 6-day	4.2.1.2	Fumarate hydratase
Control vs DSS 6-day	4.2.1.33	3-isopropylmalate dehydratase
Control vs DSS 6-day	4.2.1.46	dTDP-glucose 4,6-dehydratase
Control vs DSS 6-day	4.2.1.47	GDP-mannose 4,6-dehydratase
Control vs DSS 6-day	4.2.1.59	3-hydroxyacyl-[acyl-carrier-protein] dehydratase
Control vs DSS 6-day	4.2.1.8	Mannonate dehydratase
Control vs DSS 6-day	4.2.3.5	Chorismate synthase
Control vs DSS 6-day	4.3.3.7	4-hydroxy-tetrahydrodipicolinate synthase
Control vs DSS 6-day	5.1.1.7	Diaminopimelate epimerase
Control vs DSS 6-day	5.1.3.1	Ribulose-phosphate 3-epimerase
Control vs DSS 6-day	5.1.3.3	Aldose 1-epimerase
Control vs DSS 6-day	5.2.1.8	Peptidylprolyl isomerase
Control vs DSS 6-day	5.3.1.1	Triose-phosphate isomerase
Control vs DSS 6-day	5.3.1.12	Glucuronate isomerase
Control vs DSS 6-day	5.3.1.17	5-dehydro-4-deoxy-D-glucuronate isomerase
Control vs DSS 6-day	5.3.1.25	L-fucose isomerase
Control vs DSS 6-day	5.4.2.12	Phosphoglycerate mutase (2,3-diphosphoglycerate-independent)
Control vs DSS 6-day	5.4.2.2	Phosphoglucomutase (alpha-D-glucose-1,6-bisphosphate-dependent)
Control vs DSS 6-day	5.99.1.3	DNA topoisomerase (ATP-hydrolyzing)
Control vs DSS 6-day	6.1.1.10	Methionine--tRNA ligase
Control vs DSS 6-day	6.1.1.11	Serine--tRNA ligase
Control vs DSS 6-day	6.1.1.12	Aspartate--tRNA ligase
Control vs DSS 6-day	6.1.1.17	Glutamate--tRNA ligase
Control vs DSS 6-day	6.1.1.18	Glutamine--tRNA ligase
Control vs DSS 6-day	6.1.1.2	Tryptophan--tRNA ligase
Control vs DSS 6-day	6.1.1.20	Phenylalanine--tRNA ligase
Control vs DSS 6-day	6.1.1.22	Asparagine--tRNA ligase
Control vs DSS 6-day	6.1.1.4	Leucine--tRNA ligase
Control vs DSS 6-day	6.1.1.5	Isoleucine--tRNA ligase
Control vs DSS 6-day	6.1.1.6	Lysine--tRNA ligase
Control vs DSS 6-day	6.1.1.7	Alanine--tRNA ligase
Control vs DSS 6-day	6.1.1.9	Valine--tRNA ligase
Control vs DSS 6-day	6.2.1.30	Phenylacetate--CoA ligase
Control vs DSS 6-day	6.3.1.1	Aspartate--ammonia ligase
Control vs DSS 6-day	6.3.2.4	D-alanine--D-alanine ligase
Control vs DSS 6-day	6.3.2.5	Phosphopantothenate--cysteine ligase
Control vs DSS 6-day	6.3.2.6	Phosphoribosylaminoimidazolesuccinocarboxamide synthase
Control vs DSS 6-day	6.3.2.9	UDP-N-acetylmuramoyl-L-alanine--D-glutamate ligase
Control vs DSS 6-day	6.3.4.14	Biotin carboxylase

Control vs DSS 6-day	6.3.5.1	NAD(+) synthase (glutamine-hydrolyzing)
Control vs DSS 6-day	6.3.5.3	Phosphoribosylformylglycinamidine synthase
Control vs DSS 6-day	6.3.5.5	Carbamoyl-phosphate synthase (glutamine-hydrolyzing)
Control vs DSS 6-day	6.4.1.2	Acetyl-CoA carboxylase
Control vs DSS 6-day	6.4.1.3	Propionyl-CoA carboxylase
Control vs DSS 6-day	6.5.1.2	DNA ligase (NAD(+))
Control vs DSS 6-day	7.1.2.2	NO_NAME
Control vs DSS 6-day	7.2.1.1	NO_NAME
Control vs DSS 6-day	1.1.1.100	3-oxoacyl-[acyl-carrier-protein] reductase
Control vs DSS 6-day	1.1.1.131	Mannuronate reductase
Control vs DSS 6-day	1.1.1.133	dTDP-4-dehydrorhamnose reductase
Control vs DSS 6-day	1.1.1.169	2-dehydropantoate 2-reductase
Control vs DSS 6-day	1.1.1.193	5-amino-6-(5-phosphoribosylamino)uracil reductase
Control vs DSS 6-day	1.1.1.205	IMP dehydrogenase
Control vs DSS 6-day	1.1.1.22	UDP-glucose 6-dehydrogenase
Control vs DSS 6-day	1.1.1.23	Histidinol dehydrogenase
Control vs DSS 6-day	1.1.1.25	Shikimate dehydrogenase
Control vs DSS 6-day	1.1.1.262	4-hydroxythreonine-4-phosphate dehydrogenase
Control vs DSS 6-day	1.1.1.267	1-deoxy-D-xylulose-5-phosphate reductoisomerase
Control vs DSS 6-day	1.1.1.271	GDP-L-fucose synthase
Control vs DSS 6-day	1.1.1.290	4-phosphoerythronate dehydrogenase
Control vs DSS 6-day	1.1.1.37	Malate dehydrogenase
Control vs DSS 6-day	1.1.1.40	Malate dehydrogenase (oxaloacetate-decarboxylating) (NADP(+))
Control vs DSS 6-day	1.1.1.42	Isocitrate dehydrogenase (NADP(+))
Control vs DSS 6-day	1.1.1.44	Phosphogluconate dehydrogenase (NADP(+)-dependent, decarboxylating)
Control vs DSS 6-day	1.1.1.49	Glucose-6-phosphate dehydrogenase (NADP(+))
Control vs DSS 6-day	1.1.1.58	Tagaturonate reductase
Control vs DSS 6-day	1.1.1.85	3-isopropylmalate dehydrogenase
Control vs DSS 6-day	1.1.1.86	Ketol-acid reductoisomerase (NADP(+))
Control vs DSS 6-day	1.1.1.94	Glycerol-3-phosphate dehydrogenase (NAD(P)(+))
Control vs DSS 6-day	1.1.1.95	Phosphoglycerate dehydrogenase
Control vs DSS 6-day	1.11.1.1	NADH peroxidase
Control vs DSS 6-day	1.11.1.15	Peroxiredoxin
Control vs DSS 6-day	1.15.1.1	Superoxide dismutase
Control vs DSS 6-day	1.16.3.2	Bacterial non-heme ferritin
Control vs DSS 6-day	1.17.4.1	Ribonucleoside-diphosphate reductase
Control vs DSS 6-day	1.17.4.2	Ribonucleoside-triphosphate reductase
Control vs DSS 6-day	1.17.7.3	(E)-4-hydroxy-3-methylbut-2-enyl-diphosphate synthase (flavodoxin)
Control vs DSS 6-day	1.17.7.4	NO_NAME
Control vs DSS 6-day	1.2.1.11	Aspartate-semialdehyde dehydrogenase
Control vs DSS 6-day	1.2.1.38	N-acetyl-gamma-glutamyl-phosphate reductase
Control vs DSS 6-day	1.2.1.41	Glutamate-5-semialdehyde dehydrogenase
Control vs DSS 6-day	1.2.7.8	Indolepyruvate ferredoxin oxidoreductase

Control vs DSS 6-day	1.3.1.1	Dihydrouracil dehydrogenase (NAD(+))
Control vs DSS 6-day	1.3.1.14	Dihydroorotate dehydrogenase (NAD(+))
Control vs DSS 6-day	1.3.1.26	Transferred entry
Control vs DSS 6-day	1.3.1.9	Enoyl-[acyl-carrier-protein] reductase (NADH)
Control vs DSS 6-day	1.3.1.98	UDP-N-acetylmuramate dehydrogenase
Control vs DSS 6-day	1.3.98.1	Dihydroorotate oxidase (fumarate)
Control vs DSS 6-day	1.3.99.5	3-oxo-5-alpha-steroid 4-dehydrogenase (acceptor)
Control vs DSS 6-day	1.4.1.13	Glutamate synthase (NADPH)
Control vs DSS 6-day	1.4.1.16	Diaminopimelate dehydrogenase
Control vs DSS 6-day	1.4.1.2	Glutamate dehydrogenase
Control vs DSS 6-day	1.4.1.3	Glutamate dehydrogenase (NAD(P)(+))
Control vs DSS 6-day	1.4.3.16	L-aspartate oxidase
Control vs DSS 6-day	1.4.4.2	Glycine dehydrogenase (aminomethyl-transferring)
Control vs DSS 6-day	1.5.1.2	Pyrroline-5-carboxylate reductase
Control vs DSS 6-day	1.5.1.20	Methylenetetrahydrofolate reductase (NAD(P)H)
Control vs DSS 6-day	1.5.1.3	Dihydrofolate reductase
Control vs DSS 6-day	1.5.1.39	FMN reductase (NAD(P)H)
Control vs DSS 6-day	1.5.1.5	Methylenetetrahydrofolate dehydrogenase (NADP(+))
Control vs DSS 6-day	1.6.5.11	NADH dehydrogenase (quinone)
Control vs DSS 6-day	1.6.5.3	NADH
Control vs DSS 6-day	1.6.99.1	NADPH dehydrogenase
Control vs DSS 6-day	1.6.99.5	Transferred entry
Control vs DSS 6-day	1.7.1.13	PreQ(1) synthase
Control vs DSS 6-day	1.8.1.4	Dihydrolipoyl dehydrogenase
Control vs DSS 6-day	1.8.1.9	Thioredoxin-disulfide reductase
Control vs DSS 6-day	1.97.1.4	[Formate-C-acetyltransferase]-activating enzyme
Control vs DSS 6-day	2.1.1.13	Methionine synthase
Control vs DSS 6-day	2.1.1.163	Demethylmenaquinone methyltransferase
Control vs DSS 6-day	2.1.1.182	16S rRNA (adenine(1518)-N(6)/adenine(1519)-N(6))-dimethyltransferase
Control vs DSS 6-day	2.1.1.185	23S rRNA (guanosine(2251)-2'-O)-methyltransferase
Control vs DSS 6-day	2.1.1.191	23S rRNA (cytosine(1962)-C(5))-methyltransferase
Control vs DSS 6-day	2.1.1.192	23S rRNA (adenine(2503)-C(2))-methyltransferase
Control vs DSS 6-day	2.1.1.193	16S rRNA (uracil(1498)-N(3))-methyltransferase
Control vs DSS 6-day	2.1.1.197	Malonyl-[acyl-carrier protein] O-methyltransferase
Control vs DSS 6-day	2.1.1.198	16S rRNA (cytidine(1402)-2'-O)-methyltransferase
Control vs DSS 6-day	2.1.1.199	16S rRNA (cytosine(1402)-N(4))-methyltransferase
Control vs DSS 6-day	2.1.1.228	tRNA (guanine(37)-N(1))-methyltransferase
Control vs DSS 6-day	2.1.1.33	tRNA (guanine(46)-N(7))-methyltransferase
Control vs DSS 6-day	2.1.1.45	Thymidylate synthase
Control vs DSS 6-day	2.1.1.63	Methylated-DNA--[protein]-cysteine S-methyltransferase
Control vs DSS 6-day	2.1.2.1	Glycine hydroxymethyltransferase
Control vs DSS 6-day	2.1.2.10	Aminomethyltransferase
Control vs DSS 6-day	2.1.2.11	3-methyl-2-oxobutanoate hydroxymethyltransferase

Control vs DSS 6-day	2.1.2.2	Phosphoribosylglycinamide formyltransferase
Control vs DSS 6-day	2.1.2.3	Phosphoribosylaminoimidazolecarboxamide formyltransferase
Control vs DSS 6-day	2.1.2.9	Methionyl-tRNA formyltransferase
Control vs DSS 6-day	2.1.3.2	Aspartate carbamoyltransferase
Control vs DSS 6-day	2.1.3.9	N-acetylornithine carbamoyltransferase
Control vs DSS 6-day	2.2.1.6	Acetolactate synthase
Control vs DSS 6-day	2.2.1.7	1-deoxy-D-xylulose-5-phosphate synthase
Control vs DSS 6-day	2.2.1.9	synthase
Control vs DSS 6-day	2.3.1.129	Acyl-[acyl-carrier-protein]--UDP-N-acetylglucosamine O-acyltransferase
Control vs DSS 6-day	2.3.1.179	Beta-ketoacyl-[acyl-carrier-protein] synthase II
Control vs DSS 6-day	2.3.1.180	Beta-ketoacyl-[acyl-carrier-protein] synthase III
Control vs DSS 6-day	2.3.1.181	Lipoyl(octanoyl) transferase
Control vs DSS 6-day	2.3.1.234	N(6)-L-threonylcarbamoyladenine synthase
Control vs DSS 6-day	2.3.1.28	Chloramphenicol O-acetyltransferase
Control vs DSS 6-day	2.3.1.29	Glycine C-acetyltransferase
Control vs DSS 6-day	2.3.1.31	Homoserine O-acetyltransferase
Control vs DSS 6-day	2.3.1.39	[Acyl-carrier-protein] S-malonyltransferase
Control vs DSS 6-day	2.3.1.47	8-amino-7-oxononanoate synthase
Control vs DSS 6-day	2.3.1.51	1-acylglycerol-3-phosphate O-acyltransferase
Control vs DSS 6-day	2.3.3.13	2-isopropylmalate synthase
Control vs DSS 6-day	2.4.1.18	1,4-alpha-glucan branching enzyme
Control vs DSS 6-day	2.4.1.182	Lipid-A-disaccharide synthase
Control vs DSS 6-day	2.4.1.21	Starch synthase
Control vs DSS 6-day	2.4.1.212	Hyaluronan synthase
Control vs DSS 6-day	2.4.1.227	acetylglucosaminyltransferase
Control vs DSS 6-day	2.4.1.320	1,4-beta-mannosyl-N-acetylglucosamine phosphorylase
Control vs DSS 6-day	2.4.1.54	Undecaprenyl-phosphate mannosyltransferase
Control vs DSS 6-day	2.4.2.1	Purine-nucleoside phosphorylase
Control vs DSS 6-day	2.4.2.10	Orotate phosphoribosyltransferase
Control vs DSS 6-day	2.4.2.14	Amidophosphoribosyltransferase
Control vs DSS 6-day	2.4.2.17	ATP phosphoribosyltransferase
Control vs DSS 6-day	2.4.2.18	Anthranilate phosphoribosyltransferase
Control vs DSS 6-day	2.4.2.19	Nicotinate-nucleotide diphosphorylase (carboxylating)
Control vs DSS 6-day	2.4.2.22	Xanthine phosphoribosyltransferase
Control vs DSS 6-day	2.4.2.29	tRNA-guanine(34) transglycosylase
Control vs DSS 6-day	2.4.2.53	Undecaprenyl-phosphate 4-deoxy-4-formamido-L-arabinose transferase
Control vs DSS 6-day	2.4.2.7	Adenine phosphoribosyltransferase
Control vs DSS 6-day	2.4.2.8	Hypoxanthine phosphoribosyltransferase
Control vs DSS 6-day	2.4.99.17	S-adenosylmethionine
Control vs DSS 6-day	2.5.1.15	Dihydropteroate synthase
Control vs DSS 6-day	2.5.1.17	Cob(I)yrinic acid a,c-diamide adenosyltransferase
Control vs DSS 6-day	2.5.1.19	3-phosphoshikimate 1-carboxyvinyltransferase
Control vs DSS 6-day	2.5.1.47	Cysteine synthase

Control vs DSS 6-day	2.5.1.55	3-deoxy-8-phosphooctulonate synthase
Control vs DSS 6-day	2.5.1.6	Methionine adenosyltransferase
Control vs DSS 6-day	2.5.1.7	UDP-N-acetylglucosamine 1-carboxyvinyltransferase
Control vs DSS 6-day	2.5.1.72	Quinolinate synthase
Control vs DSS 6-day	2.5.1.75	tRNA dimethylallyltransferase
Control vs DSS 6-day	2.5.1.78	6,7-dimethyl-8-ribityllumazine synthase
Control vs DSS 6-day	2.5.1.9	Riboflavin synthase
Control vs DSS 6-day	2.6.1.1	Aspartate transaminase
Control vs DSS 6-day	2.6.1.16	Glutamine--fructose-6-phosphate transaminase (isomerizing)
Control vs DSS 6-day	2.6.1.42	Branched-chain-amino-acid transaminase
Control vs DSS 6-day	2.6.1.52	Phosphoserine transaminase
Control vs DSS 6-day	2.6.1.62	Adenosylmethionine--8-amino-7-oxononanoate transaminase
Control vs DSS 6-day	2.6.1.83	LL-diaminopimelate aminotransferase
Control vs DSS 6-day	2.6.1.85	Aminodeoxychorismate synthase
Control vs DSS 6-day	2.6.1.9	Histidinol-phosphate transaminase
Control vs DSS 6-day	2.6.99.2	Pyridoxine 5'-phosphate synthase
Control vs DSS 6-day	2.7.1.107	Diacylglycerol kinase (ATP)
Control vs DSS 6-day	2.7.1.11	6-phosphofructokinase
Control vs DSS 6-day	2.7.1.130	Tetraacyldisaccharide 4'-kinase
Control vs DSS 6-day	2.7.1.148	4-(cytidine 5'-diphospho)-2-C-methyl-D-erythritol kinase
Control vs DSS 6-day	2.7.1.180	FAD
Control vs DSS 6-day	2.7.1.21	Thymidine kinase
Control vs DSS 6-day	2.7.1.25	Adenylyl-sulfate kinase
Control vs DSS 6-day	2.7.1.26	Riboflavin kinase
Control vs DSS 6-day	2.7.1.33	Pantothenate kinase
Control vs DSS 6-day	2.7.1.35	Pyridoxal kinase
Control vs DSS 6-day	2.7.1.40	Pyruvate kinase
Control vs DSS 6-day	2.7.1.48	Uridine kinase
Control vs DSS 6-day	2.7.1.69	Protein-N(pi)-phosphohistidine--sugar phosphotransferase
Control vs DSS 6-day	2.7.1.71	Shikimate kinase
Control vs DSS 6-day	2.7.1.90	Diphosphate--fructose-6-phosphate 1-phosphotransferase
Control vs DSS 6-day	2.7.13.3	Histidine kinase
Control vs DSS 6-day	2.7.2.1	Acetate kinase
Control vs DSS 6-day	2.7.2.11	Glutamate 5-kinase
Control vs DSS 6-day	2.7.2.3	Phosphoglycerate kinase
Control vs DSS 6-day	2.7.2.4	Aspartate kinase
Control vs DSS 6-day	2.7.2.8	Acetylglutamate kinase
Control vs DSS 6-day	2.7.4.1	Polyphosphate kinase
Control vs DSS 6-day	2.7.4.16	Thiamine-phosphate kinase
Control vs DSS 6-day	2.7.4.22	UMP kinase
Control vs DSS 6-day	2.7.4.25	(d)CMP kinase
Control vs DSS 6-day	2.7.4.3	Adenylate kinase
Control vs DSS 6-day	2.7.4.8	Guanylate kinase

Control vs DSS 6-day	2.7.6.1	Ribose-phosphate diphosphokinase
Control vs DSS 6-day	2.7.6.3	2-amino-4-hydroxy-6-hydroxymethyldihydropteridine diphosphokinase
Control vs DSS 6-day	2.7.7.13	Mannose-1-phosphate guanylyltransferase
Control vs DSS 6-day	2.7.7.18	Nicotinate-nucleotide adenylyltransferase
Control vs DSS 6-day	2.7.7.2	FAD synthetase
Control vs DSS 6-day	2.7.7.24	Glucose-1-phosphate thymidylyltransferase
Control vs DSS 6-day	2.7.7.3	Pantetheine-phosphate adenylyltransferase
Control vs DSS 6-day	2.7.7.38	3-deoxy-manno-octulosonate cytidylyltransferase
Control vs DSS 6-day	2.7.7.4	Sulfate adenylyltransferase
Control vs DSS 6-day	2.7.7.41	Phosphatidate cytidylyltransferase
Control vs DSS 6-day	2.7.7.6	DNA-directed RNA polymerase
Control vs DSS 6-day	2.7.7.60	2-C-methyl-D-erythritol 4-phosphate cytidylyltransferase
Control vs DSS 6-day	2.7.7.7	DNA-directed DNA polymerase
Control vs DSS 6-day	2.7.7.72	CCA tRNA nucleotidyltransferase
Control vs DSS 6-day	2.7.7.8	Polyribonucleotide nucleotidyltransferase
Control vs DSS 6-day	2.7.7.85	Diadenylate cyclase
Control vs DSS 6-day	2.7.8.13	Phospho-N-acetylmuramoyl-pentapeptide-transferase
Control vs DSS 6-day	2.7.8.8	CDP-diacylglycerol--serine O-phosphatidyltransferase
Control vs DSS 6-day	2.7.9.1	Pyruvate, phosphate dikinase
Control vs DSS 6-day	2.8.1.13	tRNA-uridine 2-sulfurtransferase
Control vs DSS 6-day	2.8.1.6	Biotin synthase
Control vs DSS 6-day	2.8.1.7	Cysteine desulfurase
Control vs DSS 6-day	2.8.1.8	Lipoyl synthase
Control vs DSS 6-day	2.8.4.3	tRNA-2-methylthio-N(6)-dimethylallyladenosine synthase
Control vs DSS 6-day	2.8.4.4	[Ribosomal protein S12] (aspartate(89)-C(3))-methylthiotransferase
Control vs DSS 6-day	3.1.1.11	Pectinesterase
Control vs DSS 6-day	3.1.1.29	Aminoacyl-tRNA hydrolase
Control vs DSS 6-day	3.1.1.96	D-aminoacyl-tRNA deacylase
Control vs DSS 6-day	3.1.11.5	Exodeoxyribonuclease V
Control vs DSS 6-day	3.1.11.6	Exodeoxyribonuclease VII
Control vs DSS 6-day	3.1.13.1	Exoribonuclease II
Control vs DSS 6-day	3.1.21.2	Deoxyribonuclease IV
Control vs DSS 6-day	3.1.21.4	Type II site-specific deoxyribonuclease
Control vs DSS 6-day	3.1.22.4	Crossover junction endodeoxyribonuclease
Control vs DSS 6-day	3.1.26.11	Ribonuclease Z
Control vs DSS 6-day	3.1.26.3	Ribonuclease III
Control vs DSS 6-day	3.1.26.4	Ribonuclease H
Control vs DSS 6-day	3.1.26.5	Ribonuclease P
Control vs DSS 6-day	3.1.3.11	Fructose-bisphosphatase
Control vs DSS 6-day	3.1.3.15	Histidinol-phosphatase
Control vs DSS 6-day	3.1.3.25	Inositol-phosphate phosphatase
Control vs DSS 6-day	3.1.3.45	3-deoxy-manno-octulosonate-8-phosphatase
Control vs DSS 6-day	3.1.3.5	5'-nucleotidase

Control vs DSS 6-day	3.1.3.7	3'(2'),5'-bisphosphate nucleotidase
Control vs DSS 6-day	3.1.4.46	Glycerophosphodiester phosphodiesterase
Control vs DSS 6-day	3.1.6.1	Arylsulfatase
Control vs DSS 6-day	3.2.1.131	Xylan alpha-1,2-glucuronosidase
Control vs DSS 6-day	3.2.1.135	Neopullulanase
Control vs DSS 6-day	3.2.1.165	Exo-1,4-beta-D-glucosaminidase
Control vs DSS 6-day	3.2.1.172	Unsaturated rhamnogalacturonyl hydrolase
Control vs DSS 6-day	3.2.1.21	Beta-glucosidase
Control vs DSS 6-day	3.2.1.22	Alpha-galactosidase
Control vs DSS 6-day	3.2.1.23	Beta-galactosidase
Control vs DSS 6-day	3.2.1.25	Beta-mannosidase
Control vs DSS 6-day	3.2.1.31	Beta-glucuronidase
Control vs DSS 6-day	3.2.1.37	Xylan 1,4-beta-xylosidase
Control vs DSS 6-day	3.2.1.41	Pullulanase
Control vs DSS 6-day	3.2.1.55	Non-reducing end alpha-L-arabinofuranosidase
Control vs DSS 6-day	3.2.1.8	Endo-1,4-beta-xylanase
Control vs DSS 6-day	3.2.2.27	Uracil-DNA glycosylase
Control vs DSS 6-day	3.2.2.n1	Cytokinin riboside 5'-monophosphate phosphoribohydrolase
Control vs DSS 6-day	3.3.1.1	Adenosylhomocysteinase
Control vs DSS 6-day	3.4.11.18	Methionyl aminopeptidase
Control vs DSS 6-day	3.4.11.4	Tripeptide aminopeptidase
Control vs DSS 6-day	3.4.11.9	Xaa-Pro aminopeptidase
Control vs DSS 6-day	3.4.13.18	Cytosol nonspecific dipeptidase
Control vs DSS 6-day	3.4.13.20	Beta-Ala-His dipeptidase
Control vs DSS 6-day	3.4.14.12	Xaa-Xaa-Pro tripeptidyl-peptidase
Control vs DSS 6-day	3.4.14.4	Dipeptidyl-peptidase III
Control vs DSS 6-day	3.4.15.5	Peptidyl-dipeptidase Dcp
Control vs DSS 6-day	3.4.16.4	Serine-type D-Ala-D-Ala carboxypeptidase
Control vs DSS 6-day	3.4.17.13	Muramoyltetrapeptide carboxypeptidase
Control vs DSS 6-day	3.4.21.102	C-terminal processing peptidase
Control vs DSS 6-day	3.4.21.105	Rhomboid protease
Control vs DSS 6-day	3.4.21.107	Peptidase Do
Control vs DSS 6-day	3.4.21.53	Endopeptidase La
Control vs DSS 6-day	3.4.21.89	Signal peptidase I
Control vs DSS 6-day	3.4.21.92	Endopeptidase Clp
Control vs DSS 6-day	3.4.23.36	Signal peptidase II
Control vs DSS 6-day	3.4.24.55	Pitrilysin
Control vs DSS 6-day	3.5.1.10	Formyltetrahydrofolate deformylase
Control vs DSS 6-day	3.5.1.100	(R)-amidase
Control vs DSS 6-day	3.5.1.104	Peptidoglycan-N-acetylglucosamine deacetylase
Control vs DSS 6-day	3.5.1.108	UDP-3-O-acyl-N-acetylglucosamine deacetylase
Control vs DSS 6-day	3.5.1.2	Glutaminase
Control vs DSS 6-day	3.5.1.25	N-acetylglucosamine-6-phosphate deacetylase

Control vs DSS 6-day	3.5.1.28	N-acetylmuramoyl-L-alanine amidase
Control vs DSS 6-day	3.5.1.88	Peptide deformylase
Control vs DSS 6-day	3.5.2.3	Dihydroorotase
Control vs DSS 6-day	3.5.2.6	Beta-lactamase
Control vs DSS 6-day	3.5.4.10	IMP cyclohydrolase
Control vs DSS 6-day	3.5.4.16	GTP cyclohydrolase I
Control vs DSS 6-day	3.5.4.19	Phosphoribosyl-AMP cyclohydrolase
Control vs DSS 6-day	3.5.4.25	GTP cyclohydrolase II
Control vs DSS 6-day	3.5.4.26	Diaminohydroxyphosphoribosylaminopyrimidine deaminase
Control vs DSS 6-day	3.5.4.3	Guanine deaminase
Control vs DSS 6-day	3.5.4.5	Cytidine deaminase
Control vs DSS 6-day	3.5.4.9	Methenyltetrahydrofolate cyclohydrolase
Control vs DSS 6-day	3.5.99.6	Glucosamine-6-phosphate deaminase
Control vs DSS 6-day	3.6.1.23	dUTP diphosphatase
Control vs DSS 6-day	3.6.1.27	Undecaprenyl-diphosphate phosphatase
Control vs DSS 6-day	3.6.1.31	Phosphoribosyl-ATP diphosphatase
Control vs DSS 6-day	3.6.1.66	XTP/dITP diphosphatase
Control vs DSS 6-day	3.6.3.31	Polyamine-transporting ATPase
Control vs DSS 6-day	3.6.3.41	Heme-transporting ATPase
Control vs DSS 6-day	3.6.4.12	DNA helicase
Control vs DSS 6-day	3.6.4.13	RNA helicase
Control vs DSS 6-day	3.6.5.n1	Elongation factor 4
Control vs DSS 6-day	4.1.1.11	Aspartate 1-decarboxylase
Control vs DSS 6-day	4.1.1.19	Arginine decarboxylase
Control vs DSS 6-day	4.1.1.20	Diaminopimelate decarboxylase
Control vs DSS 6-day	4.1.1.23	Orotidine-5'-phosphate decarboxylase
Control vs DSS 6-day	4.1.1.36	Phosphopantothenoylcysteine decarboxylase
Control vs DSS 6-day	4.1.1.49	Phosphoenolpyruvate carboxykinase (ATP)
Control vs DSS 6-day	4.1.1.65	Phosphatidylserine decarboxylase
Control vs DSS 6-day	4.1.2.19	Rhamnulose-1-phosphate aldolase
Control vs DSS 6-day	4.1.2.25	Dihydroneopterin aldolase
Control vs DSS 6-day	4.1.2.4	Deoxyribose-phosphate aldolase
Control vs DSS 6-day	4.1.2.50	6-carboxytetrahydropterin synthase
Control vs DSS 6-day	4.1.3.3	N-acetylneuraminate lyase
Control vs DSS 6-day	4.1.3.36	1,4-dihydroxy-2-naphthoyl-CoA synthase
Control vs DSS 6-day	4.1.3.38	Aminodeoxychorismate lyase
Control vs DSS 6-day	4.1.99.12	3,4-dihydroxy-2-butanone-4-phosphate synthase
Control vs DSS 6-day	4.2.1.10	3-dehydroquinate dehydratase
Control vs DSS 6-day	4.2.1.11	Phosphopyruvate hydratase
Control vs DSS 6-day	4.2.1.136	ADP-dependent NAD(P)H-hydrate dehydratase
Control vs DSS 6-day	4.2.1.17	Enoyl-CoA hydratase
Control vs DSS 6-day	4.2.1.19	Imidazoleglycerol-phosphate dehydratase
Control vs DSS 6-day	4.2.1.2	Fumarate hydratase

Control vs DSS 6-day	4.2.1.20	Tryptophan synthase
Control vs DSS 6-day	4.2.1.3	Aconitate hydratase
Control vs DSS 6-day	4.2.1.33	3-isopropylmalate dehydratase
Control vs DSS 6-day	4.2.1.46	dTDP-glucose 4,6-dehydratase
Control vs DSS 6-day	4.2.1.59	3-hydroxyacyl-[acyl-carrier-protein] dehydratase
Control vs DSS 6-day	4.2.1.8	Mannonate dehydratase
Control vs DSS 6-day	4.2.1.9	Dihydroxy-acid dehydratase
Control vs DSS 6-day	4.2.2.2	Pectate lyase
Control vs DSS 6-day	4.2.3.1	Threonine synthase
Control vs DSS 6-day	4.2.3.3	Methylglyoxal synthase
Control vs DSS 6-day	4.2.3.4	3-dehydroquinate synthase
Control vs DSS 6-day	4.2.3.5	Chorismate synthase
Control vs DSS 6-day	4.2.99.18	DNA-(apurinic or apyrimidinic site) lyase
Control vs DSS 6-day	4.3.1.17	L-serine ammonia-lyase
Control vs DSS 6-day	4.3.2.1	Argininosuccinate lyase
Control vs DSS 6-day	4.3.2.10	NO_NAME
Control vs DSS 6-day	4.3.2.2	Adenylosuccinate lyase
Control vs DSS 6-day	4.3.3.7	4-hydroxy-tetrahydrodipicolinate synthase
Control vs DSS 6-day	4.3.99.3	7-carboxy-7-deazaguanine synthase
Control vs DSS 6-day	4.6.1.12	2-C-methyl-D-erythritol 2,4-cyclodiphosphate synthase
Control vs DSS 6-day	5.1.1.1	Alanine racemase
Control vs DSS 6-day	5.1.1.3	Glutamate racemase
Control vs DSS 6-day	5.1.1.7	Diaminopimelate epimerase
Control vs DSS 6-day	5.1.3.13	dTDP-4-dehydrorhamnose 3,5-epimerase
Control vs DSS 6-day	5.1.3.3	Aldose 1-epimerase
Control vs DSS 6-day	5.1.3.32	L-rhamnose mutarotase
Control vs DSS 6-day	5.1.3.4	L-ribulose-5-phosphate 4-epimerase
Control vs DSS 6-day	5.1.99.6	NAD(P)H-hydrate epimerase
Control vs DSS 6-day	5.2.1.8	Peptidylprolyl isomerase
Control vs DSS 6-day	5.3.1.1	Triose-phosphate isomerase
Control vs DSS 6-day	5.3.1.12	Glucuronate isomerase
Control vs DSS 6-day	5.3.1.16	isomerase
Control vs DSS 6-day	5.3.1.17	5-dehydro-4-deoxy-D-glucuronate isomerase
Control vs DSS 6-day	5.3.1.24	Phosphoribosylanthranilate isomerase
Control vs DSS 6-day	5.3.1.4	L-arabinose isomerase
Control vs DSS 6-day	5.3.1.5	Xylose isomerase
Control vs DSS 6-day	5.3.1.9	Glucose-6-phosphate isomerase
Control vs DSS 6-day	5.3.3.2	Isopentenyl-diphosphate Delta-isomerase
Control vs DSS 6-day	5.4.2.12	Phosphoglycerate mutase (2,3-diphosphoglycerate-independent)
Control vs DSS 6-day	5.4.2.2	Phosphoglucomutase (alpha-D-glucose-1,6-bisphosphate-dependent)
Control vs DSS 6-day	5.4.2.6	Beta-phosphoglucomutase
Control vs DSS 6-day	5.4.4.2	Isochorismate synthase
Control vs DSS 6-day	5.4.99.12	tRNA pseudouridine(38-40) synthase

Control vs DSS 6-day	5.4.99.18	5-(carboxyamino)imidazole ribonucleotide mutase
Control vs DSS 6-day	5.4.99.25	tRNA pseudouridine(55) synthase
Control vs DSS 6-day	5.99.1.2	DNA topoisomerase
Control vs DSS 6-day	5.99.1.3	DNA topoisomerase (ATP-hydrolyzing)
Control vs DSS 6-day	6.1.1.1	Tyrosine--tRNA ligase
Control vs DSS 6-day	6.1.1.10	Methionine--tRNA ligase
Control vs DSS 6-day	6.1.1.11	Serine--tRNA ligase
Control vs DSS 6-day	6.1.1.12	Aspartate--tRNA ligase
Control vs DSS 6-day	6.1.1.14	Glycine--tRNA ligase
Control vs DSS 6-day	6.1.1.15	Proline--tRNA ligase
Control vs DSS 6-day	6.1.1.16	Cysteine--tRNA ligase
Control vs DSS 6-day	6.1.1.17	Glutamate--tRNA ligase
Control vs DSS 6-day	6.1.1.18	Glutamine--tRNA ligase
Control vs DSS 6-day	6.1.1.19	Arginine--tRNA ligase
Control vs DSS 6-day	6.1.1.2	Tryptophan--tRNA ligase
Control vs DSS 6-day	6.1.1.20	Phenylalanine--tRNA ligase
Control vs DSS 6-day	6.1.1.21	Histidine--tRNA ligase
Control vs DSS 6-day	6.1.1.22	Asparagine--tRNA ligase
Control vs DSS 6-day	6.1.1.3	Threonine--tRNA ligase
Control vs DSS 6-day	6.1.1.4	Leucine--tRNA ligase
Control vs DSS 6-day	6.1.1.5	Isoleucine--tRNA ligase
Control vs DSS 6-day	6.1.1.6	Lysine--tRNA ligase
Control vs DSS 6-day	6.1.1.7	Alanine--tRNA ligase
Control vs DSS 6-day	6.1.1.9	Valine--tRNA ligase
Control vs DSS 6-day	6.2.1.3	Long-chain-fatty-acid--CoA ligase
Control vs DSS 6-day	6.2.1.30	Phenylacetate--CoA ligase
Control vs DSS 6-day	6.3.1.1	Aspartate--ammonia ligase
Control vs DSS 6-day	6.3.1.2	Glutamate--ammonia ligase
Control vs DSS 6-day	6.3.2.1	Pantoate--beta-alanine ligase (AMP-forming)
Control vs DSS 6-day	6.3.2.10	UDP-N-acetylmuramoyl-tripeptide--D-alanyl-D-alanine ligase
Control vs DSS 6-day	6.3.2.13	UDP-N-acetylmuramoyl-L-alanyl-D-glutamate--2,6-diaminopimelate ligase
Control vs DSS 6-day	6.3.2.4	D-alanine--D-alanine ligase
Control vs DSS 6-day	6.3.2.5	Phosphopantothenate--cysteine ligase
Control vs DSS 6-day	6.3.2.6	Phosphoribosylaminoimidazolesuccinocarboxamide synthase
Control vs DSS 6-day	6.3.2.8	UDP-N-acetylmuramate--L-alanine ligase
Control vs DSS 6-day	6.3.2.9	UDP-N-acetylmuramoyl-L-alanine--D-glutamate ligase
Control vs DSS 6-day	6.3.3.2	5-formyltetrahydrofolate cyclo-ligase
Control vs DSS 6-day	6.3.3.3	Dethiobiotin synthase
Control vs DSS 6-day	6.3.4.13	Phosphoribosylamine--glycine ligase
Control vs DSS 6-day	6.3.4.14	Biotin carboxylase
Control vs DSS 6-day	6.3.4.19	tRNA(Ile)-lysidine synthetase
Control vs DSS 6-day	6.3.4.2	CTP synthase (glutamine hydrolyzing)
Control vs DSS 6-day	6.3.4.3	Formate--tetrahydrofolate ligase

Control vs DSS 6-day	6.3.4.4	Adenylosuccinate synthase
Control vs DSS 6-day	6.3.5.1	NAD(+) synthase (glutamine-hydrolyzing)
Control vs DSS 6-day	6.3.5.2	GMP synthase (glutamine-hydrolyzing)
Control vs DSS 6-day	6.3.5.3	Phosphoribosylformylglycinamidine synthase
Control vs DSS 6-day	6.3.5.5	Carbamoyl-phosphate synthase (glutamine-hydrolyzing)
Control vs DSS 6-day	6.4.1.2	Acetyl-CoA carboxylase
Control vs DSS 6-day	6.4.1.3	Propionyl-CoA carboxylase
Control vs DSS 6-day	6.5.1.2	DNA ligase (NAD(+))
Control vs DSS 6-day	7.1.2.2	NO_NAME
Control vs DSS 6-day	7.2.1.1	NO_NAME
Control vs DSS 6-day	1.1.1.37	Malate dehydrogenase
Control vs DSS 6-day	1.1.1.86	Ketol-acid reductoisomerase (NADP(+))
Control vs DSS 6-day	1.11.1.1	NADH peroxidase
Control vs DSS 6-day	1.11.1.15	Peroxiredoxin
Control vs DSS 6-day	1.16.3.2	Bacterial non-heme ferritin
Control vs DSS 6-day	1.4.1.16	Diaminopimelate dehydrogenase
Control vs DSS 6-day	2.1.2.3	Phosphoribosylaminoimidazolecarboxamide formyltransferase
Control vs DSS 6-day	2.3.1.179	Beta-ketoacyl-[acyl-carrier-protein] synthase II
Control vs DSS 6-day	2.3.1.39	[Acyl-carrier-protein] S-malonyltransferase
Control vs DSS 6-day	2.3.1.79	Maltose O-acetyltransferase
Control vs DSS 6-day	2.5.1.72	Quinolinate synthase
Control vs DSS 6-day	2.6.1.52	Phosphoserine transaminase
Control vs DSS 6-day	2.7.1.11	6-phosphofructokinase
Control vs DSS 6-day	2.7.13.3	Histidine kinase
Control vs DSS 6-day	2.7.2.3	Phosphoglycerate kinase
Control vs DSS 6-day	2.7.7.6	DNA-directed RNA polymerase
Control vs DSS 6-day	3.1.21.3	Type I site-specific deoxyribonuclease
Control vs DSS 6-day	3.4.11.18	Methionyl aminopeptidase
Control vs DSS 6-day	3.5.1.2	Glutaminase
Control vs DSS 6-day	3.5.4.10	IMP cyclohydrolase
Control vs DSS 6-day	3.5.99.6	Glucosamine-6-phosphate deaminase
Control vs DSS 6-day	3.6.4.12	DNA helicase
Control vs DSS 6-day	4.1.1.15	Glutamate decarboxylase
Control vs DSS 6-day	4.1.1.19	Arginine decarboxylase
Control vs DSS 6-day	4.1.1.49	Phosphoenolpyruvate carboxykinase (ATP)
Control vs DSS 6-day	5.2.1.8	Peptidylprolyl isomerase
Control vs DSS 6-day	5.3.1.1	Triose-phosphate isomerase
Control vs DSS 6-day	5.3.1.12	Glucuronate isomerase
Control vs DSS 6-day	5.3.1.5	Xylose isomerase
Control vs DSS 6-day	5.4.2.12	Phosphoglycerate mutase (2,3-diphosphoglycerate-independent)
Control vs DSS 6-day	5.99.1.3	DNA topoisomerase (ATP-hydrolyzing)
Control vs DSS 6-day	6.1.1.14	Glycine--tRNA ligase
Control vs DSS 6-day	6.1.1.22	Asparagine--tRNA ligase

Control vs DSS 6-day	6.1.1.3	Threonine--tRNA ligase
Control vs DSS 6-day	6.1.1.4	Leucine--tRNA ligase
Control vs DSS 6-day	6.3.4.4	Adenylosuccinate synthase
Control vs DSS 6-day	6.3.5.5	Carbamoyl-phosphate synthase (glutamine-hydrolyzing)
Control vs DSS 6-day	7.2.1.1	NO_NAME
Control vs DSS 6-day	1.1.1.205	IMP dehydrogenase
Control vs DSS 6-day	1.1.1.37	Malate dehydrogenase
Control vs DSS 6-day	1.1.1.86	Ketol-acid reductoisomerase (NADP(+))
Control vs DSS 6-day	1.1.1.95	Phosphoglycerate dehydrogenase
Control vs DSS 6-day	1.11.1.1	NADH peroxidase
Control vs DSS 6-day	2.6.1.52	Phosphoserine transaminase
Control vs DSS 6-day	2.7.1.90	Diphosphate--fructose-6-phosphate 1-phosphotransferase
Control vs DSS 6-day	2.7.13.3	Histidine kinase
Control vs DSS 6-day	2.7.7.6	DNA-directed RNA polymerase
Control vs DSS 6-day	4.1.1.49	Phosphoenolpyruvate carboxykinase (ATP)
Control vs DSS 6-day	4.1.3.36	1,4-dihydroxy-2-naphthoyl-CoA synthase
Control vs DSS 6-day	4.2.1.2	Fumarate hydratase
Control vs DSS 6-day	5.3.1.1	Triose-phosphate isomerase
Control vs DSS 6-day	6.1.1.11	Serine--tRNA ligase
Control vs DSS 6-day	6.1.1.14	Glycine--tRNA ligase
Control vs DSS 6-day	7.2.1.1	NO_NAME
Control vs DSS 6-day	1.1.1.205	IMP dehydrogenase
Control vs DSS 6-day	1.1.1.267	1-deoxy-D-xylulose-5-phosphate reductoisomerase
Control vs DSS 6-day	1.1.1.37	Malate dehydrogenase
Control vs DSS 6-day	1.1.1.58	Tagaturonate reductase
Control vs DSS 6-day	1.1.1.85	3-isopropylmalate dehydrogenase
Control vs DSS 6-day	1.1.1.86	Ketol-acid reductoisomerase (NADP(+))
Control vs DSS 6-day	1.1.1.95	Phosphoglycerate dehydrogenase
Control vs DSS 6-day	1.11.1.1	NADH peroxidase
Control vs DSS 6-day	1.15.1.1	Superoxide dismutase
Control vs DSS 6-day	1.17.4.1	Ribonucleoside-diphosphate reductase
Control vs DSS 6-day	1.17.4.2	Ribonucleoside-triphosphate reductase
Control vs DSS 6-day	1.2.1.11	Aspartate-semialdehyde dehydrogenase
Control vs DSS 6-day	1.2.7.8	Indolepyruvate ferredoxin oxidoreductase
Control vs DSS 6-day	1.3.1.14	Dihydroorotate dehydrogenase (NAD(+))
Control vs DSS 6-day	1.4.1.16	Diaminopimelate dehydrogenase
Control vs DSS 6-day	1.4.1.2	Glutamate dehydrogenase
Control vs DSS 6-day	1.4.1.3	Glutamate dehydrogenase (NAD(P)(+))
Control vs DSS 6-day	1.4.3.16	L-aspartate oxidase
Control vs DSS 6-day	1.6.5.11	NADH dehydrogenase (quinone)
Control vs DSS 6-day	1.6.5.3	NADH
Control vs DSS 6-day	1.6.99.5	Transferred entry
Control vs DSS 6-day	1.8.1.9	Thioredoxin-disulfide reductase

Control vs DSS 6-day	2.1.1.182	16S rRNA (adenine(1518)-N(6)/adenine(1519)-N(6))-dimethyltransferase
Control vs DSS 6-day	2.1.1.191	23S rRNA (cytosine(1962)-C(5))-methyltransferase
Control vs DSS 6-day	2.1.1.228	tRNA (guanine(37)-N(1))-methyltransferase
Control vs DSS 6-day	2.1.2.1	Glycine hydroxymethyltransferase
Control vs DSS 6-day	2.1.2.11	3-methyl-2-oxobutanoate hydroxymethyltransferase
Control vs DSS 6-day	2.1.3.2	Aspartate carbamoyltransferase
Control vs DSS 6-day	2.3.1.179	Beta-ketoacyl-[acyl-carrier-protein] synthase II
Control vs DSS 6-day	2.3.1.180	Beta-ketoacyl-[acyl-carrier-protein] synthase III
Control vs DSS 6-day	2.3.1.181	Lipoyl(octanoyl) transferase
Control vs DSS 6-day	2.3.1.234	N(6)-L-threonylcarbamoyladenine synthase
Control vs DSS 6-day	2.3.1.47	8-amino-7-oxononanoate synthase
Control vs DSS 6-day	2.4.1.21	Starch synthase
Control vs DSS 6-day	2.4.1.320	1,4-beta-mannosyl-N-acetylglucosamine phosphorylase
Control vs DSS 6-day	2.4.2.1	Purine-nucleoside phosphorylase
Control vs DSS 6-day	2.4.2.10	Orotate phosphoribosyltransferase
Control vs DSS 6-day	2.4.2.29	tRNA-guanine(34) transglycosylase
Control vs DSS 6-day	2.4.99.17	S-adenosylmethionine
Control vs DSS 6-day	2.5.1.7	UDP-N-acetylglucosamine 1-carboxyvinyltransferase
Control vs DSS 6-day	2.6.1.16	Glutamine--fructose-6-phosphate transaminase (isomerizing)
Control vs DSS 6-day	2.6.1.52	Phosphoserine transaminase
Control vs DSS 6-day	2.7.1.33	Pantothenate kinase
Control vs DSS 6-day	2.7.1.40	Pyruvate kinase
Control vs DSS 6-day	2.7.13.3	Histidine kinase
Control vs DSS 6-day	2.7.2.1	Acetate kinase
Control vs DSS 6-day	2.7.2.3	Phosphoglycerate kinase
Control vs DSS 6-day	2.7.6.1	Ribose-phosphate diphosphokinase
Control vs DSS 6-day	2.7.7.24	Glucose-1-phosphate thymidylyltransferase
Control vs DSS 6-day	2.7.7.6	DNA-directed RNA polymerase
Control vs DSS 6-day	2.7.7.7	DNA-directed DNA polymerase
Control vs DSS 6-day	2.7.7.8	Polyribonucleotide nucleotidyltransferase
Control vs DSS 6-day	2.7.8.13	Phospho-N-acetylmuramoyl-pentapeptide-transferase
Control vs DSS 6-day	2.8.4.4	[Ribosomal protein S12] (aspartate(89)-C(3))-methylthiotransferase
Control vs DSS 6-day	3.1.3.11	Fructose-bisphosphatase
Control vs DSS 6-day	3.4.11.18	Methionyl aminopeptidase
Control vs DSS 6-day	3.4.11.4	Tripeptide aminopeptidase
Control vs DSS 6-day	3.4.11.9	Xaa-Pro aminopeptidase
Control vs DSS 6-day	3.4.13.20	Beta-Ala-His dipeptidase
Control vs DSS 6-day	3.4.21.107	Peptidase Do
Control vs DSS 6-day	3.4.21.92	Endopeptidase Clp
Control vs DSS 6-day	3.5.99.6	Glucosamine-6-phosphate deaminase
Control vs DSS 6-day	3.6.4.12	DNA helicase
Control vs DSS 6-day	3.6.5.n1	Elongation factor 4
Control vs DSS 6-day	4.1.1.11	Aspartate 1-decarboxylase

Control vs DSS 6-day	4.1.1.19	Arginine decarboxylase
Control vs DSS 6-day	4.1.1.23	Orotidine-5'-phosphate decarboxylase
Control vs DSS 6-day	4.1.1.49	Phosphoenolpyruvate carboxykinase (ATP)
Control vs DSS 6-day	4.1.2.4	Deoxyribose-phosphate aldolase
Control vs DSS 6-day	4.2.1.11	Phosphopyruvate hydratase
Control vs DSS 6-day	4.2.1.33	3-isopropylmalate dehydratase
Control vs DSS 6-day	4.3.2.1	Argininosuccinate lyase
Control vs DSS 6-day	5.2.1.8	Peptidylprolyl isomerase
Control vs DSS 6-day	5.3.1.1	Triose-phosphate isomerase
Control vs DSS 6-day	5.3.1.14	L-rhamnose isomerase
Control vs DSS 6-day	5.3.1.17	5-dehydro-4-deoxy-D-glucuronate isomerase
Control vs DSS 6-day	5.3.1.4	L-arabinose isomerase
Control vs DSS 6-day	5.3.1.9	Glucose-6-phosphate isomerase
Control vs DSS 6-day	5.4.2.12	Phosphoglycerate mutase (2,3-diphosphoglycerate-independent)
Control vs DSS 6-day	5.4.2.2	Phosphoglucomutase (alpha-D-glucose-1,6-bisphosphate-dependent)
Control vs DSS 6-day	5.99.1.3	DNA topoisomerase (ATP-hydrolyzing)
Control vs DSS 6-day	6.1.1.10	Methionine--tRNA ligase
Control vs DSS 6-day	6.1.1.11	Serine--tRNA ligase
Control vs DSS 6-day	6.1.1.12	Aspartate--tRNA ligase
Control vs DSS 6-day	6.1.1.14	Glycine--tRNA ligase
Control vs DSS 6-day	6.1.1.16	Cysteine--tRNA ligase
Control vs DSS 6-day	6.1.1.20	Phenylalanine--tRNA ligase
Control vs DSS 6-day	6.1.1.22	Asparagine--tRNA ligase
Control vs DSS 6-day	6.1.1.3	Threonine--tRNA ligase
Control vs DSS 6-day	6.1.1.5	Isoleucine--tRNA ligase
Control vs DSS 6-day	6.3.2.6	Phosphoribosylaminoimidazolesuccinocarboxamide synthase
Control vs DSS 6-day	6.3.5.3	Phosphoribosylformylglycinamidine synthase
Control vs DSS 6-day	6.3.5.5	Carbamoyl-phosphate synthase (glutamine-hydrolyzing)
Control vs DSS 6-day	7.1.2.2	NO_NAME
Control vs DSS 6-day	7.2.1.1	NO_NAME
Control vs DSS 6-day	1.1.1.169	2-dehydropantoate 2-reductase
Control vs DSS 6-day	1.1.1.205	IMP dehydrogenase
Control vs DSS 6-day	1.1.1.22	UDP-glucose 6-dehydrogenase
Control vs DSS 6-day	1.1.1.25	Shikimate dehydrogenase
Control vs DSS 6-day	1.1.1.262	4-hydroxythreonine-4-phosphate dehydrogenase
Control vs DSS 6-day	1.1.1.271	GDP-L-fucose synthase
Control vs DSS 6-day	1.1.1.28	D-lactate dehydrogenase
Control vs DSS 6-day	1.1.1.290	4-phosphoerythronate dehydrogenase
Control vs DSS 6-day	1.1.1.37	Malate dehydrogenase
Control vs DSS 6-day	1.1.1.58	Tagaturonate reductase
Control vs DSS 6-day	1.1.1.86	Ketol-acid reductoisomerase (NADP(+))
Control vs DSS 6-day	1.1.1.95	Phosphoglycerate dehydrogenase
Control vs DSS 6-day	1.11.1.1	NADH peroxidase

Control vs DSS 6-day	1.11.1.15	Peroxiredoxin
Control vs DSS 6-day	1.15.1.1	Superoxide dismutase
Control vs DSS 6-day	1.17.4.1	Ribonucleoside-diphosphate reductase
Control vs DSS 6-day	1.17.4.2	Ribonucleoside-triphosphate reductase
Control vs DSS 6-day	1.2.1.11	Aspartate-semialdehyde dehydrogenase
Control vs DSS 6-day	1.2.7.8	Indolepyruvate ferredoxin oxidoreductase
Control vs DSS 6-day	1.3.1.1	Dihydrouracil dehydrogenase (NAD(+))
Control vs DSS 6-day	1.3.1.14	Dihydroorotate dehydrogenase (NAD(+))
Control vs DSS 6-day	1.3.1.9	Enoyl-[acyl-carrier-protein] reductase (NADH)
Control vs DSS 6-day	1.3.98.1	Dihydroorotate oxidase (fumarate)
Control vs DSS 6-day	1.4.1.13	Glutamate synthase (NADPH)
Control vs DSS 6-day	1.4.1.16	Diaminopimelate dehydrogenase
Control vs DSS 6-day	1.4.1.2	Glutamate dehydrogenase
Control vs DSS 6-day	1.4.1.3	Glutamate dehydrogenase (NAD(P)(+))
Control vs DSS 6-day	1.4.4.2	Glycine dehydrogenase (aminomethyl-transferring)
Control vs DSS 6-day	1.6.5.11	NADH dehydrogenase (quinone)
Control vs DSS 6-day	1.8.1.9	Thioredoxin-disulfide reductase
Control vs DSS 6-day	2.1.1.163	Demethylmenaquinone methyltransferase
Control vs DSS 6-day	2.1.1.182	16S rRNA (adenine(1518)-N(6)/adenine(1519)-N(6))-dimethyltransferase
Control vs DSS 6-day	2.1.1.192	23S rRNA (adenine(2503)-C(2))-methyltransferase
Control vs DSS 6-day	2.1.1.228	tRNA (guanine(37)-N(1))-methyltransferase
Control vs DSS 6-day	2.1.1.37	DNA (cytosine-5-)-methyltransferase
Control vs DSS 6-day	2.1.2.1	Glycine hydroxymethyltransferase
Control vs DSS 6-day	2.1.2.3	Phosphoribosylaminoimidazolecarboxamide formyltransferase
Control vs DSS 6-day	2.1.2.9	Methionyl-tRNA formyltransferase
Control vs DSS 6-day	2.1.3.2	Aspartate carbamoyltransferase
Control vs DSS 6-day	2.1.3.9	N-acetylornithine carbamoyltransferase
Control vs DSS 6-day	2.2.1.6	Acetolactate synthase
Control vs DSS 6-day	2.2.1.7	1-deoxy-D-xylulose-5-phosphate synthase
Control vs DSS 6-day	2.3.1.129	Acyl-[acyl-carrier-protein]--UDP-N-acetylglucosamine O-acyltransferase
Control vs DSS 6-day	2.3.1.179	Beta-ketoacyl-[acyl-carrier-protein] synthase II
Control vs DSS 6-day	2.3.1.180	Beta-ketoacyl-[acyl-carrier-protein] synthase III
Control vs DSS 6-day	2.3.1.234	N(6)-L-threonylcarbamoyladenine synthase
Control vs DSS 6-day	2.3.1.47	8-amino-7-oxononanoate synthase
Control vs DSS 6-day	2.3.1.51	1-acylglycerol-3-phosphate O-acyltransferase
Control vs DSS 6-day	2.3.3.13	2-isopropylmalate synthase
Control vs DSS 6-day	2.4.1.18	1,4-alpha-glucan branching enzyme
Control vs DSS 6-day	2.4.1.182	Lipid-A-disaccharide synthase
Control vs DSS 6-day	2.4.1.21	Starch synthase
Control vs DSS 6-day	2.4.1.212	Hyaluronan synthase
Control vs DSS 6-day	2.4.2.10	Orotate phosphoribosyltransferase
Control vs DSS 6-day	2.4.2.14	Amidophosphoribosyltransferase
Control vs DSS 6-day	2.4.2.17	ATP phosphoribosyltransferase

Control vs DSS 6-day	2.4.2.19	Nicotinate-nucleotide diphosphorylase (carboxylating)
Control vs DSS 6-day	2.5.1.15	Dihydropteroate synthase
Control vs DSS 6-day	2.5.1.47	Cysteine synthase
Control vs DSS 6-day	2.5.1.6	Methionine adenosyltransferase
Control vs DSS 6-day	2.5.1.7	UDP-N-acetylglucosamine 1-carboxyvinyltransferase
Control vs DSS 6-day	2.5.1.75	tRNA dimethylallyltransferase
Control vs DSS 6-day	2.5.1.78	6,7-dimethyl-8-ribityllumazine synthase
Control vs DSS 6-day	2.6.1.16	Glutamine--fructose-6-phosphate transaminase (isomerizing)
Control vs DSS 6-day	2.6.1.42	Branched-chain-amino-acid transaminase
Control vs DSS 6-day	2.6.1.52	Phosphoserine transaminase
Control vs DSS 6-day	2.6.1.83	LL-diaminopimelate aminotransferase
Control vs DSS 6-day	2.7.1.11	6-phosphofructokinase
Control vs DSS 6-day	2.7.1.40	Pyruvate kinase
Control vs DSS 6-day	2.7.1.69	Protein-N(pi)-phosphohistidine--sugar phosphotransferase
Control vs DSS 6-day	2.7.1.90	Diphosphate--fructose-6-phosphate 1-phosphotransferase
Control vs DSS 6-day	2.7.2.1	Acetate kinase
Control vs DSS 6-day	2.7.2.3	Phosphoglycerate kinase
Control vs DSS 6-day	2.7.2.4	Aspartate kinase
Control vs DSS 6-day	2.7.4.6	Nucleoside-diphosphate kinase
Control vs DSS 6-day	2.7.6.1	Ribose-phosphate diphosphokinase
Control vs DSS 6-day	2.7.7.6	DNA-directed RNA polymerase
Control vs DSS 6-day	2.7.7.7	DNA-directed DNA polymerase
Control vs DSS 6-day	2.7.7.72	CCA tRNA nucleotidyltransferase
Control vs DSS 6-day	2.7.7.8	Polyribonucleotide nucleotidyltransferase
Control vs DSS 6-day	2.7.8.13	Phospho-N-acetylmuramoyl-pentapeptide-transferase
Control vs DSS 6-day	2.8.1.7	Cysteine desulfurase
Control vs DSS 6-day	2.8.1.8	Lipoyl synthase
Control vs DSS 6-day	3.1.21.3	Type I site-specific deoxyribonuclease
Control vs DSS 6-day	3.1.22.4	Crossover junction endodeoxyribonuclease
Control vs DSS 6-day	3.1.26.5	Ribonuclease P
Control vs DSS 6-day	3.1.3.11	Fructose-bisphosphatase
Control vs DSS 6-day	3.1.4.46	Glycerophosphodiester phosphodiesterase
Control vs DSS 6-day	3.2.1.135	Neopullulanase
Control vs DSS 6-day	3.2.1.172	Unsaturated rhamnogalacturonyl hydrolase
Control vs DSS 6-day	3.2.1.22	Alpha-galactosidase
Control vs DSS 6-day	3.2.1.23	Beta-galactosidase
Control vs DSS 6-day	3.2.1.37	Xylan 1,4-beta-xylosidase
Control vs DSS 6-day	3.2.2.27	Uracil-DNA glycosylase
Control vs DSS 6-day	3.2.2.n1	Cytokinin riboside 5'-monophosphate phosphoribohydrolase
Control vs DSS 6-day	3.4.11.18	Methionyl aminopeptidase
Control vs DSS 6-day	3.4.11.4	Tripeptide aminopeptidase
Control vs DSS 6-day	3.4.13.18	Cytosol nonspecific dipeptidase
Control vs DSS 6-day	3.4.14.12	Xaa-Xaa-Pro tripeptidyl-peptidase

Control vs DSS 6-day	3.4.21.53	Endopeptidase La
Control vs DSS 6-day	3.4.21.89	Signal peptidase I
Control vs DSS 6-day	3.5.1.10	Formyltetrahydrofolate deformylase
Control vs DSS 6-day	3.5.1.108	UDP-3-O-acyl-N-acetylglucosamine deacetylase
Control vs DSS 6-day	3.5.1.25	N-acetylglucosamine-6-phosphate deacetylase
Control vs DSS 6-day	3.5.4.10	IMP cyclohydrolase
Control vs DSS 6-day	3.5.4.16	GTP cyclohydrolase I
Control vs DSS 6-day	3.5.99.6	Glucosamine-6-phosphate deaminase
Control vs DSS 6-day	3.6.3.31	Polyamine-transporting ATPase
Control vs DSS 6-day	3.6.3.41	Heme-transporting ATPase
Control vs DSS 6-day	3.6.4.12	DNA helicase
Control vs DSS 6-day	3.6.4.13	RNA helicase
Control vs DSS 6-day	4.1.1.11	Aspartate 1-decarboxylase
Control vs DSS 6-day	4.1.1.23	Orotidine-5'-phosphate decarboxylase
Control vs DSS 6-day	4.1.1.36	Phosphopantothenoylcysteine decarboxylase
Control vs DSS 6-day	4.1.1.49	Phosphoenolpyruvate carboxykinase (ATP)
Control vs DSS 6-day	4.1.1.65	Phosphatidylserine decarboxylase
Control vs DSS 6-day	4.1.3.36	1,4-dihydroxy-2-naphthoyl-CoA synthase
Control vs DSS 6-day	4.2.1.10	3-dehydroquinate dehydratase
Control vs DSS 6-day	4.2.1.11	Phosphopyruvate hydratase
Control vs DSS 6-day	4.2.1.17	Enoyl-CoA hydratase
Control vs DSS 6-day	4.2.1.2	Fumarate hydratase
Control vs DSS 6-day	4.2.1.3	Aconitate hydratase
Control vs DSS 6-day	4.2.1.33	3-isopropylmalate dehydratase
Control vs DSS 6-day	4.2.1.47	GDP-mannose 4,6-dehydratase
Control vs DSS 6-day	4.2.1.59	3-hydroxyacyl-[acyl-carrier-protein] dehydratase
Control vs DSS 6-day	4.2.1.8	Mannonate dehydratase
Control vs DSS 6-day	4.2.1.9	Dihydroxy-acid dehydratase
Control vs DSS 6-day	4.2.2.2	Pectate lyase
Control vs DSS 6-day	4.2.3.3	Methylglyoxal synthase
Control vs DSS 6-day	4.3.1.17	L-serine ammonia-lyase
Control vs DSS 6-day	4.3.3.7	4-hydroxy-tetrahydrodipicolinate synthase
Control vs DSS 6-day	5.1.3.3	Aldose 1-epimerase
Control vs DSS 6-day	5.2.1.8	Peptidylprolyl isomerase
Control vs DSS 6-day	5.3.1.17	5-dehydro-4-deoxy-D-glucuronate isomerase
Control vs DSS 6-day	5.3.1.25	L-fucose isomerase
Control vs DSS 6-day	5.4.2.12	Phosphoglycerate mutase (2,3-diphosphoglycerate-independent)
Control vs DSS 6-day	5.4.2.2	Phosphoglucomutase (alpha-D-glucose-1,6-bisphosphate-dependent)
Control vs DSS 6-day	5.99.1.3	DNA topoisomerase (ATP-hydrolyzing)
Control vs DSS 6-day	6.1.1.10	Methionine--tRNA ligase
Control vs DSS 6-day	6.1.1.11	Serine--tRNA ligase
Control vs DSS 6-day	6.1.1.12	Aspartate--tRNA ligase
Control vs DSS 6-day	6.1.1.14	Glycine--tRNA ligase

Control vs DSS 6-day	6.1.1.15	Proline--tRNA ligase
Control vs DSS 6-day	6.1.1.17	Glutamate--tRNA ligase
Control vs DSS 6-day	6.1.1.18	Glutamine--tRNA ligase
Control vs DSS 6-day	6.1.1.20	Phenylalanine--tRNA ligase
Control vs DSS 6-day	6.1.1.21	Histidine--tRNA ligase
Control vs DSS 6-day	6.1.1.22	Asparagine--tRNA ligase
Control vs DSS 6-day	6.1.1.3	Threonine--tRNA ligase
Control vs DSS 6-day	6.1.1.4	Leucine--tRNA ligase
Control vs DSS 6-day	6.1.1.5	Isoleucine--tRNA ligase
Control vs DSS 6-day	6.1.1.6	Lysine--tRNA ligase
Control vs DSS 6-day	6.1.1.7	Alanine--tRNA ligase
Control vs DSS 6-day	6.1.1.9	Valine--tRNA ligase
Control vs DSS 6-day	6.3.2.4	D-alanine--D-alanine ligase
Control vs DSS 6-day	6.3.2.5	Phosphopantothenate--cysteine ligase
Control vs DSS 6-day	6.3.2.6	Phosphoribosylaminoimidazolesuccinocarboxamide synthase
Control vs DSS 6-day	6.3.2.9	UDP-N-acetylmuramoyl-L-alanine--D-glutamate ligase
Control vs DSS 6-day	6.3.4.13	Phosphoribosylamine--glycine ligase
Control vs DSS 6-day	6.3.4.3	Formate--tetrahydrofolate ligase
Control vs DSS 6-day	6.3.5.3	Phosphoribosylformylglycinamidine synthase
Control vs DSS 6-day	6.3.5.5	Carbamoyl-phosphate synthase (glutamine-hydrolyzing)
Control vs DSS 6-day	6.5.1.2	DNA ligase (NAD(+))
Control vs DSS 6-day	7.1.2.2	NO_NAME
Control vs DSS 6-day	7.2.1.1	NO_NAME
Control vs DSS 6-day	1.1.1.133	dTDP-4-dehydrorhamnose reductase
Control vs DSS 6-day	1.1.1.169	2-dehydropantoate 2-reductase
Control vs DSS 6-day	1.1.1.205	IMP dehydrogenase
Control vs DSS 6-day	1.1.1.22	UDP-glucose 6-dehydrogenase
Control vs DSS 6-day	1.1.1.25	Shikimate dehydrogenase
Control vs DSS 6-day	1.1.1.262	4-hydroxythreonine-4-phosphate dehydrogenase
Control vs DSS 6-day	1.1.1.37	Malate dehydrogenase
Control vs DSS 6-day	1.1.1.42	Isocitrate dehydrogenase (NADP(+))
Control vs DSS 6-day	1.1.1.44	Phosphogluconate dehydrogenase (NADP(+)-dependent, decarboxylating)
Control vs DSS 6-day	1.1.1.58	Tagaturonate reductase
Control vs DSS 6-day	1.1.1.95	Phosphoglycerate dehydrogenase
Control vs DSS 6-day	1.1.9.1	Alcohol dehydrogenase (azurin)
Control vs DSS 6-day	1.11.1.1	NADH peroxidase
Control vs DSS 6-day	1.11.1.15	Peroxiredoxin
Control vs DSS 6-day	1.17.4.2	Ribonucleoside-triphosphate reductase
Control vs DSS 6-day	1.17.7.3	(E)-4-hydroxy-3-methylbut-2-enyl-diphosphate synthase (flavodoxin)
Control vs DSS 6-day	1.3.1.1	Dihydrouracil dehydrogenase (NAD(+))
Control vs DSS 6-day	1.3.98.1	Dihydroorotate oxidase (fumarate)
Control vs DSS 6-day	1.3.99.5	3-oxo-5-alpha-steroid 4-dehydrogenase (acceptor)
Control vs DSS 6-day	1.4.1.13	Glutamate synthase (NADPH)

Control vs DSS 6-day	1.4.1.16	Diaminopimelate dehydrogenase
Control vs DSS 6-day	1.4.1.2	Glutamate dehydrogenase
Control vs DSS 6-day	1.4.1.21	Aspartate dehydrogenase
Control vs DSS 6-day	1.4.1.3	Glutamate dehydrogenase (NAD(P)(+))
Control vs DSS 6-day	1.4.3.5	Pyridoxal 5'-phosphate synthase
Control vs DSS 6-day	1.4.4.2	Glycine dehydrogenase (aminomethyl-transferring)
Control vs DSS 6-day	1.5.1.2	Pyrroline-5-carboxylate reductase
Control vs DSS 6-day	1.5.1.20	Methylenetetrahydrofolate reductase (NAD(P)H)
Control vs DSS 6-day	1.6.5.11	NADH dehydrogenase (quinone)
Control vs DSS 6-day	1.6.5.3	NADH
Control vs DSS 6-day	1.6.99.1	NADPH dehydrogenase
Control vs DSS 6-day	1.6.99.5	Transferred entry
Control vs DSS 6-day	1.8.1.4	Dihydrolipoyl dehydrogenase
Control vs DSS 6-day	1.8.1.9	Thioredoxin-disulfide reductase
Control vs DSS 6-day	1.97.1.4	[Formate-C-acetyltransferase]-activating enzyme
Control vs DSS 6-day	2.1.1.13	Methionine synthase
Control vs DSS 6-day	2.1.1.163	Demethylmenaquinone methyltransferase
Control vs DSS 6-day	2.1.1.177	23S rRNA (pseudouridine(1915)-N(3))-methyltransferase
Control vs DSS 6-day	2.1.1.185	23S rRNA (guanosine(2251)-2'-O)-methyltransferase
Control vs DSS 6-day	2.1.1.192	23S rRNA (adenine(2503)-C(2))-methyltransferase
Control vs DSS 6-day	2.1.1.193	16S rRNA (uracil(1498)-N(3))-methyltransferase
Control vs DSS 6-day	2.1.1.198	16S rRNA (cytidine(1402)-2'-O)-methyltransferase
Control vs DSS 6-day	2.1.1.37	DNA (cytosine-5-)-methyltransferase
Control vs DSS 6-day	2.1.1.63	Methylated-DNA--[protein]-cysteine S-methyltransferase
Control vs DSS 6-day	2.1.1.72	Site-specific DNA-methyltransferase (adenine-specific)
Control vs DSS 6-day	2.1.2.2	Phosphoribosylglycinamide formyltransferase
Control vs DSS 6-day	2.2.1.6	Acetolactate synthase
Control vs DSS 6-day	2.2.1.7	1-deoxy-D-xylulose-5-phosphate synthase
Control vs DSS 6-day	2.3.1.28	Chloramphenicol O-acetyltransferase
Control vs DSS 6-day	2.3.1.31	Homoserine O-acetyltransferase
Control vs DSS 6-day	2.3.1.47	8-amino-7-oxononanoate synthase
Control vs DSS 6-day	2.3.3.13	2-isopropylmalate synthase
Control vs DSS 6-day	2.4.1.182	Lipid-A-disaccharide synthase
Control vs DSS 6-day	2.4.1.21	Starch synthase
Control vs DSS 6-day	2.4.1.212	Hyaluronan synthase
Control vs DSS 6-day	2.4.1.320	1,4-beta-mannosyl-N-acetylglucosamine phosphorylase
Control vs DSS 6-day	2.4.2.14	Amidophosphoribosyltransferase
Control vs DSS 6-day	2.5.1.15	Dihydropteroate synthase
Control vs DSS 6-day	2.5.1.17	Cob(I)yrinic acid a,c-diamide adenosyltransferase
Control vs DSS 6-day	2.5.1.47	Cysteine synthase
Control vs DSS 6-day	2.5.1.6	Methionine adenosyltransferase
Control vs DSS 6-day	2.5.1.72	Quinolinate synthase
Control vs DSS 6-day	2.5.1.75	tRNA dimethylallyltransferase

Control vs DSS 6-day	2.6.1.16	Glutamine--fructose-6-phosphate transaminase (isomerizing)
Control vs DSS 6-day	2.6.1.52	Phosphoserine transaminase
Control vs DSS 6-day	2.7.1.15	Ribokinase
Control vs DSS 6-day	2.7.1.180	FAD
Control vs DSS 6-day	2.7.1.26	Riboflavin kinase
Control vs DSS 6-day	2.7.1.33	Pantothenate kinase
Control vs DSS 6-day	2.7.1.35	Pyridoxal kinase
Control vs DSS 6-day	2.7.1.40	Pyruvate kinase
Control vs DSS 6-day	2.7.1.5	Rhamnulokinase
Control vs DSS 6-day	2.7.1.69	Protein-N(pi)-phosphohistidine--sugar phosphotransferase
Control vs DSS 6-day	2.7.1.71	Shikimate kinase
Control vs DSS 6-day	2.7.1.90	Diphosphate--fructose-6-phosphate 1-phosphotransferase
Control vs DSS 6-day	2.7.13.3	Histidine kinase
Control vs DSS 6-day	2.7.2.1	Acetate kinase
Control vs DSS 6-day	2.7.2.11	Glutamate 5-kinase
Control vs DSS 6-day	2.7.2.3	Phosphoglycerate kinase
Control vs DSS 6-day	2.7.4.1	Polyphosphate kinase
Control vs DSS 6-day	2.7.4.25	(d)CMP kinase
Control vs DSS 6-day	2.7.7.13	Mannose-1-phosphate guanylyltransferase
Control vs DSS 6-day	2.7.7.2	FAD synthetase
Control vs DSS 6-day	2.7.7.49	RNA-directed DNA polymerase
Control vs DSS 6-day	2.7.7.6	DNA-directed RNA polymerase
Control vs DSS 6-day	2.7.7.60	2-C-methyl-D-erythritol 4-phosphate cytidylyltransferase
Control vs DSS 6-day	2.7.7.63	Lipoate--protein ligase
Control vs DSS 6-day	2.7.7.7	DNA-directed DNA polymerase
Control vs DSS 6-day	2.7.7.8	Polyribonucleotide nucleotidyltransferase
Control vs DSS 6-day	2.7.7.85	Diadenylate cyclase
Control vs DSS 6-day	2.8.1.8	Lipoyl synthase
Control vs DSS 6-day	3.1.1.11	Pectinesterase
Control vs DSS 6-day	3.1.1.29	Aminoacyl-tRNA hydrolase
Control vs DSS 6-day	3.1.11.2	Exodeoxyribonuclease III
Control vs DSS 6-day	3.1.11.6	Exodeoxyribonuclease VII
Control vs DSS 6-day	3.1.13.1	Exoribonuclease II
Control vs DSS 6-day	3.1.21.2	Deoxyribonuclease IV
Control vs DSS 6-day	3.1.21.3	Type I site-specific deoxyribonuclease
Control vs DSS 6-day	3.1.26.11	Ribonuclease Z
Control vs DSS 6-day	3.1.26.4	Ribonuclease H
Control vs DSS 6-day	3.1.3.1	Alkaline phosphatase
Control vs DSS 6-day	3.1.3.11	Fructose-bisphosphatase
Control vs DSS 6-day	3.1.3.25	Inositol-phosphate phosphatase
Control vs DSS 6-day	3.1.6.1	Arylsulfatase
Control vs DSS 6-day	3.2.1.131	Xylan alpha-1,2-glucuronosidase
Control vs DSS 6-day	3.2.1.165	Exo-1,4-beta-D-glucosaminidase

Control vs DSS 6-day	3.2.1.172	Unsaturated rhamnogalacturonyl hydrolase
Control vs DSS 6-day	3.2.1.177	Alpha-D-xyloside xylohydrolase
Control vs DSS 6-day	3.2.1.21	Beta-glucosidase
Control vs DSS 6-day	3.2.1.22	Alpha-galactosidase
Control vs DSS 6-day	3.2.1.23	Beta-galactosidase
Control vs DSS 6-day	3.2.1.25	Beta-mannosidase
Control vs DSS 6-day	3.2.1.31	Beta-glucuronidase
Control vs DSS 6-day	3.2.1.37	Xylan 1,4-beta-xylosidase
Control vs DSS 6-day	3.2.1.51	Alpha-L-fucosidase
Control vs DSS 6-day	3.2.1.55	Non-reducing end alpha-L-arabinofuranosidase
Control vs DSS 6-day	3.2.1.8	Endo-1,4-beta-xylanase
Control vs DSS 6-day	3.2.1.82	Exo-poly-alpha-galacturonosidase
Control vs DSS 6-day	3.4.11.9	Xaa-Pro aminopeptidase
Control vs DSS 6-day	3.4.13.20	Beta-Ala-His dipeptidase
Control vs DSS 6-day	3.4.14.12	Xaa-Xaa-Pro tripeptidyl-peptidase
Control vs DSS 6-day	3.4.14.4	Dipeptidyl-peptidase III
Control vs DSS 6-day	3.4.15.5	Peptidyl-dipeptidase Dcp
Control vs DSS 6-day	3.4.16.4	Serine-type D-Ala-D-Ala carboxypeptidase
Control vs DSS 6-day	3.4.21.105	Rhomboid protease
Control vs DSS 6-day	3.4.21.107	Peptidase Do
Control vs DSS 6-day	3.4.21.53	Endopeptidase La
Control vs DSS 6-day	3.4.21.89	Signal peptidase I
Control vs DSS 6-day	3.5.1.10	Formyltetrahydrofolate deformylase
Control vs DSS 6-day	3.5.1.100	(R)-amidase
Control vs DSS 6-day	3.5.1.104	Peptidoglycan-N-acetylglucosamine deacetylase
Control vs DSS 6-day	3.5.1.2	Glutaminase
Control vs DSS 6-day	3.5.1.25	N-acetylglucosamine-6-phosphate deacetylase
Control vs DSS 6-day	3.5.1.28	N-acetylmuramoyl-L-alanine amidase
Control vs DSS 6-day	3.5.1.88	Peptide deformylase
Control vs DSS 6-day	3.5.2.3	Dihydroorotase
Control vs DSS 6-day	3.5.2.7	Imidazolonepropionase
Control vs DSS 6-day	3.5.4.16	GTP cyclohydrolase I
Control vs DSS 6-day	3.5.4.25	GTP cyclohydrolase II
Control vs DSS 6-day	3.5.99.6	Glucosamine-6-phosphate deaminase
Control vs DSS 6-day	3.6.1.27	Undecaprenyl-diphosphate phosphatase
Control vs DSS 6-day	3.6.3.54	Cu(+) exporting ATPase
Control vs DSS 6-day	3.6.4.12	DNA helicase
Control vs DSS 6-day	3.6.4.13	RNA helicase
Control vs DSS 6-day	4.1.1.19	Arginine decarboxylase
Control vs DSS 6-day	4.1.1.20	Diaminopimelate decarboxylase
Control vs DSS 6-day	4.1.1.36	Phosphopantothenoylcysteine decarboxylase
Control vs DSS 6-day	4.1.1.49	Phosphoenolpyruvate carboxykinase (ATP)
Control vs DSS 6-day	4.1.1.65	Phosphatidylserine decarboxylase

Control vs DSS 6-day	4.1.2.19	Rhamnulose-1-phosphate aldolase
Control vs DSS 6-day	4.1.99.12	3,4-dihydroxy-2-butanone-4-phosphate synthase
Control vs DSS 6-day	4.2.1.10	3-dehydroquinate dehydratase
Control vs DSS 6-day	4.2.1.126	N-acetylmuramic acid 6-phosphate etherase
Control vs DSS 6-day	4.2.1.136	ADP-dependent NAD(P)H-hydrate dehydratase
Control vs DSS 6-day	4.2.1.2	Fumarate hydratase
Control vs DSS 6-day	4.2.1.3	Aconitate hydratase
Control vs DSS 6-day	4.2.1.33	3-isopropylmalate dehydratase
Control vs DSS 6-day	4.2.1.46	dTDP-glucose 4,6-dehydratase
Control vs DSS 6-day	4.2.2.2	Pectate lyase
Control vs DSS 6-day	4.2.3.1	Threonine synthase
Control vs DSS 6-day	4.3.1.17	L-serine ammonia-lyase
Control vs DSS 6-day	4.3.1.4	Formimidoyltetrahydrofolate cyclodeaminase
Control vs DSS 6-day	4.3.2.10	NO_NAME
Control vs DSS 6-day	5.1.3.1	Ribulose-phosphate 3-epimerase
Control vs DSS 6-day	5.1.3.3	Aldose 1-epimerase
Control vs DSS 6-day	5.1.99.6	NAD(P)H-hydrate epimerase
Control vs DSS 6-day	5.2.1.8	Peptidylprolyl isomerase
Control vs DSS 6-day	5.3.1.12	Glucuronate isomerase
Control vs DSS 6-day	5.3.1.14	L-rhamnose isomerase
Control vs DSS 6-day	5.3.1.16	isomerase
Control vs DSS 6-day	5.3.1.17	5-dehydro-4-deoxy-D-glucuronate isomerase
Control vs DSS 6-day	5.3.1.9	Glucose-6-phosphate isomerase
Control vs DSS 6-day	5.4.99.18	5-(carboxyamino)imidazole ribonucleotide mutase
Control vs DSS 6-day	5.4.99.25	tRNA pseudouridine(55) synthase
Control vs DSS 6-day	5.99.1.2	DNA topoisomerase
Control vs DSS 6-day	5.99.1.3	DNA topoisomerase (ATP-hydrolyzing)
Control vs DSS 6-day	6.1.1.12	Aspartate--tRNA ligase
Control vs DSS 6-day	6.1.1.15	Proline--tRNA ligase
Control vs DSS 6-day	6.1.1.18	Glutamine--tRNA ligase
Control vs DSS 6-day	6.1.1.20	Phenylalanine--tRNA ligase
Control vs DSS 6-day	6.1.1.22	Asparagine--tRNA ligase
Control vs DSS 6-day	6.1.1.3	Threonine--tRNA ligase
Control vs DSS 6-day	6.1.1.5	Isoleucine--tRNA ligase
Control vs DSS 6-day	6.1.1.6	Lysine--tRNA ligase
Control vs DSS 6-day	6.2.1.30	Phenylacetate--CoA ligase
Control vs DSS 6-day	6.3.1.2	Glutamate--ammonia ligase
Control vs DSS 6-day	6.3.2.5	Phosphopantothenate--cysteine ligase
Control vs DSS 6-day	6.3.3.3	Dethiobiotin synthase
Control vs DSS 6-day	6.3.4.13	Phosphoribosylamine--glycine ligase
Control vs DSS 6-day	6.3.4.2	CTP synthase (glutamine hydrolyzing)
Control vs DSS 6-day	6.3.5.5	Carbamoyl-phosphate synthase (glutamine-hydrolyzing)
Control vs DSS 6-day	6.5.1.2	DNA ligase (NAD(+))

Control vs DSS 6-day	7.1.2.2	NO_NAME
Control vs DSS 6-day	7.2.1.1	NO_NAME
Control vs DSS 6-day	2.7.7.6	DNA-directed RNA polymerase
Control vs DSS 6-day	1.1.1.37	Malate dehydrogenase
Control vs DSS 6-day	1.11.1.1	NADH peroxidase
Control vs DSS 6-day	1.4.1.2	Glutamate dehydrogenase
Control vs DSS 6-day	2.6.1.52	Phosphoserine transaminase
Control vs DSS 6-day	2.7.7.6	DNA-directed RNA polymerase
Control vs DSS 6-day	3.4.11.18	Methionyl aminopeptidase
Control vs DSS 6-day	4.1.1.49	Phosphoenolpyruvate carboxykinase (ATP)
Control vs DSS 6-day	4.3.2.1	Argininosuccinate lyase
Control vs DSS 6-day	5.3.1.5	Xylose isomerase
Control vs DSS 6-day	5.4.2.12	Phosphoglycerate mutase (2,3-diphosphoglycerate-independent)
Control vs DSS 6-day	5.99.1.3	DNA topoisomerase (ATP-hydrolyzing)
Control vs DSS 6-day	6.1.1.14	Glycine--tRNA ligase
Control vs DSS 6-day	6.1.1.15	Proline--tRNA ligase
Control vs DSS 6-day	6.1.1.22	Asparagine--tRNA ligase
Control vs DSS 6-day	6.3.5.5	Carbamoyl-phosphate synthase (glutamine-hydrolyzing)
Control vs DSS 6-day	1.1.1.37	Malate dehydrogenase
Control vs DSS 6-day	1.3.1.9	Enoyl-[acyl-carrier-protein] reductase (NADH)
Control vs DSS 6-day	2.7.7.6	DNA-directed RNA polymerase
Control vs DSS 6-day	4.3.2.1	Argininosuccinate lyase
Control vs DSS 6-day	5.3.1.12	Glucuronate isomerase
Control vs DSS 6-day	6.1.1.14	Glycine--tRNA ligase
Control vs DSS 6-day	6.1.1.22	Asparagine--tRNA ligase
Control vs DSS 6-day	1.1.1.205	IMP dehydrogenase
Control vs DSS 6-day	1.1.1.86	Ketol-acid reductoisomerase (NADP(+))
Control vs DSS 6-day	1.1.1.95	Phosphoglycerate dehydrogenase
Control vs DSS 6-day	1.11.1.1	NADH peroxidase
Control vs DSS 6-day	1.11.1.15	Peroxiredoxin
Control vs DSS 6-day	1.15.1.1	Superoxide dismutase
Control vs DSS 6-day	1.16.3.2	Bacterial non-heme ferritin
Control vs DSS 6-day	1.17.4.1	Ribonucleoside-diphosphate reductase
Control vs DSS 6-day	1.17.7.4	NO_NAME
Control vs DSS 6-day	1.2.1.11	Aspartate-semialdehyde dehydrogenase
Control vs DSS 6-day	1.2.1.38	N-acetyl-gamma-glutamyl-phosphate reductase
Control vs DSS 6-day	1.3.1.9	Enoyl-[acyl-carrier-protein] reductase (NADH)
Control vs DSS 6-day	1.4.1.16	Diaminopimelate dehydrogenase
Control vs DSS 6-day	1.4.1.2	Glutamate dehydrogenase
Control vs DSS 6-day	1.4.1.3	Glutamate dehydrogenase (NAD(P)(+))
Control vs DSS 6-day	2.1.1.198	16S rRNA (cytidine(1402)-2'-O)-methyltransferase
Control vs DSS 6-day	2.1.1.45	Thymidylate synthase
Control vs DSS 6-day	2.1.1.72	Site-specific DNA-methyltransferase (adenine-specific)

Control vs DSS 6-day	2.1.2.1	Glycine hydroxymethyltransferase
Control vs DSS 6-day	2.1.2.3	Phosphoribosylaminoimidazolecarboxamide formyltransferase
Control vs DSS 6-day	2.1.3.2	Aspartate carbamoyltransferase
Control vs DSS 6-day	2.3.1.129	Acyl-[acyl-carrier-protein]--UDP-N-acetylglucosamine O-acyltransferase
Control vs DSS 6-day	2.3.1.179	Beta-ketoacyl-[acyl-carrier-protein] synthase II
Control vs DSS 6-day	2.3.1.180	Beta-ketoacyl-[acyl-carrier-protein] synthase III
Control vs DSS 6-day	2.3.1.234	N(6)-L-threonylcarbamoyladenine synthase
Control vs DSS 6-day	2.3.1.47	8-amino-7-oxononanoate synthase
Control vs DSS 6-day	2.3.1.50	Serine C-palmitoyltransferase
Control vs DSS 6-day	2.3.1.79	Maltose O-acetyltransferase
Control vs DSS 6-day	2.4.1.1	Glycogen phosphorylase
Control vs DSS 6-day	2.4.2.1	Purine-nucleoside phosphorylase
Control vs DSS 6-day	2.4.2.10	Orotate phosphoribosyltransferase
Control vs DSS 6-day	2.4.2.29	tRNA-guanine(34) transglycosylase
Control vs DSS 6-day	2.4.99.17	S-adenosylmethionine
Control vs DSS 6-day	2.6.1.52	Phosphoserine transaminase
Control vs DSS 6-day	2.7.1.11	6-phosphofructokinase
Control vs DSS 6-day	2.7.1.40	Pyruvate kinase
Control vs DSS 6-day	2.7.1.90	Diphosphate--fructose-6-phosphate 1-phosphotransferase
Control vs DSS 6-day	2.7.13.3	Histidine kinase
Control vs DSS 6-day	2.7.2.3	Phosphoglycerate kinase
Control vs DSS 6-day	2.7.2.4	Aspartate kinase
Control vs DSS 6-day	2.7.4.25	(d)CMP kinase
Control vs DSS 6-day	2.7.6.1	Ribose-phosphate diphosphokinase
Control vs DSS 6-day	2.7.7.4	Sulfate adenylyltransferase
Control vs DSS 6-day	2.7.7.6	DNA-directed RNA polymerase
Control vs DSS 6-day	2.7.7.8	Polyribonucleotide nucleotidyltransferase
Control vs DSS 6-day	2.7.9.1	Pyruvate, phosphate dikinase
Control vs DSS 6-day	3.1.21.3	Type I site-specific deoxyribonuclease
Control vs DSS 6-day	3.4.11.18	Methionyl aminopeptidase
Control vs DSS 6-day	3.4.21.92	Endopeptidase Clp
Control vs DSS 6-day	3.5.1.108	UDP-3-O-acyl-N-acetylglucosamine deacetylase
Control vs DSS 6-day	3.5.4.10	IMP cyclohydrolase
Control vs DSS 6-day	3.5.99.6	Glucosamine-6-phosphate deaminase
Control vs DSS 6-day	3.6.4.12	DNA helicase
Control vs DSS 6-day	4.1.1.15	Glutamate decarboxylase
Control vs DSS 6-day	4.1.1.19	Arginine decarboxylase
Control vs DSS 6-day	4.1.1.23	Orotidine-5'-phosphate decarboxylase
Control vs DSS 6-day	4.1.1.49	Phosphoenolpyruvate carboxykinase (ATP)
Control vs DSS 6-day	4.2.1.11	Phosphopyruvate hydratase
Control vs DSS 6-day	4.2.1.2	Fumarate hydratase
Control vs DSS 6-day	4.2.1.20	Tryptophan synthase
Control vs DSS 6-day	4.2.1.59	3-hydroxyacyl-[acyl-carrier-protein] dehydratase

Control vs DSS 6-day	4.3.2.2	Adenylosuccinate lyase
Control vs DSS 6-day	5.2.1.8	Peptidylprolyl isomerase
Control vs DSS 6-day	5.3.1.1	Triose-phosphate isomerase
Control vs DSS 6-day	5.3.1.12	Glucuronate isomerase
Control vs DSS 6-day	5.3.1.17	5-dehydro-4-deoxy-D-glucuronate isomerase
Control vs DSS 6-day	5.3.1.25	L-fucose isomerase
Control vs DSS 6-day	5.3.1.9	Glucose-6-phosphate isomerase
Control vs DSS 6-day	5.4.2.11	Phosphoglycerate mutase (2,3-diphosphoglycerate-dependent)
Control vs DSS 6-day	5.4.2.12	Phosphoglycerate mutase (2,3-diphosphoglycerate-independent)
Control vs DSS 6-day	5.99.1.3	DNA topoisomerase (ATP-hydrolyzing)
Control vs DSS 6-day	6.1.1.1	Tyrosine--tRNA ligase
Control vs DSS 6-day	6.1.1.10	Methionine--tRNA ligase
Control vs DSS 6-day	6.1.1.11	Serine--tRNA ligase
Control vs DSS 6-day	6.1.1.12	Aspartate--tRNA ligase
Control vs DSS 6-day	6.1.1.14	Glycine--tRNA ligase
Control vs DSS 6-day	6.1.1.15	Proline--tRNA ligase
Control vs DSS 6-day	6.1.1.19	Arginine--tRNA ligase
Control vs DSS 6-day	6.1.1.20	Phenylalanine--tRNA ligase
Control vs DSS 6-day	6.1.1.22	Asparagine--tRNA ligase
Control vs DSS 6-day	6.1.1.3	Threonine--tRNA ligase
Control vs DSS 6-day	6.2.1.30	Phenylacetate--CoA ligase
Control vs DSS 6-day	6.3.4.2	CTP synthase (glutamine hydrolyzing)
Control vs DSS 6-day	6.3.4.4	Adenylosuccinate synthase
Control vs DSS 6-day	6.3.5.2	GMP synthase (glutamine-hydrolyzing)
Control vs DSS 6-day	6.3.5.3	Phosphoribosylformylglycinamidine synthase
Control vs DSS 6-day	6.3.5.5	Carbamoyl-phosphate synthase (glutamine-hydrolyzing)
Control vs DSS 6-day	7.1.2.2	NO_NAME
Control vs DSS 6-day	7.2.1.1	NO_NAME
Control vs DSS 6-day	1.1.1.100	3-oxoacyl-[acyl-carrier-protein] reductase
Control vs DSS 6-day	1.1.1.37	Malate dehydrogenase
Control vs DSS 6-day	1.11.1.1	NADH peroxidase
Control vs DSS 6-day	1.4.1.3	Glutamate dehydrogenase (NAD(P)(+))
Control vs DSS 6-day	2.7.1.90	Diphosphate--fructose-6-phosphate 1-phosphotransferase
Control vs DSS 6-day	2.7.13.3	Histidine kinase
Control vs DSS 6-day	2.7.7.6	DNA-directed RNA polymerase
Control vs DSS 6-day	2.7.7.8	Polyribonucleotide nucleotidyltransferase
Control vs DSS 6-day	3.4.11.18	Methionyl aminopeptidase
Control vs DSS 6-day	3.5.99.6	Glucosamine-6-phosphate deaminase
Control vs DSS 6-day	4.1.1.49	Phosphoenolpyruvate carboxykinase (ATP)
Control vs DSS 6-day	4.2.1.46	dTDP-glucose 4,6-dehydratase
Control vs DSS 6-day	5.99.1.3	DNA topoisomerase (ATP-hydrolyzing)
Control vs DSS 6-day	1.1.1.100	3-oxoacyl-[acyl-carrier-protein] reductase
Control vs DSS 6-day	1.1.1.122	D-threo-aldose 1-dehydrogenase

Control vs DSS 6-day	1.1.1.169	2-dehydropantoate 2-reductase
Control vs DSS 6-day	1.1.1.205	IMP dehydrogenase
Control vs DSS 6-day	1.1.1.22	UDP-glucose 6-dehydrogenase
Control vs DSS 6-day	1.1.1.267	1-deoxy-D-xylulose-5-phosphate reductoisomerase
Control vs DSS 6-day	1.1.1.290	4-phosphoerythronate dehydrogenase
Control vs DSS 6-day	1.1.1.37	Malate dehydrogenase
Control vs DSS 6-day	1.1.1.42	Isocitrate dehydrogenase (NADP(+))
Control vs DSS 6-day	1.1.1.44	Phosphogluconate dehydrogenase (NADP(+)-dependent, decarboxylating)
Control vs DSS 6-day	1.1.1.58	Tagaturonate reductase
Control vs DSS 6-day	1.1.1.85	3-isopropylmalate dehydrogenase
Control vs DSS 6-day	1.1.1.86	Ketol-acid reductoisomerase (NADP(+))
Control vs DSS 6-day	1.1.1.94	Glycerol-3-phosphate dehydrogenase (NAD(P)(+))
Control vs DSS 6-day	1.1.1.95	Phosphoglycerate dehydrogenase
Control vs DSS 6-day	1.11.1.1	NADH peroxidase
Control vs DSS 6-day	1.15.1.1	Superoxide dismutase
Control vs DSS 6-day	1.16.3.2	Bacterial non-heme ferritin
Control vs DSS 6-day	1.17.4.1	Ribonucleoside-diphosphate reductase
Control vs DSS 6-day	1.17.4.2	Ribonucleoside-triphosphate reductase
Control vs DSS 6-day	1.17.7.4	NO_NAME
Control vs DSS 6-day	1.2.1.11	Aspartate-semialdehyde dehydrogenase
Control vs DSS 6-day	1.2.1.38	N-acetyl-gamma-glutamyl-phosphate reductase
Control vs DSS 6-day	1.2.7.8	Indolepyruvate ferredoxin oxidoreductase
Control vs DSS 6-day	1.3.1.1	Dihydrouracil dehydrogenase (NAD(+))
Control vs DSS 6-day	1.3.1.14	Dihydroorotate dehydrogenase (NAD(+))
Control vs DSS 6-day	1.3.1.26	Transferred entry
Control vs DSS 6-day	1.3.1.9	Enoyl-[acyl-carrier-protein] reductase (NADH)
Control vs DSS 6-day	1.3.98.1	Dihydroorotate oxidase (fumarate)
Control vs DSS 6-day	1.3.99.5	3-oxo-5-alpha-steroid 4-dehydrogenase (acceptor)
Control vs DSS 6-day	1.4.1.2	Glutamate dehydrogenase
Control vs DSS 6-day	1.4.1.21	Aspartate dehydrogenase
Control vs DSS 6-day	1.4.1.3	Glutamate dehydrogenase (NAD(P)(+))
Control vs DSS 6-day	1.4.3.16	L-aspartate oxidase
Control vs DSS 6-day	1.4.3.5	Pyridoxal 5'-phosphate synthase
Control vs DSS 6-day	1.4.4.2	Glycine dehydrogenase (aminomethyl-transferring)
Control vs DSS 6-day	1.5.1.5	Methylenetetrahydrofolate dehydrogenase (NADP(+))
Control vs DSS 6-day	1.6.5.11	NADH dehydrogenase (quinone)
Control vs DSS 6-day	1.6.5.3	NADH
Control vs DSS 6-day	1.6.99.1	NADPH dehydrogenase
Control vs DSS 6-day	1.6.99.5	Transferred entry
Control vs DSS 6-day	1.8.1.4	Dihydrolipoyl dehydrogenase
Control vs DSS 6-day	1.8.1.9	Thioredoxin-disulfide reductase
Control vs DSS 6-day	2.1.1.13	Methionine synthase
Control vs DSS 6-day	2.1.1.163	Demethylmenaquinone methyltransferase

Control vs DSS 6-day	2.1.1.182	16S rRNA (adenine(1518)-N(6)/adenine(1519)-N(6))-dimethyltransferase
Control vs DSS 6-day	2.1.1.185	23S rRNA (guanosine(2251)-2'-O)-methyltransferase
Control vs DSS 6-day	2.1.1.191	23S rRNA (cytosine(1962)-C(5))-methyltransferase
Control vs DSS 6-day	2.1.1.192	23S rRNA (adenine(2503)-C(2))-methyltransferase
Control vs DSS 6-day	2.1.1.193	16S rRNA (uracil(1498)-N(3))-methyltransferase
Control vs DSS 6-day	2.1.1.198	16S rRNA (cytidine(1402)-2'-O)-methyltransferase
Control vs DSS 6-day	2.1.1.199	16S rRNA (cytosine(1402)-N(4))-methyltransferase
Control vs DSS 6-day	2.1.1.228	tRNA (guanine(37)-N(1))-methyltransferase
Control vs DSS 6-day	2.1.1.37	DNA (cytosine-5-)-methyltransferase
Control vs DSS 6-day	2.1.2.1	Glycine hydroxymethyltransferase
Control vs DSS 6-day	2.1.2.10	Aminomethyltransferase
Control vs DSS 6-day	2.1.2.11	3-methyl-2-oxobutanoate hydroxymethyltransferase
Control vs DSS 6-day	2.1.2.2	Phosphoribosylglycinamide formyltransferase
Control vs DSS 6-day	2.1.2.3	Phosphoribosylaminoimidazolecarboxamide formyltransferase
Control vs DSS 6-day	2.1.3.2	Aspartate carbamoyltransferase
Control vs DSS 6-day	2.1.3.9	N-acetylornithine carbamoyltransferase
Control vs DSS 6-day	2.2.1.6	Acetolactate synthase
Control vs DSS 6-day	2.2.1.7	1-deoxy-D-xylulose-5-phosphate synthase
Control vs DSS 6-day	2.3.1.129	Acyl-[acyl-carrier-protein]--UDP-N-acetylglucosamine O-acyltransferase
Control vs DSS 6-day	2.3.1.179	Beta-ketoacyl-[acyl-carrier-protein] synthase II
Control vs DSS 6-day	2.3.1.180	Beta-ketoacyl-[acyl-carrier-protein] synthase III
Control vs DSS 6-day	2.3.1.181	Lipoyl(octanoyl) transferase
Control vs DSS 6-day	2.3.1.234	N(6)-L-threonylcarbamoyladenine synthase
Control vs DSS 6-day	2.3.1.29	Glycine C-acetyltransferase
Control vs DSS 6-day	2.3.1.31	Homoserine O-acetyltransferase
Control vs DSS 6-day	2.3.1.39	[Acyl-carrier-protein] S-malonyltransferase
Control vs DSS 6-day	2.3.1.47	8-amino-7-oxononanoate synthase
Control vs DSS 6-day	2.3.1.51	1-acylglycerol-3-phosphate O-acyltransferase
Control vs DSS 6-day	2.3.3.13	2-isopropylmalate synthase
Control vs DSS 6-day	2.4.1.18	1,4-alpha-glucan branching enzyme
Control vs DSS 6-day	2.4.1.21	Starch synthase
Control vs DSS 6-day	2.4.1.212	Hyaluronan synthase
Control vs DSS 6-day	2.4.1.320	1,4-beta-mannosyl-N-acetylglucosamine phosphorylase
Control vs DSS 6-day	2.4.2.10	Orotate phosphoribosyltransferase
Control vs DSS 6-day	2.4.2.14	Amidophosphoribosyltransferase
Control vs DSS 6-day	2.4.2.19	Nicotinate-nucleotide diphosphorylase (carboxylating)
Control vs DSS 6-day	2.4.2.29	tRNA-guanine(34) transglycosylase
Control vs DSS 6-day	2.4.2.8	Hypoxanthine phosphoribosyltransferase
Control vs DSS 6-day	2.4.99.17	S-adenosylmethionine
Control vs DSS 6-day	2.5.1.17	Cob(I)yrinic acid a,c-diamide adenosyltransferase
Control vs DSS 6-day	2.5.1.19	3-phosphoshikimate 1-carboxyvinyltransferase
Control vs DSS 6-day	2.5.1.47	Cysteine synthase
Control vs DSS 6-day	2.5.1.7	UDP-N-acetylglucosamine 1-carboxyvinyltransferase

Control vs DSS 6-day	2.5.1.72	Quinolinate synthase
Control vs DSS 6-day	2.5.1.78	6,7-dimethyl-8-ribityllumazine synthase
Control vs DSS 6-day	2.6.1.16	Glutamine--fructose-6-phosphate transaminase (isomerizing)
Control vs DSS 6-day	2.6.1.42	Branched-chain-amino-acid transaminase
Control vs DSS 6-day	2.6.1.52	Phosphoserine transaminase
Control vs DSS 6-day	2.6.1.83	LL-diaminopimelate aminotransferase
Control vs DSS 6-day	2.6.99.2	Pyridoxine 5'-phosphate synthase
Control vs DSS 6-day	2.7.1.11	6-phosphofructokinase
Control vs DSS 6-day	2.7.1.15	Ribokinase
Control vs DSS 6-day	2.7.1.21	Thymidine kinase
Control vs DSS 6-day	2.7.1.33	Pantothenate kinase
Control vs DSS 6-day	2.7.1.35	Pyridoxal kinase
Control vs DSS 6-day	2.7.1.40	Pyruvate kinase
Control vs DSS 6-day	2.7.1.48	Uridine kinase
Control vs DSS 6-day	2.7.1.5	Rhamnulokinase
Control vs DSS 6-day	2.7.1.69	Protein-N(pi)-phosphohistidine--sugar phosphotransferase
Control vs DSS 6-day	2.7.1.71	Shikimate kinase
Control vs DSS 6-day	2.7.1.90	Diphosphate--fructose-6-phosphate 1-phosphotransferase
Control vs DSS 6-day	2.7.13.3	Histidine kinase
Control vs DSS 6-day	2.7.2.1	Acetate kinase
Control vs DSS 6-day	2.7.2.3	Phosphoglycerate kinase
Control vs DSS 6-day	2.7.2.4	Aspartate kinase
Control vs DSS 6-day	2.7.4.25	(d)CMP kinase
Control vs DSS 6-day	2.7.4.3	Adenylate kinase
Control vs DSS 6-day	2.7.4.6	Nucleoside-diphosphate kinase
Control vs DSS 6-day	2.7.6.1	Ribose-phosphate diphosphokinase
Control vs DSS 6-day	2.7.7.13	Mannose-1-phosphate guanylyltransferase
Control vs DSS 6-day	2.7.7.6	DNA-directed RNA polymerase
Control vs DSS 6-day	2.7.7.63	Lipoate--protein ligase
Control vs DSS 6-day	2.7.7.7	DNA-directed DNA polymerase
Control vs DSS 6-day	2.7.7.72	CCA tRNA nucleotidyltransferase
Control vs DSS 6-day	2.7.7.8	Polyribonucleotide nucleotidyltransferase
Control vs DSS 6-day	2.7.7.85	Diadenylate cyclase
Control vs DSS 6-day	2.7.8.13	Phospho-N-acetylmuramoyl-pentapeptide-transferase
Control vs DSS 6-day	2.7.8.8	CDP-diacylglycerol--serine O-phosphatidyltransferase
Control vs DSS 6-day	2.8.4.3	tRNA-2-methylthio-N(6)-dimethylallyladenosine synthase
Control vs DSS 6-day	3.1.1.11	Pectinesterase
Control vs DSS 6-day	3.1.1.31	6-phosphogluconolactonase
Control vs DSS 6-day	3.1.13.1	Exoribonuclease II
Control vs DSS 6-day	3.1.21.3	Type I site-specific deoxyribonuclease
Control vs DSS 6-day	3.1.3.1	Alkaline phosphatase
Control vs DSS 6-day	3.1.3.11	Fructose-bisphosphatase
Control vs DSS 6-day	3.1.3.25	Inositol-phosphate phosphatase

Control vs DSS 6-day	3.1.3.5	5'-nucleotidase
Control vs DSS 6-day	3.1.6.1	Arylsulfatase
Control vs DSS 6-day	3.2.1.172	Unsaturated rhamnogalacturonyl hydrolase
Control vs DSS 6-day	3.2.1.177	Alpha-D-xyloside xylohydrolase
Control vs DSS 6-day	3.2.1.21	Beta-glucosidase
Control vs DSS 6-day	3.2.1.22	Alpha-galactosidase
Control vs DSS 6-day	3.2.1.23	Beta-galactosidase
Control vs DSS 6-day	3.2.1.37	Xylan 1,4-beta-xylosidase
Control vs DSS 6-day	3.2.1.55	Non-reducing end alpha-L-arabinofuranosidase
Control vs DSS 6-day	3.2.1.8	Endo-1,4-beta-xylanase
Control vs DSS 6-day	3.2.2.27	Uracil-DNA glycosylase
Control vs DSS 6-day	3.2.2.n1	Cytokinin riboside 5'-monophosphate phosphoribohydrolase
Control vs DSS 6-day	3.4.11.18	Methionyl aminopeptidase
Control vs DSS 6-day	3.4.11.9	Xaa-Pro aminopeptidase
Control vs DSS 6-day	3.4.13.18	Cytosol nonspecific dipeptidase
Control vs DSS 6-day	3.4.13.20	Beta-Ala-His dipeptidase
Control vs DSS 6-day	3.4.14.12	Xaa-Xaa-Pro tripeptidyl-peptidase
Control vs DSS 6-day	3.4.16.4	Serine-type D-Ala-D-Ala carboxypeptidase
Control vs DSS 6-day	3.4.21.105	Rhomboid protease
Control vs DSS 6-day	3.4.21.107	Peptidase Do
Control vs DSS 6-day	3.4.21.53	Endopeptidase La
Control vs DSS 6-day	3.4.21.89	Signal peptidase I
Control vs DSS 6-day	3.4.21.92	Endopeptidase Clp
Control vs DSS 6-day	3.4.23.36	Signal peptidase II
Control vs DSS 6-day	3.5.1.10	Formyltetrahydrofolate deformylase
Control vs DSS 6-day	3.5.1.100	(R)-amidase
Control vs DSS 6-day	3.5.1.108	UDP-3-O-acyl-N-acetylglucosamine deacetylase
Control vs DSS 6-day	3.5.1.28	N-acetylmuramoyl-L-alanine amidase
Control vs DSS 6-day	3.5.4.10	IMP cyclohydrolase
Control vs DSS 6-day	3.5.4.16	GTP cyclohydrolase I
Control vs DSS 6-day	3.5.4.9	Methenyltetrahydrofolate cyclohydrolase
Control vs DSS 6-day	3.5.99.6	Glucosamine-6-phosphate deaminase
Control vs DSS 6-day	3.6.3.31	Polyamine-transporting ATPase
Control vs DSS 6-day	3.6.3.41	Heme-transporting ATPase
Control vs DSS 6-day	3.6.3.44	Xenobiotic-transporting ATPase
Control vs DSS 6-day	3.6.3.54	Cu(+) exporting ATPase
Control vs DSS 6-day	3.6.4.12	DNA helicase
Control vs DSS 6-day	3.6.4.13	RNA helicase
Control vs DSS 6-day	4.1.1.11	Aspartate 1-decarboxylase
Control vs DSS 6-day	4.1.1.15	Glutamate decarboxylase
Control vs DSS 6-day	4.1.1.19	Arginine decarboxylase
Control vs DSS 6-day	4.1.1.20	Diaminopimelate decarboxylase
Control vs DSS 6-day	4.1.1.23	Orotidine-5'-phosphate decarboxylase

Control vs DSS 6-day	4.1.1.36	Phosphopantothenoylcysteine decarboxylase
Control vs DSS 6-day	4.1.1.49	Phosphoenolpyruvate carboxykinase (ATP)
Control vs DSS 6-day	4.1.1.65	Phosphatidylserine decarboxylase
Control vs DSS 6-day	4.1.2.50	6-carboxytetrahydropterin synthase
Control vs DSS 6-day	4.1.3.36	1,4-dihydroxy-2-naphthoyl-CoA synthase
Control vs DSS 6-day	4.2.1.10	3-dehydroquinate dehydratase
Control vs DSS 6-day	4.2.1.11	Phosphopyruvate hydratase
Control vs DSS 6-day	4.2.1.17	Enoyl-CoA hydratase
Control vs DSS 6-day	4.2.1.2	Fumarate hydratase
Control vs DSS 6-day	4.2.1.3	Aconitate hydratase
Control vs DSS 6-day	4.2.1.33	3-isopropylmalate dehydratase
Control vs DSS 6-day	4.2.1.46	dTDP-glucose 4,6-dehydratase
Control vs DSS 6-day	4.2.1.47	GDP-mannose 4,6-dehydratase
Control vs DSS 6-day	4.2.1.59	3-hydroxyacyl-[acyl-carrier-protein] dehydratase
Control vs DSS 6-day	4.2.1.8	Mannonate dehydratase
Control vs DSS 6-day	4.2.1.9	Dihydroxy-acid dehydratase
Control vs DSS 6-day	4.2.3.4	3-dehydroquinate synthase
Control vs DSS 6-day	4.2.3.5	Chorismate synthase
Control vs DSS 6-day	4.3.1.3	Histidine ammonia-lyase
Control vs DSS 6-day	4.3.1.4	Formimidoyltetrahydrofolate cyclodeaminase
Control vs DSS 6-day	4.3.2.1	Argininosuccinate lyase
Control vs DSS 6-day	4.3.2.2	Adenylosuccinate lyase
Control vs DSS 6-day	4.3.3.7	4-hydroxy-tetrahydrodipicolinate synthase
Control vs DSS 6-day	5.1.1.1	Alanine racemase
Control vs DSS 6-day	5.1.3.3	Aldose 1-epimerase
Control vs DSS 6-day	5.1.3.4	L-ribulose-5-phosphate 4-epimerase
Control vs DSS 6-day	5.2.1.8	Peptidylprolyl isomerase
Control vs DSS 6-day	5.3.1.1	Triose-phosphate isomerase
Control vs DSS 6-day	5.3.1.12	Glucuronate isomerase
Control vs DSS 6-day	5.3.1.14	L-rhamnose isomerase
Control vs DSS 6-day	5.3.1.17	5-dehydro-4-deoxy-D-glucuronate isomerase
Control vs DSS 6-day	5.3.1.25	L-fucose isomerase
Control vs DSS 6-day	5.3.1.4	L-arabinose isomerase
Control vs DSS 6-day	5.3.1.5	Xylose isomerase
Control vs DSS 6-day	5.3.1.9	Glucose-6-phosphate isomerase
Control vs DSS 6-day	5.3.3.2	Isopentenyl-diphosphate Delta-isomerase
Control vs DSS 6-day	5.4.2.12	Phosphoglycerate mutase (2,3-diphosphoglycerate-independent)
Control vs DSS 6-day	5.4.2.2	Phosphoglucomutase (alpha-D-glucose-1,6-bisphosphate-dependent)
Control vs DSS 6-day	5.99.1.2	DNA topoisomerase
Control vs DSS 6-day	5.99.1.3	DNA topoisomerase (ATP-hydrolyzing)
Control vs DSS 6-day	6.1.1.1	Tyrosine--tRNA ligase
Control vs DSS 6-day	6.1.1.10	Methionine--tRNA ligase
Control vs DSS 6-day	6.1.1.11	Serine--tRNA ligase

Control vs DSS 6-day	6.1.1.12	Aspartate--tRNA ligase
Control vs DSS 6-day	6.1.1.14	Glycine--tRNA ligase
Control vs DSS 6-day	6.1.1.15	Proline--tRNA ligase
Control vs DSS 6-day	6.1.1.17	Glutamate--tRNA ligase
Control vs DSS 6-day	6.1.1.18	Glutamine--tRNA ligase
Control vs DSS 6-day	6.1.1.19	Arginine--tRNA ligase
Control vs DSS 6-day	6.1.1.2	Tryptophan--tRNA ligase
Control vs DSS 6-day	6.1.1.20	Phenylalanine--tRNA ligase
Control vs DSS 6-day	6.1.1.21	Histidine--tRNA ligase
Control vs DSS 6-day	6.1.1.22	Asparagine--tRNA ligase
Control vs DSS 6-day	6.1.1.3	Threonine--tRNA ligase
Control vs DSS 6-day	6.1.1.4	Leucine--tRNA ligase
Control vs DSS 6-day	6.1.1.5	Isoleucine--tRNA ligase
Control vs DSS 6-day	6.1.1.6	Lysine--tRNA ligase
Control vs DSS 6-day	6.1.1.7	Alanine--tRNA ligase
Control vs DSS 6-day	6.1.1.9	Valine--tRNA ligase
Control vs DSS 6-day	6.2.1.30	Phenylacetate--CoA ligase
Control vs DSS 6-day	6.3.1.1	Aspartate--ammonia ligase
Control vs DSS 6-day	6.3.2.13	UDP-N-acetylmuramoyl-L-alanyl-D-glutamate--2,6-diaminopimelate ligase
Control vs DSS 6-day	6.3.2.4	D-alanine--D-alanine ligase
Control vs DSS 6-day	6.3.2.5	Phosphopantothenate--cysteine ligase
Control vs DSS 6-day	6.3.2.6	Phosphoribosylaminoimidazolesuccinocarboxamide synthase
Control vs DSS 6-day	6.3.2.9	UDP-N-acetylmuramoyl-L-alanine--D-glutamate ligase
Control vs DSS 6-day	6.3.4.13	Phosphoribosylamine--glycine ligase
Control vs DSS 6-day	6.3.4.14	Biotin carboxylase
Control vs DSS 6-day	6.3.4.2	CTP synthase (glutamine hydrolyzing)
Control vs DSS 6-day	6.3.5.2	GMP synthase (glutamine-hydrolyzing)
Control vs DSS 6-day	6.3.5.3	Phosphoribosylformylglycinamidine synthase
Control vs DSS 6-day	6.3.5.5	Carbamoyl-phosphate synthase (glutamine-hydrolyzing)
Control vs DSS 6-day	6.4.1.2	Acetyl-CoA carboxylase
Control vs DSS 6-day	6.5.1.2	DNA ligase (NAD(+))
Control vs DSS 6-day	7.1.2.2	NO_NAME
Control vs DSS 6-day	7.2.1.1	NO_NAME
Control vs DSS 6-day	1.1.1.37	Malate dehydrogenase
Control vs DSS 6-day	1.11.1.1	NADH peroxidase
Control vs DSS 6-day	1.17.4.1	Ribonucleoside-diphosphate reductase
Control vs DSS 6-day	1.2.1.11	Aspartate-semialdehyde dehydrogenase
Control vs DSS 6-day	1.2.1.12	Glyceraldehyde-3-phosphate dehydrogenase (phosphorylating)
Control vs DSS 6-day	1.4.1.3	Glutamate dehydrogenase (NAD(P)(+))
Control vs DSS 6-day	2.7.1.90	Diphosphate--fructose-6-phosphate 1-phosphotransferase
Control vs DSS 6-day	2.7.13.3	Histidine kinase
Control vs DSS 6-day	2.7.7.6	DNA-directed RNA polymerase
Control vs DSS 6-day	2.7.7.8	Polyribonucleotide nucleotidyltransferase

Control vs DSS 6-day	3.1.21.3	Type I site-specific deoxyribonuclease
Control vs DSS 6-day	3.6.4.12	DNA helicase
Control vs DSS 6-day	4.1.1.49	Phosphoenolpyruvate carboxykinase (ATP)
Control vs DSS 6-day	5.3.1.1	Triose-phosphate isomerase
Control vs DSS 6-day	5.3.1.12	Glucuronate isomerase
Control vs DSS 6-day	5.99.1.3	DNA topoisomerase (ATP-hydrolyzing)
Control vs DSS 6-day	6.1.1.22	Asparagine--tRNA ligase
Control vs DSS 6-day	6.1.1.7	Alanine--tRNA ligase
Control vs DSS 6-day	1.1.1.37	Malate dehydrogenase
Control vs DSS 6-day	1.11.1.1	NADH peroxidase
Control vs DSS 6-day	1.3.1.9	Enoyl-[acyl-carrier-protein] reductase (NADH)
Control vs DSS 6-day	2.1.1.72	Site-specific DNA-methyltransferase (adenine-specific)
Control vs DSS 6-day	2.7.1.11	6-phosphofructokinase
Control vs DSS 6-day	2.7.1.90	Diphosphate--fructose-6-phosphate 1-phosphotransferase
Control vs DSS 6-day	2.7.13.3	Histidine kinase
Control vs DSS 6-day	2.7.7.6	DNA-directed RNA polymerase
Control vs DSS 6-day	2.7.7.8	Polyribonucleotide nucleotidyltransferase
Control vs DSS 6-day	3.1.21.3	Type I site-specific deoxyribonuclease
Control vs DSS 6-day	3.6.4.12	DNA helicase
Control vs DSS 6-day	3.6.5.n1	Elongation factor 4
Control vs DSS 6-day	4.1.1.49	Phosphoenolpyruvate carboxykinase (ATP)
Control vs DSS 6-day	5.3.1.17	5-dehydro-4-deoxy-D-glucuronate isomerase
Control vs DSS 6-day	5.4.2.12	Phosphoglycerate mutase (2,3-diphosphoglycerate-independent)
Control vs DSS 6-day	5.99.1.3	DNA topoisomerase (ATP-hydrolyzing)
Control vs DSS 6-day	6.1.1.14	Glycine--tRNA ligase
Control vs DSS 6-day	6.1.1.3	Threonine--tRNA ligase
Control vs DSS 6-day	6.3.5.5	Carbamoyl-phosphate synthase (glutamine-hydrolyzing)
Control vs DSS 6-day	1.1.1.205	IMP dehydrogenase
Control vs DSS 6-day	1.1.1.37	Malate dehydrogenase
Control vs DSS 6-day	1.1.1.58	Tagaturonate reductase
Control vs DSS 6-day	1.1.1.86	Ketol-acid reductoisomerase (NADP(+))
Control vs DSS 6-day	1.11.1.1	NADH peroxidase
Control vs DSS 6-day	1.11.1.15	Peroxiredoxin
Control vs DSS 6-day	1.17.4.1	Ribonucleoside-diphosphate reductase
Control vs DSS 6-day	1.17.4.2	Ribonucleoside-triphosphate reductase
Control vs DSS 6-day	1.2.7.8	Indolepyruvate ferredoxin oxidoreductase
Control vs DSS 6-day	1.3.1.14	Dihydroorotate dehydrogenase (NAD(+))
Control vs DSS 6-day	1.4.1.16	Diaminopimelate dehydrogenase
Control vs DSS 6-day	1.4.1.2	Glutamate dehydrogenase
Control vs DSS 6-day	1.4.1.3	Glutamate dehydrogenase (NAD(P)(+))
Control vs DSS 6-day	1.4.3.16	L-aspartate oxidase
Control vs DSS 6-day	1.5.1.5	Methylenetetrahydrofolate dehydrogenase (NADP(+))
Control vs DSS 6-day	1.6.5.3	NADH

Control vs DSS 6-day	1.8.1.9	Thioredoxin-disulfide reductase
Control vs DSS 6-day	2.1.1.182	16S rRNA (adenine(1518)-N(6)/adenine(1519)-N(6))-dimethyltransferase
Control vs DSS 6-day	2.1.1.192	23S rRNA (adenine(2503)-C(2))-methyltransferase
Control vs DSS 6-day	2.1.1.37	DNA (cytosine-5-)-methyltransferase
Control vs DSS 6-day	2.1.1.72	Site-specific DNA-methyltransferase (adenine-specific)
Control vs DSS 6-day	2.1.2.1	Glycine hydroxymethyltransferase
Control vs DSS 6-day	2.1.2.11	3-methyl-2-oxobutanoate hydroxymethyltransferase
Control vs DSS 6-day	2.1.2.3	Phosphoribosylaminoimidazolecarboxamide formyltransferase
Control vs DSS 6-day	2.1.2.9	Methionyl-tRNA formyltransferase
Control vs DSS 6-day	2.1.3.2	Aspartate carbamoyltransferase
Control vs DSS 6-day	2.3.1.179	Beta-ketoacyl-[acyl-carrier-protein] synthase II
Control vs DSS 6-day	2.3.1.180	Beta-ketoacyl-[acyl-carrier-protein] synthase III
Control vs DSS 6-day	2.3.1.234	N(6)-L-threonylcarbamoyladenine synthase
Control vs DSS 6-day	2.3.1.47	8-amino-7-oxononanoate synthase
Control vs DSS 6-day	2.3.1.79	Maltose O-acetyltransferase
Control vs DSS 6-day	2.3.3.13	2-isopropylmalate synthase
Control vs DSS 6-day	2.4.1.18	1,4-alpha-glucan branching enzyme
Control vs DSS 6-day	2.4.1.21	Starch synthase
Control vs DSS 6-day	2.4.1.320	1,4-beta-mannosyl-N-acetylglucosamine phosphorylase
Control vs DSS 6-day	2.4.2.10	Orotate phosphoribosyltransferase
Control vs DSS 6-day	2.4.2.14	Amidophosphoribosyltransferase
Control vs DSS 6-day	2.4.2.19	Nicotinate-nucleotide diphosphorylase (carboxylating)
Control vs DSS 6-day	2.4.2.29	tRNA-guanine(34) transglycosylase
Control vs DSS 6-day	2.5.1.7	UDP-N-acetylglucosamine 1-carboxyvinyltransferase
Control vs DSS 6-day	2.5.1.78	6,7-dimethyl-8-ribityllumazine synthase
Control vs DSS 6-day	2.6.1.16	Glutamine--fructose-6-phosphate transaminase (isomerizing)
Control vs DSS 6-day	2.6.1.52	Phosphoserine transaminase
Control vs DSS 6-day	2.7.1.33	Pantothenate kinase
Control vs DSS 6-day	2.7.1.40	Pyruvate kinase
Control vs DSS 6-day	2.7.1.90	Diphosphate--fructose-6-phosphate 1-phosphotransferase
Control vs DSS 6-day	2.7.13.3	Histidine kinase
Control vs DSS 6-day	2.7.2.1	Acetate kinase
Control vs DSS 6-day	2.7.2.3	Phosphoglycerate kinase
Control vs DSS 6-day	2.7.2.4	Aspartate kinase
Control vs DSS 6-day	2.7.6.1	Ribose-phosphate diphosphokinase
Control vs DSS 6-day	2.7.7.33	Glucose-1-phosphate cytidylyltransferase
Control vs DSS 6-day	2.7.7.6	DNA-directed RNA polymerase
Control vs DSS 6-day	2.7.7.7	DNA-directed DNA polymerase
Control vs DSS 6-day	2.7.7.71	D-glycero-alpha-D-manno-heptose 1-phosphate guanylyltransferase
Control vs DSS 6-day	2.7.7.8	Polyribonucleotide nucleotidyltransferase
Control vs DSS 6-day	2.7.8.13	Phospho-N-acetylmuramoyl-pentapeptide-transferase
Control vs DSS 6-day	2.8.4.3	tRNA-2-methylthio-N(6)-dimethylallyladenosine synthase
Control vs DSS 6-day	3.1.21.3	Type I site-specific deoxyribonuclease

Control vs DSS 6-day	3.2.1.1	Alpha-amylase
Control vs DSS 6-day	3.2.1.14	Chitinase
Control vs DSS 6-day	3.4.11.18	Methionyl aminopeptidase
Control vs DSS 6-day	3.4.11.4	Tripeptide aminopeptidase
Control vs DSS 6-day	3.4.11.9	Xaa-Pro aminopeptidase
Control vs DSS 6-day	3.4.13.18	Cytosol nonspecific dipeptidase
Control vs DSS 6-day	3.4.21.107	Peptidase Do
Control vs DSS 6-day	3.4.21.53	Endopeptidase La
Control vs DSS 6-day	3.4.21.89	Signal peptidase I
Control vs DSS 6-day	3.4.21.92	Endopeptidase Clp
Control vs DSS 6-day	3.5.1.108	UDP-3-O-acyl-N-acetylglucosamine deacetylase
Control vs DSS 6-day	3.5.4.10	IMP cyclohydrolase
Control vs DSS 6-day	3.5.4.16	GTP cyclohydrolase I
Control vs DSS 6-day	3.5.4.9	Methenyltetrahydrofolate cyclohydrolase
Control vs DSS 6-day	3.6.3.31	Polyamine-transporting ATPase
Control vs DSS 6-day	3.6.4.12	DNA helicase
Control vs DSS 6-day	4.1.1.11	Aspartate 1-decarboxylase
Control vs DSS 6-day	4.1.1.15	Glutamate decarboxylase
Control vs DSS 6-day	4.1.1.19	Arginine decarboxylase
Control vs DSS 6-day	4.1.1.49	Phosphoenolpyruvate carboxykinase (ATP)
Control vs DSS 6-day	4.1.2.4	Deoxyribose-phosphate aldolase
Control vs DSS 6-day	4.1.3.36	1,4-dihydroxy-2-naphthoyl-CoA synthase
Control vs DSS 6-day	4.2.1.11	Phosphopyruvate hydratase
Control vs DSS 6-day	4.2.1.17	Enoyl-CoA hydratase
Control vs DSS 6-day	4.2.1.59	3-hydroxyacyl-[acyl-carrier-protein] dehydratase
Control vs DSS 6-day	4.2.3.5	Chorismate synthase
Control vs DSS 6-day	4.3.1.17	L-serine ammonia-lyase
Control vs DSS 6-day	5.2.1.8	Peptidylprolyl isomerase
Control vs DSS 6-day	5.3.1.1	Triose-phosphate isomerase
Control vs DSS 6-day	5.3.1.12	Glucuronate isomerase
Control vs DSS 6-day	5.3.1.14	L-rhamnose isomerase
Control vs DSS 6-day	5.3.1.17	5-dehydro-4-deoxy-D-glucuronate isomerase
Control vs DSS 6-day	5.3.1.25	L-fucose isomerase
Control vs DSS 6-day	5.3.1.4	L-arabinose isomerase
Control vs DSS 6-day	5.4.2.12	Phosphoglycerate mutase (2,3-diphosphoglycerate-independent)
Control vs DSS 6-day	5.4.2.2	Phosphoglucomutase (alpha-D-glucose-1,6-bisphosphate-dependent)
Control vs DSS 6-day	5.4.99.18	5-(carboxyamino)imidazole ribonucleotide mutase
Control vs DSS 6-day	5.99.1.3	DNA topoisomerase (ATP-hydrolyzing)
Control vs DSS 6-day	6.1.1.10	Methionine--tRNA ligase
Control vs DSS 6-day	6.1.1.11	Serine--tRNA ligase
Control vs DSS 6-day	6.1.1.12	Aspartate--tRNA ligase
Control vs DSS 6-day	6.1.1.14	Glycine--tRNA ligase
Control vs DSS 6-day	6.1.1.16	Cysteine--tRNA ligase

Control vs DSS 6-day	6.1.1.18	Glutamine--tRNA ligase
Control vs DSS 6-day	6.1.1.2	Tryptophan--tRNA ligase
Control vs DSS 6-day	6.1.1.20	Phenylalanine--tRNA ligase
Control vs DSS 6-day	6.1.1.22	Asparagine--tRNA ligase
Control vs DSS 6-day	6.1.1.4	Leucine--tRNA ligase
Control vs DSS 6-day	6.1.1.5	Isoleucine--tRNA ligase
Control vs DSS 6-day	6.1.1.6	Lysine--tRNA ligase
Control vs DSS 6-day	6.1.1.7	Alanine--tRNA ligase
Control vs DSS 6-day	6.3.2.6	Phosphoribosylaminoimidazolesuccinocarboxamide synthase
Control vs DSS 6-day	6.3.2.8	UDP-N-acetylmuramate--L-alanine ligase
Control vs DSS 6-day	6.3.4.13	Phosphoribosylamine--glycine ligase
Control vs DSS 6-day	6.3.4.2	CTP synthase (glutamine hydrolyzing)
Control vs DSS 6-day	6.3.5.3	Phosphoribosylformylglycinamidine synthase
Control vs DSS 6-day	6.3.5.5	Carbamoyl-phosphate synthase (glutamine-hydrolyzing)
Control vs DSS 6-day	6.5.1.2	DNA ligase (NAD(+))
Control vs DSS 6-day	7.1.2.2	NO_NAME
Control vs DSS 6-day	7.2.1.1	NO_NAME
Control vs DSS 6-day	1.1.1.100	3-oxoacyl-[acyl-carrier-protein] reductase
Control vs DSS 6-day	1.1.1.271	GDP-L-fucose synthase
Control vs DSS 6-day	1.1.1.3	Homoserine dehydrogenase
Control vs DSS 6-day	1.1.1.37	Malate dehydrogenase
Control vs DSS 6-day	1.1.1.86	Ketol-acid reductoisomerase (NADP(+))
Control vs DSS 6-day	1.1.1.95	Phosphoglycerate dehydrogenase
Control vs DSS 6-day	1.11.1.1	NADH peroxidase
Control vs DSS 6-day	1.11.1.15	Peroxiredoxin
Control vs DSS 6-day	1.15.1.1	Superoxide dismutase
Control vs DSS 6-day	1.16.3.2	Bacterial non-heme ferritin
Control vs DSS 6-day	1.2.1.11	Aspartate-semialdehyde dehydrogenase
Control vs DSS 6-day	1.3.1.9	Enoyl-[acyl-carrier-protein] reductase (NADH)
Control vs DSS 6-day	1.4.1.2	Glutamate dehydrogenase
Control vs DSS 6-day	1.4.1.3	Glutamate dehydrogenase (NAD(P)(+))
Control vs DSS 6-day	2.1.1.37	DNA (cytosine-5-)-methyltransferase
Control vs DSS 6-day	2.1.1.72	Site-specific DNA-methyltransferase (adenine-specific)
Control vs DSS 6-day	2.1.2.1	Glycine hydroxymethyltransferase
Control vs DSS 6-day	2.1.2.3	Phosphoribosylaminoimidazolecarboxamide formyltransferase
Control vs DSS 6-day	2.3.1.179	Beta-ketoacyl-[acyl-carrier-protein] synthase II
Control vs DSS 6-day	2.3.1.180	Beta-ketoacyl-[acyl-carrier-protein] synthase III
Control vs DSS 6-day	2.3.1.47	8-amino-7-oxononanoate synthase
Control vs DSS 6-day	2.3.1.50	Serine C-palmitoyltransferase
Control vs DSS 6-day	2.3.1.51	1-acylglycerol-3-phosphate O-acyltransferase
Control vs DSS 6-day	2.4.1.1	Glycogen phosphorylase
Control vs DSS 6-day	2.4.1.18	1,4-alpha-glucan branching enzyme
Control vs DSS 6-day	2.4.2.10	Orotate phosphoribosyltransferase

Control vs DSS 6-day	2.5.1.17	Cob(I)yrinic acid a,c-diamide adenosyltransferase
Control vs DSS 6-day	2.5.1.78	6,7-dimethyl-8-ribityllumazine synthase
Control vs DSS 6-day	2.6.1.16	Glutamine--fructose-6-phosphate transaminase (isomerizing)
Control vs DSS 6-day	2.6.1.42	Branched-chain-amino-acid transaminase
Control vs DSS 6-day	2.6.1.52	Phosphoserine transaminase
Control vs DSS 6-day	2.7.1.11	6-phosphofructokinase
Control vs DSS 6-day	2.7.1.90	Diphosphate--fructose-6-phosphate 1-phosphotransferase
Control vs DSS 6-day	2.7.13.3	Histidine kinase
Control vs DSS 6-day	2.7.2.3	Phosphoglycerate kinase
Control vs DSS 6-day	2.7.2.4	Aspartate kinase
Control vs DSS 6-day	2.7.6.1	Ribose-phosphate diphosphokinase
Control vs DSS 6-day	2.7.7.49	RNA-directed DNA polymerase
Control vs DSS 6-day	2.7.7.6	DNA-directed RNA polymerase
Control vs DSS 6-day	2.7.7.8	Polyribonucleotide nucleotidyltransferase
Control vs DSS 6-day	2.7.8.13	Phospho-N-acetylmuramoyl-pentapeptide-transferase
Control vs DSS 6-day	3.1.21.3	Type I site-specific deoxyribonuclease
Control vs DSS 6-day	3.1.3.25	Inositol-phosphate phosphatase
Control vs DSS 6-day	3.1.4.46	Glycerophosphodiester phosphodiesterase
Control vs DSS 6-day	3.4.11.18	Methionyl aminopeptidase
Control vs DSS 6-day	3.4.22.8	Clostripain
Control vs DSS 6-day	3.5.1.10	Formyltetrahydrofolate deformylase
Control vs DSS 6-day	3.5.1.108	UDP-3-O-acyl-N-acetylglucosamine deacetylase
Control vs DSS 6-day	3.5.4.10	IMP cyclohydrolase
Control vs DSS 6-day	3.5.4.25	GTP cyclohydrolase II
Control vs DSS 6-day	3.5.99.6	Glucosamine-6-phosphate deaminase
Control vs DSS 6-day	3.6.3.44	Xenobiotic-transporting ATPase
Control vs DSS 6-day	3.6.4.12	DNA helicase
Control vs DSS 6-day	3.6.5.n1	Elongation factor 4
Control vs DSS 6-day	4.1.1.15	Glutamate decarboxylase
Control vs DSS 6-day	4.1.1.19	Arginine decarboxylase
Control vs DSS 6-day	4.1.1.23	Orotidine-5'-phosphate decarboxylase
Control vs DSS 6-day	4.1.1.49	Phosphoenolpyruvate carboxykinase (ATP)
Control vs DSS 6-day	4.1.2.4	Deoxyribose-phosphate aldolase
Control vs DSS 6-day	4.1.99.12	3,4-dihydroxy-2-butanone-4-phosphate synthase
Control vs DSS 6-day	4.2.1.11	Phosphopyruvate hydratase
Control vs DSS 6-day	4.2.1.2	Fumarate hydratase
Control vs DSS 6-day	4.2.99.18	DNA-(apurinic or apyrimidinic site) lyase
Control vs DSS 6-day	4.3.2.1	Argininosuccinate lyase
Control vs DSS 6-day	5.1.3.3	Aldose 1-epimerase
Control vs DSS 6-day	5.2.1.8	Peptidylprolyl isomerase
Control vs DSS 6-day	5.3.1.1	Triose-phosphate isomerase
Control vs DSS 6-day	5.3.1.12	Glucuronate isomerase
Control vs DSS 6-day	5.3.1.5	Xylose isomerase

Control vs DSS 6-day	5.4.2.12	Phosphoglycerate mutase (2,3-diphosphoglycerate-independent)
Control vs DSS 6-day	5.99.1.3	DNA topoisomerase (ATP-hydrolyzing)
Control vs DSS 6-day	6.1.1.12	Aspartate--tRNA ligase
Control vs DSS 6-day	6.1.1.14	Glycine--tRNA ligase
Control vs DSS 6-day	6.1.1.15	Proline--tRNA ligase
Control vs DSS 6-day	6.1.1.20	Phenylalanine--tRNA ligase
Control vs DSS 6-day	6.1.1.22	Asparagine--tRNA ligase
Control vs DSS 6-day	6.1.1.3	Threonine--tRNA ligase
Control vs DSS 6-day	6.3.2.6	Phosphoribosylaminoimidazolesuccinocarboxamide synthase
Control vs DSS 6-day	6.3.4.2	CTP synthase (glutamine hydrolyzing)
Control vs DSS 6-day	6.3.4.4	Adenylosuccinate synthase
Control vs DSS 6-day	6.3.5.3	Phosphoribosylformylglycinamidine synthase
Control vs DSS 6-day	6.3.5.5	Carbamoyl-phosphate synthase (glutamine-hydrolyzing)
Control vs DSS 6-day	7.1.2.2	NO_NAME
Control vs DSS 6-day	7.2.1.1	NO_NAME
Control vs DSS 6-day	1.1.1.100	3-oxoacyl-[acyl-carrier-protein] reductase
Control vs DSS 6-day	1.1.1.122	D-threo-aldose 1-dehydrogenase
Control vs DSS 6-day	1.1.1.133	dTDP-4-dehydrorhamnose reductase
Control vs DSS 6-day	1.1.1.169	2-dehydropantoate 2-reductase
Control vs DSS 6-day	1.1.1.205	IMP dehydrogenase
Control vs DSS 6-day	1.1.1.22	UDP-glucose 6-dehydrogenase
Control vs DSS 6-day	1.1.1.262	4-hydroxythreonine-4-phosphate dehydrogenase
Control vs DSS 6-day	1.1.1.267	1-deoxy-D-xylulose-5-phosphate reductoisomerase
Control vs DSS 6-day	1.1.1.271	GDP-L-fucose synthase
Control vs DSS 6-day	1.1.1.28	D-lactate dehydrogenase
Control vs DSS 6-day	1.1.1.290	4-phosphoerythronate dehydrogenase
Control vs DSS 6-day	1.1.1.37	Malate dehydrogenase
Control vs DSS 6-day	1.1.1.40	Malate dehydrogenase (oxaloacetate-decarboxylating) (NADP(+))
Control vs DSS 6-day	1.1.1.42	Isocitrate dehydrogenase (NADP(+))
Control vs DSS 6-day	1.1.1.44	Phosphogluconate dehydrogenase (NADP(+)-dependent, decarboxylating)
Control vs DSS 6-day	1.1.1.49	Glucose-6-phosphate dehydrogenase (NADP(+))
Control vs DSS 6-day	1.1.1.58	Tagaturonate reductase
Control vs DSS 6-day	1.1.1.85	3-isopropylmalate dehydrogenase
Control vs DSS 6-day	1.1.1.86	Ketol-acid reductoisomerase (NADP(+))
Control vs DSS 6-day	1.1.1.94	Glycerol-3-phosphate dehydrogenase (NAD(P)(+))
Control vs DSS 6-day	1.1.1.95	Phosphoglycerate dehydrogenase
Control vs DSS 6-day	1.11.1.1	NADH peroxidase
Control vs DSS 6-day	1.11.1.15	Peroxiredoxin
Control vs DSS 6-day	1.15.1.1	Superoxide dismutase
Control vs DSS 6-day	1.16.3.2	Bacterial non-heme ferritin
Control vs DSS 6-day	1.17.4.1	Ribonucleoside-diphosphate reductase
Control vs DSS 6-day	1.17.4.2	Ribonucleoside-triphosphate reductase
Control vs DSS 6-day	1.17.7.3	(E)-4-hydroxy-3-methylbut-2-enyl-diphosphate synthase (flavodoxin)

Control vs DSS 6-day	1.2.1.11	Aspartate-semialdehyde dehydrogenase
Control vs DSS 6-day	1.2.1.38	N-acetyl-gamma-glutamyl-phosphate reductase
Control vs DSS 6-day	1.2.1.41	Glutamate-5-semialdehyde dehydrogenase
Control vs DSS 6-day	1.2.7.8	Indolepyruvate ferredoxin oxidoreductase
Control vs DSS 6-day	1.3.1.1	Dihydrouracil dehydrogenase (NAD(+))
Control vs DSS 6-day	1.3.1.14	Dihydroorotate dehydrogenase (NAD(+))
Control vs DSS 6-day	1.3.1.26	Transferred entry
Control vs DSS 6-day	1.3.1.9	Enoyl-[acyl-carrier-protein] reductase (NADH)
Control vs DSS 6-day	1.3.98.1	Dihydroorotate oxidase (fumarate)
Control vs DSS 6-day	1.3.99.5	3-oxo-5-alpha-steroid 4-dehydrogenase (acceptor)
Control vs DSS 6-day	1.4.1.13	Glutamate synthase (NADPH)
Control vs DSS 6-day	1.4.1.16	Diaminopimelate dehydrogenase
Control vs DSS 6-day	1.4.1.2	Glutamate dehydrogenase
Control vs DSS 6-day	1.4.1.21	Aspartate dehydrogenase
Control vs DSS 6-day	1.4.1.3	Glutamate dehydrogenase (NAD(P)(+))
Control vs DSS 6-day	1.4.3.16	L-aspartate oxidase
Control vs DSS 6-day	1.4.3.5	Pyridoxal 5'-phosphate synthase
Control vs DSS 6-day	1.4.4.2	Glycine dehydrogenase (aminomethyl-transferring)
Control vs DSS 6-day	1.5.1.2	Pyrroline-5-carboxylate reductase
Control vs DSS 6-day	1.5.1.20	Methylenetetrahydrofolate reductase (NAD(P)H)
Control vs DSS 6-day	1.5.1.3	Dihydrofolate reductase
Control vs DSS 6-day	1.5.1.39	FMN reductase (NAD(P)H)
Control vs DSS 6-day	1.5.1.5	Methylenetetrahydrofolate dehydrogenase (NADP(+))
Control vs DSS 6-day	1.6.5.11	NADH dehydrogenase (quinone)
Control vs DSS 6-day	1.6.5.3	NADH
Control vs DSS 6-day	1.6.99.1	NADPH dehydrogenase
Control vs DSS 6-day	1.7.1.13	PreQ(1) synthase
Control vs DSS 6-day	1.8.1.4	Dihydrolipoyl dehydrogenase
Control vs DSS 6-day	1.8.1.9	Thioredoxin-disulfide reductase
Control vs DSS 6-day	1.97.1.4	[Formate-C-acetyltransferase]-activating enzyme
Control vs DSS 6-day	2.1.1.13	Methionine synthase
Control vs DSS 6-day	2.1.1.163	Demethylmenaquinone methyltransferase
Control vs DSS 6-day	2.1.1.177	23S rRNA (pseudouridine(1915)-N(3))-methyltransferase
Control vs DSS 6-day	2.1.1.182	16S rRNA (adenine(1518)-N(6)/adenine(1519)-N(6))-dimethyltransferase
Control vs DSS 6-day	2.1.1.191	23S rRNA (cytosine(1962)-C(5))-methyltransferase
Control vs DSS 6-day	2.1.1.192	23S rRNA (adenine(2503)-C(2))-methyltransferase
Control vs DSS 6-day	2.1.1.193	16S rRNA (uracil(1498)-N(3))-methyltransferase
Control vs DSS 6-day	2.1.1.198	16S rRNA (cytidine(1402)-2'-O)-methyltransferase
Control vs DSS 6-day	2.1.1.199	16S rRNA (cytosine(1402)-N(4))-methyltransferase
Control vs DSS 6-day	2.1.1.228	tRNA (guanine(37)-N(1))-methyltransferase
Control vs DSS 6-day	2.1.1.33	tRNA (guanine(46)-N(7))-methyltransferase
Control vs DSS 6-day	2.1.1.45	Thymidylate synthase
Control vs DSS 6-day	2.1.1.63	Methylated-DNA--[protein]-cysteine S-methyltransferase

Control vs DSS 6-day	2.1.1.72	Site-specific DNA-methyltransferase (adenine-specific)
Control vs DSS 6-day	2.1.2.1	Glycine hydroxymethyltransferase
Control vs DSS 6-day	2.1.2.10	Aminomethyltransferase
Control vs DSS 6-day	2.1.2.2	Phosphoribosylglycinamide formyltransferase
Control vs DSS 6-day	2.1.2.3	Phosphoribosylaminoimidazolecarboxamide formyltransferase
Control vs DSS 6-day	2.1.2.9	Methionyl-tRNA formyltransferase
Control vs DSS 6-day	2.1.3.2	Aspartate carbamoyltransferase
Control vs DSS 6-day	2.1.3.9	N-acetylornithine carbamoyltransferase
Control vs DSS 6-day	2.2.1.1	Transketolase
Control vs DSS 6-day	2.2.1.6	Acetolactate synthase
Control vs DSS 6-day	2.2.1.7	1-deoxy-D-xylulose-5-phosphate synthase
Control vs DSS 6-day	2.2.1.9	synthase
Control vs DSS 6-day	2.3.1.129	Acyl-[acyl-carrier-protein]--UDP-N-acetylglucosamine O-acyltransferase
Control vs DSS 6-day	2.3.1.179	Beta-ketoacyl-[acyl-carrier-protein] synthase II
Control vs DSS 6-day	2.3.1.180	Beta-ketoacyl-[acyl-carrier-protein] synthase III
Control vs DSS 6-day	2.3.1.234	N(6)-L-threonylcarbamoyladenine synthase
Control vs DSS 6-day	2.3.1.28	Chloramphenicol O-acetyltransferase
Control vs DSS 6-day	2.3.1.29	Glycine C-acetyltransferase
Control vs DSS 6-day	2.3.1.31	Homoserine O-acetyltransferase
Control vs DSS 6-day	2.3.1.39	[Acyl-carrier-protein] S-malonyltransferase
Control vs DSS 6-day	2.3.1.47	8-amino-7-oxononanoate synthase
Control vs DSS 6-day	2.3.1.51	1-acylglycerol-3-phosphate O-acyltransferase
Control vs DSS 6-day	2.3.3.13	2-isopropylmalate synthase
Control vs DSS 6-day	2.4.1.18	1,4-alpha-glucan branching enzyme
Control vs DSS 6-day	2.4.1.21	Starch synthase
Control vs DSS 6-day	2.4.1.212	Hyaluronan synthase
Control vs DSS 6-day	2.4.1.320	1,4-beta-mannosyl-N-acetylglucosamine phosphorylase
Control vs DSS 6-day	2.4.2.1	Purine-nucleoside phosphorylase
Control vs DSS 6-day	2.4.2.10	Orotate phosphoribosyltransferase
Control vs DSS 6-day	2.4.2.14	Amidophosphoribosyltransferase
Control vs DSS 6-day	2.4.2.17	ATP phosphoribosyltransferase
Control vs DSS 6-day	2.4.2.19	Nicotinate-nucleotide diphosphorylase (carboxylating)
Control vs DSS 6-day	2.4.2.29	tRNA-guanine(34) transglycosylase
Control vs DSS 6-day	2.4.2.8	Hypoxanthine phosphoribosyltransferase
Control vs DSS 6-day	2.4.99.17	S-adenosylmethionine
Control vs DSS 6-day	2.5.1.15	Dihydropteroate synthase
Control vs DSS 6-day	2.5.1.17	Cob(I)yrinic acid a,c-diamide adenosyltransferase
Control vs DSS 6-day	2.5.1.19	3-phosphoshikimate 1-carboxyvinyltransferase
Control vs DSS 6-day	2.5.1.3	Thiamine-phosphate diphosphorylase
Control vs DSS 6-day	2.5.1.47	Cysteine synthase
Control vs DSS 6-day	2.5.1.6	Methionine adenosyltransferase
Control vs DSS 6-day	2.5.1.7	UDP-N-acetylglucosamine 1-carboxyvinyltransferase
Control vs DSS 6-day	2.5.1.72	Quinolinate synthase

Control vs DSS 6-day	2.5.1.75	tRNA dimethylallyltransferase
Control vs DSS 6-day	2.5.1.78	6,7-dimethyl-8-ribityllumazine synthase
Control vs DSS 6-day	2.6.1.1	Aspartate transaminase
Control vs DSS 6-day	2.6.1.16	Glutamine--fructose-6-phosphate transaminase (isomerizing)
Control vs DSS 6-day	2.6.1.42	Branched-chain-amino-acid transaminase
Control vs DSS 6-day	2.6.1.52	Phosphoserine transaminase
Control vs DSS 6-day	2.6.1.62	Adenosylmethionine--8-amino-7-oxononanoate transaminase
Control vs DSS 6-day	2.6.1.83	LL-diaminopimelate aminotransferase
Control vs DSS 6-day	2.6.1.9	Histidinol-phosphate transaminase
Control vs DSS 6-day	2.6.99.2	Pyridoxine 5'-phosphate synthase
Control vs DSS 6-day	2.7.1.11	6-phosphofructokinase
Control vs DSS 6-day	2.7.1.130	Tetraacyldisaccharide 4'-kinase
Control vs DSS 6-day	2.7.1.148	4-(cytidine 5'-diphospho)-2-C-methyl-D-erythritol kinase
Control vs DSS 6-day	2.7.1.15	Ribokinase
Control vs DSS 6-day	2.7.1.180	FAD
Control vs DSS 6-day	2.7.1.21	Thymidine kinase
Control vs DSS 6-day	2.7.1.26	Riboflavin kinase
Control vs DSS 6-day	2.7.1.33	Pantothenate kinase
Control vs DSS 6-day	2.7.1.35	Pyridoxal kinase
Control vs DSS 6-day	2.7.1.40	Pyruvate kinase
Control vs DSS 6-day	2.7.1.48	Uridine kinase
Control vs DSS 6-day	2.7.1.5	Rhamnulokinase
Control vs DSS 6-day	2.7.1.69	Protein-N(pi)-phosphohistidine--sugar phosphotransferase
Control vs DSS 6-day	2.7.1.71	Shikimate kinase
Control vs DSS 6-day	2.7.1.90	Diphosphate--fructose-6-phosphate 1-phosphotransferase
Control vs DSS 6-day	2.7.13.3	Histidine kinase
Control vs DSS 6-day	2.7.2.1	Acetate kinase
Control vs DSS 6-day	2.7.2.11	Glutamate 5-kinase
Control vs DSS 6-day	2.7.2.3	Phosphoglycerate kinase
Control vs DSS 6-day	2.7.2.4	Aspartate kinase
Control vs DSS 6-day	2.7.4.1	Polyphosphate kinase
Control vs DSS 6-day	2.7.4.16	Thiamine-phosphate kinase
Control vs DSS 6-day	2.7.4.25	(d)CMP kinase
Control vs DSS 6-day	2.7.4.3	Adenylate kinase
Control vs DSS 6-day	2.7.4.6	Nucleoside-diphosphate kinase
Control vs DSS 6-day	2.7.4.8	Guanylate kinase
Control vs DSS 6-day	2.7.6.1	Ribose-phosphate diphosphokinase
Control vs DSS 6-day	2.7.6.2	Thiamine diphosphokinase
Control vs DSS 6-day	2.7.6.3	2-amino-4-hydroxy-6-hydroxymethyldihydropteridine diphosphokinase
Control vs DSS 6-day	2.7.7.13	Mannose-1-phosphate guanylyltransferase
Control vs DSS 6-day	2.7.7.18	Nicotinate-nucleotide adenylyltransferase
Control vs DSS 6-day	2.7.7.2	FAD synthetase
Control vs DSS 6-day	2.7.7.24	Glucose-1-phosphate thymidylyltransferase

Control vs DSS 6-day	2.7.7.3	Pantetheine-phosphate adenylyltransferase
Control vs DSS 6-day	2.7.7.4	Sulfate adenylyltransferase
Control vs DSS 6-day	2.7.7.6	DNA-directed RNA polymerase
Control vs DSS 6-day	2.7.7.7	DNA-directed DNA polymerase
Control vs DSS 6-day	2.7.7.72	CCA tRNA nucleotidyltransferase
Control vs DSS 6-day	2.7.7.8	Polyribonucleotide nucleotidyltransferase
Control vs DSS 6-day	2.7.7.85	Diadenylate cyclase
Control vs DSS 6-day	2.7.7.87	L-threonylcarbamoyladenylate synthase
Control vs DSS 6-day	2.7.8.13	Phospho-N-acetylmuramoyl-pentapeptide-transferase
Control vs DSS 6-day	2.7.8.8	CDP-diacylglycerol--serine O-phosphatidyltransferase
Control vs DSS 6-day	2.7.9.1	Pyruvate, phosphate dikinase
Control vs DSS 6-day	2.8.1.6	Biotin synthase
Control vs DSS 6-day	2.8.1.7	Cysteine desulfurase
Control vs DSS 6-day	2.8.1.8	Lipoyl synthase
Control vs DSS 6-day	2.8.4.3	tRNA-2-methylthio-N(6)-dimethylallyladenosine synthase
Control vs DSS 6-day	2.8.4.4	[Ribosomal protein S12] (aspartate(89)-C(3))-methylthiotransferase
Control vs DSS 6-day	3.1.1.11	Pectinesterase
Control vs DSS 6-day	3.1.1.29	Aminoacyl-tRNA hydrolase
Control vs DSS 6-day	3.1.1.31	6-phosphogluconolactonase
Control vs DSS 6-day	3.1.1.96	D-aminoacyl-tRNA deacylase
Control vs DSS 6-day	3.1.11.2	Exodeoxyribonuclease III
Control vs DSS 6-day	3.1.11.6	Exodeoxyribonuclease VII
Control vs DSS 6-day	3.1.13.1	Exoribonuclease II
Control vs DSS 6-day	3.1.21.2	Deoxyribonuclease IV
Control vs DSS 6-day	3.1.21.3	Type I site-specific deoxyribonuclease
Control vs DSS 6-day	3.1.22.4	Crossover junction endodeoxyribonuclease
Control vs DSS 6-day	3.1.26.11	Ribonuclease Z
Control vs DSS 6-day	3.1.26.4	Ribonuclease H
Control vs DSS 6-day	3.1.3.1	Alkaline phosphatase
Control vs DSS 6-day	3.1.3.11	Fructose-bisphosphatase
Control vs DSS 6-day	3.1.3.15	Histidinol-phosphatase
Control vs DSS 6-day	3.1.3.25	Inositol-phosphate phosphatase
Control vs DSS 6-day	3.1.3.45	3-deoxy-manno-octulosonate-8-phosphatase
Control vs DSS 6-day	3.1.3.5	5'-nucleotidase
Control vs DSS 6-day	3.1.4.46	Glycerophosphodiester phosphodiesterase
Control vs DSS 6-day	3.1.6.1	Arylsulfatase
Control vs DSS 6-day	3.2.1.131	Xylan alpha-1,2-glucuronosidase
Control vs DSS 6-day	3.2.1.135	Neopullulanase
Control vs DSS 6-day	3.2.1.165	Exo-1,4-beta-D-glucosaminidase
Control vs DSS 6-day	3.2.1.172	Unsaturated rhamnogalacturonyl hydrolase
Control vs DSS 6-day	3.2.1.177	Alpha-D-xyloside xylohydrolase
Control vs DSS 6-day	3.2.1.21	Beta-glucosidase
Control vs DSS 6-day	3.2.1.22	Alpha-galactosidase

Control vs DSS 6-day	3.2.1.23	Beta-galactosidase
Control vs DSS 6-day	3.2.1.25	Beta-mannosidase
Control vs DSS 6-day	3.2.1.31	Beta-glucuronidase
Control vs DSS 6-day	3.2.1.37	Xylan 1,4-beta-xylosidase
Control vs DSS 6-day	3.2.1.51	Alpha-L-fucosidase
Control vs DSS 6-day	3.2.1.52	Beta-N-acetylhexosaminidase
Control vs DSS 6-day	3.2.1.55	Non-reducing end alpha-L-arabinofuranosidase
Control vs DSS 6-day	3.2.1.8	Endo-1,4-beta-xylanase
Control vs DSS 6-day	3.2.1.82	Exo-poly-alpha-galacturonosidase
Control vs DSS 6-day	3.2.2.27	Uracil-DNA glycosylase
Control vs DSS 6-day	3.2.2.n1	Cytokinin riboside 5'-monophosphate phosphoribohydrolase
Control vs DSS 6-day	3.3.1.1	Adenosylhomocysteinase
Control vs DSS 6-day	3.4.11.18	Methionyl aminopeptidase
Control vs DSS 6-day	3.4.11.4	Tripeptide aminopeptidase
Control vs DSS 6-day	3.4.13.18	Cytosol nonspecific dipeptidase
Control vs DSS 6-day	3.4.13.20	Beta-Ala-His dipeptidase
Control vs DSS 6-day	3.4.14.12	Xaa-Xaa-Pro tripeptidyl-peptidase
Control vs DSS 6-day	3.4.14.4	Dipeptidyl-peptidase III
Control vs DSS 6-day	3.4.15.5	Peptidyl-dipeptidase Dcp
Control vs DSS 6-day	3.4.16.4	Serine-type D-Ala-D-Ala carboxypeptidase
Control vs DSS 6-day	3.4.17.13	Muramoyltetrapeptide carboxypeptidase
Control vs DSS 6-day	3.4.21.102	C-terminal processing peptidase
Control vs DSS 6-day	3.4.21.105	Rhomboid protease
Control vs DSS 6-day	3.4.21.107	Peptidase Do
Control vs DSS 6-day	3.4.21.53	Endopeptidase La
Control vs DSS 6-day	3.4.21.89	Signal peptidase I
Control vs DSS 6-day	3.4.21.92	Endopeptidase Clp
Control vs DSS 6-day	3.4.23.36	Signal peptidase II
Control vs DSS 6-day	3.4.24.55	Pitrilysin
Control vs DSS 6-day	3.5.1.10	Formyltetrahydrofolate deformylase
Control vs DSS 6-day	3.5.1.100	(R)-amidase
Control vs DSS 6-day	3.5.1.104	Peptidoglycan-N-acetylglucosamine deacetylase
Control vs DSS 6-day	3.5.1.108	UDP-3-O-acyl-N-acetylglucosamine deacetylase
Control vs DSS 6-day	3.5.1.2	Glutaminase
Control vs DSS 6-day	3.5.1.25	N-acetylglucosamine-6-phosphate deacetylase
Control vs DSS 6-day	3.5.1.28	N-acetylmuramoyl-L-alanine amidase
Control vs DSS 6-day	3.5.1.88	Peptide deformylase
Control vs DSS 6-day	3.5.2.3	Dihydroorotase
Control vs DSS 6-day	3.5.2.7	Imidazolonepropionase
Control vs DSS 6-day	3.5.4.10	IMP cyclohydrolase
Control vs DSS 6-day	3.5.4.16	GTP cyclohydrolase I
Control vs DSS 6-day	3.5.4.19	Phosphoribosyl-AMP cyclohydrolase
Control vs DSS 6-day	3.5.4.25	GTP cyclohydrolase II

Control vs DSS 6-day	3.5.4.33	tRNA(adenine(34)) deaminase
Control vs DSS 6-day	3.5.4.9	Methenyltetrahydrofolate cyclohydrolase
Control vs DSS 6-day	3.5.99.6	Glucosamine-6-phosphate deaminase
Control vs DSS 6-day	3.6.1.27	Undecaprenyl-diphosphate phosphatase
Control vs DSS 6-day	3.6.1.31	Phosphoribosyl-ATP diphosphatase
Control vs DSS 6-day	3.6.1.66	XTP/dITP diphosphatase
Control vs DSS 6-day	3.6.3.31	Polyamine-transporting ATPase
Control vs DSS 6-day	3.6.3.41	Heme-transporting ATPase
Control vs DSS 6-day	3.6.3.44	Xenobiotic-transporting ATPase
Control vs DSS 6-day	3.6.3.54	Cu(+) exporting ATPase
Control vs DSS 6-day	3.6.4.12	DNA helicase
Control vs DSS 6-day	3.6.4.13	RNA helicase
Control vs DSS 6-day	3.6.5.n1	Elongation factor 4
Control vs DSS 6-day	4.1.1.11	Aspartate 1-decarboxylase
Control vs DSS 6-day	4.1.1.12	Aspartate 4-decarboxylase
Control vs DSS 6-day	4.1.1.15	Glutamate decarboxylase
Control vs DSS 6-day	4.1.1.19	Arginine decarboxylase
Control vs DSS 6-day	4.1.1.20	Diaminopimelate decarboxylase
Control vs DSS 6-day	4.1.1.23	Orotidine-5'-phosphate decarboxylase
Control vs DSS 6-day	4.1.1.36	Phosphopantothenoylcysteine decarboxylase
Control vs DSS 6-day	4.1.1.49	Phosphoenolpyruvate carboxykinase (ATP)
Control vs DSS 6-day	4.1.1.65	Phosphatidylserine decarboxylase
Control vs DSS 6-day	4.1.2.19	Rhamnulose-1-phosphate aldolase
Control vs DSS 6-day	4.1.2.4	Deoxyribose-phosphate aldolase
Control vs DSS 6-day	4.1.2.50	6-carboxytetrahydropterin synthase
Control vs DSS 6-day	4.1.3.36	1,4-dihydroxy-2-naphthoyl-CoA synthase
Control vs DSS 6-day	4.1.99.12	3,4-dihydroxy-2-butanone-4-phosphate synthase
Control vs DSS 6-day	4.2.1.10	3-dehydroquinate dehydratase
Control vs DSS 6-day	4.2.1.11	Phosphopyruvate hydratase
Control vs DSS 6-day	4.2.1.126	N-acetylmuramic acid 6-phosphate etherase
Control vs DSS 6-day	4.2.1.136	ADP-dependent NAD(P)H-hydrate dehydratase
Control vs DSS 6-day	4.2.1.17	Enoyl-CoA hydratase
Control vs DSS 6-day	4.2.1.19	Imidazoleglycerol-phosphate dehydratase
Control vs DSS 6-day	4.2.1.2	Fumarate hydratase
Control vs DSS 6-day	4.2.1.20	Tryptophan synthase
Control vs DSS 6-day	4.2.1.3	Aconitate hydratase
Control vs DSS 6-day	4.2.1.33	3-isopropylmalate dehydratase
Control vs DSS 6-day	4.2.1.46	dTDP-glucose 4,6-dehydratase
Control vs DSS 6-day	4.2.1.47	GDP-mannose 4,6-dehydratase
Control vs DSS 6-day	4.2.1.59	3-hydroxyacyl-[acyl-carrier-protein] dehydratase
Control vs DSS 6-day	4.2.1.8	Mannonate dehydratase
Control vs DSS 6-day	4.2.1.9	Dihydroxy-acid dehydratase
Control vs DSS 6-day	4.2.2.2	Pectate lyase

Control vs DSS 6-day	4.2.3.1	Threonine synthase
Control vs DSS 6-day	4.2.3.3	Methylglyoxal synthase
Control vs DSS 6-day	4.2.3.4	3-dehydroquinate synthase
Control vs DSS 6-day	4.2.3.5	Chorismate synthase
Control vs DSS 6-day	4.3.1.17	L-serine ammonia-lyase
Control vs DSS 6-day	4.3.1.3	Histidine ammonia-lyase
Control vs DSS 6-day	4.3.1.4	Formimidoyltetrahydrofolate cyclodeaminase
Control vs DSS 6-day	4.3.2.1	Argininosuccinate lyase
Control vs DSS 6-day	4.3.2.10	NO_NAME
Control vs DSS 6-day	4.3.2.2	Adenylosuccinate lyase
Control vs DSS 6-day	4.3.3.7	4-hydroxy-tetrahydrodipicolinate synthase
Control vs DSS 6-day	5.1.1.1	Alanine racemase
Control vs DSS 6-day	5.1.1.7	Diaminopimelate epimerase
Control vs DSS 6-day	5.1.3.1	Ribulose-phosphate 3-epimerase
Control vs DSS 6-day	5.1.3.13	dTDP-4-dehydrorhamnose 3,5-epimerase
Control vs DSS 6-day	5.1.3.3	Aldose 1-epimerase
Control vs DSS 6-day	5.1.3.32	L-rhamnose mutarotase
Control vs DSS 6-day	5.1.3.4	L-ribulose-5-phosphate 4-epimerase
Control vs DSS 6-day	5.1.99.6	NAD(P)H-hydrate epimerase
Control vs DSS 6-day	5.2.1.8	Peptidylprolyl isomerase
Control vs DSS 6-day	5.3.1.1	Triose-phosphate isomerase
Control vs DSS 6-day	5.3.1.12	Glucuronate isomerase
Control vs DSS 6-day	5.3.1.14	L-rhamnose isomerase
Control vs DSS 6-day	5.3.1.16	isomerase
Control vs DSS 6-day	5.3.1.17	5-dehydro-4-deoxy-D-glucuronate isomerase
Control vs DSS 6-day	5.3.1.25	L-fucose isomerase
Control vs DSS 6-day	5.3.1.4	L-arabinose isomerase
Control vs DSS 6-day	5.3.1.5	Xylose isomerase
Control vs DSS 6-day	5.3.1.9	Glucose-6-phosphate isomerase
Control vs DSS 6-day	5.3.3.2	Isopentenyl-diphosphate Delta-isomerase
Control vs DSS 6-day	5.4.2.10	Phosphoglucosamine mutase
Control vs DSS 6-day	5.4.2.12	Phosphoglycerate mutase (2,3-diphosphoglycerate-independent)
Control vs DSS 6-day	5.4.2.2	Phosphoglucomutase (alpha-D-glucose-1,6-bisphosphate-dependent)
Control vs DSS 6-day	5.4.2.8	Phosphomannomutase
Control vs DSS 6-day	5.4.99.18	5-(carboxyamino)imidazole ribonucleotide mutase
Control vs DSS 6-day	5.4.99.25	tRNA pseudouridine(55) synthase
Control vs DSS 6-day	5.99.1.2	DNA topoisomerase
Control vs DSS 6-day	5.99.1.3	DNA topoisomerase (ATP-hydrolyzing)
Control vs DSS 6-day	6.1.1.1	Tyrosine--tRNA ligase
Control vs DSS 6-day	6.1.1.10	Methionine--tRNA ligase
Control vs DSS 6-day	6.1.1.11	Serine--tRNA ligase
Control vs DSS 6-day	6.1.1.12	Aspartate--tRNA ligase
Control vs DSS 6-day	6.1.1.14	Glycine--tRNA ligase

Control vs DSS 6-day	6.1.1.15	Proline--tRNA ligase
Control vs DSS 6-day	6.1.1.16	Cysteine--tRNA ligase
Control vs DSS 6-day	6.1.1.17	Glutamate--tRNA ligase
Control vs DSS 6-day	6.1.1.18	Glutamine--tRNA ligase
Control vs DSS 6-day	6.1.1.19	Arginine--tRNA ligase
Control vs DSS 6-day	6.1.1.2	Tryptophan--tRNA ligase
Control vs DSS 6-day	6.1.1.20	Phenylalanine--tRNA ligase
Control vs DSS 6-day	6.1.1.21	Histidine--tRNA ligase
Control vs DSS 6-day	6.1.1.22	Asparagine--tRNA ligase
Control vs DSS 6-day	6.1.1.3	Threonine--tRNA ligase
Control vs DSS 6-day	6.1.1.4	Leucine--tRNA ligase
Control vs DSS 6-day	6.1.1.5	Isoleucine--tRNA ligase
Control vs DSS 6-day	6.1.1.6	Lysine--tRNA ligase
Control vs DSS 6-day	6.1.1.7	Alanine--tRNA ligase
Control vs DSS 6-day	6.1.1.9	Valine--tRNA ligase
Control vs DSS 6-day	6.2.1.30	Phenylacetate--CoA ligase
Control vs DSS 6-day	6.3.1.1	Aspartate--ammonia ligase
Control vs DSS 6-day	6.3.1.2	Glutamate--ammonia ligase
Control vs DSS 6-day	6.3.2.1	Pantoate--beta-alanine ligase (AMP-forming)
Control vs DSS 6-day	6.3.2.13	UDP-N-acetylmuramoyl-L-alanyl-D-glutamate--2,6-diaminopimelate ligase
Control vs DSS 6-day	6.3.2.4	D-alanine--D-alanine ligase
Control vs DSS 6-day	6.3.2.5	Phosphopantothenate--cysteine ligase
Control vs DSS 6-day	6.3.2.6	Phosphoribosylaminoimidazolesuccinocarboxamide synthase
Control vs DSS 6-day	6.3.2.8	UDP-N-acetylmuramate--L-alanine ligase
Control vs DSS 6-day	6.3.2.9	UDP-N-acetylmuramoyl-L-alanine--D-glutamate ligase
Control vs DSS 6-day	6.3.3.3	Dethiobiotin synthase
Control vs DSS 6-day	6.3.4.13	Phosphoribosylamine--glycine ligase
Control vs DSS 6-day	6.3.4.14	Biotin carboxylase
Control vs DSS 6-day	6.3.4.19	tRNA(Ile)-lysidine synthetase
Control vs DSS 6-day	6.3.4.2	CTP synthase (glutamine hydrolyzing)
Control vs DSS 6-day	6.3.4.3	Formate--tetrahydrofolate ligase
Control vs DSS 6-day	6.3.4.4	Adenylosuccinate synthase
Control vs DSS 6-day	6.3.5.1	NAD(+) synthase (glutamine-hydrolyzing)
Control vs DSS 6-day	6.3.5.2	GMP synthase (glutamine-hydrolyzing)
Control vs DSS 6-day	6.3.5.3	Phosphoribosylformylglycinamidine synthase
Control vs DSS 6-day	6.3.5.5	Carbamoyl-phosphate synthase (glutamine-hydrolyzing)
Control vs DSS 6-day	6.4.1.2	Acetyl-CoA carboxylase
Control vs DSS 6-day	6.4.1.3	Propionyl-CoA carboxylase
Control vs DSS 6-day	6.5.1.2	DNA ligase (NAD(+))
Control vs DSS 6-day	7.1.2.2	NO_NAME
Control vs DSS 6-day	7.2.1.1	NO_NAME
Control vs DSS 6-day	1.3.1.9	Enoyl-[acyl-carrier-protein] reductase (NADH)
Control vs DSS 6-day	1.4.1.2	Glutamate dehydrogenase

Control vs DSS 6-day	2.1.2.1	Glycine hydroxymethyltransferase
Control vs DSS 6-day	2.3.1.180	Beta-ketoacyl-[acyl-carrier-protein] synthase III
Control vs DSS 6-day	2.7.7.6	DNA-directed RNA polymerase
Control vs DSS 6-day	4.1.1.49	Phosphoenolpyruvate carboxykinase (ATP)
Control vs DSS 6-day	4.3.2.1	Argininosuccinate lyase
Control vs DSS 6-day	6.1.1.14	Glycine--tRNA ligase
Control vs DSS 6-day	1.7.99.1	Hydroxylamine reductase
Control vs DSS 6-day	2.7.13.3	Histidine kinase
Control vs DSS 6-day	4.1.1.49	Phosphoenolpyruvate carboxykinase (ATP)
Control vs DSS 6-day	2.7.6.1	Ribose-phosphate diphosphokinase
Control vs DSS 6-day	1.11.1.1	NADH peroxidase
Control vs DSS 6-day	2.7.9.1	Pyruvate, phosphate dikinase
Control vs DSS 6-day	2.7.9.1	Pyruvate, phosphate dikinase
Control vs DSS 6-day	4.1.1.49	Phosphoenolpyruvate carboxykinase (ATP)
Control vs DSS 6-day	1.11.1.1	NADH peroxidase
Control vs DSS 6-day	2.7.13.3	Histidine kinase
Control vs DSS 6-day	2.7.9.1	Pyruvate, phosphate dikinase
Control vs DSS 6-day	4.1.1.49	Phosphoenolpyruvate carboxykinase (ATP)
Control vs DSS 6-day	2.7.13.3	Histidine kinase
Control vs DSS 6-day	2.7.9.1	Pyruvate, phosphate dikinase
Control vs DSS 6-day	2.7.9.1	Pyruvate, phosphate dikinase
Control vs DSS 6-day	2.7.9.1	Pyruvate, phosphate dikinase
Control vs DSS 6-day	2.7.9.1	Pyruvate, phosphate dikinase
Control vs DSS 6-day	1.1.1.100	3-oxoacyl-[acyl-carrier-protein] reductase
Control vs DSS 6-day	1.1.1.205	IMP dehydrogenase
Control vs DSS 6-day	1.1.1.271	GDP-L-fucose synthase
Control vs DSS 6-day	1.1.1.37	Malate dehydrogenase
Control vs DSS 6-day	1.1.1.86	Ketol-acid reductoisomerase (NADP(+))
Control vs DSS 6-day	1.1.1.94	Glycerol-3-phosphate dehydrogenase (NAD(P)(+))
Control vs DSS 6-day	1.1.1.95	Phosphoglycerate dehydrogenase
Control vs DSS 6-day	1.11.1.1	NADH peroxidase
Control vs DSS 6-day	1.11.1.15	Peroxiredoxin
Control vs DSS 6-day	1.16.3.2	Bacterial non-heme ferritin
Control vs DSS 6-day	1.2.1.11	Aspartate-semialdehyde dehydrogenase
Control vs DSS 6-day	1.3.1.9	Enoyl-[acyl-carrier-protein] reductase (NADH)
Control vs DSS 6-day	1.4.1.2	Glutamate dehydrogenase
Control vs DSS 6-day	1.4.1.3	Glutamate dehydrogenase (NAD(P)(+))
Control vs DSS 6-day	2.1.1.198	16S rRNA (cytidine(1402)-2'-O)-methyltransferase
Control vs DSS 6-day	2.1.2.3	Phosphoribosylaminoimidazolecarboxamide formyltransferase
Control vs DSS 6-day	2.3.1.179	Beta-ketoacyl-[acyl-carrier-protein] synthase II
Control vs DSS 6-day	2.3.1.180	Beta-ketoacyl-[acyl-carrier-protein] synthase III
Control vs DSS 6-day	2.4.1.1	Glycogen phosphorylase
Control vs DSS 6-day	2.4.1.18	1,4-alpha-glucan branching enzyme

Control vs DSS 6-day	2.4.2.10	Orotate phosphoribosyltransferase
Control vs DSS 6-day	2.4.2.29	tRNA-guanine(34) transglycosylase
Control vs DSS 6-day	2.5.1.17	Cob(I)yrinic acid a,c-diamide adenosyltransferase
Control vs DSS 6-day	2.6.1.52	Phosphoserine transaminase
Control vs DSS 6-day	2.7.1.11	6-phosphofructokinase
Control vs DSS 6-day	2.7.1.40	Pyruvate kinase
Control vs DSS 6-day	2.7.1.90	Diphosphate--fructose-6-phosphate 1-phosphotransferase
Control vs DSS 6-day	2.7.13.3	Histidine kinase
Control vs DSS 6-day	2.7.2.3	Phosphoglycerate kinase
Control vs DSS 6-day	2.7.2.4	Aspartate kinase
Control vs DSS 6-day	2.7.6.1	Ribose-phosphate diphosphokinase
Control vs DSS 6-day	2.7.7.6	DNA-directed RNA polymerase
Control vs DSS 6-day	2.7.7.8	Polyribonucleotide nucleotidyltransferase
Control vs DSS 6-day	2.7.9.1	Pyruvate, phosphate dikinase
Control vs DSS 6-day	3.4.11.18	Methionyl aminopeptidase
Control vs DSS 6-day	3.5.1.10	Formyltetrahydrofolate deformylase
Control vs DSS 6-day	3.5.1.108	UDP-3-O-acyl-N-acetylglucosamine deacetylase
Control vs DSS 6-day	3.5.1.2	Glutaminase
Control vs DSS 6-day	3.5.1.24	Choloylglycine hydrolase
Control vs DSS 6-day	3.5.4.10	IMP cyclohydrolase
Control vs DSS 6-day	3.5.99.6	Glucosamine-6-phosphate deaminase
Control vs DSS 6-day	3.6.5.n1	Elongation factor 4
Control vs DSS 6-day	4.1.1.15	Glutamate decarboxylase
Control vs DSS 6-day	4.1.1.49	Phosphoenolpyruvate carboxykinase (ATP)
Control vs DSS 6-day	4.1.2.4	Deoxyribose-phosphate aldolase
Control vs DSS 6-day	4.2.1.11	Phosphopyruvate hydratase
Control vs DSS 6-day	4.2.1.59	3-hydroxyacyl-[acyl-carrier-protein] dehydratase
Control vs DSS 6-day	4.2.99.18	DNA-(apurinic or apyrimidinic site) lyase
Control vs DSS 6-day	5.1.3.3	Aldose 1-epimerase
Control vs DSS 6-day	5.2.1.8	Peptidylprolyl isomerase
Control vs DSS 6-day	5.3.1.1	Triose-phosphate isomerase
Control vs DSS 6-day	5.3.1.12	Glucuronate isomerase
Control vs DSS 6-day	5.3.1.5	Xylose isomerase
Control vs DSS 6-day	5.3.1.9	Glucose-6-phosphate isomerase
Control vs DSS 6-day	5.4.2.12	Phosphoglycerate mutase (2,3-diphosphoglycerate-independent)
Control vs DSS 6-day	5.99.1.3	DNA topoisomerase (ATP-hydrolyzing)
Control vs DSS 6-day	6.1.1.11	Serine--tRNA ligase
Control vs DSS 6-day	6.1.1.12	Aspartate--tRNA ligase
Control vs DSS 6-day	6.1.1.14	Glycine--tRNA ligase
Control vs DSS 6-day	6.1.1.20	Phenylalanine--tRNA ligase
Control vs DSS 6-day	6.1.1.22	Asparagine--tRNA ligase
Control vs DSS 6-day	6.3.1.1	Aspartate--ammonia ligase
Control vs DSS 6-day	6.3.2.6	Phosphoribosylaminoimidazolesuccinocarboxamide synthase

Control vs DSS 6-day	6.3.4.4	Adenylosuccinate synthase
Control vs DSS 6-day	6.3.5.3	Phosphoribosylformylglycinamidine synthase
Control vs DSS 6-day	6.3.5.5	Carbamoyl-phosphate synthase (glutamine-hydrolyzing)
Control vs DSS 6-day	7.1.2.2	NO_NAME
Control vs DSS 6-day	7.2.1.1	NO_NAME
Control vs DSS 6-day	2.7.13.3	Histidine kinase
Control vs DSS 6-day	3.1.21.3	Type I site-specific deoxyribonuclease
Control vs DSS 6-day	2.1.1.72	Site-specific DNA-methyltransferase (adenine-specific)
Control vs DSS 6-day	2.7.13.3	Histidine kinase
Control vs DSS 6-day	3.6.4.12	DNA helicase
Control vs DSS 6-day	2.7.9.1	Pyruvate, phosphate dikinase
Control vs DSS 6-day	3.6.3.20	Glycerol-3-phosphate-transporting ATPase
Control vs DSS 6-day	4.1.1.49	Phosphoenolpyruvate carboxykinase (ATP)
Control vs DSS 6-day	2.7.13.3	Histidine kinase
Control vs DSS 6-day	2.7.9.1	Pyruvate, phosphate dikinase
Control vs DSS 6-day	1.8.99.5	Dissimilatory sulfite reductase
Control vs DSS 6-day	2.7.9.1	Pyruvate, phosphate dikinase
Control vs DSS 6-day	4.1.1.49	Phosphoenolpyruvate carboxykinase (ATP)
Control vs DSS 6-day	1.11.1.1	NADH peroxidase
Control vs DSS 6-day	1.17.1.2	4-hydroxy-3-methylbut-2-enyl diphosphate reductase
Control vs DSS 6-day	2.7.13.3	Histidine kinase
Control vs DSS 6-day	2.7.9.1	Pyruvate, phosphate dikinase
Control vs DSS 6-day	3.6.3.20	Glycerol-3-phosphate-transporting ATPase
Control vs DSS 6-day	2.7.7.49	RNA-directed DNA polymerase
Control vs DSS 6-day	2.7.9.1	Pyruvate, phosphate dikinase
Control vs DSS 6-day	2.7.13.3	Histidine kinase
Control vs DSS 6-day	4.1.1.49	Phosphoenolpyruvate carboxykinase (ATP)
Control vs DSS 6-day	2.2.1.6	Acetolactate synthase
Control vs DSS 6-day	2.3.1.8	Phosphate acetyltransferase
Control vs DSS 6-day	2.4.1.1	Glycogen phosphorylase
Control vs DSS 6-day	2.7.1.23	NAD(+) kinase
Control vs DSS 6-day	2.7.1.40	Pyruvate kinase
Control vs DSS 6-day	2.7.1.69	Protein-N(pi)-phosphohistidine--sugar phosphotransferase
Control vs DSS 6-day	2.7.6.1	Ribose-phosphate diphosphokinase
Control vs DSS 6-day	2.7.7.6	DNA-directed RNA polymerase
Control vs DSS 6-day	2.8.4.4	[Ribosomal protein S12] (aspartate(89)-C(3))-methylthiotransferase
Control vs DSS 6-day	3.5.1.88	Peptide deformylase
Control vs DSS 6-day	3.5.99.6	Glucosamine-6-phosphate deaminase
Control vs DSS 6-day	4.2.1.11	Phosphopyruvate hydratase
Control vs DSS 6-day	4.2.1.46	dTDP-glucose 4,6-dehydratase
Control vs DSS 6-day	5.1.3.1	Ribulose-phosphate 3-epimerase
Control vs DSS 6-day	5.1.3.13	dTDP-4-dehydrorhamnose 3,5-epimerase
Control vs DSS 6-day	5.1.3.2	UDP-glucose 4-epimerase

Control vs DSS 6-day	5.2.1.8	Peptidylprolyl isomerase
Control vs DSS 6-day	5.3.1.8	Mannose-6-phosphate isomerase
Control vs DSS 6-day	5.4.2.12	Phosphoglycerate mutase (2,3-diphosphoglycerate-independent)
Control vs DSS 6-day	5.99.1.3	DNA topoisomerase (ATP-hydrolyzing)
Control vs DSS 6-day	6.1.1.2	Tryptophan--tRNA ligase
Control vs DSS 6-day	1.11.1.1	NADH peroxidase
Control vs DSS 6-day	2.1.1.72	Site-specific DNA-methyltransferase (adenine-specific)
Control vs DSS 6-day	3.1.21.3	Type I site-specific deoxyribonuclease
Control vs DSS 6-day	4.2.1.11	Phosphopyruvate hydratase
Control vs DSS 6-day	4.1.2.9	Phosphoketolase
Control vs DSS 6-day	4.2.1.11	Phosphopyruvate hydratase
Control vs DSS 6-day	1.1.1.157	3-hydroxybutyryl-CoA dehydrogenase
Control vs DSS 6-day	1.1.1.86	Ketol-acid reductoisomerase (NADP(+))
Control vs DSS 6-day	2.4.1.1	Glycogen phosphorylase
Control vs DSS 6-day	2.7.1.130	Tetraacyldisaccharide 4'-kinase
Control vs DSS 6-day	2.7.1.40	Pyruvate kinase
Control vs DSS 6-day	2.7.13.3	Histidine kinase
Control vs DSS 6-day	2.7.7.6	DNA-directed RNA polymerase
Control vs DSS 6-day	2.8.1.13	tRNA-uridine 2-sulfurtransferase
Control vs DSS 6-day	3.6.3.14	H(+)-transporting two-sector ATPase
Control vs DSS 6-day	4.1.1.49	Phosphoenolpyruvate carboxykinase (ATP)
Control vs DSS 6-day	4.1.99.17	Phosphomethylpyrimidine synthase
Control vs DSS 6-day	5.3.1.6	Ribose-5-phosphate isomerase
Control vs DSS 6-day	6.1.1.7	Alanine--tRNA ligase
Control vs DSS 6-day	1.11.1.15	Peroxiredoxin
Control vs DSS 6-day	1.11.1.15	Peroxiredoxin
Control vs DSS 6-day	1.1.1.27	L-lactate dehydrogenase
Control vs DSS 6-day	1.2.1.12	Glyceraldehyde-3-phosphate dehydrogenase (phosphorylating)
Control vs DSS 6-day	2.7.1.40	Pyruvate kinase
Control vs DSS 6-day	2.7.1.69	Protein-N(pi)-phosphohistidine--sugar phosphotransferase
Control vs DSS 6-day	2.7.2.1	Acetate kinase
Control vs DSS 6-day	2.7.2.3	Phosphoglycerate kinase
Control vs DSS 6-day	2.7.6.1	Ribose-phosphate diphosphokinase
Control vs DSS 6-day	2.7.7.6	DNA-directed RNA polymerase
Control vs DSS 6-day	3.2.1.122	Maltose-6'-phosphate glucosidase
Control vs DSS 6-day	3.2.1.86	6-phospho-beta-glucosidase
Control vs DSS 6-day	3.4.11.5	Prolyl aminopeptidase
Control vs DSS 6-day	3.4.19.3	Pyroglutamyl-peptidase I
Control vs DSS 6-day	4.1.2.40	Tagatose-bisphosphate aldolase
Control vs DSS 6-day	4.2.1.11	Phosphopyruvate hydratase
Control vs DSS 6-day	5.4.2.10	Phosphoglucosamine mutase
Control vs DSS 6-day	5.4.2.11	Phosphoglycerate mutase (2,3-diphosphoglycerate-dependent)
Control vs DSS 6-day	6.1.1.3	Threonine--tRNA ligase

Control vs DSS 6-day	6.3.1.2	Glutamate--ammonia ligase
Control vs DSS 6-day	7.1.2.2	NO_NAME
Control vs DSS 6-day	4.3.2.2	Adenylosuccinate lyase
Control vs DSS 6-day	6.3.5.2	GMP synthase (glutamine-hydrolyzing)
Control vs DSS 6-day	1.1.1.100	3-oxoacyl-[acyl-carrier-protein] reductase
Control vs DSS 6-day	1.1.1.133	dTDP-4-dehydrorhamnose reductase
Control vs DSS 6-day	1.1.1.218	Morphine 6-dehydrogenase
Control vs DSS 6-day	1.1.1.27	L-lactate dehydrogenase
Control vs DSS 6-day	1.1.1.28	D-lactate dehydrogenase
Control vs DSS 6-day	1.1.1.44	Phosphogluconate dehydrogenase (NADP(+)-dependent, decarboxylating)
Control vs DSS 6-day	1.1.1.49	Glucose-6-phosphate dehydrogenase (NADP(+))
Control vs DSS 6-day	1.1.1.88	Hydroxymethylglutaryl-CoA reductase
Control vs DSS 6-day	1.1.1.94	Glycerol-3-phosphate dehydrogenase (NAD(P)(+))
Control vs DSS 6-day	1.11.1.15	Peroxiredoxin
Control vs DSS 6-day	1.18.1.2	Ferredoxin--NADP(+) reductase
Control vs DSS 6-day	1.2.1.11	Aspartate-semialdehyde dehydrogenase
Control vs DSS 6-day	1.2.1.12	Glyceraldehyde-3-phosphate dehydrogenase (phosphorylating)
Control vs DSS 6-day	1.2.3.3	Pyruvate oxidase
Control vs DSS 6-day	1.3.1.98	UDP-N-acetylmuramate dehydrogenase
Control vs DSS 6-day	1.5.1.3	Dihydrofolate reductase
Control vs DSS 6-day	1.5.1.36	Flavin reductase (NADH)
Control vs DSS 6-day	1.5.1.5	Methylenetetrahydrofolate dehydrogenase (NADP(+))
Control vs DSS 6-day	1.7.1.7	GMP reductase
Control vs DSS 6-day	1.8.1.7	Glutathione-disulfide reductase
Control vs DSS 6-day	1.8.1.9	Thioredoxin-disulfide reductase
Control vs DSS 6-day	1.8.4.11	Peptide-methionine (S)-S-oxide reductase
Control vs DSS 6-day	1.8.4.12	Peptide-methionine (R)-S-oxide reductase
Control vs DSS 6-day	2.1.1.14	5-methyltetrahydropteroyltriglutamate--homocysteine S-methyltransferase
Control vs DSS 6-day	2.1.1.163	Demethylmenaquinone methyltransferase
Control vs DSS 6-day	2.1.1.182	16S rRNA (adenine(1518)-N(6)/adenine(1519)-N(6))-dimethyltransferase
Control vs DSS 6-day	2.1.1.198	16S rRNA (cytidine(1402)-2'-O)-methyltransferase
Control vs DSS 6-day	2.1.1.199	16S rRNA (cytosine(1402)-N(4))-methyltransferase
Control vs DSS 6-day	2.1.1.207	tRNA (cytidine(34)-2'-O)-methyltransferase
Control vs DSS 6-day	2.1.1.33	tRNA (guanine(46)-N(7))-methyltransferase
Control vs DSS 6-day	2.1.1.45	Thymidylate synthase
Control vs DSS 6-day	2.1.1.74	(FADH(2)-oxidizing)
Control vs DSS 6-day	2.1.2.1	Glycine hydroxymethyltransferase
Control vs DSS 6-day	2.1.2.9	Methionyl-tRNA formyltransferase
Control vs DSS 6-day	2.2.1.7	1-deoxy-D-xylulose-5-phosphate synthase
Control vs DSS 6-day	2.3.1.157	Glucosamine-1-phosphate N-acetyltransferase
Control vs DSS 6-day	2.3.1.234	N(6)-L-threonylcarbamoyladenine synthase
Control vs DSS 6-day	2.3.1.274	NO_NAME
Control vs DSS 6-day	2.3.1.8	Phosphate acetyltransferase

Control vs DSS 6-day	2.3.1.81	Aminoglycoside N(3')-acetyltransferase
Control vs DSS 6-day	2.3.1.9	Acetyl-CoA C-acetyltransferase
Control vs DSS 6-day	2.3.2.3	Lysyltransferase
Control vs DSS 6-day	2.4.1.227	acetylglucosaminyltransferase
Control vs DSS 6-day	2.4.2.22	Xanthine phosphoribosyltransferase
Control vs DSS 6-day	2.4.2.6	Nucleoside deoxyribosyltransferase
Control vs DSS 6-day	2.4.2.7	Adenine phosphoribosyltransferase
Control vs DSS 6-day	2.4.2.8	Hypoxanthine phosphoribosyltransferase
Control vs DSS 6-day	2.4.2.9	Uracil phosphoribosyltransferase
Control vs DSS 6-day	2.5.1.10	(2E,6E)-farnesyl diphosphate synthase
Control vs DSS 6-day	2.5.1.145	NO_NAME
Control vs DSS 6-day	2.5.1.30	Heptaprenyl diphosphate synthase
Control vs DSS 6-day	2.5.1.6	Methionine adenosyltransferase
Control vs DSS 6-day	2.5.1.7	UDP-N-acetylglucosamine 1-carboxyvinyltransferase
Control vs DSS 6-day	2.6.1.16	Glutamine--fructose-6-phosphate transaminase (isomerizing)
Control vs DSS 6-day	2.7.1.11	6-phosphofructokinase
Control vs DSS 6-day	2.7.1.113	Deoxyguanosine kinase
Control vs DSS 6-day	2.7.1.144	Tagatose-6-phosphate kinase
Control vs DSS 6-day	2.7.1.15	Ribokinase
Control vs DSS 6-day	2.7.1.180	FAD
Control vs DSS 6-day	2.7.1.23	NAD(+) kinase
Control vs DSS 6-day	2.7.1.24	Dephospho-CoA kinase
Control vs DSS 6-day	2.7.1.26	Riboflavin kinase
Control vs DSS 6-day	2.7.1.33	Pantothenate kinase
Control vs DSS 6-day	2.7.1.40	Pyruvate kinase
Control vs DSS 6-day	2.7.1.6	Galactokinase
Control vs DSS 6-day	2.7.1.69	Protein-N(pi)-phosphohistidine--sugar phosphotransferase
Control vs DSS 6-day	2.7.1.76	Deoxyadenosine kinase
Control vs DSS 6-day	2.7.11.1	Non-specific serine/threonine protein kinase
Control vs DSS 6-day	2.7.11.32	[Pyruvate, phosphate dikinase] kinase
Control vs DSS 6-day	2.7.13.3	Histidine kinase
Control vs DSS 6-day	2.7.2.1	Acetate kinase
Control vs DSS 6-day	2.7.2.3	Phosphoglycerate kinase
Control vs DSS 6-day	2.7.3.9	Phosphoenolpyruvate--protein phosphotransferase
Control vs DSS 6-day	2.7.4.22	UMP kinase
Control vs DSS 6-day	2.7.4.25	(d)CMP kinase
Control vs DSS 6-day	2.7.4.27	([Pyruvate, phosphate dikinase] phosphate) phosphotransferase
Control vs DSS 6-day	2.7.4.3	Adenylate kinase
Control vs DSS 6-day	2.7.4.8	Guanylate kinase
Control vs DSS 6-day	2.7.6.1	Ribose-phosphate diphosphokinase
Control vs DSS 6-day	2.7.6.2	Thiamine diphosphokinase
Control vs DSS 6-day	2.7.6.5	GTP diphosphokinase
Control vs DSS 6-day	2.7.7.12	UDP-glucose--hexose-1-phosphate uridylyltransferase

Control vs DSS 6-day	2.7.7.18	Nicotinate-nucleotide adenylyltransferase
Control vs DSS 6-day	2.7.7.2	FAD synthetase
Control vs DSS 6-day	2.7.7.23	UDP-N-acetylglucosamine diphosphorylase
Control vs DSS 6-day	2.7.7.24	Glucose-1-phosphate thymidylyltransferase
Control vs DSS 6-day	2.7.7.41	Phosphatidate cytidylyltransferase
Control vs DSS 6-day	2.7.7.6	DNA-directed RNA polymerase
Control vs DSS 6-day	2.7.7.7	DNA-directed DNA polymerase
Control vs DSS 6-day	2.7.7.72	CCA tRNA nucleotidyltransferase
Control vs DSS 6-day	2.7.7.85	Diadenylate cyclase
Control vs DSS 6-day	2.7.7.9	UTP--glucose-1-phosphate uridylyltransferase
Control vs DSS 6-day	2.7.8.13	Phospho-N-acetylmuramoyl-pentapeptide-transferase
Control vs DSS 6-day	2.7.8.5	CDP-diacylglycerol--glycerol-3-phosphate 3-phosphatidyltransferase
Control vs DSS 6-day	2.7.8.7	Holo-[acyl-carrier-protein] synthase
Control vs DSS 6-day	2.8.1.13	tRNA-uridine 2-sulfurtransferase
Control vs DSS 6-day	2.8.1.4	tRNA sulfurtransferase
Control vs DSS 6-day	2.8.1.7	Cysteine desulfurase
Control vs DSS 6-day	2.8.3.16	Formyl-CoA transferase
Control vs DSS 6-day	3.1.1.96	D-aminoacyl-tRNA deacylase
Control vs DSS 6-day	3.1.11.2	Exodeoxyribonuclease III
Control vs DSS 6-day	3.1.11.6	Exodeoxyribonuclease VII
Control vs DSS 6-day	3.1.13.1	Exoribonuclease II
Control vs DSS 6-day	3.1.22.4	Crossover junction endodeoxyribonuclease
Control vs DSS 6-day	3.1.26.11	Ribonuclease Z
Control vs DSS 6-day	3.1.26.3	Ribonuclease III
Control vs DSS 6-day	3.1.26.4	Ribonuclease H
Control vs DSS 6-day	3.1.26.5	Ribonuclease P
Control vs DSS 6-day	3.1.26.8	Ribonuclease M5
Control vs DSS 6-day	3.1.3.1	Alkaline phosphatase
Control vs DSS 6-day	3.1.3.16	Protein-serine/threonine phosphatase
Control vs DSS 6-day	3.1.3.23	Sugar-phosphatase
Control vs DSS 6-day	3.1.3.48	Protein-tyrosine-phosphatase
Control vs DSS 6-day	3.1.3.5	5'-nucleotidase
Control vs DSS 6-day	3.1.3.73	Adenosylcobalamin/alpha-ribazole phosphatase
Control vs DSS 6-day	3.1.4.46	Glycerophosphodiester phosphodiesterase
Control vs DSS 6-day	3.1.5.1	dGTPase
Control vs DSS 6-day	3.2.1.10	Oligo-1,6-glucosidase
Control vs DSS 6-day	3.2.1.122	Maltose-6'-phosphate glucosidase
Control vs DSS 6-day	3.2.1.135	Neopullulanase
Control vs DSS 6-day	3.2.1.17	Lysozyme
Control vs DSS 6-day	3.2.1.20	Alpha-glucosidase
Control vs DSS 6-day	3.2.1.23	Beta-galactosidase
Control vs DSS 6-day	3.2.1.26	Beta-fructofuranosidase
Control vs DSS 6-day	3.2.1.70	Glucan 1,6-alpha-glucosidase

Control vs DSS 6-day	3.2.1.85	6-phospho-beta-galactosidase
Control vs DSS 6-day	3.2.1.86	6-phospho-beta-glucosidase
Control vs DSS 6-day	3.2.2.1	Purine nucleosidase
Control vs DSS 6-day	3.2.2.20	DNA-3-methyladenine glycosylase I
Control vs DSS 6-day	3.2.2.23	DNA-formamidopyrimidine glycosylase
Control vs DSS 6-day	3.2.2.27	Uracil-DNA glycosylase
Control vs DSS 6-day	3.2.2.9	Adenosylhomocysteine nucleosidase
Control vs DSS 6-day	3.4.11.18	Methionyl aminopeptidase
Control vs DSS 6-day	3.4.11.4	Tripeptide aminopeptidase
Control vs DSS 6-day	3.4.11.5	Prolyl aminopeptidase
Control vs DSS 6-day	3.4.13.3	Transferred entry
Control vs DSS 6-day	3.4.13.9	Xaa-Pro dipeptidase
Control vs DSS 6-day	3.4.14.11	Xaa-Pro dipeptidyl-peptidase
Control vs DSS 6-day	3.4.19.3	Pyroglutamyl-peptidase I
Control vs DSS 6-day	3.4.21.102	C-terminal processing peptidase
Control vs DSS 6-day	3.4.21.88	Repressor LexA
Control vs DSS 6-day	3.4.21.89	Signal peptidase I
Control vs DSS 6-day	3.4.21.92	Endopeptidase Clp
Control vs DSS 6-day	3.4.22.40	Bleomycin hydrolase
Control vs DSS 6-day	3.4.23.36	Signal peptidase II
Control vs DSS 6-day	3.4.25.2	HslU--HslV peptidase
Control vs DSS 6-day	3.5.1.2	Glutaminase
Control vs DSS 6-day	3.5.1.24	Choloylglycine hydrolase
Control vs DSS 6-day	3.5.1.88	Peptide deformylase
Control vs DSS 6-day	3.5.4.9	Methenyltetrahydrofolate cyclohydrolase
Control vs DSS 6-day	3.5.99.6	Glucosamine-6-phosphate deaminase
Control vs DSS 6-day	3.6.1.1	Inorganic diphosphatase
Control vs DSS 6-day	3.6.1.13	ADP-ribose diphosphatase
Control vs DSS 6-day	3.6.1.66	XTP/dITP diphosphatase
Control vs DSS 6-day	3.6.1.7	Acylphosphatase
Control vs DSS 6-day	3.6.3.3	Cadmium-exporting ATPase
Control vs DSS 6-day	3.6.4.12	DNA helicase
Control vs DSS 6-day	3.6.5.3	Protein-synthesizing GTPase
Control vs DSS 6-day	3.6.5.n1	Elongation factor 4
Control vs DSS 6-day	4.1.1.31	Phosphoenolpyruvate carboxylase
Control vs DSS 6-day	4.1.1.33	Diphosphomevalonate decarboxylase
Control vs DSS 6-day	4.1.1.36	Phosphopantothenoylcysteine decarboxylase
Control vs DSS 6-day	4.1.1.5	Acetolactate decarboxylase
Control vs DSS 6-day	4.1.1.8	Oxalyl-CoA decarboxylase
Control vs DSS 6-day	4.1.2.4	Deoxyribose-phosphate aldolase
Control vs DSS 6-day	4.1.2.40	Tagatose-bisphosphate aldolase
Control vs DSS 6-day	4.2.1.11	Phosphopyruvate hydratase
Control vs DSS 6-day	4.2.1.136	ADP-dependent NAD(P)H-hydrate dehydratase

Control vs DSS 6-day	4.2.1.2	Fumarate hydratase
Control vs DSS 6-day	4.2.1.46	dTDP-glucose 4,6-dehydratase
Control vs DSS 6-day	4.2.99.18	DNA-(apurinic or apyrimidinic site) lyase
Control vs DSS 6-day	4.3.2.2	Adenylosuccinate lyase
Control vs DSS 6-day	4.4.1.21	S-ribosylhomocysteine lyase
Control vs DSS 6-day	5.1.1.1	Alanine racemase
Control vs DSS 6-day	5.1.1.13	Aspartate racemase
Control vs DSS 6-day	5.1.1.3	Glutamate racemase
Control vs DSS 6-day	5.1.1.7	Diaminopimelate epimerase
Control vs DSS 6-day	5.1.3.1	Ribulose-phosphate 3-epimerase
Control vs DSS 6-day	5.1.3.13	dTDP-4-dehydrorhamnose 3,5-epimerase
Control vs DSS 6-day	5.2.1.8	Peptidylprolyl isomerase
Control vs DSS 6-day	5.3.1.1	Triose-phosphate isomerase
Control vs DSS 6-day	5.3.1.26	Galactose-6-phosphate isomerase
Control vs DSS 6-day	5.3.1.6	Ribose-5-phosphate isomerase
Control vs DSS 6-day	5.3.1.8	Mannose-6-phosphate isomerase
Control vs DSS 6-day	5.3.1.9	Glucose-6-phosphate isomerase
Control vs DSS 6-day	5.3.3.2	Isopentenyl-diphosphate Delta-isomerase
Control vs DSS 6-day	5.4.2.10	Phosphoglucosamine mutase
Control vs DSS 6-day	5.4.2.11	Phosphoglycerate mutase (2,3-diphosphoglycerate-dependent)
Control vs DSS 6-day	5.4.2.2	Phosphoglucomutase (alpha-D-glucose-1,6-bisphosphate-dependent)
Control vs DSS 6-day	5.4.2.6	Beta-phosphoglucomutase
Control vs DSS 6-day	5.4.99.12	tRNA pseudouridine(38-40) synthase
Control vs DSS 6-day	5.4.99.62	D-ribose pyranase
Control vs DSS 6-day	5.99.1.2	DNA topoisomerase
Control vs DSS 6-day	5.99.1.3	DNA topoisomerase (ATP-hydrolyzing)
Control vs DSS 6-day	6.1.1.10	Methionine--tRNA ligase
Control vs DSS 6-day	6.1.1.11	Serine--tRNA ligase
Control vs DSS 6-day	6.1.1.12	Aspartate--tRNA ligase
Control vs DSS 6-day	6.1.1.14	Glycine--tRNA ligase
Control vs DSS 6-day	6.1.1.15	Proline--tRNA ligase
Control vs DSS 6-day	6.1.1.17	Glutamate--tRNA ligase
Control vs DSS 6-day	6.1.1.2	Tryptophan--tRNA ligase
Control vs DSS 6-day	6.1.1.20	Phenylalanine--tRNA ligase
Control vs DSS 6-day	6.1.1.21	Histidine--tRNA ligase
Control vs DSS 6-day	6.1.1.22	Asparagine--tRNA ligase
Control vs DSS 6-day	6.1.1.3	Threonine--tRNA ligase
Control vs DSS 6-day	6.1.1.5	Isoleucine--tRNA ligase
Control vs DSS 6-day	6.1.1.6	Lysine--tRNA ligase
Control vs DSS 6-day	6.1.1.7	Alanine--tRNA ligase
Control vs DSS 6-day	6.1.1.9	Valine--tRNA ligase
Control vs DSS 6-day	6.3.1.2	Glutamate--ammonia ligase
Control vs DSS 6-day	6.3.1.5	NAD(+) synthase

Control vs DSS 6-day	6.3.2.10	UDP-N-acetylmuramoyl-tripeptide--D-alanyl-D-alanine ligase
Control vs DSS 6-day	6.3.2.4	D-alanine--D-alanine ligase
Control vs DSS 6-day	6.3.2.5	Phosphopantothenate--cysteine ligase
Control vs DSS 6-day	6.3.2.8	UDP-N-acetylmuramate--L-alanine ligase
Control vs DSS 6-day	6.3.2.9	UDP-N-acetylmuramoyl-L-alanine--D-glutamate ligase
Control vs DSS 6-day	6.3.3.2	5-formyltetrahydrofolate cyclo-ligase
Control vs DSS 6-day	6.3.4.19	tRNA(Ile)-lysidine synthetase
Control vs DSS 6-day	6.3.4.2	CTP synthase (glutamine hydrolyzing)
Control vs DSS 6-day	6.3.4.21	Nicotinate phosphoribosyltransferase
Control vs DSS 6-day	6.3.4.3	Formate--tetrahydrofolate ligase
Control vs DSS 6-day	6.3.4.4	Adenylosuccinate synthase
Control vs DSS 6-day	6.3.5.2	GMP synthase (glutamine-hydrolyzing)
Control vs DSS 6-day	6.3.5.4	Asparagine synthase (glutamine-hydrolyzing)
Control vs DSS 6-day	6.3.5.5	Carbamoyl-phosphate synthase (glutamine-hydrolyzing)
Control vs DSS 6-day	6.3.5.7	Glutaminyl-tRNA synthase (glutamine-hydrolyzing)
Control vs DSS 6-day	6.5.1.2	DNA ligase (NAD(+))
Control vs DSS 6-day	7.1.2.2	NO_NAME
Control vs DSS 6-day	7.3.2.1	NO_NAME
Control vs DSS 6-day	7.3.2.2	NO_NAME
Control vs DSS 6-day	1.1.1.44	Phosphogluconate dehydrogenase (NADP(+)-dependent, decarboxylating)
Control vs DSS 6-day	1.2.1.12	Glyceraldehyde-3-phosphate dehydrogenase (phosphorylating)
Control vs DSS 6-day	2.7.2.3	Phosphoglycerate kinase
Control vs DSS 6-day	2.7.7.6	DNA-directed RNA polymerase
Control vs DSS 6-day	3.1.13.1	Exoribonuclease II
Control vs DSS 6-day	3.2.1.122	Maltose-6'-phosphate glucosidase
Control vs DSS 6-day	3.4.19.3	Pyroglutamyl-peptidase I
Control vs DSS 6-day	3.4.22.40	Bleomycin hydrolase
Control vs DSS 6-day	4.1.2.40	Tagatose-bisphosphate aldolase
Control vs DSS 6-day	4.2.1.11	Phosphopyruvate hydratase
Control vs DSS 6-day	5.4.2.11	Phosphoglycerate mutase (2,3-diphosphoglycerate-dependent)
Control vs DSS 6-day	6.1.1.22	Asparagine--tRNA ligase
Control vs DSS 6-day	6.3.1.2	Glutamate--ammonia ligase
Control vs DSS 6-day	1.1.1.133	dTDP-4-dehydrorhamnose reductase
Control vs DSS 6-day	1.1.1.27	L-lactate dehydrogenase
Control vs DSS 6-day	1.1.1.28	D-lactate dehydrogenase
Control vs DSS 6-day	1.2.1.12	Glyceraldehyde-3-phosphate dehydrogenase (phosphorylating)
Control vs DSS 6-day	1.7.1.7	GMP reductase
Control vs DSS 6-day	2.2.1.7	1-deoxy-D-xylulose-5-phosphate synthase
Control vs DSS 6-day	2.5.1.30	Heptaprenyl diphosphate synthase
Control vs DSS 6-day	2.7.1.144	Tagatose-6-phosphate kinase
Control vs DSS 6-day	2.7.1.26	Riboflavin kinase
Control vs DSS 6-day	2.7.1.40	Pyruvate kinase
Control vs DSS 6-day	2.7.1.6	Galactokinase

Control vs DSS 6-day	2.7.1.69	Protein-N(pi)-phosphohistidine--sugar phosphotransferase
Control vs DSS 6-day	2.7.10.1	Receptor protein-tyrosine kinase
Control vs DSS 6-day	2.7.13.3	Histidine kinase
Control vs DSS 6-day	2.7.3.9	Phosphoenolpyruvate--protein phosphotransferase
Control vs DSS 6-day	2.7.6.1	Ribose-phosphate diphosphokinase
Control vs DSS 6-day	2.7.7.12	UDP-glucose--hexose-1-phosphate uridylyltransferase
Control vs DSS 6-day	2.7.7.2	FAD synthetase
Control vs DSS 6-day	2.7.7.6	DNA-directed RNA polymerase
Control vs DSS 6-day	2.7.7.7	DNA-directed DNA polymerase
Control vs DSS 6-day	3.1.13.1	Exoribonuclease II
Control vs DSS 6-day	3.1.3.1	Alkaline phosphatase
Control vs DSS 6-day	3.1.3.23	Sugar-phosphatase
Control vs DSS 6-day	3.1.3.48	Protein-tyrosine-phosphatase
Control vs DSS 6-day	3.1.3.5	5'-nucleotidase
Control vs DSS 6-day	3.2.1.10	Oligo-1,6-glucosidase
Control vs DSS 6-day	3.2.1.135	Neopullulanase
Control vs DSS 6-day	3.2.1.20	Alpha-glucosidase
Control vs DSS 6-day	3.2.1.23	Beta-galactosidase
Control vs DSS 6-day	3.2.1.26	Beta-fructofuranosidase
Control vs DSS 6-day	3.2.1.86	6-phospho-beta-glucosidase
Control vs DSS 6-day	3.4.11.5	Prolyl aminopeptidase
Control vs DSS 6-day	3.4.14.11	Xaa-Pro dipeptidyl-peptidase
Control vs DSS 6-day	3.4.22.40	Bleomycin hydrolase
Control vs DSS 6-day	3.5.1.2	Glutaminase
Control vs DSS 6-day	3.6.4.12	DNA helicase
Control vs DSS 6-day	4.2.1.11	Phosphopyruvate hydratase
Control vs DSS 6-day	4.2.1.136	ADP-dependent NAD(P)H-hydrate dehydratase
Control vs DSS 6-day	4.2.1.46	dTDP-glucose 4,6-dehydratase
Control vs DSS 6-day	5.2.1.8	Peptidylprolyl isomerase
Control vs DSS 6-day	5.4.2.11	Phosphoglycerate mutase (2,3-diphosphoglycerate-dependent)
Control vs DSS 6-day	5.4.2.6	Beta-phosphoglucomutase
Control vs DSS 6-day	6.1.1.14	Glycine--tRNA ligase
Control vs DSS 6-day	6.1.1.20	Phenylalanine--tRNA ligase
Control vs DSS 6-day	6.1.1.22	Asparagine--tRNA ligase
Control vs DSS 6-day	6.1.1.3	Threonine--tRNA ligase
Control vs DSS 6-day	6.1.1.7	Alanine--tRNA ligase
Control vs DSS 6-day	6.3.4.21	Nicotinate phosphoribosyltransferase
Control vs DSS 6-day	6.3.5.4	Asparagine synthase (glutamine-hydrolyzing)
Control vs DSS 6-day	6.3.5.5	Carbamoyl-phosphate synthase (glutamine-hydrolyzing)
Control vs DSS 6-day	7.1.2.2	NO_NAME
Control vs DSS 6-day	4.1.1.49	Phosphoenolpyruvate carboxykinase (ATP)
Control vs DSS 6-day	4.2.1.11	Phosphopyruvate hydratase
Control vs DSS 6-day	4.1.1.49	Phosphoenolpyruvate carboxykinase (ATP)

Control vs DSS 6-day	1.1.1.133	dTDP-4-dehydrorhamnose reductase
Control vs DSS 6-day	1.1.1.193	5-amino-6-(5-phosphoribosylamino)uracil reductase
Control vs DSS 6-day	1.1.1.205	IMP dehydrogenase
Control vs DSS 6-day	1.1.1.23	Histidinol dehydrogenase
Control vs DSS 6-day	1.1.1.25	Shikimate dehydrogenase
Control vs DSS 6-day	1.1.1.267	1-deoxy-D-xylulose-5-phosphate reductoisomerase
Control vs DSS 6-day	1.1.1.3	Homoserine dehydrogenase
Control vs DSS 6-day	1.1.1.37	Malate dehydrogenase
Control vs DSS 6-day	1.1.1.42	Isocitrate dehydrogenase (NADP(+))
Control vs DSS 6-day	1.1.1.85	3-isopropylmalate dehydrogenase
Control vs DSS 6-day	1.1.1.86	Ketol-acid reductoisomerase (NADP(+))
Control vs DSS 6-day	1.1.1.94	Glycerol-3-phosphate dehydrogenase (NAD(P)(+))
Control vs DSS 6-day	1.1.1.95	Phosphoglycerate dehydrogenase
Control vs DSS 6-day	1.11.1.15	Peroxiredoxin
Control vs DSS 6-day	1.11.1.6	Catalase
Control vs DSS 6-day	1.17.1.8	4-hydroxy-tetrahydrodipicolinate reductase
Control vs DSS 6-day	1.17.4.1	Ribonucleoside-diphosphate reductase
Control vs DSS 6-day	1.17.7.3	(E)-4-hydroxy-3-methylbut-2-enyl-diphosphate synthase (flavodoxin)
Control vs DSS 6-day	1.17.7.4	NO_NAME
Control vs DSS 6-day	1.17.99.6	Epoxyqueuosine reductase
Control vs DSS 6-day	1.2.1.11	Aspartate-semialdehyde dehydrogenase
Control vs DSS 6-day	1.2.1.38	N-acetyl-gamma-glutamyl-phosphate reductase
Control vs DSS 6-day	1.2.1.41	Glutamate-5-semialdehyde dehydrogenase
Control vs DSS 6-day	1.2.1.70	Glutamyl-tRNA reductase
Control vs DSS 6-day	1.21.98.1	Cyclic dehypoxanthinyl futalosine synthase
Control vs DSS 6-day	1.3.1.76	Precorrin-2 dehydrogenase
Control vs DSS 6-day	1.3.1.9	Enoyl-[acyl-carrier-protein] reductase (NADH)
Control vs DSS 6-day	1.3.3.4	Protoporphyrinogen oxidase
Control vs DSS 6-day	1.3.5.1	Succinate dehydrogenase (quinone)
Control vs DSS 6-day	1.4.1.16	Diaminopimelate dehydrogenase
Control vs DSS 6-day	1.5.1.2	Pyrroline-5-carboxylate reductase
Control vs DSS 6-day	1.5.1.20	Methylenetetrahydrofolate reductase (NAD(P)H)
Control vs DSS 6-day	1.5.1.5	Methylenetetrahydrofolate dehydrogenase (NADP(+))
Control vs DSS 6-day	1.7.2.2	Nitrite reductase (cytochrome; ammonia-forming)
Control vs DSS 6-day	1.8.1.9	Thioredoxin-disulfide reductase
Control vs DSS 6-day	1.8.4.11	Peptide-methionine (S)-S-oxide reductase
Control vs DSS 6-day	1.8.4.12	Peptide-methionine (R)-S-oxide reductase
Control vs DSS 6-day	2.1.1.107	Uroporphyrinogen-III C-methyltransferase
Control vs DSS 6-day	2.1.1.13	Methionine synthase
Control vs DSS 6-day	2.1.1.148	Thymidylate synthase (FAD)
Control vs DSS 6-day	2.1.1.163	Demethylmenaquinone methyltransferase
Control vs DSS 6-day	2.1.1.166	23S rRNA (uridine(2552)-2'-O)-methyltransferase
Control vs DSS 6-day	2.1.1.177	23S rRNA (pseudouridine(1915)-N(3))-methyltransferase

Control vs DSS 6-day	2.1.1.182	16S rRNA (adenine(1518)-N(6)/adenine(1519)-N(6))-dimethyltransferase
Control vs DSS 6-day	2.1.1.192	23S rRNA (adenine(2503)-C(2))-methyltransferase
Control vs DSS 6-day	2.1.1.193	16S rRNA (uracil(1498)-N(3))-methyltransferase
Control vs DSS 6-day	2.1.1.195	Cobalt-precorrin-5B (C(1))-methyltransferase
Control vs DSS 6-day	2.1.1.198	16S rRNA (cytidine(1402)-2'-O)-methyltransferase
Control vs DSS 6-day	2.1.1.199	16S rRNA (cytosine(1402)-N(4))-methyltransferase
Control vs DSS 6-day	2.1.1.207	tRNA (cytidine(34)-2'-O)-methyltransferase
Control vs DSS 6-day	2.1.1.220	tRNA (adenine(58)-N(1))-methyltransferase
Control vs DSS 6-day	2.1.1.228	tRNA (guanine(37)-N(1))-methyltransferase
Control vs DSS 6-day	2.1.1.33	tRNA (guanine(46)-N(7))-methyltransferase
Control vs DSS 6-day	2.1.1.37	DNA (cytosine-5-)-methyltransferase
Control vs DSS 6-day	2.1.1.72	Site-specific DNA-methyltransferase (adenine-specific)
Control vs DSS 6-day	2.1.1.74	(FADH(2)-oxidizing)
Control vs DSS 6-day	2.1.1.77	Protein-L-isoaspartate(D-aspartate) O-methyltransferase
Control vs DSS 6-day	2.1.2.1	Glycine hydroxymethyltransferase
Control vs DSS 6-day	2.1.2.11	3-methyl-2-oxobutanoate hydroxymethyltransferase
Control vs DSS 6-day	2.1.2.2	Phosphoribosylglycinamide formyltransferase
Control vs DSS 6-day	2.1.2.3	Phosphoribosylaminoimidazolecarboxamide formyltransferase
Control vs DSS 6-day	2.1.2.9	Methionyl-tRNA formyltransferase
Control vs DSS 6-day	2.1.3.2	Aspartate carbamoyltransferase
Control vs DSS 6-day	2.1.3.3	Ornithine carbamoyltransferase
Control vs DSS 6-day	2.2.1.6	Acetolactate synthase
Control vs DSS 6-day	2.2.1.7	1-deoxy-D-xylulose-5-phosphate synthase
Control vs DSS 6-day	2.3.1.1	Amino-acid N-acetyltransferase
Control vs DSS 6-day	2.3.1.157	Glucosamine-1-phosphate N-acetyltransferase
Control vs DSS 6-day	2.3.1.179	Beta-ketoacyl-[acyl-carrier-protein] synthase II
Control vs DSS 6-day	2.3.1.234	N(6)-L-threonylcarbamoyladenine synthase
Control vs DSS 6-day	2.3.1.266	NO_NAME
Control vs DSS 6-day	2.3.1.30	Serine O-acetyltransferase
Control vs DSS 6-day	2.3.1.31	Homoserine O-acetyltransferase
Control vs DSS 6-day	2.3.1.35	Glutamate N-acetyltransferase
Control vs DSS 6-day	2.3.1.39	[Acyl-carrier-protein] S-malonyltransferase
Control vs DSS 6-day	2.3.1.51	1-acylglycerol-3-phosphate O-acyltransferase
Control vs DSS 6-day	2.3.1.n2	Phosphate acyltransferase
Control vs DSS 6-day	2.3.1.n3	Glycerol-3-phosphate acyltransferase (acyl-phosphate transferring)
Control vs DSS 6-day	2.3.3.13	2-isopropylmalate synthase
Control vs DSS 6-day	2.4.1.182	Lipid-A-disaccharide synthase
Control vs DSS 6-day	2.4.1.21	Starch synthase
Control vs DSS 6-day	2.4.1.227	acetylglucosaminyltransferase
Control vs DSS 6-day	2.4.2.10	Orotate phosphoribosyltransferase
Control vs DSS 6-day	2.4.2.14	Amidophosphoribosyltransferase
Control vs DSS 6-day	2.4.2.17	ATP phosphoribosyltransferase
Control vs DSS 6-day	2.4.2.21	Nicotinate-nucleotide--dimethylbenzimidazole phosphoribosyltransferase

Control vs DSS 6-day	2.4.2.29	tRNA-guanine(34) transglycosylase
Control vs DSS 6-day	2.4.2.8	Hypoxanthine phosphoribosyltransferase
Control vs DSS 6-day	2.4.2.9	Uracil phosphoribosyltransferase
Control vs DSS 6-day	2.4.99.17	S-adenosylmethionine
Control vs DSS 6-day	2.5.1.129	NO_NAME
Control vs DSS 6-day	2.5.1.19	3-phosphoshikimate 1-carboxyvinyltransferase
Control vs DSS 6-day	2.5.1.3	Thiamine-phosphate diphosphorylase
Control vs DSS 6-day	2.5.1.55	3-deoxy-8-phosphooctulonate synthase
Control vs DSS 6-day	2.5.1.6	Methionine adenosyltransferase
Control vs DSS 6-day	2.5.1.61	Hydroxymethylbilane synthase
Control vs DSS 6-day	2.5.1.7	UDP-N-acetylglucosamine 1-carboxyvinyltransferase
Control vs DSS 6-day	2.5.1.75	tRNA dimethylallyltransferase
Control vs DSS 6-day	2.5.1.78	6,7-dimethyl-8-ribityllumazine synthase
Control vs DSS 6-day	2.6.1.16	Glutamine--fructose-6-phosphate transaminase (isomerizing)
Control vs DSS 6-day	2.6.1.42	Branched-chain-amino-acid transaminase
Control vs DSS 6-day	2.6.1.62	Adenosylmethionine--8-amino-7-oxononanoate transaminase
Control vs DSS 6-day	2.6.1.9	Histidinol-phosphate transaminase
Control vs DSS 6-day	2.6.99.2	Pyridoxine 5'-phosphate synthase
Control vs DSS 6-day	2.7.1.11	6-phosphofructokinase
Control vs DSS 6-day	2.7.1.130	Tetraacyldisaccharide 4'-kinase
Control vs DSS 6-day	2.7.1.148	4-(cytidine 5'-diphospho)-2-C-methyl-D-erythritol kinase
Control vs DSS 6-day	2.7.1.167	D-glycero-beta-D-manno-heptose-7-phosphate kinase
Control vs DSS 6-day	2.7.1.180	FAD
Control vs DSS 6-day	2.7.1.23	NAD(+) kinase
Control vs DSS 6-day	2.7.1.24	Dephospho-CoA kinase
Control vs DSS 6-day	2.7.1.26	Riboflavin kinase
Control vs DSS 6-day	2.7.1.30	Glycerol kinase
Control vs DSS 6-day	2.7.1.33	Pantothenate kinase
Control vs DSS 6-day	2.7.1.40	Pyruvate kinase
Control vs DSS 6-day	2.7.1.71	Shikimate kinase
Control vs DSS 6-day	2.7.13.3	Histidine kinase
Control vs DSS 6-day	2.7.2.1	Acetate kinase
Control vs DSS 6-day	2.7.2.11	Glutamate 5-kinase
Control vs DSS 6-day	2.7.2.3	Phosphoglycerate kinase
Control vs DSS 6-day	2.7.2.4	Aspartate kinase
Control vs DSS 6-day	2.7.2.8	Acetylglutamate kinase
Control vs DSS 6-day	2.7.3.9	Phosphoenolpyruvate--protein phosphotransferase
Control vs DSS 6-day	2.7.4.1	Polyphosphate kinase
Control vs DSS 6-day	2.7.4.16	Thiamine-phosphate kinase
Control vs DSS 6-day	2.7.4.22	UMP kinase
Control vs DSS 6-day	2.7.4.25	(d)CMP kinase
Control vs DSS 6-day	2.7.4.3	Adenylate kinase
Control vs DSS 6-day	2.7.4.6	Nucleoside-diphosphate kinase

Control vs DSS 6-day	2.7.4.8	Guanylate kinase
Control vs DSS 6-day	2.7.4.9	dTMP kinase
Control vs DSS 6-day	2.7.6.1	Ribose-phosphate diphosphokinase
Control vs DSS 6-day	2.7.7.2	FAD synthetase
Control vs DSS 6-day	2.7.7.23	UDP-N-acetylglucosamine diphosphorylase
Control vs DSS 6-day	2.7.7.24	Glucose-1-phosphate thymidylyltransferase
Control vs DSS 6-day	2.7.7.3	Pantetheine-phosphate adenylyltransferase
Control vs DSS 6-day	2.7.7.38	3-deoxy-manno-octulosonate cytidylyltransferase
Control vs DSS 6-day	2.7.7.41	Phosphatidate cytidylyltransferase
Control vs DSS 6-day	2.7.7.6	DNA-directed RNA polymerase
Control vs DSS 6-day	2.7.7.60	2-C-methyl-D-erythritol 4-phosphate cytidylyltransferase
Control vs DSS 6-day	2.7.7.7	DNA-directed DNA polymerase
Control vs DSS 6-day	2.7.7.70	D-glycero-beta-D-manno-heptose 1-phosphate adenylyltransferase
Control vs DSS 6-day	2.7.7.77	Molybdenum cofactor guanylyltransferase
Control vs DSS 6-day	2.7.7.8	Polyribonucleotide nucleotidyltransferase
Control vs DSS 6-day	2.7.7.85	Diadenylate cyclase
Control vs DSS 6-day	2.7.7.9	UTP--glucose-1-phosphate uridylyltransferase
Control vs DSS 6-day	2.7.8.13	Phospho-N-acetylmuramoyl-pentapeptide-transferase
Control vs DSS 6-day	2.7.8.26	Adenosylcobinamide-GDP ribazoletransferase
Control vs DSS 6-day	2.7.8.7	Holo-[acyl-carrier-protein] synthase
Control vs DSS 6-day	2.8.1.10	Thiazole synthase
Control vs DSS 6-day	2.8.1.6	Biotin synthase
Control vs DSS 6-day	2.8.4.3	tRNA-2-methylthio-N(6)-dimethylallyladenosine synthase
Control vs DSS 6-day	2.8.4.4	[Ribosomal protein S12] (aspartate(89)-C(3))-methylthiotransferase
Control vs DSS 6-day	3.1.1.29	Aminoacyl-tRNA hydrolase
Control vs DSS 6-day	3.1.1.61	Protein-glutamate methylesterase
Control vs DSS 6-day	3.1.1.96	D-aminoacyl-tRNA deacylase
Control vs DSS 6-day	3.1.11.6	Exodeoxyribonuclease VII
Control vs DSS 6-day	3.1.13.1	Exoribonuclease II
Control vs DSS 6-day	3.1.21.2	Deoxyribonuclease IV
Control vs DSS 6-day	3.1.21.3	Type I site-specific deoxyribonuclease
Control vs DSS 6-day	3.1.22.4	Crossover junction endodeoxyribonuclease
Control vs DSS 6-day	3.1.26.3	Ribonuclease III
Control vs DSS 6-day	3.1.26.4	Ribonuclease H
Control vs DSS 6-day	3.1.26.5	Ribonuclease P
Control vs DSS 6-day	3.1.3.25	Inositol-phosphate phosphatase
Control vs DSS 6-day	3.1.3.5	5'-nucleotidase
Control vs DSS 6-day	3.1.4.58	NO_NAME
Control vs DSS 6-day	3.2.2.n1	Cytokinin riboside 5'-monophosphate phosphoribohydrolase
Control vs DSS 6-day	3.3.1.1	Adenosylhomocysteinase
Control vs DSS 6-day	3.4.11.18	Methionyl aminopeptidase
Control vs DSS 6-day	3.4.21.53	Endopeptidase La
Control vs DSS 6-day	3.4.21.88	Repressor LexA

Control vs DSS 6-day	3.4.21.89	Signal peptidase I
Control vs DSS 6-day	3.4.21.92	Endopeptidase Clp
Control vs DSS 6-day	3.4.23.36	Signal peptidase II
Control vs DSS 6-day	3.4.25.2	HslU--HslV peptidase
Control vs DSS 6-day	3.5.1.108	UDP-3-O-acyl-N-acetylglucosamine deacetylase
Control vs DSS 6-day	3.5.1.2	Glutaminase
Control vs DSS 6-day	3.5.1.44	Protein-glutamine glutaminase
Control vs DSS 6-day	3.5.1.88	Peptide deformylase
Control vs DSS 6-day	3.5.2.3	Dihydroorotase
Control vs DSS 6-day	3.5.4.10	IMP cyclohydrolase
Control vs DSS 6-day	3.5.4.16	GTP cyclohydrolase I
Control vs DSS 6-day	3.5.4.19	Phosphoribosyl-AMP cyclohydrolase
Control vs DSS 6-day	3.5.4.25	GTP cyclohydrolase II
Control vs DSS 6-day	3.5.4.26	Diaminohydroxyphosphoribosylaminopyrimidine deaminase
Control vs DSS 6-day	3.5.4.33	tRNA(adenine(34)) deaminase
Control vs DSS 6-day	3.5.4.9	Methenyltetrahydrofolate cyclohydrolase
Control vs DSS 6-day	3.6.1.23	dUTP diphosphatase
Control vs DSS 6-day	3.6.1.27	Undecaprenyl-diphosphate phosphatase
Control vs DSS 6-day	3.6.1.31	Phosphoribosyl-ATP diphosphatase
Control vs DSS 6-day	3.6.1.7	Acylphosphatase
Control vs DSS 6-day	3.6.3.31	Polyamine-transporting ATPase
Control vs DSS 6-day	3.6.4.12	DNA helicase
Control vs DSS 6-day	3.6.5.n1	Elongation factor 4
Control vs DSS 6-day	4.1.1.11	Aspartate 1-decarboxylase
Control vs DSS 6-day	4.1.1.23	Orotidine-5'-phosphate decarboxylase
Control vs DSS 6-day	4.1.1.36	Phosphopantothenoylcysteine decarboxylase
Control vs DSS 6-day	4.1.1.37	Uroporphyrinogen decarboxylase
Control vs DSS 6-day	4.1.1.49	Phosphoenolpyruvate carboxykinase (ATP)
Control vs DSS 6-day	4.1.1.65	Phosphatidylserine decarboxylase
Control vs DSS 6-day	4.1.99.12	3,4-dihydroxy-2-butanone-4-phosphate synthase
Control vs DSS 6-day	4.1.99.22	NO_NAME
Control vs DSS 6-day	4.2.1.10	3-dehydroquinate dehydratase
Control vs DSS 6-day	4.2.1.11	Phosphopyruvate hydratase
Control vs DSS 6-day	4.2.1.136	ADP-dependent NAD(P)H-hydrate dehydratase
Control vs DSS 6-day	4.2.1.151	Chorismate dehydratase
Control vs DSS 6-day	4.2.1.19	Imidazoleglycerol-phosphate dehydratase
Control vs DSS 6-day	4.2.1.20	Tryptophan synthase
Control vs DSS 6-day	4.2.1.24	Porphobilinogen synthase
Control vs DSS 6-day	4.2.1.33	3-isopropylmalate dehydratase
Control vs DSS 6-day	4.2.1.46	dTDP-glucose 4,6-dehydratase
Control vs DSS 6-day	4.2.1.59	3-hydroxyacyl-[acyl-carrier-protein] dehydratase
Control vs DSS 6-day	4.2.1.9	Dihydroxy-acid dehydratase
Control vs DSS 6-day	4.2.3.3	Methylglyoxal synthase

Control vs DSS 6-day	4.2.3.4	3-dehydroquinate synthase
Control vs DSS 6-day	4.2.3.5	Chorismate synthase
Control vs DSS 6-day	4.2.99.18	DNA-(apurinic or apyrimidinic site) lyase
Control vs DSS 6-day	4.3.1.1	Aspartate ammonia-lyase
Control vs DSS 6-day	4.3.2.1	Argininosuccinate lyase
Control vs DSS 6-day	4.3.2.10	NO_NAME
Control vs DSS 6-day	4.3.2.2	Adenylosuccinate lyase
Control vs DSS 6-day	4.3.3.7	4-hydroxy-tetrahydrodipicolinate synthase
Control vs DSS 6-day	4.6.1.12	2-C-methyl-D-erythritol 2,4-cyclodiphosphate synthase
Control vs DSS 6-day	4.6.1.17	NO_NAME
Control vs DSS 6-day	4.99.1.1	Ferrochelatase
Control vs DSS 6-day	5.1.1.1	Alanine racemase
Control vs DSS 6-day	5.1.1.3	Glutamate racemase
Control vs DSS 6-day	5.1.1.7	Diaminopimelate epimerase
Control vs DSS 6-day	5.1.3.1	Ribulose-phosphate 3-epimerase
Control vs DSS 6-day	5.1.3.13	dTDP-4-dehydrorhamnose 3,5-epimerase
Control vs DSS 6-day	5.1.3.20	ADP-glyceromanno-heptose 6-epimerase
Control vs DSS 6-day	5.1.99.6	NAD(P)H-hydrate epimerase
Control vs DSS 6-day	5.2.1.8	Peptidylprolyl isomerase
Control vs DSS 6-day	5.3.1.1	Triose-phosphate isomerase
Control vs DSS 6-day	5.3.1.16	isomerase
Control vs DSS 6-day	5.3.1.23	S-methyl-5-thioribose-1-phosphate isomerase
Control vs DSS 6-day	5.3.1.28	D-sedoheptulose 7-phosphate isomerase
Control vs DSS 6-day	5.3.1.9	Glucose-6-phosphate isomerase
Control vs DSS 6-day	5.4.2.10	Phosphoglucosamine mutase
Control vs DSS 6-day	5.4.2.12	Phosphoglycerate mutase (2,3-diphosphoglycerate-independent)
Control vs DSS 6-day	5.4.3.8	Glutamate-1-semialdehyde 2,1-aminomutase
Control vs DSS 6-day	5.4.99.12	tRNA pseudouridine(38-40) synthase
Control vs DSS 6-day	5.4.99.18	5-(carboxyamino)imidazole ribonucleotide mutase
Control vs DSS 6-day	5.4.99.25	tRNA pseudouridine(55) synthase
Control vs DSS 6-day	5.99.1.2	DNA topoisomerase
Control vs DSS 6-day	5.99.1.3	DNA topoisomerase (ATP-hydrolyzing)
Control vs DSS 6-day	6.1.1.1	Tyrosine--tRNA ligase
Control vs DSS 6-day	6.1.1.10	Methionine--tRNA ligase
Control vs DSS 6-day	6.1.1.11	Serine--tRNA ligase
Control vs DSS 6-day	6.1.1.14	Glycine--tRNA ligase
Control vs DSS 6-day	6.1.1.15	Proline--tRNA ligase
Control vs DSS 6-day	6.1.1.16	Cysteine--tRNA ligase
Control vs DSS 6-day	6.1.1.17	Glutamate--tRNA ligase
Control vs DSS 6-day	6.1.1.19	Arginine--tRNA ligase
Control vs DSS 6-day	6.1.1.20	Phenylalanine--tRNA ligase
Control vs DSS 6-day	6.1.1.21	Histidine--tRNA ligase
Control vs DSS 6-day	6.1.1.23	Aspartate--tRNA(Asn) ligase

Control vs DSS 6-day	6.1.1.3	Threonine--tRNA ligase
Control vs DSS 6-day	6.1.1.4	Leucine--tRNA ligase
Control vs DSS 6-day	6.1.1.5	Isoleucine--tRNA ligase
Control vs DSS 6-day	6.1.1.6	Lysine--tRNA ligase
Control vs DSS 6-day	6.1.1.7	Alanine--tRNA ligase
Control vs DSS 6-day	6.1.1.9	Valine--tRNA ligase
Control vs DSS 6-day	6.3.1.5	NAD(+) synthase
Control vs DSS 6-day	6.3.2.1	Pantoate--beta-alanine ligase (AMP-forming)
Control vs DSS 6-day	6.3.2.13	UDP-N-acetylmuramoyl-L-alanyl-D-glutamate--2,6-diaminopimelate ligase
Control vs DSS 6-day	6.3.2.4	D-alanine--D-alanine ligase
Control vs DSS 6-day	6.3.2.5	Phosphopantothenate--cysteine ligase
Control vs DSS 6-day	6.3.2.6	Phosphoribosylaminoimidazolesuccinocarboxamide synthase
Control vs DSS 6-day	6.3.2.8	UDP-N-acetylmuramate--L-alanine ligase
Control vs DSS 6-day	6.3.2.9	UDP-N-acetylmuramoyl-L-alanine--D-glutamate ligase
Control vs DSS 6-day	6.3.3.1	Phosphoribosylformylglycinamidine cyclo-ligase
Control vs DSS 6-day	6.3.3.2	5-formyltetrahydrofolate cyclo-ligase
Control vs DSS 6-day	6.3.3.3	Dethiobiotin synthase
Control vs DSS 6-day	6.3.4.13	Phosphoribosylamine--glycine ligase
Control vs DSS 6-day	6.3.4.15	Biotin--[acetyl-CoA-carboxylase] ligase
Control vs DSS 6-day	6.3.4.19	tRNA(Ile)-lysidine synthetase
Control vs DSS 6-day	6.3.4.2	CTP synthase (glutamine hydrolyzing)
Control vs DSS 6-day	6.3.4.20	7-cyano-7-deazaguanine synthase
Control vs DSS 6-day	6.3.4.21	Nicotinate phosphoribosyltransferase
Control vs DSS 6-day	6.3.4.4	Adenylosuccinate synthase
Control vs DSS 6-day	6.3.4.5	Argininosuccinate synthase
Control vs DSS 6-day	6.3.5.2	GMP synthase (glutamine-hydrolyzing)
Control vs DSS 6-day	6.3.5.5	Carbamoyl-phosphate synthase (glutamine-hydrolyzing)
Control vs DSS 6-day	6.3.5.7	Glutaminyl-tRNA synthase (glutamine-hydrolyzing)
Control vs DSS 6-day	6.5.1.2	DNA ligase (NAD(+))
Control vs DSS 6-day	7.1.2.2	NO_NAME
Control vs DSS 6-day	1.7.99.1	Hydroxylamine reductase
Control vs DSS 6-day	2.1.1.37	DNA (cytosine-5-)-methyltransferase
Control vs DSS 6-day	4.1.1.49	Phosphoenolpyruvate carboxykinase (ATP)
Control vs DSS 6-day	4.2.1.11	Phosphopyruvate hydratase
Control vs DSS 6-day	5.3.1.5	Xylose isomerase
Control vs DSS 6-day	2.7.13.3	Histidine kinase
Control vs DSS 6-day	3.1.21.3	Type I site-specific deoxyribonuclease
Control vs DSS 6-day	2.7.13.3	Histidine kinase
Control vs DSS 6-day	1.1.1.25	Shikimate dehydrogenase
Control vs DSS 6-day	1.1.1.37	Malate dehydrogenase
Control vs DSS 6-day	1.1.1.91	Aryl-alcohol dehydrogenase (NADP(+))
Control vs DSS 6-day	2.1.1.72	Site-specific DNA-methyltransferase (adenine-specific)
Control vs DSS 6-day	2.3.1.51	1-acylglycerol-3-phosphate O-acyltransferase

Control vs DSS 6-day	2.6.1.52	Phosphoserine transaminase
Control vs DSS 6-day	2.7.13.3	Histidine kinase
Control vs DSS 6-day	3.1.21.3	Type I site-specific deoxyribonuclease
Control vs DSS 6-day	3.1.3.23	Sugar-phosphatase
Control vs DSS 6-day	3.1.3.25	Inositol-phosphate phosphatase
Control vs DSS 6-day	3.4.21.92	Endopeptidase Clp
Control vs DSS 6-day	3.6.4.12	DNA helicase
Control vs DSS 6-day	4.1.99.12	3,4-dihydroxy-2-butanone-4-phosphate synthase
Control vs DSS 6-day	1.1.1.100	3-oxoacyl-[acyl-carrier-protein] reductase
Control vs DSS 6-day	1.1.1.205	IMP dehydrogenase
Control vs DSS 6-day	1.1.1.22	UDP-glucose 6-dehydrogenase
Control vs DSS 6-day	1.1.1.37	Malate dehydrogenase
Control vs DSS 6-day	1.1.1.86	Ketol-acid reductoisomerase (NADP(+))
Control vs DSS 6-day	1.1.1.95	Phosphoglycerate dehydrogenase
Control vs DSS 6-day	1.11.1.15	Peroxiredoxin
Control vs DSS 6-day	1.11.1.6	Catalase
Control vs DSS 6-day	1.15.1.1	Superoxide dismutase
Control vs DSS 6-day	1.16.3.1	Ferroxidase
Control vs DSS 6-day	1.16.3.2	Bacterial non-heme ferritin
Control vs DSS 6-day	1.17.7.4	NO_NAME
Control vs DSS 6-day	1.2.1.11	Aspartate-semialdehyde dehydrogenase
Control vs DSS 6-day	1.2.1.12	Glyceraldehyde-3-phosphate dehydrogenase (phosphorylating)
Control vs DSS 6-day	1.3.1.9	Enoyl-[acyl-carrier-protein] reductase (NADH)
Control vs DSS 6-day	1.3.5.1	Succinate dehydrogenase (quinone)
Control vs DSS 6-day	1.3.8.1	Short-chain acyl-CoA dehydrogenase
Control vs DSS 6-day	1.5.1.3	Dihydrofolate reductase
Control vs DSS 6-day	1.5.1.5	Methylenetetrahydrofolate dehydrogenase (NADP(+))
Control vs DSS 6-day	1.8.1.4	Dihydrolipoyl dehydrogenase
Control vs DSS 6-day	2.1.1.163	Demethylmenaquinone methyltransferase
Control vs DSS 6-day	2.1.1.45	Thymidylate synthase
Control vs DSS 6-day	2.1.2.1	Glycine hydroxymethyltransferase
Control vs DSS 6-day	2.1.2.10	Aminomethyltransferase
Control vs DSS 6-day	2.1.2.3	Phosphoribosylaminoimidazolecarboxamide formyltransferase
Control vs DSS 6-day	2.1.3.2	Aspartate carbamoyltransferase
Control vs DSS 6-day	2.3.1.129	Acyl-[acyl-carrier-protein]--UDP-N-acetylglucosamine O-acyltransferase
Control vs DSS 6-day	2.3.1.179	Beta-ketoacyl-[acyl-carrier-protein] synthase II
Control vs DSS 6-day	2.3.1.180	Beta-ketoacyl-[acyl-carrier-protein] synthase III
Control vs DSS 6-day	2.3.1.29	Glycine C-acetyltransferase
Control vs DSS 6-day	2.4.2.10	Orotate phosphoribosyltransferase
Control vs DSS 6-day	2.4.2.19	Nicotinate-nucleotide diphosphorylase (carboxylating)
Control vs DSS 6-day	2.4.2.8	Hypoxanthine phosphoribosyltransferase
Control vs DSS 6-day	2.4.99.17	S-adenosylmethionine
Control vs DSS 6-day	2.5.1.6	Methionine adenosyltransferase

Control vs DSS 6-day	2.5.1.78	6,7-dimethyl-8-ribityllumazine synthase
Control vs DSS 6-day	2.5.1.9	Riboflavin synthase
Control vs DSS 6-day	2.6.1.52	Phosphoserine transaminase
Control vs DSS 6-day	2.7.1.11	6-phosphofructokinase
Control vs DSS 6-day	2.7.1.2	Glucokinase
Control vs DSS 6-day	2.7.1.33	Pantothenate kinase
Control vs DSS 6-day	2.7.1.40	Pyruvate kinase
Control vs DSS 6-day	2.7.1.90	Diphosphate--fructose-6-phosphate 1-phosphotransferase
Control vs DSS 6-day	2.7.13.3	Histidine kinase
Control vs DSS 6-day	2.7.2.3	Phosphoglycerate kinase
Control vs DSS 6-day	2.7.2.4	Aspartate kinase
Control vs DSS 6-day	2.7.4.16	Thiamine-phosphate kinase
Control vs DSS 6-day	2.7.4.22	UMP kinase
Control vs DSS 6-day	2.7.4.3	Adenylate kinase
Control vs DSS 6-day	2.7.4.8	Guanylate kinase
Control vs DSS 6-day	2.7.7.4	Sulfate adenylyltransferase
Control vs DSS 6-day	2.7.7.49	RNA-directed DNA polymerase
Control vs DSS 6-day	2.7.7.6	DNA-directed RNA polymerase
Control vs DSS 6-day	2.7.7.8	Polyribonucleotide nucleotidyltransferase
Control vs DSS 6-day	2.7.9.1	Pyruvate, phosphate dikinase
Control vs DSS 6-day	3.1.21.3	Type I site-specific deoxyribonuclease
Control vs DSS 6-day	3.1.26.5	Ribonuclease P
Control vs DSS 6-day	3.1.3.25	Inositol-phosphate phosphatase
Control vs DSS 6-day	3.1.3.7	3'(2'),5'-bisphosphate nucleotidase
Control vs DSS 6-day	3.2.1.23	Beta-galactosidase
Control vs DSS 6-day	3.3.1.1	Adenosylhomocysteinase
Control vs DSS 6-day	3.4.11.18	Methionyl aminopeptidase
Control vs DSS 6-day	3.4.21.92	Endopeptidase Clp
Control vs DSS 6-day	3.5.1.108	UDP-3-O-acyl-N-acetylglucosamine deacetylase
Control vs DSS 6-day	3.5.1.25	N-acetylglucosamine-6-phosphate deacetylase
Control vs DSS 6-day	3.5.4.10	IMP cyclohydrolase
Control vs DSS 6-day	3.5.4.9	Methenyltetrahydrofolate cyclohydrolase
Control vs DSS 6-day	3.5.99.6	Glucosamine-6-phosphate deaminase
Control vs DSS 6-day	4.1.1.23	Orotidine-5'-phosphate decarboxylase
Control vs DSS 6-day	4.1.1.49	Phosphoenolpyruvate carboxykinase (ATP)
Control vs DSS 6-day	4.2.1.10	3-dehydroquinate dehydratase
Control vs DSS 6-day	4.2.1.11	Phosphopyruvate hydratase
Control vs DSS 6-day	4.2.1.2	Fumarate hydratase
Control vs DSS 6-day	4.2.1.59	3-hydroxyacyl-[acyl-carrier-protein] dehydratase
Control vs DSS 6-day	4.2.1.8	Mannonate dehydratase
Control vs DSS 6-day	4.3.2.2	Adenylosuccinate lyase
Control vs DSS 6-day	4.3.3.7	4-hydroxy-tetrahydrodipicolinate synthase
Control vs DSS 6-day	5.1.3.2	UDP-glucose 4-epimerase

Control vs DSS 6-day	5.1.3.3	Aldose 1-epimerase
Control vs DSS 6-day	5.1.99.1	Methylmalonyl-CoA epimerase
Control vs DSS 6-day	5.2.1.8	Peptidylprolyl isomerase
Control vs DSS 6-day	5.3.1.1	Triose-phosphate isomerase
Control vs DSS 6-day	5.3.1.5	Xylose isomerase
Control vs DSS 6-day	5.3.1.9	Glucose-6-phosphate isomerase
Control vs DSS 6-day	5.4.2.11	Phosphoglycerate mutase (2,3-diphosphoglycerate-dependent)
Control vs DSS 6-day	5.4.2.12	Phosphoglycerate mutase (2,3-diphosphoglycerate-independent)
Control vs DSS 6-day	5.4.99.18	5-(carboxyamino)imidazole ribonucleotide mutase
Control vs DSS 6-day	5.99.1.2	DNA topoisomerase
Control vs DSS 6-day	5.99.1.3	DNA topoisomerase (ATP-hydrolyzing)
Control vs DSS 6-day	6.1.1.1	Tyrosine--tRNA ligase
Control vs DSS 6-day	6.1.1.14	Glycine--tRNA ligase
Control vs DSS 6-day	6.1.1.15	Proline--tRNA ligase
Control vs DSS 6-day	6.1.1.19	Arginine--tRNA ligase
Control vs DSS 6-day	6.1.1.2	Tryptophan--tRNA ligase
Control vs DSS 6-day	6.1.1.20	Phenylalanine--tRNA ligase
Control vs DSS 6-day	6.1.1.22	Asparagine--tRNA ligase
Control vs DSS 6-day	6.1.1.3	Threonine--tRNA ligase
Control vs DSS 6-day	6.1.1.4	Leucine--tRNA ligase
Control vs DSS 6-day	6.1.1.6	Lysine--tRNA ligase
Control vs DSS 6-day	6.3.2.4	D-alanine--D-alanine ligase
Control vs DSS 6-day	6.3.2.6	Phosphoribosylaminoimidazolesuccinocarboxamide synthase
Control vs DSS 6-day	6.3.4.4	Adenylosuccinate synthase
Control vs DSS 6-day	6.3.5.2	GMP synthase (glutamine-hydrolyzing)
Control vs DSS 6-day	7.2.1.1	NO_NAME
Control vs DSS 6-day	1.1.1.37	Malate dehydrogenase
Control vs DSS 6-day	1.2.1.12	Glyceraldehyde-3-phosphate dehydrogenase (phosphorylating)
Control vs DSS 6-day	2.1.1.72	Site-specific DNA-methyltransferase (adenine-specific)
Control vs DSS 6-day	2.7.7.6	DNA-directed RNA polymerase
Control vs DSS 6-day	3.1.21.3	Type I site-specific deoxyribonuclease
Control vs DSS 6-day	4.1.1.49	Phosphoenolpyruvate carboxykinase (ATP)
Control vs DSS 6-day	5.4.2.12	Phosphoglycerate mutase (2,3-diphosphoglycerate-independent)
Control vs DSS 6-day	6.1.1.3	Threonine--tRNA ligase
Control vs DSS 6-day	1.1.1.37	Malate dehydrogenase
Control vs DSS 6-day	1.3.8.1	Short-chain acyl-CoA dehydrogenase
Control vs DSS 6-day	2.7.13.3	Histidine kinase
Control vs DSS 6-day	2.7.7.6	DNA-directed RNA polymerase
Control vs DSS 6-day	4.1.1.49	Phosphoenolpyruvate carboxykinase (ATP)
Control vs DSS 6-day	2.7.7.6	DNA-directed RNA polymerase
Control vs DSS 6-day	2.7.7.6	DNA-directed RNA polymerase
Control vs DSS 6-day	3.6.4.12	DNA helicase
Control vs DSS 6-day	2.4.1.1	Glycogen phosphorylase

Control vs DSS 6-day	2.7.6.1	Ribose-phosphate diphosphokinase
Control vs DSS 6-day	2.7.7.6	DNA-directed RNA polymerase
Control vs DSS 6-day	3.1.21.3	Type I site-specific deoxyribonuclease
Control vs DSS 6-day	2.7.13.3	Histidine kinase
Control vs DSS 6-day	2.7.13.3	Histidine kinase
Control vs DSS 6-day	2.7.7.6	DNA-directed RNA polymerase
Control vs DSS 6-day	2.7.13.3	Histidine kinase
Control vs DSS 6-day	2.7.7.6	DNA-directed RNA polymerase
Control vs DSS 6-day	2.1.1.37	DNA (cytosine-5-)-methyltransferase
Control vs DSS 6-day	2.7.7.6	DNA-directed RNA polymerase
Control vs DSS 6-day	2.1.1.37	DNA (cytosine-5-)-methyltransferase
Control vs DSS 6-day	2.7.13.3	Histidine kinase
Control vs DSS 6-day	2.7.13.3	Histidine kinase
Control vs DSS 6-day	2.7.7.6	DNA-directed RNA polymerase
Control vs DSS 6-day	3.1.21.3	Type I site-specific deoxyribonuclease
Control vs DSS 6-day	2.7.13.3	Histidine kinase
Control vs DSS 6-day	2.7.7.6	DNA-directed RNA polymerase
Control vs DSS 6-day	2.7.7.6	DNA-directed RNA polymerase
Control vs DSS 6-day	2.7.7.6	DNA-directed RNA polymerase
Control vs DSS 6-day	2.1.1.37	DNA (cytosine-5-)-methyltransferase
Control vs DSS 6-day	2.7.9.1	Pyruvate, phosphate dikinase
Control vs DSS 6-day	2.7.13.3	Histidine kinase
Control vs DSS 6-day	2.7.9.1	Pyruvate, phosphate dikinase
Control vs DSS 6-day	4.1.1.49	Phosphoenolpyruvate carboxykinase (ATP)
Control vs DSS 6-day	2.7.9.1	Pyruvate, phosphate dikinase
Control vs DSS 6-day	2.7.9.1	Pyruvate, phosphate dikinase
Control vs DSS 6-day	3.6.3.20	Glycerol-3-phosphate-transporting ATPase
Control vs DSS 6-day	4.1.1.49	Phosphoenolpyruvate carboxykinase (ATP)
Control vs DSS 6-day	1.8.99.5	Dissimilatory sulfite reductase

Genus	Species	p-value	FDR
g__Bacillus	s__Bacillus_subtilis_group	0.0108	0.0108
g__Escherichia	s__Escherichia_coli	0.0151	0.0151
g__Bacteroides	s__Bacteroides_caccae	0.0179	0.0179
g__Bacteroides	s__Bacteroides_caccae	0.0179	0.0179
g__Bacteroides	s__Bacteroides_caecimuris	0.0179	0.0179
g__Bacteroides	s__Bacteroides_caecimuris	0.0179	0.0179
g__Bacteroides	s__Bacteroides_caecimuris	0.0179	0.0179
g__Bacteroides	s__Bacteroides_faecis	0.0179	0.0179
g__Bacteroides	s__Bacteroides_faecis	0.0179	0.0179
g__Bacteroides	s__Bacteroides_finegoldii	0.0179	0.0179
g__Bacteroides	s__Bacteroides_finegoldii	0.0179	0.0179
g__Bacteroides	s__Bacteroides_fragilis	0.0179	0.0179
g__Bacteroides	s__Bacteroides_ovatus	0.0179	0.0179
g__Bacteroides	s__Bacteroides_ovatus	0.0179	0.0179
g__Bacteroides	s__Bacteroides_thetaiotaomicron	0.0179	0.0179
g__Bacteroides	s__Bacteroides_thetaiotaomicron	0.0179	0.0179
g__Bacteroides	s__Bacteroides_xylanisolvens	0.0179	0.0179
g__Bacteroides	s__Bacteroides_xylanisolvens	0.0179	0.0179
g__Catenibacterium	s__Catenibacterium_mitsuokai	0.0179	0.0179
g__Lactobacillus	s__Lactobacillus_johnsonii	0.0179	0.0179
g__Parabacteroides	s__Parabacteroides_goldsteinii	0.0179	0.0179
g__Bacillus	s__Bacillus_paralicheniformis	0.0416	0.0416
g__Klebsiella	s__Klebsiella_pneumoniae	0.0416	0.0416
g__Bacteroides	s__Bacteroides_nordii	0.0108	0.0108
g__Bacteroides	s__Bacteroides_caccae	0.0129	0.0129
g__Bacteroides	s__Bacteroides_xylanisolvens	0.0129	0.0129
g__Bacteroides	s__Bacteroides_caccae	0.0133	0.0133
g__Bacteroides	s__Bacteroides_caccae	0.0133	0.0133
g__Bacteroides	s__Bacteroides_caccae	0.0133	0.0133
g__Bacteroides	s__Bacteroides_finegoldii	0.0133	0.0133
g__Bacteroides	s__Bacteroides_fragilis	0.0133	0.0133
g__Bacteroides	s__Bacteroides_fragilis	0.0133	0.0133
g__Bacteroides	s__Bacteroides_fragilis	0.0133	0.0133
g__Bacteroides	s__Bacteroides_fragilis	0.0133	0.0133
g__Bacteroides	s__Bacteroides_fragilis	0.0133	0.0133
g__Bacteroides	s__Bacteroides_fragilis	0.0133	0.0133
g__Bacteroides	s__Bacteroides_ovatus	0.0133	0.0133
g__Bacteroides	s__Bacteroides_ovatus	0.0133	0.0133
g__Bacteroides	s__Bacteroides_salyersiae	0.0133	0.0133
g__Bacteroides	s__Bacteroides_uniformis	0.0133	0.0133
g__Bacteroides	s__Bacteroides_xylanisolvens	0.0133	0.0133

g__Bacteroides	s__Bacteroides_xylanisolvens	0.0133	0.0133
g__Bacteroides	s__Bacteroides_xylanisolvens	0.0133	0.0133
g__Catenibacterium	s__Catenibacterium_mitsuokai	0.0133	0.0133
g__Catenibacterium	s__Catenibacterium_mitsuokai	0.0133	0.0133
g__Bacteroides	s__Bacteroides_uniformis	0.0142	0.0142
g__Catenibacterium	s__Catenibacterium_mitsuokai	0.0142	0.0142
g__Bacteroides	s__Bacteroides_caccae	0.0146	0.0146
g__Bacteroides	s__Bacteroides_caccae	0.0146	0.0146
g__Bacteroides	s__Bacteroides_caccae	0.0146	0.0146
g__Bacteroides	s__Bacteroides_caccae	0.0146	0.0146
g__Bacteroides	s__Bacteroides_caccae	0.0146	0.0146
g__Bacteroides	s__Bacteroides_caccae	0.0146	0.0146
g__Bacteroides	s__Bacteroides_caecimuris	0.0146	0.0146
g__Bacteroides	s__Bacteroides_caecimuris	0.0146	0.0146
g__Bacteroides	s__Bacteroides_cellulosilyticus	0.0146	0.0146
g__Bacteroides	s__Bacteroides_cellulosilyticus	0.0146	0.0146
g__Bacteroides	s__Bacteroides_cellulosilyticus	0.0146	0.0146
g__Bacteroides	s__Bacteroides_faecis	0.0146	0.0146
g__Bacteroides	s__Bacteroides_faecis	0.0146	0.0146
g__Bacteroides	s__Bacteroides_faecis	0.0146	0.0146
g__Bacteroides	s__Bacteroides_faecis	0.0146	0.0146
g__Bacteroides	s__Bacteroides_faecis	0.0146	0.0146
g__Bacteroides	s__Bacteroides_finegoldii	0.0146	0.0146
g__Bacteroides	s__Bacteroides_finegoldii	0.0146	0.0146
g__Bacteroides	s__Bacteroides_finegoldii	0.0146	0.0146
g__Bacteroides	s__Bacteroides_finegoldii	0.0146	0.0146
g__Bacteroides	s__Bacteroides_finegoldii	0.0146	0.0146
g__Bacteroides	s__Bacteroides_finegoldii	0.0146	0.0146
g__Bacteroides	s__Bacteroides_fragilis	0.0146	0.0146
g__Bacteroides	s__Bacteroides_fragilis	0.0146	0.0146
g__Bacteroides	s__Bacteroides_fragilis	0.0146	0.0146
g__Bacteroides	s__Bacteroides_fragilis	0.0146	0.0146
g__Bacteroides	s__Bacteroides_fragilis	0.0146	0.0146
g__Bacteroides	s__Bacteroides_fragilis	0.0146	0.0146
g__Bacteroides	s__Bacteroides_fragilis	0.0146	0.0146
g__Bacteroides	s__Bacteroides_intestinalis	0.0146	0.0146
g__Bacteroides	s__Bacteroides_nordii	0.0146	0.0146
g__Bacteroides	s__Bacteroides_ovatus	0.0146	0.0146
g__Bacteroides	s__Bacteroides_ovatus	0.0146	0.0146
g__Bacteroides	s__Bacteroides_ovatus	0.0146	0.0146
g__Bacteroides	s__Bacteroides_ovatus	0.0146	0.0146
g__Bacteroides	s__Bacteroides_ovatus	0.0146	0.0146
g__Bacteroides	s__Bacteroides_ovatus	0.0146	0.0146

g__Bacteroides	s__Bacteroides_ovatus	0.0146	0.0146
g__Bacteroides	s__Bacteroides_salyersiae	0.0146	0.0146
g__Bacteroides	s__Bacteroides_stercoris	0.0146	0.0146
g__Bacteroides	s__Bacteroides_thetaiotaomicron	0.0146	0.0146
g__Bacteroides	s__Bacteroides_thetaiotaomicron	0.0146	0.0146
g__Bacteroides	s__Bacteroides_uniformis	0.0146	0.0146
g__Bacteroides	s__Bacteroides_uniformis	0.0146	0.0146
g__Bacteroides	s__Bacteroides_uniformis	0.0146	0.0146
g__Bacteroides	s__Bacteroides_xylanisolvens	0.0146	0.0146
g__Bacteroides	s__Bacteroides_xylanisolvens	0.0146	0.0146
g__Bacteroides	s__Bacteroides_xylanisolvens	0.0146	0.0146
g__Bacteroides	s__Bacteroides_xylanisolvens	0.0146	0.0146
g__Bacteroides	s__Bacteroides_xylanisolvens	0.0146	0.0146
g__Catenibacterium	s__Catenibacterium_mitsuokai	0.0146	0.0146
g__Catenibacterium	s__Catenibacterium_mitsuokai	0.0146	0.0146
g__Catenibacterium	s__Catenibacterium_mitsuokai	0.0146	0.0146
g__Klebsiella	s__Klebsiella_oxytoca	0.0146	0.0146
g__Lactobacillus	s__Lactobacillus_paragasseri	0.0146	0.0146
g__Lactobacillus	s__Lactobacillus_taiwanensis	0.0146	0.0146
g__Lactobacillus	s__Lactobacillus_taiwanensis	0.0146	0.0146
g__Acutalibacter	s__Acutalibacter_muris	0.0151	0.0151
g__Adlercreutzia	s__Adlercreutzia_equolifaciens	0.0151	0.0151
g__Adlercreutzia	s__Adlercreutzia_equolifaciens	0.0151	0.0151
g__Adlercreutzia	s__Adlercreutzia_equolifaciens	0.0151	0.0151
g__Akkermansia	s__Akkermansia_muciniphila	0.0151	0.0151
g__Alistipes	s__Alistipes_finegoldii	0.0151	0.0151
g__Alistipes	s__Alistipes_finegoldii	0.0151	0.0151
g__Alistipes	s__Alistipes_finegoldii	0.0151	0.0151
g__Alistipes	s__Alistipes_finegoldii	0.0151	0.0151
g__Alistipes	s__Alistipes_finegoldii	0.0151	0.0151
g__Alistipes	s__Alistipes_finegoldii	0.0151	0.0151
g__Alistipes	s__Alistipes_finegoldii	0.0151	0.0151
g__Alistipes	s__Alistipes_finegoldii	0.0151	0.0151
g__Alistipes	s__Alistipes_finegoldii	0.0151	0.0151
g__Alistipes	s__Alistipes_finegoldii	0.0151	0.0151
g__Alistipes	s__Alistipes_finegoldii	0.0151	0.0151
g__Alistipes	s__Alistipes_finegoldii	0.0151	0.0151
g__Alistipes	s__Alistipes_onderdonkii	0.0151	0.0151
g__Alistipes	s__Alistipes_onderdonkii	0.0151	0.0151
g__Alistipes	s__Alistipes_onderdonkii	0.0151	0.0151
g__Alistipes	s__Alistipes_onderdonkii	0.0151	0.0151
g__Alistipes	s__Alistipes_onderdonkii	0.0151	0.0151
g__Alistipes	s__Alistipes_onderdonkii	0.0151	0.0151

g__Alistipes	s__Alistipes_onderdonkii	0.0151	0.0151
g__Alistipes	s__Alistipes_onderdonkii	0.0151	0.0151
g__Alistipes	s__Alistipes_onderdonkii	0.0151	0.0151
g__Alistipes	s__Alistipes_onderdonkii	0.0151	0.0151
g__Alistipes	s__Alistipes_onderdonkii	0.0151	0.0151
g__Alistipes	s__Alistipes_onderdonkii	0.0151	0.0151
g__Alistipes	s__Alistipes_shahii	0.0151	0.0151
g__Alistipes	s__Alistipes_shahii	0.0151	0.0151
g__Alistipes	s__Alistipes_shahii	0.0151	0.0151
g__Alistipes	s__Alistipes_shahii	0.0151	0.0151
g__Alistipes	s__Alistipes_shahii	0.0151	0.0151
g__Alistipes	s__Alistipes_shahii	0.0151	0.0151
g__Alistipes	s__Alistipes_shahii	0.0151	0.0151
g__Alistipes	s__Alistipes_shahii	0.0151	0.0151
g__Alistipes	s__Alistipes_shahii	0.0151	0.0151
g__Alistipes	s__Alistipes_shahii	0.0151	0.0151
g__Alistipes	s__Alistipes_shahii	0.0151	0.0151
g__Alistipes	s__Alistipes_shahii	0.0151	0.0151
g__Alistipes	s__Alistipes_shahii	0.0151	0.0151
g__Bacteroides	s__Bacteroides_caccae	0.0151	0.0151
g__Bacteroides	s__Bacteroides_caccae	0.0151	0.0151
g__Bacteroides	s__Bacteroides_caccae	0.0151	0.0151
g__Bacteroides	s__Bacteroides_caccae	0.0151	0.0151
g__Bacteroides	s__Bacteroides_caccae	0.0151	0.0151
g__Bacteroides	s__Bacteroides_caccae	0.0151	0.0151
g__Bacteroides	s__Bacteroides_caccae	0.0151	0.0151
g__Bacteroides	s__Bacteroides_caccae	0.0151	0.0151
g__Bacteroides	s__Bacteroides_caccae	0.0151	0.0151
g__Bacteroides	s__Bacteroides_caccae	0.0151	0.0151
g__Bacteroides	s__Bacteroides_caccae	0.0151	0.0151
g__Bacteroides	s__Bacteroides_caccae	0.0151	0.0151
g__Bacteroides	s__Bacteroides_caccae	0.0151	0.0151
g__Bacteroides	s__Bacteroides_caccae	0.0151	0.0151
g__Bacteroides	s__Bacteroides_caccae	0.0151	0.0151
g__Bacteroides	s__Bacteroides_caccae	0.0151	0.0151
g__Bacteroides	s__Bacteroides_caccae	0.0151	0.0151
g__Bacteroides	s__Bacteroides_caccae	0.0151	0.0151
g__Bacteroides	s__Bacteroides_caccae	0.0151	0.0151
g__Bacteroides	s__Bacteroides_caccae	0.0151	0.0151
g__Bacteroides	s__Bacteroides_caccae	0.0151	0.0151
g__Bacteroides	s__Bacteroides_caccae	0.0151	0.0151
g__Bacteroides	s__Bacteroides_caccae	0.0151	0.0151
g__Bacteroides	s__Bacteroides_caccae	0.0151	0.0151

g__Bacteroides	s__Bacteroides_caccae	0.0151	0.0151
g__Bacteroides	s__Bacteroides_caccae	0.0151	0.0151
g__Bacteroides	s__Bacteroides_caccae	0.0151	0.0151
g__Bacteroides	s__Bacteroides_caccae	0.0151	0.0151
g__Bacteroides	s__Bacteroides_caccae	0.0151	0.0151
g__Bacteroides	s__Bacteroides_caccae	0.0151	0.0151
g__Bacteroides	s__Bacteroides_caccae	0.0151	0.0151
g__Bacteroides	s__Bacteroides_caccae	0.0151	0.0151
g__Bacteroides	s__Bacteroides_caccae	0.0151	0.0151
g__Bacteroides	s__Bacteroides_caccae	0.0151	0.0151
g__Bacteroides	s__Bacteroides_caccae	0.0151	0.0151
g__Bacteroides	s__Bacteroides_caccae	0.0151	0.0151
g__Bacteroides	s__Bacteroides_caccae	0.0151	0.0151
g__Bacteroides	s__Bacteroides_caccae	0.0151	0.0151
g__Bacteroides	s__Bacteroides_caccae	0.0151	0.0151
g__Bacteroides	s__Bacteroides_caccae	0.0151	0.0151
g__Bacteroides	s__Bacteroides_caccae	0.0151	0.0151
g__Bacteroides	s__Bacteroides_caccae	0.0151	0.0151
g__Bacteroides	s__Bacteroides_caccae	0.0151	0.0151
g__Bacteroides	s__Bacteroides_caccae	0.0151	0.0151
g__Bacteroides	s__Bacteroides_caccae	0.0151	0.0151
g__Bacteroides	s__Bacteroides_caccae	0.0151	0.0151
g__Bacteroides	s__Bacteroides_caccae	0.0151	0.0151
g__Bacteroides	s__Bacteroides_caccae	0.0151	0.0151
g__Bacteroides	s__Bacteroides_caccae	0.0151	0.0151
g__Bacteroides	s__Bacteroides_caccae	0.0151	0.0151
g__Bacteroides	s__Bacteroides_caccae	0.0151	0.0151
g__Bacteroides	s__Bacteroides_caccae	0.0151	0.0151
g__Bacteroides	s__Bacteroides_caccae	0.0151	0.0151
g__Bacteroides	s__Bacteroides_caccae	0.0151	0.0151
g__Bacteroides	s__Bacteroides_caccae	0.0151	0.0151
g__Bacteroides	s__Bacteroides_caccae	0.0151	0.0151
g__Bacteroides	s__Bacteroides_caccae	0.0151	0.0151
g__Bacteroides	s__Bacteroides_caccae	0.0151	0.0151
g__Bacteroides	s__Bacteroides_caccae	0.0151	0.0151
g__Bacteroides	s__Bacteroides_caccae	0.0151	0.0151
g__Bacteroides	s__Bacteroides_caccae	0.0151	0.0151
g__Bacteroides	s__Bacteroides_caccae	0.0151	0.0151
g__Bacteroides	s__Bacteroides_caccae	0.0151	0.0151
g__Bacteroides	s__Bacteroides_caccae	0.0151	0.0151
g__Bacteroides	s__Bacteroides_caccae	0.0151	0.0151
g__Bacteroides	s__Bacteroides_caccae	0.0151	0.0151
g__Bacteroides	s__Bacteroides_caccae	0.0151	0.0151

g__Bacteroides	s__Bacteroides_caccae	0.0151	0.0151
g__Bacteroides	s__Bacteroides_caccae	0.0151	0.0151
g__Bacteroides	s__Bacteroides_caccae	0.0151	0.0151
g__Bacteroides	s__Bacteroides_caccae	0.0151	0.0151
g__Bacteroides	s__Bacteroides_caccae	0.0151	0.0151
g__Bacteroides	s__Bacteroides_caccae	0.0151	0.0151
g__Bacteroides	s__Bacteroides_caccae	0.0151	0.0151
g__Bacteroides	s__Bacteroides_caccae	0.0151	0.0151
g__Bacteroides	s__Bacteroides_caccae	0.0151	0.0151
g__Bacteroides	s__Bacteroides_caccae	0.0151	0.0151
g__Bacteroides	s__Bacteroides_caccae	0.0151	0.0151
g__Bacteroides	s__Bacteroides_caccae	0.0151	0.0151
g__Bacteroides	s__Bacteroides_caccae	0.0151	0.0151
g__Bacteroides	s__Bacteroides_caccae	0.0151	0.0151
g__Bacteroides	s__Bacteroides_caccae	0.0151	0.0151
g__Bacteroides	s__Bacteroides_caccae	0.0151	0.0151
g__Bacteroides	s__Bacteroides_caccae	0.0151	0.0151
g__Bacteroides	s__Bacteroides_caccae	0.0151	0.0151
g__Bacteroides	s__Bacteroides_caccae	0.0151	0.0151
g__Bacteroides	s__Bacteroides_caccae	0.0151	0.0151
g__Bacteroides	s__Bacteroides_caccae	0.0151	0.0151
g__Bacteroides	s__Bacteroides_caccae	0.0151	0.0151
g__Bacteroides	s__Bacteroides_caccae	0.0151	0.0151
g__Bacteroides	s__Bacteroides_caccae	0.0151	0.0151
g__Bacteroides	s__Bacteroides_caccae	0.0151	0.0151
g__Bacteroides	s__Bacteroides_caccae	0.0151	0.0151
g__Bacteroides	s__Bacteroides_caccae	0.0151	0.0151
g__Bacteroides	s__Bacteroides_caccae	0.0151	0.0151
g__Bacteroides	s__Bacteroides_caccae	0.0151	0.0151
g__Bacteroides	s__Bacteroides_caccae	0.0151	0.0151
g__Bacteroides	s__Bacteroides_caccae	0.0151	0.0151
g__Bacteroides	s__Bacteroides_caccae	0.0151	0.0151
g__Bacteroides	s__Bacteroides_caccae	0.0151	0.0151
g__Bacteroides	s__Bacteroides_caccae	0.0151	0.0151
g__Bacteroides	s__Bacteroides_caccae	0.0151	0.0151
g__Bacteroides	s__Bacteroides_caccae	0.0151	0.0151
g__Bacteroides	s__Bacteroides_caccae	0.0151	0.0151
g__Bacteroides	s__Bacteroides_caccae	0.0151	0.0151
g__Bacteroides	s__Bacteroides_caccae	0.0151	0.0151
g__Bacteroides	s__Bacteroides_caccae	0.0151	0.0151
g__Bacteroides	s__Bacteroides_caccae	0.0151	0.0151
g__Bacteroides	s__Bacteroides_caccae	0.0151	0.0151
g__Bacteroides	s__Bacteroides_caccae	0.0151	0.0151

g__Bacteroides	s__Bacteroides_caccae	0.0151	0.0151
g__Bacteroides	s__Bacteroides_caccae	0.0151	0.0151
g__Bacteroides	s__Bacteroides_caccae	0.0151	0.0151
g__Bacteroides	s__Bacteroides_caccae	0.0151	0.0151
g__Bacteroides	s__Bacteroides_caccae	0.0151	0.0151
g__Bacteroides	s__Bacteroides_caccae	0.0151	0.0151
g__Bacteroides	s__Bacteroides_caccae	0.0151	0.0151
g__Bacteroides	s__Bacteroides_caccae	0.0151	0.0151
g__Bacteroides	s__Bacteroides_caccae	0.0151	0.0151
g__Bacteroides	s__Bacteroides_caccae	0.0151	0.0151
g__Bacteroides	s__Bacteroides_caccae	0.0151	0.0151
g__Bacteroides	s__Bacteroides_caccae	0.0151	0.0151
g__Bacteroides	s__Bacteroides_caccae	0.0151	0.0151
g__Bacteroides	s__Bacteroides_caccae	0.0151	0.0151
g__Bacteroides	s__Bacteroides_caccae	0.0151	0.0151
g__Bacteroides	s__Bacteroides_caccae	0.0151	0.0151
g__Bacteroides	s__Bacteroides_caccae	0.0151	0.0151
g__Bacteroides	s__Bacteroides_caccae	0.0151	0.0151
g__Bacteroides	s__Bacteroides_caccae	0.0151	0.0151
g__Bacteroides	s__Bacteroides_caccae	0.0151	0.0151
g__Bacteroides	s__Bacteroides_caccae	0.0151	0.0151
g__Bacteroides	s__Bacteroides_caccae	0.0151	0.0151
g__Bacteroides	s__Bacteroides_caccae	0.0151	0.0151
g__Bacteroides	s__Bacteroides_caccae	0.0151	0.0151
g__Bacteroides	s__Bacteroides_caecimuris	0.0151	0.0151
g__Bacteroides	s__Bacteroides_caecimuris	0.0151	0.0151
g__Bacteroides	s__Bacteroides_caecimuris	0.0151	0.0151
g__Bacteroides	s__Bacteroides_caecimuris	0.0151	0.0151
g__Bacteroides	s__Bacteroides_caecimuris	0.0151	0.0151
g__Bacteroides	s__Bacteroides_caecimuris	0.0151	0.0151
g__Bacteroides	s__Bacteroides_caecimuris	0.0151	0.0151
g__Bacteroides	s__Bacteroides_caecimuris	0.0151	0.0151
g__Bacteroides	s__Bacteroides_caecimuris	0.0151	0.0151
g__Bacteroides	s__Bacteroides_caecimuris	0.0151	0.0151
g__Bacteroides	s__Bacteroides_caecimuris	0.0151	0.0151
g__Bacteroides	s__Bacteroides_caecimuris	0.0151	0.0151
g__Bacteroides	s__Bacteroides_caecimuris	0.0151	0.0151
g__Bacteroides	s__Bacteroides_caecimuris	0.0151	0.0151
g__Bacteroides	s__Bacteroides_caecimuris	0.0151	0.0151
g__Bacteroides	s__Bacteroides_caecimuris	0.0151	0.0151
g__Bacteroides	s__Bacteroides_caecimuris	0.0151	0.0151
g__Bacteroides	s__Bacteroides_caecimuris	0.0151	0.0151
g__Bacteroides	s__Bacteroides_caecimuris	0.0151	0.0151

g__Bacteroides	s__Bacteroides_caecimuris	0.0151	0.0151
g__Bacteroides	s__Bacteroides_caecimuris	0.0151	0.0151
g__Bacteroides	s__Bacteroides_caecimuris	0.0151	0.0151
g__Bacteroides	s__Bacteroides_caecimuris	0.0151	0.0151
g__Bacteroides	s__Bacteroides_caecimuris	0.0151	0.0151
g__Bacteroides	s__Bacteroides_caecimuris	0.0151	0.0151
g__Bacteroides	s__Bacteroides_caecimuris	0.0151	0.0151
g__Bacteroides	s__Bacteroides_caecimuris	0.0151	0.0151
g__Bacteroides	s__Bacteroides_caecimuris	0.0151	0.0151
g__Bacteroides	s__Bacteroides_caecimuris	0.0151	0.0151
g__Bacteroides	s__Bacteroides_caecimuris	0.0151	0.0151
g__Bacteroides	s__Bacteroides_caecimuris	0.0151	0.0151
g__Bacteroides	s__Bacteroides_caecimuris	0.0151	0.0151
g__Bacteroides	s__Bacteroides_caecimuris	0.0151	0.0151
g__Bacteroides	s__Bacteroides_caecimuris	0.0151	0.0151
g__Bacteroides	s__Bacteroides_caecimuris	0.0151	0.0151
g__Bacteroides	s__Bacteroides_caecimuris	0.0151	0.0151
g__Bacteroides	s__Bacteroides_caecimuris	0.0151	0.0151
g__Bacteroides	s__Bacteroides_caecimuris	0.0151	0.0151
g__Bacteroides	s__Bacteroides_caecimuris	0.0151	0.0151
g__Bacteroides	s__Bacteroides_caecimuris	0.0151	0.0151
g__Bacteroides	s__Bacteroides_caecimuris	0.0151	0.0151
g__Bacteroides	s__Bacteroides_caecimuris	0.0151	0.0151
g__Bacteroides	s__Bacteroides_caecimuris	0.0151	0.0151
g__Bacteroides	s__Bacteroides_caecimuris	0.0151	0.0151
g__Bacteroides	s__Bacteroides_caecimuris	0.0151	0.0151
g__Bacteroides	s__Bacteroides_caecimuris	0.0151	0.0151
g__Bacteroides	s__Bacteroides_caecimuris	0.0151	0.0151
g__Bacteroides	s__Bacteroides_caecimuris	0.0151	0.0151
g__Bacteroides	s__Bacteroides_caecimuris	0.0151	0.0151
g__Bacteroides	s__Bacteroides_caecimuris	0.0151	0.0151
g__Bacteroides	s__Bacteroides_caecimuris	0.0151	0.0151
g__Bacteroides	s__Bacteroides_caecimuris	0.0151	0.0151
g__Bacteroides	s__Bacteroides_caecimuris	0.0151	0.0151
g__Bacteroides	s__Bacteroides_caecimuris	0.0151	0.0151
g__Bacteroides	s__Bacteroides_caecimuris	0.0151	0.0151
g__Bacteroides	s__Bacteroides_caecimuris	0.0151	0.0151
g__Bacteroides	s__Bacteroides_caecimuris	0.0151	0.0151
g__Bacteroides	s__Bacteroides_caecimuris	0.0151	0.0151
g__Bacteroides	s__Bacteroides_caecimuris	0.0151	0.0151
g__Bacteroides	s__Bacteroides_caecimuris	0.0151	0.0151
g__Bacteroides	s__Bacteroides_caecimuris	0.0151	0.0151
g__Bacteroides	s__Bacteroides_caecimuris	0.0151	0.0151

g__Bacteroides	s__Bacteroides_caecimuris	0.0151	0.0151
g__Bacteroides	s__Bacteroides_caecimuris	0.0151	0.0151
g__Bacteroides	s__Bacteroides_caecimuris	0.0151	0.0151
g__Bacteroides	s__Bacteroides_caecimuris	0.0151	0.0151
g__Bacteroides	s__Bacteroides_caecimuris	0.0151	0.0151
g__Bacteroides	s__Bacteroides_caecimuris	0.0151	0.0151
g__Bacteroides	s__Bacteroides_caecimuris	0.0151	0.0151
g__Bacteroides	s__Bacteroides_caecimuris	0.0151	0.0151
g__Bacteroides	s__Bacteroides_caecimuris	0.0151	0.0151
g__Bacteroides	s__Bacteroides_caecimuris	0.0151	0.0151
g__Bacteroides	s__Bacteroides_caecimuris	0.0151	0.0151
g__Bacteroides	s__Bacteroides_caecimuris	0.0151	0.0151
g__Bacteroides	s__Bacteroides_caecimuris	0.0151	0.0151
g__Bacteroides	s__Bacteroides_caecimuris	0.0151	0.0151
g__Bacteroides	s__Bacteroides_caecimuris	0.0151	0.0151
g__Bacteroides	s__Bacteroides_caecimuris	0.0151	0.0151
g__Bacteroides	s__Bacteroides_caecimuris	0.0151	0.0151
g__Bacteroides	s__Bacteroides_caecimuris	0.0151	0.0151
g__Bacteroides	s__Bacteroides_caecimuris	0.0151	0.0151
g__Bacteroides	s__Bacteroides_caecimuris	0.0151	0.0151
g__Bacteroides	s__Bacteroides_caecimuris	0.0151	0.0151
g__Bacteroides	s__Bacteroides_caecimuris	0.0151	0.0151
g__Bacteroides	s__Bacteroides_caecimuris	0.0151	0.0151
g__Bacteroides	s__Bacteroides_caecimuris	0.0151	0.0151
g__Bacteroides	s__Bacteroides_caecimuris	0.0151	0.0151
g__Bacteroides	s__Bacteroides_caecimuris	0.0151	0.0151
g__Bacteroides	s__Bacteroides_caecimuris	0.0151	0.0151
g__Bacteroides	s__Bacteroides_caecimuris	0.0151	0.0151
g__Bacteroides	s__Bacteroides_caecimuris	0.0151	0.0151
g__Bacteroides	s__Bacteroides_caecimuris	0.0151	0.0151
g__Bacteroides	s__Bacteroides_caecimuris	0.0151	0.0151
g__Bacteroides	s__Bacteroides_caecimuris	0.0151	0.0151
g__Bacteroides	s__Bacteroides_caecimuris	0.0151	0.0151
g__Bacteroides	s__Bacteroides_caecimuris	0.0151	0.0151
g__Bacteroides	s__Bacteroides_caecimuris	0.0151	0.0151
g__Bacteroides	s__Bacteroides_caecimuris	0.0151	0.0151
g__Bacteroides	s__Bacteroides_caecimuris	0.0151	0.0151
g__Bacteroides	s__Bacteroides_caecimuris	0.0151	0.0151
g__Bacteroides	s__Bacteroides_caecimuris	0.0151	0.0151
g__Bacteroides	s__Bacteroides_caecimuris	0.0151	0.0151
g__Bacteroides	s__Bacteroides_caecimuris	0.0151	0.0151
g__Bacteroides	s__Bacteroides_caecimuris	0.0151	0.0151
g__Bacteroides	s__Bacteroides_caecimuris	0.0151	0.0151

g__Bacteroides	s__Bacteroides_caecimuris	0.0151	0.0151
g__Bacteroides	s__Bacteroides_caecimuris	0.0151	0.0151
g__Bacteroides	s__Bacteroides_caecimuris	0.0151	0.0151
g__Bacteroides	s__Bacteroides_caecimuris	0.0151	0.0151
g__Bacteroides	s__Bacteroides_caecimuris	0.0151	0.0151
g__Bacteroides	s__Bacteroides_caecimuris	0.0151	0.0151
g__Bacteroides	s__Bacteroides_caecimuris	0.0151	0.0151
g__Bacteroides	s__Bacteroides_caecimuris	0.0151	0.0151
g__Bacteroides	s__Bacteroides_caecimuris	0.0151	0.0151
g__Bacteroides	s__Bacteroides_caecimuris	0.0151	0.0151
g__Bacteroides	s__Bacteroides_caecimuris	0.0151	0.0151
g__Bacteroides	s__Bacteroides_caecimuris	0.0151	0.0151
g__Bacteroides	s__Bacteroides_caecimuris	0.0151	0.0151
g__Bacteroides	s__Bacteroides_caecimuris	0.0151	0.0151
g__Bacteroides	s__Bacteroides_caecimuris	0.0151	0.0151
g__Bacteroides	s__Bacteroides_caecimuris	0.0151	0.0151
g__Bacteroides	s__Bacteroides_caecimuris	0.0151	0.0151
g__Bacteroides	s__Bacteroides_caecimuris	0.0151	0.0151
g__Bacteroides	s__Bacteroides_caecimuris	0.0151	0.0151
g__Bacteroides	s__Bacteroides_caecimuris	0.0151	0.0151
g__Bacteroides	s__Bacteroides_caecimuris	0.0151	0.0151
g__Bacteroides	s__Bacteroides_caecimuris	0.0151	0.0151
g__Bacteroides	s__Bacteroides_caecimuris	0.0151	0.0151
g__Bacteroides	s__Bacteroides_caecimuris	0.0151	0.0151
g__Bacteroides	s__Bacteroides_caecimuris	0.0151	0.0151
g__Bacteroides	s__Bacteroides_caecimuris	0.0151	0.0151
g__Bacteroides	s__Bacteroides_caecimuris	0.0151	0.0151
g__Bacteroides	s__Bacteroides_caecimuris	0.0151	0.0151
g__Bacteroides	s__Bacteroides_caecimuris	0.0151	0.0151
g__Bacteroides	s__Bacteroides_caecimuris	0.0151	0.0151
g__Bacteroides	s__Bacteroides_caecimuris	0.0151	0.0151
g__Bacteroides	s__Bacteroides_caecimuris	0.0151	0.0151
g__Bacteroides	s__Bacteroides_caecimuris	0.0151	0.0151
g__Bacteroides	s__Bacteroides_caecimuris	0.0151	0.0151
g__Bacteroides	s__Bacteroides_caecimuris	0.0151	0.0151
g__Bacteroides	s__Bacteroides_caecimuris	0.0151	0.0151
g__Bacteroides	s__Bacteroides_caecimuris	0.0151	0.0151
g__Bacteroides	s__Bacteroides_caecimuris	0.0151	0.0151
g__Bacteroides	s__Bacteroides_caecimuris	0.0151	0.0151
g__Bacteroides	s__Bacteroides_caecimuris	0.0151	0.0151
g__Bacteroides	s__Bacteroides_caecimuris	0.0151	0.0151
g__Bacteroides	s__Bacteroides_caecimuris	0.0151	0.0151
g__Bacteroides	s__Bacteroides_caecimuris	0.0151	0.0151

g__Bacteroides	s__Bacteroides_caecimuris	0.0151	0.0151
g__Bacteroides	s__Bacteroides_caecimuris	0.0151	0.0151
g__Bacteroides	s__Bacteroides_caecimuris	0.0151	0.0151
g__Bacteroides	s__Bacteroides_caecimuris	0.0151	0.0151
g__Bacteroides	s__Bacteroides_caecimuris	0.0151	0.0151
g__Bacteroides	s__Bacteroides_caecimuris	0.0151	0.0151
g__Bacteroides	s__Bacteroides_caecimuris	0.0151	0.0151
g__Bacteroides	s__Bacteroides_caecimuris	0.0151	0.0151
g__Bacteroides	s__Bacteroides_caecimuris	0.0151	0.0151
g__Bacteroides	s__Bacteroides_caecimuris	0.0151	0.0151
g__Bacteroides	s__Bacteroides_caecimuris	0.0151	0.0151
g__Bacteroides	s__Bacteroides_caecimuris	0.0151	0.0151
g__Bacteroides	s__Bacteroides_caecimuris	0.0151	0.0151
g__Bacteroides	s__Bacteroides_caecimuris	0.0151	0.0151
g__Bacteroides	s__Bacteroides_caecimuris	0.0151	0.0151
g__Bacteroides	s__Bacteroides_caecimuris	0.0151	0.0151
g__Bacteroides	s__Bacteroides_caecimuris	0.0151	0.0151
g__Bacteroides	s__Bacteroides_caecimuris	0.0151	0.0151
g__Bacteroides	s__Bacteroides_caecimuris	0.0151	0.0151
g__Bacteroides	s__Bacteroides_caecimuris	0.0151	0.0151
g__Bacteroides	s__Bacteroides_caecimuris	0.0151	0.0151
g__Bacteroides	s__Bacteroides_caecimuris	0.0151	0.0151
g__Bacteroides	s__Bacteroides_caecimuris	0.0151	0.0151
g__Bacteroides	s__Bacteroides_caecimuris	0.0151	0.0151
g__Bacteroides	s__Bacteroides_caecimuris	0.0151	0.0151
g__Bacteroides	s__Bacteroides_caecimuris	0.0151	0.0151
g__Bacteroides	s__Bacteroides_caecimuris	0.0151	0.0151
g__Bacteroides	s__Bacteroides_caecimuris	0.0151	0.0151
g__Bacteroides	s__Bacteroides_caecimuris	0.0151	0.0151
g__Bacteroides	s__Bacteroides_caecimuris	0.0151	0.0151
g__Bacteroides	s__Bacteroides_caecimuris	0.0151	0.0151
g__Bacteroides	s__Bacteroides_caecimuris	0.0151	0.0151
g__Bacteroides	s__Bacteroides_caecimuris	0.0151	0.0151
g__Bacteroides	s__Bacteroides_caecimuris	0.0151	0.0151
g__Bacteroides	s__Bacteroides_caecimuris	0.0151	0.0151
g__Bacteroides	s__Bacteroides_caecimuris	0.0151	0.0151
g__Bacteroides	s__Bacteroides_caecimuris	0.0151	0.0151
g__Bacteroides	s__Bacteroides_caecimuris	0.0151	0.0151
g__Bacteroides	s__Bacteroides_caecimuris	0.0151	0.0151
g__Bacteroides	s__Bacteroides_caecimuris	0.0151	0.0151
g__Bacteroides	s__Bacteroides_caecimuris	0.0151	0.0151
g__Bacteroides	s__Bacteroides_caecimuris	0.0151	0.0151
g__Bacteroides	s__Bacteroides_caecimuris	0.0151	0.0151

g__Bacteroides	s__Bacteroides_caecimuris	0.0151	0.0151
g__Bacteroides	s__Bacteroides_caecimuris	0.0151	0.0151
g__Bacteroides	s__Bacteroides_caecimuris	0.0151	0.0151
g__Bacteroides	s__Bacteroides_caecimuris	0.0151	0.0151
g__Bacteroides	s__Bacteroides_caecimuris	0.0151	0.0151
g__Bacteroides	s__Bacteroides_caecimuris	0.0151	0.0151
g__Bacteroides	s__Bacteroides_caecimuris	0.0151	0.0151
g__Bacteroides	s__Bacteroides_caecimuris	0.0151	0.0151
g__Bacteroides	s__Bacteroides_caecimuris	0.0151	0.0151
g__Bacteroides	s__Bacteroides_caecimuris	0.0151	0.0151
g__Bacteroides	s__Bacteroides_caecimuris	0.0151	0.0151
g__Bacteroides	s__Bacteroides_caecimuris	0.0151	0.0151
g__Bacteroides	s__Bacteroides_caecimuris	0.0151	0.0151
g__Bacteroides	s__Bacteroides_caecimuris	0.0151	0.0151
g__Bacteroides	s__Bacteroides_caecimuris	0.0151	0.0151
g__Bacteroides	s__Bacteroides_caecimuris	0.0151	0.0151
g__Bacteroides	s__Bacteroides_caecimuris	0.0151	0.0151
g__Bacteroides	s__Bacteroides_caecimuris	0.0151	0.0151
g__Bacteroides	s__Bacteroides_caecimuris	0.0151	0.0151
g__Bacteroides	s__Bacteroides_caecimuris	0.0151	0.0151
g__Bacteroides	s__Bacteroides_caecimuris	0.0151	0.0151
g__Bacteroides	s__Bacteroides_caecimuris	0.0151	0.0151
g__Bacteroides	s__Bacteroides_caecimuris	0.0151	0.0151
g__Bacteroides	s__Bacteroides_caecimuris	0.0151	0.0151
g__Bacteroides	s__Bacteroides_caecimuris	0.0151	0.0151
g__Bacteroides	s__Bacteroides_caecimuris	0.0151	0.0151
g__Bacteroides	s__Bacteroides_caecimuris	0.0151	0.0151
g__Bacteroides	s__Bacteroides_caecimuris	0.0151	0.0151
g__Bacteroides	s__Bacteroides_caecimuris	0.0151	0.0151
g__Bacteroides	s__Bacteroides_caecimuris	0.0151	0.0151
g__Bacteroides	s__Bacteroides_caecimuris	0.0151	0.0151
g__Bacteroides	s__Bacteroides_caecimuris	0.0151	0.0151
g__Bacteroides	s__Bacteroides_caecimuris	0.0151	0.0151
g__Bacteroides	s__Bacteroides_caecimuris	0.0151	0.0151
g__Bacteroides	s__Bacteroides_caecimuris	0.0151	0.0151
g__Bacteroides	s__Bacteroides_caecimuris	0.0151	0.0151
g__Bacteroides	s__Bacteroides_caecimuris	0.0151	0.0151
g__Bacteroides	s__Bacteroides_caecimuris	0.0151	0.0151
g__Bacteroides	s__Bacteroides_caecimuris	0.0151	0.0151
g__Bacteroides	s__Bacteroides_caecimuris	0.0151	0.0151
g__Bacteroides	s__Bacteroides_caecimuris	0.0151	0.0151
g__Bacteroides	s__Bacteroides_caecimuris	0.0151	0.0151
g__Bacteroides	s__Bacteroides_caecimuris	0.0151	0.0151

g__Bacteroides	s__Bacteroides_caecimuris	0.0151	0.0151
g__Bacteroides	s__Bacteroides_caecimuris	0.0151	0.0151
g__Bacteroides	s__Bacteroides_caecimuris	0.0151	0.0151
g__Bacteroides	s__Bacteroides_caecimuris	0.0151	0.0151
g__Bacteroides	s__Bacteroides_caecimuris	0.0151	0.0151
g__Bacteroides	s__Bacteroides_caecimuris	0.0151	0.0151
g__Bacteroides	s__Bacteroides_caecimuris	0.0151	0.0151
g__Bacteroides	s__Bacteroides_caecimuris	0.0151	0.0151
g__Bacteroides	s__Bacteroides_caecimuris	0.0151	0.0151
g__Bacteroides	s__Bacteroides_caecimuris	0.0151	0.0151
g__Bacteroides	s__Bacteroides_caecimuris	0.0151	0.0151
g__Bacteroides	s__Bacteroides_caecimuris	0.0151	0.0151
g__Bacteroides	s__Bacteroides_caecimuris	0.0151	0.0151
g__Bacteroides	s__Bacteroides_caecimuris	0.0151	0.0151
g__Bacteroides	s__Bacteroides_caecimuris	0.0151	0.0151
g__Bacteroides	s__Bacteroides_caecimuris	0.0151	0.0151
g__Bacteroides	s__Bacteroides_caecimuris	0.0151	0.0151
g__Bacteroides	s__Bacteroides_caecimuris	0.0151	0.0151
g__Bacteroides	s__Bacteroides_caecimuris	0.0151	0.0151
g__Bacteroides	s__Bacteroides_caecimuris	0.0151	0.0151
g__Bacteroides	s__Bacteroides_caecimuris	0.0151	0.0151
g__Bacteroides	s__Bacteroides_caecimuris	0.0151	0.0151
g__Bacteroides	s__Bacteroides_caecimuris	0.0151	0.0151
g__Bacteroides	s__Bacteroides_caecimuris	0.0151	0.0151
g__Bacteroides	s__Bacteroides_caecimuris	0.0151	0.0151
g__Bacteroides	s__Bacteroides_caecimuris	0.0151	0.0151
g__Bacteroides	s__Bacteroides_caecimuris	0.0151	0.0151
g__Bacteroides	s__Bacteroides_caecimuris	0.0151	0.0151
g__Bacteroides	s__Bacteroides_caecimuris	0.0151	0.0151
g__Bacteroides	s__Bacteroides_caecimuris	0.0151	0.0151
g__Bacteroides	s__Bacteroides_caecimuris	0.0151	0.0151
g__Bacteroides	s__Bacteroides_caecimuris	0.0151	0.0151
g__Bacteroides	s__Bacteroides_caecimuris	0.0151	0.0151
g__Bacteroides	s__Bacteroides_caecimuris	0.0151	0.0151
g__Bacteroides	s__Bacteroides_caecimuris	0.0151	0.0151
g__Bacteroides	s__Bacteroides_caecimuris	0.0151	0.0151
g__Bacteroides	s__Bacteroides_caecimuris	0.0151	0.0151
g__Bacteroides	s__Bacteroides_caecimuris	0.0151	0.0151
g__Bacteroides	s__Bacteroides_caecimuris	0.0151	0.0151
g__Bacteroides	s__Bacteroides_caecimuris	0.0151	0.0151
g__Bacteroides	s__Bacteroides_caecimuris	0.0151	0.0151
g__Bacteroides	s__Bacteroides_caecimuris	0.0151	0.0151
g__Bacteroides	s__Bacteroides_caecimuris	0.0151	0.0151

g__Bacteroides	s__Bacteroides_caecimuris	0.0151	0.0151
g__Bacteroides	s__Bacteroides_caecimuris	0.0151	0.0151
g__Bacteroides	s__Bacteroides_caecimuris	0.0151	0.0151
g__Bacteroides	s__Bacteroides_caecimuris	0.0151	0.0151
g__Bacteroides	s__Bacteroides_caecimuris	0.0151	0.0151
g__Bacteroides	s__Bacteroides_caecimuris	0.0151	0.0151
g__Bacteroides	s__Bacteroides_caecimuris	0.0151	0.0151
g__Bacteroides	s__Bacteroides_caecimuris	0.0151	0.0151
g__Bacteroides	s__Bacteroides_caecimuris	0.0151	0.0151
g__Bacteroides	s__Bacteroides_caecimuris	0.0151	0.0151
g__Bacteroides	s__Bacteroides_caecimuris	0.0151	0.0151
g__Bacteroides	s__Bacteroides_caecimuris	0.0151	0.0151
g__Bacteroides	s__Bacteroides_caecimuris	0.0151	0.0151
g__Bacteroides	s__Bacteroides_caecimuris	0.0151	0.0151
g__Bacteroides	s__Bacteroides_caecimuris	0.0151	0.0151
g__Bacteroides	s__Bacteroides_caecimuris	0.0151	0.0151
g__Bacteroides	s__Bacteroides_caecimuris	0.0151	0.0151
g__Bacteroides	s__Bacteroides_caecimuris	0.0151	0.0151
g__Bacteroides	s__Bacteroides_caecimuris	0.0151	0.0151
g__Bacteroides	s__Bacteroides_caecimuris	0.0151	0.0151
g__Bacteroides	s__Bacteroides_caecimuris	0.0151	0.0151
g__Bacteroides	s__Bacteroides_caecimuris	0.0151	0.0151
g__Bacteroides	s__Bacteroides_caecimuris	0.0151	0.0151
g__Bacteroides	s__Bacteroides_caecimuris	0.0151	0.0151
g__Bacteroides	s__Bacteroides_caecimuris	0.0151	0.0151
g__Bacteroides	s__Bacteroides_caecimuris	0.0151	0.0151
g__Bacteroides	s__Bacteroides_caecimuris	0.0151	0.0151
g__Bacteroides	s__Bacteroides_caecimuris	0.0151	0.0151
g__Bacteroides	s__Bacteroides_caecimuris	0.0151	0.0151
g__Bacteroides	s__Bacteroides_caecimuris	0.0151	0.0151
g__Bacteroides	s__Bacteroides_caecimuris	0.0151	0.0151
g__Bacteroides	s__Bacteroides_caecimuris	0.0151	0.0151
g__Bacteroides	s__Bacteroides_caecimuris	0.0151	0.0151
g__Bacteroides	s__Bacteroides_caecimuris	0.0151	0.0151
g__Bacteroides	s__Bacteroides_caecimuris	0.0151	0.0151
g__Bacteroides	s__Bacteroides_caecimuris	0.0151	0.0151
g__Bacteroides	s__Bacteroides_caecimuris	0.0151	0.0151
g__Bacteroides	s__Bacteroides_caecimuris	0.0151	0.0151
g__Bacteroides	s__Bacteroides_caecimuris	0.0151	0.0151
g__Bacteroides	s__Bacteroides_caecimuris	0.0151	0.0151
g__Bacteroides	s__Bacteroides_caecimuris	0.0151	0.0151
g__Bacteroides	s__Bacteroides_caecimuris	0.0151	0.0151
g__Bacteroides	s__Bacteroides_caecimuris	0.0151	0.0151

g__Bacteroides	s__Bacteroides_caecimuris	0.0151	0.0151
g__Bacteroides	s__Bacteroides_caecimuris	0.0151	0.0151
g__Bacteroides	s__Bacteroides_caecimuris	0.0151	0.0151
g__Bacteroides	s__Bacteroides_caecimuris	0.0151	0.0151
g__Bacteroides	s__Bacteroides_caecimuris	0.0151	0.0151
g__Bacteroides	s__Bacteroides_caecimuris	0.0151	0.0151
g__Bacteroides	s__Bacteroides_caecimuris	0.0151	0.0151
g__Bacteroides	s__Bacteroides_caecimuris	0.0151	0.0151
g__Bacteroides	s__Bacteroides_caecimuris	0.0151	0.0151
g__Bacteroides	s__Bacteroides_caecimuris	0.0151	0.0151
g__Bacteroides	s__Bacteroides_caecimuris	0.0151	0.0151
g__Bacteroides	s__Bacteroides_caecimuris	0.0151	0.0151
g__Bacteroides	s__Bacteroides_caecimuris	0.0151	0.0151
g__Bacteroides	s__Bacteroides_caecimuris	0.0151	0.0151
g__Bacteroides	s__Bacteroides_caecimuris	0.0151	0.0151
g__Bacteroides	s__Bacteroides_caecimuris	0.0151	0.0151
g__Bacteroides	s__Bacteroides_caecimuris	0.0151	0.0151
g__Bacteroides	s__Bacteroides_caecimuris	0.0151	0.0151
g__Bacteroides	s__Bacteroides_caecimuris	0.0151	0.0151
g__Bacteroides	s__Bacteroides_caecimuris	0.0151	0.0151
g__Bacteroides	s__Bacteroides_caecimuris	0.0151	0.0151
g__Bacteroides	s__Bacteroides_caecimuris	0.0151	0.0151
g__Bacteroides	s__Bacteroides_caecimuris	0.0151	0.0151
g__Bacteroides	s__Bacteroides_caecimuris	0.0151	0.0151
g__Bacteroides	s__Bacteroides_caecimuris	0.0151	0.0151
g__Bacteroides	s__Bacteroides_caecimuris	0.0151	0.0151
g__Bacteroides	s__Bacteroides_caecimuris	0.0151	0.0151
g__Bacteroides	s__Bacteroides_caecimuris	0.0151	0.0151
g__Bacteroides	s__Bacteroides_caecimuris	0.0151	0.0151
g__Bacteroides	s__Bacteroides_caecimuris	0.0151	0.0151
g__Bacteroides	s__Bacteroides_caecimuris	0.0151	0.0151
g__Bacteroides	s__Bacteroides_caecimuris	0.0151	0.0151
g__Bacteroides	s__Bacteroides_caecimuris	0.0151	0.0151
g__Bacteroides	s__Bacteroides_caecimuris	0.0151	0.0151
g__Bacteroides	s__Bacteroides_caecimuris	0.0151	0.0151
g__Bacteroides	s__Bacteroides_caecimuris	0.0151	0.0151
g__Bacteroides	s__Bacteroides_caecimuris	0.0151	0.0151
g__Bacteroides	s__Bacteroides_caecimuris	0.0151	0.0151
g__Bacteroides	s__Bacteroides_caecimuris	0.0151	0.0151
g__Bacteroides	s__Bacteroides_caecimuris	0.0151	0.0151
g__Bacteroides	s__Bacteroides_cellulosilyticus	0.0151	0.0151
g__Bacteroides	s__Bacteroides_cellulosilyticus	0.0151	0.0151
g__Bacteroides	s__Bacteroides_cellulosilyticus	0.0151	0.0151

g__Bacteroides	s__Bacteroides_cellulosilyticus	0.0151	0.0151
g__Bacteroides	s__Bacteroides_cellulosilyticus	0.0151	0.0151
g__Bacteroides	s__Bacteroides_cellulosilyticus	0.0151	0.0151
g__Bacteroides	s__Bacteroides_cellulosilyticus	0.0151	0.0151
g__Bacteroides	s__Bacteroides_cellulosilyticus	0.0151	0.0151
g__Bacteroides	s__Bacteroides_cellulosilyticus	0.0151	0.0151
g__Bacteroides	s__Bacteroides_cellulosilyticus	0.0151	0.0151
g__Bacteroides	s__Bacteroides_cellulosilyticus	0.0151	0.0151
g__Bacteroides	s__Bacteroides_cellulosilyticus	0.0151	0.0151
g__Bacteroides	s__Bacteroides_cellulosilyticus	0.0151	0.0151
g__Bacteroides	s__Bacteroides_cellulosilyticus	0.0151	0.0151
g__Bacteroides	s__Bacteroides_cellulosilyticus	0.0151	0.0151
g__Bacteroides	s__Bacteroides_cellulosilyticus	0.0151	0.0151
g__Bacteroides	s__Bacteroides_cellulosilyticus	0.0151	0.0151
g__Bacteroides	s__Bacteroides_cellulosilyticus	0.0151	0.0151
g__Bacteroides	s__Bacteroides_cellulosilyticus	0.0151	0.0151
g__Bacteroides	s__Bacteroides_cellulosilyticus	0.0151	0.0151
g__Bacteroides	s__Bacteroides_cellulosilyticus	0.0151	0.0151
g__Bacteroides	s__Bacteroides_cellulosilyticus	0.0151	0.0151
g__Bacteroides	s__Bacteroides_eggerthii	0.0151	0.0151
g__Bacteroides	s__Bacteroides_eggerthii	0.0151	0.0151
g__Bacteroides	s__Bacteroides_eggerthii	0.0151	0.0151
g__Bacteroides	s__Bacteroides_eggerthii	0.0151	0.0151
g__Bacteroides	s__Bacteroides_eggerthii	0.0151	0.0151
g__Bacteroides	s__Bacteroides_eggerthii	0.0151	0.0151
g__Bacteroides	s__Bacteroides_eggerthii	0.0151	0.0151
g__Bacteroides	s__Bacteroides_eggerthii	0.0151	0.0151
g__Bacteroides	s__Bacteroides_eggerthii	0.0151	0.0151
g__Bacteroides	s__Bacteroides_eggerthii	0.0151	0.0151
g__Bacteroides	s__Bacteroides_eggerthii	0.0151	0.0151
g__Bacteroides	s__Bacteroides_eggerthii	0.0151	0.0151
g__Bacteroides	s__Bacteroides_eggerthii	0.0151	0.0151
g__Bacteroides	s__Bacteroides_eggerthii	0.0151	0.0151
g__Bacteroides	s__Bacteroides_faecis	0.0151	0.0151
g__Bacteroides	s__Bacteroides_faecis	0.0151	0.0151
g__Bacteroides	s__Bacteroides_faecis	0.0151	0.0151
g__Bacteroides	s__Bacteroides_faecis	0.0151	0.0151
g__Bacteroides	s__Bacteroides_faecis	0.0151	0.0151
g__Bacteroides	s__Bacteroides_faecis	0.0151	0.0151
g__Bacteroides	s__Bacteroides_faecis	0.0151	0.0151
g__Bacteroides	s__Bacteroides_faecis	0.0151	0.0151
g__Bacteroides	s__Bacteroides_faecis	0.0151	0.0151
g__Bacteroides	s__Bacteroides_faecis	0.0151	0.0151

g__Bacteroides	s__Bacteroides_faecis	0.0151	0.0151
g__Bacteroides	s__Bacteroides_faecis	0.0151	0.0151
g__Bacteroides	s__Bacteroides_faecis	0.0151	0.0151
g__Bacteroides	s__Bacteroides_faecis	0.0151	0.0151
g__Bacteroides	s__Bacteroides_faecis	0.0151	0.0151
g__Bacteroides	s__Bacteroides_faecis	0.0151	0.0151
g__Bacteroides	s__Bacteroides_faecis	0.0151	0.0151
g__Bacteroides	s__Bacteroides_faecis	0.0151	0.0151
g__Bacteroides	s__Bacteroides_faecis	0.0151	0.0151
g__Bacteroides	s__Bacteroides_faecis	0.0151	0.0151
g__Bacteroides	s__Bacteroides_faecis	0.0151	0.0151
g__Bacteroides	s__Bacteroides_faecis	0.0151	0.0151
g__Bacteroides	s__Bacteroides_faecis	0.0151	0.0151
g__Bacteroides	s__Bacteroides_faecis	0.0151	0.0151
g__Bacteroides	s__Bacteroides_faecis	0.0151	0.0151
g__Bacteroides	s__Bacteroides_faecis	0.0151	0.0151
g__Bacteroides	s__Bacteroides_faecis	0.0151	0.0151
g__Bacteroides	s__Bacteroides_faecis	0.0151	0.0151
g__Bacteroides	s__Bacteroides_faecis	0.0151	0.0151
g__Bacteroides	s__Bacteroides_faecis	0.0151	0.0151
g__Bacteroides	s__Bacteroides_faecis	0.0151	0.0151
g__Bacteroides	s__Bacteroides_faecis	0.0151	0.0151
g__Bacteroides	s__Bacteroides_faecis	0.0151	0.0151
g__Bacteroides	s__Bacteroides_faecis	0.0151	0.0151
g__Bacteroides	s__Bacteroides_faecis	0.0151	0.0151
g__Bacteroides	s__Bacteroides_faecis	0.0151	0.0151
g__Bacteroides	s__Bacteroides_faecis	0.0151	0.0151
g__Bacteroides	s__Bacteroides_faecis	0.0151	0.0151
g__Bacteroides	s__Bacteroides_faecis	0.0151	0.0151
g__Bacteroides	s__Bacteroides_faecis	0.0151	0.0151
g__Bacteroides	s__Bacteroides_faecis	0.0151	0.0151
g__Bacteroides	s__Bacteroides_faecis	0.0151	0.0151
g__Bacteroides	s__Bacteroides_faecis	0.0151	0.0151
g__Bacteroides	s__Bacteroides_faecis	0.0151	0.0151
g__Bacteroides	s__Bacteroides_faecis	0.0151	0.0151
g__Bacteroides	s__Bacteroides_faecis	0.0151	0.0151
g__Bacteroides	s__Bacteroides_faecis	0.0151	0.0151
g__Bacteroides	s__Bacteroides_faecis	0.0151	0.0151
g__Bacteroides	s__Bacteroides_faecis	0.0151	0.0151
g__Bacteroides	s__Bacteroides_faecis	0.0151	0.0151
g__Bacteroides	s__Bacteroides_faecis	0.0151	0.0151
g__Bacteroides	s__Bacteroides_faecis	0.0151	0.0151
g__Bacteroides	s__Bacteroides_faecis	0.0151	0.0151

g__Bacteroides	s__Bacteroides_faecis	0.0151	0.0151
g__Bacteroides	s__Bacteroides_faecis	0.0151	0.0151
g__Bacteroides	s__Bacteroides_faecis	0.0151	0.0151
g__Bacteroides	s__Bacteroides_faecis	0.0151	0.0151
g__Bacteroides	s__Bacteroides_faecis	0.0151	0.0151
g__Bacteroides	s__Bacteroides_faecis	0.0151	0.0151
g__Bacteroides	s__Bacteroides_faecis	0.0151	0.0151
g__Bacteroides	s__Bacteroides_faecis	0.0151	0.0151
g__Bacteroides	s__Bacteroides_faecis	0.0151	0.0151
g__Bacteroides	s__Bacteroides_faecis	0.0151	0.0151
g__Bacteroides	s__Bacteroides_faecis	0.0151	0.0151
g__Bacteroides	s__Bacteroides_faecis	0.0151	0.0151
g__Bacteroides	s__Bacteroides_faecis	0.0151	0.0151
g__Bacteroides	s__Bacteroides_faecis	0.0151	0.0151
g__Bacteroides	s__Bacteroides_faecis	0.0151	0.0151
g__Bacteroides	s__Bacteroides_faecis	0.0151	0.0151
g__Bacteroides	s__Bacteroides_faecis	0.0151	0.0151
g__Bacteroides	s__Bacteroides_faecis	0.0151	0.0151
g__Bacteroides	s__Bacteroides_finegoldii	0.0151	0.0151
g__Bacteroides	s__Bacteroides_finegoldii	0.0151	0.0151
g__Bacteroides	s__Bacteroides_finegoldii	0.0151	0.0151
g__Bacteroides	s__Bacteroides_finegoldii	0.0151	0.0151
g__Bacteroides	s__Bacteroides_finegoldii	0.0151	0.0151
g__Bacteroides	s__Bacteroides_finegoldii	0.0151	0.0151
g__Bacteroides	s__Bacteroides_finegoldii	0.0151	0.0151
g__Bacteroides	s__Bacteroides_finegoldii	0.0151	0.0151
g__Bacteroides	s__Bacteroides_finegoldii	0.0151	0.0151
g__Bacteroides	s__Bacteroides_finegoldii	0.0151	0.0151
g__Bacteroides	s__Bacteroides_finegoldii	0.0151	0.0151
g__Bacteroides	s__Bacteroides_finegoldii	0.0151	0.0151
g__Bacteroides	s__Bacteroides_finegoldii	0.0151	0.0151
g__Bacteroides	s__Bacteroides_finegoldii	0.0151	0.0151
g__Bacteroides	s__Bacteroides_finegoldii	0.0151	0.0151
g__Bacteroides	s__Bacteroides_finegoldii	0.0151	0.0151
g__Bacteroides	s__Bacteroides_finegoldii	0.0151	0.0151
g__Bacteroides	s__Bacteroides_finegoldii	0.0151	0.0151
g__Bacteroides	s__Bacteroides_finegoldii	0.0151	0.0151
g__Bacteroides	s__Bacteroides_finegoldii	0.0151	0.0151
g__Bacteroides	s__Bacteroides_finegoldii	0.0151	0.0151
g__Bacteroides	s__Bacteroides_finegoldii	0.0151	0.0151
g__Bacteroides	s__Bacteroides_finegoldii	0.0151	0.0151
g__Bacteroides	s__Bacteroides_finegoldii	0.0151	0.0151
g__Bacteroides	s__Bacteroides_finegoldii	0.0151	0.0151

g__Bacteroides	s__Bacteroides_finegoldii	0.0151	0.0151
g__Bacteroides	s__Bacteroides_finegoldii	0.0151	0.0151
g__Bacteroides	s__Bacteroides_finegoldii	0.0151	0.0151
g__Bacteroides	s__Bacteroides_finegoldii	0.0151	0.0151
g__Bacteroides	s__Bacteroides_finegoldii	0.0151	0.0151
g__Bacteroides	s__Bacteroides_finegoldii	0.0151	0.0151
g__Bacteroides	s__Bacteroides_finegoldii	0.0151	0.0151
g__Bacteroides	s__Bacteroides_finegoldii	0.0151	0.0151
g__Bacteroides	s__Bacteroides_finegoldii	0.0151	0.0151
g__Bacteroides	s__Bacteroides_finegoldii	0.0151	0.0151
g__Bacteroides	s__Bacteroides_finegoldii	0.0151	0.0151
g__Bacteroides	s__Bacteroides_finegoldii	0.0151	0.0151
g__Bacteroides	s__Bacteroides_finegoldii	0.0151	0.0151
g__Bacteroides	s__Bacteroides_finegoldii	0.0151	0.0151
g__Bacteroides	s__Bacteroides_finegoldii	0.0151	0.0151
g__Bacteroides	s__Bacteroides_finegoldii	0.0151	0.0151
g__Bacteroides	s__Bacteroides_finegoldii	0.0151	0.0151
g__Bacteroides	s__Bacteroides_finegoldii	0.0151	0.0151
g__Bacteroides	s__Bacteroides_finegoldii	0.0151	0.0151
g__Bacteroides	s__Bacteroides_finegoldii	0.0151	0.0151
g__Bacteroides	s__Bacteroides_finegoldii	0.0151	0.0151
g__Bacteroides	s__Bacteroides_finegoldii	0.0151	0.0151
g__Bacteroides	s__Bacteroides_finegoldii	0.0151	0.0151
g__Bacteroides	s__Bacteroides_finegoldii	0.0151	0.0151
g__Bacteroides	s__Bacteroides_finegoldii	0.0151	0.0151
g__Bacteroides	s__Bacteroides_finegoldii	0.0151	0.0151
g__Bacteroides	s__Bacteroides_finegoldii	0.0151	0.0151
g__Bacteroides	s__Bacteroides_finegoldii	0.0151	0.0151
g__Bacteroides	s__Bacteroides_finegoldii	0.0151	0.0151
g__Bacteroides	s__Bacteroides_finegoldii	0.0151	0.0151
g__Bacteroides	s__Bacteroides_finegoldii	0.0151	0.0151
g__Bacteroides	s__Bacteroides_finegoldii	0.0151	0.0151
g__Bacteroides	s__Bacteroides_finegoldii	0.0151	0.0151
g__Bacteroides	s__Bacteroides_finegoldii	0.0151	0.0151
g__Bacteroides	s__Bacteroides_finegoldii	0.0151	0.0151
g__Bacteroides	s__Bacteroides_finegoldii	0.0151	0.0151
g__Bacteroides	s__Bacteroides_finegoldii	0.0151	0.0151
g__Bacteroides	s__Bacteroides_finegoldii	0.0151	0.0151
g__Bacteroides	s__Bacteroides_finegoldii	0.0151	0.0151
g__Bacteroides	s__Bacteroides_finegoldii	0.0151	0.0151
g__Bacteroides	s__Bacteroides_finegoldii	0.0151	0.0151
g__Bacteroides	s__Bacteroides_finegoldii	0.0151	0.0151
g__Bacteroides	s__Bacteroides_finegoldii	0.0151	0.0151

g__Bacteroides	s__Bacteroides_finegoldii	0.0151	0.0151
g__Bacteroides	s__Bacteroides_finegoldii	0.0151	0.0151
g__Bacteroides	s__Bacteroides_finegoldii	0.0151	0.0151
g__Bacteroides	s__Bacteroides_finegoldii	0.0151	0.0151
g__Bacteroides	s__Bacteroides_finegoldii	0.0151	0.0151
g__Bacteroides	s__Bacteroides_finegoldii	0.0151	0.0151
g__Bacteroides	s__Bacteroides_finegoldii	0.0151	0.0151
g__Bacteroides	s__Bacteroides_finegoldii	0.0151	0.0151
g__Bacteroides	s__Bacteroides_finegoldii	0.0151	0.0151
g__Bacteroides	s__Bacteroides_finegoldii	0.0151	0.0151
g__Bacteroides	s__Bacteroides_finegoldii	0.0151	0.0151
g__Bacteroides	s__Bacteroides_finegoldii	0.0151	0.0151
g__Bacteroides	s__Bacteroides_finegoldii	0.0151	0.0151
g__Bacteroides	s__Bacteroides_finegoldii	0.0151	0.0151
g__Bacteroides	s__Bacteroides_finegoldii	0.0151	0.0151
g__Bacteroides	s__Bacteroides_finegoldii	0.0151	0.0151
g__Bacteroides	s__Bacteroides_finegoldii	0.0151	0.0151
g__Bacteroides	s__Bacteroides_finegoldii	0.0151	0.0151
g__Bacteroides	s__Bacteroides_finegoldii	0.0151	0.0151
g__Bacteroides	s__Bacteroides_finegoldii	0.0151	0.0151
g__Bacteroides	s__Bacteroides_finegoldii	0.0151	0.0151
g__Bacteroides	s__Bacteroides_finegoldii	0.0151	0.0151
g__Bacteroides	s__Bacteroides_finegoldii	0.0151	0.0151
g__Bacteroides	s__Bacteroides_finegoldii	0.0151	0.0151
g__Bacteroides	s__Bacteroides_finegoldii	0.0151	0.0151
g__Bacteroides	s__Bacteroides_finegoldii	0.0151	0.0151
g__Bacteroides	s__Bacteroides_finegoldii	0.0151	0.0151
g__Bacteroides	s__Bacteroides_finegoldii	0.0151	0.0151
g__Bacteroides	s__Bacteroides_finegoldii	0.0151	0.0151
g__Bacteroides	s__Bacteroides_finegoldii	0.0151	0.0151
g__Bacteroides	s__Bacteroides_finegoldii	0.0151	0.0151
g__Bacteroides	s__Bacteroides_finegoldii	0.0151	0.0151
g__Bacteroides	s__Bacteroides_finegoldii	0.0151	0.0151
g__Bacteroides	s__Bacteroides_finegoldii	0.0151	0.0151
g__Bacteroides	s__Bacteroides_finegoldii	0.0151	0.0151
g__Bacteroides	s__Bacteroides_finegoldii	0.0151	0.0151
g__Bacteroides	s__Bacteroides_finegoldii	0.0151	0.0151
g__Bacteroides	s__Bacteroides_finegoldii	0.0151	0.0151
g__Bacteroides	s__Bacteroides_finegoldii	0.0151	0.0151
g__Bacteroides	s__Bacteroides_finegoldii	0.0151	0.0151
g__Bacteroides	s__Bacteroides_finegoldii	0.0151	0.0151
g__Bacteroides	s__Bacteroides_finegoldii	0.0151	0.0151
g__Bacteroides	s__Bacteroides_finegoldii	0.0151	0.0151

g__Bacteroides	s__Bacteroides_finegoldii	0.0151	0.0151
g__Bacteroides	s__Bacteroides_finegoldii	0.0151	0.0151
g__Bacteroides	s__Bacteroides_finegoldii	0.0151	0.0151
g__Bacteroides	s__Bacteroides_finegoldii	0.0151	0.0151
g__Bacteroides	s__Bacteroides_finegoldii	0.0151	0.0151
g__Bacteroides	s__Bacteroides_finegoldii	0.0151	0.0151
g__Bacteroides	s__Bacteroides_finegoldii	0.0151	0.0151
g__Bacteroides	s__Bacteroides_finegoldii	0.0151	0.0151
g__Bacteroides	s__Bacteroides_finegoldii	0.0151	0.0151
g__Bacteroides	s__Bacteroides_finegoldii	0.0151	0.0151
g__Bacteroides	s__Bacteroides_finegoldii	0.0151	0.0151
g__Bacteroides	s__Bacteroides_finegoldii	0.0151	0.0151
g__Bacteroides	s__Bacteroides_finegoldii	0.0151	0.0151
g__Bacteroides	s__Bacteroides_finegoldii	0.0151	0.0151
g__Bacteroides	s__Bacteroides_finegoldii	0.0151	0.0151
g__Bacteroides	s__Bacteroides_finegoldii	0.0151	0.0151
g__Bacteroides	s__Bacteroides_finegoldii	0.0151	0.0151
g__Bacteroides	s__Bacteroides_finegoldii	0.0151	0.0151
g__Bacteroides	s__Bacteroides_finegoldii	0.0151	0.0151
g__Bacteroides	s__Bacteroides_finegoldii	0.0151	0.0151
g__Bacteroides	s__Bacteroides_finegoldii	0.0151	0.0151
g__Bacteroides	s__Bacteroides_finegoldii	0.0151	0.0151
g__Bacteroides	s__Bacteroides_finegoldii	0.0151	0.0151
g__Bacteroides	s__Bacteroides_fragilis	0.0151	0.0151
g__Bacteroides	s__Bacteroides_fragilis	0.0151	0.0151
g__Bacteroides	s__Bacteroides_fragilis	0.0151	0.0151
g__Bacteroides	s__Bacteroides_fragilis	0.0151	0.0151
g__Bacteroides	s__Bacteroides_fragilis	0.0151	0.0151
g__Bacteroides	s__Bacteroides_fragilis	0.0151	0.0151
g__Bacteroides	s__Bacteroides_fragilis	0.0151	0.0151
g__Bacteroides	s__Bacteroides_fragilis	0.0151	0.0151
g__Bacteroides	s__Bacteroides_fragilis	0.0151	0.0151
g__Bacteroides	s__Bacteroides_fragilis	0.0151	0.0151
g__Bacteroides	s__Bacteroides_fragilis	0.0151	0.0151
g__Bacteroides	s__Bacteroides_fragilis	0.0151	0.0151
g__Bacteroides	s__Bacteroides_fragilis	0.0151	0.0151
g__Bacteroides	s__Bacteroides_fragilis	0.0151	0.0151
g__Bacteroides	s__Bacteroides_fragilis	0.0151	0.0151
g__Bacteroides	s__Bacteroides_fragilis	0.0151	0.0151
g__Bacteroides	s__Bacteroides_fragilis	0.0151	0.0151
g__Bacteroides	s__Bacteroides_fragilis	0.0151	0.0151
g__Bacteroides	s__Bacteroides_fragilis	0.0151	0.0151
g__Bacteroides	s__Bacteroides_fragilis	0.0151	0.0151

g__Bacteroides	s__Bacteroides_fragilis	0.0151	0.0151
g__Bacteroides	s__Bacteroides_fragilis	0.0151	0.0151
g__Bacteroides	s__Bacteroides_fragilis	0.0151	0.0151
g__Bacteroides	s__Bacteroides_fragilis	0.0151	0.0151
g__Bacteroides	s__Bacteroides_fragilis	0.0151	0.0151
g__Bacteroides	s__Bacteroides_fragilis	0.0151	0.0151
g__Bacteroides	s__Bacteroides_fragilis	0.0151	0.0151
g__Bacteroides	s__Bacteroides_fragilis	0.0151	0.0151
g__Bacteroides	s__Bacteroides_fragilis	0.0151	0.0151
g__Bacteroides	s__Bacteroides_fragilis	0.0151	0.0151
g__Bacteroides	s__Bacteroides_fragilis	0.0151	0.0151
g__Bacteroides	s__Bacteroides_fragilis	0.0151	0.0151
g__Bacteroides	s__Bacteroides_fragilis	0.0151	0.0151
g__Bacteroides	s__Bacteroides_fragilis	0.0151	0.0151
g__Bacteroides	s__Bacteroides_fragilis	0.0151	0.0151
g__Bacteroides	s__Bacteroides_fragilis	0.0151	0.0151
g__Bacteroides	s__Bacteroides_fragilis	0.0151	0.0151
g__Bacteroides	s__Bacteroides_fragilis	0.0151	0.0151
g__Bacteroides	s__Bacteroides_fragilis	0.0151	0.0151
g__Bacteroides	s__Bacteroides_fragilis	0.0151	0.0151
g__Bacteroides	s__Bacteroides_fragilis	0.0151	0.0151
g__Bacteroides	s__Bacteroides_fragilis	0.0151	0.0151
g__Bacteroides	s__Bacteroides_fragilis	0.0151	0.0151
g__Bacteroides	s__Bacteroides_fragilis	0.0151	0.0151
g__Bacteroides	s__Bacteroides_fragilis	0.0151	0.0151
g__Bacteroides	s__Bacteroides_fragilis	0.0151	0.0151
g__Bacteroides	s__Bacteroides_fragilis	0.0151	0.0151
g__Bacteroides	s__Bacteroides_fragilis	0.0151	0.0151
g__Bacteroides	s__Bacteroides_fragilis	0.0151	0.0151
g__Bacteroides	s__Bacteroides_fragilis	0.0151	0.0151
g__Bacteroides	s__Bacteroides_fragilis	0.0151	0.0151
g__Bacteroides	s__Bacteroides_fragilis	0.0151	0.0151
g__Bacteroides	s__Bacteroides_fragilis	0.0151	0.0151
g__Bacteroides	s__Bacteroides_fragilis	0.0151	0.0151
g__Bacteroides	s__Bacteroides_fragilis	0.0151	0.0151
g__Bacteroides	s__Bacteroides_fragilis	0.0151	0.0151
g__Bacteroides	s__Bacteroides_fragilis	0.0151	0.0151
g__Bacteroides	s__Bacteroides_fragilis	0.0151	0.0151
g__Bacteroides	s__Bacteroides_fragilis	0.0151	0.0151
g__Bacteroides	s__Bacteroides_fragilis	0.0151	0.0151
g__Bacteroides	s__Bacteroides_fragilis	0.0151	0.0151
g__Bacteroides	s__Bacteroides_fragilis	0.0151	0.0151
g__Bacteroides	s__Bacteroides_fragilis	0.0151	0.0151

g__Bacteroides	s__Bacteroides_fragilis	0.0151	0.0151
g__Bacteroides	s__Bacteroides_fragilis	0.0151	0.0151
g__Bacteroides	s__Bacteroides_fragilis	0.0151	0.0151
g__Bacteroides	s__Bacteroides_fragilis	0.0151	0.0151
g__Bacteroides	s__Bacteroides_fragilis	0.0151	0.0151
g__Bacteroides	s__Bacteroides_fragilis	0.0151	0.0151
g__Bacteroides	s__Bacteroides_fragilis	0.0151	0.0151
g__Bacteroides	s__Bacteroides_fragilis	0.0151	0.0151
g__Bacteroides	s__Bacteroides_fragilis	0.0151	0.0151
g__Bacteroides	s__Bacteroides_fragilis	0.0151	0.0151
g__Bacteroides	s__Bacteroides_fragilis	0.0151	0.0151
g__Bacteroides	s__Bacteroides_fragilis	0.0151	0.0151
g__Bacteroides	s__Bacteroides_fragilis	0.0151	0.0151
g__Bacteroides	s__Bacteroides_fragilis	0.0151	0.0151
g__Bacteroides	s__Bacteroides_fragilis	0.0151	0.0151
g__Bacteroides	s__Bacteroides_fragilis	0.0151	0.0151
g__Bacteroides	s__Bacteroides_fragilis	0.0151	0.0151
g__Bacteroides	s__Bacteroides_fragilis	0.0151	0.0151
g__Bacteroides	s__Bacteroides_fragilis	0.0151	0.0151
g__Bacteroides	s__Bacteroides_fragilis	0.0151	0.0151
g__Bacteroides	s__Bacteroides_fragilis	0.0151	0.0151
g__Bacteroides	s__Bacteroides_fragilis	0.0151	0.0151
g__Bacteroides	s__Bacteroides_fragilis	0.0151	0.0151
g__Bacteroides	s__Bacteroides_fragilis	0.0151	0.0151
g__Bacteroides	s__Bacteroides_fragilis	0.0151	0.0151
g__Bacteroides	s__Bacteroides_fragilis	0.0151	0.0151
g__Bacteroides	s__Bacteroides_fragilis	0.0151	0.0151
g__Bacteroides	s__Bacteroides_fragilis	0.0151	0.0151
g__Bacteroides	s__Bacteroides_fragilis	0.0151	0.0151
g__Bacteroides	s__Bacteroides_fragilis	0.0151	0.0151
g__Bacteroides	s__Bacteroides_fragilis	0.0151	0.0151
g__Bacteroides	s__Bacteroides_fragilis	0.0151	0.0151
g__Bacteroides	s__Bacteroides_fragilis	0.0151	0.0151
g__Bacteroides	s__Bacteroides_fragilis	0.0151	0.0151
g__Bacteroides	s__Bacteroides_fragilis	0.0151	0.0151
g__Bacteroides	s__Bacteroides_fragilis	0.0151	0.0151
g__Bacteroides	s__Bacteroides_fragilis	0.0151	0.0151
g__Bacteroides	s__Bacteroides_fragilis	0.0151	0.0151
g__Bacteroides	s__Bacteroides_fragilis	0.0151	0.0151
g__Bacteroides	s__Bacteroides_fragilis	0.0151	0.0151
g__Bacteroides	s__Bacteroides_fragilis	0.0151	0.0151
g__Bacteroides	s__Bacteroides_fragilis	0.0151	0.0151
g__Bacteroides	s__Bacteroides_fragilis	0.0151	0.0151

g__Bacteroides	s__Bacteroides_fragilis	0.0151	0.0151
g__Bacteroides	s__Bacteroides_fragilis	0.0151	0.0151
g__Bacteroides	s__Bacteroides_fragilis	0.0151	0.0151
g__Bacteroides	s__Bacteroides_fragilis	0.0151	0.0151
g__Bacteroides	s__Bacteroides_fragilis	0.0151	0.0151
g__Bacteroides	s__Bacteroides_fragilis	0.0151	0.0151
g__Bacteroides	s__Bacteroides_fragilis	0.0151	0.0151
g__Bacteroides	s__Bacteroides_fragilis	0.0151	0.0151
g__Bacteroides	s__Bacteroides_fragilis	0.0151	0.0151
g__Bacteroides	s__Bacteroides_fragilis	0.0151	0.0151
g__Bacteroides	s__Bacteroides_fragilis	0.0151	0.0151
g__Bacteroides	s__Bacteroides_fragilis	0.0151	0.0151
g__Bacteroides	s__Bacteroides_fragilis	0.0151	0.0151
g__Bacteroides	s__Bacteroides_fragilis	0.0151	0.0151
g__Bacteroides	s__Bacteroides_fragilis	0.0151	0.0151
g__Bacteroides	s__Bacteroides_fragilis	0.0151	0.0151
g__Bacteroides	s__Bacteroides_fragilis	0.0151	0.0151
g__Bacteroides	s__Bacteroides_fragilis	0.0151	0.0151
g__Bacteroides	s__Bacteroides_fragilis	0.0151	0.0151
g__Bacteroides	s__Bacteroides_fragilis	0.0151	0.0151
g__Bacteroides	s__Bacteroides_fragilis	0.0151	0.0151
g__Bacteroides	s__Bacteroides_fragilis	0.0151	0.0151
g__Bacteroides	s__Bacteroides_fragilis	0.0151	0.0151
g__Bacteroides	s__Bacteroides_fragilis	0.0151	0.0151
g__Bacteroides	s__Bacteroides_fragilis	0.0151	0.0151
g__Bacteroides	s__Bacteroides_fragilis	0.0151	0.0151
g__Bacteroides	s__Bacteroides_fragilis	0.0151	0.0151
g__Bacteroides	s__Bacteroides_fragilis	0.0151	0.0151
g__Bacteroides	s__Bacteroides_fragilis	0.0151	0.0151
g__Bacteroides	s__Bacteroides_fragilis	0.0151	0.0151
g__Bacteroides	s__Bacteroides_fragilis	0.0151	0.0151
g__Bacteroides	s__Bacteroides_fragilis	0.0151	0.0151
g__Bacteroides	s__Bacteroides_fragilis	0.0151	0.0151
g__Bacteroides	s__Bacteroides_fragilis	0.0151	0.0151
g__Bacteroides	s__Bacteroides_fragilis	0.0151	0.0151
g__Bacteroides	s__Bacteroides_fragilis	0.0151	0.0151
g__Bacteroides	s__Bacteroides_fragilis	0.0151	0.0151
g__Bacteroides	s__Bacteroides_fragilis	0.0151	0.0151
g__Bacteroides	s__Bacteroides_fragilis	0.0151	0.0151
g__Bacteroides	s__Bacteroides_fragilis	0.0151	0.0151
g__Bacteroides	s__Bacteroides_fragilis	0.0151	0.0151
g__Bacteroides	s__Bacteroides_fragilis	0.0151	0.0151
g__Bacteroides	s__Bacteroides_fragilis	0.0151	0.0151

g__Bacteroides	s__Bacteroides_fragilis	0.0151	0.0151
g__Bacteroides	s__Bacteroides_fragilis	0.0151	0.0151
g__Bacteroides	s__Bacteroides_fragilis	0.0151	0.0151
g__Bacteroides	s__Bacteroides_graminisolvens	0.0151	0.0151
g__Bacteroides	s__Bacteroides_helcogenes	0.0151	0.0151
g__Bacteroides	s__Bacteroides_helcogenes	0.0151	0.0151
g__Bacteroides	s__Bacteroides_helcogenes	0.0151	0.0151
g__Bacteroides	s__Bacteroides_helcogenes	0.0151	0.0151
g__Bacteroides	s__Bacteroides_helcogenes	0.0151	0.0151
g__Bacteroides	s__Bacteroides_helcogenes	0.0151	0.0151
g__Bacteroides	s__Bacteroides_helcogenes	0.0151	0.0151
g__Bacteroides	s__Bacteroides_helcogenes	0.0151	0.0151
g__Bacteroides	s__Bacteroides_helcogenes	0.0151	0.0151
g__Bacteroides	s__Bacteroides_helcogenes	0.0151	0.0151
g__Bacteroides	s__Bacteroides_helcogenes	0.0151	0.0151
g__Bacteroides	s__Bacteroides_helcogenes	0.0151	0.0151
g__Bacteroides	s__Bacteroides_heparinolyticus	0.0151	0.0151
g__Bacteroides	s__Bacteroides_heparinolyticus	0.0151	0.0151
g__Bacteroides	s__Bacteroides_heparinolyticus	0.0151	0.0151
g__Bacteroides	s__Bacteroides_heparinolyticus	0.0151	0.0151
g__Bacteroides	s__Bacteroides_heparinolyticus	0.0151	0.0151
g__Bacteroides	s__Bacteroides_intestinalis	0.0151	0.0151
g__Bacteroides	s__Bacteroides_intestinalis	0.0151	0.0151
g__Bacteroides	s__Bacteroides_intestinalis	0.0151	0.0151
g__Bacteroides	s__Bacteroides_intestinalis	0.0151	0.0151
g__Bacteroides	s__Bacteroides_intestinalis	0.0151	0.0151
g__Bacteroides	s__Bacteroides_intestinalis	0.0151	0.0151
g__Bacteroides	s__Bacteroides_intestinalis	0.0151	0.0151
g__Bacteroides	s__Bacteroides_intestinalis	0.0151	0.0151
g__Bacteroides	s__Bacteroides_intestinalis	0.0151	0.0151
g__Bacteroides	s__Bacteroides_intestinalis	0.0151	0.0151
g__Bacteroides	s__Bacteroides_intestinalis	0.0151	0.0151
g__Bacteroides	s__Bacteroides_intestinalis	0.0151	0.0151
g__Bacteroides	s__Bacteroides_intestinalis	0.0151	0.0151
g__Bacteroides	s__Bacteroides_intestinalis	0.0151	0.0151
g__Bacteroides	s__Bacteroides_intestinalis	0.0151	0.0151
g__Bacteroides	s__Bacteroides_intestinalis	0.0151	0.0151
g__Bacteroides	s__Bacteroides_intestinalis	0.0151	0.0151
g__Bacteroides	s__Bacteroides_intestinalis	0.0151	0.0151
g__Bacteroides	s__Bacteroides_intestinalis	0.0151	0.0151
g__Bacteroides	s__Bacteroides_intestinalis	0.0151	0.0151
g__Bacteroides	s__Bacteroides_intestinalis	0.0151	0.0151
g__Bacteroides	s__Bacteroides_intestinalis	0.0151	0.0151

g__Bacteroides	s__Bacteroides_intestinalis	0.0151	0.0151
g__Bacteroides	s__Bacteroides_intestinalis	0.0151	0.0151
g__Bacteroides	s__Bacteroides_intestinalis	0.0151	0.0151
g__Bacteroides	s__Bacteroides_intestinalis	0.0151	0.0151
g__Bacteroides	s__Bacteroides_intestinalis	0.0151	0.0151
g__Bacteroides	s__Bacteroides_intestinalis	0.0151	0.0151
g__Bacteroides	s__Bacteroides_intestinalis	0.0151	0.0151
g__Bacteroides	s__Bacteroides_intestinalis	0.0151	0.0151
g__Bacteroides	s__Bacteroides_intestinalis	0.0151	0.0151
g__Bacteroides	s__Bacteroides_intestinalis	0.0151	0.0151
g__Bacteroides	s__Bacteroides_intestinalis	0.0151	0.0151
g__Bacteroides	s__Bacteroides_intestinalis	0.0151	0.0151
g__Bacteroides	s__Bacteroides_intestinalis	0.0151	0.0151
g__Bacteroides	s__Bacteroides_intestinalis	0.0151	0.0151
g__Bacteroides	s__Bacteroides_intestinalis	0.0151	0.0151
g__Bacteroides	s__Bacteroides_intestinalis	0.0151	0.0151
g__Bacteroides	s__Bacteroides_intestinalis	0.0151	0.0151
g__Bacteroides	s__Bacteroides_intestinalis	0.0151	0.0151
g__Bacteroides	s__Bacteroides_intestinalis	0.0151	0.0151
g__Bacteroides	s__Bacteroides_intestinalis	0.0151	0.0151
g__Bacteroides	s__Bacteroides_intestinalis	0.0151	0.0151
g__Bacteroides	s__Bacteroides_intestinalis	0.0151	0.0151
g__Bacteroides	s__Bacteroides_intestinalis	0.0151	0.0151
g__Bacteroides	s__Bacteroides_intestinalis	0.0151	0.0151
g__Bacteroides	s__Bacteroides_intestinalis	0.0151	0.0151
g__Bacteroides	s__Bacteroides_intestinalis	0.0151	0.0151
g__Bacteroides	s__Bacteroides_intestinalis	0.0151	0.0151
g__Bacteroides	s__Bacteroides_intestinalis	0.0151	0.0151
g__Bacteroides	s__Bacteroides_intestinalis	0.0151	0.0151
g__Bacteroides	s__Bacteroides_nordii	0.0151	0.0151
g__Bacteroides	s__Bacteroides_nordii	0.0151	0.0151
g__Bacteroides	s__Bacteroides_nordii	0.0151	0.0151
g__Bacteroides	s__Bacteroides_nordii	0.0151	0.0151
g__Bacteroides	s__Bacteroides_nordii	0.0151	0.0151
g__Bacteroides	s__Bacteroides_nordii	0.0151	0.0151
g__Bacteroides	s__Bacteroides_nordii	0.0151	0.0151
g__Bacteroides	s__Bacteroides_nordii	0.0151	0.0151
g__Bacteroides	s__Bacteroides_ovatus	0.0151	0.0151
g__Bacteroides	s__Bacteroides_ovatus	0.0151	0.0151
g__Bacteroides	s__Bacteroides_ovatus	0.0151	0.0151
g__Bacteroides	s__Bacteroides_ovatus	0.0151	0.0151
g__Bacteroides	s__Bacteroides_ovatus	0.0151	0.0151
g__Bacteroides	s__Bacteroides_ovatus	0.0151	0.0151

g__Bacteroides	s__Bacteroides_ovatus	0.0151	0.0151
g__Bacteroides	s__Bacteroides_ovatus	0.0151	0.0151
g__Bacteroides	s__Bacteroides_ovatus	0.0151	0.0151
g__Bacteroides	s__Bacteroides_ovatus	0.0151	0.0151
g__Bacteroides	s__Bacteroides_ovatus	0.0151	0.0151
g__Bacteroides	s__Bacteroides_ovatus	0.0151	0.0151
g__Bacteroides	s__Bacteroides_ovatus	0.0151	0.0151
g__Bacteroides	s__Bacteroides_ovatus	0.0151	0.0151
g__Bacteroides	s__Bacteroides_ovatus	0.0151	0.0151
g__Bacteroides	s__Bacteroides_ovatus	0.0151	0.0151
g__Bacteroides	s__Bacteroides_ovatus	0.0151	0.0151
g__Bacteroides	s__Bacteroides_ovatus	0.0151	0.0151
g__Bacteroides	s__Bacteroides_ovatus	0.0151	0.0151
g__Bacteroides	s__Bacteroides_ovatus	0.0151	0.0151
g__Bacteroides	s__Bacteroides_ovatus	0.0151	0.0151
g__Bacteroides	s__Bacteroides_ovatus	0.0151	0.0151
g__Bacteroides	s__Bacteroides_ovatus	0.0151	0.0151
g__Bacteroides	s__Bacteroides_ovatus	0.0151	0.0151
g__Bacteroides	s__Bacteroides_ovatus	0.0151	0.0151
g__Bacteroides	s__Bacteroides_ovatus	0.0151	0.0151
g__Bacteroides	s__Bacteroides_ovatus	0.0151	0.0151
g__Bacteroides	s__Bacteroides_ovatus	0.0151	0.0151
g__Bacteroides	s__Bacteroides_ovatus	0.0151	0.0151
g__Bacteroides	s__Bacteroides_ovatus	0.0151	0.0151
g__Bacteroides	s__Bacteroides_ovatus	0.0151	0.0151
g__Bacteroides	s__Bacteroides_ovatus	0.0151	0.0151
g__Bacteroides	s__Bacteroides_ovatus	0.0151	0.0151
g__Bacteroides	s__Bacteroides_ovatus	0.0151	0.0151
g__Bacteroides	s__Bacteroides_ovatus	0.0151	0.0151
g__Bacteroides	s__Bacteroides_ovatus	0.0151	0.0151
g__Bacteroides	s__Bacteroides_ovatus	0.0151	0.0151
g__Bacteroides	s__Bacteroides_ovatus	0.0151	0.0151
g__Bacteroides	s__Bacteroides_ovatus	0.0151	0.0151
g__Bacteroides	s__Bacteroides_ovatus	0.0151	0.0151
g__Bacteroides	s__Bacteroides_ovatus	0.0151	0.0151
g__Bacteroides	s__Bacteroides_ovatus	0.0151	0.0151
g__Bacteroides	s__Bacteroides_ovatus	0.0151	0.0151
g__Bacteroides	s__Bacteroides_ovatus	0.0151	0.0151
g__Bacteroides	s__Bacteroides_ovatus	0.0151	0.0151
g__Bacteroides	s__Bacteroides_ovatus	0.0151	0.0151
g__Bacteroides	s__Bacteroides_ovatus	0.0151	0.0151
g__Bacteroides	s__Bacteroides_ovatus	0.0151	0.0151
g__Bacteroides	s__Bacteroides_ovatus	0.0151	0.0151

g__Bacteroides	s__Bacteroides_ovatus	0.0151	0.0151
g__Bacteroides	s__Bacteroides_ovatus	0.0151	0.0151
g__Bacteroides	s__Bacteroides_ovatus	0.0151	0.0151
g__Bacteroides	s__Bacteroides_ovatus	0.0151	0.0151
g__Bacteroides	s__Bacteroides_ovatus	0.0151	0.0151
g__Bacteroides	s__Bacteroides_ovatus	0.0151	0.0151
g__Bacteroides	s__Bacteroides_ovatus	0.0151	0.0151
g__Bacteroides	s__Bacteroides_ovatus	0.0151	0.0151
g__Bacteroides	s__Bacteroides_ovatus	0.0151	0.0151
g__Bacteroides	s__Bacteroides_ovatus	0.0151	0.0151
g__Bacteroides	s__Bacteroides_ovatus	0.0151	0.0151
g__Bacteroides	s__Bacteroides_ovatus	0.0151	0.0151
g__Bacteroides	s__Bacteroides_ovatus	0.0151	0.0151
g__Bacteroides	s__Bacteroides_ovatus	0.0151	0.0151
g__Bacteroides	s__Bacteroides_ovatus	0.0151	0.0151
g__Bacteroides	s__Bacteroides_ovatus	0.0151	0.0151
g__Bacteroides	s__Bacteroides_ovatus	0.0151	0.0151
g__Bacteroides	s__Bacteroides_ovatus	0.0151	0.0151
g__Bacteroides	s__Bacteroides_ovatus	0.0151	0.0151
g__Bacteroides	s__Bacteroides_ovatus	0.0151	0.0151
g__Bacteroides	s__Bacteroides_ovatus	0.0151	0.0151
g__Bacteroides	s__Bacteroides_ovatus	0.0151	0.0151
g__Bacteroides	s__Bacteroides_ovatus	0.0151	0.0151
g__Bacteroides	s__Bacteroides_ovatus	0.0151	0.0151
g__Bacteroides	s__Bacteroides_ovatus	0.0151	0.0151
g__Bacteroides	s__Bacteroides_ovatus	0.0151	0.0151
g__Bacteroides	s__Bacteroides_ovatus	0.0151	0.0151
g__Bacteroides	s__Bacteroides_ovatus	0.0151	0.0151
g__Bacteroides	s__Bacteroides_ovatus	0.0151	0.0151
g__Bacteroides	s__Bacteroides_ovatus	0.0151	0.0151
g__Bacteroides	s__Bacteroides_ovatus	0.0151	0.0151
g__Bacteroides	s__Bacteroides_ovatus	0.0151	0.0151
g__Bacteroides	s__Bacteroides_ovatus	0.0151	0.0151
g__Bacteroides	s__Bacteroides_ovatus	0.0151	0.0151
g__Bacteroides	s__Bacteroides_ovatus	0.0151	0.0151
g__Bacteroides	s__Bacteroides_ovatus	0.0151	0.0151
g__Bacteroides	s__Bacteroides_ovatus	0.0151	0.0151
g__Bacteroides	s__Bacteroides_ovatus	0.0151	0.0151
g__Bacteroides	s__Bacteroides_ovatus	0.0151	0.0151
g__Bacteroides	s__Bacteroides_ovatus	0.0151	0.0151
g__Bacteroides	s__Bacteroides_ovatus	0.0151	0.0151
g__Bacteroides	s__Bacteroides_ovatus	0.0151	0.0151
g__Bacteroides	s__Bacteroides_ovatus	0.0151	0.0151

g__Bacteroides	s__Bacteroides_ovatus	0.0151	0.0151
g__Bacteroides	s__Bacteroides_ovatus	0.0151	0.0151
g__Bacteroides	s__Bacteroides_ovatus	0.0151	0.0151
g__Bacteroides	s__Bacteroides_ovatus	0.0151	0.0151
g__Bacteroides	s__Bacteroides_ovatus	0.0151	0.0151
g__Bacteroides	s__Bacteroides_ovatus	0.0151	0.0151
g__Bacteroides	s__Bacteroides_ovatus	0.0151	0.0151
g__Bacteroides	s__Bacteroides_ovatus	0.0151	0.0151
g__Bacteroides	s__Bacteroides_ovatus	0.0151	0.0151
g__Bacteroides	s__Bacteroides_ovatus	0.0151	0.0151
g__Bacteroides	s__Bacteroides_ovatus	0.0151	0.0151
g__Bacteroides	s__Bacteroides_ovatus	0.0151	0.0151
g__Bacteroides	s__Bacteroides_ovatus	0.0151	0.0151
g__Bacteroides	s__Bacteroides_ovatus	0.0151	0.0151
g__Bacteroides	s__Bacteroides_ovatus	0.0151	0.0151
g__Bacteroides	s__Bacteroides_ovatus	0.0151	0.0151
g__Bacteroides	s__Bacteroides_ovatus	0.0151	0.0151
g__Bacteroides	s__Bacteroides_ovatus	0.0151	0.0151
g__Bacteroides	s__Bacteroides_ovatus	0.0151	0.0151
g__Bacteroides	s__Bacteroides_ovatus	0.0151	0.0151
g__Bacteroides	s__Bacteroides_ovatus	0.0151	0.0151
g__Bacteroides	s__Bacteroides_ovatus	0.0151	0.0151
g__Bacteroides	s__Bacteroides_ovatus	0.0151	0.0151
g__Bacteroides	s__Bacteroides_ovatus	0.0151	0.0151
g__Bacteroides	s__Bacteroides_ovatus	0.0151	0.0151
g__Bacteroides	s__Bacteroides_ovatus	0.0151	0.0151
g__Bacteroides	s__Bacteroides_ovatus	0.0151	0.0151
g__Bacteroides	s__Bacteroides_ovatus	0.0151	0.0151
g__Bacteroides	s__Bacteroides_ovatus	0.0151	0.0151
g__Bacteroides	s__Bacteroides_ovatus	0.0151	0.0151
g__Bacteroides	s__Bacteroides_ovatus	0.0151	0.0151
g__Bacteroides	s__Bacteroides_ovatus	0.0151	0.0151
g__Bacteroides	s__Bacteroides_ovatus	0.0151	0.0151
g__Bacteroides	s__Bacteroides_ovatus	0.0151	0.0151
g__Bacteroides	s__Bacteroides_ovatus	0.0151	0.0151
g__Bacteroides	s__Bacteroides_ovatus	0.0151	0.0151
g__Bacteroides	s__Bacteroides_ovatus	0.0151	0.0151
g__Bacteroides	s__Bacteroides_ovatus	0.0151	0.0151
g__Bacteroides	s__Bacteroides_ovatus	0.0151	0.0151
g__Bacteroides	s__Bacteroides_ovatus	0.0151	0.0151
g__Bacteroides	s__Bacteroides_ovatus	0.0151	0.0151
g__Bacteroides	s__Bacteroides_ovatus	0.0151	0.0151
g__Bacteroides	s__Bacteroides_ovatus	0.0151	0.0151

g__Bacteroides	s__Bacteroides_ovatus	0.0151	0.0151
g__Bacteroides	s__Bacteroides_ovatus	0.0151	0.0151
g__Bacteroides	s__Bacteroides_ovatus	0.0151	0.0151
g__Bacteroides	s__Bacteroides_ovatus	0.0151	0.0151
g__Bacteroides	s__Bacteroides_ovatus	0.0151	0.0151
g__Bacteroides	s__Bacteroides_ovatus	0.0151	0.0151
g__Bacteroides	s__Bacteroides_ovatus	0.0151	0.0151
g__Bacteroides	s__Bacteroides_ovatus	0.0151	0.0151
g__Bacteroides	s__Bacteroides_ovatus	0.0151	0.0151
g__Bacteroides	s__Bacteroides_ovatus	0.0151	0.0151
g__Bacteroides	s__Bacteroides_ovatus	0.0151	0.0151
g__Bacteroides	s__Bacteroides_ovatus	0.0151	0.0151
g__Bacteroides	s__Bacteroides_ovatus	0.0151	0.0151
g__Bacteroides	s__Bacteroides_ovatus	0.0151	0.0151
g__Bacteroides	s__Bacteroides_ovatus	0.0151	0.0151
g__Bacteroides	s__Bacteroides_ovatus	0.0151	0.0151
g__Bacteroides	s__Bacteroides_ovatus	0.0151	0.0151
g__Bacteroides	s__Bacteroides_ovatus	0.0151	0.0151
g__Bacteroides	s__Bacteroides_ovatus	0.0151	0.0151
g__Bacteroides	s__Bacteroides_ovatus	0.0151	0.0151
g__Bacteroides	s__Bacteroides_ovatus	0.0151	0.0151
g__Bacteroides	s__Bacteroides_ovatus	0.0151	0.0151
g__Bacteroides	s__Bacteroides_ovatus	0.0151	0.0151
g__Bacteroides	s__Bacteroides_ovatus	0.0151	0.0151
g__Bacteroides	s__Bacteroides_ovatus	0.0151	0.0151
g__Bacteroides	s__Bacteroides_ovatus	0.0151	0.0151
g__Bacteroides	s__Bacteroides_ovatus	0.0151	0.0151
g__Bacteroides	s__Bacteroides_ovatus	0.0151	0.0151
g__Bacteroides	s__Bacteroides_ovatus	0.0151	0.0151
g__Bacteroides	s__Bacteroides_ovatus	0.0151	0.0151
g__Bacteroides	s__Bacteroides_ovatus	0.0151	0.0151
g__Bacteroides	s__Bacteroides_ovatus	0.0151	0.0151
g__Bacteroides	s__Bacteroides_ovatus	0.0151	0.0151
g__Bacteroides	s__Bacteroides_ovatus	0.0151	0.0151
g__Bacteroides	s__Bacteroides_ovatus	0.0151	0.0151
g__Bacteroides	s__Bacteroides_ovatus	0.0151	0.0151
g__Bacteroides	s__Bacteroides_ovatus	0.0151	0.0151
g__Bacteroides	s__Bacteroides_ovatus	0.0151	0.0151
g__Bacteroides	s__Bacteroides_ovatus	0.0151	0.0151
g__Bacteroides	s__Bacteroides_ovatus	0.0151	0.0151
g__Bacteroides	s__Bacteroides_ovatus	0.0151	0.0151
g__Bacteroides	s__Bacteroides_ovatus	0.0151	0.0151
g__Bacteroides	s__Bacteroides_ovatus	0.0151	0.0151

g__Bacteroides	s__Bacteroides_ovatus	0.0151	0.0151
g__Bacteroides	s__Bacteroides_ovatus	0.0151	0.0151
g__Bacteroides	s__Bacteroides_ovatus	0.0151	0.0151
g__Bacteroides	s__Bacteroides_ovatus	0.0151	0.0151
g__Bacteroides	s__Bacteroides_ovatus	0.0151	0.0151
g__Bacteroides	s__Bacteroides_ovatus	0.0151	0.0151
g__Bacteroides	s__Bacteroides_ovatus	0.0151	0.0151
g__Bacteroides	s__Bacteroides_ovatus	0.0151	0.0151
g__Bacteroides	s__Bacteroides_ovatus	0.0151	0.0151
g__Bacteroides	s__Bacteroides_ovatus	0.0151	0.0151
g__Bacteroides	s__Bacteroides_ovatus	0.0151	0.0151
g__Bacteroides	s__Bacteroides_ovatus	0.0151	0.0151
g__Bacteroides	s__Bacteroides_ovatus	0.0151	0.0151
g__Bacteroides	s__Bacteroides_ovatus	0.0151	0.0151
g__Bacteroides	s__Bacteroides_ovatus	0.0151	0.0151
g__Bacteroides	s__Bacteroides_ovatus	0.0151	0.0151
g__Bacteroides	s__Bacteroides_ovatus	0.0151	0.0151
g__Bacteroides	s__Bacteroides_ovatus	0.0151	0.0151
g__Bacteroides	s__Bacteroides_ovatus	0.0151	0.0151
g__Bacteroides	s__Bacteroides_ovatus	0.0151	0.0151
g__Bacteroides	s__Bacteroides_ovatus	0.0151	0.0151
g__Bacteroides	s__Bacteroides_ovatus	0.0151	0.0151
g__Bacteroides	s__Bacteroides_ovatus	0.0151	0.0151
g__Bacteroides	s__Bacteroides_ovatus	0.0151	0.0151
g__Bacteroides	s__Bacteroides_ovatus	0.0151	0.0151
g__Bacteroides	s__Bacteroides_ovatus	0.0151	0.0151
g__Bacteroides	s__Bacteroides_ovatus	0.0151	0.0151
g__Bacteroides	s__Bacteroides_ovatus	0.0151	0.0151
g__Bacteroides	s__Bacteroides_ovatus	0.0151	0.0151
g__Bacteroides	s__Bacteroides_ovatus	0.0151	0.0151
g__Bacteroides	s__Bacteroides_ovatus	0.0151	0.0151
g__Bacteroides	s__Bacteroides_ovatus	0.0151	0.0151
g__Bacteroides	s__Bacteroides_ovatus	0.0151	0.0151
g__Bacteroides	s__Bacteroides_ovatus	0.0151	0.0151
g__Bacteroides	s__Bacteroides_ovatus	0.0151	0.0151
g__Bacteroides	s__Bacteroides_ovatus	0.0151	0.0151
g__Bacteroides	s__Bacteroides_ovatus	0.0151	0.0151
g__Bacteroides	s__Bacteroides_ovatus	0.0151	0.0151
g__Bacteroides	s__Bacteroides_ovatus	0.0151	0.0151
g__Bacteroides	s__Bacteroides_ovatus	0.0151	0.0151
g__Bacteroides	s__Bacteroides_ovatus	0.0151	0.0151
g__Bacteroides	s__Bacteroides_ovatus	0.0151	0.0151
g__Bacteroides	s__Bacteroides_ovatus	0.0151	0.0151

g__Bacteroides	s__Bacteroides_ovatus	0.0151	0.0151
g__Bacteroides	s__Bacteroides_ovatus	0.0151	0.0151
g__Bacteroides	s__Bacteroides_ovatus	0.0151	0.0151
g__Bacteroides	s__Bacteroides_ovatus	0.0151	0.0151
g__Bacteroides	s__Bacteroides_salyersiae	0.0151	0.0151
g__Bacteroides	s__Bacteroides_salyersiae	0.0151	0.0151
g__Bacteroides	s__Bacteroides_salyersiae	0.0151	0.0151
g__Bacteroides	s__Bacteroides_salyersiae	0.0151	0.0151
g__Bacteroides	s__Bacteroides_salyersiae	0.0151	0.0151
g__Bacteroides	s__Bacteroides_salyersiae	0.0151	0.0151
g__Bacteroides	s__Bacteroides_salyersiae	0.0151	0.0151
g__Bacteroides	s__Bacteroides_salyersiae	0.0151	0.0151
g__Bacteroides	s__Bacteroides_salyersiae	0.0151	0.0151
g__Bacteroides	s__Bacteroides_stercoris	0.0151	0.0151
g__Bacteroides	s__Bacteroides_stercoris	0.0151	0.0151
g__Bacteroides	s__Bacteroides_stercoris	0.0151	0.0151
g__Bacteroides	s__Bacteroides_stercoris	0.0151	0.0151
g__Bacteroides	s__Bacteroides_stercoris	0.0151	0.0151
g__Bacteroides	s__Bacteroides_stercoris	0.0151	0.0151
g__Bacteroides	s__Bacteroides_stercoris	0.0151	0.0151
g__Bacteroides	s__Bacteroides_stercoris	0.0151	0.0151
g__Bacteroides	s__Bacteroides_stercoris	0.0151	0.0151
g__Bacteroides	s__Bacteroides_stercoris	0.0151	0.0151
g__Bacteroides	s__Bacteroides_stercoris	0.0151	0.0151
g__Bacteroides	s__Bacteroides_stercoris	0.0151	0.0151
g__Bacteroides	s__Bacteroides_stercoris	0.0151	0.0151
g__Bacteroides	s__Bacteroides_stercoris	0.0151	0.0151
g__Bacteroides	s__Bacteroides_stercoris	0.0151	0.0151
g__Bacteroides	s__Bacteroides_stercoris	0.0151	0.0151
g__Bacteroides	s__Bacteroides_thetaiotaomicron	0.0151	0.0151
g__Bacteroides	s__Bacteroides_thetaiotaomicron	0.0151	0.0151
g__Bacteroides	s__Bacteroides_thetaiotaomicron	0.0151	0.0151
g__Bacteroides	s__Bacteroides_thetaiotaomicron	0.0151	0.0151
g__Bacteroides	s__Bacteroides_thetaiotaomicron	0.0151	0.0151
g__Bacteroides	s__Bacteroides_thetaiotaomicron	0.0151	0.0151
g__Bacteroides	s__Bacteroides_thetaiotaomicron	0.0151	0.0151
g__Bacteroides	s__Bacteroides_thetaiotaomicron	0.0151	0.0151
g__Bacteroides	s__Bacteroides_thetaiotaomicron	0.0151	0.0151
g__Bacteroides	s__Bacteroides_thetaiotaomicron	0.0151	0.0151
g__Bacteroides	s__Bacteroides_thetaiotaomicron	0.0151	0.0151
g__Bacteroides	s__Bacteroides_thetaiotaomicron	0.0151	0.0151
g__Bacteroides	s__Bacteroides_thetaiotaomicron	0.0151	0.0151
g__Bacteroides	s__Bacteroides_thetaiotaomicron	0.0151	0.0151

g__Bacteroides	s__Bacteroides_thetaiotaomicron	0.0151	0.0151
g__Bacteroides	s__Bacteroides_thetaiotaomicron	0.0151	0.0151
g__Bacteroides	s__Bacteroides_thetaiotaomicron	0.0151	0.0151
g__Bacteroides	s__Bacteroides_thetaiotaomicron	0.0151	0.0151
g__Bacteroides	s__Bacteroides_thetaiotaomicron	0.0151	0.0151
g__Bacteroides	s__Bacteroides_thetaiotaomicron	0.0151	0.0151
g__Bacteroides	s__Bacteroides_thetaiotaomicron	0.0151	0.0151
g__Bacteroides	s__Bacteroides_thetaiotaomicron	0.0151	0.0151
g__Bacteroides	s__Bacteroides_thetaiotaomicron	0.0151	0.0151
g__Bacteroides	s__Bacteroides_thetaiotaomicron	0.0151	0.0151
g__Bacteroides	s__Bacteroides_thetaiotaomicron	0.0151	0.0151
g__Bacteroides	s__Bacteroides_thetaiotaomicron	0.0151	0.0151
g__Bacteroides	s__Bacteroides_thetaiotaomicron	0.0151	0.0151
g__Bacteroides	s__Bacteroides_thetaiotaomicron	0.0151	0.0151
g__Bacteroides	s__Bacteroides_thetaiotaomicron	0.0151	0.0151
g__Bacteroides	s__Bacteroides_thetaiotaomicron	0.0151	0.0151
g__Bacteroides	s__Bacteroides_thetaiotaomicron	0.0151	0.0151
g__Bacteroides	s__Bacteroides_thetaiotaomicron	0.0151	0.0151
g__Bacteroides	s__Bacteroides_thetaiotaomicron	0.0151	0.0151
g__Bacteroides	s__Bacteroides_thetaiotaomicron	0.0151	0.0151
g__Bacteroides	s__Bacteroides_thetaiotaomicron	0.0151	0.0151
g__Bacteroides	s__Bacteroides_thetaiotaomicron	0.0151	0.0151
g__Bacteroides	s__Bacteroides_thetaiotaomicron	0.0151	0.0151
g__Bacteroides	s__Bacteroides_thetaiotaomicron	0.0151	0.0151
g__Bacteroides	s__Bacteroides_thetaiotaomicron	0.0151	0.0151
g__Bacteroides	s__Bacteroides_thetaiotaomicron	0.0151	0.0151
g__Bacteroides	s__Bacteroides_thetaiotaomicron	0.0151	0.0151
g__Bacteroides	s__Bacteroides_thetaiotaomicron	0.0151	0.0151
g__Bacteroides	s__Bacteroides_thetaiotaomicron	0.0151	0.0151
g__Bacteroides	s__Bacteroides_thetaiotaomicron	0.0151	0.0151
g__Bacteroides	s__Bacteroides_thetaiotaomicron	0.0151	0.0151
g__Bacteroides	s__Bacteroides_thetaiotaomicron	0.0151	0.0151
g__Bacteroides	s__Bacteroides_thetaiotaomicron	0.0151	0.0151
g__Bacteroides	s__Bacteroides_thetaiotaomicron	0.0151	0.0151
g__Bacteroides	s__Bacteroides_thetaiotaomicron	0.0151	0.0151
g__Bacteroides	s__Bacteroides_thetaiotaomicron	0.0151	0.0151
g__Bacteroides	s__Bacteroides_thetaiotaomicron	0.0151	0.0151
g__Bacteroides	s__Bacteroides_thetaiotaomicron	0.0151	0.0151
g__Bacteroides	s__Bacteroides_thetaiotaomicron	0.0151	0.0151
g__Bacteroides	s__Bacteroides_thetaiotaomicron	0.0151	0.0151
g__Bacteroides	s__Bacteroides_thetaiotaomicron	0.0151	0.0151
g__Bacteroides	s__Bacteroides_thetaiotaomicron	0.0151	0.0151
g__Bacteroides	s__Bacteroides_thetaiotaomicron	0.0151	0.0151

g__Bacteroides	s__Bacteroides_thetaiotaomicron	0.0151	0.0151
g__Bacteroides	s__Bacteroides_thetaiotaomicron	0.0151	0.0151
g__Bacteroides	s__Bacteroides_thetaiotaomicron	0.0151	0.0151
g__Bacteroides	s__Bacteroides_thetaiotaomicron	0.0151	0.0151
g__Bacteroides	s__Bacteroides_thetaiotaomicron	0.0151	0.0151
g__Bacteroides	s__Bacteroides_thetaiotaomicron	0.0151	0.0151
g__Bacteroides	s__Bacteroides_thetaiotaomicron	0.0151	0.0151
g__Bacteroides	s__Bacteroides_thetaiotaomicron	0.0151	0.0151
g__Bacteroides	s__Bacteroides_thetaiotaomicron	0.0151	0.0151
g__Bacteroides	s__Bacteroides_thetaiotaomicron	0.0151	0.0151
g__Bacteroides	s__Bacteroides_thetaiotaomicron	0.0151	0.0151
g__Bacteroides	s__Bacteroides_thetaiotaomicron	0.0151	0.0151
g__Bacteroides	s__Bacteroides_thetaiotaomicron	0.0151	0.0151
g__Bacteroides	s__Bacteroides_thetaiotaomicron	0.0151	0.0151
g__Bacteroides	s__Bacteroides_thetaiotaomicron	0.0151	0.0151
g__Bacteroides	s__Bacteroides_thetaiotaomicron	0.0151	0.0151
g__Bacteroides	s__Bacteroides_thetaiotaomicron	0.0151	0.0151
g__Bacteroides	s__Bacteroides_thetaiotaomicron	0.0151	0.0151
g__Bacteroides	s__Bacteroides_thetaiotaomicron	0.0151	0.0151
g__Bacteroides	s__Bacteroides_thetaiotaomicron	0.0151	0.0151
g__Bacteroides	s__Bacteroides_thetaiotaomicron	0.0151	0.0151
g__Bacteroides	s__Bacteroides_thetaiotaomicron	0.0151	0.0151
g__Bacteroides	s__Bacteroides_thetaiotaomicron	0.0151	0.0151
g__Bacteroides	s__Bacteroides_thetaiotaomicron	0.0151	0.0151
g__Bacteroides	s__Bacteroides_thetaiotaomicron	0.0151	0.0151
g__Bacteroides	s__Bacteroides_thetaiotaomicron	0.0151	0.0151
g__Bacteroides	s__Bacteroides_thetaiotaomicron	0.0151	0.0151
g__Bacteroides	s__Bacteroides_thetaiotaomicron	0.0151	0.0151
g__Bacteroides	s__Bacteroides_thetaiotaomicron	0.0151	0.0151
g__Bacteroides	s__Bacteroides_thetaiotaomicron	0.0151	0.0151
g__Bacteroides	s__Bacteroides_thetaiotaomicron	0.0151	0.0151
g__Bacteroides	s__Bacteroides_thetaiotaomicron	0.0151	0.0151
g__Bacteroides	s__Bacteroides_thetaiotaomicron	0.0151	0.0151
g__Bacteroides	s__Bacteroides_thetaiotaomicron	0.0151	0.0151
g__Bacteroides	s__Bacteroides_thetaiotaomicron	0.0151	0.0151
g__Bacteroides	s__Bacteroides_uniformis	0.0151	0.0151
g__Bacteroides	s__Bacteroides_uniformis	0.0151	0.0151
g__Bacteroides	s__Bacteroides_uniformis	0.0151	0.0151
g__Bacteroides	s__Bacteroides_uniformis	0.0151	0.0151
g__Bacteroides	s__Bacteroides_uniformis	0.0151	0.0151
g__Bacteroides	s__Bacteroides_uniformis	0.0151	0.0151
g__Bacteroides	s__Bacteroides_uniformis	0.0151	0.0151
g__Bacteroides	s__Bacteroides_uniformis	0.0151	0.0151

g__Bacteroides	s__Bacteroides_uniformis	0.0151	0.0151
g__Bacteroides	s__Bacteroides_uniformis	0.0151	0.0151
g__Bacteroides	s__Bacteroides_uniformis	0.0151	0.0151
g__Bacteroides	s__Bacteroides_uniformis	0.0151	0.0151
g__Bacteroides	s__Bacteroides_uniformis	0.0151	0.0151
g__Bacteroides	s__Bacteroides_uniformis	0.0151	0.0151
g__Bacteroides	s__Bacteroides_uniformis	0.0151	0.0151
g__Bacteroides	s__Bacteroides_uniformis	0.0151	0.0151
g__Bacteroides	s__Bacteroides_uniformis	0.0151	0.0151
g__Bacteroides	s__Bacteroides_uniformis	0.0151	0.0151
g__Bacteroides	s__Bacteroides_uniformis	0.0151	0.0151
g__Bacteroides	s__Bacteroides_uniformis	0.0151	0.0151
g__Bacteroides	s__Bacteroides_uniformis	0.0151	0.0151
g__Bacteroides	s__Bacteroides_uniformis	0.0151	0.0151
g__Bacteroides	s__Bacteroides_uniformis	0.0151	0.0151
g__Bacteroides	s__Bacteroides_uniformis	0.0151	0.0151
g__Bacteroides	s__Bacteroides_uniformis	0.0151	0.0151
g__Bacteroides	s__Bacteroides_uniformis	0.0151	0.0151
g__Bacteroides	s__Bacteroides_uniformis	0.0151	0.0151
g__Bacteroides	s__Bacteroides_uniformis	0.0151	0.0151
g__Bacteroides	s__Bacteroides_uniformis	0.0151	0.0151
g__Bacteroides	s__Bacteroides_uniformis	0.0151	0.0151
g__Bacteroides	s__Bacteroides_uniformis	0.0151	0.0151
g__Bacteroides	s__Bacteroides_uniformis	0.0151	0.0151
g__Bacteroides	s__Bacteroides_uniformis	0.0151	0.0151
g__Bacteroides	s__Bacteroides_uniformis	0.0151	0.0151
g__Bacteroides	s__Bacteroides_uniformis	0.0151	0.0151
g__Bacteroides	s__Bacteroides_uniformis	0.0151	0.0151
g__Bacteroides	s__Bacteroides_uniformis	0.0151	0.0151
g__Bacteroides	s__Bacteroides_uniformis	0.0151	0.0151
g__Bacteroides	s__Bacteroides_uniformis	0.0151	0.0151
g__Bacteroides	s__Bacteroides_uniformis	0.0151	0.0151
g__Bacteroides	s__Bacteroides_uniformis	0.0151	0.0151
g__Bacteroides	s__Bacteroides_uniformis	0.0151	0.0151
g__Bacteroides	s__Bacteroides_uniformis	0.0151	0.0151
g__Bacteroides	s__Bacteroides_uniformis	0.0151	0.0151
g__Bacteroides	s__Bacteroides_uniformis	0.0151	0.0151
g__Bacteroides	s__Bacteroides_uniformis	0.0151	0.0151
g__Bacteroides	s__Bacteroides_uniformis	0.0151	0.0151
g__Bacteroides	s__Bacteroides_uniformis	0.0151	0.0151
g__Bacteroides	s__Bacteroides_uniformis	0.0151	0.0151
g__Bacteroides	s__Bacteroides_xylanisolvens	0.0151	0.0151
g__Bacteroides	s__Bacteroides_xylanisolvens	0.0151	0.0151

g__Bacteroides	s__Bacteroides_xylanisolvens	0.0151	0.0151
g__Bacteroides	s__Bacteroides_xylanisolvens	0.0151	0.0151
g__Bacteroides	s__Bacteroides_xylanisolvens	0.0151	0.0151
g__Bacteroides	s__Bacteroides_xylanisolvens	0.0151	0.0151
g__Bacteroides	s__Bacteroides_xylanisolvens	0.0151	0.0151
g__Bacteroides	s__Bacteroides_xylanisolvens	0.0151	0.0151
g__Bacteroides	s__Bacteroides_xylanisolvens	0.0151	0.0151
g__Bacteroides	s__Bacteroides_xylanisolvens	0.0151	0.0151
g__Bacteroides	s__Bacteroides_xylanisolvens	0.0151	0.0151
g__Bacteroides	s__Bacteroides_xylanisolvens	0.0151	0.0151
g__Bacteroides	s__Bacteroides_xylanisolvens	0.0151	0.0151
g__Bacteroides	s__Bacteroides_xylanisolvens	0.0151	0.0151
g__Bacteroides	s__Bacteroides_xylanisolvens	0.0151	0.0151
g__Bacteroides	s__Bacteroides_xylanisolvens	0.0151	0.0151
g__Bacteroides	s__Bacteroides_xylanisolvens	0.0151	0.0151
g__Bacteroides	s__Bacteroides_xylanisolvens	0.0151	0.0151
g__Bacteroides	s__Bacteroides_xylanisolvens	0.0151	0.0151
g__Bacteroides	s__Bacteroides_xylanisolvens	0.0151	0.0151
g__Bacteroides	s__Bacteroides_xylanisolvens	0.0151	0.0151
g__Bacteroides	s__Bacteroides_xylanisolvens	0.0151	0.0151
g__Bacteroides	s__Bacteroides_xylanisolvens	0.0151	0.0151
g__Bacteroides	s__Bacteroides_xylanisolvens	0.0151	0.0151
g__Bacteroides	s__Bacteroides_xylanisolvens	0.0151	0.0151
g__Bacteroides	s__Bacteroides_xylanisolvens	0.0151	0.0151
g__Bacteroides	s__Bacteroides_xylanisolvens	0.0151	0.0151
g__Bacteroides	s__Bacteroides_xylanisolvens	0.0151	0.0151
g__Bacteroides	s__Bacteroides_xylanisolvens	0.0151	0.0151
g__Bacteroides	s__Bacteroides_xylanisolvens	0.0151	0.0151
g__Bacteroides	s__Bacteroides_xylanisolvens	0.0151	0.0151
g__Bacteroides	s__Bacteroides_xylanisolvens	0.0151	0.0151
g__Bacteroides	s__Bacteroides_xylanisolvens	0.0151	0.0151
g__Bacteroides	s__Bacteroides_xylanisolvens	0.0151	0.0151
g__Bacteroides	s__Bacteroides_xylanisolvens	0.0151	0.0151
g__Bacteroides	s__Bacteroides_xylanisolvens	0.0151	0.0151
g__Bacteroides	s__Bacteroides_xylanisolvens	0.0151	0.0151
g__Bacteroides	s__Bacteroides_xylanisolvens	0.0151	0.0151
g__Bacteroides	s__Bacteroides_xylanisolvens	0.0151	0.0151
g__Bacteroides	s__Bacteroides_xylanisolvens	0.0151	0.0151
g__Bacteroides	s__Bacteroides_xylanisolvens	0.0151	0.0151
g__Bacteroides	s__Bacteroides_xylanisolvens	0.0151	0.0151
g__Bacteroides	s__Bacteroides_xylanisolvens	0.0151	0.0151
g__Bacteroides	s__Bacteroides_xylanisolvens	0.0151	0.0151
g__Bacteroides	s__Bacteroides_xylanisolvens	0.0151	0.0151

g__Bacteroides	s__Bacteroides_xylanisolvens	0.0151	0.0151
g__Bacteroides	s__Bacteroides_xylanisolvens	0.0151	0.0151
g__Bacteroides	s__Bacteroides_xylanisolvens	0.0151	0.0151
g__Bacteroides	s__Bacteroides_xylanisolvens	0.0151	0.0151
g__Bacteroides	s__Bacteroides_xylanisolvens	0.0151	0.0151
g__Bacteroides	s__Bacteroides_xylanisolvens	0.0151	0.0151
g__Bacteroides	s__Bacteroides_xylanisolvens	0.0151	0.0151
g__Bacteroides	s__Bacteroides_xylanisolvens	0.0151	0.0151
g__Bacteroides	s__Bacteroides_xylanisolvens	0.0151	0.0151
g__Bacteroides	s__Bacteroides_xylanisolvens	0.0151	0.0151
g__Bacteroides	s__Bacteroides_xylanisolvens	0.0151	0.0151
g__Bacteroides	s__Bacteroides_xylanisolvens	0.0151	0.0151
g__Bacteroides	s__Bacteroides_xylanisolvens	0.0151	0.0151
g__Bacteroides	s__Bacteroides_xylanisolvens	0.0151	0.0151
g__Bacteroides	s__Bacteroides_xylanisolvens	0.0151	0.0151
g__Bacteroides	s__Bacteroides_xylanisolvens	0.0151	0.0151
g__Bacteroides	s__Bacteroides_xylanisolvens	0.0151	0.0151
g__Bacteroides	s__Bacteroides_xylanisolvens	0.0151	0.0151
g__Bacteroides	s__Bacteroides_xylanisolvens	0.0151	0.0151
g__Bacteroides	s__Bacteroides_xylanisolvens	0.0151	0.0151
g__Bacteroides	s__Bacteroides_xylanisolvens	0.0151	0.0151
g__Bacteroides	s__Bacteroides_xylanisolvens	0.0151	0.0151
g__Bacteroides	s__Bacteroides_xylanisolvens	0.0151	0.0151
g__Bacteroides	s__Bacteroides_xylanisolvens	0.0151	0.0151
g__Bacteroides	s__Bacteroides_xylanisolvens	0.0151	0.0151
g__Bacteroides	s__Bacteroides_xylanisolvens	0.0151	0.0151
g__Bacteroides	s__Bacteroides_xylanisolvens	0.0151	0.0151
g__Bacteroides	s__Bacteroides_xylanisolvens	0.0151	0.0151
g__Bacteroides	s__Bacteroides_xylanisolvens	0.0151	0.0151
g__Bacteroides	s__Bacteroides_xylanisolvens	0.0151	0.0151
g__Bacteroides	s__Bacteroides_xylanisolvens	0.0151	0.0151
g__Bacteroides	s__Bacteroides_xylanisolvens	0.0151	0.0151
g__Bacteroides	s__Bacteroides_xylanisolvens	0.0151	0.0151
g__Bacteroides	s__Bacteroides_xylanisolvens	0.0151	0.0151
g__Bacteroides	s__Bacteroides_xylanisolvens	0.0151	0.0151
g__Bacteroides	s__Bacteroides_xylanisolvens	0.0151	0.0151
g__Bacteroides	s__Bacteroides_xylanisolvens	0.0151	0.0151
g__Bacteroides	s__Bacteroides_xylanisolvens	0.0151	0.0151
g__Bacteroides	s__Bacteroides_xylanisolvens	0.0151	0.0151
g__Bacteroides	s__Bacteroides_xylanisolvens	0.0151	0.0151
g__Bacteroides	s__Bacteroides_xylanisolvens	0.0151	0.0151
g__Bacteroides	s__Bacteroides_xylanisolvens	0.0151	0.0151
g__Bacteroides	s__Bacteroides_xylanisolvens	0.0151	0.0151

g__Bacteroides	s__Bacteroides_xylanisolvens	0.0151	0.0151
g__Bacteroides	s__Bacteroides_xylanisolvens	0.0151	0.0151
g__Bacteroides	s__Bacteroides_xylanisolvens	0.0151	0.0151
g__Bacteroides	s__Bacteroides_xylanisolvens	0.0151	0.0151
g__Bacteroides	s__Bacteroides_xylanisolvens	0.0151	0.0151
g__Bacteroides	s__Bacteroides_xylanisolvens	0.0151	0.0151
g__Bacteroides	s__Bacteroides_xylanisolvens	0.0151	0.0151
g__Bacteroides	s__Bacteroides_xylanisolvens	0.0151	0.0151
g__Bacteroides	s__Bacteroides_xylanisolvens	0.0151	0.0151
g__Bacteroides	s__Bacteroides_xylanisolvens	0.0151	0.0151
g__Bacteroides	s__Bacteroides_xylanisolvens	0.0151	0.0151
g__Bacteroides	s__Bacteroides_xylanisolvens	0.0151	0.0151
g__Bacteroides	s__Bacteroides_xylanisolvens	0.0151	0.0151
g__Bacteroides	s__Bacteroides_xylanisolvens	0.0151	0.0151
g__Bacteroides	s__Bacteroides_xylanisolvens	0.0151	0.0151
g__Bacteroides	s__Bacteroides_xylanisolvens	0.0151	0.0151
g__Bacteroides	s__Bacteroides_xylanisolvens	0.0151	0.0151
g__Bacteroides	s__Bacteroides_xylanisolvens	0.0151	0.0151
g__Bacteroides	s__Bacteroides_xylanisolvens	0.0151	0.0151
g__Bacteroides	s__Bacteroides_xylanisolvens	0.0151	0.0151
g__Bacteroides	s__Bacteroides_xylanisolvens	0.0151	0.0151
g__Bacteroides	s__Bacteroides_xylanisolvens	0.0151	0.0151
g__Bacteroides	s__Bacteroides_xylanisolvens	0.0151	0.0151
g__Bacteroides	s__Bacteroides_xylanisolvens	0.0151	0.0151
g__Bacteroides	s__Bacteroides_xylanisolvens	0.0151	0.0151
g__Bacteroides	s__Bacteroides_xylanisolvens	0.0151	0.0151
g__Bacteroides	s__Bacteroides_xylanisolvens	0.0151	0.0151
g__Bacteroides	s__Bacteroides_xylanisolvens	0.0151	0.0151
g__Bacteroides	s__Bacteroides_xylanisolvens	0.0151	0.0151
g__Bacteroides	s__Bacteroides_xylanisolvens	0.0151	0.0151
g__Bacteroides	s__Bacteroides_xylanisolvens	0.0151	0.0151
g__Bacteroides	s__Bacteroides_xylanisolvens	0.0151	0.0151
g__Bacteroides	s__Bacteroides_xylanisolvens	0.0151	0.0151
g__Bacteroides	s__Bacteroides_xylanisolvens	0.0151	0.0151
g__Bacteroides	s__Bacteroides_xylanisolvens	0.0151	0.0151
g__Bacteroides	s__Bacteroides_xylanisolvens	0.0151	0.0151
g__Bacteroides	s__Bacteroides_xylanisolvens	0.0151	0.0151
g__Bacteroides	s__Bacteroides_xylanisolvens	0.0151	0.0151
g__Bacteroides	s__Bacteroides_xylanisolvens	0.0151	0.0151
g__Bacteroides	s__Bacteroides_xylanisolvens	0.0151	0.0151
g__Bacteroides	s__Bacteroides_xylanisolvens	0.0151	0.0151
g__Bacteroides	s__Bacteroides_xylanisolvens	0.0151	0.0151
g__Bacteroides	s__Bacteroides_xylanisolvens	0.0151	0.0151

g__Bacteroides	s__Bacteroides_xylanisolvens	0.0151	0.0151
g__Bacteroides	s__Bacteroides_xylanisolvens	0.0151	0.0151
g__Bacteroides	s__Bacteroides_xylanisolvens	0.0151	0.0151
g__Bacteroides	s__Bacteroides_xylanisolvens	0.0151	0.0151
g__Bacteroides	s__Bacteroides_xylanisolvens	0.0151	0.0151
g__Bacteroides	s__Bacteroides_xylanisolvens	0.0151	0.0151
g__Bacteroides	s__Bacteroides_xylanisolvens	0.0151	0.0151
g__Bacteroides	s__Bacteroides_xylanisolvens	0.0151	0.0151
g__Bacteroides	s__Bacteroides_xylanisolvens	0.0151	0.0151
g__Bacteroides	s__Bacteroides_xylanisolvens	0.0151	0.0151
g__Bacteroides	s__Bacteroides_xylanisolvens	0.0151	0.0151
g__Bacteroides	s__Bacteroides_xylanisolvens	0.0151	0.0151
g__Bacteroides	s__Bacteroides_xylanisolvens	0.0151	0.0151
g__Bacteroides	s__Bacteroides_xylanisolvens	0.0151	0.0151
g__Bacteroides	s__Bacteroides_xylanisolvens	0.0151	0.0151
g__Bacteroides	s__Bacteroides_xylanisolvens	0.0151	0.0151
g__Bacteroides	s__Bacteroides_xylanisolvens	0.0151	0.0151
g__Bacteroides	s__Bacteroides_xylanisolvens	0.0151	0.0151
g__Bacteroides	s__Bacteroides_xylanisolvens	0.0151	0.0151
g__Bacteroides	s__Bacteroides_xylanisolvens	0.0151	0.0151
g__Bacteroides	s__Bacteroides_xylanisolvens	0.0151	0.0151
g__Bacteroides	s__Bacteroides_xylanisolvens	0.0151	0.0151
g__Bacteroides	s__Bacteroides_xylanisolvens	0.0151	0.0151
g__Bacteroides	s__Bacteroides_xylanisolvens	0.0151	0.0151
g__Bacteroides	s__Bacteroides_xylanisolvens	0.0151	0.0151
g__Bacteroides	s__Bacteroides_xylanisolvens	0.0151	0.0151
g__Bacteroides	s__Bacteroides_xylanisolvens	0.0151	0.0151
g__Bacteroides	s__Bacteroides_xylanisolvens	0.0151	0.0151
g__Bacteroides	s__Bacteroides_xylanisolvens	0.0151	0.0151
g__Bacteroides	s__Bacteroides_xylanisolvens	0.0151	0.0151
g__Bacteroides	s__Bacteroides_xylanisolvens	0.0151	0.0151
g__Bacteroides	s__Bacteroides_xylanisolvens	0.0151	0.0151
g__Bacteroides	s__Bacteroides_xylanisolvens	0.0151	0.0151
g__Bacteroides	s__Bacteroides_xylanisolvens	0.0151	0.0151
g__Bacteroides	s__Bacteroides_xylanisolvens	0.0151	0.0151
g__Bacteroides	s__Bacteroides_xylanisolvens	0.0151	0.0151
g__Bacteroides	s__Bacteroides_xylanisolvens	0.0151	0.0151
g__Bacteroides	s__Bacteroides_xylanisolvens	0.0151	0.0151
g__Bacteroides	s__Bacteroides_xylanisolvens	0.0151	0.0151
g__Bacteroides	s__Bacteroides_xylanisolvens	0.0151	0.0151
g__Bacteroides	s__Bacteroides_xylanisolvens	0.0151	0.0151
g__Bacteroides	s__Bacteroides_xylanisolvens	0.0151	0.0151
g__Bacteroides	s__Bacteroides_xylanisolvens	0.0151	0.0151

g__Bacteroides	s__Bacteroides_xylanisolvens	0.0151	0.0151
g__Bacteroides	s__Bacteroides_xylanisolvens	0.0151	0.0151
g__Bacteroides	s__Bacteroides_xylanisolvens	0.0151	0.0151
g__Bacteroides	s__Bacteroides_xylanisolvens	0.0151	0.0151
g__Bacteroides	s__Bacteroides_xylanisolvens	0.0151	0.0151
g__Bacteroides	s__Bacteroides_xylanisolvens	0.0151	0.0151
g__Bacteroides	s__Bacteroides_xylanisolvens	0.0151	0.0151
g__Bacteroides	s__Bacteroides_xylanisolvens	0.0151	0.0151
g__Bacteroides	s__Bacteroides_xylanisolvens	0.0151	0.0151
g__Bacteroides	s__Bacteroides_xylanisolvens	0.0151	0.0151
g__Bacteroides	s__Bacteroides_xylanisolvens	0.0151	0.0151
g__Bacteroides	s__Bacteroides_xylanisolvens	0.0151	0.0151
g__Bacteroides	s__Bacteroides_xylanisolvens	0.0151	0.0151
g__Bacteroides	s__Bacteroides_xylanisolvens	0.0151	0.0151
g__Bacteroides	s__Bacteroides_xylanisolvens	0.0151	0.0151
g__Bacteroides	s__Bacteroides_xylanisolvens	0.0151	0.0151
g__Bacteroides	s__Bacteroides_xylanisolvens	0.0151	0.0151
g__Bacteroides	s__Bacteroides_xylanisolvens	0.0151	0.0151
g__Bacteroides	s__Bacteroides_xylanisolvens	0.0151	0.0151
g__Bacteroides	s__Bacteroides_xylanisolvens	0.0151	0.0151
g__Bacteroides	s__Bacteroides_xylanisolvens	0.0151	0.0151
g__Bacteroides	s__Bacteroides_xylanisolvens	0.0151	0.0151
g__Bacteroides	s__Bacteroides_xylanisolvens	0.0151	0.0151
g__Bacteroides	s__Bacteroides_xylanisolvens	0.0151	0.0151
g__Bacteroides	s__Bacteroides_xylanisolvens	0.0151	0.0151
g__Bacteroides	s__Bacteroides_xylanisolvens	0.0151	0.0151
g__Bacteroides	s__Bacteroides_xylanisolvens	0.0151	0.0151
g__Bacteroides	s__Bacteroides_xylanisolvens	0.0151	0.0151
g__Bacteroides	s__Bacteroides_xylanisolvens	0.0151	0.0151
g__Bacteroides	s__Bacteroides_xylanisolvens	0.0151	0.0151
g__Bacteroides	s__Bacteroides_xylanisolvens	0.0151	0.0151
g__Bacteroides	s__Bacteroides_xylanisolvens	0.0151	0.0151
g__Bacteroides	s__Bacteroides_xylanisolvens	0.0151	0.0151
g__Bacteroides	s__Bacteroides_xylanisolvens	0.0151	0.0151
g__Bacteroides	s__Bacteroides_xylanisolvens	0.0151	0.0151
g__Bacteroides	s__Bacteroides_xylanisolvens	0.0151	0.0151
g__Bacteroides	s__Bacteroides_xylanisolvens	0.0151	0.0151
g__Bacteroides	s__Bacteroides_xylanisolvens	0.0151	0.0151
g__Bacteroides	s__Bacteroides_xylanisolvens	0.0151	0.0151
g__Bacteroides	s__Bacteroides_xylanisolvens	0.0151	0.0151
g__Bacteroides	s__Bacteroides_xylanisolvens	0.0151	0.0151
g__Bacteroides	s__Bacteroides_xylanisolvens	0.0151	0.0151
g__Bacteroides	s__Bacteroides_xylanisolvens	0.0151	0.0151

g__Bacteroides	s__Bacteroides_xylanisolvens	0.0151	0.0151
g__Bacteroides	s__Bacteroides_xylanisolvens	0.0151	0.0151
g__Bacteroides	s__Bacteroides_xylanisolvens	0.0151	0.0151
g__Bacteroides	s__Bacteroides_xylanisolvens	0.0151	0.0151
g__Bacteroides	s__Bacteroides_xylanisolvens	0.0151	0.0151
g__Bacteroides	s__Bacteroides_xylanisolvens	0.0151	0.0151
g__Bacteroides	s__Bacteroides_xylanisolvens	0.0151	0.0151
g__Bacteroides	s__Bacteroides_xylanisolvens	0.0151	0.0151
g__Bacteroides	s__Bacteroides_xylanisolvens	0.0151	0.0151
g__Bacteroides	s__Bacteroides_xylanisolvens	0.0151	0.0151
g__Bacteroides	s__Bacteroides_xylanisolvens	0.0151	0.0151
g__Bacteroides	s__Bacteroides_xylanisolvens	0.0151	0.0151
g__Bacteroides	s__Bacteroides_xylanisolvens	0.0151	0.0151
g__Bacteroides	s__Bacteroides_xylanisolvens	0.0151	0.0151
g__Bacteroides	s__Bacteroides_xylanisolvens	0.0151	0.0151
g__Bacteroides	s__Bacteroides_xylanisolvens	0.0151	0.0151
g__Bacteroides	s__Bacteroides_xylanisolvens	0.0151	0.0151
g__Bacteroides	s__Bacteroides_xylanisolvens	0.0151	0.0151
g__Bacteroides	s__Bacteroides_xylanisolvens	0.0151	0.0151
g__Bacteroides	s__Bacteroides_xylanisolvens	0.0151	0.0151
g__Bacteroides	s__Bacteroides_xylanisolvens	0.0151	0.0151
g__Bacteroides	s__Bacteroides_xylanisolvens	0.0151	0.0151
g__Bacteroides	s__Bacteroides_xylanisolvens	0.0151	0.0151
g__Bacteroides	s__Bacteroides_xylanisolvens	0.0151	0.0151
g__Bacteroides	s__Bacteroides_xylanisolvens	0.0151	0.0151
g__Bacteroides	s__Bacteroides_xylanisolvens	0.0151	0.0151
g__Bacteroides	s__Bacteroides_xylanisolvens	0.0151	0.0151
g__Bacteroides	s__Bacteroides_xylanisolvens	0.0151	0.0151
g__Bacteroides	s__Bacteroides_xylanisolvens	0.0151	0.0151
g__Bacteroides	s__Bacteroides_xylanisolvens	0.0151	0.0151
g__Bacteroides	s__Bacteroides_xylanisolvens	0.0151	0.0151
g__Bacteroides	s__Bacteroides_xylanisolvens	0.0151	0.0151
g__Bacteroides	s__Bacteroides_xylanisolvens	0.0151	0.0151
g__Bacteroides	s__Bacteroides_xylanisolvens	0.0151	0.0151
g__Bacteroides	s__Bacteroides_xylanisolvens	0.0151	0.0151
g__Bacteroides	s__Bacteroides_xylanisolvens	0.0151	0.0151
g__Bacteroides	s__Bacteroides_xylanisolvens	0.0151	0.0151
g__Bacteroides	s__Bacteroides_xylanisolvens	0.0151	0.0151
g__Bacteroides	s__Bacteroides_xylanisolvens	0.0151	0.0151
g__Bacteroides	s__Bacteroides_xylanisolvens	0.0151	0.0151
g__Bacteroides	s__Bacteroides_xylanisolvens	0.0151	0.0151
g__Bacteroides	s__Bacteroides_xylanisolvens	0.0151	0.0151
g__Bacteroides	s__Bacteroides_xylanisolvens	0.0151	0.0151

g__Bacteroides	s__Bacteroides_xylanisolvens	0.0151	0.0151
g__Bacteroides	s__Bacteroides_xylanisolvens	0.0151	0.0151
g__Bacteroides	s__Bacteroides_xylanisolvens	0.0151	0.0151
g__Bacteroides	s__Bacteroides_xylanisolvens	0.0151	0.0151
g__Bacteroides	s__Bacteroides_xylanisolvens	0.0151	0.0151
g__Bacteroides	s__Bacteroides_xylanisolvens	0.0151	0.0151
g__Bacteroides	s__Bacteroides_xylanisolvens	0.0151	0.0151
g__Bacteroides	s__Bacteroides_xylanisolvens	0.0151	0.0151
g__Bacteroides	s__Bacteroides_xylanisolvens	0.0151	0.0151
g__Bacteroides	s__Bacteroides_xylanisolvens	0.0151	0.0151
g__Bacteroides	s__Bacteroides_xylanisolvens	0.0151	0.0151
g__Bacteroides	s__Bacteroides_xylanisolvens	0.0151	0.0151
g__Bacteroides	s__Bacteroides_xylanisolvens	0.0151	0.0151
g__Bacteroides	s__Bacteroides_xylanisolvens	0.0151	0.0151
g__Bacteroides	s__Bacteroides_xylanisolvens	0.0151	0.0151
g__Bacteroides	s__Bacteroides_xylanisolvens	0.0151	0.0151
g__Bacteroides	s__Bacteroides_xylanisolvens	0.0151	0.0151
g__Bacteroides	s__Bacteroides_xylanisolvens	0.0151	0.0151
g__Bacteroides	s__Bacteroides_xylanisolvens	0.0151	0.0151
g__Bacteroides	s__Bacteroides_xylanisolvens	0.0151	0.0151
g__Bacteroides	s__Bacteroides_xylanisolvens	0.0151	0.0151
g__Bacteroides	s__Bacteroides_xylanisolvens	0.0151	0.0151
g__Bacteroides	s__Bacteroides_xylanisolvens	0.0151	0.0151
g__Bacteroides	s__Bacteroides_xylanisolvens	0.0151	0.0151
g__Bacteroides	s__Bacteroides_xylanisolvens	0.0151	0.0151
g__Bacteroides	s__Bacteroides_xylanisolvens	0.0151	0.0151
g__Bacteroides	s__Bacteroides_xylanisolvens	0.0151	0.0151
g__Bacteroides	s__Bacteroides_xylanisolvens	0.0151	0.0151
g__Bacteroides	s__Bacteroides_xylanisolvens	0.0151	0.0151
g__Bacteroides	s__Bacteroides_xylanisolvens	0.0151	0.0151
g__Bacteroides	s__Bacteroides_xylanisolvens	0.0151	0.0151
g__Bacteroides	s__Bacteroides_xylanisolvens	0.0151	0.0151
g__Bacteroides	s__Bacteroides_xylanisolvens	0.0151	0.0151
g__Bacteroides	s__Bacteroides_xylanisolvens	0.0151	0.0151
g__Bacteroides	s__Bacteroides_xylanisolvens	0.0151	0.0151
g__Bacteroides	s__Bacteroides_xylanisolvens	0.0151	0.0151
g__Bacteroides	s__Bacteroides_xylanisolvens	0.0151	0.0151
g__Bacteroides	s__Bacteroides_xylanisolvens	0.0151	0.0151
g__Bacteroides	s__Bacteroides_xylanisolvens	0.0151	0.0151
g__Bacteroides	s__Bacteroides_xylanisolvens	0.0151	0.0151
g__Bacteroides	s__Bacteroides_xylanisolvens	0.0151	0.0151
g__Bacteroides	s__Bacteroides_xylanisolvens	0.0151	0.0151
g__Bacteroides	s__Bacteroides_xylanisolvens	0.0151	0.0151

g__Bacteroides	s__Bacteroides_xylanisolvens	0.0151	0.0151
g__Bacteroides	s__Bacteroides_xylanisolvens	0.0151	0.0151
g__Bacteroides	s__Bacteroides_xylanisolvens	0.0151	0.0151
g__Bacteroides	s__Bacteroides_xylanisolvens	0.0151	0.0151
g__Bacteroides	s__Bacteroides_xylanisolvens	0.0151	0.0151
g__Bacteroides	s__Bacteroides_xylanisolvens	0.0151	0.0151
g__Bacteroides	s__Bacteroides_xylanisolvens	0.0151	0.0151
g__Bacteroides	s__Bacteroides_xylanisolvens	0.0151	0.0151
g__Bacteroides	s__Bacteroides_xylanisolvens	0.0151	0.0151
g__Bacteroides	s__Bacteroides_xylanisolvens	0.0151	0.0151
g__Bacteroides	s__Bacteroides_xylanisolvens	0.0151	0.0151
g__Bacteroides	s__Bacteroides_xylanisolvens	0.0151	0.0151
g__Bacteroides	s__Bacteroides_xylanisolvens	0.0151	0.0151
g__Bacteroides	s__Bacteroides_xylanisolvens	0.0151	0.0151
g__Bacteroides	s__Bacteroides_xylanisolvens	0.0151	0.0151
g__Bacteroides	s__Bacteroides_xylanisolvens	0.0151	0.0151
g__Bacteroides	s__Bacteroides_xylanisolvens	0.0151	0.0151
g__Bacteroides	s__Bacteroides_xylanisolvens	0.0151	0.0151
g__Bacteroides	s__Bacteroides_xylanisolvens	0.0151	0.0151
g__Bacteroides	s__Bacteroides_xylanisolvens	0.0151	0.0151
g__Bacteroides	s__Bacteroides_xylanisolvens	0.0151	0.0151
g__Bacteroides	s__Bacteroides_xylanisolvens	0.0151	0.0151
g__Bacteroides	s__Bacteroides_xylanisolvens	0.0151	0.0151
g__Bacteroides	s__Bacteroides_zoogleoformans	0.0151	0.0151
g__Bacteroides	s__Bacteroides_zoogleoformans	0.0151	0.0151
g__Bacteroides	s__Bacteroides_zoogleoformans	0.0151	0.0151
g__Bacteroides	s__Bacteroides_zoogleoformans	0.0151	0.0151
g__Bacteroides	s__Bacteroides_zoogleoformans	0.0151	0.0151
g__Bacteroides	s__Bacteroides_zoogleoformans	0.0151	0.0151
g__Bacteroides	s__Bacteroides_zoogleoformans	0.0151	0.0151
g__Barnesiella	s__Barnesiella_viscericola	0.0151	0.0151
g__Barnesiella	s__Barnesiella_viscericola	0.0151	0.0151
g__Barnesiella	s__Barnesiella_viscericola	0.0151	0.0151
g__Bifidobacterium	s__Bifidobacterium_longum	0.0151	0.0151
g__Blautia	s__Blautia_obeum	0.0151	0.0151
g__Blautia	s__Blautia_obeum	0.0151	0.0151
g__Blautia	s__Blautia_producta	0.0151	0.0151
g__Butyricimonas	s__Butyricimonas_virosa	0.0151	0.0151
g__Butyrivibrio	s__Butyrivibrio_crossotus	0.0151	0.0151
g__Butyrivibrio	s__Butyrivibrio_fibrisolvens	0.0151	0.0151
g__Butyrivibrio	s__Butyrivibrio_hungatei	0.0151	0.0151
g__Butyrivibrio	s__Butyrivibrio_proteoclasticus	0.0151	0.0151
g__Catenibacterium	s__Catenibacterium_mitsuokai	0.0151	0.0151

g__Catenibacterium	s__Catenibacterium_mitsuokai	0.0151	0.0151
g__Catenibacterium	s__Catenibacterium_mitsuokai	0.0151	0.0151
g__Catenibacterium	s__Catenibacterium_mitsuokai	0.0151	0.0151
g__Catenibacterium	s__Catenibacterium_mitsuokai	0.0151	0.0151
g__Catenibacterium	s__Catenibacterium_mitsuokai	0.0151	0.0151
g__Catenibacterium	s__Catenibacterium_mitsuokai	0.0151	0.0151
g__Catenibacterium	s__Catenibacterium_mitsuokai	0.0151	0.0151
g__Catenibacterium	s__Catenibacterium_mitsuokai	0.0151	0.0151
g__Catenibacterium	s__Catenibacterium_mitsuokai	0.0151	0.0151
g__Catenibacterium	s__Catenibacterium_mitsuokai	0.0151	0.0151
g__Catenibacterium	s__Catenibacterium_mitsuokai	0.0151	0.0151
g__Catenibacterium	s__Catenibacterium_mitsuokai	0.0151	0.0151
g__Catenibacterium	s__Catenibacterium_mitsuokai	0.0151	0.0151
g__Catenibacterium	s__Catenibacterium_mitsuokai	0.0151	0.0151
g__Catenibacterium	s__Catenibacterium_mitsuokai	0.0151	0.0151
g__Catenibacterium	s__Catenibacterium_mitsuokai	0.0151	0.0151
g__Catenibacterium	s__Catenibacterium_mitsuokai	0.0151	0.0151
g__Catenibacterium	s__Catenibacterium_mitsuokai	0.0151	0.0151
g__Catenibacterium	s__Catenibacterium_mitsuokai	0.0151	0.0151
g__Catenibacterium	s__Catenibacterium_mitsuokai	0.0151	0.0151
g__Catenibacterium	s__Catenibacterium_mitsuokai	0.0151	0.0151
g__Catenibacterium	s__Catenibacterium_mitsuokai	0.0151	0.0151
g__Catenibacterium	s__Catenibacterium_mitsuokai	0.0151	0.0151
g__Catenibacterium	s__Catenibacterium_mitsuokai	0.0151	0.0151
g__Catenibacterium	s__Catenibacterium_mitsuokai	0.0151	0.0151
g__Catenibacterium	s__Catenibacterium_mitsuokai	0.0151	0.0151
g__Catenibacterium	s__Catenibacterium_mitsuokai	0.0151	0.0151
g__Catenibacterium	s__Catenibacterium_mitsuokai	0.0151	0.0151
g__Catenibacterium	s__Catenibacterium_mitsuokai	0.0151	0.0151
g__Catenibacterium	s__Catenibacterium_mitsuokai	0.0151	0.0151
g__Catenibacterium	s__Catenibacterium_mitsuokai	0.0151	0.0151
g__Catenibacterium	s__Catenibacterium_mitsuokai	0.0151	0.0151
g__Catenibacterium	s__Catenibacterium_mitsuokai	0.0151	0.0151
g__Catenibacterium	s__Catenibacterium_mitsuokai	0.0151	0.0151
g__Catenibacterium	s__Catenibacterium_mitsuokai	0.0151	0.0151
g__Catenibacterium	s__Catenibacterium_mitsuokai	0.0151	0.0151
g__Catenibacterium	s__Catenibacterium_mitsuokai	0.0151	0.0151
g__Coprobacter	s__Coprobacter_fastidiosus	0.0151	0.0151
g__Coprobacter	s__Coprobacter_secundus	0.0151	0.0151
g__Coprobacter	s__Coprobacter_secundus	0.0151	0.0151
g__Coprococcus	s__Coprococcus_comes	0.0151	0.0151
g__Coprococcus	s__Coprococcus_eutactus	0.0151	0.0151
g__Desulfovibrio	s__Desulfovibrio_fairfieldensis	0.0151	0.0151

g__Dorea	s__Dorea_formicigenerans	0.0151	0.0151
g__Dorea	s__Dorea_longicatena	0.0151	0.0151
g__Dorea	s__Dorea_longicatena	0.0151	0.0151
g__Eubacterium	s__Eubacterium_ventriosum	0.0151	0.0151
g__Faecalibacterium	s__Faecalibacterium_prausnitzii	0.0151	0.0151
g__Faecalibaculum	s__Faecalibaculum_rodentium	0.0151	0.0151
g__Faecalibaculum	s__Faecalibaculum_rodentium	0.0151	0.0151
g__Faecalibaculum	s__Faecalibaculum_rodentium	0.0151	0.0151
g__Faecalibaculum	s__Faecalibaculum_rodentium	0.0151	0.0151
g__Faecalibaculum	s__Faecalibaculum_rodentium	0.0151	0.0151
g__Faecalibaculum	s__Faecalibaculum_rodentium	0.0151	0.0151
g__Faecalibaculum	s__Faecalibaculum_rodentium	0.0151	0.0151
g__Faecalibaculum	s__Faecalibaculum_rodentium	0.0151	0.0151
g__Faecalibaculum	s__Faecalibaculum_rodentium	0.0151	0.0151
g__Faecalibaculum	s__Faecalibaculum_rodentium	0.0151	0.0151
g__Flavonifractor	s__Flavonifractor_plautii	0.0151	0.0151
g__Flavonifractor	s__Flavonifractor_plautii	0.0151	0.0151
g__Gardnerella	s__Gardnerella_vaginalis	0.0151	0.0151
g__Gardnerella	s__Gardnerella_vaginalis	0.0151	0.0151
g__Klebsiella	s__Klebsiella_oxytoca	0.0151	0.0151
g__Klebsiella	s__Klebsiella_oxytoca	0.0151	0.0151
g__Klebsiella	s__Klebsiella_oxytoca	0.0151	0.0151
g__Klebsiella	s__Klebsiella_oxytoca	0.0151	0.0151
g__Klebsiella	s__Klebsiella_oxytoca	0.0151	0.0151
g__Klebsiella	s__Klebsiella_oxytoca	0.0151	0.0151
g__Lactobacillus	s__Lactobacillus_acetotolerans	0.0151	0.0151
g__Lactobacillus	s__Lactobacillus_acidophilus	0.0151	0.0151
g__Lactobacillus	s__Lactobacillus_gasseri	0.0151	0.0151
g__Lactobacillus	s__Lactobacillus_gasseri	0.0151	0.0151
g__Lactobacillus	s__Lactobacillus_gasseri	0.0151	0.0151
g__Lactobacillus	s__Lactobacillus_gasseri	0.0151	0.0151
g__Lactobacillus	s__Lactobacillus_gasseri	0.0151	0.0151
g__Lactobacillus	s__Lactobacillus_gasseri	0.0151	0.0151
g__Lactobacillus	s__Lactobacillus_gasseri	0.0151	0.0151
g__Lactobacillus	s__Lactobacillus_gasseri	0.0151	0.0151
g__Lactobacillus	s__Lactobacillus_gasseri	0.0151	0.0151
g__Lactobacillus	s__Lactobacillus_gasseri	0.0151	0.0151
g__Lactobacillus	s__Lactobacillus_gasseri	0.0151	0.0151
g__Lactobacillus	s__Lactobacillus_gasseri	0.0151	0.0151
g__Lactobacillus	s__Lactobacillus_gasseri	0.0151	0.0151
g__Lactobacillus	s__Lactobacillus_gasseri	0.0151	0.0151
g__Lactobacillus	s__Lactobacillus_intestinalis	0.0151	0.0151
g__Lactobacillus	s__Lactobacillus_intestinalis	0.0151	0.0151

g__Lactobacillus	s__Lactobacillus_johnsonii	0.0151	0.0151
g__Lactobacillus	s__Lactobacillus_johnsonii	0.0151	0.0151
g__Lactobacillus	s__Lactobacillus_johnsonii	0.0151	0.0151
g__Lactobacillus	s__Lactobacillus_johnsonii	0.0151	0.0151
g__Lactobacillus	s__Lactobacillus_johnsonii	0.0151	0.0151
g__Lactobacillus	s__Lactobacillus_johnsonii	0.0151	0.0151
g__Lactobacillus	s__Lactobacillus_johnsonii	0.0151	0.0151
g__Lactobacillus	s__Lactobacillus_johnsonii	0.0151	0.0151
g__Lactobacillus	s__Lactobacillus_johnsonii	0.0151	0.0151
g__Lactobacillus	s__Lactobacillus_johnsonii	0.0151	0.0151
g__Lactobacillus	s__Lactobacillus_johnsonii	0.0151	0.0151
g__Lactobacillus	s__Lactobacillus_johnsonii	0.0151	0.0151
g__Lactobacillus	s__Lactobacillus_johnsonii	0.0151	0.0151
g__Lactobacillus	s__Lactobacillus_johnsonii	0.0151	0.0151
g__Lactobacillus	s__Lactobacillus_johnsonii	0.0151	0.0151
g__Lactobacillus	s__Lactobacillus_johnsonii	0.0151	0.0151
g__Lactobacillus	s__Lactobacillus_johnsonii	0.0151	0.0151
g__Lactobacillus	s__Lactobacillus_johnsonii	0.0151	0.0151
g__Lactobacillus	s__Lactobacillus_johnsonii	0.0151	0.0151
g__Lactobacillus	s__Lactobacillus_johnsonii	0.0151	0.0151
g__Lactobacillus	s__Lactobacillus_johnsonii	0.0151	0.0151
g__Lactobacillus	s__Lactobacillus_johnsonii	0.0151	0.0151
g__Lactobacillus	s__Lactobacillus_johnsonii	0.0151	0.0151
g__Lactobacillus	s__Lactobacillus_johnsonii	0.0151	0.0151
g__Lactobacillus	s__Lactobacillus_johnsonii	0.0151	0.0151
g__Lactobacillus	s__Lactobacillus_johnsonii	0.0151	0.0151
g__Lactobacillus	s__Lactobacillus_johnsonii	0.0151	0.0151
g__Lactobacillus	s__Lactobacillus_johnsonii	0.0151	0.0151
g__Lactobacillus	s__Lactobacillus_johnsonii	0.0151	0.0151
g__Lactobacillus	s__Lactobacillus_johnsonii	0.0151	0.0151
g__Lactobacillus	s__Lactobacillus_johnsonii	0.0151	0.0151
g__Lactobacillus	s__Lactobacillus_johnsonii	0.0151	0.0151
g__Lactobacillus	s__Lactobacillus_johnsonii	0.0151	0.0151
g__Lactobacillus	s__Lactobacillus_johnsonii	0.0151	0.0151
g__Lactobacillus	s__Lactobacillus_johnsonii	0.0151	0.0151
g__Lactobacillus	s__Lactobacillus_johnsonii	0.0151	0.0151
g__Lactobacillus	s__Lactobacillus_johnsonii	0.0151	0.0151
g__Lactobacillus	s__Lactobacillus_johnsonii	0.0151	0.0151
g__Lactobacillus	s__Lactobacillus_johnsonii	0.0151	0.0151
g__Lactobacillus	s__Lactobacillus_johnsonii	0.0151	0.0151
g__Lactobacillus	s__Lactobacillus_johnsonii	0.0151	0.0151
g__Lactobacillus	s__Lactobacillus_johnsonii	0.0151	0.0151
g__Lactobacillus	s__Lactobacillus_johnsonii	0.0151	0.0151

g__Lactobacillus	s__Lactobacillus_johnsonii	0.0151	0.0151
g__Lactobacillus	s__Lactobacillus_johnsonii	0.0151	0.0151
g__Lactobacillus	s__Lactobacillus_johnsonii	0.0151	0.0151
g__Lactobacillus	s__Lactobacillus_johnsonii	0.0151	0.0151
g__Lactobacillus	s__Lactobacillus_johnsonii	0.0151	0.0151
g__Lactobacillus	s__Lactobacillus_johnsonii	0.0151	0.0151
g__Lactobacillus	s__Lactobacillus_johnsonii	0.0151	0.0151
g__Lactobacillus	s__Lactobacillus_johnsonii	0.0151	0.0151
g__Lactobacillus	s__Lactobacillus_johnsonii	0.0151	0.0151
g__Lactobacillus	s__Lactobacillus_johnsonii	0.0151	0.0151
g__Lactobacillus	s__Lactobacillus_johnsonii	0.0151	0.0151
g__Lactobacillus	s__Lactobacillus_johnsonii	0.0151	0.0151
g__Lactobacillus	s__Lactobacillus_johnsonii	0.0151	0.0151
g__Lactobacillus	s__Lactobacillus_johnsonii	0.0151	0.0151
g__Lactobacillus	s__Lactobacillus_johnsonii	0.0151	0.0151
g__Lactobacillus	s__Lactobacillus_johnsonii	0.0151	0.0151
g__Lactobacillus	s__Lactobacillus_johnsonii	0.0151	0.0151
g__Lactobacillus	s__Lactobacillus_johnsonii	0.0151	0.0151
g__Lactobacillus	s__Lactobacillus_johnsonii	0.0151	0.0151
g__Lactobacillus	s__Lactobacillus_johnsonii	0.0151	0.0151
g__Lactobacillus	s__Lactobacillus_johnsonii	0.0151	0.0151
g__Lactobacillus	s__Lactobacillus_johnsonii	0.0151	0.0151
g__Lactobacillus	s__Lactobacillus_johnsonii	0.0151	0.0151
g__Lactobacillus	s__Lactobacillus_johnsonii	0.0151	0.0151
g__Lactobacillus	s__Lactobacillus_johnsonii	0.0151	0.0151
g__Lactobacillus	s__Lactobacillus_johnsonii	0.0151	0.0151
g__Lactobacillus	s__Lactobacillus_johnsonii	0.0151	0.0151
g__Lactobacillus	s__Lactobacillus_johnsonii	0.0151	0.0151
g__Lactobacillus	s__Lactobacillus_johnsonii	0.0151	0.0151
g__Lactobacillus	s__Lactobacillus_johnsonii	0.0151	0.0151
g__Lactobacillus	s__Lactobacillus_johnsonii	0.0151	0.0151
g__Lactobacillus	s__Lactobacillus_johnsonii	0.0151	0.0151
g__Lactobacillus	s__Lactobacillus_johnsonii	0.0151	0.0151
g__Lactobacillus	s__Lactobacillus_johnsonii	0.0151	0.0151
g__Lactobacillus	s__Lactobacillus_johnsonii	0.0151	0.0151
g__Lactobacillus	s__Lactobacillus_johnsonii	0.0151	0.0151
g__Lactobacillus	s__Lactobacillus_johnsonii	0.0151	0.0151
g__Lactobacillus	s__Lactobacillus_johnsonii	0.0151	0.0151
g__Lactobacillus	s__Lactobacillus_johnsonii	0.0151	0.0151
g__Lactobacillus	s__Lactobacillus_johnsonii	0.0151	0.0151
g__Lactobacillus	s__Lactobacillus_johnsonii	0.0151	0.0151
g__Lactobacillus	s__Lactobacillus_johnsonii	0.0151	0.0151
g__Lactobacillus	s__Lactobacillus_johnsonii	0.0151	0.0151

g__Lactobacillus	s__Lactobacillus_johnsonii	0.0151	0.0151
g__Lactobacillus	s__Lactobacillus_johnsonii	0.0151	0.0151
g__Lactobacillus	s__Lactobacillus_johnsonii	0.0151	0.0151
g__Lactobacillus	s__Lactobacillus_johnsonii	0.0151	0.0151
g__Lactobacillus	s__Lactobacillus_johnsonii	0.0151	0.0151
g__Lactobacillus	s__Lactobacillus_johnsonii	0.0151	0.0151
g__Lactobacillus	s__Lactobacillus_johnsonii	0.0151	0.0151
g__Lactobacillus	s__Lactobacillus_johnsonii	0.0151	0.0151
g__Lactobacillus	s__Lactobacillus_johnsonii	0.0151	0.0151
g__Lactobacillus	s__Lactobacillus_johnsonii	0.0151	0.0151
g__Lactobacillus	s__Lactobacillus_johnsonii	0.0151	0.0151
g__Lactobacillus	s__Lactobacillus_johnsonii	0.0151	0.0151
g__Lactobacillus	s__Lactobacillus_johnsonii	0.0151	0.0151
g__Lactobacillus	s__Lactobacillus_johnsonii	0.0151	0.0151
g__Lactobacillus	s__Lactobacillus_johnsonii	0.0151	0.0151
g__Lactobacillus	s__Lactobacillus_johnsonii	0.0151	0.0151
g__Lactobacillus	s__Lactobacillus_johnsonii	0.0151	0.0151
g__Lactobacillus	s__Lactobacillus_johnsonii	0.0151	0.0151
g__Lactobacillus	s__Lactobacillus_johnsonii	0.0151	0.0151
g__Lactobacillus	s__Lactobacillus_johnsonii	0.0151	0.0151
g__Lactobacillus	s__Lactobacillus_johnsonii	0.0151	0.0151
g__Lactobacillus	s__Lactobacillus_johnsonii	0.0151	0.0151
g__Lactobacillus	s__Lactobacillus_johnsonii	0.0151	0.0151
g__Lactobacillus	s__Lactobacillus_johnsonii	0.0151	0.0151
g__Lactobacillus	s__Lactobacillus_johnsonii	0.0151	0.0151
g__Lactobacillus	s__Lactobacillus_johnsonii	0.0151	0.0151
g__Lactobacillus	s__Lactobacillus_johnsonii	0.0151	0.0151
g__Lactobacillus	s__Lactobacillus_johnsonii	0.0151	0.0151
g__Lactobacillus	s__Lactobacillus_johnsonii	0.0151	0.0151
g__Lactobacillus	s__Lactobacillus_johnsonii	0.0151	0.0151
g__Lactobacillus	s__Lactobacillus_johnsonii	0.0151	0.0151
g__Lactobacillus	s__Lactobacillus_johnsonii	0.0151	0.0151
g__Lactobacillus	s__Lactobacillus_johnsonii	0.0151	0.0151
g__Lactobacillus	s__Lactobacillus_johnsonii	0.0151	0.0151
g__Lactobacillus	s__Lactobacillus_johnsonii	0.0151	0.0151
g__Lactobacillus	s__Lactobacillus_johnsonii	0.0151	0.0151
g__Lactobacillus	s__Lactobacillus_johnsonii	0.0151	0.0151
g__Lactobacillus	s__Lactobacillus_johnsonii	0.0151	0.0151
g__Lactobacillus	s__Lactobacillus_johnsonii	0.0151	0.0151
g__Lactobacillus	s__Lactobacillus_johnsonii	0.0151	0.0151
g__Lactobacillus	s__Lactobacillus_johnsonii	0.0151	0.0151
g__Lactobacillus	s__Lactobacillus_johnsonii	0.0151	0.0151
g__Lactobacillus	s__Lactobacillus_johnsonii	0.0151	0.0151

g__Lactobacillus	s__Lactobacillus_johnsonii	0.0151	0.0151
g__Lactobacillus	s__Lactobacillus_johnsonii	0.0151	0.0151
g__Lactobacillus	s__Lactobacillus_johnsonii	0.0151	0.0151
g__Lactobacillus	s__Lactobacillus_johnsonii	0.0151	0.0151
g__Lactobacillus	s__Lactobacillus_johnsonii	0.0151	0.0151
g__Lactobacillus	s__Lactobacillus_johnsonii	0.0151	0.0151
g__Lactobacillus	s__Lactobacillus_johnsonii	0.0151	0.0151
g__Lactobacillus	s__Lactobacillus_johnsonii	0.0151	0.0151
g__Lactobacillus	s__Lactobacillus_johnsonii	0.0151	0.0151
g__Lactobacillus	s__Lactobacillus_johnsonii	0.0151	0.0151
g__Lactobacillus	s__Lactobacillus_johnsonii	0.0151	0.0151
g__Lactobacillus	s__Lactobacillus_johnsonii	0.0151	0.0151
g__Lactobacillus	s__Lactobacillus_johnsonii	0.0151	0.0151
g__Lactobacillus	s__Lactobacillus_johnsonii	0.0151	0.0151
g__Lactobacillus	s__Lactobacillus_johnsonii	0.0151	0.0151
g__Lactobacillus	s__Lactobacillus_johnsonii	0.0151	0.0151
g__Lactobacillus	s__Lactobacillus_johnsonii	0.0151	0.0151
g__Lactobacillus	s__Lactobacillus_johnsonii	0.0151	0.0151
g__Lactobacillus	s__Lactobacillus_johnsonii	0.0151	0.0151
g__Lactobacillus	s__Lactobacillus_johnsonii	0.0151	0.0151
g__Lactobacillus	s__Lactobacillus_johnsonii	0.0151	0.0151
g__Lactobacillus	s__Lactobacillus_johnsonii	0.0151	0.0151
g__Lactobacillus	s__Lactobacillus_johnsonii	0.0151	0.0151
g__Lactobacillus	s__Lactobacillus_johnsonii	0.0151	0.0151
g__Lactobacillus	s__Lactobacillus_johnsonii	0.0151	0.0151
g__Lactobacillus	s__Lactobacillus_johnsonii	0.0151	0.0151
g__Lactobacillus	s__Lactobacillus_johnsonii	0.0151	0.0151
g__Lactobacillus	s__Lactobacillus_johnsonii	0.0151	0.0151
g__Lactobacillus	s__Lactobacillus_johnsonii	0.0151	0.0151
g__Lactobacillus	s__Lactobacillus_johnsonii	0.0151	0.0151
g__Lactobacillus	s__Lactobacillus_johnsonii	0.0151	0.0151
g__Lactobacillus	s__Lactobacillus_johnsonii	0.0151	0.0151
g__Lactobacillus	s__Lactobacillus_johnsonii	0.0151	0.0151
g__Lactobacillus	s__Lactobacillus_johnsonii	0.0151	0.0151
g__Lactobacillus	s__Lactobacillus_johnsonii	0.0151	0.0151
g__Lactobacillus	s__Lactobacillus_johnsonii	0.0151	0.0151
g__Lactobacillus	s__Lactobacillus_johnsonii	0.0151	0.0151
g__Lactobacillus	s__Lactobacillus_johnsonii	0.0151	0.0151
g__Lactobacillus	s__Lactobacillus_johnsonii	0.0151	0.0151
g__Lactobacillus	s__Lactobacillus_johnsonii	0.0151	0.0151
g__Lactobacillus	s__Lactobacillus_johnsonii	0.0151	0.0151
g__Lactobacillus	s__Lactobacillus_johnsonii	0.0151	0.0151
g__Lactobacillus	s__Lactobacillus_johnsonii	0.0151	0.0151

g__Lactobacillus	s__Lactobacillus_johnsonii	0.0151	0.0151
g__Lactobacillus	s__Lactobacillus_johnsonii	0.0151	0.0151
g__Lactobacillus	s__Lactobacillus_johnsonii	0.0151	0.0151
g__Lactobacillus	s__Lactobacillus_johnsonii	0.0151	0.0151
g__Lactobacillus	s__Lactobacillus_johnsonii	0.0151	0.0151
g__Lactobacillus	s__Lactobacillus_johnsonii	0.0151	0.0151
g__Lactobacillus	s__Lactobacillus_johnsonii	0.0151	0.0151
g__Lactobacillus	s__Lactobacillus_johnsonii	0.0151	0.0151
g__Lactobacillus	s__Lactobacillus_johnsonii	0.0151	0.0151
g__Lactobacillus	s__Lactobacillus_johnsonii	0.0151	0.0151
g__Lactobacillus	s__Lactobacillus_johnsonii	0.0151	0.0151
g__Lactobacillus	s__Lactobacillus_johnsonii	0.0151	0.0151
g__Lactobacillus	s__Lactobacillus_paragasseri	0.0151	0.0151
g__Lactobacillus	s__Lactobacillus_paragasseri	0.0151	0.0151
g__Lactobacillus	s__Lactobacillus_paragasseri	0.0151	0.0151
g__Lactobacillus	s__Lactobacillus_paragasseri	0.0151	0.0151
g__Lactobacillus	s__Lactobacillus_paragasseri	0.0151	0.0151
g__Lactobacillus	s__Lactobacillus_paragasseri	0.0151	0.0151
g__Lactobacillus	s__Lactobacillus_paragasseri	0.0151	0.0151
g__Lactobacillus	s__Lactobacillus_paragasseri	0.0151	0.0151
g__Lactobacillus	s__Lactobacillus_paragasseri	0.0151	0.0151
g__Lactobacillus	s__Lactobacillus_paragasseri	0.0151	0.0151
g__Lactobacillus	s__Lactobacillus_taiwanensis	0.0151	0.0151
g__Lactobacillus	s__Lactobacillus_taiwanensis	0.0151	0.0151
g__Lactobacillus	s__Lactobacillus_taiwanensis	0.0151	0.0151
g__Lactobacillus	s__Lactobacillus_taiwanensis	0.0151	0.0151
g__Lactobacillus	s__Lactobacillus_taiwanensis	0.0151	0.0151
g__Lactobacillus	s__Lactobacillus_taiwanensis	0.0151	0.0151
g__Lactobacillus	s__Lactobacillus_taiwanensis	0.0151	0.0151
g__Lactobacillus	s__Lactobacillus_taiwanensis	0.0151	0.0151
g__Lactobacillus	s__Lactobacillus_taiwanensis	0.0151	0.0151
g__Lactobacillus	s__Lactobacillus_taiwanensis	0.0151	0.0151
g__Lactobacillus	s__Lactobacillus_taiwanensis	0.0151	0.0151
g__Lactobacillus	s__Lactobacillus_taiwanensis	0.0151	0.0151
g__Lactobacillus	s__Lactobacillus_taiwanensis	0.0151	0.0151
g__Lactobacillus	s__Lactobacillus_taiwanensis	0.0151	0.0151
g__Lactobacillus	s__Lactobacillus_taiwanensis	0.0151	0.0151
g__Lactobacillus	s__Lactobacillus_taiwanensis	0.0151	0.0151
g__Lactobacillus	s__Lactobacillus_taiwanensis	0.0151	0.0151
g__Lactobacillus	s__Lactobacillus_taiwanensis	0.0151	0.0151
g__Lactobacillus	s__Lactobacillus_taiwanensis	0.0151	0.0151
g__Lactobacillus	s__Lactobacillus_taiwanensis	0.0151	0.0151
g__Lactobacillus	s__Lactobacillus_taiwanensis	0.0151	0.0151

g__Lactobacillus	s__Lactobacillus_taiwanensis	0.0151	0.0151
g__Lactobacillus	s__Lactobacillus_taiwanensis	0.0151	0.0151
g__Lactobacillus	s__Lactobacillus_taiwanensis	0.0151	0.0151
g__Lactobacillus	s__Lactobacillus_taiwanensis	0.0151	0.0151
g__Lactobacillus	s__Lactobacillus_taiwanensis	0.0151	0.0151
g__Lactobacillus	s__Lactobacillus_taiwanensis	0.0151	0.0151
g__Lactobacillus	s__Lactobacillus_taiwanensis	0.0151	0.0151
g__Lactobacillus	s__Lactobacillus_taiwanensis	0.0151	0.0151
g__Lactobacillus	s__Lactobacillus_taiwanensis	0.0151	0.0151
g__Lactobacillus	s__Lactobacillus_taiwanensis	0.0151	0.0151
g__Lactobacillus	s__Lactobacillus_taiwanensis	0.0151	0.0151
g__Lactobacillus	s__Lactobacillus_taiwanensis	0.0151	0.0151
g__Lactobacillus	s__Lactobacillus_taiwanensis	0.0151	0.0151
g__Lactobacillus	s__Lactobacillus_taiwanensis	0.0151	0.0151
g__Lactobacillus	s__Lactobacillus_taiwanensis	0.0151	0.0151
g__Lactobacillus	s__Lactobacillus_taiwanensis	0.0151	0.0151
g__Lactobacillus	s__Lactobacillus_taiwanensis	0.0151	0.0151
g__Lactobacillus	s__Lactobacillus_taiwanensis	0.0151	0.0151
g__Lactobacillus	s__Lactobacillus_taiwanensis	0.0151	0.0151
g__Lactobacillus	s__Lactobacillus_taiwanensis	0.0151	0.0151
g__Lactobacillus	s__Lactobacillus_taiwanensis	0.0151	0.0151
g__Lawsonibacter	s__Lawsonibacter_asaccharolyticus	0.0151	0.0151
g__Marvinbryantia	s__Marvinbryantia_formatexigens	0.0151	0.0151
g__Mucispirillum	s__Mucispirillum_schaedleri	0.0151	0.0151
g__Mucispirillum	s__Mucispirillum_schaedleri	0.0151	0.0151
g__Mucispirillum	s__Mucispirillum_schaedleri	0.0151	0.0151
g__Mucispirillum	s__Mucispirillum_schaedleri	0.0151	0.0151
g__Mucispirillum	s__Mucispirillum_schaedleri	0.0151	0.0151
g__Mucispirillum	s__Mucispirillum_schaedleri	0.0151	0.0151
g__Mucispirillum	s__Mucispirillum_schaedleri	0.0151	0.0151
g__Mucispirillum	s__Mucispirillum_schaedleri	0.0151	0.0151
g__Mucispirillum	s__Mucispirillum_schaedleri	0.0151	0.0151
g__Mucispirillum	s__Mucispirillum_schaedleri	0.0151	0.0151
g__Mucispirillum	s__Mucispirillum_schaedleri	0.0151	0.0151
g__Mucispirillum	s__Mucispirillum_schaedleri	0.0151	0.0151
g__Mucispirillum	s__Mucispirillum_schaedleri	0.0151	0.0151
g__Mucispirillum	s__Mucispirillum_schaedleri	0.0151	0.0151
g__Mucispirillum	s__Mucispirillum_schaedleri	0.0151	0.0151
g__Mucispirillum	s__Mucispirillum_schaedleri	0.0151	0.0151
g__Mucispirillum	s__Mucispirillum_schaedleri	0.0151	0.0151
g__Mucispirillum	s__Mucispirillum_schaedleri	0.0151	0.0151
g__Mucispirillum	s__Mucispirillum_schaedleri	0.0151	0.0151
g__Mucispirillum	s__Mucispirillum_schaedleri	0.0151	0.0151

g__Mucispirillum	s__Mucispirillum_schaedleri	0.0151	0.0151
g__Mucispirillum	s__Mucispirillum_schaedleri	0.0151	0.0151
g__Mucispirillum	s__Mucispirillum_schaedleri	0.0151	0.0151
g__Mucispirillum	s__Mucispirillum_schaedleri	0.0151	0.0151
g__Mucispirillum	s__Mucispirillum_schaedleri	0.0151	0.0151
g__Mucispirillum	s__Mucispirillum_schaedleri	0.0151	0.0151
g__Mucispirillum	s__Mucispirillum_schaedleri	0.0151	0.0151
g__Mucispirillum	s__Mucispirillum_schaedleri	0.0151	0.0151
g__Mucispirillum	s__Mucispirillum_schaedleri	0.0151	0.0151
g__Mucispirillum	s__Mucispirillum_schaedleri	0.0151	0.0151
g__Mucispirillum	s__Mucispirillum_schaedleri	0.0151	0.0151
g__Mucispirillum	s__Mucispirillum_schaedleri	0.0151	0.0151
g__Mucispirillum	s__Mucispirillum_schaedleri	0.0151	0.0151
g__Mucispirillum	s__Mucispirillum_schaedleri	0.0151	0.0151
g__Mucispirillum	s__Mucispirillum_schaedleri	0.0151	0.0151
g__Mucispirillum	s__Mucispirillum_schaedleri	0.0151	0.0151
g__Mucispirillum	s__Mucispirillum_schaedleri	0.0151	0.0151
g__Mucispirillum	s__Mucispirillum_schaedleri	0.0151	0.0151
g__Mucispirillum	s__Mucispirillum_schaedleri	0.0151	0.0151
g__Mucispirillum	s__Mucispirillum_schaedleri	0.0151	0.0151
g__Mucispirillum	s__Mucispirillum_schaedleri	0.0151	0.0151
g__Mucispirillum	s__Mucispirillum_schaedleri	0.0151	0.0151
g__Mucispirillum	s__Mucispirillum_schaedleri	0.0151	0.0151
g__Mucispirillum	s__Mucispirillum_schaedleri	0.0151	0.0151
g__Mucispirillum	s__Mucispirillum_schaedleri	0.0151	0.0151
g__Mucispirillum	s__Mucispirillum_schaedleri	0.0151	0.0151
g__Mucispirillum	s__Mucispirillum_schaedleri	0.0151	0.0151
g__Mucispirillum	s__Mucispirillum_schaedleri	0.0151	0.0151
g__Mucispirillum	s__Mucispirillum_schaedleri	0.0151	0.0151
g__Mucispirillum	s__Mucispirillum_schaedleri	0.0151	0.0151
g__Mucispirillum	s__Mucispirillum_schaedleri	0.0151	0.0151
g__Mucispirillum	s__Mucispirillum_schaedleri	0.0151	0.0151
g__Mucispirillum	s__Mucispirillum_schaedleri	0.0151	0.0151
g__Mucispirillum	s__Mucispirillum_schaedleri	0.0151	0.0151
g__Mucispirillum	s__Mucispirillum_schaedleri	0.0151	0.0151
g__Mucispirillum	s__Mucispirillum_schaedleri	0.0151	0.0151
g__Mucispirillum	s__Mucispirillum_schaedleri	0.0151	0.0151
g__Mucispirillum	s__Mucispirillum_schaedleri	0.0151	0.0151
g__Mucispirillum	s__Mucispirillum_schaedleri	0.0151	0.0151
g__Mucispirillum	s__Mucispirillum_schaedleri	0.0151	0.0151
g__Mucispirillum	s__Mucispirillum_schaedleri	0.0151	0.0151
g__Mucispirillum	s__Mucispirillum_schaedleri	0.0151	0.0151
g__Mucispirillum	s__Mucispirillum_schaedleri	0.0151	0.0151

g__Mucispirillum	s__Mucispirillum_schaedleri	0.0151	0.0151
g__Mucispirillum	s__Mucispirillum_schaedleri	0.0151	0.0151
g__Mucispirillum	s__Mucispirillum_schaedleri	0.0151	0.0151
g__Mucispirillum	s__Mucispirillum_schaedleri	0.0151	0.0151
g__Mucispirillum	s__Mucispirillum_schaedleri	0.0151	0.0151
g__Mucispirillum	s__Mucispirillum_schaedleri	0.0151	0.0151
g__Mucispirillum	s__Mucispirillum_schaedleri	0.0151	0.0151
g__Mucispirillum	s__Mucispirillum_schaedleri	0.0151	0.0151
g__Mucispirillum	s__Mucispirillum_schaedleri	0.0151	0.0151
g__Mucispirillum	s__Mucispirillum_schaedleri	0.0151	0.0151
g__Mucispirillum	s__Mucispirillum_schaedleri	0.0151	0.0151
g__Mucispirillum	s__Mucispirillum_schaedleri	0.0151	0.0151
g__Mucispirillum	s__Mucispirillum_schaedleri	0.0151	0.0151
g__Mucispirillum	s__Mucispirillum_schaedleri	0.0151	0.0151
g__Mucispirillum	s__Mucispirillum_schaedleri	0.0151	0.0151
g__Mucispirillum	s__Mucispirillum_schaedleri	0.0151	0.0151
g__Mucispirillum	s__Mucispirillum_schaedleri	0.0151	0.0151
g__Mucispirillum	s__Mucispirillum_schaedleri	0.0151	0.0151
g__Mucispirillum	s__Mucispirillum_schaedleri	0.0151	0.0151
g__Mucispirillum	s__Mucispirillum_schaedleri	0.0151	0.0151
g__Mucispirillum	s__Mucispirillum_schaedleri	0.0151	0.0151
g__Mucispirillum	s__Mucispirillum_schaedleri	0.0151	0.0151
g__Mucispirillum	s__Mucispirillum_schaedleri	0.0151	0.0151
g__Mucispirillum	s__Mucispirillum_schaedleri	0.0151	0.0151
g__Mucispirillum	s__Mucispirillum_schaedleri	0.0151	0.0151
g__Mucispirillum	s__Mucispirillum_schaedleri	0.0151	0.0151
g__Mucispirillum	s__Mucispirillum_schaedleri	0.0151	0.0151
g__Mucispirillum	s__Mucispirillum_schaedleri	0.0151	0.0151
g__Mucispirillum	s__Mucispirillum_schaedleri	0.0151	0.0151
g__Mucispirillum	s__Mucispirillum_schaedleri	0.0151	0.0151
g__Mucispirillum	s__Mucispirillum_schaedleri	0.0151	0.0151
g__Mucispirillum	s__Mucispirillum_schaedleri	0.0151	0.0151
g__Mucispirillum	s__Mucispirillum_schaedleri	0.0151	0.0151
g__Mucispirillum	s__Mucispirillum_schaedleri	0.0151	0.0151
g__Mucispirillum	s__Mucispirillum_schaedleri	0.0151	0.0151
g__Mucispirillum	s__Mucispirillum_schaedleri	0.0151	0.0151
g__Mucispirillum	s__Mucispirillum_schaedleri	0.0151	0.0151
g__Mucispirillum	s__Mucispirillum_schaedleri	0.0151	0.0151
g__Mucispirillum	s__Mucispirillum_schaedleri	0.0151	0.0151
g__Mucispirillum	s__Mucispirillum_schaedleri	0.0151	0.0151
g__Mucispirillum	s__Mucispirillum_schaedleri	0.0151	0.0151
g__Mucispirillum	s__Mucispirillum_schaedleri	0.0151	0.0151
g__Mucispirillum	s__Mucispirillum_schaedleri	0.0151	0.0151

g__Mucispirillum	s__Mucispirillum_schaedleri	0.0151	0.0151
g__Mucispirillum	s__Mucispirillum_schaedleri	0.0151	0.0151
g__Mucispirillum	s__Mucispirillum_schaedleri	0.0151	0.0151
g__Mucispirillum	s__Mucispirillum_schaedleri	0.0151	0.0151
g__Mucispirillum	s__Mucispirillum_schaedleri	0.0151	0.0151
g__Mucispirillum	s__Mucispirillum_schaedleri	0.0151	0.0151
g__Mucispirillum	s__Mucispirillum_schaedleri	0.0151	0.0151
g__Mucispirillum	s__Mucispirillum_schaedleri	0.0151	0.0151
g__Mucispirillum	s__Mucispirillum_schaedleri	0.0151	0.0151
g__Mucispirillum	s__Mucispirillum_schaedleri	0.0151	0.0151
g__Mucispirillum	s__Mucispirillum_schaedleri	0.0151	0.0151
g__Mucispirillum	s__Mucispirillum_schaedleri	0.0151	0.0151
g__Mucispirillum	s__Mucispirillum_schaedleri	0.0151	0.0151
g__Mucispirillum	s__Mucispirillum_schaedleri	0.0151	0.0151
g__Mucispirillum	s__Mucispirillum_schaedleri	0.0151	0.0151
g__Mucispirillum	s__Mucispirillum_schaedleri	0.0151	0.0151
g__Mucispirillum	s__Mucispirillum_schaedleri	0.0151	0.0151
g__Mucispirillum	s__Mucispirillum_schaedleri	0.0151	0.0151
g__Mucispirillum	s__Mucispirillum_schaedleri	0.0151	0.0151
g__Mucispirillum	s__Mucispirillum_schaedleri	0.0151	0.0151
g__Mucispirillum	s__Mucispirillum_schaedleri	0.0151	0.0151
g__Mucispirillum	s__Mucispirillum_schaedleri	0.0151	0.0151
g__Mucispirillum	s__Mucispirillum_schaedleri	0.0151	0.0151
g__Mucispirillum	s__Mucispirillum_schaedleri	0.0151	0.0151
g__Mucispirillum	s__Mucispirillum_schaedleri	0.0151	0.0151
g__Mucispirillum	s__Mucispirillum_schaedleri	0.0151	0.0151
g__Mucispirillum	s__Mucispirillum_schaedleri	0.0151	0.0151
g__Mucispirillum	s__Mucispirillum_schaedleri	0.0151	0.0151
g__Mucispirillum	s__Mucispirillum_schaedleri	0.0151	0.0151
g__Mucispirillum	s__Mucispirillum_schaedleri	0.0151	0.0151
g__Mucispirillum	s__Mucispirillum_schaedleri	0.0151	0.0151
g__Mucispirillum	s__Mucispirillum_schaedleri	0.0151	0.0151
g__Mucispirillum	s__Mucispirillum_schaedleri	0.0151	0.0151
g__Mucispirillum	s__Mucispirillum_schaedleri	0.0151	0.0151
g__Mucispirillum	s__Mucispirillum_schaedleri	0.0151	0.0151
g__Mucispirillum	s__Mucispirillum_schaedleri	0.0151	0.0151
g__Mucispirillum	s__Mucispirillum_schaedleri	0.0151	0.0151
g__Mucispirillum	s__Mucispirillum_schaedleri	0.0151	0.0151
g__Mucispirillum	s__Mucispirillum_schaedleri	0.0151	0.0151
g__Mucispirillum	s__Mucispirillum_schaedleri	0.0151	0.0151
g__Mucispirillum	s__Mucispirillum_schaedleri	0.0151	0.0151
g__Mucispirillum	s__Mucispirillum_schaedleri	0.0151	0.0151
g__Mucispirillum	s__Mucispirillum_schaedleri	0.0151	0.0151

g__Mucispirillum	s__Mucispirillum_schaedleri	0.0151	0.0151
g__Mucispirillum	s__Mucispirillum_schaedleri	0.0151	0.0151
g__Mucispirillum	s__Mucispirillum_schaedleri	0.0151	0.0151
g__Mucispirillum	s__Mucispirillum_schaedleri	0.0151	0.0151
g__Mucispirillum	s__Mucispirillum_schaedleri	0.0151	0.0151
g__Mucispirillum	s__Mucispirillum_schaedleri	0.0151	0.0151
g__Mucispirillum	s__Mucispirillum_schaedleri	0.0151	0.0151
g__Mucispirillum	s__Mucispirillum_schaedleri	0.0151	0.0151
g__Mucispirillum	s__Mucispirillum_schaedleri	0.0151	0.0151
g__Mucispirillum	s__Mucispirillum_schaedleri	0.0151	0.0151
g__Mucispirillum	s__Mucispirillum_schaedleri	0.0151	0.0151
g__Mucispirillum	s__Mucispirillum_schaedleri	0.0151	0.0151
g__Mucispirillum	s__Mucispirillum_schaedleri	0.0151	0.0151
g__Mucispirillum	s__Mucispirillum_schaedleri	0.0151	0.0151
g__Mucispirillum	s__Mucispirillum_schaedleri	0.0151	0.0151
g__Mucispirillum	s__Mucispirillum_schaedleri	0.0151	0.0151
g__Mucispirillum	s__Mucispirillum_schaedleri	0.0151	0.0151
g__Mucispirillum	s__Mucispirillum_schaedleri	0.0151	0.0151
g__Mucispirillum	s__Mucispirillum_schaedleri	0.0151	0.0151
g__Mucispirillum	s__Mucispirillum_schaedleri	0.0151	0.0151
g__Mucispirillum	s__Mucispirillum_schaedleri	0.0151	0.0151
g__Mucispirillum	s__Mucispirillum_schaedleri	0.0151	0.0151
g__Mucispirillum	s__Mucispirillum_schaedleri	0.0151	0.0151
g__Mucispirillum	s__Mucispirillum_schaedleri	0.0151	0.0151
g__Mucispirillum	s__Mucispirillum_schaedleri	0.0151	0.0151
g__Mucispirillum	s__Mucispirillum_schaedleri	0.0151	0.0151
g__Mucispirillum	s__Mucispirillum_schaedleri	0.0151	0.0151
g__Mucispirillum	s__Mucispirillum_schaedleri	0.0151	0.0151
g__Mucispirillum	s__Mucispirillum_schaedleri	0.0151	0.0151
g__Mucispirillum	s__Mucispirillum_schaedleri	0.0151	0.0151
g__Mucispirillum	s__Mucispirillum_schaedleri	0.0151	0.0151
g__Mucispirillum	s__Mucispirillum_schaedleri	0.0151	0.0151
g__Mucispirillum	s__Mucispirillum_schaedleri	0.0151	0.0151
g__Mucispirillum	s__Mucispirillum_schaedleri	0.0151	0.0151
g__Mucispirillum	s__Mucispirillum_schaedleri	0.0151	0.0151
g__Mucispirillum	s__Mucispirillum_schaedleri	0.0151	0.0151
g__Mucispirillum	s__Mucispirillum_schaedleri	0.0151	0.0151
g__Mucispirillum	s__Mucispirillum_schaedleri	0.0151	0.0151
g__Mucispirillum	s__Mucispirillum_schaedleri	0.0151	0.0151
g__Mucispirillum	s__Mucispirillum_schaedleri	0.0151	0.0151
g__Mucispirillum	s__Mucispirillum_schaedleri	0.0151	0.0151
g__Mucispirillum	s__Mucispirillum_schaedleri	0.0151	0.0151
g__Mucispirillum	s__Mucispirillum_schaedleri	0.0151	0.0151

g__Mucispirillum	s__Mucispirillum_schaedleri	0.0151	0.0151
g__Mucispirillum	s__Mucispirillum_schaedleri	0.0151	0.0151
g__Mucispirillum	s__Mucispirillum_schaedleri	0.0151	0.0151
g__Mucispirillum	s__Mucispirillum_schaedleri	0.0151	0.0151
g__Mucispirillum	s__Mucispirillum_schaedleri	0.0151	0.0151
g__Mucispirillum	s__Mucispirillum_schaedleri	0.0151	0.0151
g__Mucispirillum	s__Mucispirillum_schaedleri	0.0151	0.0151
g__Mucispirillum	s__Mucispirillum_schaedleri	0.0151	0.0151
g__Mucispirillum	s__Mucispirillum_schaedleri	0.0151	0.0151
g__Mucispirillum	s__Mucispirillum_schaedleri	0.0151	0.0151
g__Mucispirillum	s__Mucispirillum_schaedleri	0.0151	0.0151
g__Mucispirillum	s__Mucispirillum_schaedleri	0.0151	0.0151
g__Mucispirillum	s__Mucispirillum_schaedleri	0.0151	0.0151
g__Mucispirillum	s__Mucispirillum_schaedleri	0.0151	0.0151
g__Mucispirillum	s__Mucispirillum_schaedleri	0.0151	0.0151
g__Mucispirillum	s__Mucispirillum_schaedleri	0.0151	0.0151
g__Mucispirillum	s__Mucispirillum_schaedleri	0.0151	0.0151
g__Mucispirillum	s__Mucispirillum_schaedleri	0.0151	0.0151
g__Mucispirillum	s__Mucispirillum_schaedleri	0.0151	0.0151
g__Mucispirillum	s__Mucispirillum_schaedleri	0.0151	0.0151
g__Mucispirillum	s__Mucispirillum_schaedleri	0.0151	0.0151
g__Mucispirillum	s__Mucispirillum_schaedleri	0.0151	0.0151
g__Mucispirillum	s__Mucispirillum_schaedleri	0.0151	0.0151
g__Mucispirillum	s__Mucispirillum_schaedleri	0.0151	0.0151
g__Mucispirillum	s__Mucispirillum_schaedleri	0.0151	0.0151
g__Mucispirillum	s__Mucispirillum_schaedleri	0.0151	0.0151
g__Mucispirillum	s__Mucispirillum_schaedleri	0.0151	0.0151
g__Mucispirillum	s__Mucispirillum_schaedleri	0.0151	0.0151
g__Mucispirillum	s__Mucispirillum_schaedleri	0.0151	0.0151
g__Mucispirillum	s__Mucispirillum_schaedleri	0.0151	0.0151
g__Mucispirillum	s__Mucispirillum_schaedleri	0.0151	0.0151
g__Mucispirillum	s__Mucispirillum_schaedleri	0.0151	0.0151
g__Mucispirillum	s__Mucispirillum_schaedleri	0.0151	0.0151
g__Mucispirillum	s__Mucispirillum_schaedleri	0.0151	0.0151
g__Mucispirillum	s__Mucispirillum_schaedleri	0.0151	0.0151
g__Mucispirillum	s__Mucispirillum_schaedleri	0.0151	0.0151
g__Mucispirillum	s__Mucispirillum_schaedleri	0.0151	0.0151
g__Mucispirillum	s__Mucispirillum_schaedleri	0.0151	0.0151
g__Mucispirillum	s__Mucispirillum_schaedleri	0.0151	0.0151
g__Mucispirillum	s__Mucispirillum_schaedleri	0.0151	0.0151
g__Mucispirillum	s__Mucispirillum_schaedleri	0.0151	0.0151
g__Mucispirillum	s__Mucispirillum_schaedleri	0.0151	0.0151
g__Mucispirillum	s__Mucispirillum_schaedleri	0.0151	0.0151

g__Mucispirillum	s__Mucispirillum_schaedleri	0.0151	0.0151
g__Mucispirillum	s__Mucispirillum_schaedleri	0.0151	0.0151
g__Mucispirillum	s__Mucispirillum_schaedleri	0.0151	0.0151
g__Mucispirillum	s__Mucispirillum_schaedleri	0.0151	0.0151
g__Mucispirillum	s__Mucispirillum_schaedleri	0.0151	0.0151
g__Mucispirillum	s__Mucispirillum_schaedleri	0.0151	0.0151
g__Mucispirillum	s__Mucispirillum_schaedleri	0.0151	0.0151
g__Mucispirillum	s__Mucispirillum_schaedleri	0.0151	0.0151
g__Mucispirillum	s__Mucispirillum_schaedleri	0.0151	0.0151
g__Mucispirillum	s__Mucispirillum_schaedleri	0.0151	0.0151
g__Mucispirillum	s__Mucispirillum_schaedleri	0.0151	0.0151
g__Mucispirillum	s__Mucispirillum_schaedleri	0.0151	0.0151
g__Mucispirillum	s__Mucispirillum_schaedleri	0.0151	0.0151
g__Mucispirillum	s__Mucispirillum_schaedleri	0.0151	0.0151
g__Mucispirillum	s__Mucispirillum_schaedleri	0.0151	0.0151
g__Mucispirillum	s__Mucispirillum_schaedleri	0.0151	0.0151
g__Mucispirillum	s__Mucispirillum_schaedleri	0.0151	0.0151
g__Mucispirillum	s__Mucispirillum_schaedleri	0.0151	0.0151
g__Mucispirillum	s__Mucispirillum_schaedleri	0.0151	0.0151
g__Mucispirillum	s__Mucispirillum_schaedleri	0.0151	0.0151
g__Mucispirillum	s__Mucispirillum_schaedleri	0.0151	0.0151
g__Mucispirillum	s__Mucispirillum_schaedleri	0.0151	0.0151
g__Mucispirillum	s__Mucispirillum_schaedleri	0.0151	0.0151
g__Mucispirillum	s__Mucispirillum_schaedleri	0.0151	0.0151
g__Mucispirillum	s__Mucispirillum_schaedleri	0.0151	0.0151
g__Mucispirillum	s__Mucispirillum_schaedleri	0.0151	0.0151
g__Mucispirillum	s__Mucispirillum_schaedleri	0.0151	0.0151
g__Mucispirillum	s__Mucispirillum_schaedleri	0.0151	0.0151
g__Mucispirillum	s__Mucispirillum_schaedleri	0.0151	0.0151
g__Mucispirillum	s__Mucispirillum_schaedleri	0.0151	0.0151
g__Mucispirillum	s__Mucispirillum_schaedleri	0.0151	0.0151
g__Mucispirillum	s__Mucispirillum_schaedleri	0.0151	0.0151
g__Mucispirillum	s__Mucispirillum_schaedleri	0.0151	0.0151
g__Mucispirillum	s__Mucispirillum_schaedleri	0.0151	0.0151
g__Mucispirillum	s__Mucispirillum_schaedleri	0.0151	0.0151
g__Muribaculum	s__Muribaculum_intestinale	0.0151	0.0151
g__Muribaculum	s__Muribaculum_intestinale	0.0151	0.0151
g__Muribaculum	s__Muribaculum_intestinale	0.0151	0.0151
g__Muribaculum	s__Muribaculum_intestinale	0.0151	0.0151
g__Muribaculum	s__Muribaculum_intestinale	0.0151	0.0151
g__Odoribacter	s__Odoribacter_splanchnicus	0.0151	0.0151
g__Odoribacter	s__Odoribacter_splanchnicus	0.0151	0.0151
g__Ornithobacterium	s__Ornithobacterium_rhinotracheale	0.0151	0.0151

g__Parabacteroides	s__Parabacteroides_distasonis	0.0151	0.0151
g__Parabacteroides	s__Parabacteroides_distasonis	0.0151	0.0151
g__Parabacteroides	s__Parabacteroides_distasonis	0.0151	0.0151
g__Parabacteroides	s__Parabacteroides_distasonis	0.0151	0.0151
g__Parabacteroides	s__Parabacteroides_distasonis	0.0151	0.0151
g__Parabacteroides	s__Parabacteroides_distasonis	0.0151	0.0151
g__Parabacteroides	s__Parabacteroides_distasonis	0.0151	0.0151
g__Parabacteroides	s__Parabacteroides_distasonis	0.0151	0.0151
g__Parabacteroides	s__Parabacteroides_distasonis	0.0151	0.0151
g__Parabacteroides	s__Parabacteroides_distasonis	0.0151	0.0151
g__Parabacteroides	s__Parabacteroides_distasonis	0.0151	0.0151
g__Parabacteroides	s__Parabacteroides_distasonis	0.0151	0.0151
g__Parabacteroides	s__Parabacteroides_distasonis	0.0151	0.0151
g__Parabacteroides	s__Parabacteroides_goldsteinii	0.0151	0.0151
g__Parabacteroides	s__Parabacteroides_goldsteinii	0.0151	0.0151
g__Parabacteroides	s__Parabacteroides_goldsteinii	0.0151	0.0151
g__Parabacteroides	s__Parabacteroides_goldsteinii	0.0151	0.0151
g__Parabacteroides	s__Parabacteroides_goldsteinii	0.0151	0.0151
g__Parabacteroides	s__Parabacteroides_goldsteinii	0.0151	0.0151
g__Parabacteroides	s__Parabacteroides_goldsteinii	0.0151	0.0151
g__Parabacteroides	s__Parabacteroides_goldsteinii	0.0151	0.0151
g__Parabacteroides	s__Parabacteroides_goldsteinii	0.0151	0.0151
g__Parabacteroides	s__Parabacteroides_goldsteinii	0.0151	0.0151
g__Parabacteroides	s__Parabacteroides_goldsteinii	0.0151	0.0151
g__Parabacteroides	s__Parabacteroides_goldsteinii	0.0151	0.0151
g__Parabacteroides	s__Parabacteroides_goldsteinii	0.0151	0.0151
g__Parabacteroides	s__Parabacteroides_goldsteinii	0.0151	0.0151
g__Parabacteroides	s__Parabacteroides_goldsteinii	0.0151	0.0151
g__Parabacteroides	s__Parabacteroides_goldsteinii	0.0151	0.0151
g__Parabacteroides	s__Parabacteroides_goldsteinii	0.0151	0.0151
g__Parabacteroides	s__Parabacteroides_goldsteinii	0.0151	0.0151
g__Parabacteroides	s__Parabacteroides_goldsteinii	0.0151	0.0151
g__Parabacteroides	s__Parabacteroides_goldsteinii	0.0151	0.0151
g__Parabacteroides	s__Parabacteroides_goldsteinii	0.0151	0.0151
g__Parabacteroides	s__Parabacteroides_goldsteinii	0.0151	0.0151
g__Parabacteroides	s__Parabacteroides_goldsteinii	0.0151	0.0151
g__Parabacteroides	s__Parabacteroides_goldsteinii	0.0151	0.0151
g__Parabacteroides	s__Parabacteroides_goldsteinii	0.0151	0.0151
g__Parabacteroides	s__Parabacteroides_goldsteinii	0.0151	0.0151
g__Parabacteroides	s__Parabacteroides_goldsteinii	0.0151	0.0151
g__Parabacteroides	s__Parabacteroides_goldsteinii	0.0151	0.0151
g__Parabacteroides	s__Parabacteroides_goldsteinii	0.0151	0.0151
g__Parabacteroides	s__Parabacteroides_goldsteinii	0.0151	0.0151

g__Parabacteroides	s__Parabacteroides_goldsteinii	0.0151	0.0151
g__Parabacteroides	s__Parabacteroides_goldsteinii	0.0151	0.0151
g__Parabacteroides	s__Parabacteroides_goldsteinii	0.0151	0.0151
g__Parabacteroides	s__Parabacteroides_goldsteinii	0.0151	0.0151
g__Parabacteroides	s__Parabacteroides_goldsteinii	0.0151	0.0151
g__Parabacteroides	s__Parabacteroides_goldsteinii	0.0151	0.0151
g__Parabacteroides	s__Parabacteroides_goldsteinii	0.0151	0.0151
g__Parabacteroides	s__Parabacteroides_goldsteinii	0.0151	0.0151
g__Parabacteroides	s__Parabacteroides_goldsteinii	0.0151	0.0151
g__Parabacteroides	s__Parabacteroides_goldsteinii	0.0151	0.0151
g__Parabacteroides	s__Parabacteroides_goldsteinii	0.0151	0.0151
g__Parabacteroides	s__Parabacteroides_goldsteinii	0.0151	0.0151
g__Parabacteroides	s__Parabacteroides_goldsteinii	0.0151	0.0151
g__Parabacteroides	s__Parabacteroides_goldsteinii	0.0151	0.0151
g__Parabacteroides	s__Parabacteroides_goldsteinii	0.0151	0.0151
g__Parabacteroides	s__Parabacteroides_goldsteinii	0.0151	0.0151
g__Parabacteroides	s__Parabacteroides_goldsteinii	0.0151	0.0151
g__Parabacteroides	s__Parabacteroides_goldsteinii	0.0151	0.0151
g__Parabacteroides	s__Parabacteroides_goldsteinii	0.0151	0.0151
g__Parabacteroides	s__Parabacteroides_goldsteinii	0.0151	0.0151
g__Parabacteroides	s__Parabacteroides_goldsteinii	0.0151	0.0151
g__Parabacteroides	s__Parabacteroides_goldsteinii	0.0151	0.0151
g__Parabacteroides	s__Parabacteroides_goldsteinii	0.0151	0.0151
g__Parabacteroides	s__Parabacteroides_goldsteinii	0.0151	0.0151
g__Parabacteroides	s__Parabacteroides_goldsteinii	0.0151	0.0151
g__Parabacteroides	s__Parabacteroides_goldsteinii	0.0151	0.0151
g__Parabacteroides	s__Parabacteroides_goldsteinii	0.0151	0.0151
g__Parabacteroides	s__Parabacteroides_goldsteinii	0.0151	0.0151
g__Parabacteroides	s__Parabacteroides_goldsteinii	0.0151	0.0151
g__Parabacteroides	s__Parabacteroides_goldsteinii	0.0151	0.0151
g__Parabacteroides	s__Parabacteroides_goldsteinii	0.0151	0.0151
g__Parabacteroides	s__Parabacteroides_goldsteinii	0.0151	0.0151
g__Parabacteroides	s__Parabacteroides_johnsonii	0.0151	0.0151
g__Parabacteroides	s__Parabacteroides_johnsonii	0.0151	0.0151
g__Parabacteroides	s__Parabacteroides_johnsonii	0.0151	0.0151
g__Parabacteroides	s__Parabacteroides_johnsonii	0.0151	0.0151
g__Parabacteroides	s__Parabacteroides_johnsonii	0.0151	0.0151
g__Parabacteroides	s__Parabacteroides_merdae	0.0151	0.0151
g__Parabacteroides	s__Parabacteroides_merdae	0.0151	0.0151
g__Parabacteroides	s__Parabacteroides_merdae	0.0151	0.0151
g__Parabacteroides	s__Parabacteroides_merdae	0.0151	0.0151
g__Parabacteroides	s__Parabacteroides_merdae	0.0151	0.0151
g__Paraprevotella	s__Paraprevotella_clara	0.0151	0.0151

g__Paraprevotella	s__Paraprevotella_xylaniphila	0.0151	0.0151
g__Paraprevotella	s__Paraprevotella_xylaniphila	0.0151	0.0151
g__Porphyromonas	s__Porphyromonas_gingivalis	0.0151	0.0151
g__Porphyromonas	s__Porphyromonas_somerae	0.0151	0.0151
g__Prevotella	s__Prevotella_bivia	0.0151	0.0151
g__Prevotella	s__Prevotella_bivia	0.0151	0.0151
g__Prevotella	s__Prevotella_corporis	0.0151	0.0151
g__Prevotella	s__Prevotella_denticola	0.0151	0.0151
g__Prevotella	s__Prevotella_fusca	0.0151	0.0151
g__Prevotella	s__Prevotella_histicola	0.0151	0.0151
g__Prevotella	s__Prevotella_intermedia	0.0151	0.0151
g__Prevotella	s__Prevotella_intermedia	0.0151	0.0151
g__Prevotella	s__Prevotella_intermedia	0.0151	0.0151
g__Prevotella	s__Prevotella_melaninogenica	0.0151	0.0151
g__Prevotella	s__Prevotella_melaninogenica	0.0151	0.0151
g__Prevotella	s__Prevotella_multiformis	0.0151	0.0151
g__Prevotella	s__Prevotella_nigrescens	0.0151	0.0151
g__Prevotella	s__Prevotella_scopos	0.0151	0.0151
g__Pseudobutyrivibrio	s__Pseudobutyrivibrio_xylanivorans	0.0151	0.0151
g__Roseburia	s__Roseburia_hominis	0.0151	0.0151
g__Roseburia	s__Roseburia_hominis	0.0151	0.0151
g__Roseburia	s__Roseburia_intestinalis	0.0151	0.0151
g__Sellimonas	s__Sellimonas_intestinalis	0.0151	0.0151
g__Subdoligranulum	s__Subdoligranulum_variabile	0.0151	0.0151
g__Desulfovibrio	s__Desulfovibrio_desulfuricans	0.0151	0.0151
g__Bacteroides	s__Bacteroides_caccae	0.04	0.04
g__Bacteroides	s__Bacteroides_intestinalis	0.04	0.04
g__Bacteroides	s__Bacteroides_thetaiotaomicron	0.04	0.04
g__Bacteroides	s__Bacteroides_thetaiotaomicron	0.04	0.04
g__Bacteroides	s__Bacteroides_uniformis	0.04	0.04
g__Bacteroides	s__Bacteroides_xylanisolvens	0.04	0.04
g__Catenibacterium	s__Catenibacterium_mitsuokai	0.04	0.04
g__Faecalibaculum	s__Faecalibaculum_rodentium	0.04	0.04
g__Klebsiella	s__Klebsiella_oxytoca	0.04	0.04
g__Lactobacillus	s__Lactobacillus_gasseri	0.04	0.04
g__Lactobacillus	s__Lactobacillus_gasseri	0.04	0.04
g__Lactobacillus	s__Lactobacillus_paragasseri	0.04	0.04
g__Alistipes	s__Alistipes_finegoldii	0.0431	0.0431
g__Alistipes	s__Alistipes_onderdonkii	0.0431	0.0431
g__Bacteroides	s__Bacteroides_caccae	0.0431	0.0431
g__Bacteroides	s__Bacteroides_caccae	0.0431	0.0431
g__Bacteroides	s__Bacteroides_caccae	0.0431	0.0431
g__Bacteroides	s__Bacteroides_caecimuris	0.0431	0.0431

g__Bacteroides	s__Bacteroides_cellulosilyticus	0.0431	0.0431
g__Bacteroides	s__Bacteroides_cellulosilyticus	0.0431	0.0431
g__Bacteroides	s__Bacteroides_faecis	0.0431	0.0431
g__Bacteroides	s__Bacteroides_faecis	0.0431	0.0431
g__Bacteroides	s__Bacteroides_finegoldii	0.0431	0.0431
g__Bacteroides	s__Bacteroides_finegoldii	0.0431	0.0431
g__Bacteroides	s__Bacteroides_fragilis	0.0431	0.0431
g__Bacteroides	s__Bacteroides_fragilis	0.0431	0.0431
g__Bacteroides	s__Bacteroides_fragilis	0.0431	0.0431
g__Bacteroides	s__Bacteroides_fragilis	0.0431	0.0431
g__Bacteroides	s__Bacteroides_fragilis	0.0431	0.0431
g__Bacteroides	s__Bacteroides_fragilis	0.0431	0.0431
g__Bacteroides	s__Bacteroides_helcogenes	0.0431	0.0431
g__Bacteroides	s__Bacteroides_intestinalis	0.0431	0.0431
g__Bacteroides	s__Bacteroides_intestinalis	0.0431	0.0431
g__Bacteroides	s__Bacteroides_intestinalis	0.0431	0.0431
g__Bacteroides	s__Bacteroides_intestinalis	0.0431	0.0431
g__Bacteroides	s__Bacteroides_ovatus	0.0431	0.0431
g__Bacteroides	s__Bacteroides_ovatus	0.0431	0.0431
g__Bacteroides	s__Bacteroides_thetaiotaomicron	0.0431	0.0431
g__Bacteroides	s__Bacteroides_thetaiotaomicron	0.0431	0.0431
g__Bacteroides	s__Bacteroides_thetaiotaomicron	0.0431	0.0431
g__Bacteroides	s__Bacteroides_uniformis	0.0431	0.0431
g__Bacteroides	s__Bacteroides_uniformis	0.0431	0.0431
g__Bacteroides	s__Bacteroides_xylanisolvens	0.0431	0.0431
g__Bacteroides	s__Bacteroides_xylanisolvens	0.0431	0.0431
g__Bacteroides	s__Bacteroides_xylanisolvens	0.0431	0.0431
g__Bacteroides	s__Bacteroides_xylanisolvens	0.0431	0.0431
g__Bacteroides	s__Bacteroides_xylanisolvens	0.0431	0.0431
g__Bacteroides	s__Bacteroides_zoogleoformans	0.0431	0.0431
g__Catenibacterium	s__Catenibacterium_mitsuokai	0.0431	0.0431
g__Catenibacterium	s__Catenibacterium_mitsuokai	0.0431	0.0431
g__Catenibacterium	s__Catenibacterium_mitsuokai	0.0431	0.0431
g__Catenibacterium	s__Catenibacterium_mitsuokai	0.0431	0.0431
g__Catenibacterium	s__Catenibacterium_mitsuokai	0.0431	0.0431
g__Catenibacterium	s__Catenibacterium_mitsuokai	0.0431	0.0431
g__Dorea	s__Dorea_longicatena	0.0431	0.0431
g__Klebsiella	s__Klebsiella_oxytoca	0.0431	0.0431
g__Lactobacillus	s__Lactobacillus_johnsonii	0.0431	0.0431
g__Mucispirillum	s__Mucispirillum_schaedleri	0.0431	0.0431
g__Parabacteroides	s__Parabacteroides_goldsteinii	0.0431	0.0431
g__Parabacteroides	s__Parabacteroides_goldsteinii	0.0431	0.0431
g__Acinetobacter	s__Acinetobacter_johnsonii	0.0442	0.0442

g__Acutalibacter	s__Acutalibacter_muris	0.0442	0.0442
g__Akkermansia	s__Akkermansia_muciniphila	0.0442	0.0442
g__Alistipes	s__Alistipes_finegoldii	0.0442	0.0442
g__Alistipes	s__Alistipes_finegoldii	0.0442	0.0442
g__Alistipes	s__Alistipes_finegoldii	0.0442	0.0442
g__Alistipes	s__Alistipes_onderdonkii	0.0442	0.0442
g__Alistipes	s__Alistipes_onderdonkii	0.0442	0.0442
g__Alistipes	s__Alistipes_onderdonkii	0.0442	0.0442
g__Alistipes	s__Alistipes_onderdonkii	0.0442	0.0442
g__Alistipes	s__Alistipes_shahii	0.0442	0.0442
g__Alistipes	s__Alistipes_shahii	0.0442	0.0442
g__Alistipes	s__Alistipes_shahii	0.0442	0.0442
g__Anaerostipes	s__Anaerostipes_hadrus	0.0442	0.0442
g__Bacteroides	s__Bacteroides_caccae	0.0442	0.0442
g__Bacteroides	s__Bacteroides_caccae	0.0442	0.0442
g__Bacteroides	s__Bacteroides_caccae	0.0442	0.0442
g__Bacteroides	s__Bacteroides_caccae	0.0442	0.0442
g__Bacteroides	s__Bacteroides_caccae	0.0442	0.0442
g__Bacteroides	s__Bacteroides_caccae	0.0442	0.0442
g__Bacteroides	s__Bacteroides_caccae	0.0442	0.0442
g__Bacteroides	s__Bacteroides_caccae	0.0442	0.0442
g__Bacteroides	s__Bacteroides_caccae	0.0442	0.0442
g__Bacteroides	s__Bacteroides_caccae	0.0442	0.0442
g__Bacteroides	s__Bacteroides_caccae	0.0442	0.0442
g__Bacteroides	s__Bacteroides_caccae	0.0442	0.0442
g__Bacteroides	s__Bacteroides_caccae	0.0442	0.0442
g__Bacteroides	s__Bacteroides_caccae	0.0442	0.0442
g__Bacteroides	s__Bacteroides_caccae	0.0442	0.0442
g__Bacteroides	s__Bacteroides_caccae	0.0442	0.0442
g__Bacteroides	s__Bacteroides_caccae	0.0442	0.0442
g__Bacteroides	s__Bacteroides_caccae	0.0442	0.0442
g__Bacteroides	s__Bacteroides_caccae	0.0442	0.0442
g__Bacteroides	s__Bacteroides_caccae	0.0442	0.0442
g__Bacteroides	s__Bacteroides_caccae	0.0442	0.0442
g__Bacteroides	s__Bacteroides_caecimuris	0.0442	0.0442
g__Bacteroides	s__Bacteroides_caecimuris	0.0442	0.0442
g__Bacteroides	s__Bacteroides_caecimuris	0.0442	0.0442
g__Bacteroides	s__Bacteroides_caecimuris	0.0442	0.0442
g__Bacteroides	s__Bacteroides_caecimuris	0.0442	0.0442
g__Bacteroides	s__Bacteroides_caecimuris	0.0442	0.0442
g__Bacteroides	s__Bacteroides_caecimuris	0.0442	0.0442
g__Bacteroides	s__Bacteroides_caecimuris	0.0442	0.0442
g__Bacteroides	s__Bacteroides_caecimuris	0.0442	0.0442

g__Bacteroides	s__Bacteroides_caecimuris	0.0442	0.0442
g__Bacteroides	s__Bacteroides_caecimuris	0.0442	0.0442
g__Bacteroides	s__Bacteroides_caecimuris	0.0442	0.0442
g__Bacteroides	s__Bacteroides_caecimuris	0.0442	0.0442
g__Bacteroides	s__Bacteroides_caecimuris	0.0442	0.0442
g__Bacteroides	s__Bacteroides_caecimuris	0.0442	0.0442
g__Bacteroides	s__Bacteroides_caecimuris	0.0442	0.0442
g__Bacteroides	s__Bacteroides_caecimuris	0.0442	0.0442
g__Bacteroides	s__Bacteroides_caecimuris	0.0442	0.0442
g__Bacteroides	s__Bacteroides_caecimuris	0.0442	0.0442
g__Bacteroides	s__Bacteroides_caecimuris	0.0442	0.0442
g__Bacteroides	s__Bacteroides_caecimuris	0.0442	0.0442
g__Bacteroides	s__Bacteroides_caecimuris	0.0442	0.0442
g__Bacteroides	s__Bacteroides_caecimuris	0.0442	0.0442
g__Bacteroides	s__Bacteroides_cellulosilyticus	0.0442	0.0442
g__Bacteroides	s__Bacteroides_cellulosilyticus	0.0442	0.0442
g__Bacteroides	s__Bacteroides_cellulosilyticus	0.0442	0.0442
g__Bacteroides	s__Bacteroides_cellulosilyticus	0.0442	0.0442
g__Bacteroides	s__Bacteroides_cellulosilyticus	0.0442	0.0442
g__Bacteroides	s__Bacteroides_cellulosilyticus	0.0442	0.0442
g__Bacteroides	s__Bacteroides_cellulosilyticus	0.0442	0.0442
g__Bacteroides	s__Bacteroides_cellulosilyticus	0.0442	0.0442
g__Bacteroides	s__Bacteroides_cellulosilyticus	0.0442	0.0442
g__Bacteroides	s__Bacteroides_cellulosilyticus	0.0442	0.0442
g__Bacteroides	s__Bacteroides_cellulosilyticus	0.0442	0.0442
g__Bacteroides	s__Bacteroides_eggerthii	0.0442	0.0442
g__Bacteroides	s__Bacteroides_eggerthii	0.0442	0.0442
g__Bacteroides	s__Bacteroides_faecis	0.0442	0.0442
g__Bacteroides	s__Bacteroides_faecis	0.0442	0.0442
g__Bacteroides	s__Bacteroides_faecis	0.0442	0.0442
g__Bacteroides	s__Bacteroides_faecis	0.0442	0.0442
g__Bacteroides	s__Bacteroides_faecis	0.0442	0.0442
g__Bacteroides	s__Bacteroides_faecis	0.0442	0.0442
g__Bacteroides	s__Bacteroides_faecis	0.0442	0.0442
g__Bacteroides	s__Bacteroides_faecis	0.0442	0.0442
g__Bacteroides	s__Bacteroides_faecis	0.0442	0.0442
g__Bacteroides	s__Bacteroides_faecis	0.0442	0.0442
g__Bacteroides	s__Bacteroides_faecis	0.0442	0.0442
g__Bacteroides	s__Bacteroides_faecis	0.0442	0.0442
g__Bacteroides	s__Bacteroides_faecis	0.0442	0.0442
g__Bacteroides	s__Bacteroides_faecis	0.0442	0.0442
g__Bacteroides	s__Bacteroides_faecis	0.0442	0.0442
g__Bacteroides	s__Bacteroides_finegoldii	0.0442	0.0442

g__Bacteroides	s__Bacteroides_finegoldii	0.0442	0.0442
g__Bacteroides	s__Bacteroides_finegoldii	0.0442	0.0442
g__Bacteroides	s__Bacteroides_finegoldii	0.0442	0.0442
g__Bacteroides	s__Bacteroides_finegoldii	0.0442	0.0442
g__Bacteroides	s__Bacteroides_finegoldii	0.0442	0.0442
g__Bacteroides	s__Bacteroides_finegoldii	0.0442	0.0442
g__Bacteroides	s__Bacteroides_finegoldii	0.0442	0.0442
g__Bacteroides	s__Bacteroides_finegoldii	0.0442	0.0442
g__Bacteroides	s__Bacteroides_finegoldii	0.0442	0.0442
g__Bacteroides	s__Bacteroides_finegoldii	0.0442	0.0442
g__Bacteroides	s__Bacteroides_finegoldii	0.0442	0.0442
g__Bacteroides	s__Bacteroides_finegoldii	0.0442	0.0442
g__Bacteroides	s__Bacteroides_finegoldii	0.0442	0.0442
g__Bacteroides	s__Bacteroides_finegoldii	0.0442	0.0442
g__Bacteroides	s__Bacteroides_finegoldii	0.0442	0.0442
g__Bacteroides	s__Bacteroides_finegoldii	0.0442	0.0442
g__Bacteroides	s__Bacteroides_finegoldii	0.0442	0.0442
g__Bacteroides	s__Bacteroides_finegoldii	0.0442	0.0442
g__Bacteroides	s__Bacteroides_fragilis	0.0442	0.0442
g__Bacteroides	s__Bacteroides_fragilis	0.0442	0.0442
g__Bacteroides	s__Bacteroides_fragilis	0.0442	0.0442
g__Bacteroides	s__Bacteroides_fragilis	0.0442	0.0442
g__Bacteroides	s__Bacteroides_fragilis	0.0442	0.0442
g__Bacteroides	s__Bacteroides_fragilis	0.0442	0.0442
g__Bacteroides	s__Bacteroides_fragilis	0.0442	0.0442
g__Bacteroides	s__Bacteroides_fragilis	0.0442	0.0442
g__Bacteroides	s__Bacteroides_fragilis	0.0442	0.0442
g__Bacteroides	s__Bacteroides_fragilis	0.0442	0.0442
g__Bacteroides	s__Bacteroides_fragilis	0.0442	0.0442
g__Bacteroides	s__Bacteroides_fragilis	0.0442	0.0442
g__Bacteroides	s__Bacteroides_fragilis	0.0442	0.0442
g__Bacteroides	s__Bacteroides_fragilis	0.0442	0.0442
g__Bacteroides	s__Bacteroides_fragilis	0.0442	0.0442
g__Bacteroides	s__Bacteroides_fragilis	0.0442	0.0442
g__Bacteroides	s__Bacteroides_fragilis	0.0442	0.0442
g__Bacteroides	s__Bacteroides_fragilis	0.0442	0.0442
g__Bacteroides	s__Bacteroides_fragilis	0.0442	0.0442
g__Bacteroides	s__Bacteroides_fragilis	0.0442	0.0442
g__Bacteroides	s__Bacteroides_fragilis	0.0442	0.0442
g__Bacteroides	s__Bacteroides_fragilis	0.0442	0.0442
g__Bacteroides	s__Bacteroides_helcogenes	0.0442	0.0442
g__Bacteroides	s__Bacteroides_helcogenes	0.0442	0.0442
g__Bacteroides	s__Bacteroides_heparinolyticus	0.0442	0.0442

g__Bacteroides	s__Bacteroides_heparinolyticus	0.0442	0.0442
g__Bacteroides	s__Bacteroides_intestinalis	0.0442	0.0442
g__Bacteroides	s__Bacteroides_intestinalis	0.0442	0.0442
g__Bacteroides	s__Bacteroides_intestinalis	0.0442	0.0442
g__Bacteroides	s__Bacteroides_intestinalis	0.0442	0.0442
g__Bacteroides	s__Bacteroides_intestinalis	0.0442	0.0442
g__Bacteroides	s__Bacteroides_intestinalis	0.0442	0.0442
g__Bacteroides	s__Bacteroides_intestinalis	0.0442	0.0442
g__Bacteroides	s__Bacteroides_intestinalis	0.0442	0.0442
g__Bacteroides	s__Bacteroides_intestinalis	0.0442	0.0442
g__Bacteroides	s__Bacteroides_intestinalis	0.0442	0.0442
g__Bacteroides	s__Bacteroides_intestinalis	0.0442	0.0442
g__Bacteroides	s__Bacteroides_intestinalis	0.0442	0.0442
g__Bacteroides	s__Bacteroides_intestinalis	0.0442	0.0442
g__Bacteroides	s__Bacteroides_intestinalis	0.0442	0.0442
g__Bacteroides	s__Bacteroides_intestinalis	0.0442	0.0442
g__Bacteroides	s__Bacteroides_intestinalis	0.0442	0.0442
g__Bacteroides	s__Bacteroides_intestinalis	0.0442	0.0442
g__Bacteroides	s__Bacteroides_intestinalis	0.0442	0.0442
g__Bacteroides	s__Bacteroides_intestinalis	0.0442	0.0442
g__Bacteroides	s__Bacteroides_intestinalis	0.0442	0.0442
g__Bacteroides	s__Bacteroides_intestinalis	0.0442	0.0442
g__Bacteroides	s__Bacteroides_intestinalis	0.0442	0.0442
g__Bacteroides	s__Bacteroides_intestinalis	0.0442	0.0442
g__Bacteroides	s__Bacteroides_intestinalis	0.0442	0.0442
g__Bacteroides	s__Bacteroides_intestinalis	0.0442	0.0442
g__Bacteroides	s__Bacteroides_intestinalis	0.0442	0.0442
g__Bacteroides	s__Bacteroides_intestinalis	0.0442	0.0442
g__Bacteroides	s__Bacteroides_intestinalis	0.0442	0.0442
g__Bacteroides	s__Bacteroides_intestinalis	0.0442	0.0442
g__Bacteroides	s__Bacteroides_intestinalis	0.0442	0.0442
g__Bacteroides	s__Bacteroides_intestinalis	0.0442	0.0442
g__Bacteroides	s__Bacteroides_intestinalis	0.0442	0.0442
g__Bacteroides	s__Bacteroides_nordii	0.0442	0.0442
g__Bacteroides	s__Bacteroides_nordii	0.0442	0.0442
g__Bacteroides	s__Bacteroides_nordii	0.0442	0.0442
g__Bacteroides	s__Bacteroides_ovatus	0.0442	0.0442
g__Bacteroides	s__Bacteroides_ovatus	0.0442	0.0442
g__Bacteroides	s__Bacteroides_ovatus	0.0442	0.0442
g__Bacteroides	s__Bacteroides_ovatus	0.0442	0.0442
g__Bacteroides	s__Bacteroides_ovatus	0.0442	0.0442
g__Bacteroides	s__Bacteroides_ovatus	0.0442	0.0442
g__Bacteroides	s__Bacteroides_ovatus	0.0442	0.0442

g__Bacteroides	s__Bacteroides_ovatus	0.0442	0.0442
g__Bacteroides	s__Bacteroides_ovatus	0.0442	0.0442
g__Bacteroides	s__Bacteroides_ovatus	0.0442	0.0442
g__Bacteroides	s__Bacteroides_ovatus	0.0442	0.0442
g__Bacteroides	s__Bacteroides_salyersiae	0.0442	0.0442
g__Bacteroides	s__Bacteroides_salyersiae	0.0442	0.0442
g__Bacteroides	s__Bacteroides_salyersiae	0.0442	0.0442
g__Bacteroides	s__Bacteroides_salyersiae	0.0442	0.0442
g__Bacteroides	s__Bacteroides_salyersiae	0.0442	0.0442
g__Bacteroides	s__Bacteroides_salyersiae	0.0442	0.0442
g__Bacteroides	s__Bacteroides_salyersiae	0.0442	0.0442
g__Bacteroides	s__Bacteroides_stercoris	0.0442	0.0442
g__Bacteroides	s__Bacteroides_stercoris	0.0442	0.0442
g__Bacteroides	s__Bacteroides_thetaiotaomicron	0.0442	0.0442
g__Bacteroides	s__Bacteroides_thetaiotaomicron	0.0442	0.0442
g__Bacteroides	s__Bacteroides_thetaiotaomicron	0.0442	0.0442
g__Bacteroides	s__Bacteroides_thetaiotaomicron	0.0442	0.0442
g__Bacteroides	s__Bacteroides_thetaiotaomicron	0.0442	0.0442
g__Bacteroides	s__Bacteroides_thetaiotaomicron	0.0442	0.0442
g__Bacteroides	s__Bacteroides_thetaiotaomicron	0.0442	0.0442
g__Bacteroides	s__Bacteroides_thetaiotaomicron	0.0442	0.0442
g__Bacteroides	s__Bacteroides_thetaiotaomicron	0.0442	0.0442
g__Bacteroides	s__Bacteroides_thetaiotaomicron	0.0442	0.0442
g__Bacteroides	s__Bacteroides_thetaiotaomicron	0.0442	0.0442
g__Bacteroides	s__Bacteroides_thetaiotaomicron	0.0442	0.0442
g__Bacteroides	s__Bacteroides_thetaiotaomicron	0.0442	0.0442
g__Bacteroides	s__Bacteroides_thetaiotaomicron	0.0442	0.0442
g__Bacteroides	s__Bacteroides_thetaiotaomicron	0.0442	0.0442
g__Bacteroides	s__Bacteroides_thetaiotaomicron	0.0442	0.0442
g__Bacteroides	s__Bacteroides_thetaiotaomicron	0.0442	0.0442
g__Bacteroides	s__Bacteroides_uniformis	0.0442	0.0442
g__Bacteroides	s__Bacteroides_uniformis	0.0442	0.0442
g__Bacteroides	s__Bacteroides_uniformis	0.0442	0.0442
g__Bacteroides	s__Bacteroides_uniformis	0.0442	0.0442
g__Bacteroides	s__Bacteroides_uniformis	0.0442	0.0442
g__Bacteroides	s__Bacteroides_uniformis	0.0442	0.0442
g__Bacteroides	s__Bacteroides_uniformis	0.0442	0.0442
g__Bacteroides	s__Bacteroides_uniformis	0.0442	0.0442
g__Bacteroides	s__Bacteroides_uniformis	0.0442	0.0442
g__Bacteroides	s__Bacteroides_uniformis	0.0442	0.0442
g__Bacteroides	s__Bacteroides_uniformis	0.0442	0.0442
g__Bacteroides	s__Bacteroides_uniformis	0.0442	0.0442
g__Bacteroides	s__Bacteroides_uniformis	0.0442	0.0442

g__Bacteroides	s__Bacteroides_uniformis	0.0442	0.0442
g__Bacteroides	s__Bacteroides_uniformis	0.0442	0.0442
g__Bacteroides	s__Bacteroides_uniformis	0.0442	0.0442
g__Bacteroides	s__Bacteroides_uniformis	0.0442	0.0442
g__Bacteroides	s__Bacteroides_uniformis	0.0442	0.0442
g__Bacteroides	s__Bacteroides_uniformis	0.0442	0.0442
g__Bacteroides	s__Bacteroides_uniformis	0.0442	0.0442
g__Bacteroides	s__Bacteroides_uniformis	0.0442	0.0442
g__Bacteroides	s__Bacteroides_uniformis	0.0442	0.0442
g__Bacteroides	s__Bacteroides_uniformis	0.0442	0.0442
g__Bacteroides	s__Bacteroides_uniformis	0.0442	0.0442
g__Bacteroides	s__Bacteroides_uniformis	0.0442	0.0442
g__Bacteroides	s__Bacteroides_uniformis	0.0442	0.0442
g__Bacteroides	s__Bacteroides_uniformis	0.0442	0.0442
g__Bacteroides	s__Bacteroides_xylanisolvens	0.0442	0.0442
g__Bacteroides	s__Bacteroides_xylanisolvens	0.0442	0.0442
g__Bacteroides	s__Bacteroides_xylanisolvens	0.0442	0.0442
g__Bacteroides	s__Bacteroides_xylanisolvens	0.0442	0.0442
g__Bacteroides	s__Bacteroides_xylanisolvens	0.0442	0.0442
g__Bacteroides	s__Bacteroides_xylanisolvens	0.0442	0.0442
g__Bacteroides	s__Bacteroides_xylanisolvens	0.0442	0.0442
g__Bacteroides	s__Bacteroides_xylanisolvens	0.0442	0.0442
g__Bacteroides	s__Bacteroides_xylanisolvens	0.0442	0.0442
g__Bacteroides	s__Bacteroides_xylanisolvens	0.0442	0.0442
g__Bacteroides	s__Bacteroides_xylanisolvens	0.0442	0.0442
g__Bacteroides	s__Bacteroides_xylanisolvens	0.0442	0.0442
g__Bacteroides	s__Bacteroides_xylanisolvens	0.0442	0.0442
g__Bacteroides	s__Bacteroides_xylanisolvens	0.0442	0.0442
g__Bacteroides	s__Bacteroides_xylanisolvens	0.0442	0.0442
g__Bacteroides	s__Bacteroides_xylanisolvens	0.0442	0.0442
g__Bacteroides	s__Bacteroides_xylanisolvens	0.0442	0.0442
g__Bacteroides	s__Bacteroides_xylanisolvens	0.0442	0.0442
g__Bacteroides	s__Bacteroides_xylanisolvens	0.0442	0.0442
g__Bacteroides	s__Bacteroides_xylanisolvens	0.0442	0.0442
g__Bacteroides	s__Bacteroides_xylanisolvens	0.0442	0.0442
g__Bacteroides	s__Bacteroides_xylanisolvens	0.0442	0.0442
g__Bacteroides	s__Bacteroides_xylanisolvens	0.0442	0.0442
g__Bacteroides	s__Bacteroides_xylanisolvens	0.0442	0.0442
g__Bacteroides	s__Bacteroides_xylanisolvens	0.0442	0.0442
g__Bacteroides	s__Bacteroides_xylanisolvens	0.0442	0.0442
g__Blautia	s__Blautia_coccoides	0.0442	0.0442
g__Blautia	s__Blautia_hansenii	0.0442	0.0442
g__Blautia	s__Blautia_wexlerae	0.0442	0.0442

g__Blautia	s__Blautia_wexlerae	0.0442	0.0442
g__Blautia	s__Blautia_wexlerae	0.0442	0.0442
g__Catenibacterium	s__Catenibacterium_mitsuokai	0.0442	0.0442
g__Catenibacterium	s__Catenibacterium_mitsuokai	0.0442	0.0442
g__Catenibacterium	s__Catenibacterium_mitsuokai	0.0442	0.0442
g__Catenibacterium	s__Catenibacterium_mitsuokai	0.0442	0.0442
g__Catenibacterium	s__Catenibacterium_mitsuokai	0.0442	0.0442
g__Catenibacterium	s__Catenibacterium_mitsuokai	0.0442	0.0442
g__Catenibacterium	s__Catenibacterium_mitsuokai	0.0442	0.0442
g__Catenibacterium	s__Catenibacterium_mitsuokai	0.0442	0.0442
g__Catenibacterium	s__Catenibacterium_mitsuokai	0.0442	0.0442
g__Catenibacterium	s__Catenibacterium_mitsuokai	0.0442	0.0442
g__Catenibacterium	s__Catenibacterium_mitsuokai	0.0442	0.0442
g__Catenibacterium	s__Catenibacterium_mitsuokai	0.0442	0.0442
g__Catenibacterium	s__Catenibacterium_mitsuokai	0.0442	0.0442
g__Catenibacterium	s__Catenibacterium_mitsuokai	0.0442	0.0442
g__Catenibacterium	s__Catenibacterium_mitsuokai	0.0442	0.0442
g__Catenibacterium	s__Catenibacterium_mitsuokai	0.0442	0.0442
g__Clostridioides	s__Clostridioides_difficile	0.0442	0.0442
g__Clostridioides	s__Clostridioides_difficile	0.0442	0.0442
g__Coprococcus	s__Coprococcus_comes	0.0442	0.0442
g__Coprococcus	s__Coprococcus_comes	0.0442	0.0442
g__Coprococcus	s__Coprococcus_eutactus	0.0442	0.0442
g__Dorea	s__Dorea_formicigenerans	0.0442	0.0442
g__Dorea	s__Dorea_longicatena	0.0442	0.0442
g__Dorea	s__Dorea_longicatena	0.0442	0.0442
g__Faecalibacterium	s__Faecalibacterium_prausnitzii	0.0442	0.0442
g__Faecalibaculum	s__Faecalibaculum_rodentium	0.0442	0.0442
g__Faecalibaculum	s__Faecalibaculum_rodentium	0.0442	0.0442
g__Faecalibaculum	s__Faecalibaculum_rodentium	0.0442	0.0442
g__Faecalibaculum	s__Faecalibaculum_rodentium	0.0442	0.0442
g__Faecalibaculum	s__Faecalibaculum_rodentium	0.0442	0.0442
g__Faecalibaculum	s__Faecalibaculum_rodentium	0.0442	0.0442
g__Faecalibaculum	s__Faecalibaculum_rodentium	0.0442	0.0442
g__Faecalibaculum	s__Faecalibaculum_rodentium	0.0442	0.0442
g__Faecalibaculum	s__Faecalibaculum_rodentium	0.0442	0.0442
g__Faecalibaculum	s__Faecalibaculum_rodentium	0.0442	0.0442
g__Flavonifractor	s__Flavonifractor_plautii	0.0442	0.0442
g__Flavonifractor	s__Flavonifractor_plautii	0.0442	0.0442
g__Klebsiella	s__Klebsiella_oxytoca	0.0442	0.0442
g__Klebsiella	s__Klebsiella_oxytoca	0.0442	0.0442
g__Klebsiella	s__Klebsiella_oxytoca	0.0442	0.0442
g__Klebsiella	s__Klebsiella_oxytoca	0.0442	0.0442

g__Lactobacillus	s__Lactobacillus_gasseri	0.0442	0.0442
g__Lactobacillus	s__Lactobacillus_gasseri	0.0442	0.0442
g__Lactobacillus	s__Lactobacillus_gasseri	0.0442	0.0442
g__Lactobacillus	s__Lactobacillus_johnsonii	0.0442	0.0442
g__Lactobacillus	s__Lactobacillus_johnsonii	0.0442	0.0442
g__Lactobacillus	s__Lactobacillus_johnsonii	0.0442	0.0442
g__Lactobacillus	s__Lactobacillus_johnsonii	0.0442	0.0442
g__Lactobacillus	s__Lactobacillus_johnsonii	0.0442	0.0442
g__Lactobacillus	s__Lactobacillus_johnsonii	0.0442	0.0442
g__Lactobacillus	s__Lactobacillus_johnsonii	0.0442	0.0442
g__Lactobacillus	s__Lactobacillus_johnsonii	0.0442	0.0442
g__Lactobacillus	s__Lactobacillus_johnsonii	0.0442	0.0442
g__Lactobacillus	s__Lactobacillus_johnsonii	0.0442	0.0442
g__Lactobacillus	s__Lactobacillus_johnsonii	0.0442	0.0442
g__Lactobacillus	s__Lactobacillus_johnsonii	0.0442	0.0442
g__Lactobacillus	s__Lactobacillus_johnsonii	0.0442	0.0442
g__Lactobacillus	s__Lactobacillus_johnsonii	0.0442	0.0442
g__Lactobacillus	s__Lactobacillus_johnsonii	0.0442	0.0442
g__Lactobacillus	s__Lactobacillus_johnsonii	0.0442	0.0442
g__Lactobacillus	s__Lactobacillus_johnsonii	0.0442	0.0442
g__Lactobacillus	s__Lactobacillus_johnsonii	0.0442	0.0442
g__Lactobacillus	s__Lactobacillus_johnsonii	0.0442	0.0442
g__Lactobacillus	s__Lactobacillus_johnsonii	0.0442	0.0442
g__Lactobacillus	s__Lactobacillus_johnsonii	0.0442	0.0442
g__Lactobacillus	s__Lactobacillus_johnsonii	0.0442	0.0442
g__Lactobacillus	s__Lactobacillus_johnsonii	0.0442	0.0442
g__Lactobacillus	s__Lactobacillus_johnsonii	0.0442	0.0442
g__Lactobacillus	s__Lactobacillus_johnsonii	0.0442	0.0442
g__Lactobacillus	s__Lactobacillus_johnsonii	0.0442	0.0442
g__Lactobacillus	s__Lactobacillus_johnsonii	0.0442	0.0442
g__Lactobacillus	s__Lactobacillus_johnsonii	0.0442	0.0442
g__Lactobacillus	s__Lactobacillus_johnsonii	0.0442	0.0442
g__Lactobacillus	s__Lactobacillus_johnsonii	0.0442	0.0442
g__Lactobacillus	s__Lactobacillus_johnsonii	0.0442	0.0442
g__Lactobacillus	s__Lactobacillus_johnsonii	0.0442	0.0442
g__Lactobacillus	s__Lactobacillus_johnsonii	0.0442	0.0442
g__Lactobacillus	s__Lactobacillus_johnsonii	0.0442	0.0442
g__Lactobacillus	s__Lactobacillus_johnsonii	0.0442	0.0442
g__Lactobacillus	s__Lactobacillus_johnsonii	0.0442	0.0442
g__Lactobacillus	s__Lactobacillus_johnsonii	0.0442	0.0442
g__Lactobacillus	s__Lactobacillus_johnsonii	0.0442	0.0442
g__Lactobacillus	s__Lactobacillus_johnsonii	0.0442	0.0442
g__Lactobacillus	s__Lactobacillus_johnsonii	0.0442	0.0442

g__Lactobacillus	s__Lactobacillus_johnsonii	0.0442	0.0442
g__Lactobacillus	s__Lactobacillus_johnsonii	0.0442	0.0442
g__Lactobacillus	s__Lactobacillus_johnsonii	0.0442	0.0442
g__Lactobacillus	s__Lactobacillus_johnsonii	0.0442	0.0442
g__Lactobacillus	s__Lactobacillus_paragasseri	0.0442	0.0442
g__Lactobacillus	s__Lactobacillus_taiwanensis	0.0442	0.0442
g__Lactobacillus	s__Lactobacillus_taiwanensis	0.0442	0.0442
g__Lactobacillus	s__Lactobacillus_taiwanensis	0.0442	0.0442
g__Lactobacillus	s__Lactobacillus_taiwanensis	0.0442	0.0442
g__Lactobacillus	s__Lactobacillus_taiwanensis	0.0442	0.0442
g__Lactobacillus	s__Lactobacillus_taiwanensis	0.0442	0.0442
g__Lactobacillus	s__Lactobacillus_taiwanensis	0.0442	0.0442
g__Lawsonibacter	s__Lawsonibacter_asaccharolyticus	0.0442	0.0442
g__Mucispirillum	s__Mucispirillum_schaedleri	0.0442	0.0442
g__Mucispirillum	s__Mucispirillum_schaedleri	0.0442	0.0442
g__Mucispirillum	s__Mucispirillum_schaedleri	0.0442	0.0442
g__Mucispirillum	s__Mucispirillum_schaedleri	0.0442	0.0442
g__Mucispirillum	s__Mucispirillum_schaedleri	0.0442	0.0442
g__Mucispirillum	s__Mucispirillum_schaedleri	0.0442	0.0442
g__Mucispirillum	s__Mucispirillum_schaedleri	0.0442	0.0442
g__Mucispirillum	s__Mucispirillum_schaedleri	0.0442	0.0442
g__Mucispirillum	s__Mucispirillum_schaedleri	0.0442	0.0442
g__Mucispirillum	s__Mucispirillum_schaedleri	0.0442	0.0442
g__Mucispirillum	s__Mucispirillum_schaedleri	0.0442	0.0442
g__Mucispirillum	s__Mucispirillum_schaedleri	0.0442	0.0442
g__Mucispirillum	s__Mucispirillum_schaedleri	0.0442	0.0442
g__Mucispirillum	s__Mucispirillum_schaedleri	0.0442	0.0442
g__Mucispirillum	s__Mucispirillum_schaedleri	0.0442	0.0442
g__Mucispirillum	s__Mucispirillum_schaedleri	0.0442	0.0442
g__Mucispirillum	s__Mucispirillum_schaedleri	0.0442	0.0442
g__Parabacteroides	s__Parabacteroides_goldsteinii	0.0442	0.0442
g__Parabacteroides	s__Parabacteroides_goldsteinii	0.0442	0.0442
g__Parabacteroides	s__Parabacteroides_goldsteinii	0.0442	0.0442
g__Parabacteroides	s__Parabacteroides_goldsteinii	0.0442	0.0442
g__Parabacteroides	s__Parabacteroides_goldsteinii	0.0442	0.0442
g__Parabacteroides	s__Parabacteroides_goldsteinii	0.0442	0.0442
g__Parabacteroides	s__Parabacteroides_goldsteinii	0.0442	0.0442
g__Parabacteroides	s__Parabacteroides_goldsteinii	0.0442	0.0442
g__Parabacteroides	s__Parabacteroides_goldsteinii	0.0442	0.0442
g__Parabacteroides	s__Parabacteroides_goldsteinii	0.0442	0.0442
g__Parabacteroides	s__Parabacteroides_goldsteinii	0.0442	0.0442
g__Parabacteroides	s__Parabacteroides_goldsteinii	0.0442	0.0442
g__Parabacteroides	s__Parabacteroides_goldsteinii	0.0442	0.0442

g__Parabacteroides	s__Parabacteroides_goldsteinii	0.0442	0.0442
g__Parabacteroides	s__Parabacteroides_goldsteinii	0.0442	0.0442
g__Parabacteroides	s__Parabacteroides_goldsteinii	0.0442	0.0442
g__Parabacteroides	s__Parabacteroides_goldsteinii	0.0442	0.0442
g__Parabacteroides	s__Parabacteroides_goldsteinii	0.0442	0.0442
g__Parabacteroides	s__Parabacteroides_goldsteinii	0.0442	0.0442
g__Parabacteroides	s__Parabacteroides_goldsteinii	0.0442	0.0442
g__Parabacteroides	s__Parabacteroides_goldsteinii	0.0442	0.0442
g__Parabacteroides	s__Parabacteroides_goldsteinii	0.0442	0.0442
g__Parabacteroides	s__Parabacteroides_goldsteinii	0.0442	0.0442
g__Parabacteroides	s__Parabacteroides_goldsteinii	0.0442	0.0442
g__Parabacteroides	s__Parabacteroides_goldsteinii	0.0442	0.0442
g__Parabacteroides	s__Parabacteroides_goldsteinii	0.0442	0.0442
g__Parabacteroides	s__Parabacteroides_goldsteinii	0.0442	0.0442
g__Parabacteroides	s__Parabacteroides_goldsteinii	0.0442	0.0442
g__Parabacteroides	s__Parabacteroides_goldsteinii	0.0442	0.0442
g__Parabacteroides	s__Parabacteroides_goldsteinii	0.0442	0.0442
g__Parabacteroides	s__Parabacteroides_goldsteinii	0.0442	0.0442
g__Parabacteroides	s__Parabacteroides_goldsteinii	0.0442	0.0442
g__Parabacteroides	s__Parabacteroides_goldsteinii	0.0442	0.0442
g__Parabacteroides	s__Parabacteroides_goldsteinii	0.0442	0.0442
g__Parabacteroides	s__Parabacteroides_goldsteinii	0.0442	0.0442
g__Parabacteroides	s__Parabacteroides_goldsteinii	0.0442	0.0442
g__Parabacteroides	s__Parabacteroides_goldsteinii	0.0442	0.0442
g__Parabacteroides	s__Parabacteroides_goldsteinii	0.0442	0.0442
g__Parabacteroides	s__Parabacteroides_goldsteinii	0.0442	0.0442
g__Parabacteroides	s__Parabacteroides_johnsonii	0.0442	0.0442
g__Parabacteroides	s__Parabacteroides_johnsonii	0.0442	0.0442
g__Parabacteroides	s__Parabacteroides_johnsonii	0.0442	0.0442
g__Pediococcus	s__Pediococcus_acidilactici	0.0442	0.0442
g__Pediococcus	s__Pediococcus_acidilactici	0.0442	0.0442
g__Pediococcus	s__Pediococcus_acidilactici	0.0442	0.0442
g__Prevotella	s__Prevotella_dentalis	0.0442	0.0442
g__Prevotella	s__Prevotella_denticola	0.0442	0.0442
g__Roseburia	s__Roseburia_hominis	0.0442	0.0442
g__Sellimonas	s__Sellimonas_intestinalis	0.0442	0.0442
g__Acinetobacter	s__Acinetobacter_johnsonii	0.0442	0.0442
g__Acutalibacter	s__Acutalibacter_muris	0.0151	0.0151
g__Acutalibacter	s__Acutalibacter_muris	0.0442	0.0442
g__Adlercreutzia	s__Adlercreutzia_equolifaciens	0.0151	0.0151
g__Adlercreutzia	s__Adlercreutzia_equolifaciens	0.0151	0.0151
g__Adlercreutzia	s__Adlercreutzia_equolifaciens	0.0151	0.0151
g__Akkermansia	s__Akkermansia_muciniphila	0.0442	0.0442

g__Akkermansia	s__Akkermansia_muciniphila	0.0151	0.0151
g__Alistipes	s__Alistipes_finegoldii	0.0151	0.0151
g__Alistipes	s__Alistipes_finegoldii	0.0151	0.0151
g__Alistipes	s__Alistipes_finegoldii	0.0151	0.0151
g__Alistipes	s__Alistipes_finegoldii	0.0431	0.0431
g__Alistipes	s__Alistipes_finegoldii	0.0151	0.0151
g__Alistipes	s__Alistipes_finegoldii	0.0442	0.0442
g__Alistipes	s__Alistipes_finegoldii	0.0151	0.0151
g__Alistipes	s__Alistipes_finegoldii	0.0151	0.0151
g__Alistipes	s__Alistipes_finegoldii	0.0442	0.0442
g__Alistipes	s__Alistipes_finegoldii	0.0151	0.0151
g__Alistipes	s__Alistipes_finegoldii	0.0151	0.0151
g__Alistipes	s__Alistipes_finegoldii	0.0151	0.0151
g__Alistipes	s__Alistipes_finegoldii	0.0151	0.0151
g__Alistipes	s__Alistipes_finegoldii	0.0151	0.0151
g__Alistipes	s__Alistipes_finegoldii	0.0442	0.0442
g__Alistipes	s__Alistipes_finegoldii	0.0151	0.0151
g__Alistipes	s__Alistipes_onderdonkii	0.0151	0.0151
g__Alistipes	s__Alistipes_onderdonkii	0.0151	0.0151
g__Alistipes	s__Alistipes_onderdonkii	0.0151	0.0151
g__Alistipes	s__Alistipes_onderdonkii	0.0431	0.0431
g__Alistipes	s__Alistipes_onderdonkii	0.0151	0.0151
g__Alistipes	s__Alistipes_onderdonkii	0.0151	0.0151
g__Alistipes	s__Alistipes_onderdonkii	0.0442	0.0442
g__Alistipes	s__Alistipes_onderdonkii	0.0151	0.0151
g__Alistipes	s__Alistipes_onderdonkii	0.0442	0.0442
g__Alistipes	s__Alistipes_onderdonkii	0.0442	0.0442
g__Alistipes	s__Alistipes_onderdonkii	0.0151	0.0151
g__Alistipes	s__Alistipes_onderdonkii	0.0151	0.0151
g__Alistipes	s__Alistipes_onderdonkii	0.0151	0.0151
g__Alistipes	s__Alistipes_onderdonkii	0.0151	0.0151
g__Alistipes	s__Alistipes_onderdonkii	0.0151	0.0151
g__Alistipes	s__Alistipes_onderdonkii	0.0442	0.0442
g__Alistipes	s__Alistipes_onderdonkii	0.0151	0.0151
g__Alistipes	s__Alistipes_shahii	0.0151	0.0151
g__Alistipes	s__Alistipes_shahii	0.0151	0.0151
g__Alistipes	s__Alistipes_shahii	0.0151	0.0151
g__Alistipes	s__Alistipes_shahii	0.0151	0.0151
g__Alistipes	s__Alistipes_shahii	0.0151	0.0151
g__Alistipes	s__Alistipes_shahii	0.0442	0.0442
g__Alistipes	s__Alistipes_shahii	0.0151	0.0151
g__Alistipes	s__Alistipes_shahii	0.0151	0.0151
g__Alistipes	s__Alistipes_shahii	0.0442	0.0442

g__Alistipes	s__Alistipes_shahii	0.0151	0.0151
g__Alistipes	s__Alistipes_shahii	0.0151	0.0151
g__Alistipes	s__Alistipes_shahii	0.0151	0.0151
g__Alistipes	s__Alistipes_shahii	0.0151	0.0151
g__Alistipes	s__Alistipes_shahii	0.0151	0.0151
g__Alistipes	s__Alistipes_shahii	0.0442	0.0442
g__Alistipes	s__Alistipes_shahii	0.0151	0.0151
g__Anaerostipes	s__Anaerostipes_hadrus	0.0442	0.0442
g__Bacillus	s__Bacillus_subtilis_group	0.0108	0.0108
g__Bacteroides	s__Bacteroides_caccae	0.0151	0.0151
g__Bacteroides	s__Bacteroides_caccae	0.0151	0.0151
g__Bacteroides	s__Bacteroides_caccae	0.0151	0.0151
g__Bacteroides	s__Bacteroides_caccae	0.0151	0.0151
g__Bacteroides	s__Bacteroides_caccae	0.0151	0.0151
g__Bacteroides	s__Bacteroides_caccae	0.0442	0.0442
g__Bacteroides	s__Bacteroides_caccae	0.0151	0.0151
g__Bacteroides	s__Bacteroides_caccae	0.0151	0.0151
g__Bacteroides	s__Bacteroides_caccae	0.0151	0.0151
g__Bacteroides	s__Bacteroides_caccae	0.0179	0.0179
g__Bacteroides	s__Bacteroides_caccae	0.0151	0.0151
g__Bacteroides	s__Bacteroides_caccae	0.0151	0.0151
g__Bacteroides	s__Bacteroides_caccae	0.0151	0.0151
g__Bacteroides	s__Bacteroides_caccae	0.0151	0.0151
g__Bacteroides	s__Bacteroides_caccae	0.0442	0.0442
g__Bacteroides	s__Bacteroides_caccae	0.0442	0.0442
g__Bacteroides	s__Bacteroides_caccae	0.0442	0.0442
g__Bacteroides	s__Bacteroides_caccae	0.0151	0.0151
g__Bacteroides	s__Bacteroides_caccae	0.0151	0.0151
g__Bacteroides	s__Bacteroides_caccae	0.0442	0.0442
g__Bacteroides	s__Bacteroides_caccae	0.0442	0.0442
g__Bacteroides	s__Bacteroides_caccae	0.0151	0.0151
g__Bacteroides	s__Bacteroides_caccae	0.0146	0.0146
g__Bacteroides	s__Bacteroides_caccae	0.0151	0.0151
g__Bacteroides	s__Bacteroides_caccae	0.0151	0.0151
g__Bacteroides	s__Bacteroides_caccae	0.0151	0.0151
g__Bacteroides	s__Bacteroides_caccae	0.0151	0.0151
g__Bacteroides	s__Bacteroides_caccae	0.0151	0.0151
g__Bacteroides	s__Bacteroides_caccae	0.0151	0.0151
g__Bacteroides	s__Bacteroides_caccae	0.0151	0.0151
g__Bacteroides	s__Bacteroides_caccae	0.0146	0.0146
g__Bacteroides	s__Bacteroides_caccae	0.0151	0.0151
g__Bacteroides	s__Bacteroides_caccae	0.0151	0.0151
g__Bacteroides	s__Bacteroides_caccae	0.0151	0.0151

g__Bacteroides	s__Bacteroides_caccae	0.0151	0.0151
g__Bacteroides	s__Bacteroides_caccae	0.0151	0.0151
g__Bacteroides	s__Bacteroides_caccae	0.0151	0.0151
g__Bacteroides	s__Bacteroides_caccae	0.0151	0.0151
g__Bacteroides	s__Bacteroides_caccae	0.0151	0.0151
g__Bacteroides	s__Bacteroides_caccae	0.0442	0.0442
g__Bacteroides	s__Bacteroides_caccae	0.0151	0.0151
g__Bacteroides	s__Bacteroides_caccae	0.0151	0.0151
g__Bacteroides	s__Bacteroides_caccae	0.0151	0.0151
g__Bacteroides	s__Bacteroides_caccae	0.04	0.04
g__Bacteroides	s__Bacteroides_caccae	0.0151	0.0151
g__Bacteroides	s__Bacteroides_caccae	0.0151	0.0151
g__Bacteroides	s__Bacteroides_caccae	0.0151	0.0151
g__Bacteroides	s__Bacteroides_caccae	0.0442	0.0442
g__Bacteroides	s__Bacteroides_caccae	0.0151	0.0151
g__Bacteroides	s__Bacteroides_caccae	0.0151	0.0151
g__Bacteroides	s__Bacteroides_caccae	0.0151	0.0151
g__Bacteroides	s__Bacteroides_caccae	0.0151	0.0151
g__Bacteroides	s__Bacteroides_caccae	0.0151	0.0151
g__Bacteroides	s__Bacteroides_caccae	0.0442	0.0442
g__Bacteroides	s__Bacteroides_caccae	0.0151	0.0151
g__Bacteroides	s__Bacteroides_caccae	0.0442	0.0442
g__Bacteroides	s__Bacteroides_caccae	0.0151	0.0151
g__Bacteroides	s__Bacteroides_caccae	0.0442	0.0442
g__Bacteroides	s__Bacteroides_caccae	0.0151	0.0151
g__Bacteroides	s__Bacteroides_caccae	0.0151	0.0151
g__Bacteroides	s__Bacteroides_caccae	0.0151	0.0151
g__Bacteroides	s__Bacteroides_caccae	0.0151	0.0151
g__Bacteroides	s__Bacteroides_caccae	0.0151	0.0151
g__Bacteroides	s__Bacteroides_caccae	0.0151	0.0151
g__Bacteroides	s__Bacteroides_caccae	0.0151	0.0151
g__Bacteroides	s__Bacteroides_caccae	0.0151	0.0151
g__Bacteroides	s__Bacteroides_caccae	0.0151	0.0151
g__Bacteroides	s__Bacteroides_caccae	0.0151	0.0151
g__Bacteroides	s__Bacteroides_caccae	0.0151	0.0151
g__Bacteroides	s__Bacteroides_caccae	0.0151	0.0151
g__Bacteroides	s__Bacteroides_caccae	0.0151	0.0151
g__Bacteroides	s__Bacteroides_caccae	0.0151	0.0151
g__Bacteroides	s__Bacteroides_caccae	0.0151	0.0151
g__Bacteroides	s__Bacteroides_caccae	0.0151	0.0151
g__Bacteroides	s__Bacteroides_caccae	0.0151	0.0151
g__Bacteroides	s__Bacteroides_caccae	0.0151	0.0151
g__Bacteroides	s__Bacteroides_caccae	0.0151	0.0151

g__Bacteroides	s__Bacteroides_caccae	0.0151	0.0151
g__Bacteroides	s__Bacteroides_caccae	0.0442	0.0442
g__Bacteroides	s__Bacteroides_caccae	0.0151	0.0151
g__Bacteroides	s__Bacteroides_caccae	0.0442	0.0442
g__Bacteroides	s__Bacteroides_caccae	0.0151	0.0151
g__Bacteroides	s__Bacteroides_caccae	0.0151	0.0151
g__Bacteroides	s__Bacteroides_caccae	0.0442	0.0442
g__Bacteroides	s__Bacteroides_caccae	0.0151	0.0151
g__Bacteroides	s__Bacteroides_caccae	0.0151	0.0151
g__Bacteroides	s__Bacteroides_caccae	0.0151	0.0151
g__Bacteroides	s__Bacteroides_caccae	0.0151	0.0151
g__Bacteroides	s__Bacteroides_caccae	0.0151	0.0151
g__Bacteroides	s__Bacteroides_caccae	0.0151	0.0151
g__Bacteroides	s__Bacteroides_caccae	0.0151	0.0151
g__Bacteroides	s__Bacteroides_caccae	0.0151	0.0151
g__Bacteroides	s__Bacteroides_caccae	0.0151	0.0151
g__Bacteroides	s__Bacteroides_caccae	0.0431	0.0431
g__Bacteroides	s__Bacteroides_caccae	0.0146	0.0146
g__Bacteroides	s__Bacteroides_caccae	0.0151	0.0151
g__Bacteroides	s__Bacteroides_caccae	0.0151	0.0151
g__Bacteroides	s__Bacteroides_caccae	0.0151	0.0151
g__Bacteroides	s__Bacteroides_caccae	0.0151	0.0151
g__Bacteroides	s__Bacteroides_caccae	0.0151	0.0151
g__Bacteroides	s__Bacteroides_caccae	0.0151	0.0151
g__Bacteroides	s__Bacteroides_caccae	0.0151	0.0151
g__Bacteroides	s__Bacteroides_caccae	0.0146	0.0146
g__Bacteroides	s__Bacteroides_caccae	0.0151	0.0151
g__Bacteroides	s__Bacteroides_caccae	0.0442	0.0442
g__Bacteroides	s__Bacteroides_caccae	0.0151	0.0151
g__Bacteroides	s__Bacteroides_caccae	0.0151	0.0151
g__Bacteroides	s__Bacteroides_caccae	0.0151	0.0151
g__Bacteroides	s__Bacteroides_caccae	0.0151	0.0151
g__Bacteroides	s__Bacteroides_caccae	0.0151	0.0151
g__Bacteroides	s__Bacteroides_caccae	0.0151	0.0151
g__Bacteroides	s__Bacteroides_caccae	0.0151	0.0151
g__Bacteroides	s__Bacteroides_caccae	0.0151	0.0151
g__Bacteroides	s__Bacteroides_caccae	0.0129	0.0129
g__Bacteroides	s__Bacteroides_caccae	0.0442	0.0442
g__Bacteroides	s__Bacteroides_caccae	0.0151	0.0151
g__Bacteroides	s__Bacteroides_caccae	0.0151	0.0151
g__Bacteroides	s__Bacteroides_caccae	0.0133	0.0133
g__Bacteroides	s__Bacteroides_caccae	0.0151	0.0151
g__Bacteroides	s__Bacteroides_caccae	0.0151	0.0151

g__Bacteroides	s__Bacteroides_caccae	0.0151	0.0151
g__Bacteroides	s__Bacteroides_caccae	0.0151	0.0151
g__Bacteroides	s__Bacteroides_caccae	0.0151	0.0151
g__Bacteroides	s__Bacteroides_caccae	0.0151	0.0151
g__Bacteroides	s__Bacteroides_caccae	0.0151	0.0151
g__Bacteroides	s__Bacteroides_caccae	0.0146	0.0146
g__Bacteroides	s__Bacteroides_caccae	0.0431	0.0431
g__Bacteroides	s__Bacteroides_caccae	0.0133	0.0133
g__Bacteroides	s__Bacteroides_caccae	0.0151	0.0151
g__Bacteroides	s__Bacteroides_caccae	0.0146	0.0146
g__Bacteroides	s__Bacteroides_caccae	0.0151	0.0151
g__Bacteroides	s__Bacteroides_caccae	0.0151	0.0151
g__Bacteroides	s__Bacteroides_caccae	0.0151	0.0151
g__Bacteroides	s__Bacteroides_caccae	0.0151	0.0151
g__Bacteroides	s__Bacteroides_caccae	0.0431	0.0431
g__Bacteroides	s__Bacteroides_caccae	0.0151	0.0151
g__Bacteroides	s__Bacteroides_caccae	0.0151	0.0151
g__Bacteroides	s__Bacteroides_caccae	0.0151	0.0151
g__Bacteroides	s__Bacteroides_caccae	0.0151	0.0151
g__Bacteroides	s__Bacteroides_caccae	0.0151	0.0151
g__Bacteroides	s__Bacteroides_caccae	0.0151	0.0151
g__Bacteroides	s__Bacteroides_caccae	0.0151	0.0151
g__Bacteroides	s__Bacteroides_caccae	0.0151	0.0151
g__Bacteroides	s__Bacteroides_caccae	0.0151	0.0151
g__Bacteroides	s__Bacteroides_caccae	0.0151	0.0151
g__Bacteroides	s__Bacteroides_caccae	0.0151	0.0151
g__Bacteroides	s__Bacteroides_caccae	0.0151	0.0151
g__Bacteroides	s__Bacteroides_caccae	0.0442	0.0442
g__Bacteroides	s__Bacteroides_caccae	0.0151	0.0151
g__Bacteroides	s__Bacteroides_caccae	0.0151	0.0151
g__Bacteroides	s__Bacteroides_caccae	0.0151	0.0151
g__Bacteroides	s__Bacteroides_caccae	0.0442	0.0442
g__Bacteroides	s__Bacteroides_caccae	0.0151	0.0151
g__Bacteroides	s__Bacteroides_caccae	0.0151	0.0151
g__Bacteroides	s__Bacteroides_caccae	0.0151	0.0151
g__Bacteroides	s__Bacteroides_caccae	0.0151	0.0151
g__Bacteroides	s__Bacteroides_caccae	0.0442	0.0442
g__Bacteroides	s__Bacteroides_caccae	0.0151	0.0151
g__Bacteroides	s__Bacteroides_caccae	0.0442	0.0442
g__Bacteroides	s__Bacteroides_caccae	0.0133	0.0133
g__Bacteroides	s__Bacteroides_caccae	0.0151	0.0151
g__Bacteroides	s__Bacteroides_caccae	0.0151	0.0151
g__Bacteroides	s__Bacteroides_caccae	0.0151	0.0151

g__Bacteroides	s__Bacteroides_caccae	0.0151	0.0151
g__Bacteroides	s__Bacteroides_caccae	0.0151	0.0151
g__Bacteroides	s__Bacteroides_caccae	0.0151	0.0151
g__Bacteroides	s__Bacteroides_caccae	0.0151	0.0151
g__Bacteroides	s__Bacteroides_caccae	0.0151	0.0151
g__Bacteroides	s__Bacteroides_caccae	0.0442	0.0442
g__Bacteroides	s__Bacteroides_caccae	0.0151	0.0151
g__Bacteroides	s__Bacteroides_caccae	0.0151	0.0151
g__Bacteroides	s__Bacteroides_caecimuris	0.0151	0.0151
g__Bacteroides	s__Bacteroides_caecimuris	0.0151	0.0151
g__Bacteroides	s__Bacteroides_caecimuris	0.0151	0.0151
g__Bacteroides	s__Bacteroides_caecimuris	0.0151	0.0151
g__Bacteroides	s__Bacteroides_caecimuris	0.0442	0.0442
g__Bacteroides	s__Bacteroides_caecimuris	0.0151	0.0151
g__Bacteroides	s__Bacteroides_caecimuris	0.0151	0.0151
g__Bacteroides	s__Bacteroides_caecimuris	0.0151	0.0151
g__Bacteroides	s__Bacteroides_caecimuris	0.0151	0.0151
g__Bacteroides	s__Bacteroides_caecimuris	0.0151	0.0151
g__Bacteroides	s__Bacteroides_caecimuris	0.0151	0.0151
g__Bacteroides	s__Bacteroides_caecimuris	0.0151	0.0151
g__Bacteroides	s__Bacteroides_caecimuris	0.0151	0.0151
g__Bacteroides	s__Bacteroides_caecimuris	0.0151	0.0151
g__Bacteroides	s__Bacteroides_caecimuris	0.0151	0.0151
g__Bacteroides	s__Bacteroides_caecimuris	0.0151	0.0151
g__Bacteroides	s__Bacteroides_caecimuris	0.0151	0.0151
g__Bacteroides	s__Bacteroides_caecimuris	0.0151	0.0151
g__Bacteroides	s__Bacteroides_caecimuris	0.0151	0.0151
g__Bacteroides	s__Bacteroides_caecimuris	0.0151	0.0151
g__Bacteroides	s__Bacteroides_caecimuris	0.0151	0.0151
g__Bacteroides	s__Bacteroides_caecimuris	0.0151	0.0151
g__Bacteroides	s__Bacteroides_caecimuris	0.0151	0.0151
g__Bacteroides	s__Bacteroides_caecimuris	0.0151	0.0151
g__Bacteroides	s__Bacteroides_caecimuris	0.0151	0.0151
g__Bacteroides	s__Bacteroides_caecimuris	0.0151	0.0151
g__Bacteroides	s__Bacteroides_caecimuris	0.0151	0.0151
g__Bacteroides	s__Bacteroides_caecimuris	0.0151	0.0151
g__Bacteroides	s__Bacteroides_caecimuris	0.0151	0.0151
g__Bacteroides	s__Bacteroides_caecimuris	0.0151	0.0151
g__Bacteroides	s__Bacteroides_caecimuris	0.0151	0.0151
g__Bacteroides	s__Bacteroides_caecimuris	0.0151	0.0151
g__Bacteroides	s__Bacteroides_caecimuris	0.0151	0.0151
g__Bacteroides	s__Bacteroides_caecimuris	0.0151	0.0151
g__Bacteroides	s__Bacteroides_caecimuris	0.0151	0.0151

g__Bacteroides	s__Bacteroides_caecimuris	0.0151	0.0151
g__Bacteroides	s__Bacteroides_caecimuris	0.0151	0.0151
g__Bacteroides	s__Bacteroides_caecimuris	0.0151	0.0151
g__Bacteroides	s__Bacteroides_caecimuris	0.0151	0.0151
g__Bacteroides	s__Bacteroides_caecimuris	0.0442	0.0442
g__Bacteroides	s__Bacteroides_caecimuris	0.0151	0.0151
g__Bacteroides	s__Bacteroides_caecimuris	0.0151	0.0151
g__Bacteroides	s__Bacteroides_caecimuris	0.0151	0.0151
g__Bacteroides	s__Bacteroides_caecimuris	0.0151	0.0151
g__Bacteroides	s__Bacteroides_caecimuris	0.0151	0.0151
g__Bacteroides	s__Bacteroides_caecimuris	0.0151	0.0151
g__Bacteroides	s__Bacteroides_caecimuris	0.0151	0.0151
g__Bacteroides	s__Bacteroides_caecimuris	0.0151	0.0151
g__Bacteroides	s__Bacteroides_caecimuris	0.0151	0.0151
g__Bacteroides	s__Bacteroides_caecimuris	0.0151	0.0151
g__Bacteroides	s__Bacteroides_caecimuris	0.0151	0.0151
g__Bacteroides	s__Bacteroides_caecimuris	0.0151	0.0151
g__Bacteroides	s__Bacteroides_caecimuris	0.0151	0.0151
g__Bacteroides	s__Bacteroides_caecimuris	0.0151	0.0151
g__Bacteroides	s__Bacteroides_caecimuris	0.0151	0.0151
g__Bacteroides	s__Bacteroides_caecimuris	0.0151	0.0151
g__Bacteroides	s__Bacteroides_caecimuris	0.0151	0.0151
g__Bacteroides	s__Bacteroides_caecimuris	0.0151	0.0151
g__Bacteroides	s__Bacteroides_caecimuris	0.0431	0.0431
g__Bacteroides	s__Bacteroides_caecimuris	0.0151	0.0151
g__Bacteroides	s__Bacteroides_caecimuris	0.0151	0.0151
g__Bacteroides	s__Bacteroides_caecimuris	0.0151	0.0151
g__Bacteroides	s__Bacteroides_caecimuris	0.0151	0.0151
g__Bacteroides	s__Bacteroides_caecimuris	0.0151	0.0151
g__Bacteroides	s__Bacteroides_caecimuris	0.0151	0.0151
g__Bacteroides	s__Bacteroides_caecimuris	0.0151	0.0151
g__Bacteroides	s__Bacteroides_caecimuris	0.0151	0.0151
g__Bacteroides	s__Bacteroides_caecimuris	0.0151	0.0151
g__Bacteroides	s__Bacteroides_caecimuris	0.0442	0.0442
g__Bacteroides	s__Bacteroides_caecimuris	0.0151	0.0151
g__Bacteroides	s__Bacteroides_caecimuris	0.0151	0.0151
g__Bacteroides	s__Bacteroides_caecimuris	0.0151	0.0151
g__Bacteroides	s__Bacteroides_caecimuris	0.0151	0.0151
g__Bacteroides	s__Bacteroides_caecimuris	0.0151	0.0151
g__Bacteroides	s__Bacteroides_caecimuris	0.0151	0.0151
g__Bacteroides	s__Bacteroides_caecimuris	0.0151	0.0151
g__Bacteroides	s__Bacteroides_caecimuris	0.0151	0.0151
g__Bacteroides	s__Bacteroides_caecimuris	0.0151	0.0151

g__Bacteroides	s__Bacteroides_caecimuris	0.0151	0.0151
g__Bacteroides	s__Bacteroides_caecimuris	0.0151	0.0151
g__Bacteroides	s__Bacteroides_caecimuris	0.0151	0.0151
g__Bacteroides	s__Bacteroides_caecimuris	0.0151	0.0151
g__Bacteroides	s__Bacteroides_caecimuris	0.0151	0.0151
g__Bacteroides	s__Bacteroides_caecimuris	0.0151	0.0151
g__Bacteroides	s__Bacteroides_caecimuris	0.0151	0.0151
g__Bacteroides	s__Bacteroides_caecimuris	0.0151	0.0151
g__Bacteroides	s__Bacteroides_caecimuris	0.0151	0.0151
g__Bacteroides	s__Bacteroides_caecimuris	0.0151	0.0151
g__Bacteroides	s__Bacteroides_caecimuris	0.0151	0.0151
g__Bacteroides	s__Bacteroides_caecimuris	0.0151	0.0151
g__Bacteroides	s__Bacteroides_caecimuris	0.0151	0.0151
g__Bacteroides	s__Bacteroides_caecimuris	0.0442	0.0442
g__Bacteroides	s__Bacteroides_caecimuris	0.0151	0.0151
g__Bacteroides	s__Bacteroides_caecimuris	0.0151	0.0151
g__Bacteroides	s__Bacteroides_caecimuris	0.0151	0.0151
g__Bacteroides	s__Bacteroides_caecimuris	0.0151	0.0151
g__Bacteroides	s__Bacteroides_caecimuris	0.0151	0.0151
g__Bacteroides	s__Bacteroides_caecimuris	0.0151	0.0151
g__Bacteroides	s__Bacteroides_caecimuris	0.0151	0.0151
g__Bacteroides	s__Bacteroides_caecimuris	0.0151	0.0151
g__Bacteroides	s__Bacteroides_caecimuris	0.0151	0.0151
g__Bacteroides	s__Bacteroides_caecimuris	0.0151	0.0151
g__Bacteroides	s__Bacteroides_caecimuris	0.0442	0.0442
g__Bacteroides	s__Bacteroides_caecimuris	0.0151	0.0151
g__Bacteroides	s__Bacteroides_caecimuris	0.0151	0.0151
g__Bacteroides	s__Bacteroides_caecimuris	0.0151	0.0151
g__Bacteroides	s__Bacteroides_caecimuris	0.0151	0.0151
g__Bacteroides	s__Bacteroides_caecimuris	0.0151	0.0151
g__Bacteroides	s__Bacteroides_caecimuris	0.0151	0.0151
g__Bacteroides	s__Bacteroides_caecimuris	0.0151	0.0151
g__Bacteroides	s__Bacteroides_caecimuris	0.0151	0.0151
g__Bacteroides	s__Bacteroides_caecimuris	0.0151	0.0151
g__Bacteroides	s__Bacteroides_caecimuris	0.0151	0.0151
g__Bacteroides	s__Bacteroides_caecimuris	0.0151	0.0151
g__Bacteroides	s__Bacteroides_caecimuris	0.0151	0.0151
g__Bacteroides	s__Bacteroides_caecimuris	0.0151	0.0151
g__Bacteroides	s__Bacteroides_caecimuris	0.0151	0.0151
g__Bacteroides	s__Bacteroides_caecimuris	0.0151	0.0151
g__Bacteroides	s__Bacteroides_caecimuris	0.0151	0.0151
g__Bacteroides	s__Bacteroides_caecimuris	0.0151	0.0151
g__Bacteroides	s__Bacteroides_caecimuris	0.0151	0.0151

g__Bacteroides	s__Bacteroides_caecimuris	0.0151	0.0151
g__Bacteroides	s__Bacteroides_caecimuris	0.0151	0.0151
g__Bacteroides	s__Bacteroides_caecimuris	0.0151	0.0151
g__Bacteroides	s__Bacteroides_caecimuris	0.0151	0.0151
g__Bacteroides	s__Bacteroides_caecimuris	0.0151	0.0151
g__Bacteroides	s__Bacteroides_caecimuris	0.0151	0.0151
g__Bacteroides	s__Bacteroides_caecimuris	0.0151	0.0151
g__Bacteroides	s__Bacteroides_caecimuris	0.0151	0.0151
g__Bacteroides	s__Bacteroides_caecimuris	0.0151	0.0151
g__Bacteroides	s__Bacteroides_caecimuris	0.0151	0.0151
g__Bacteroides	s__Bacteroides_caecimuris	0.0151	0.0151
g__Bacteroides	s__Bacteroides_caecimuris	0.0442	0.0442
g__Bacteroides	s__Bacteroides_caecimuris	0.0151	0.0151
g__Bacteroides	s__Bacteroides_caecimuris	0.0442	0.0442
g__Bacteroides	s__Bacteroides_caecimuris	0.0151	0.0151
g__Bacteroides	s__Bacteroides_caecimuris	0.0151	0.0151
g__Bacteroides	s__Bacteroides_caecimuris	0.0151	0.0151
g__Bacteroides	s__Bacteroides_caecimuris	0.0151	0.0151
g__Bacteroides	s__Bacteroides_caecimuris	0.0151	0.0151
g__Bacteroides	s__Bacteroides_caecimuris	0.0442	0.0442
g__Bacteroides	s__Bacteroides_caecimuris	0.0151	0.0151
g__Bacteroides	s__Bacteroides_caecimuris	0.0151	0.0151
g__Bacteroides	s__Bacteroides_caecimuris	0.0151	0.0151
g__Bacteroides	s__Bacteroides_caecimuris	0.0151	0.0151
g__Bacteroides	s__Bacteroides_caecimuris	0.0151	0.0151
g__Bacteroides	s__Bacteroides_caecimuris	0.0151	0.0151
g__Bacteroides	s__Bacteroides_caecimuris	0.0151	0.0151
g__Bacteroides	s__Bacteroides_caecimuris	0.0151	0.0151
g__Bacteroides	s__Bacteroides_caecimuris	0.0151	0.0151
g__Bacteroides	s__Bacteroides_caecimuris	0.0151	0.0151
g__Bacteroides	s__Bacteroides_caecimuris	0.0151	0.0151
g__Bacteroides	s__Bacteroides_caecimuris	0.0151	0.0151
g__Bacteroides	s__Bacteroides_caecimuris	0.0151	0.0151
g__Bacteroides	s__Bacteroides_caecimuris	0.0151	0.0151
g__Bacteroides	s__Bacteroides_caecimuris	0.0151	0.0151
g__Bacteroides	s__Bacteroides_caecimuris	0.0151	0.0151
g__Bacteroides	s__Bacteroides_caecimuris	0.0151	0.0151
g__Bacteroides	s__Bacteroides_caecimuris	0.0442	0.0442
g__Bacteroides	s__Bacteroides_caecimuris	0.0151	0.0151
g__Bacteroides	s__Bacteroides_caecimuris	0.0151	0.0151
g__Bacteroides	s__Bacteroides_caecimuris	0.0151	0.0151
g__Bacteroides	s__Bacteroides_caecimuris	0.0151	0.0151
g__Bacteroides	s__Bacteroides_caecimuris	0.0151	0.0151

g__Bacteroides	s__Bacteroides_caecimuris	0.0151	0.0151
g__Bacteroides	s__Bacteroides_caecimuris	0.0151	0.0151
g__Bacteroides	s__Bacteroides_caecimuris	0.0151	0.0151
g__Bacteroides	s__Bacteroides_caecimuris	0.0151	0.0151
g__Bacteroides	s__Bacteroides_caecimuris	0.0151	0.0151
g__Bacteroides	s__Bacteroides_caecimuris	0.0151	0.0151
g__Bacteroides	s__Bacteroides_caecimuris	0.0151	0.0151
g__Bacteroides	s__Bacteroides_caecimuris	0.0151	0.0151
g__Bacteroides	s__Bacteroides_caecimuris	0.0151	0.0151
g__Bacteroides	s__Bacteroides_caecimuris	0.0151	0.0151
g__Bacteroides	s__Bacteroides_caecimuris	0.0151	0.0151
g__Bacteroides	s__Bacteroides_caecimuris	0.0151	0.0151
g__Bacteroides	s__Bacteroides_caecimuris	0.0151	0.0151
g__Bacteroides	s__Bacteroides_caecimuris	0.0151	0.0151
g__Bacteroides	s__Bacteroides_caecimuris	0.0151	0.0151
g__Bacteroides	s__Bacteroides_caecimuris	0.0151	0.0151
g__Bacteroides	s__Bacteroides_caecimuris	0.0151	0.0151
g__Bacteroides	s__Bacteroides_caecimuris	0.0151	0.0151
g__Bacteroides	s__Bacteroides_caecimuris	0.0151	0.0151
g__Bacteroides	s__Bacteroides_caecimuris	0.0442	0.0442
g__Bacteroides	s__Bacteroides_caecimuris	0.0442	0.0442
g__Bacteroides	s__Bacteroides_caecimuris	0.0151	0.0151
g__Bacteroides	s__Bacteroides_caecimuris	0.0146	0.0146
g__Bacteroides	s__Bacteroides_caecimuris	0.0151	0.0151
g__Bacteroides	s__Bacteroides_caecimuris	0.0151	0.0151
g__Bacteroides	s__Bacteroides_caecimuris	0.0151	0.0151
g__Bacteroides	s__Bacteroides_caecimuris	0.0151	0.0151
g__Bacteroides	s__Bacteroides_caecimuris	0.0151	0.0151
g__Bacteroides	s__Bacteroides_caecimuris	0.0151	0.0151
g__Bacteroides	s__Bacteroides_caecimuris	0.0151	0.0151
g__Bacteroides	s__Bacteroides_caecimuris	0.0151	0.0151
g__Bacteroides	s__Bacteroides_caecimuris	0.0151	0.0151
g__Bacteroides	s__Bacteroides_caecimuris	0.0442	0.0442
g__Bacteroides	s__Bacteroides_caecimuris	0.0151	0.0151
g__Bacteroides	s__Bacteroides_caecimuris	0.0151	0.0151
g__Bacteroides	s__Bacteroides_caecimuris	0.0151	0.0151
g__Bacteroides	s__Bacteroides_caecimuris	0.0151	0.0151
g__Bacteroides	s__Bacteroides_caecimuris	0.0151	0.0151
g__Bacteroides	s__Bacteroides_caecimuris	0.0151	0.0151
g__Bacteroides	s__Bacteroides_caecimuris	0.0151	0.0151
g__Bacteroides	s__Bacteroides_caecimuris	0.0151	0.0151
g__Bacteroides	s__Bacteroides_caecimuris	0.0151	0.0151
g__Bacteroides	s__Bacteroides_caecimuris	0.0151	0.0151

g__Bacteroides	s__Bacteroides_caecimuris	0.0442	0.0442
g__Bacteroides	s__Bacteroides_caecimuris	0.0442	0.0442
g__Bacteroides	s__Bacteroides_caecimuris	0.0442	0.0442
g__Bacteroides	s__Bacteroides_caecimuris	0.0151	0.0151
g__Bacteroides	s__Bacteroides_caecimuris	0.0151	0.0151
g__Bacteroides	s__Bacteroides_caecimuris	0.0151	0.0151
g__Bacteroides	s__Bacteroides_caecimuris	0.0151	0.0151
g__Bacteroides	s__Bacteroides_caecimuris	0.0151	0.0151
g__Bacteroides	s__Bacteroides_caecimuris	0.0151	0.0151
g__Bacteroides	s__Bacteroides_caecimuris	0.0151	0.0151
g__Bacteroides	s__Bacteroides_caecimuris	0.0151	0.0151
g__Bacteroides	s__Bacteroides_caecimuris	0.0151	0.0151
g__Bacteroides	s__Bacteroides_caecimuris	0.0151	0.0151
g__Bacteroides	s__Bacteroides_caecimuris	0.0442	0.0442
g__Bacteroides	s__Bacteroides_caecimuris	0.0151	0.0151
g__Bacteroides	s__Bacteroides_caecimuris	0.0442	0.0442
g__Bacteroides	s__Bacteroides_caecimuris	0.0151	0.0151
g__Bacteroides	s__Bacteroides_caecimuris	0.0151	0.0151
g__Bacteroides	s__Bacteroides_caecimuris	0.0151	0.0151
g__Bacteroides	s__Bacteroides_caecimuris	0.0151	0.0151
g__Bacteroides	s__Bacteroides_caecimuris	0.0151	0.0151
g__Bacteroides	s__Bacteroides_caecimuris	0.0151	0.0151
g__Bacteroides	s__Bacteroides_caecimuris	0.0151	0.0151
g__Bacteroides	s__Bacteroides_caecimuris	0.0151	0.0151
g__Bacteroides	s__Bacteroides_caecimuris	0.0151	0.0151
g__Bacteroides	s__Bacteroides_caecimuris	0.0151	0.0151
g__Bacteroides	s__Bacteroides_caecimuris	0.0151	0.0151
g__Bacteroides	s__Bacteroides_caecimuris	0.0151	0.0151
g__Bacteroides	s__Bacteroides_caecimuris	0.0442	0.0442
g__Bacteroides	s__Bacteroides_caecimuris	0.0151	0.0151
g__Bacteroides	s__Bacteroides_caecimuris	0.0151	0.0151
g__Bacteroides	s__Bacteroides_caecimuris	0.0151	0.0151
g__Bacteroides	s__Bacteroides_caecimuris	0.0151	0.0151
g__Bacteroides	s__Bacteroides_caecimuris	0.0151	0.0151
g__Bacteroides	s__Bacteroides_caecimuris	0.0151	0.0151
g__Bacteroides	s__Bacteroides_caecimuris	0.0151	0.0151
g__Bacteroides	s__Bacteroides_caecimuris	0.0151	0.0151
g__Bacteroides	s__Bacteroides_caecimuris	0.0151	0.0151
g__Bacteroides	s__Bacteroides_caecimuris	0.0151	0.0151
g__Bacteroides	s__Bacteroides_caecimuris	0.0151	0.0151
g__Bacteroides	s__Bacteroides_caecimuris	0.0151	0.0151
g__Bacteroides	s__Bacteroides_caecimuris	0.0151	0.0151
g__Bacteroides	s__Bacteroides_caecimuris	0.0151	0.0151

g__Bacteroides	s__Bacteroides_caecimuris	0.0151	0.0151
g__Bacteroides	s__Bacteroides_caecimuris	0.0151	0.0151
g__Bacteroides	s__Bacteroides_caecimuris	0.0151	0.0151
g__Bacteroides	s__Bacteroides_caecimuris	0.0151	0.0151
g__Bacteroides	s__Bacteroides_caecimuris	0.0151	0.0151
g__Bacteroides	s__Bacteroides_caecimuris	0.0151	0.0151
g__Bacteroides	s__Bacteroides_caecimuris	0.0151	0.0151
g__Bacteroides	s__Bacteroides_caecimuris	0.0151	0.0151
g__Bacteroides	s__Bacteroides_caecimuris	0.0442	0.0442
g__Bacteroides	s__Bacteroides_caecimuris	0.0151	0.0151
g__Bacteroides	s__Bacteroides_caecimuris	0.0151	0.0151
g__Bacteroides	s__Bacteroides_caecimuris	0.0151	0.0151
g__Bacteroides	s__Bacteroides_caecimuris	0.0151	0.0151
g__Bacteroides	s__Bacteroides_caecimuris	0.0151	0.0151
g__Bacteroides	s__Bacteroides_caecimuris	0.0151	0.0151
g__Bacteroides	s__Bacteroides_caecimuris	0.0151	0.0151
g__Bacteroides	s__Bacteroides_caecimuris	0.0151	0.0151
g__Bacteroides	s__Bacteroides_caecimuris	0.0151	0.0151
g__Bacteroides	s__Bacteroides_caecimuris	0.0151	0.0151
g__Bacteroides	s__Bacteroides_caecimuris	0.0151	0.0151
g__Bacteroides	s__Bacteroides_caecimuris	0.0151	0.0151
g__Bacteroides	s__Bacteroides_caecimuris	0.0151	0.0151
g__Bacteroides	s__Bacteroides_caecimuris	0.0151	0.0151
g__Bacteroides	s__Bacteroides_caecimuris	0.0151	0.0151
g__Bacteroides	s__Bacteroides_caecimuris	0.0151	0.0151
g__Bacteroides	s__Bacteroides_caecimuris	0.0151	0.0151
g__Bacteroides	s__Bacteroides_caecimuris	0.0151	0.0151
g__Bacteroides	s__Bacteroides_caecimuris	0.0151	0.0151
g__Bacteroides	s__Bacteroides_caecimuris	0.0151	0.0151
g__Bacteroides	s__Bacteroides_caecimuris	0.0151	0.0151
g__Bacteroides	s__Bacteroides_caecimuris	0.0442	0.0442
g__Bacteroides	s__Bacteroides_caecimuris	0.0151	0.0151
g__Bacteroides	s__Bacteroides_caecimuris	0.0151	0.0151
g__Bacteroides	s__Bacteroides_caecimuris	0.0151	0.0151
g__Bacteroides	s__Bacteroides_caecimuris	0.0151	0.0151
g__Bacteroides	s__Bacteroides_caecimuris	0.0442	0.0442
g__Bacteroides	s__Bacteroides_caecimuris	0.0151	0.0151
g__Bacteroides	s__Bacteroides_caecimuris	0.0151	0.0151
g__Bacteroides	s__Bacteroides_caecimuris	0.0151	0.0151
g__Bacteroides	s__Bacteroides_caecimuris	0.0151	0.0151
g__Bacteroides	s__Bacteroides_caecimuris	0.0151	0.0151
g__Bacteroides	s__Bacteroides_caecimuris	0.0151	0.0151
g__Bacteroides	s__Bacteroides_caecimuris	0.0151	0.0151

g__Bacteroides	s__Bacteroides_caecimuris	0.0151	0.0151
g__Bacteroides	s__Bacteroides_caecimuris	0.0151	0.0151
g__Bacteroides	s__Bacteroides_caecimuris	0.0151	0.0151
g__Bacteroides	s__Bacteroides_caecimuris	0.0151	0.0151
g__Bacteroides	s__Bacteroides_caecimuris	0.0151	0.0151
g__Bacteroides	s__Bacteroides_caecimuris	0.0151	0.0151
g__Bacteroides	s__Bacteroides_caecimuris	0.0151	0.0151
g__Bacteroides	s__Bacteroides_caecimuris	0.0151	0.0151
g__Bacteroides	s__Bacteroides_caecimuris	0.0151	0.0151
g__Bacteroides	s__Bacteroides_caecimuris	0.0151	0.0151
g__Bacteroides	s__Bacteroides_caecimuris	0.0151	0.0151
g__Bacteroides	s__Bacteroides_caecimuris	0.0151	0.0151
g__Bacteroides	s__Bacteroides_caecimuris	0.0151	0.0151
g__Bacteroides	s__Bacteroides_caecimuris	0.0151	0.0151
g__Bacteroides	s__Bacteroides_caecimuris	0.0151	0.0151
g__Bacteroides	s__Bacteroides_caecimuris	0.0151	0.0151
g__Bacteroides	s__Bacteroides_caecimuris	0.0151	0.0151
g__Bacteroides	s__Bacteroides_caecimuris	0.0151	0.0151
g__Bacteroides	s__Bacteroides_caecimuris	0.0151	0.0151
g__Bacteroides	s__Bacteroides_caecimuris	0.0442	0.0442
g__Bacteroides	s__Bacteroides_caecimuris	0.0151	0.0151
g__Bacteroides	s__Bacteroides_caecimuris	0.0151	0.0151
g__Bacteroides	s__Bacteroides_caecimuris	0.0146	0.0146
g__Bacteroides	s__Bacteroides_caecimuris	0.0151	0.0151
g__Bacteroides	s__Bacteroides_caecimuris	0.0151	0.0151
g__Bacteroides	s__Bacteroides_caecimuris	0.0151	0.0151
g__Bacteroides	s__Bacteroides_caecimuris	0.0151	0.0151
g__Bacteroides	s__Bacteroides_caecimuris	0.0151	0.0151
g__Bacteroides	s__Bacteroides_caecimuris	0.0151	0.0151
g__Bacteroides	s__Bacteroides_caecimuris	0.0151	0.0151
g__Bacteroides	s__Bacteroides_caecimuris	0.0151	0.0151
g__Bacteroides	s__Bacteroides_caecimuris	0.0151	0.0151
g__Bacteroides	s__Bacteroides_caecimuris	0.0151	0.0151
g__Bacteroides	s__Bacteroides_caecimuris	0.0442	0.0442
g__Bacteroides	s__Bacteroides_caecimuris	0.0151	0.0151
g__Bacteroides	s__Bacteroides_caecimuris	0.0151	0.0151
g__Bacteroides	s__Bacteroides_caecimuris	0.0151	0.0151
g__Bacteroides	s__Bacteroides_caecimuris	0.0151	0.0151
g__Bacteroides	s__Bacteroides_caecimuris	0.0151	0.0151
g__Bacteroides	s__Bacteroides_caecimuris	0.0151	0.0151
g__Bacteroides	s__Bacteroides_caecimuris	0.0151	0.0151
g__Bacteroides	s__Bacteroides_caecimuris	0.0151	0.0151
g__Bacteroides	s__Bacteroides_caecimuris	0.0151	0.0151

g__Bacteroides	s__Bacteroides_caecimuris	0.0151	0.0151
g__Bacteroides	s__Bacteroides_caecimuris	0.0151	0.0151
g__Bacteroides	s__Bacteroides_caecimuris	0.0151	0.0151
g__Bacteroides	s__Bacteroides_caecimuris	0.0151	0.0151
g__Bacteroides	s__Bacteroides_caecimuris	0.0151	0.0151
g__Bacteroides	s__Bacteroides_caecimuris	0.0151	0.0151
g__Bacteroides	s__Bacteroides_caecimuris	0.0151	0.0151
g__Bacteroides	s__Bacteroides_caecimuris	0.0151	0.0151
g__Bacteroides	s__Bacteroides_caecimuris	0.0151	0.0151
g__Bacteroides	s__Bacteroides_caecimuris	0.0151	0.0151
g__Bacteroides	s__Bacteroides_caecimuris	0.0151	0.0151
g__Bacteroides	s__Bacteroides_caecimuris	0.0151	0.0151
g__Bacteroides	s__Bacteroides_caecimuris	0.0151	0.0151
g__Bacteroides	s__Bacteroides_caecimuris	0.0151	0.0151
g__Bacteroides	s__Bacteroides_caecimuris	0.0151	0.0151
g__Bacteroides	s__Bacteroides_caecimuris	0.0151	0.0151
g__Bacteroides	s__Bacteroides_caecimuris	0.0151	0.0151
g__Bacteroides	s__Bacteroides_caecimuris	0.0151	0.0151
g__Bacteroides	s__Bacteroides_caecimuris	0.0151	0.0151
g__Bacteroides	s__Bacteroides_caecimuris	0.0151	0.0151
g__Bacteroides	s__Bacteroides_caecimuris	0.0151	0.0151
g__Bacteroides	s__Bacteroides_caecimuris	0.0151	0.0151
g__Bacteroides	s__Bacteroides_caecimuris	0.0151	0.0151
g__Bacteroides	s__Bacteroides_caecimuris	0.0151	0.0151
g__Bacteroides	s__Bacteroides_caecimuris	0.0151	0.0151
g__Bacteroides	s__Bacteroides_caecimuris	0.0151	0.0151
g__Bacteroides	s__Bacteroides_caecimuris	0.0151	0.0151
g__Bacteroides	s__Bacteroides_caecimuris	0.0151	0.0151
g__Bacteroides	s__Bacteroides_caecimuris	0.0151	0.0151
g__Bacteroides	s__Bacteroides_caecimuris	0.0151	0.0151
g__Bacteroides	s__Bacteroides_caecimuris	0.0151	0.0151
g__Bacteroides	s__Bacteroides_caecimuris	0.0151	0.0151
g__Bacteroides	s__Bacteroides_caecimuris	0.0151	0.0151
g__Bacteroides	s__Bacteroides_caecimuris	0.0151	0.0151
g__Bacteroides	s__Bacteroides_caecimuris	0.0151	0.0151
g__Bacteroides	s__Bacteroides_caecimuris	0.0151	0.0151
g__Bacteroides	s__Bacteroides_caecimuris	0.0151	0.0151
g__Bacteroides	s__Bacteroides_caecimuris	0.0151	0.0151
g__Bacteroides	s__Bacteroides_caecimuris	0.0151	0.0151
g__Bacteroides	s__Bacteroides_caecimuris	0.0151	0.0151
g__Bacteroides	s__Bacteroides_caecimuris	0.0151	0.0151
g__Bacteroides	s__Bacteroides_caecimuris	0.0151	0.0151
g__Bacteroides	s__Bacteroides_caecimuris	0.0151	0.0151

g__Bacteroides	s__Bacteroides_caecimuris	0.0151	0.0151
g__Bacteroides	s__Bacteroides_caecimuris	0.0151	0.0151
g__Bacteroides	s__Bacteroides_caecimuris	0.0151	0.0151
g__Bacteroides	s__Bacteroides_caecimuris	0.0151	0.0151
g__Bacteroides	s__Bacteroides_caecimuris	0.0151	0.0151
g__Bacteroides	s__Bacteroides_caecimuris	0.0151	0.0151
g__Bacteroides	s__Bacteroides_caecimuris	0.0151	0.0151
g__Bacteroides	s__Bacteroides_caecimuris	0.0151	0.0151
g__Bacteroides	s__Bacteroides_caecimuris	0.0151	0.0151
g__Bacteroides	s__Bacteroides_caecimuris	0.0151	0.0151
g__Bacteroides	s__Bacteroides_cellulosilyticus	0.0151	0.0151
g__Bacteroides	s__Bacteroides_cellulosilyticus	0.0151	0.0151
g__Bacteroides	s__Bacteroides_cellulosilyticus	0.0179	0.0179
g__Bacteroides	s__Bacteroides_cellulosilyticus	0.0146	0.0146
g__Bacteroides	s__Bacteroides_cellulosilyticus	0.0442	0.0442
g__Bacteroides	s__Bacteroides_cellulosilyticus	0.0146	0.0146
g__Bacteroides	s__Bacteroides_cellulosilyticus	0.0151	0.0151
g__Bacteroides	s__Bacteroides_cellulosilyticus	0.0431	0.0431
g__Bacteroides	s__Bacteroides_cellulosilyticus	0.0151	0.0151
g__Bacteroides	s__Bacteroides_cellulosilyticus	0.0442	0.0442
g__Bacteroides	s__Bacteroides_cellulosilyticus	0.0146	0.0146
g__Bacteroides	s__Bacteroides_cellulosilyticus	0.0151	0.0151
g__Bacteroides	s__Bacteroides_cellulosilyticus	0.0151	0.0151
g__Bacteroides	s__Bacteroides_cellulosilyticus	0.0151	0.0151
g__Bacteroides	s__Bacteroides_cellulosilyticus	0.0151	0.0151
g__Bacteroides	s__Bacteroides_cellulosilyticus	0.0151	0.0151
g__Bacteroides	s__Bacteroides_cellulosilyticus	0.0151	0.0151
g__Bacteroides	s__Bacteroides_cellulosilyticus	0.0151	0.0151
g__Bacteroides	s__Bacteroides_cellulosilyticus	0.0442	0.0442
g__Bacteroides	s__Bacteroides_cellulosilyticus	0.0151	0.0151
g__Bacteroides	s__Bacteroides_cellulosilyticus	0.0151	0.0151
g__Bacteroides	s__Bacteroides_cellulosilyticus	0.0151	0.0151
g__Bacteroides	s__Bacteroides_cellulosilyticus	0.0442	0.0442
g__Bacteroides	s__Bacteroides_cellulosilyticus	0.0442	0.0442
g__Bacteroides	s__Bacteroides_cellulosilyticus	0.0151	0.0151
g__Bacteroides	s__Bacteroides_cellulosilyticus	0.0442	0.0442
g__Bacteroides	s__Bacteroides_cellulosilyticus	0.0151	0.0151
g__Bacteroides	s__Bacteroides_cellulosilyticus	0.0151	0.0151
g__Bacteroides	s__Bacteroides_cellulosilyticus	0.0442	0.0442
g__Bacteroides	s__Bacteroides_cellulosilyticus	0.0151	0.0151
g__Bacteroides	s__Bacteroides_cellulosilyticus	0.0151	0.0151
g__Bacteroides	s__Bacteroides_cellulosilyticus	0.0151	0.0151
g__Bacteroides	s__Bacteroides_cellulosilyticus	0.0442	0.0442

g__Bacteroides	s__Bacteroides_cellulosilyticus	0.0442	0.0442
g__Bacteroides	s__Bacteroides_cellulosilyticus	0.0431	0.0431
g__Bacteroides	s__Bacteroides_cellulosilyticus	0.0442	0.0442
g__Bacteroides	s__Bacteroides_cellulosilyticus	0.0442	0.0442
g__Bacteroides	s__Bacteroides_cellulosilyticus	0.0151	0.0151
g__Bacteroides	s__Bacteroides_eggerthii	0.0151	0.0151
g__Bacteroides	s__Bacteroides_eggerthii	0.0151	0.0151
g__Bacteroides	s__Bacteroides_eggerthii	0.0442	0.0442
g__Bacteroides	s__Bacteroides_eggerthii	0.0442	0.0442
g__Bacteroides	s__Bacteroides_eggerthii	0.0151	0.0151
g__Bacteroides	s__Bacteroides_eggerthii	0.0151	0.0151
g__Bacteroides	s__Bacteroides_eggerthii	0.0151	0.0151
g__Bacteroides	s__Bacteroides_eggerthii	0.0151	0.0151
g__Bacteroides	s__Bacteroides_eggerthii	0.0151	0.0151
g__Bacteroides	s__Bacteroides_eggerthii	0.0151	0.0151
g__Bacteroides	s__Bacteroides_eggerthii	0.0151	0.0151
g__Bacteroides	s__Bacteroides_eggerthii	0.0151	0.0151
g__Bacteroides	s__Bacteroides_eggerthii	0.0151	0.0151
g__Bacteroides	s__Bacteroides_eggerthii	0.0151	0.0151
g__Bacteroides	s__Bacteroides_eggerthii	0.0151	0.0151
g__Bacteroides	s__Bacteroides_eggerthii	0.0151	0.0151
g__Bacteroides	s__Bacteroides_faecis	0.0146	0.0146
g__Bacteroides	s__Bacteroides_faecis	0.0146	0.0146
g__Bacteroides	s__Bacteroides_faecis	0.0151	0.0151
g__Bacteroides	s__Bacteroides_faecis	0.0151	0.0151
g__Bacteroides	s__Bacteroides_faecis	0.0151	0.0151
g__Bacteroides	s__Bacteroides_faecis	0.0151	0.0151
g__Bacteroides	s__Bacteroides_faecis	0.0151	0.0151
g__Bacteroides	s__Bacteroides_faecis	0.0179	0.0179
g__Bacteroides	s__Bacteroides_faecis	0.0151	0.0151
g__Bacteroides	s__Bacteroides_faecis	0.0442	0.0442
g__Bacteroides	s__Bacteroides_faecis	0.0442	0.0442
g__Bacteroides	s__Bacteroides_faecis	0.0151	0.0151
g__Bacteroides	s__Bacteroides_faecis	0.0442	0.0442
g__Bacteroides	s__Bacteroides_faecis	0.0151	0.0151
g__Bacteroides	s__Bacteroides_faecis	0.0151	0.0151
g__Bacteroides	s__Bacteroides_faecis	0.0442	0.0442
g__Bacteroides	s__Bacteroides_faecis	0.0442	0.0442
g__Bacteroides	s__Bacteroides_faecis	0.0151	0.0151
g__Bacteroides	s__Bacteroides_faecis	0.0151	0.0151
g__Bacteroides	s__Bacteroides_faecis	0.0151	0.0151
g__Bacteroides	s__Bacteroides_faecis	0.0151	0.0151
g__Bacteroides	s__Bacteroides_faecis	0.0431	0.0431

g__Bacteroides	s__Bacteroides_faecis	0.0151	0.0151
g__Bacteroides	s__Bacteroides_faecis	0.0151	0.0151
g__Bacteroides	s__Bacteroides_faecis	0.0151	0.0151
g__Bacteroides	s__Bacteroides_faecis	0.0151	0.0151
g__Bacteroides	s__Bacteroides_faecis	0.0151	0.0151
g__Bacteroides	s__Bacteroides_faecis	0.0151	0.0151
g__Bacteroides	s__Bacteroides_faecis	0.0151	0.0151
g__Bacteroides	s__Bacteroides_faecis	0.0151	0.0151
g__Bacteroides	s__Bacteroides_faecis	0.0442	0.0442
g__Bacteroides	s__Bacteroides_faecis	0.0151	0.0151
g__Bacteroides	s__Bacteroides_faecis	0.0151	0.0151
g__Bacteroides	s__Bacteroides_faecis	0.0151	0.0151
g__Bacteroides	s__Bacteroides_faecis	0.0151	0.0151
g__Bacteroides	s__Bacteroides_faecis	0.0151	0.0151
g__Bacteroides	s__Bacteroides_faecis	0.0151	0.0151
g__Bacteroides	s__Bacteroides_faecis	0.0151	0.0151
g__Bacteroides	s__Bacteroides_faecis	0.0151	0.0151
g__Bacteroides	s__Bacteroides_faecis	0.0146	0.0146
g__Bacteroides	s__Bacteroides_faecis	0.0151	0.0151
g__Bacteroides	s__Bacteroides_faecis	0.0151	0.0151
g__Bacteroides	s__Bacteroides_faecis	0.0442	0.0442
g__Bacteroides	s__Bacteroides_faecis	0.0151	0.0151
g__Bacteroides	s__Bacteroides_faecis	0.0151	0.0151
g__Bacteroides	s__Bacteroides_faecis	0.0442	0.0442
g__Bacteroides	s__Bacteroides_faecis	0.0151	0.0151
g__Bacteroides	s__Bacteroides_faecis	0.0151	0.0151
g__Bacteroides	s__Bacteroides_faecis	0.0151	0.0151
g__Bacteroides	s__Bacteroides_faecis	0.0151	0.0151
g__Bacteroides	s__Bacteroides_faecis	0.0151	0.0151
g__Bacteroides	s__Bacteroides_faecis	0.0151	0.0151
g__Bacteroides	s__Bacteroides_faecis	0.0151	0.0151
g__Bacteroides	s__Bacteroides_faecis	0.0442	0.0442
g__Bacteroides	s__Bacteroides_faecis	0.0151	0.0151
g__Bacteroides	s__Bacteroides_faecis	0.0151	0.0151
g__Bacteroides	s__Bacteroides_faecis	0.0442	0.0442
g__Bacteroides	s__Bacteroides_faecis	0.0151	0.0151
g__Bacteroides	s__Bacteroides_faecis	0.0442	0.0442
g__Bacteroides	s__Bacteroides_faecis	0.0146	0.0146
g__Bacteroides	s__Bacteroides_faecis	0.0151	0.0151
g__Bacteroides	s__Bacteroides_faecis	0.0151	0.0151
g__Bacteroides	s__Bacteroides_faecis	0.0442	0.0442
g__Bacteroides	s__Bacteroides_faecis	0.0151	0.0151
g__Bacteroides	s__Bacteroides_faecis	0.0151	0.0151

g__Bacteroides	s__Bacteroides_faecis	0.0151	0.0151
g__Bacteroides	s__Bacteroides_faecis	0.0151	0.0151
g__Bacteroides	s__Bacteroides_faecis	0.0151	0.0151
g__Bacteroides	s__Bacteroides_faecis	0.0151	0.0151
g__Bacteroides	s__Bacteroides_faecis	0.0431	0.0431
g__Bacteroides	s__Bacteroides_faecis	0.0151	0.0151
g__Bacteroides	s__Bacteroides_faecis	0.0151	0.0151
g__Bacteroides	s__Bacteroides_faecis	0.0151	0.0151
g__Bacteroides	s__Bacteroides_faecis	0.0442	0.0442
g__Bacteroides	s__Bacteroides_faecis	0.0146	0.0146
g__Bacteroides	s__Bacteroides_faecis	0.0151	0.0151
g__Bacteroides	s__Bacteroides_faecis	0.0151	0.0151
g__Bacteroides	s__Bacteroides_faecis	0.0151	0.0151
g__Bacteroides	s__Bacteroides_faecis	0.0151	0.0151
g__Bacteroides	s__Bacteroides_faecis	0.0151	0.0151
g__Bacteroides	s__Bacteroides_faecis	0.0151	0.0151
g__Bacteroides	s__Bacteroides_faecis	0.0151	0.0151
g__Bacteroides	s__Bacteroides_faecis	0.0151	0.0151
g__Bacteroides	s__Bacteroides_faecis	0.0151	0.0151
g__Bacteroides	s__Bacteroides_faecis	0.0151	0.0151
g__Bacteroides	s__Bacteroides_faecis	0.0442	0.0442
g__Bacteroides	s__Bacteroides_faecis	0.0151	0.0151
g__Bacteroides	s__Bacteroides_faecis	0.0151	0.0151
g__Bacteroides	s__Bacteroides_faecis	0.0442	0.0442
g__Bacteroides	s__Bacteroides_faecis	0.0151	0.0151
g__Bacteroides	s__Bacteroides_faecis	0.0151	0.0151
g__Bacteroides	s__Bacteroides_faecis	0.0151	0.0151
g__Bacteroides	s__Bacteroides_faecis	0.0151	0.0151
g__Bacteroides	s__Bacteroides_faecis	0.0151	0.0151
g__Bacteroides	s__Bacteroides_faecis	0.0151	0.0151
g__Bacteroides	s__Bacteroides_finegoldii	0.0151	0.0151
g__Bacteroides	s__Bacteroides_finegoldii	0.0151	0.0151
g__Bacteroides	s__Bacteroides_finegoldii	0.0151	0.0151
g__Bacteroides	s__Bacteroides_finegoldii	0.0151	0.0151
g__Bacteroides	s__Bacteroides_finegoldii	0.0151	0.0151
g__Bacteroides	s__Bacteroides_finegoldii	0.0151	0.0151
g__Bacteroides	s__Bacteroides_finegoldii	0.0442	0.0442
g__Bacteroides	s__Bacteroides_finegoldii	0.0151	0.0151
g__Bacteroides	s__Bacteroides_finegoldii	0.0151	0.0151
g__Bacteroides	s__Bacteroides_finegoldii	0.0442	0.0442
g__Bacteroides	s__Bacteroides_finegoldii	0.0151	0.0151
g__Bacteroides	s__Bacteroides_finegoldii	0.0151	0.0151
g__Bacteroides	s__Bacteroides_finegoldii	0.0151	0.0151

g__Bacteroides	s__Bacteroides_finegoldii	0.0151	0.0151
g__Bacteroides	s__Bacteroides_finegoldii	0.0151	0.0151
g__Bacteroides	s__Bacteroides_finegoldii	0.0151	0.0151
g__Bacteroides	s__Bacteroides_finegoldii	0.0151	0.0151
g__Bacteroides	s__Bacteroides_finegoldii	0.0151	0.0151
g__Bacteroides	s__Bacteroides_finegoldii	0.0442	0.0442
g__Bacteroides	s__Bacteroides_finegoldii	0.0151	0.0151
g__Bacteroides	s__Bacteroides_finegoldii	0.0151	0.0151
g__Bacteroides	s__Bacteroides_finegoldii	0.0151	0.0151
g__Bacteroides	s__Bacteroides_finegoldii	0.0151	0.0151
g__Bacteroides	s__Bacteroides_finegoldii	0.0151	0.0151
g__Bacteroides	s__Bacteroides_finegoldii	0.0151	0.0151
g__Bacteroides	s__Bacteroides_finegoldii	0.0151	0.0151
g__Bacteroides	s__Bacteroides_finegoldii	0.0151	0.0151
g__Bacteroides	s__Bacteroides_finegoldii	0.0151	0.0151
g__Bacteroides	s__Bacteroides_finegoldii	0.0151	0.0151
g__Bacteroides	s__Bacteroides_finegoldii	0.0442	0.0442
g__Bacteroides	s__Bacteroides_finegoldii	0.0151	0.0151
g__Bacteroides	s__Bacteroides_finegoldii	0.0151	0.0151
g__Bacteroides	s__Bacteroides_finegoldii	0.0151	0.0151
g__Bacteroides	s__Bacteroides_finegoldii	0.0151	0.0151
g__Bacteroides	s__Bacteroides_finegoldii	0.0442	0.0442
g__Bacteroides	s__Bacteroides_finegoldii	0.0151	0.0151
g__Bacteroides	s__Bacteroides_finegoldii	0.0151	0.0151
g__Bacteroides	s__Bacteroides_finegoldii	0.0442	0.0442
g__Bacteroides	s__Bacteroides_finegoldii	0.0151	0.0151
g__Bacteroides	s__Bacteroides_finegoldii	0.0151	0.0151
g__Bacteroides	s__Bacteroides_finegoldii	0.0146	0.0146
g__Bacteroides	s__Bacteroides_finegoldii	0.0151	0.0151
g__Bacteroides	s__Bacteroides_finegoldii	0.0151	0.0151
g__Bacteroides	s__Bacteroides_finegoldii	0.0151	0.0151
g__Bacteroides	s__Bacteroides_finegoldii	0.0151	0.0151
g__Bacteroides	s__Bacteroides_finegoldii	0.0151	0.0151
g__Bacteroides	s__Bacteroides_finegoldii	0.0151	0.0151
g__Bacteroides	s__Bacteroides_finegoldii	0.0151	0.0151
g__Bacteroides	s__Bacteroides_finegoldii	0.0151	0.0151
g__Bacteroides	s__Bacteroides_finegoldii	0.0151	0.0151
g__Bacteroides	s__Bacteroides_finegoldii	0.0151	0.0151
g__Bacteroides	s__Bacteroides_finegoldii	0.0151	0.0151
g__Bacteroides	s__Bacteroides_finegoldii	0.0151	0.0151
g__Bacteroides	s__Bacteroides_finegoldii	0.0151	0.0151
g__Bacteroides	s__Bacteroides_finegoldii	0.0151	0.0151
g__Bacteroides	s__Bacteroides_finegoldii	0.0442	0.0442

g__Bacteroides	s__Bacteroides_finegoldii	0.0151	0.0151
g__Bacteroides	s__Bacteroides_finegoldii	0.0146	0.0146
g__Bacteroides	s__Bacteroides_finegoldii	0.0442	0.0442
g__Bacteroides	s__Bacteroides_finegoldii	0.0151	0.0151
g__Bacteroides	s__Bacteroides_finegoldii	0.0151	0.0151
g__Bacteroides	s__Bacteroides_finegoldii	0.0151	0.0151
g__Bacteroides	s__Bacteroides_finegoldii	0.0151	0.0151
g__Bacteroides	s__Bacteroides_finegoldii	0.0151	0.0151
g__Bacteroides	s__Bacteroides_finegoldii	0.0151	0.0151
g__Bacteroides	s__Bacteroides_finegoldii	0.0151	0.0151
g__Bacteroides	s__Bacteroides_finegoldii	0.0442	0.0442
g__Bacteroides	s__Bacteroides_finegoldii	0.0151	0.0151
g__Bacteroides	s__Bacteroides_finegoldii	0.0151	0.0151
g__Bacteroides	s__Bacteroides_finegoldii	0.0151	0.0151
g__Bacteroides	s__Bacteroides_finegoldii	0.0151	0.0151
g__Bacteroides	s__Bacteroides_finegoldii	0.0151	0.0151
g__Bacteroides	s__Bacteroides_finegoldii	0.0151	0.0151
g__Bacteroides	s__Bacteroides_finegoldii	0.0151	0.0151
g__Bacteroides	s__Bacteroides_finegoldii	0.0151	0.0151
g__Bacteroides	s__Bacteroides_finegoldii	0.0151	0.0151
g__Bacteroides	s__Bacteroides_finegoldii	0.0151	0.0151
g__Bacteroides	s__Bacteroides_finegoldii	0.0151	0.0151
g__Bacteroides	s__Bacteroides_finegoldii	0.0442	0.0442
g__Bacteroides	s__Bacteroides_finegoldii	0.0151	0.0151
g__Bacteroides	s__Bacteroides_finegoldii	0.0146	0.0146
g__Bacteroides	s__Bacteroides_finegoldii	0.0151	0.0151
g__Bacteroides	s__Bacteroides_finegoldii	0.0151	0.0151
g__Bacteroides	s__Bacteroides_finegoldii	0.0151	0.0151
g__Bacteroides	s__Bacteroides_finegoldii	0.0442	0.0442
g__Bacteroides	s__Bacteroides_finegoldii	0.0151	0.0151
g__Bacteroides	s__Bacteroides_finegoldii	0.0151	0.0151
g__Bacteroides	s__Bacteroides_finegoldii	0.0151	0.0151
g__Bacteroides	s__Bacteroides_finegoldii	0.0151	0.0151
g__Bacteroides	s__Bacteroides_finegoldii	0.0151	0.0151
g__Bacteroides	s__Bacteroides_finegoldii	0.0151	0.0151
g__Bacteroides	s__Bacteroides_finegoldii	0.0151	0.0151
g__Bacteroides	s__Bacteroides_finegoldii	0.0431	0.0431
g__Bacteroides	s__Bacteroides_finegoldii	0.0151	0.0151
g__Bacteroides	s__Bacteroides_finegoldii	0.0442	0.0442
g__Bacteroides	s__Bacteroides_finegoldii	0.0151	0.0151
g__Bacteroides	s__Bacteroides_finegoldii	0.0442	0.0442
g__Bacteroides	s__Bacteroides_finegoldii	0.0151	0.0151
g__Bacteroides	s__Bacteroides_finegoldii	0.0151	0.0151

g__Bacteroides	s__Bacteroides_finegoldii	0.0151	0.0151
g__Bacteroides	s__Bacteroides_finegoldii	0.0151	0.0151
g__Bacteroides	s__Bacteroides_finegoldii	0.0151	0.0151
g__Bacteroides	s__Bacteroides_finegoldii	0.0151	0.0151
g__Bacteroides	s__Bacteroides_finegoldii	0.0151	0.0151
g__Bacteroides	s__Bacteroides_finegoldii	0.0151	0.0151
g__Bacteroides	s__Bacteroides_finegoldii	0.0431	0.0431
g__Bacteroides	s__Bacteroides_finegoldii	0.0151	0.0151
g__Bacteroides	s__Bacteroides_finegoldii	0.0146	0.0146
g__Bacteroides	s__Bacteroides_finegoldii	0.0151	0.0151
g__Bacteroides	s__Bacteroides_finegoldii	0.0151	0.0151
g__Bacteroides	s__Bacteroides_finegoldii	0.0151	0.0151
g__Bacteroides	s__Bacteroides_finegoldii	0.0151	0.0151
g__Bacteroides	s__Bacteroides_finegoldii	0.0151	0.0151
g__Bacteroides	s__Bacteroides_finegoldii	0.0151	0.0151
g__Bacteroides	s__Bacteroides_finegoldii	0.0151	0.0151
g__Bacteroides	s__Bacteroides_finegoldii	0.0442	0.0442
g__Bacteroides	s__Bacteroides_finegoldii	0.0151	0.0151
g__Bacteroides	s__Bacteroides_finegoldii	0.0442	0.0442
g__Bacteroides	s__Bacteroides_finegoldii	0.0151	0.0151
g__Bacteroides	s__Bacteroides_finegoldii	0.0151	0.0151
g__Bacteroides	s__Bacteroides_finegoldii	0.0151	0.0151
g__Bacteroides	s__Bacteroides_finegoldii	0.0442	0.0442
g__Bacteroides	s__Bacteroides_finegoldii	0.0151	0.0151
g__Bacteroides	s__Bacteroides_finegoldii	0.0151	0.0151
g__Bacteroides	s__Bacteroides_finegoldii	0.0151	0.0151
g__Bacteroides	s__Bacteroides_finegoldii	0.0151	0.0151
g__Bacteroides	s__Bacteroides_finegoldii	0.0442	0.0442
g__Bacteroides	s__Bacteroides_finegoldii	0.0151	0.0151
g__Bacteroides	s__Bacteroides_finegoldii	0.0151	0.0151
g__Bacteroides	s__Bacteroides_finegoldii	0.0151	0.0151
g__Bacteroides	s__Bacteroides_finegoldii	0.0151	0.0151
g__Bacteroides	s__Bacteroides_finegoldii	0.0146	0.0146
g__Bacteroides	s__Bacteroides_finegoldii	0.0151	0.0151
g__Bacteroides	s__Bacteroides_finegoldii	0.0151	0.0151
g__Bacteroides	s__Bacteroides_finegoldii	0.0151	0.0151
g__Bacteroides	s__Bacteroides_finegoldii	0.0151	0.0151
g__Bacteroides	s__Bacteroides_finegoldii	0.0151	0.0151
g__Bacteroides	s__Bacteroides_finegoldii	0.0151	0.0151
g__Bacteroides	s__Bacteroides_finegoldii	0.0151	0.0151
g__Bacteroides	s__Bacteroides_finegoldii	0.0151	0.0151
g__Bacteroides	s__Bacteroides_finegoldii	0.0151	0.0151
g__Bacteroides	s__Bacteroides_finegoldii	0.0151	0.0151

g__Bacteroides	s__Bacteroides_finegoldii	0.0151	0.0151
g__Bacteroides	s__Bacteroides_finegoldii	0.0442	0.0442
g__Bacteroides	s__Bacteroides_finegoldii	0.0151	0.0151
g__Bacteroides	s__Bacteroides_finegoldii	0.0151	0.0151
g__Bacteroides	s__Bacteroides_finegoldii	0.0146	0.0146
g__Bacteroides	s__Bacteroides_finegoldii	0.0151	0.0151
g__Bacteroides	s__Bacteroides_finegoldii	0.0133	0.0133
g__Bacteroides	s__Bacteroides_finegoldii	0.0151	0.0151
g__Bacteroides	s__Bacteroides_finegoldii	0.0151	0.0151
g__Bacteroides	s__Bacteroides_finegoldii	0.0151	0.0151
g__Bacteroides	s__Bacteroides_finegoldii	0.0151	0.0151
g__Bacteroides	s__Bacteroides_finegoldii	0.0151	0.0151
g__Bacteroides	s__Bacteroides_finegoldii	0.0151	0.0151
g__Bacteroides	s__Bacteroides_finegoldii	0.0151	0.0151
g__Bacteroides	s__Bacteroides_finegoldii	0.0151	0.0151
g__Bacteroides	s__Bacteroides_finegoldii	0.0151	0.0151
g__Bacteroides	s__Bacteroides_finegoldii	0.0151	0.0151
g__Bacteroides	s__Bacteroides_finegoldii	0.0151	0.0151
g__Bacteroides	s__Bacteroides_finegoldii	0.0151	0.0151
g__Bacteroides	s__Bacteroides_finegoldii	0.0151	0.0151
g__Bacteroides	s__Bacteroides_finegoldii	0.0442	0.0442
g__Bacteroides	s__Bacteroides_finegoldii	0.0151	0.0151
g__Bacteroides	s__Bacteroides_finegoldii	0.0151	0.0151
g__Bacteroides	s__Bacteroides_fragilis	0.0442	0.0442
g__Bacteroides	s__Bacteroides_fragilis	0.0151	0.0151
g__Bacteroides	s__Bacteroides_fragilis	0.0442	0.0442
g__Bacteroides	s__Bacteroides_fragilis	0.0442	0.0442
g__Bacteroides	s__Bacteroides_fragilis	0.0442	0.0442
g__Bacteroides	s__Bacteroides_fragilis	0.0442	0.0442
g__Bacteroides	s__Bacteroides_fragilis	0.0151	0.0151
g__Bacteroides	s__Bacteroides_fragilis	0.0151	0.0151
g__Bacteroides	s__Bacteroides_fragilis	0.0151	0.0151
g__Bacteroides	s__Bacteroides_fragilis	0.0151	0.0151
g__Bacteroides	s__Bacteroides_fragilis	0.0151	0.0151
g__Bacteroides	s__Bacteroides_fragilis	0.0151	0.0151
g__Bacteroides	s__Bacteroides_fragilis	0.0151	0.0151
g__Bacteroides	s__Bacteroides_fragilis	0.0151	0.0151
g__Bacteroides	s__Bacteroides_fragilis	0.0151	0.0151
g__Bacteroides	s__Bacteroides_fragilis	0.0151	0.0151
g__Bacteroides	s__Bacteroides_fragilis	0.0151	0.0151
g__Bacteroides	s__Bacteroides_fragilis	0.0151	0.0151
g__Bacteroides	s__Bacteroides_fragilis	0.0151	0.0151
g__Bacteroides	s__Bacteroides_fragilis	0.0151	0.0151

g__Bacteroides	s__Bacteroides_fragilis	0.0151	0.0151
g__Bacteroides	s__Bacteroides_fragilis	0.0151	0.0151
g__Bacteroides	s__Bacteroides_fragilis	0.0151	0.0151
g__Bacteroides	s__Bacteroides_fragilis	0.0146	0.0146
g__Bacteroides	s__Bacteroides_fragilis	0.0151	0.0151
g__Bacteroides	s__Bacteroides_fragilis	0.0151	0.0151
g__Bacteroides	s__Bacteroides_fragilis	0.0151	0.0151
g__Bacteroides	s__Bacteroides_fragilis	0.0442	0.0442
g__Bacteroides	s__Bacteroides_fragilis	0.0151	0.0151
g__Bacteroides	s__Bacteroides_fragilis	0.0133	0.0133
g__Bacteroides	s__Bacteroides_fragilis	0.0151	0.0151
g__Bacteroides	s__Bacteroides_fragilis	0.0133	0.0133
g__Bacteroides	s__Bacteroides_fragilis	0.0151	0.0151
g__Bacteroides	s__Bacteroides_fragilis	0.0151	0.0151
g__Bacteroides	s__Bacteroides_fragilis	0.0133	0.0133
g__Bacteroides	s__Bacteroides_fragilis	0.0151	0.0151
g__Bacteroides	s__Bacteroides_fragilis	0.0133	0.0133
g__Bacteroides	s__Bacteroides_fragilis	0.0151	0.0151
g__Bacteroides	s__Bacteroides_fragilis	0.0151	0.0151
g__Bacteroides	s__Bacteroides_fragilis	0.0151	0.0151
g__Bacteroides	s__Bacteroides_fragilis	0.0151	0.0151
g__Bacteroides	s__Bacteroides_fragilis	0.0431	0.0431
g__Bacteroides	s__Bacteroides_fragilis	0.0151	0.0151
g__Bacteroides	s__Bacteroides_fragilis	0.0151	0.0151
g__Bacteroides	s__Bacteroides_fragilis	0.0151	0.0151
g__Bacteroides	s__Bacteroides_fragilis	0.0151	0.0151
g__Bacteroides	s__Bacteroides_fragilis	0.0442	0.0442
g__Bacteroides	s__Bacteroides_fragilis	0.0151	0.0151
g__Bacteroides	s__Bacteroides_fragilis	0.0431	0.0431
g__Bacteroides	s__Bacteroides_fragilis	0.0151	0.0151
g__Bacteroides	s__Bacteroides_fragilis	0.0151	0.0151
g__Bacteroides	s__Bacteroides_fragilis	0.0151	0.0151
g__Bacteroides	s__Bacteroides_fragilis	0.0442	0.0442
g__Bacteroides	s__Bacteroides_fragilis	0.0151	0.0151
g__Bacteroides	s__Bacteroides_fragilis	0.0151	0.0151
g__Bacteroides	s__Bacteroides_fragilis	0.0151	0.0151
g__Bacteroides	s__Bacteroides_fragilis	0.0146	0.0146
g__Bacteroides	s__Bacteroides_fragilis	0.0442	0.0442
g__Bacteroides	s__Bacteroides_fragilis	0.0133	0.0133
g__Bacteroides	s__Bacteroides_fragilis	0.0151	0.0151
g__Bacteroides	s__Bacteroides_fragilis	0.0151	0.0151
g__Bacteroides	s__Bacteroides_fragilis	0.0151	0.0151
g__Bacteroides	s__Bacteroides_fragilis	0.0151	0.0151

g__Bacteroides	s__Bacteroides_fragilis	0.0151	0.0151
g__Bacteroides	s__Bacteroides_fragilis	0.0151	0.0151
g__Bacteroides	s__Bacteroides_fragilis	0.0151	0.0151
g__Bacteroides	s__Bacteroides_fragilis	0.0442	0.0442
g__Bacteroides	s__Bacteroides_fragilis	0.0151	0.0151
g__Bacteroides	s__Bacteroides_fragilis	0.0151	0.0151
g__Bacteroides	s__Bacteroides_fragilis	0.0151	0.0151
g__Bacteroides	s__Bacteroides_fragilis	0.0151	0.0151
g__Bacteroides	s__Bacteroides_fragilis	0.0151	0.0151
g__Bacteroides	s__Bacteroides_fragilis	0.0151	0.0151
g__Bacteroides	s__Bacteroides_fragilis	0.0151	0.0151
g__Bacteroides	s__Bacteroides_fragilis	0.0151	0.0151
g__Bacteroides	s__Bacteroides_fragilis	0.0151	0.0151
g__Bacteroides	s__Bacteroides_fragilis	0.0151	0.0151
g__Bacteroides	s__Bacteroides_fragilis	0.0151	0.0151
g__Bacteroides	s__Bacteroides_fragilis	0.0151	0.0151
g__Bacteroides	s__Bacteroides_fragilis	0.0431	0.0431
g__Bacteroides	s__Bacteroides_fragilis	0.0151	0.0151
g__Bacteroides	s__Bacteroides_fragilis	0.0151	0.0151
g__Bacteroides	s__Bacteroides_fragilis	0.0151	0.0151
g__Bacteroides	s__Bacteroides_fragilis	0.0151	0.0151
g__Bacteroides	s__Bacteroides_fragilis	0.0151	0.0151
g__Bacteroides	s__Bacteroides_fragilis	0.0431	0.0431
g__Bacteroides	s__Bacteroides_fragilis	0.0442	0.0442
g__Bacteroides	s__Bacteroides_fragilis	0.0151	0.0151
g__Bacteroides	s__Bacteroides_fragilis	0.0151	0.0151
g__Bacteroides	s__Bacteroides_fragilis	0.0442	0.0442
g__Bacteroides	s__Bacteroides_fragilis	0.0151	0.0151
g__Bacteroides	s__Bacteroides_fragilis	0.0151	0.0151
g__Bacteroides	s__Bacteroides_fragilis	0.0151	0.0151
g__Bacteroides	s__Bacteroides_fragilis	0.0151	0.0151
g__Bacteroides	s__Bacteroides_fragilis	0.0151	0.0151
g__Bacteroides	s__Bacteroides_fragilis	0.0151	0.0151
g__Bacteroides	s__Bacteroides_fragilis	0.0151	0.0151
g__Bacteroides	s__Bacteroides_fragilis	0.0151	0.0151
g__Bacteroides	s__Bacteroides_fragilis	0.0442	0.0442
g__Bacteroides	s__Bacteroides_fragilis	0.0442	0.0442
g__Bacteroides	s__Bacteroides_fragilis	0.0151	0.0151
g__Bacteroides	s__Bacteroides_fragilis	0.0146	0.0146
g__Bacteroides	s__Bacteroides_fragilis	0.0151	0.0151
g__Bacteroides	s__Bacteroides_fragilis	0.0151	0.0151
g__Bacteroides	s__Bacteroides_fragilis	0.0151	0.0151
g__Bacteroides	s__Bacteroides_fragilis	0.0431	0.0431

g__Bacteroides	s__Bacteroides_fragilis	0.0151	0.0151
g__Bacteroides	s__Bacteroides_fragilis	0.0151	0.0151
g__Bacteroides	s__Bacteroides_fragilis	0.0151	0.0151
g__Bacteroides	s__Bacteroides_fragilis	0.0151	0.0151
g__Bacteroides	s__Bacteroides_fragilis	0.0151	0.0151
g__Bacteroides	s__Bacteroides_fragilis	0.0431	0.0431
g__Bacteroides	s__Bacteroides_fragilis	0.0151	0.0151
g__Bacteroides	s__Bacteroides_fragilis	0.0151	0.0151
g__Bacteroides	s__Bacteroides_fragilis	0.0151	0.0151
g__Bacteroides	s__Bacteroides_fragilis	0.0151	0.0151
g__Bacteroides	s__Bacteroides_fragilis	0.0151	0.0151
g__Bacteroides	s__Bacteroides_fragilis	0.0151	0.0151
g__Bacteroides	s__Bacteroides_fragilis	0.0151	0.0151
g__Bacteroides	s__Bacteroides_fragilis	0.0151	0.0151
g__Bacteroides	s__Bacteroides_fragilis	0.0151	0.0151
g__Bacteroides	s__Bacteroides_fragilis	0.0151	0.0151
g__Bacteroides	s__Bacteroides_fragilis	0.0151	0.0151
g__Bacteroides	s__Bacteroides_fragilis	0.0151	0.0151
g__Bacteroides	s__Bacteroides_fragilis	0.0146	0.0146
g__Bacteroides	s__Bacteroides_fragilis	0.0151	0.0151
g__Bacteroides	s__Bacteroides_fragilis	0.0151	0.0151
g__Bacteroides	s__Bacteroides_fragilis	0.0151	0.0151
g__Bacteroides	s__Bacteroides_fragilis	0.0151	0.0151
g__Bacteroides	s__Bacteroides_fragilis	0.0151	0.0151
g__Bacteroides	s__Bacteroides_fragilis	0.0151	0.0151
g__Bacteroides	s__Bacteroides_fragilis	0.0151	0.0151
g__Bacteroides	s__Bacteroides_fragilis	0.0151	0.0151
g__Bacteroides	s__Bacteroides_fragilis	0.0151	0.0151
g__Bacteroides	s__Bacteroides_fragilis	0.0146	0.0146
g__Bacteroides	s__Bacteroides_fragilis	0.0151	0.0151
g__Bacteroides	s__Bacteroides_fragilis	0.0151	0.0151
g__Bacteroides	s__Bacteroides_fragilis	0.0151	0.0151
g__Bacteroides	s__Bacteroides_fragilis	0.0151	0.0151
g__Bacteroides	s__Bacteroides_fragilis	0.0151	0.0151
g__Bacteroides	s__Bacteroides_fragilis	0.0442	0.0442
g__Bacteroides	s__Bacteroides_fragilis	0.0151	0.0151
g__Bacteroides	s__Bacteroides_fragilis	0.0151	0.0151
g__Bacteroides	s__Bacteroides_fragilis	0.0146	0.0146
g__Bacteroides	s__Bacteroides_fragilis	0.0151	0.0151
g__Bacteroides	s__Bacteroides_fragilis	0.0151	0.0151
g__Bacteroides	s__Bacteroides_fragilis	0.0151	0.0151
g__Bacteroides	s__Bacteroides_fragilis	0.0151	0.0151
g__Bacteroides	s__Bacteroides_fragilis	0.0151	0.0151

g__Bacteroides	s__Bacteroides_fragilis	0.0151	0.0151
g__Bacteroides	s__Bacteroides_fragilis	0.0151	0.0151
g__Bacteroides	s__Bacteroides_fragilis	0.0151	0.0151
g__Bacteroides	s__Bacteroides_fragilis	0.0151	0.0151
g__Bacteroides	s__Bacteroides_fragilis	0.0151	0.0151
g__Bacteroides	s__Bacteroides_fragilis	0.0151	0.0151
g__Bacteroides	s__Bacteroides_fragilis	0.0151	0.0151
g__Bacteroides	s__Bacteroides_fragilis	0.0151	0.0151
g__Bacteroides	s__Bacteroides_fragilis	0.0442	0.0442
g__Bacteroides	s__Bacteroides_fragilis	0.0151	0.0151
g__Bacteroides	s__Bacteroides_fragilis	0.0151	0.0151
g__Bacteroides	s__Bacteroides_fragilis	0.0146	0.0146
g__Bacteroides	s__Bacteroides_fragilis	0.0442	0.0442
g__Bacteroides	s__Bacteroides_fragilis	0.0151	0.0151
g__Bacteroides	s__Bacteroides_fragilis	0.0151	0.0151
g__Bacteroides	s__Bacteroides_fragilis	0.0151	0.0151
g__Bacteroides	s__Bacteroides_fragilis	0.0151	0.0151
g__Bacteroides	s__Bacteroides_fragilis	0.0151	0.0151
g__Bacteroides	s__Bacteroides_fragilis	0.0151	0.0151
g__Bacteroides	s__Bacteroides_fragilis	0.0151	0.0151
g__Bacteroides	s__Bacteroides_fragilis	0.0151	0.0151
g__Bacteroides	s__Bacteroides_fragilis	0.0151	0.0151
g__Bacteroides	s__Bacteroides_fragilis	0.0151	0.0151
g__Bacteroides	s__Bacteroides_fragilis	0.0151	0.0151
g__Bacteroides	s__Bacteroides_fragilis	0.0151	0.0151
g__Bacteroides	s__Bacteroides_fragilis	0.0442	0.0442
g__Bacteroides	s__Bacteroides_fragilis	0.0151	0.0151
g__Bacteroides	s__Bacteroides_fragilis	0.0151	0.0151
g__Bacteroides	s__Bacteroides_fragilis	0.0442	0.0442
g__Bacteroides	s__Bacteroides_fragilis	0.0151	0.0151
g__Bacteroides	s__Bacteroides_fragilis	0.0442	0.0442
g__Bacteroides	s__Bacteroides_fragilis	0.0442	0.0442
g__Bacteroides	s__Bacteroides_fragilis	0.0151	0.0151
g__Bacteroides	s__Bacteroides_fragilis	0.0151	0.0151
g__Bacteroides	s__Bacteroides_fragilis	0.0151	0.0151
g__Bacteroides	s__Bacteroides_fragilis	0.0151	0.0151
g__Bacteroides	s__Bacteroides_fragilis	0.0151	0.0151
g__Bacteroides	s__Bacteroides_fragilis	0.0151	0.0151
g__Bacteroides	s__Bacteroides_fragilis	0.0442	0.0442
g__Bacteroides	s__Bacteroides_fragilis	0.0151	0.0151
g__Bacteroides	s__Bacteroides_fragilis	0.0151	0.0151
g__Bacteroides	s__Bacteroides_fragilis	0.0151	0.0151
g__Bacteroides	s__Bacteroides_fragilis	0.0133	0.0133

g__Bacteroides	s__Bacteroides_fragilis	0.0151	0.0151
g__Bacteroides	s__Bacteroides_fragilis	0.0151	0.0151
g__Bacteroides	s__Bacteroides_graminisolvens	0.0151	0.0151
g__Bacteroides	s__Bacteroides_helcogenes	0.0151	0.0151
g__Bacteroides	s__Bacteroides_helcogenes	0.0151	0.0151
g__Bacteroides	s__Bacteroides_helcogenes	0.0151	0.0151
g__Bacteroides	s__Bacteroides_helcogenes	0.0151	0.0151
g__Bacteroides	s__Bacteroides_helcogenes	0.0151	0.0151
g__Bacteroides	s__Bacteroides_helcogenes	0.0151	0.0151
g__Bacteroides	s__Bacteroides_helcogenes	0.0151	0.0151
g__Bacteroides	s__Bacteroides_helcogenes	0.0151	0.0151
g__Bacteroides	s__Bacteroides_helcogenes	0.0151	0.0151
g__Bacteroides	s__Bacteroides_helcogenes	0.0442	0.0442
g__Bacteroides	s__Bacteroides_helcogenes	0.0431	0.0431
g__Bacteroides	s__Bacteroides_helcogenes	0.0442	0.0442
g__Bacteroides	s__Bacteroides_helcogenes	0.0151	0.0151
g__Bacteroides	s__Bacteroides_helcogenes	0.0151	0.0151
g__Bacteroides	s__Bacteroides_helcogenes	0.0151	0.0151
g__Bacteroides	s__Bacteroides_heparinolyticus	0.0442	0.0442
g__Bacteroides	s__Bacteroides_heparinolyticus	0.0151	0.0151
g__Bacteroides	s__Bacteroides_heparinolyticus	0.0151	0.0151
g__Bacteroides	s__Bacteroides_heparinolyticus	0.0151	0.0151
g__Bacteroides	s__Bacteroides_heparinolyticus	0.0151	0.0151
g__Bacteroides	s__Bacteroides_heparinolyticus	0.0442	0.0442
g__Bacteroides	s__Bacteroides_heparinolyticus	0.0151	0.0151
g__Bacteroides	s__Bacteroides_intestinalis	0.0442	0.0442
g__Bacteroides	s__Bacteroides_intestinalis	0.0151	0.0151
g__Bacteroides	s__Bacteroides_intestinalis	0.0151	0.0151
g__Bacteroides	s__Bacteroides_intestinalis	0.0151	0.0151
g__Bacteroides	s__Bacteroides_intestinalis	0.0151	0.0151
g__Bacteroides	s__Bacteroides_intestinalis	0.0151	0.0151
g__Bacteroides	s__Bacteroides_intestinalis	0.0442	0.0442
g__Bacteroides	s__Bacteroides_intestinalis	0.0442	0.0442
g__Bacteroides	s__Bacteroides_intestinalis	0.0442	0.0442
g__Bacteroides	s__Bacteroides_intestinalis	0.0151	0.0151
g__Bacteroides	s__Bacteroides_intestinalis	0.0442	0.0442
g__Bacteroides	s__Bacteroides_intestinalis	0.0151	0.0151
g__Bacteroides	s__Bacteroides_intestinalis	0.0151	0.0151
g__Bacteroides	s__Bacteroides_intestinalis	0.0431	0.0431
g__Bacteroides	s__Bacteroides_intestinalis	0.0442	0.0442
g__Bacteroides	s__Bacteroides_intestinalis	0.0442	0.0442
g__Bacteroides	s__Bacteroides_intestinalis	0.0442	0.0442
g__Bacteroides	s__Bacteroides_intestinalis	0.0151	0.0151

g__Bacteroides	s__Bacteroides_intestinalis	0.0151	0.0151
g__Bacteroides	s__Bacteroides_intestinalis	0.0151	0.0151
g__Bacteroides	s__Bacteroides_intestinalis	0.0442	0.0442
g__Bacteroides	s__Bacteroides_intestinalis	0.0431	0.0431
g__Bacteroides	s__Bacteroides_intestinalis	0.0151	0.0151
g__Bacteroides	s__Bacteroides_intestinalis	0.0151	0.0151
g__Bacteroides	s__Bacteroides_intestinalis	0.04	0.04
g__Bacteroides	s__Bacteroides_intestinalis	0.0151	0.0151
g__Bacteroides	s__Bacteroides_intestinalis	0.0151	0.0151
g__Bacteroides	s__Bacteroides_intestinalis	0.0442	0.0442
g__Bacteroides	s__Bacteroides_intestinalis	0.0442	0.0442
g__Bacteroides	s__Bacteroides_intestinalis	0.0442	0.0442
g__Bacteroides	s__Bacteroides_intestinalis	0.0442	0.0442
g__Bacteroides	s__Bacteroides_intestinalis	0.0151	0.0151
g__Bacteroides	s__Bacteroides_intestinalis	0.0151	0.0151
g__Bacteroides	s__Bacteroides_intestinalis	0.0151	0.0151
g__Bacteroides	s__Bacteroides_intestinalis	0.0442	0.0442
g__Bacteroides	s__Bacteroides_intestinalis	0.0151	0.0151
g__Bacteroides	s__Bacteroides_intestinalis	0.0151	0.0151
g__Bacteroides	s__Bacteroides_intestinalis	0.0151	0.0151
g__Bacteroides	s__Bacteroides_intestinalis	0.0151	0.0151
g__Bacteroides	s__Bacteroides_intestinalis	0.0151	0.0151
g__Bacteroides	s__Bacteroides_intestinalis	0.0442	0.0442
g__Bacteroides	s__Bacteroides_intestinalis	0.0151	0.0151
g__Bacteroides	s__Bacteroides_intestinalis	0.0431	0.0431
g__Bacteroides	s__Bacteroides_intestinalis	0.0151	0.0151
g__Bacteroides	s__Bacteroides_intestinalis	0.0151	0.0151
g__Bacteroides	s__Bacteroides_intestinalis	0.0442	0.0442
g__Bacteroides	s__Bacteroides_intestinalis	0.0151	0.0151
g__Bacteroides	s__Bacteroides_intestinalis	0.0151	0.0151
g__Bacteroides	s__Bacteroides_intestinalis	0.0442	0.0442
g__Bacteroides	s__Bacteroides_intestinalis	0.0151	0.0151
g__Bacteroides	s__Bacteroides_intestinalis	0.0151	0.0151
g__Bacteroides	s__Bacteroides_intestinalis	0.0151	0.0151
g__Bacteroides	s__Bacteroides_intestinalis	0.0151	0.0151
g__Bacteroides	s__Bacteroides_intestinalis	0.0151	0.0151
g__Bacteroides	s__Bacteroides_intestinalis	0.0442	0.0442
g__Bacteroides	s__Bacteroides_intestinalis	0.0151	0.0151
g__Bacteroides	s__Bacteroides_intestinalis	0.0151	0.0151
g__Bacteroides	s__Bacteroides_intestinalis	0.0151	0.0151
g__Bacteroides	s__Bacteroides_intestinalis	0.0146	0.0146
g__Bacteroides	s__Bacteroides_intestinalis	0.0442	0.0442
g__Bacteroides	s__Bacteroides_intestinalis	0.0151	0.0151

g__Bacteroides	s__Bacteroides_intestinalis	0.0442	0.0442
g__Bacteroides	s__Bacteroides_intestinalis	0.0151	0.0151
g__Bacteroides	s__Bacteroides_intestinalis	0.0151	0.0151
g__Bacteroides	s__Bacteroides_intestinalis	0.0151	0.0151
g__Bacteroides	s__Bacteroides_intestinalis	0.0442	0.0442
g__Bacteroides	s__Bacteroides_intestinalis	0.0431	0.0431
g__Bacteroides	s__Bacteroides_intestinalis	0.0151	0.0151
g__Bacteroides	s__Bacteroides_intestinalis	0.0442	0.0442
g__Bacteroides	s__Bacteroides_intestinalis	0.0151	0.0151
g__Bacteroides	s__Bacteroides_intestinalis	0.0151	0.0151
g__Bacteroides	s__Bacteroides_intestinalis	0.0442	0.0442
g__Bacteroides	s__Bacteroides_intestinalis	0.0442	0.0442
g__Bacteroides	s__Bacteroides_intestinalis	0.0151	0.0151
g__Bacteroides	s__Bacteroides_intestinalis	0.0442	0.0442
g__Bacteroides	s__Bacteroides_intestinalis	0.0151	0.0151
g__Bacteroides	s__Bacteroides_intestinalis	0.0151	0.0151
g__Bacteroides	s__Bacteroides_intestinalis	0.0442	0.0442
g__Bacteroides	s__Bacteroides_intestinalis	0.0151	0.0151
g__Bacteroides	s__Bacteroides_intestinalis	0.0442	0.0442
g__Bacteroides	s__Bacteroides_intestinalis	0.0442	0.0442
g__Bacteroides	s__Bacteroides_intestinalis	0.0442	0.0442
g__Bacteroides	s__Bacteroides_intestinalis	0.0442	0.0442
g__Bacteroides	s__Bacteroides_intestinalis	0.0442	0.0442
g__Bacteroides	s__Bacteroides_intestinalis	0.0442	0.0442
g__Bacteroides	s__Bacteroides_intestinalis	0.0151	0.0151
g__Bacteroides	s__Bacteroides_intestinalis	0.0151	0.0151
g__Bacteroides	s__Bacteroides_intestinalis	0.0151	0.0151
g__Bacteroides	s__Bacteroides_intestinalis	0.0151	0.0151
g__Bacteroides	s__Bacteroides_nordii	0.0151	0.0151
g__Bacteroides	s__Bacteroides_nordii	0.0151	0.0151
g__Bacteroides	s__Bacteroides_nordii	0.0442	0.0442
g__Bacteroides	s__Bacteroides_nordii	0.0108	0.0108
g__Bacteroides	s__Bacteroides_nordii	0.0151	0.0151
g__Bacteroides	s__Bacteroides_nordii	0.0442	0.0442
g__Bacteroides	s__Bacteroides_nordii	0.0151	0.0151
g__Bacteroides	s__Bacteroides_nordii	0.0151	0.0151
g__Bacteroides	s__Bacteroides_nordii	0.0151	0.0151
g__Bacteroides	s__Bacteroides_nordii	0.0151	0.0151
g__Bacteroides	s__Bacteroides_nordii	0.0442	0.0442
g__Bacteroides	s__Bacteroides_nordii	0.0146	0.0146
g__Bacteroides	s__Bacteroides_nordii	0.0151	0.0151
g__Bacteroides	s__Bacteroides_ovatus	0.0151	0.0151
g__Bacteroides	s__Bacteroides_ovatus	0.0151	0.0151

g__Bacteroides	s__Bacteroides_ovatus	0.0151	0.0151
g__Bacteroides	s__Bacteroides_ovatus	0.0151	0.0151
g__Bacteroides	s__Bacteroides_ovatus	0.0151	0.0151
g__Bacteroides	s__Bacteroides_ovatus	0.0151	0.0151
g__Bacteroides	s__Bacteroides_ovatus	0.0151	0.0151
g__Bacteroides	s__Bacteroides_ovatus	0.0151	0.0151
g__Bacteroides	s__Bacteroides_ovatus	0.0151	0.0151
g__Bacteroides	s__Bacteroides_ovatus	0.0151	0.0151
g__Bacteroides	s__Bacteroides_ovatus	0.0151	0.0151
g__Bacteroides	s__Bacteroides_ovatus	0.0151	0.0151
g__Bacteroides	s__Bacteroides_ovatus	0.0151	0.0151
g__Bacteroides	s__Bacteroides_ovatus	0.0146	0.0146
g__Bacteroides	s__Bacteroides_ovatus	0.0151	0.0151
g__Bacteroides	s__Bacteroides_ovatus	0.0179	0.0179
g__Bacteroides	s__Bacteroides_ovatus	0.0151	0.0151
g__Bacteroides	s__Bacteroides_ovatus	0.0151	0.0151
g__Bacteroides	s__Bacteroides_ovatus	0.0151	0.0151
g__Bacteroides	s__Bacteroides_ovatus	0.0151	0.0151
g__Bacteroides	s__Bacteroides_ovatus	0.0133	0.0133
g__Bacteroides	s__Bacteroides_ovatus	0.0151	0.0151
g__Bacteroides	s__Bacteroides_ovatus	0.0151	0.0151
g__Bacteroides	s__Bacteroides_ovatus	0.0151	0.0151
g__Bacteroides	s__Bacteroides_ovatus	0.0151	0.0151
g__Bacteroides	s__Bacteroides_ovatus	0.0151	0.0151
g__Bacteroides	s__Bacteroides_ovatus	0.0151	0.0151
g__Bacteroides	s__Bacteroides_ovatus	0.0151	0.0151
g__Bacteroides	s__Bacteroides_ovatus	0.0151	0.0151
g__Bacteroides	s__Bacteroides_ovatus	0.0151	0.0151
g__Bacteroides	s__Bacteroides_ovatus	0.0151	0.0151
g__Bacteroides	s__Bacteroides_ovatus	0.0146	0.0146
g__Bacteroides	s__Bacteroides_ovatus	0.0151	0.0151
g__Bacteroides	s__Bacteroides_ovatus	0.0151	0.0151
g__Bacteroides	s__Bacteroides_ovatus	0.0151	0.0151
g__Bacteroides	s__Bacteroides_ovatus	0.0442	0.0442
g__Bacteroides	s__Bacteroides_ovatus	0.0151	0.0151
g__Bacteroides	s__Bacteroides_ovatus	0.0151	0.0151
g__Bacteroides	s__Bacteroides_ovatus	0.0151	0.0151
g__Bacteroides	s__Bacteroides_ovatus	0.0151	0.0151
g__Bacteroides	s__Bacteroides_ovatus	0.0442	0.0442
g__Bacteroides	s__Bacteroides_ovatus	0.0151	0.0151
g__Bacteroides	s__Bacteroides_ovatus	0.0151	0.0151
g__Bacteroides	s__Bacteroides_ovatus	0.0151	0.0151
g__Bacteroides	s__Bacteroides_ovatus	0.0151	0.0151

g__Bacteroides	s__Bacteroides_ovatus	0.0151	0.0151
g__Bacteroides	s__Bacteroides_ovatus	0.0151	0.0151
g__Bacteroides	s__Bacteroides_ovatus	0.0151	0.0151
g__Bacteroides	s__Bacteroides_ovatus	0.0151	0.0151
g__Bacteroides	s__Bacteroides_ovatus	0.0151	0.0151
g__Bacteroides	s__Bacteroides_ovatus	0.0151	0.0151
g__Bacteroides	s__Bacteroides_ovatus	0.0151	0.0151
g__Bacteroides	s__Bacteroides_ovatus	0.0151	0.0151
g__Bacteroides	s__Bacteroides_ovatus	0.0151	0.0151
g__Bacteroides	s__Bacteroides_ovatus	0.0151	0.0151
g__Bacteroides	s__Bacteroides_ovatus	0.0151	0.0151
g__Bacteroides	s__Bacteroides_ovatus	0.0442	0.0442
g__Bacteroides	s__Bacteroides_ovatus	0.0151	0.0151
g__Bacteroides	s__Bacteroides_ovatus	0.0151	0.0151
g__Bacteroides	s__Bacteroides_ovatus	0.0151	0.0151
g__Bacteroides	s__Bacteroides_ovatus	0.0151	0.0151
g__Bacteroides	s__Bacteroides_ovatus	0.0151	0.0151
g__Bacteroides	s__Bacteroides_ovatus	0.0151	0.0151
g__Bacteroides	s__Bacteroides_ovatus	0.0151	0.0151
g__Bacteroides	s__Bacteroides_ovatus	0.0151	0.0151
g__Bacteroides	s__Bacteroides_ovatus	0.0151	0.0151
g__Bacteroides	s__Bacteroides_ovatus	0.0431	0.0431
g__Bacteroides	s__Bacteroides_ovatus	0.0151	0.0151
g__Bacteroides	s__Bacteroides_ovatus	0.0146	0.0146
g__Bacteroides	s__Bacteroides_ovatus	0.0151	0.0151
g__Bacteroides	s__Bacteroides_ovatus	0.0151	0.0151
g__Bacteroides	s__Bacteroides_ovatus	0.0151	0.0151
g__Bacteroides	s__Bacteroides_ovatus	0.0151	0.0151
g__Bacteroides	s__Bacteroides_ovatus	0.0151	0.0151
g__Bacteroides	s__Bacteroides_ovatus	0.0151	0.0151
g__Bacteroides	s__Bacteroides_ovatus	0.0151	0.0151
g__Bacteroides	s__Bacteroides_ovatus	0.0151	0.0151
g__Bacteroides	s__Bacteroides_ovatus	0.0151	0.0151
g__Bacteroides	s__Bacteroides_ovatus	0.0151	0.0151
g__Bacteroides	s__Bacteroides_ovatus	0.0151	0.0151
g__Bacteroides	s__Bacteroides_ovatus	0.0151	0.0151
g__Bacteroides	s__Bacteroides_ovatus	0.0151	0.0151
g__Bacteroides	s__Bacteroides_ovatus	0.0151	0.0151
g__Bacteroides	s__Bacteroides_ovatus	0.0151	0.0151
g__Bacteroides	s__Bacteroides_ovatus	0.0151	0.0151
g__Bacteroides	s__Bacteroides_ovatus	0.0151	0.0151
g__Bacteroides	s__Bacteroides_ovatus	0.0151	0.0151
g__Bacteroides	s__Bacteroides_ovatus	0.0151	0.0151

g__Bacteroides	s__Bacteroides_ovatus	0.0151	0.0151
g__Bacteroides	s__Bacteroides_ovatus	0.0151	0.0151
g__Bacteroides	s__Bacteroides_ovatus	0.0442	0.0442
g__Bacteroides	s__Bacteroides_ovatus	0.0151	0.0151
g__Bacteroides	s__Bacteroides_ovatus	0.0151	0.0151
g__Bacteroides	s__Bacteroides_ovatus	0.0151	0.0151
g__Bacteroides	s__Bacteroides_ovatus	0.0442	0.0442
g__Bacteroides	s__Bacteroides_ovatus	0.0151	0.0151
g__Bacteroides	s__Bacteroides_ovatus	0.0151	0.0151
g__Bacteroides	s__Bacteroides_ovatus	0.0151	0.0151
g__Bacteroides	s__Bacteroides_ovatus	0.0442	0.0442
g__Bacteroides	s__Bacteroides_ovatus	0.0151	0.0151
g__Bacteroides	s__Bacteroides_ovatus	0.0151	0.0151
g__Bacteroides	s__Bacteroides_ovatus	0.0151	0.0151
g__Bacteroides	s__Bacteroides_ovatus	0.0151	0.0151
g__Bacteroides	s__Bacteroides_ovatus	0.0151	0.0151
g__Bacteroides	s__Bacteroides_ovatus	0.0151	0.0151
g__Bacteroides	s__Bacteroides_ovatus	0.0151	0.0151
g__Bacteroides	s__Bacteroides_ovatus	0.0151	0.0151
g__Bacteroides	s__Bacteroides_ovatus	0.0151	0.0151
g__Bacteroides	s__Bacteroides_ovatus	0.0151	0.0151
g__Bacteroides	s__Bacteroides_ovatus	0.0151	0.0151
g__Bacteroides	s__Bacteroides_ovatus	0.0146	0.0146
g__Bacteroides	s__Bacteroides_ovatus	0.0151	0.0151
g__Bacteroides	s__Bacteroides_ovatus	0.0151	0.0151
g__Bacteroides	s__Bacteroides_ovatus	0.0151	0.0151
g__Bacteroides	s__Bacteroides_ovatus	0.0151	0.0151
g__Bacteroides	s__Bacteroides_ovatus	0.0151	0.0151
g__Bacteroides	s__Bacteroides_ovatus	0.0442	0.0442
g__Bacteroides	s__Bacteroides_ovatus	0.0151	0.0151
g__Bacteroides	s__Bacteroides_ovatus	0.0151	0.0151
g__Bacteroides	s__Bacteroides_ovatus	0.0151	0.0151
g__Bacteroides	s__Bacteroides_ovatus	0.0151	0.0151
g__Bacteroides	s__Bacteroides_ovatus	0.0133	0.0133
g__Bacteroides	s__Bacteroides_ovatus	0.0151	0.0151
g__Bacteroides	s__Bacteroides_ovatus	0.0151	0.0151
g__Bacteroides	s__Bacteroides_ovatus	0.0151	0.0151
g__Bacteroides	s__Bacteroides_ovatus	0.0151	0.0151
g__Bacteroides	s__Bacteroides_ovatus	0.0151	0.0151
g__Bacteroides	s__Bacteroides_ovatus	0.0151	0.0151
g__Bacteroides	s__Bacteroides_ovatus	0.0151	0.0151
g__Bacteroides	s__Bacteroides_ovatus	0.0151	0.0151
g__Bacteroides	s__Bacteroides_ovatus	0.0151	0.0151

g__Bacteroides	s__Bacteroides_ovatus	0.0151	0.0151
g__Bacteroides	s__Bacteroides_ovatus	0.0151	0.0151
g__Bacteroides	s__Bacteroides_ovatus	0.0151	0.0151
g__Bacteroides	s__Bacteroides_ovatus	0.0151	0.0151
g__Bacteroides	s__Bacteroides_ovatus	0.0151	0.0151
g__Bacteroides	s__Bacteroides_ovatus	0.0151	0.0151
g__Bacteroides	s__Bacteroides_ovatus	0.0151	0.0151
g__Bacteroides	s__Bacteroides_ovatus	0.0151	0.0151
g__Bacteroides	s__Bacteroides_ovatus	0.0151	0.0151
g__Bacteroides	s__Bacteroides_ovatus	0.0151	0.0151
g__Bacteroides	s__Bacteroides_ovatus	0.0151	0.0151
g__Bacteroides	s__Bacteroides_ovatus	0.0146	0.0146
g__Bacteroides	s__Bacteroides_ovatus	0.0151	0.0151
g__Bacteroides	s__Bacteroides_ovatus	0.0151	0.0151
g__Bacteroides	s__Bacteroides_ovatus	0.0151	0.0151
g__Bacteroides	s__Bacteroides_ovatus	0.0151	0.0151
g__Bacteroides	s__Bacteroides_ovatus	0.0151	0.0151
g__Bacteroides	s__Bacteroides_ovatus	0.0151	0.0151
g__Bacteroides	s__Bacteroides_ovatus	0.0151	0.0151
g__Bacteroides	s__Bacteroides_ovatus	0.0151	0.0151
g__Bacteroides	s__Bacteroides_ovatus	0.0151	0.0151
g__Bacteroides	s__Bacteroides_ovatus	0.0151	0.0151
g__Bacteroides	s__Bacteroides_ovatus	0.0151	0.0151
g__Bacteroides	s__Bacteroides_ovatus	0.0151	0.0151
g__Bacteroides	s__Bacteroides_ovatus	0.0146	0.0146
g__Bacteroides	s__Bacteroides_ovatus	0.0151	0.0151
g__Bacteroides	s__Bacteroides_ovatus	0.0151	0.0151
g__Bacteroides	s__Bacteroides_ovatus	0.0151	0.0151
g__Bacteroides	s__Bacteroides_ovatus	0.0151	0.0151
g__Bacteroides	s__Bacteroides_ovatus	0.0151	0.0151
g__Bacteroides	s__Bacteroides_ovatus	0.0151	0.0151
g__Bacteroides	s__Bacteroides_ovatus	0.0151	0.0151
g__Bacteroides	s__Bacteroides_ovatus	0.0151	0.0151
g__Bacteroides	s__Bacteroides_ovatus	0.0151	0.0151
g__Bacteroides	s__Bacteroides_ovatus	0.0151	0.0151
g__Bacteroides	s__Bacteroides_ovatus	0.0151	0.0151
g__Bacteroides	s__Bacteroides_ovatus	0.0151	0.0151
g__Bacteroides	s__Bacteroides_ovatus	0.0442	0.0442
g__Bacteroides	s__Bacteroides_ovatus	0.0151	0.0151
g__Bacteroides	s__Bacteroides_ovatus	0.0442	0.0442
g__Bacteroides	s__Bacteroides_ovatus	0.0151	0.0151
g__Bacteroides	s__Bacteroides_ovatus	0.0151	0.0151
g__Bacteroides	s__Bacteroides_ovatus	0.0151	0.0151

g__Bacteroides	s__Bacteroides_ovatus	0.0151	0.0151
g__Bacteroides	s__Bacteroides_ovatus	0.0151	0.0151
g__Bacteroides	s__Bacteroides_ovatus	0.0151	0.0151
g__Bacteroides	s__Bacteroides_ovatus	0.0151	0.0151
g__Bacteroides	s__Bacteroides_ovatus	0.0151	0.0151
g__Bacteroides	s__Bacteroides_ovatus	0.0151	0.0151
g__Bacteroides	s__Bacteroides_ovatus	0.0151	0.0151
g__Bacteroides	s__Bacteroides_ovatus	0.0151	0.0151
g__Bacteroides	s__Bacteroides_ovatus	0.0151	0.0151
g__Bacteroides	s__Bacteroides_ovatus	0.0151	0.0151
g__Bacteroides	s__Bacteroides_ovatus	0.0151	0.0151
g__Bacteroides	s__Bacteroides_ovatus	0.0151	0.0151
g__Bacteroides	s__Bacteroides_ovatus	0.0151	0.0151
g__Bacteroides	s__Bacteroides_ovatus	0.0151	0.0151
g__Bacteroides	s__Bacteroides_ovatus	0.0151	0.0151
g__Bacteroides	s__Bacteroides_ovatus	0.0151	0.0151
g__Bacteroides	s__Bacteroides_ovatus	0.0151	0.0151
g__Bacteroides	s__Bacteroides_ovatus	0.0151	0.0151
g__Bacteroides	s__Bacteroides_ovatus	0.0151	0.0151
g__Bacteroides	s__Bacteroides_ovatus	0.0442	0.0442
g__Bacteroides	s__Bacteroides_ovatus	0.0151	0.0151
g__Bacteroides	s__Bacteroides_ovatus	0.0151	0.0151
g__Bacteroides	s__Bacteroides_ovatus	0.0151	0.0151
g__Bacteroides	s__Bacteroides_ovatus	0.0151	0.0151
g__Bacteroides	s__Bacteroides_ovatus	0.0151	0.0151
g__Bacteroides	s__Bacteroides_ovatus	0.0151	0.0151
g__Bacteroides	s__Bacteroides_ovatus	0.0151	0.0151
g__Bacteroides	s__Bacteroides_ovatus	0.0151	0.0151
g__Bacteroides	s__Bacteroides_ovatus	0.0151	0.0151
g__Bacteroides	s__Bacteroides_ovatus	0.0151	0.0151
g__Bacteroides	s__Bacteroides_ovatus	0.0151	0.0151
g__Bacteroides	s__Bacteroides_ovatus	0.0151	0.0151
g__Bacteroides	s__Bacteroides_ovatus	0.0151	0.0151
g__Bacteroides	s__Bacteroides_ovatus	0.0151	0.0151
g__Bacteroides	s__Bacteroides_ovatus	0.0431	0.0431
g__Bacteroides	s__Bacteroides_ovatus	0.0151	0.0151
g__Bacteroides	s__Bacteroides_ovatus	0.0151	0.0151
g__Bacteroides	s__Bacteroides_ovatus	0.0151	0.0151
g__Bacteroides	s__Bacteroides_ovatus	0.0151	0.0151
g__Bacteroides	s__Bacteroides_ovatus	0.0151	0.0151
g__Bacteroides	s__Bacteroides_ovatus	0.0442	0.0442
g__Bacteroides	s__Bacteroides_ovatus	0.0151	0.0151
g__Bacteroides	s__Bacteroides_ovatus	0.0151	0.0151

g__Bacteroides	s__Bacteroides_ovatus	0.0151	0.0151
g__Bacteroides	s__Bacteroides_ovatus	0.0151	0.0151
g__Bacteroides	s__Bacteroides_ovatus	0.0151	0.0151
g__Bacteroides	s__Bacteroides_ovatus	0.0151	0.0151
g__Bacteroides	s__Bacteroides_ovatus	0.0151	0.0151
g__Bacteroides	s__Bacteroides_ovatus	0.0151	0.0151
g__Bacteroides	s__Bacteroides_ovatus	0.0151	0.0151
g__Bacteroides	s__Bacteroides_ovatus	0.0151	0.0151
g__Bacteroides	s__Bacteroides_ovatus	0.0146	0.0146
g__Bacteroides	s__Bacteroides_ovatus	0.0151	0.0151
g__Bacteroides	s__Bacteroides_ovatus	0.0151	0.0151
g__Bacteroides	s__Bacteroides_ovatus	0.0151	0.0151
g__Bacteroides	s__Bacteroides_ovatus	0.0151	0.0151
g__Bacteroides	s__Bacteroides_ovatus	0.0151	0.0151
g__Bacteroides	s__Bacteroides_ovatus	0.0151	0.0151
g__Bacteroides	s__Bacteroides_ovatus	0.0151	0.0151
g__Bacteroides	s__Bacteroides_ovatus	0.0151	0.0151
g__Bacteroides	s__Bacteroides_ovatus	0.0151	0.0151
g__Bacteroides	s__Bacteroides_ovatus	0.0151	0.0151
g__Bacteroides	s__Bacteroides_ovatus	0.0151	0.0151
g__Bacteroides	s__Bacteroides_ovatus	0.0151	0.0151
g__Bacteroides	s__Bacteroides_ovatus	0.0151	0.0151
g__Bacteroides	s__Bacteroides_ovatus	0.0151	0.0151
g__Bacteroides	s__Bacteroides_ovatus	0.0151	0.0151
g__Bacteroides	s__Bacteroides_ovatus	0.0151	0.0151
g__Bacteroides	s__Bacteroides_ovatus	0.0151	0.0151
g__Bacteroides	s__Bacteroides_ovatus	0.0151	0.0151
g__Bacteroides	s__Bacteroides_ovatus	0.0151	0.0151
g__Bacteroides	s__Bacteroides_ovatus	0.0151	0.0151
g__Bacteroides	s__Bacteroides_ovatus	0.0151	0.0151
g__Bacteroides	s__Bacteroides_ovatus	0.0151	0.0151
g__Bacteroides	s__Bacteroides_ovatus	0.0151	0.0151
g__Bacteroides	s__Bacteroides_ovatus	0.0151	0.0151
g__Bacteroides	s__Bacteroides_salyersiae	0.0442	0.0442
g__Bacteroides	s__Bacteroides_salyersiae	0.0179	0.0179
g__Bacteroides	s__Bacteroides_salyersiae	0.0442	0.0442
g__Bacteroides	s__Bacteroides_salyersiae	0.0151	0.0151
g__Bacteroides	s__Bacteroides_salyersiae	0.0151	0.0151
g__Bacteroides	s__Bacteroides_salyersiae	0.0442	0.0442
g__Bacteroides	s__Bacteroides_salyersiae	0.0151	0.0151
g__Bacteroides	s__Bacteroides_salyersiae	0.0151	0.0151
g__Bacteroides	s__Bacteroides_salyersiae	0.0151	0.0151
g__Bacteroides	s__Bacteroides_salyersiae	0.0151	0.0151

g__Bacteroides	s__Bacteroides_salyersiae	0.0442	0.0442
g__Bacteroides	s__Bacteroides_salyersiae	0.0442	0.0442
g__Bacteroides	s__Bacteroides_salyersiae	0.0442	0.0442
g__Bacteroides	s__Bacteroides_salyersiae	0.0151	0.0151
g__Bacteroides	s__Bacteroides_salyersiae	0.0442	0.0442
g__Bacteroides	s__Bacteroides_salyersiae	0.0151	0.0151
g__Bacteroides	s__Bacteroides_salyersiae	0.0146	0.0146
g__Bacteroides	s__Bacteroides_salyersiae	0.0133	0.0133
g__Bacteroides	s__Bacteroides_stercoris	0.0151	0.0151
g__Bacteroides	s__Bacteroides_stercoris	0.0151	0.0151
g__Bacteroides	s__Bacteroides_stercoris	0.0151	0.0151
g__Bacteroides	s__Bacteroides_stercoris	0.0151	0.0151
g__Bacteroides	s__Bacteroides_stercoris	0.0442	0.0442
g__Bacteroides	s__Bacteroides_stercoris	0.0151	0.0151
g__Bacteroides	s__Bacteroides_stercoris	0.0151	0.0151
g__Bacteroides	s__Bacteroides_stercoris	0.0151	0.0151
g__Bacteroides	s__Bacteroides_stercoris	0.0151	0.0151
g__Bacteroides	s__Bacteroides_stercoris	0.0151	0.0151
g__Bacteroides	s__Bacteroides_stercoris	0.0151	0.0151
g__Bacteroides	s__Bacteroides_stercoris	0.0146	0.0146
g__Bacteroides	s__Bacteroides_stercoris	0.0151	0.0151
g__Bacteroides	s__Bacteroides_stercoris	0.0151	0.0151
g__Bacteroides	s__Bacteroides_stercoris	0.0151	0.0151
g__Bacteroides	s__Bacteroides_stercoris	0.0151	0.0151
g__Bacteroides	s__Bacteroides_stercoris	0.0151	0.0151
g__Bacteroides	s__Bacteroides_stercoris	0.0442	0.0442
g__Bacteroides	s__Bacteroides_stercoris	0.0151	0.0151
g__Bacteroides	s__Bacteroides_thetaiotaomicron	0.0151	0.0151
g__Bacteroides	s__Bacteroides_thetaiotaomicron	0.0151	0.0151
g__Bacteroides	s__Bacteroides_thetaiotaomicron	0.0151	0.0151
g__Bacteroides	s__Bacteroides_thetaiotaomicron	0.0151	0.0151
g__Bacteroides	s__Bacteroides_thetaiotaomicron	0.0179	0.0179
g__Bacteroides	s__Bacteroides_thetaiotaomicron	0.0151	0.0151
g__Bacteroides	s__Bacteroides_thetaiotaomicron	0.0151	0.0151
g__Bacteroides	s__Bacteroides_thetaiotaomicron	0.0151	0.0151
g__Bacteroides	s__Bacteroides_thetaiotaomicron	0.0151	0.0151
g__Bacteroides	s__Bacteroides_thetaiotaomicron	0.0151	0.0151
g__Bacteroides	s__Bacteroides_thetaiotaomicron	0.0151	0.0151
g__Bacteroides	s__Bacteroides_thetaiotaomicron	0.0151	0.0151
g__Bacteroides	s__Bacteroides_thetaiotaomicron	0.0442	0.0442
g__Bacteroides	s__Bacteroides_thetaiotaomicron	0.0151	0.0151
g__Bacteroides	s__Bacteroides_thetaiotaomicron	0.0151	0.0151
g__Bacteroides	s__Bacteroides_thetaiotaomicron	0.0442	0.0442

g__Bacteroides	s__Bacteroides_thetaiotaomicron	0.0151	0.0151
g__Bacteroides	s__Bacteroides_thetaiotaomicron	0.0151	0.0151
g__Bacteroides	s__Bacteroides_thetaiotaomicron	0.0151	0.0151
g__Bacteroides	s__Bacteroides_thetaiotaomicron	0.0151	0.0151
g__Bacteroides	s__Bacteroides_thetaiotaomicron	0.0151	0.0151
g__Bacteroides	s__Bacteroides_thetaiotaomicron	0.0151	0.0151
g__Bacteroides	s__Bacteroides_thetaiotaomicron	0.0151	0.0151
g__Bacteroides	s__Bacteroides_thetaiotaomicron	0.0151	0.0151
g__Bacteroides	s__Bacteroides_thetaiotaomicron	0.04	0.04
g__Bacteroides	s__Bacteroides_thetaiotaomicron	0.0151	0.0151
g__Bacteroides	s__Bacteroides_thetaiotaomicron	0.0151	0.0151
g__Bacteroides	s__Bacteroides_thetaiotaomicron	0.0151	0.0151
g__Bacteroides	s__Bacteroides_thetaiotaomicron	0.0151	0.0151
g__Bacteroides	s__Bacteroides_thetaiotaomicron	0.0442	0.0442
g__Bacteroides	s__Bacteroides_thetaiotaomicron	0.04	0.04
g__Bacteroides	s__Bacteroides_thetaiotaomicron	0.0151	0.0151
g__Bacteroides	s__Bacteroides_thetaiotaomicron	0.0151	0.0151
g__Bacteroides	s__Bacteroides_thetaiotaomicron	0.0151	0.0151
g__Bacteroides	s__Bacteroides_thetaiotaomicron	0.0151	0.0151
g__Bacteroides	s__Bacteroides_thetaiotaomicron	0.0431	0.0431
g__Bacteroides	s__Bacteroides_thetaiotaomicron	0.0442	0.0442
g__Bacteroides	s__Bacteroides_thetaiotaomicron	0.0442	0.0442
g__Bacteroides	s__Bacteroides_thetaiotaomicron	0.0151	0.0151
g__Bacteroides	s__Bacteroides_thetaiotaomicron	0.0151	0.0151
g__Bacteroides	s__Bacteroides_thetaiotaomicron	0.0151	0.0151
g__Bacteroides	s__Bacteroides_thetaiotaomicron	0.0151	0.0151
g__Bacteroides	s__Bacteroides_thetaiotaomicron	0.0151	0.0151
g__Bacteroides	s__Bacteroides_thetaiotaomicron	0.0146	0.0146
g__Bacteroides	s__Bacteroides_thetaiotaomicron	0.0151	0.0151
g__Bacteroides	s__Bacteroides_thetaiotaomicron	0.0151	0.0151
g__Bacteroides	s__Bacteroides_thetaiotaomicron	0.0151	0.0151
g__Bacteroides	s__Bacteroides_thetaiotaomicron	0.0151	0.0151
g__Bacteroides	s__Bacteroides_thetaiotaomicron	0.0151	0.0151
g__Bacteroides	s__Bacteroides_thetaiotaomicron	0.0431	0.0431
g__Bacteroides	s__Bacteroides_thetaiotaomicron	0.0151	0.0151
g__Bacteroides	s__Bacteroides_thetaiotaomicron	0.0151	0.0151
g__Bacteroides	s__Bacteroides_thetaiotaomicron	0.0151	0.0151
g__Bacteroides	s__Bacteroides_thetaiotaomicron	0.0151	0.0151
g__Bacteroides	s__Bacteroides_thetaiotaomicron	0.0151	0.0151
g__Bacteroides	s__Bacteroides_thetaiotaomicron	0.0151	0.0151
g__Bacteroides	s__Bacteroides_thetaiotaomicron	0.0151	0.0151
g__Bacteroides	s__Bacteroides_thetaiotaomicron	0.0151	0.0151
g__Bacteroides	s__Bacteroides_thetaiotaomicron	0.0442	0.0442

g__Bacteroides	s__Bacteroides_thetaiotaomicron	0.0151	0.0151
g__Bacteroides	s__Bacteroides_thetaiotaomicron	0.0151	0.0151
g__Bacteroides	s__Bacteroides_thetaiotaomicron	0.0151	0.0151
g__Bacteroides	s__Bacteroides_thetaiotaomicron	0.0442	0.0442
g__Bacteroides	s__Bacteroides_thetaiotaomicron	0.0151	0.0151
g__Bacteroides	s__Bacteroides_thetaiotaomicron	0.0151	0.0151
g__Bacteroides	s__Bacteroides_thetaiotaomicron	0.0442	0.0442
g__Bacteroides	s__Bacteroides_thetaiotaomicron	0.0151	0.0151
g__Bacteroides	s__Bacteroides_thetaiotaomicron	0.0442	0.0442
g__Bacteroides	s__Bacteroides_thetaiotaomicron	0.0151	0.0151
g__Bacteroides	s__Bacteroides_thetaiotaomicron	0.0151	0.0151
g__Bacteroides	s__Bacteroides_thetaiotaomicron	0.0151	0.0151
g__Bacteroides	s__Bacteroides_thetaiotaomicron	0.0151	0.0151
g__Bacteroides	s__Bacteroides_thetaiotaomicron	0.0151	0.0151
g__Bacteroides	s__Bacteroides_thetaiotaomicron	0.0151	0.0151
g__Bacteroides	s__Bacteroides_thetaiotaomicron	0.0151	0.0151
g__Bacteroides	s__Bacteroides_thetaiotaomicron	0.0151	0.0151
g__Bacteroides	s__Bacteroides_thetaiotaomicron	0.0151	0.0151
g__Bacteroides	s__Bacteroides_thetaiotaomicron	0.0151	0.0151
g__Bacteroides	s__Bacteroides_thetaiotaomicron	0.0151	0.0151
g__Bacteroides	s__Bacteroides_thetaiotaomicron	0.0151	0.0151
g__Bacteroides	s__Bacteroides_thetaiotaomicron	0.0442	0.0442
g__Bacteroides	s__Bacteroides_thetaiotaomicron	0.0442	0.0442
g__Bacteroides	s__Bacteroides_thetaiotaomicron	0.0442	0.0442
g__Bacteroides	s__Bacteroides_thetaiotaomicron	0.0151	0.0151
g__Bacteroides	s__Bacteroides_thetaiotaomicron	0.0442	0.0442
g__Bacteroides	s__Bacteroides_thetaiotaomicron	0.0151	0.0151
g__Bacteroides	s__Bacteroides_thetaiotaomicron	0.0151	0.0151
g__Bacteroides	s__Bacteroides_thetaiotaomicron	0.0151	0.0151
g__Bacteroides	s__Bacteroides_thetaiotaomicron	0.0151	0.0151
g__Bacteroides	s__Bacteroides_thetaiotaomicron	0.0146	0.0146
g__Bacteroides	s__Bacteroides_thetaiotaomicron	0.0151	0.0151
g__Bacteroides	s__Bacteroides_thetaiotaomicron	0.0151	0.0151
g__Bacteroides	s__Bacteroides_thetaiotaomicron	0.0151	0.0151
g__Bacteroides	s__Bacteroides_thetaiotaomicron	0.0151	0.0151
g__Bacteroides	s__Bacteroides_thetaiotaomicron	0.0151	0.0151
g__Bacteroides	s__Bacteroides_thetaiotaomicron	0.0442	0.0442
g__Bacteroides	s__Bacteroides_thetaiotaomicron	0.0151	0.0151
g__Bacteroides	s__Bacteroides_thetaiotaomicron	0.0151	0.0151
g__Bacteroides	s__Bacteroides_thetaiotaomicron	0.0151	0.0151
g__Bacteroides	s__Bacteroides_thetaiotaomicron	0.0151	0.0151
g__Bacteroides	s__Bacteroides_thetaiotaomicron	0.0151	0.0151
g__Bacteroides	s__Bacteroides_thetaiotaomicron	0.0151	0.0151

g__Bacteroides	s__Bacteroides_thetaiotaomicron	0.0151	0.0151
g__Bacteroides	s__Bacteroides_thetaiotaomicron	0.0151	0.0151
g__Bacteroides	s__Bacteroides_thetaiotaomicron	0.0151	0.0151
g__Bacteroides	s__Bacteroides_thetaiotaomicron	0.0151	0.0151
g__Bacteroides	s__Bacteroides_thetaiotaomicron	0.0442	0.0442
g__Bacteroides	s__Bacteroides_thetaiotaomicron	0.0442	0.0442
g__Bacteroides	s__Bacteroides_thetaiotaomicron	0.0151	0.0151
g__Bacteroides	s__Bacteroides_thetaiotaomicron	0.0151	0.0151
g__Bacteroides	s__Bacteroides_thetaiotaomicron	0.0151	0.0151
g__Bacteroides	s__Bacteroides_thetaiotaomicron	0.0431	0.0431
g__Bacteroides	s__Bacteroides_thetaiotaomicron	0.0151	0.0151
g__Bacteroides	s__Bacteroides_thetaiotaomicron	0.0442	0.0442
g__Bacteroides	s__Bacteroides_thetaiotaomicron	0.0151	0.0151
g__Bacteroides	s__Bacteroides_thetaiotaomicron	0.0151	0.0151
g__Bacteroides	s__Bacteroides_thetaiotaomicron	0.0151	0.0151
g__Bacteroides	s__Bacteroides_thetaiotaomicron	0.0151	0.0151
g__Bacteroides	s__Bacteroides_thetaiotaomicron	0.0151	0.0151
g__Bacteroides	s__Bacteroides_uniformis	0.0442	0.0442
g__Bacteroides	s__Bacteroides_uniformis	0.0442	0.0442
g__Bacteroides	s__Bacteroides_uniformis	0.0442	0.0442
g__Bacteroides	s__Bacteroides_uniformis	0.0151	0.0151
g__Bacteroides	s__Bacteroides_uniformis	0.0151	0.0151
g__Bacteroides	s__Bacteroides_uniformis	0.0151	0.0151
g__Bacteroides	s__Bacteroides_uniformis	0.0151	0.0151
g__Bacteroides	s__Bacteroides_uniformis	0.0151	0.0151
g__Bacteroides	s__Bacteroides_uniformis	0.0151	0.0151
g__Bacteroides	s__Bacteroides_uniformis	0.0151	0.0151
g__Bacteroides	s__Bacteroides_uniformis	0.0146	0.0146
g__Bacteroides	s__Bacteroides_uniformis	0.0442	0.0442
g__Bacteroides	s__Bacteroides_uniformis	0.0151	0.0151
g__Bacteroides	s__Bacteroides_uniformis	0.0151	0.0151
g__Bacteroides	s__Bacteroides_uniformis	0.0442	0.0442
g__Bacteroides	s__Bacteroides_uniformis	0.0151	0.0151
g__Bacteroides	s__Bacteroides_uniformis	0.0151	0.0151
g__Bacteroides	s__Bacteroides_uniformis	0.0151	0.0151
g__Bacteroides	s__Bacteroides_uniformis	0.0151	0.0151
g__Bacteroides	s__Bacteroides_uniformis	0.0151	0.0151
g__Bacteroides	s__Bacteroides_uniformis	0.0442	0.0442
g__Bacteroides	s__Bacteroides_uniformis	0.0442	0.0442
g__Bacteroides	s__Bacteroides_uniformis	0.0151	0.0151
g__Bacteroides	s__Bacteroides_uniformis	0.0442	0.0442
g__Bacteroides	s__Bacteroides_uniformis	0.0442	0.0442
g__Bacteroides	s__Bacteroides_uniformis	0.0151	0.0151

g__Bacteroides	s__Bacteroides_uniformis	0.0442	0.0442
g__Bacteroides	s__Bacteroides_uniformis	0.0431	0.0431
g__Bacteroides	s__Bacteroides_uniformis	0.0151	0.0151
g__Bacteroides	s__Bacteroides_uniformis	0.0442	0.0442
g__Bacteroides	s__Bacteroides_uniformis	0.0151	0.0151
g__Bacteroides	s__Bacteroides_uniformis	0.0151	0.0151
g__Bacteroides	s__Bacteroides_uniformis	0.0442	0.0442
g__Bacteroides	s__Bacteroides_uniformis	0.0151	0.0151
g__Bacteroides	s__Bacteroides_uniformis	0.0151	0.0151
g__Bacteroides	s__Bacteroides_uniformis	0.0133	0.0133
g__Bacteroides	s__Bacteroides_uniformis	0.0151	0.0151
g__Bacteroides	s__Bacteroides_uniformis	0.0151	0.0151
g__Bacteroides	s__Bacteroides_uniformis	0.0151	0.0151
g__Bacteroides	s__Bacteroides_uniformis	0.0151	0.0151
g__Bacteroides	s__Bacteroides_uniformis	0.0151	0.0151
g__Bacteroides	s__Bacteroides_uniformis	0.0151	0.0151
g__Bacteroides	s__Bacteroides_uniformis	0.0151	0.0151
g__Bacteroides	s__Bacteroides_uniformis	0.0431	0.0431
g__Bacteroides	s__Bacteroides_uniformis	0.0151	0.0151
g__Bacteroides	s__Bacteroides_uniformis	0.0442	0.0442
g__Bacteroides	s__Bacteroides_uniformis	0.04	0.04
g__Bacteroides	s__Bacteroides_uniformis	0.0442	0.0442
g__Bacteroides	s__Bacteroides_uniformis	0.0151	0.0151
g__Bacteroides	s__Bacteroides_uniformis	0.0442	0.0442
g__Bacteroides	s__Bacteroides_uniformis	0.0151	0.0151
g__Bacteroides	s__Bacteroides_uniformis	0.0151	0.0151
g__Bacteroides	s__Bacteroides_uniformis	0.0151	0.0151
g__Bacteroides	s__Bacteroides_uniformis	0.0442	0.0442
g__Bacteroides	s__Bacteroides_uniformis	0.0442	0.0442
g__Bacteroides	s__Bacteroides_uniformis	0.0142	0.0142
g__Bacteroides	s__Bacteroides_uniformis	0.0151	0.0151
g__Bacteroides	s__Bacteroides_uniformis	0.0151	0.0151
g__Bacteroides	s__Bacteroides_uniformis	0.0442	0.0442
g__Bacteroides	s__Bacteroides_uniformis	0.0442	0.0442
g__Bacteroides	s__Bacteroides_uniformis	0.0151	0.0151
g__Bacteroides	s__Bacteroides_uniformis	0.0151	0.0151
g__Bacteroides	s__Bacteroides_uniformis	0.0442	0.0442
g__Bacteroides	s__Bacteroides_uniformis	0.0442	0.0442
g__Bacteroides	s__Bacteroides_uniformis	0.0151	0.0151
g__Bacteroides	s__Bacteroides_uniformis	0.0151	0.0151
g__Bacteroides	s__Bacteroides_uniformis	0.0442	0.0442
g__Bacteroides	s__Bacteroides_uniformis	0.0442	0.0442
g__Bacteroides	s__Bacteroides_uniformis	0.0151	0.0151

g__Bacteroides	s__Bacteroides_uniformis	0.0442	0.0442
g__Bacteroides	s__Bacteroides_uniformis	0.0151	0.0151
g__Bacteroides	s__Bacteroides_uniformis	0.0151	0.0151
g__Bacteroides	s__Bacteroides_uniformis	0.0151	0.0151
g__Bacteroides	s__Bacteroides_uniformis	0.0146	0.0146
g__Bacteroides	s__Bacteroides_uniformis	0.0442	0.0442
g__Bacteroides	s__Bacteroides_uniformis	0.0151	0.0151
g__Bacteroides	s__Bacteroides_uniformis	0.0442	0.0442
g__Bacteroides	s__Bacteroides_uniformis	0.0442	0.0442
g__Bacteroides	s__Bacteroides_uniformis	0.0151	0.0151
g__Bacteroides	s__Bacteroides_uniformis	0.0151	0.0151
g__Bacteroides	s__Bacteroides_uniformis	0.0146	0.0146
g__Bacteroides	s__Bacteroides_uniformis	0.0151	0.0151
g__Bacteroides	s__Bacteroides_uniformis	0.0151	0.0151
g__Bacteroides	s__Bacteroides_uniformis	0.0151	0.0151
g__Bacteroides	s__Bacteroides_xylanisolvens	0.0151	0.0151
g__Bacteroides	s__Bacteroides_xylanisolvens	0.0151	0.0151
g__Bacteroides	s__Bacteroides_xylanisolvens	0.0151	0.0151
g__Bacteroides	s__Bacteroides_xylanisolvens	0.0151	0.0151
g__Bacteroides	s__Bacteroides_xylanisolvens	0.0151	0.0151
g__Bacteroides	s__Bacteroides_xylanisolvens	0.0151	0.0151
g__Bacteroides	s__Bacteroides_xylanisolvens	0.0151	0.0151
g__Bacteroides	s__Bacteroides_xylanisolvens	0.0146	0.0146
g__Bacteroides	s__Bacteroides_xylanisolvens	0.0151	0.0151
g__Bacteroides	s__Bacteroides_xylanisolvens	0.0431	0.0431
g__Bacteroides	s__Bacteroides_xylanisolvens	0.0151	0.0151
g__Bacteroides	s__Bacteroides_xylanisolvens	0.0151	0.0151
g__Bacteroides	s__Bacteroides_xylanisolvens	0.0151	0.0151
g__Bacteroides	s__Bacteroides_xylanisolvens	0.0151	0.0151
g__Bacteroides	s__Bacteroides_xylanisolvens	0.0151	0.0151
g__Bacteroides	s__Bacteroides_xylanisolvens	0.0151	0.0151
g__Bacteroides	s__Bacteroides_xylanisolvens	0.0151	0.0151
g__Bacteroides	s__Bacteroides_xylanisolvens	0.0151	0.0151
g__Bacteroides	s__Bacteroides_xylanisolvens	0.0151	0.0151
g__Bacteroides	s__Bacteroides_xylanisolvens	0.0151	0.0151
g__Bacteroides	s__Bacteroides_xylanisolvens	0.0151	0.0151
g__Bacteroides	s__Bacteroides_xylanisolvens	0.0179	0.0179
g__Bacteroides	s__Bacteroides_xylanisolvens	0.0151	0.0151
g__Bacteroides	s__Bacteroides_xylanisolvens	0.0151	0.0151
g__Bacteroides	s__Bacteroides_xylanisolvens	0.0151	0.0151
g__Bacteroides	s__Bacteroides_xylanisolvens	0.0151	0.0151
g__Bacteroides	s__Bacteroides_xylanisolvens	0.0151	0.0151
g__Bacteroides	s__Bacteroides_xylanisolvens	0.0151	0.0151

g__Bacteroides	s__Bacteroides_xylanisolvens	0.0151	0.0151
g__Bacteroides	s__Bacteroides_xylanisolvens	0.0151	0.0151
g__Bacteroides	s__Bacteroides_xylanisolvens	0.0151	0.0151
g__Bacteroides	s__Bacteroides_xylanisolvens	0.0151	0.0151
g__Bacteroides	s__Bacteroides_xylanisolvens	0.0151	0.0151
g__Bacteroides	s__Bacteroides_xylanisolvens	0.0151	0.0151
g__Bacteroides	s__Bacteroides_xylanisolvens	0.0151	0.0151
g__Bacteroides	s__Bacteroides_xylanisolvens	0.0151	0.0151
g__Bacteroides	s__Bacteroides_xylanisolvens	0.0151	0.0151
g__Bacteroides	s__Bacteroides_xylanisolvens	0.0151	0.0151
g__Bacteroides	s__Bacteroides_xylanisolvens	0.0151	0.0151
g__Bacteroides	s__Bacteroides_xylanisolvens	0.0151	0.0151
g__Bacteroides	s__Bacteroides_xylanisolvens	0.0151	0.0151
g__Bacteroides	s__Bacteroides_xylanisolvens	0.0151	0.0151
g__Bacteroides	s__Bacteroides_xylanisolvens	0.0151	0.0151
g__Bacteroides	s__Bacteroides_xylanisolvens	0.0151	0.0151
g__Bacteroides	s__Bacteroides_xylanisolvens	0.0151	0.0151
g__Bacteroides	s__Bacteroides_xylanisolvens	0.0151	0.0151
g__Bacteroides	s__Bacteroides_xylanisolvens	0.0151	0.0151
g__Bacteroides	s__Bacteroides_xylanisolvens	0.0151	0.0151
g__Bacteroides	s__Bacteroides_xylanisolvens	0.0151	0.0151
g__Bacteroides	s__Bacteroides_xylanisolvens	0.0151	0.0151
g__Bacteroides	s__Bacteroides_xylanisolvens	0.0151	0.0151
g__Bacteroides	s__Bacteroides_xylanisolvens	0.0151	0.0151
g__Bacteroides	s__Bacteroides_xylanisolvens	0.0151	0.0151
g__Bacteroides	s__Bacteroides_xylanisolvens	0.0151	0.0151
g__Bacteroides	s__Bacteroides_xylanisolvens	0.0151	0.0151
g__Bacteroides	s__Bacteroides_xylanisolvens	0.0151	0.0151
g__Bacteroides	s__Bacteroides_xylanisolvens	0.0151	0.0151
g__Bacteroides	s__Bacteroides_xylanisolvens	0.0151	0.0151
g__Bacteroides	s__Bacteroides_xylanisolvens	0.0151	0.0151
g__Bacteroides	s__Bacteroides_xylanisolvens	0.0151	0.0151
g__Bacteroides	s__Bacteroides_xylanisolvens	0.0431	0.0431
g__Bacteroides	s__Bacteroides_xylanisolvens	0.0151	0.0151
g__Bacteroides	s__Bacteroides_xylanisolvens	0.0151	0.0151
g__Bacteroides	s__Bacteroides_xylanisolvens	0.0151	0.0151
g__Bacteroides	s__Bacteroides_xylanisolvens	0.0151	0.0151
g__Bacteroides	s__Bacteroides_xylanisolvens	0.0442	0.0442
g__Bacteroides	s__Bacteroides_xylanisolvens	0.0133	0.0133
g__Bacteroides	s__Bacteroides_xylanisolvens	0.0151	0.0151
g__Bacteroides	s__Bacteroides_xylanisolvens	0.0151	0.0151
g__Bacteroides	s__Bacteroides_xylanisolvens	0.0151	0.0151
g__Bacteroides	s__Bacteroides_xylanisolvens	0.0151	0.0151

g__Bacteroides	s__Bacteroides_xylanisolvens	0.0151	0.0151
g__Bacteroides	s__Bacteroides_xylanisolvens	0.0151	0.0151
g__Bacteroides	s__Bacteroides_xylanisolvens	0.0431	0.0431
g__Bacteroides	s__Bacteroides_xylanisolvens	0.0442	0.0442
g__Bacteroides	s__Bacteroides_xylanisolvens	0.0151	0.0151
g__Bacteroides	s__Bacteroides_xylanisolvens	0.0151	0.0151
g__Bacteroides	s__Bacteroides_xylanisolvens	0.0151	0.0151
g__Bacteroides	s__Bacteroides_xylanisolvens	0.0151	0.0151
g__Bacteroides	s__Bacteroides_xylanisolvens	0.0151	0.0151
g__Bacteroides	s__Bacteroides_xylanisolvens	0.0151	0.0151
g__Bacteroides	s__Bacteroides_xylanisolvens	0.0151	0.0151
g__Bacteroides	s__Bacteroides_xylanisolvens	0.0151	0.0151
g__Bacteroides	s__Bacteroides_xylanisolvens	0.0151	0.0151
g__Bacteroides	s__Bacteroides_xylanisolvens	0.0151	0.0151
g__Bacteroides	s__Bacteroides_xylanisolvens	0.0151	0.0151
g__Bacteroides	s__Bacteroides_xylanisolvens	0.0151	0.0151
g__Bacteroides	s__Bacteroides_xylanisolvens	0.0442	0.0442
g__Bacteroides	s__Bacteroides_xylanisolvens	0.0151	0.0151
g__Bacteroides	s__Bacteroides_xylanisolvens	0.0151	0.0151
g__Bacteroides	s__Bacteroides_xylanisolvens	0.0151	0.0151
g__Bacteroides	s__Bacteroides_xylanisolvens	0.0151	0.0151
g__Bacteroides	s__Bacteroides_xylanisolvens	0.0151	0.0151
g__Bacteroides	s__Bacteroides_xylanisolvens	0.0151	0.0151
g__Bacteroides	s__Bacteroides_xylanisolvens	0.0151	0.0151
g__Bacteroides	s__Bacteroides_xylanisolvens	0.0151	0.0151
g__Bacteroides	s__Bacteroides_xylanisolvens	0.0151	0.0151
g__Bacteroides	s__Bacteroides_xylanisolvens	0.0151	0.0151
g__Bacteroides	s__Bacteroides_xylanisolvens	0.0151	0.0151
g__Bacteroides	s__Bacteroides_xylanisolvens	0.0151	0.0151
g__Bacteroides	s__Bacteroides_xylanisolvens	0.0151	0.0151
g__Bacteroides	s__Bacteroides_xylanisolvens	0.0151	0.0151
g__Bacteroides	s__Bacteroides_xylanisolvens	0.0151	0.0151
g__Bacteroides	s__Bacteroides_xylanisolvens	0.0151	0.0151
g__Bacteroides	s__Bacteroides_xylanisolvens	0.0151	0.0151
g__Bacteroides	s__Bacteroides_xylanisolvens	0.0151	0.0151
g__Bacteroides	s__Bacteroides_xylanisolvens	0.0151	0.0151
g__Bacteroides	s__Bacteroides_xylanisolvens	0.0151	0.0151
g__Bacteroides	s__Bacteroides_xylanisolvens	0.0151	0.0151
g__Bacteroides	s__Bacteroides_xylanisolvens	0.0442	0.0442
g__Bacteroides	s__Bacteroides_xylanisolvens	0.0151	0.0151
g__Bacteroides	s__Bacteroides_xylanisolvens	0.0151	0.0151
g__Bacteroides	s__Bacteroides_xylanisolvens	0.0151	0.0151
g__Bacteroides	s__Bacteroides_xylanisolvens	0.0151	0.0151

g__Bacteroides	s__Bacteroides_xylanisolvens	0.0151	0.0151
g__Bacteroides	s__Bacteroides_xylanisolvens	0.0151	0.0151
g__Bacteroides	s__Bacteroides_xylanisolvens	0.0151	0.0151
g__Bacteroides	s__Bacteroides_xylanisolvens	0.0151	0.0151
g__Bacteroides	s__Bacteroides_xylanisolvens	0.0151	0.0151
g__Bacteroides	s__Bacteroides_xylanisolvens	0.0151	0.0151
g__Bacteroides	s__Bacteroides_xylanisolvens	0.0442	0.0442
g__Bacteroides	s__Bacteroides_xylanisolvens	0.0151	0.0151
g__Bacteroides	s__Bacteroides_xylanisolvens	0.0151	0.0151
g__Bacteroides	s__Bacteroides_xylanisolvens	0.04	0.04
g__Bacteroides	s__Bacteroides_xylanisolvens	0.0151	0.0151
g__Bacteroides	s__Bacteroides_xylanisolvens	0.0151	0.0151
g__Bacteroides	s__Bacteroides_xylanisolvens	0.0442	0.0442
g__Bacteroides	s__Bacteroides_xylanisolvens	0.0151	0.0151
g__Bacteroides	s__Bacteroides_xylanisolvens	0.0151	0.0151
g__Bacteroides	s__Bacteroides_xylanisolvens	0.0442	0.0442
g__Bacteroides	s__Bacteroides_xylanisolvens	0.0151	0.0151
g__Bacteroides	s__Bacteroides_xylanisolvens	0.0151	0.0151
g__Bacteroides	s__Bacteroides_xylanisolvens	0.0151	0.0151
g__Bacteroides	s__Bacteroides_xylanisolvens	0.0151	0.0151
g__Bacteroides	s__Bacteroides_xylanisolvens	0.0151	0.0151
g__Bacteroides	s__Bacteroides_xylanisolvens	0.0151	0.0151
g__Bacteroides	s__Bacteroides_xylanisolvens	0.0151	0.0151
g__Bacteroides	s__Bacteroides_xylanisolvens	0.0431	0.0431
g__Bacteroides	s__Bacteroides_xylanisolvens	0.0151	0.0151
g__Bacteroides	s__Bacteroides_xylanisolvens	0.0151	0.0151
g__Bacteroides	s__Bacteroides_xylanisolvens	0.0151	0.0151
g__Bacteroides	s__Bacteroides_xylanisolvens	0.0151	0.0151
g__Bacteroides	s__Bacteroides_xylanisolvens	0.0151	0.0151
g__Bacteroides	s__Bacteroides_xylanisolvens	0.0151	0.0151
g__Bacteroides	s__Bacteroides_xylanisolvens	0.0442	0.0442
g__Bacteroides	s__Bacteroides_xylanisolvens	0.0151	0.0151
g__Bacteroides	s__Bacteroides_xylanisolvens	0.0151	0.0151
g__Bacteroides	s__Bacteroides_xylanisolvens	0.0151	0.0151
g__Bacteroides	s__Bacteroides_xylanisolvens	0.0151	0.0151
g__Bacteroides	s__Bacteroides_xylanisolvens	0.0442	0.0442
g__Bacteroides	s__Bacteroides_xylanisolvens	0.0151	0.0151
g__Bacteroides	s__Bacteroides_xylanisolvens	0.0442	0.0442
g__Bacteroides	s__Bacteroides_xylanisolvens	0.0442	0.0442
g__Bacteroides	s__Bacteroides_xylanisolvens	0.0151	0.0151
g__Bacteroides	s__Bacteroides_xylanisolvens	0.0151	0.0151
g__Bacteroides	s__Bacteroides_xylanisolvens	0.0151	0.0151
g__Bacteroides	s__Bacteroides_xylanisolvens	0.0151	0.0151

g__Bacteroides	s__Bacteroides_xylanisolvens	0.0151	0.0151
g__Bacteroides	s__Bacteroides_xylanisolvens	0.0151	0.0151
g__Bacteroides	s__Bacteroides_xylanisolvens	0.0151	0.0151
g__Bacteroides	s__Bacteroides_xylanisolvens	0.0151	0.0151
g__Bacteroides	s__Bacteroides_xylanisolvens	0.0151	0.0151
g__Bacteroides	s__Bacteroides_xylanisolvens	0.0151	0.0151
g__Bacteroides	s__Bacteroides_xylanisolvens	0.0431	0.0431
g__Bacteroides	s__Bacteroides_xylanisolvens	0.0151	0.0151
g__Bacteroides	s__Bacteroides_xylanisolvens	0.0442	0.0442
g__Bacteroides	s__Bacteroides_xylanisolvens	0.0151	0.0151
g__Bacteroides	s__Bacteroides_xylanisolvens	0.0151	0.0151
g__Bacteroides	s__Bacteroides_xylanisolvens	0.0442	0.0442
g__Bacteroides	s__Bacteroides_xylanisolvens	0.0151	0.0151
g__Bacteroides	s__Bacteroides_xylanisolvens	0.0146	0.0146
g__Bacteroides	s__Bacteroides_xylanisolvens	0.0151	0.0151
g__Bacteroides	s__Bacteroides_xylanisolvens	0.0151	0.0151
g__Bacteroides	s__Bacteroides_xylanisolvens	0.0151	0.0151
g__Bacteroides	s__Bacteroides_xylanisolvens	0.0151	0.0151
g__Bacteroides	s__Bacteroides_xylanisolvens	0.0442	0.0442
g__Bacteroides	s__Bacteroides_xylanisolvens	0.0442	0.0442
g__Bacteroides	s__Bacteroides_xylanisolvens	0.0151	0.0151
g__Bacteroides	s__Bacteroides_xylanisolvens	0.0151	0.0151
g__Bacteroides	s__Bacteroides_xylanisolvens	0.0151	0.0151
g__Bacteroides	s__Bacteroides_xylanisolvens	0.0151	0.0151
g__Bacteroides	s__Bacteroides_xylanisolvens	0.0151	0.0151
g__Bacteroides	s__Bacteroides_xylanisolvens	0.0151	0.0151
g__Bacteroides	s__Bacteroides_xylanisolvens	0.0442	0.0442
g__Bacteroides	s__Bacteroides_xylanisolvens	0.0442	0.0442
g__Bacteroides	s__Bacteroides_xylanisolvens	0.0442	0.0442
g__Bacteroides	s__Bacteroides_xylanisolvens	0.0151	0.0151
g__Bacteroides	s__Bacteroides_xylanisolvens	0.0151	0.0151
g__Bacteroides	s__Bacteroides_xylanisolvens	0.0151	0.0151
g__Bacteroides	s__Bacteroides_xylanisolvens	0.0151	0.0151
g__Bacteroides	s__Bacteroides_xylanisolvens	0.0151	0.0151
g__Bacteroides	s__Bacteroides_xylanisolvens	0.0151	0.0151
g__Bacteroides	s__Bacteroides_xylanisolvens	0.0151	0.0151
g__Bacteroides	s__Bacteroides_xylanisolvens	0.0151	0.0151
g__Bacteroides	s__Bacteroides_xylanisolvens	0.0151	0.0151
g__Bacteroides	s__Bacteroides_xylanisolvens	0.0151	0.0151
g__Bacteroides	s__Bacteroides_xylanisolvens	0.0151	0.0151
g__Bacteroides	s__Bacteroides_xylanisolvens	0.0151	0.0151
g__Bacteroides	s__Bacteroides_xylanisolvens	0.0151	0.0151
g__Bacteroides	s__Bacteroides_xylanisolvens	0.0151	0.0151

g__Bacteroides	s__Bacteroides_xylanisolvens	0.0151	0.0151
g__Bacteroides	s__Bacteroides_xylanisolvens	0.0151	0.0151
g__Bacteroides	s__Bacteroides_xylanisolvens	0.0151	0.0151
g__Bacteroides	s__Bacteroides_xylanisolvens	0.0151	0.0151
g__Bacteroides	s__Bacteroides_xylanisolvens	0.0151	0.0151
g__Bacteroides	s__Bacteroides_xylanisolvens	0.0151	0.0151
g__Bacteroides	s__Bacteroides_xylanisolvens	0.0151	0.0151
g__Bacteroides	s__Bacteroides_xylanisolvens	0.0151	0.0151
g__Bacteroides	s__Bacteroides_xylanisolvens	0.0151	0.0151
g__Bacteroides	s__Bacteroides_xylanisolvens	0.0151	0.0151
g__Bacteroides	s__Bacteroides_xylanisolvens	0.0151	0.0151
g__Bacteroides	s__Bacteroides_xylanisolvens	0.0442	0.0442
g__Bacteroides	s__Bacteroides_xylanisolvens	0.0151	0.0151
g__Bacteroides	s__Bacteroides_xylanisolvens	0.0151	0.0151
g__Bacteroides	s__Bacteroides_xylanisolvens	0.0151	0.0151
g__Bacteroides	s__Bacteroides_xylanisolvens	0.0151	0.0151
g__Bacteroides	s__Bacteroides_xylanisolvens	0.0151	0.0151
g__Bacteroides	s__Bacteroides_xylanisolvens	0.0151	0.0151
g__Bacteroides	s__Bacteroides_xylanisolvens	0.0151	0.0151
g__Bacteroides	s__Bacteroides_xylanisolvens	0.0151	0.0151
g__Bacteroides	s__Bacteroides_xylanisolvens	0.0442	0.0442
g__Bacteroides	s__Bacteroides_xylanisolvens	0.0151	0.0151
g__Bacteroides	s__Bacteroides_xylanisolvens	0.0151	0.0151
g__Bacteroides	s__Bacteroides_xylanisolvens	0.0151	0.0151
g__Bacteroides	s__Bacteroides_xylanisolvens	0.0151	0.0151
g__Bacteroides	s__Bacteroides_xylanisolvens	0.0151	0.0151
g__Bacteroides	s__Bacteroides_xylanisolvens	0.0151	0.0151
g__Bacteroides	s__Bacteroides_xylanisolvens	0.0151	0.0151
g__Bacteroides	s__Bacteroides_xylanisolvens	0.0151	0.0151
g__Bacteroides	s__Bacteroides_xylanisolvens	0.0151	0.0151
g__Bacteroides	s__Bacteroides_xylanisolvens	0.0151	0.0151
g__Bacteroides	s__Bacteroides_xylanisolvens	0.0151	0.0151
g__Bacteroides	s__Bacteroides_xylanisolvens	0.0151	0.0151
g__Bacteroides	s__Bacteroides_xylanisolvens	0.0151	0.0151
g__Bacteroides	s__Bacteroides_xylanisolvens	0.0151	0.0151
g__Bacteroides	s__Bacteroides_xylanisolvens	0.0151	0.0151
g__Bacteroides	s__Bacteroides_xylanisolvens	0.0151	0.0151
g__Bacteroides	s__Bacteroides_xylanisolvens	0.0151	0.0151
g__Bacteroides	s__Bacteroides_xylanisolvens	0.0151	0.0151
g__Bacteroides	s__Bacteroides_xylanisolvens	0.0151	0.0151
g__Bacteroides	s__Bacteroides_xylanisolvens	0.0151	0.0151
g__Bacteroides	s__Bacteroides_xylanisolvens	0.0151	0.0151
g__Bacteroides	s__Bacteroides_xylanisolvens	0.0151	0.0151

g__Bacteroides	s__Bacteroides_xylanisolvens	0.0151	0.0151
g__Bacteroides	s__Bacteroides_xylanisolvens	0.0151	0.0151
g__Bacteroides	s__Bacteroides_xylanisolvens	0.0151	0.0151
g__Bacteroides	s__Bacteroides_xylanisolvens	0.0151	0.0151
g__Bacteroides	s__Bacteroides_xylanisolvens	0.0151	0.0151
g__Bacteroides	s__Bacteroides_xylanisolvens	0.0151	0.0151
g__Bacteroides	s__Bacteroides_xylanisolvens	0.0151	0.0151
g__Bacteroides	s__Bacteroides_xylanisolvens	0.0151	0.0151
g__Bacteroides	s__Bacteroides_xylanisolvens	0.0151	0.0151
g__Bacteroides	s__Bacteroides_xylanisolvens	0.0151	0.0151
g__Bacteroides	s__Bacteroides_xylanisolvens	0.0151	0.0151
g__Bacteroides	s__Bacteroides_xylanisolvens	0.0442	0.0442
g__Bacteroides	s__Bacteroides_xylanisolvens	0.0151	0.0151
g__Bacteroides	s__Bacteroides_xylanisolvens	0.0151	0.0151
g__Bacteroides	s__Bacteroides_xylanisolvens	0.0151	0.0151
g__Bacteroides	s__Bacteroides_xylanisolvens	0.0151	0.0151
g__Bacteroides	s__Bacteroides_xylanisolvens	0.0151	0.0151
g__Bacteroides	s__Bacteroides_xylanisolvens	0.0151	0.0151
g__Bacteroides	s__Bacteroides_xylanisolvens	0.0151	0.0151
g__Bacteroides	s__Bacteroides_xylanisolvens	0.0133	0.0133
g__Bacteroides	s__Bacteroides_xylanisolvens	0.0151	0.0151
g__Bacteroides	s__Bacteroides_xylanisolvens	0.0151	0.0151
g__Bacteroides	s__Bacteroides_xylanisolvens	0.0151	0.0151
g__Bacteroides	s__Bacteroides_xylanisolvens	0.0151	0.0151
g__Bacteroides	s__Bacteroides_xylanisolvens	0.0151	0.0151
g__Bacteroides	s__Bacteroides_xylanisolvens	0.0151	0.0151
g__Bacteroides	s__Bacteroides_xylanisolvens	0.0151	0.0151
g__Bacteroides	s__Bacteroides_xylanisolvens	0.0146	0.0146
g__Bacteroides	s__Bacteroides_xylanisolvens	0.0151	0.0151
g__Bacteroides	s__Bacteroides_xylanisolvens	0.0151	0.0151
g__Bacteroides	s__Bacteroides_xylanisolvens	0.0442	0.0442
g__Bacteroides	s__Bacteroides_xylanisolvens	0.0151	0.0151
g__Bacteroides	s__Bacteroides_xylanisolvens	0.0151	0.0151
g__Bacteroides	s__Bacteroides_xylanisolvens	0.0151	0.0151
g__Bacteroides	s__Bacteroides_xylanisolvens	0.0151	0.0151
g__Bacteroides	s__Bacteroides_xylanisolvens	0.0151	0.0151
g__Bacteroides	s__Bacteroides_xylanisolvens	0.0151	0.0151
g__Bacteroides	s__Bacteroides_xylanisolvens	0.0151	0.0151
g__Bacteroides	s__Bacteroides_xylanisolvens	0.0151	0.0151
g__Bacteroides	s__Bacteroides_xylanisolvens	0.0151	0.0151
g__Bacteroides	s__Bacteroides_xylanisolvens	0.0151	0.0151
g__Bacteroides	s__Bacteroides_xylanisolvens	0.0151	0.0151
g__Bacteroides	s__Bacteroides_xylanisolvens	0.0146	0.0146

g__Bacteroides	s__Bacteroides_xylanisolvens	0.0151	0.0151
g__Bacteroides	s__Bacteroides_xylanisolvens	0.0442	0.0442
g__Bacteroides	s__Bacteroides_xylanisolvens	0.0151	0.0151
g__Bacteroides	s__Bacteroides_xylanisolvens	0.0151	0.0151
g__Bacteroides	s__Bacteroides_xylanisolvens	0.0151	0.0151
g__Bacteroides	s__Bacteroides_xylanisolvens	0.0151	0.0151
g__Bacteroides	s__Bacteroides_xylanisolvens	0.0151	0.0151
g__Bacteroides	s__Bacteroides_xylanisolvens	0.0151	0.0151
g__Bacteroides	s__Bacteroides_xylanisolvens	0.0151	0.0151
g__Bacteroides	s__Bacteroides_xylanisolvens	0.0151	0.0151
g__Bacteroides	s__Bacteroides_xylanisolvens	0.0151	0.0151
g__Bacteroides	s__Bacteroides_xylanisolvens	0.0151	0.0151
g__Bacteroides	s__Bacteroides_xylanisolvens	0.0151	0.0151
g__Bacteroides	s__Bacteroides_xylanisolvens	0.0151	0.0151
g__Bacteroides	s__Bacteroides_xylanisolvens	0.0442	0.0442
g__Bacteroides	s__Bacteroides_xylanisolvens	0.0151	0.0151
g__Bacteroides	s__Bacteroides_xylanisolvens	0.0146	0.0146
g__Bacteroides	s__Bacteroides_xylanisolvens	0.0151	0.0151
g__Bacteroides	s__Bacteroides_xylanisolvens	0.0442	0.0442
g__Bacteroides	s__Bacteroides_xylanisolvens	0.0151	0.0151
g__Bacteroides	s__Bacteroides_xylanisolvens	0.0151	0.0151
g__Bacteroides	s__Bacteroides_xylanisolvens	0.0151	0.0151
g__Bacteroides	s__Bacteroides_xylanisolvens	0.0151	0.0151
g__Bacteroides	s__Bacteroides_xylanisolvens	0.0151	0.0151
g__Bacteroides	s__Bacteroides_xylanisolvens	0.0151	0.0151
g__Bacteroides	s__Bacteroides_xylanisolvens	0.0151	0.0151
g__Bacteroides	s__Bacteroides_xylanisolvens	0.0151	0.0151
g__Bacteroides	s__Bacteroides_xylanisolvens	0.0151	0.0151
g__Bacteroides	s__Bacteroides_xylanisolvens	0.0151	0.0151
g__Bacteroides	s__Bacteroides_xylanisolvens	0.0151	0.0151
g__Bacteroides	s__Bacteroides_xylanisolvens	0.0151	0.0151
g__Bacteroides	s__Bacteroides_xylanisolvens	0.0151	0.0151
g__Bacteroides	s__Bacteroides_xylanisolvens	0.0151	0.0151
g__Bacteroides	s__Bacteroides_xylanisolvens	0.0151	0.0151
g__Bacteroides	s__Bacteroides_xylanisolvens	0.0151	0.0151
g__Bacteroides	s__Bacteroides_xylanisolvens	0.0151	0.0151
g__Bacteroides	s__Bacteroides_xylanisolvens	0.0151	0.0151
g__Bacteroides	s__Bacteroides_xylanisolvens	0.0151	0.0151
g__Bacteroides	s__Bacteroides_xylanisolvens	0.0151	0.0151
g__Bacteroides	s__Bacteroides_xylanisolvens	0.0151	0.0151
g__Bacteroides	s__Bacteroides_xylanisolvens	0.0151	0.0151
g__Bacteroides	s__Bacteroides_xylanisolvens	0.0151	0.0151
g__Bacteroides	s__Bacteroides_xylanisolvens	0.0151	0.0151

g__Bacteroides	s__Bacteroides_xylanisolvens	0.0151	0.0151
g__Bacteroides	s__Bacteroides_xylanisolvens	0.0151	0.0151
g__Bacteroides	s__Bacteroides_xylanisolvens	0.0151	0.0151
g__Bacteroides	s__Bacteroides_xylanisolvens	0.0151	0.0151
g__Bacteroides	s__Bacteroides_xylanisolvens	0.0151	0.0151
g__Bacteroides	s__Bacteroides_xylanisolvens	0.0151	0.0151
g__Bacteroides	s__Bacteroides_xylanisolvens	0.0151	0.0151
g__Bacteroides	s__Bacteroides_xylanisolvens	0.0151	0.0151
g__Bacteroides	s__Bacteroides_xylanisolvens	0.0151	0.0151
g__Bacteroides	s__Bacteroides_xylanisolvens	0.0151	0.0151
g__Bacteroides	s__Bacteroides_xylanisolvens	0.0151	0.0151
g__Bacteroides	s__Bacteroides_xylanisolvens	0.0151	0.0151
g__Bacteroides	s__Bacteroides_xylanisolvens	0.0151	0.0151
g__Bacteroides	s__Bacteroides_xylanisolvens	0.0151	0.0151
g__Bacteroides	s__Bacteroides_xylanisolvens	0.0151	0.0151
g__Bacteroides	s__Bacteroides_xylanisolvens	0.0151	0.0151
g__Bacteroides	s__Bacteroides_xylanisolvens	0.0151	0.0151
g__Bacteroides	s__Bacteroides_xylanisolvens	0.0151	0.0151
g__Bacteroides	s__Bacteroides_xylanisolvens	0.0442	0.0442
g__Bacteroides	s__Bacteroides_xylanisolvens	0.0151	0.0151
g__Bacteroides	s__Bacteroides_xylanisolvens	0.0151	0.0151
g__Bacteroides	s__Bacteroides_xylanisolvens	0.0133	0.0133
g__Bacteroides	s__Bacteroides_xylanisolvens	0.0151	0.0151
g__Bacteroides	s__Bacteroides_xylanisolvens	0.0151	0.0151
g__Bacteroides	s__Bacteroides_xylanisolvens	0.0151	0.0151
g__Bacteroides	s__Bacteroides_xylanisolvens	0.0151	0.0151
g__Bacteroides	s__Bacteroides_xylanisolvens	0.0151	0.0151
g__Bacteroides	s__Bacteroides_xylanisolvens	0.0151	0.0151
g__Bacteroides	s__Bacteroides_xylanisolvens	0.0151	0.0151
g__Bacteroides	s__Bacteroides_xylanisolvens	0.0151	0.0151
g__Bacteroides	s__Bacteroides_xylanisolvens	0.0151	0.0151
g__Bacteroides	s__Bacteroides_xylanisolvens	0.0151	0.0151
g__Bacteroides	s__Bacteroides_xylanisolvens	0.0129	0.0129
g__Bacteroides	s__Bacteroides_xylanisolvens	0.0151	0.0151
g__Bacteroides	s__Bacteroides_xylanisolvens	0.0151	0.0151
g__Bacteroides	s__Bacteroides_xylanisolvens	0.0151	0.0151
g__Bacteroides	s__Bacteroides_xylanisolvens	0.0151	0.0151
g__Bacteroides	s__Bacteroides_xylanisolvens	0.0151	0.0151
g__Bacteroides	s__Bacteroides_xylanisolvens	0.0151	0.0151
g__Bacteroides	s__Bacteroides_xylanisolvens	0.0151	0.0151
g__Bacteroides	s__Bacteroides_xylanisolvens	0.0151	0.0151
g__Bacteroides	s__Bacteroides_zoogleoformans	0.0151	0.0151
g__Bacteroides	s__Bacteroides_zoogleoformans	0.0151	0.0151

g__Bacteroides	s__Bacteroides_zoogleoformans	0.0431	0.0431
g__Bacteroides	s__Bacteroides_zoogleoformans	0.0151	0.0151
g__Bacteroides	s__Bacteroides_zoogleoformans	0.0151	0.0151
g__Bacteroides	s__Bacteroides_zoogleoformans	0.0151	0.0151
g__Bacteroides	s__Bacteroides_zoogleoformans	0.0151	0.0151
g__Bacteroides	s__Bacteroides_zoogleoformans	0.0151	0.0151
g__Barnesiella	s__Barnesiella_viscericola	0.0151	0.0151
g__Barnesiella	s__Barnesiella_viscericola	0.0151	0.0151
g__Barnesiella	s__Barnesiella_viscericola	0.0151	0.0151
g__Bifidobacterium	s__Bifidobacterium_longum	0.0151	0.0151
g__Blautia	s__Blautia_coccoides	0.0442	0.0442
g__Blautia	s__Blautia_hansenii	0.0442	0.0442
g__Blautia	s__Blautia_obeum	0.0151	0.0151
g__Blautia	s__Blautia_obeum	0.0151	0.0151
g__Blautia	s__Blautia_producta	0.0151	0.0151
g__Blautia	s__Blautia_wexlerae	0.0442	0.0442
g__Blautia	s__Blautia_wexlerae	0.0442	0.0442
g__Blautia	s__Blautia_wexlerae	0.0442	0.0442
g__Butyricimonas	s__Butyricimonas_virosa	0.0151	0.0151
g__Butyrivibrio	s__Butyrivibrio_crossotus	0.0151	0.0151
g__Butyrivibrio	s__Butyrivibrio_fibrisolvens	0.0151	0.0151
g__Butyrivibrio	s__Butyrivibrio_hungatei	0.0151	0.0151
g__Butyrivibrio	s__Butyrivibrio_proteoclasticus	0.0151	0.0151
g__Catenibacterium	s__Catenibacterium_mitsuokai	0.0146	0.0146
g__Catenibacterium	s__Catenibacterium_mitsuokai	0.0151	0.0151
g__Catenibacterium	s__Catenibacterium_mitsuokai	0.0431	0.0431
g__Catenibacterium	s__Catenibacterium_mitsuokai	0.0151	0.0151
g__Catenibacterium	s__Catenibacterium_mitsuokai	0.0151	0.0151
g__Catenibacterium	s__Catenibacterium_mitsuokai	0.0431	0.0431
g__Catenibacterium	s__Catenibacterium_mitsuokai	0.0151	0.0151
g__Catenibacterium	s__Catenibacterium_mitsuokai	0.0151	0.0151
g__Catenibacterium	s__Catenibacterium_mitsuokai	0.0151	0.0151
g__Catenibacterium	s__Catenibacterium_mitsuokai	0.0151	0.0151
g__Catenibacterium	s__Catenibacterium_mitsuokai	0.0431	0.0431
g__Catenibacterium	s__Catenibacterium_mitsuokai	0.0151	0.0151
g__Catenibacterium	s__Catenibacterium_mitsuokai	0.0151	0.0151
g__Catenibacterium	s__Catenibacterium_mitsuokai	0.0146	0.0146
g__Catenibacterium	s__Catenibacterium_mitsuokai	0.0442	0.0442
g__Catenibacterium	s__Catenibacterium_mitsuokai	0.0151	0.0151
g__Catenibacterium	s__Catenibacterium_mitsuokai	0.0151	0.0151
g__Catenibacterium	s__Catenibacterium_mitsuokai	0.0133	0.0133
g__Catenibacterium	s__Catenibacterium_mitsuokai	0.0442	0.0442
g__Catenibacterium	s__Catenibacterium_mitsuokai	0.0442	0.0442

g__Catenibacterium	s__Catenibacterium_mitsuokai	0.0442	0.0442
g__Catenibacterium	s__Catenibacterium_mitsuokai	0.0442	0.0442
g__Catenibacterium	s__Catenibacterium_mitsuokai	0.0442	0.0442
g__Catenibacterium	s__Catenibacterium_mitsuokai	0.0151	0.0151
g__Catenibacterium	s__Catenibacterium_mitsuokai	0.0442	0.0442
g__Catenibacterium	s__Catenibacterium_mitsuokai	0.0442	0.0442
g__Catenibacterium	s__Catenibacterium_mitsuokai	0.0151	0.0151
g__Catenibacterium	s__Catenibacterium_mitsuokai	0.0151	0.0151
g__Catenibacterium	s__Catenibacterium_mitsuokai	0.0151	0.0151
g__Catenibacterium	s__Catenibacterium_mitsuokai	0.0142	0.0142
g__Catenibacterium	s__Catenibacterium_mitsuokai	0.0151	0.0151
g__Catenibacterium	s__Catenibacterium_mitsuokai	0.0151	0.0151
g__Catenibacterium	s__Catenibacterium_mitsuokai	0.0151	0.0151
g__Catenibacterium	s__Catenibacterium_mitsuokai	0.0442	0.0442
g__Catenibacterium	s__Catenibacterium_mitsuokai	0.0151	0.0151
g__Catenibacterium	s__Catenibacterium_mitsuokai	0.04	0.04
g__Catenibacterium	s__Catenibacterium_mitsuokai	0.0151	0.0151
g__Catenibacterium	s__Catenibacterium_mitsuokai	0.0442	0.0442
g__Catenibacterium	s__Catenibacterium_mitsuokai	0.0442	0.0442
g__Catenibacterium	s__Catenibacterium_mitsuokai	0.0151	0.0151
g__Catenibacterium	s__Catenibacterium_mitsuokai	0.0151	0.0151
g__Catenibacterium	s__Catenibacterium_mitsuokai	0.0133	0.0133
g__Catenibacterium	s__Catenibacterium_mitsuokai	0.0151	0.0151
g__Catenibacterium	s__Catenibacterium_mitsuokai	0.0151	0.0151
g__Catenibacterium	s__Catenibacterium_mitsuokai	0.0431	0.0431
g__Catenibacterium	s__Catenibacterium_mitsuokai	0.0151	0.0151
g__Catenibacterium	s__Catenibacterium_mitsuokai	0.0151	0.0151
g__Catenibacterium	s__Catenibacterium_mitsuokai	0.0442	0.0442
g__Catenibacterium	s__Catenibacterium_mitsuokai	0.0151	0.0151
g__Catenibacterium	s__Catenibacterium_mitsuokai	0.0151	0.0151
g__Catenibacterium	s__Catenibacterium_mitsuokai	0.0442	0.0442
g__Catenibacterium	s__Catenibacterium_mitsuokai	0.0431	0.0431
g__Catenibacterium	s__Catenibacterium_mitsuokai	0.0151	0.0151
g__Catenibacterium	s__Catenibacterium_mitsuokai	0.0442	0.0442
g__Catenibacterium	s__Catenibacterium_mitsuokai	0.0151	0.0151
g__Catenibacterium	s__Catenibacterium_mitsuokai	0.0151	0.0151
g__Catenibacterium	s__Catenibacterium_mitsuokai	0.0146	0.0146
g__Catenibacterium	s__Catenibacterium_mitsuokai	0.0151	0.0151
g__Catenibacterium	s__Catenibacterium_mitsuokai	0.0151	0.0151
g__Catenibacterium	s__Catenibacterium_mitsuokai	0.0151	0.0151
g__Catenibacterium	s__Catenibacterium_mitsuokai	0.0151	0.0151
g__Catenibacterium	s__Catenibacterium_mitsuokai	0.0431	0.0431
g__Catenibacterium	s__Catenibacterium_mitsuokai	0.0442	0.0442

g__Catenibacterium	s__Catenibacterium_mitsuokai	0.0442	0.0442
g__Catenibacterium	s__Catenibacterium_mitsuokai	0.0151	0.0151
g__Catenibacterium	s__Catenibacterium_mitsuokai	0.0151	0.0151
g__Catenibacterium	s__Catenibacterium_mitsuokai	0.0151	0.0151
g__Catenibacterium	s__Catenibacterium_mitsuokai	0.0151	0.0151
g__Clostridioides	s__Clostridioides_difficile	0.0442	0.0442
g__Clostridioides	s__Clostridioides_difficile	0.0442	0.0442
g__Coprobacter	s__Coprobacter_fastidiosus	0.0151	0.0151
g__Coprobacter	s__Coprobacter_secundus	0.0151	0.0151
g__Coprobacter	s__Coprobacter_secundus	0.0151	0.0151
g__Coprococcus	s__Coprococcus_comes	0.0442	0.0442
g__Coprococcus	s__Coprococcus_comes	0.0151	0.0151
g__Coprococcus	s__Coprococcus_comes	0.0442	0.0442
g__Coprococcus	s__Coprococcus_eutactus	0.0442	0.0442
g__Coprococcus	s__Coprococcus_eutactus	0.0151	0.0151
g__Desulfovibrio	s__Desulfovibrio_fairfieldensis	0.0151	0.0151
g__Dorea	s__Dorea_formicigenerans	0.0442	0.0442
g__Dorea	s__Dorea_formicigenerans	0.0151	0.0151
g__Dorea	s__Dorea_longicatena	0.0151	0.0151
g__Dorea	s__Dorea_longicatena	0.0431	0.0431
g__Dorea	s__Dorea_longicatena	0.0442	0.0442
g__Dorea	s__Dorea_longicatena	0.0442	0.0442
g__Dorea	s__Dorea_longicatena	0.0151	0.0151
g__Escherichia	s__Escherichia_coli	0.0151	0.0151
g__Eubacterium	s__Eubacterium_ventriosum	0.0151	0.0151
g__Faecalibacterium	s__Faecalibacterium_prausnitzii	0.0442	0.0442
g__Faecalibacterium	s__Faecalibacterium_prausnitzii	0.0151	0.0151
g__Faecalibaculum	s__Faecalibaculum_rodentium	0.04	0.04
g__Faecalibaculum	s__Faecalibaculum_rodentium	0.0151	0.0151
g__Faecalibaculum	s__Faecalibaculum_rodentium	0.0442	0.0442
g__Faecalibaculum	s__Faecalibaculum_rodentium	0.0151	0.0151
g__Faecalibaculum	s__Faecalibaculum_rodentium	0.0151	0.0151
g__Faecalibaculum	s__Faecalibaculum_rodentium	0.0151	0.0151
g__Faecalibaculum	s__Faecalibaculum_rodentium	0.0442	0.0442
g__Faecalibaculum	s__Faecalibaculum_rodentium	0.0151	0.0151
g__Faecalibaculum	s__Faecalibaculum_rodentium	0.0151	0.0151
g__Faecalibaculum	s__Faecalibaculum_rodentium	0.0442	0.0442
g__Faecalibaculum	s__Faecalibaculum_rodentium	0.0151	0.0151
g__Faecalibaculum	s__Faecalibaculum_rodentium	0.0151	0.0151
g__Faecalibaculum	s__Faecalibaculum_rodentium	0.0442	0.0442
g__Faecalibaculum	s__Faecalibaculum_rodentium	0.0442	0.0442
g__Faecalibaculum	s__Faecalibaculum_rodentium	0.0442	0.0442
g__Faecalibaculum	s__Faecalibaculum_rodentium	0.0151	0.0151

g__Faecalibaculum	s__Faecalibaculum_rodentium	0.0151	0.0151
g__Faecalibaculum	s__Faecalibaculum_rodentium	0.0442	0.0442
g__Faecalibaculum	s__Faecalibaculum_rodentium	0.0442	0.0442
g__Faecalibaculum	s__Faecalibaculum_rodentium	0.0442	0.0442
g__Faecalibaculum	s__Faecalibaculum_rodentium	0.0442	0.0442
g__Flavonifractor	s__Flavonifractor_plautii	0.0151	0.0151
g__Flavonifractor	s__Flavonifractor_plautii	0.0442	0.0442
g__Flavonifractor	s__Flavonifractor_plautii	0.0442	0.0442
g__Flavonifractor	s__Flavonifractor_plautii	0.0151	0.0151
g__Gardnerella	s__Gardnerella_vaginalis	0.0151	0.0151
g__Gardnerella	s__Gardnerella_vaginalis	0.0151	0.0151
g__Klebsiella	s__Klebsiella_oxytoca	0.0146	0.0146
g__Klebsiella	s__Klebsiella_oxytoca	0.0151	0.0151
g__Klebsiella	s__Klebsiella_oxytoca	0.0431	0.0431
g__Klebsiella	s__Klebsiella_oxytoca	0.0442	0.0442
g__Klebsiella	s__Klebsiella_oxytoca	0.0442	0.0442
g__Klebsiella	s__Klebsiella_oxytoca	0.0442	0.0442
g__Klebsiella	s__Klebsiella_oxytoca	0.0151	0.0151
g__Klebsiella	s__Klebsiella_oxytoca	0.0442	0.0442
g__Klebsiella	s__Klebsiella_oxytoca	0.0151	0.0151
g__Klebsiella	s__Klebsiella_oxytoca	0.0151	0.0151
g__Klebsiella	s__Klebsiella_oxytoca	0.0151	0.0151
g__Klebsiella	s__Klebsiella_oxytoca	0.0151	0.0151
g__Klebsiella	s__Klebsiella_oxytoca	0.04	0.04
g__Lactobacillus	s__Lactobacillus_acetotolerans	0.0151	0.0151
g__Lactobacillus	s__Lactobacillus_acidophilus	0.0151	0.0151
g__Lactobacillus	s__Lactobacillus_gasseri	0.0151	0.0151
g__Lactobacillus	s__Lactobacillus_gasseri	0.0151	0.0151
g__Lactobacillus	s__Lactobacillus_gasseri	0.0151	0.0151
g__Lactobacillus	s__Lactobacillus_gasseri	0.0151	0.0151
g__Lactobacillus	s__Lactobacillus_gasseri	0.0442	0.0442
g__Lactobacillus	s__Lactobacillus_gasseri	0.0151	0.0151
g__Lactobacillus	s__Lactobacillus_gasseri	0.0151	0.0151
g__Lactobacillus	s__Lactobacillus_gasseri	0.0151	0.0151
g__Lactobacillus	s__Lactobacillus_gasseri	0.0151	0.0151
g__Lactobacillus	s__Lactobacillus_gasseri	0.0442	0.0442
g__Lactobacillus	s__Lactobacillus_gasseri	0.04	0.04
g__Lactobacillus	s__Lactobacillus_gasseri	0.04	0.04
g__Lactobacillus	s__Lactobacillus_gasseri	0.0151	0.0151
g__Lactobacillus	s__Lactobacillus_gasseri	0.0151	0.0151
g__Lactobacillus	s__Lactobacillus_gasseri	0.0151	0.0151
g__Lactobacillus	s__Lactobacillus_gasseri	0.0151	0.0151
g__Lactobacillus	s__Lactobacillus_gasseri	0.0151	0.0151

g__Lactobacillus	s__Lactobacillus_gasseri	0.0151	0.0151
g__Lactobacillus	s__Lactobacillus_gasseri	0.0442	0.0442
g__Lactobacillus	s__Lactobacillus_intestinalis	0.0151	0.0151
g__Lactobacillus	s__Lactobacillus_intestinalis	0.0151	0.0151
g__Lactobacillus	s__Lactobacillus_johnsonii	0.0151	0.0151
g__Lactobacillus	s__Lactobacillus_johnsonii	0.0151	0.0151
g__Lactobacillus	s__Lactobacillus_johnsonii	0.0151	0.0151
g__Lactobacillus	s__Lactobacillus_johnsonii	0.0151	0.0151
g__Lactobacillus	s__Lactobacillus_johnsonii	0.0151	0.0151
g__Lactobacillus	s__Lactobacillus_johnsonii	0.0151	0.0151
g__Lactobacillus	s__Lactobacillus_johnsonii	0.0151	0.0151
g__Lactobacillus	s__Lactobacillus_johnsonii	0.0151	0.0151
g__Lactobacillus	s__Lactobacillus_johnsonii	0.0151	0.0151
g__Lactobacillus	s__Lactobacillus_johnsonii	0.0151	0.0151
g__Lactobacillus	s__Lactobacillus_johnsonii	0.0151	0.0151
g__Lactobacillus	s__Lactobacillus_johnsonii	0.0442	0.0442
g__Lactobacillus	s__Lactobacillus_johnsonii	0.0151	0.0151
g__Lactobacillus	s__Lactobacillus_johnsonii	0.0151	0.0151
g__Lactobacillus	s__Lactobacillus_johnsonii	0.0151	0.0151
g__Lactobacillus	s__Lactobacillus_johnsonii	0.0442	0.0442
g__Lactobacillus	s__Lactobacillus_johnsonii	0.0151	0.0151
g__Lactobacillus	s__Lactobacillus_johnsonii	0.0442	0.0442
g__Lactobacillus	s__Lactobacillus_johnsonii	0.0151	0.0151
g__Lactobacillus	s__Lactobacillus_johnsonii	0.0151	0.0151
g__Lactobacillus	s__Lactobacillus_johnsonii	0.0151	0.0151
g__Lactobacillus	s__Lactobacillus_johnsonii	0.0442	0.0442
g__Lactobacillus	s__Lactobacillus_johnsonii	0.0151	0.0151
g__Lactobacillus	s__Lactobacillus_johnsonii	0.0151	0.0151
g__Lactobacillus	s__Lactobacillus_johnsonii	0.0151	0.0151
g__Lactobacillus	s__Lactobacillus_johnsonii	0.0151	0.0151
g__Lactobacillus	s__Lactobacillus_johnsonii	0.0442	0.0442
g__Lactobacillus	s__Lactobacillus_johnsonii	0.0151	0.0151
g__Lactobacillus	s__Lactobacillus_johnsonii	0.0151	0.0151
g__Lactobacillus	s__Lactobacillus_johnsonii	0.0151	0.0151
g__Lactobacillus	s__Lactobacillus_johnsonii	0.0151	0.0151
g__Lactobacillus	s__Lactobacillus_johnsonii	0.0151	0.0151
g__Lactobacillus	s__Lactobacillus_johnsonii	0.0151	0.0151
g__Lactobacillus	s__Lactobacillus_johnsonii	0.0442	0.0442
g__Lactobacillus	s__Lactobacillus_johnsonii	0.0442	0.0442
g__Lactobacillus	s__Lactobacillus_johnsonii	0.0151	0.0151
g__Lactobacillus	s__Lactobacillus_johnsonii	0.0442	0.0442
g__Lactobacillus	s__Lactobacillus_johnsonii	0.0442	0.0442
g__Lactobacillus	s__Lactobacillus_johnsonii	0.0151	0.0151

g__Lactobacillus	s__Lactobacillus_johnsonii	0.0151	0.0151
g__Lactobacillus	s__Lactobacillus_johnsonii	0.0442	0.0442
g__Lactobacillus	s__Lactobacillus_johnsonii	0.0151	0.0151
g__Lactobacillus	s__Lactobacillus_johnsonii	0.0151	0.0151
g__Lactobacillus	s__Lactobacillus_johnsonii	0.0442	0.0442
g__Lactobacillus	s__Lactobacillus_johnsonii	0.0151	0.0151
g__Lactobacillus	s__Lactobacillus_johnsonii	0.0442	0.0442
g__Lactobacillus	s__Lactobacillus_johnsonii	0.0151	0.0151
g__Lactobacillus	s__Lactobacillus_johnsonii	0.0151	0.0151
g__Lactobacillus	s__Lactobacillus_johnsonii	0.0442	0.0442
g__Lactobacillus	s__Lactobacillus_johnsonii	0.0151	0.0151
g__Lactobacillus	s__Lactobacillus_johnsonii	0.0151	0.0151
g__Lactobacillus	s__Lactobacillus_johnsonii	0.0151	0.0151
g__Lactobacillus	s__Lactobacillus_johnsonii	0.0151	0.0151
g__Lactobacillus	s__Lactobacillus_johnsonii	0.0151	0.0151
g__Lactobacillus	s__Lactobacillus_johnsonii	0.0151	0.0151
g__Lactobacillus	s__Lactobacillus_johnsonii	0.0151	0.0151
g__Lactobacillus	s__Lactobacillus_johnsonii	0.0151	0.0151
g__Lactobacillus	s__Lactobacillus_johnsonii	0.0151	0.0151
g__Lactobacillus	s__Lactobacillus_johnsonii	0.0151	0.0151
g__Lactobacillus	s__Lactobacillus_johnsonii	0.0442	0.0442
g__Lactobacillus	s__Lactobacillus_johnsonii	0.0151	0.0151
g__Lactobacillus	s__Lactobacillus_johnsonii	0.0151	0.0151
g__Lactobacillus	s__Lactobacillus_johnsonii	0.0151	0.0151
g__Lactobacillus	s__Lactobacillus_johnsonii	0.0151	0.0151
g__Lactobacillus	s__Lactobacillus_johnsonii	0.0151	0.0151
g__Lactobacillus	s__Lactobacillus_johnsonii	0.0151	0.0151
g__Lactobacillus	s__Lactobacillus_johnsonii	0.0151	0.0151
g__Lactobacillus	s__Lactobacillus_johnsonii	0.0151	0.0151
g__Lactobacillus	s__Lactobacillus_johnsonii	0.0151	0.0151
g__Lactobacillus	s__Lactobacillus_johnsonii	0.0151	0.0151
g__Lactobacillus	s__Lactobacillus_johnsonii	0.0151	0.0151
g__Lactobacillus	s__Lactobacillus_johnsonii	0.0151	0.0151
g__Lactobacillus	s__Lactobacillus_johnsonii	0.0151	0.0151
g__Lactobacillus	s__Lactobacillus_johnsonii	0.0151	0.0151
g__Lactobacillus	s__Lactobacillus_johnsonii	0.0151	0.0151
g__Lactobacillus	s__Lactobacillus_johnsonii	0.0151	0.0151
g__Lactobacillus	s__Lactobacillus_johnsonii	0.0151	0.0151
g__Lactobacillus	s__Lactobacillus_johnsonii	0.0151	0.0151
g__Lactobacillus	s__Lactobacillus_johnsonii	0.0151	0.0151
g__Lactobacillus	s__Lactobacillus_johnsonii	0.0442	0.0442
g__Lactobacillus	s__Lactobacillus_johnsonii	0.0442	0.0442
g__Lactobacillus	s__Lactobacillus_johnsonii	0.0151	0.0151

g__Lactobacillus	s__Lactobacillus_johnsonii	0.0151	0.0151
g__Lactobacillus	s__Lactobacillus_johnsonii	0.0151	0.0151
g__Lactobacillus	s__Lactobacillus_johnsonii	0.0151	0.0151
g__Lactobacillus	s__Lactobacillus_johnsonii	0.0442	0.0442
g__Lactobacillus	s__Lactobacillus_johnsonii	0.0151	0.0151
g__Lactobacillus	s__Lactobacillus_johnsonii	0.0151	0.0151
g__Lactobacillus	s__Lactobacillus_johnsonii	0.0151	0.0151
g__Lactobacillus	s__Lactobacillus_johnsonii	0.0151	0.0151
g__Lactobacillus	s__Lactobacillus_johnsonii	0.0151	0.0151
g__Lactobacillus	s__Lactobacillus_johnsonii	0.0151	0.0151
g__Lactobacillus	s__Lactobacillus_johnsonii	0.0151	0.0151
g__Lactobacillus	s__Lactobacillus_johnsonii	0.0151	0.0151
g__Lactobacillus	s__Lactobacillus_johnsonii	0.0151	0.0151
g__Lactobacillus	s__Lactobacillus_johnsonii	0.0151	0.0151
g__Lactobacillus	s__Lactobacillus_johnsonii	0.0151	0.0151
g__Lactobacillus	s__Lactobacillus_johnsonii	0.0151	0.0151
g__Lactobacillus	s__Lactobacillus_johnsonii	0.0151	0.0151
g__Lactobacillus	s__Lactobacillus_johnsonii	0.0151	0.0151
g__Lactobacillus	s__Lactobacillus_johnsonii	0.0151	0.0151
g__Lactobacillus	s__Lactobacillus_johnsonii	0.0442	0.0442
g__Lactobacillus	s__Lactobacillus_johnsonii	0.0151	0.0151
g__Lactobacillus	s__Lactobacillus_johnsonii	0.0442	0.0442
g__Lactobacillus	s__Lactobacillus_johnsonii	0.0151	0.0151
g__Lactobacillus	s__Lactobacillus_johnsonii	0.0442	0.0442
g__Lactobacillus	s__Lactobacillus_johnsonii	0.0151	0.0151
g__Lactobacillus	s__Lactobacillus_johnsonii	0.0151	0.0151
g__Lactobacillus	s__Lactobacillus_johnsonii	0.0151	0.0151
g__Lactobacillus	s__Lactobacillus_johnsonii	0.0442	0.0442
g__Lactobacillus	s__Lactobacillus_johnsonii	0.0151	0.0151
g__Lactobacillus	s__Lactobacillus_johnsonii	0.0151	0.0151
g__Lactobacillus	s__Lactobacillus_johnsonii	0.0151	0.0151
g__Lactobacillus	s__Lactobacillus_johnsonii	0.0151	0.0151
g__Lactobacillus	s__Lactobacillus_johnsonii	0.0151	0.0151
g__Lactobacillus	s__Lactobacillus_johnsonii	0.0442	0.0442
g__Lactobacillus	s__Lactobacillus_johnsonii	0.0151	0.0151
g__Lactobacillus	s__Lactobacillus_johnsonii	0.0151	0.0151
g__Lactobacillus	s__Lactobacillus_johnsonii	0.0151	0.0151
g__Lactobacillus	s__Lactobacillus_johnsonii	0.0151	0.0151
g__Lactobacillus	s__Lactobacillus_johnsonii	0.0151	0.0151
g__Lactobacillus	s__Lactobacillus_johnsonii	0.0151	0.0151
g__Lactobacillus	s__Lactobacillus_johnsonii	0.0151	0.0151
g__Lactobacillus	s__Lactobacillus_johnsonii	0.0151	0.0151
g__Lactobacillus	s__Lactobacillus_johnsonii	0.0442	0.0442

g__Lactobacillus	s__Lactobacillus_johnsonii	0.0442	0.0442
g__Lactobacillus	s__Lactobacillus_johnsonii	0.0151	0.0151
g__Lactobacillus	s__Lactobacillus_johnsonii	0.0151	0.0151
g__Lactobacillus	s__Lactobacillus_johnsonii	0.0442	0.0442
g__Lactobacillus	s__Lactobacillus_johnsonii	0.0151	0.0151
g__Lactobacillus	s__Lactobacillus_johnsonii	0.0151	0.0151
g__Lactobacillus	s__Lactobacillus_johnsonii	0.0151	0.0151
g__Lactobacillus	s__Lactobacillus_johnsonii	0.0151	0.0151
g__Lactobacillus	s__Lactobacillus_johnsonii	0.0151	0.0151
g__Lactobacillus	s__Lactobacillus_johnsonii	0.0151	0.0151
g__Lactobacillus	s__Lactobacillus_johnsonii	0.0151	0.0151
g__Lactobacillus	s__Lactobacillus_johnsonii	0.0151	0.0151
g__Lactobacillus	s__Lactobacillus_johnsonii	0.0151	0.0151
g__Lactobacillus	s__Lactobacillus_johnsonii	0.0151	0.0151
g__Lactobacillus	s__Lactobacillus_johnsonii	0.0151	0.0151
g__Lactobacillus	s__Lactobacillus_johnsonii	0.0151	0.0151
g__Lactobacillus	s__Lactobacillus_johnsonii	0.0151	0.0151
g__Lactobacillus	s__Lactobacillus_johnsonii	0.0151	0.0151
g__Lactobacillus	s__Lactobacillus_johnsonii	0.0151	0.0151
g__Lactobacillus	s__Lactobacillus_johnsonii	0.0151	0.0151
g__Lactobacillus	s__Lactobacillus_johnsonii	0.0151	0.0151
g__Lactobacillus	s__Lactobacillus_johnsonii	0.0151	0.0151
g__Lactobacillus	s__Lactobacillus_johnsonii	0.0151	0.0151
g__Lactobacillus	s__Lactobacillus_johnsonii	0.0151	0.0151
g__Lactobacillus	s__Lactobacillus_johnsonii	0.0442	0.0442
g__Lactobacillus	s__Lactobacillus_johnsonii	0.0151	0.0151
g__Lactobacillus	s__Lactobacillus_johnsonii	0.0151	0.0151
g__Lactobacillus	s__Lactobacillus_johnsonii	0.0442	0.0442
g__Lactobacillus	s__Lactobacillus_johnsonii	0.0151	0.0151
g__Lactobacillus	s__Lactobacillus_johnsonii	0.0442	0.0442
g__Lactobacillus	s__Lactobacillus_johnsonii	0.0151	0.0151
g__Lactobacillus	s__Lactobacillus_johnsonii	0.0151	0.0151
g__Lactobacillus	s__Lactobacillus_johnsonii	0.0151	0.0151
g__Lactobacillus	s__Lactobacillus_johnsonii	0.0151	0.0151
g__Lactobacillus	s__Lactobacillus_johnsonii	0.0151	0.0151
g__Lactobacillus	s__Lactobacillus_johnsonii	0.0442	0.0442
g__Lactobacillus	s__Lactobacillus_johnsonii	0.0442	0.0442
g__Lactobacillus	s__Lactobacillus_johnsonii	0.0151	0.0151
g__Lactobacillus	s__Lactobacillus_johnsonii	0.0442	0.0442
g__Lactobacillus	s__Lactobacillus_johnsonii	0.0151	0.0151
g__Lactobacillus	s__Lactobacillus_johnsonii	0.0151	0.0151
g__Lactobacillus	s__Lactobacillus_johnsonii	0.0151	0.0151
g__Lactobacillus	s__Lactobacillus_johnsonii	0.0431	0.0431

g__Lactobacillus	s__Lactobacillus_johnsonii	0.0151	0.0151
g__Lactobacillus	s__Lactobacillus_johnsonii	0.0442	0.0442
g__Lactobacillus	s__Lactobacillus_johnsonii	0.0151	0.0151
g__Lactobacillus	s__Lactobacillus_johnsonii	0.0151	0.0151
g__Lactobacillus	s__Lactobacillus_johnsonii	0.0151	0.0151
g__Lactobacillus	s__Lactobacillus_johnsonii	0.0151	0.0151
g__Lactobacillus	s__Lactobacillus_johnsonii	0.0151	0.0151
g__Lactobacillus	s__Lactobacillus_johnsonii	0.0442	0.0442
g__Lactobacillus	s__Lactobacillus_johnsonii	0.0442	0.0442
g__Lactobacillus	s__Lactobacillus_johnsonii	0.0151	0.0151
g__Lactobacillus	s__Lactobacillus_johnsonii	0.0442	0.0442
g__Lactobacillus	s__Lactobacillus_johnsonii	0.0151	0.0151
g__Lactobacillus	s__Lactobacillus_johnsonii	0.0151	0.0151
g__Lactobacillus	s__Lactobacillus_johnsonii	0.0151	0.0151
g__Lactobacillus	s__Lactobacillus_johnsonii	0.0151	0.0151
g__Lactobacillus	s__Lactobacillus_johnsonii	0.0151	0.0151
g__Lactobacillus	s__Lactobacillus_johnsonii	0.0151	0.0151
g__Lactobacillus	s__Lactobacillus_johnsonii	0.0151	0.0151
g__Lactobacillus	s__Lactobacillus_johnsonii	0.0151	0.0151
g__Lactobacillus	s__Lactobacillus_johnsonii	0.0151	0.0151
g__Lactobacillus	s__Lactobacillus_johnsonii	0.0151	0.0151
g__Lactobacillus	s__Lactobacillus_johnsonii	0.0151	0.0151
g__Lactobacillus	s__Lactobacillus_johnsonii	0.0151	0.0151
g__Lactobacillus	s__Lactobacillus_johnsonii	0.0442	0.0442
g__Lactobacillus	s__Lactobacillus_johnsonii	0.0442	0.0442
g__Lactobacillus	s__Lactobacillus_johnsonii	0.0151	0.0151
g__Lactobacillus	s__Lactobacillus_johnsonii	0.0151	0.0151
g__Lactobacillus	s__Lactobacillus_johnsonii	0.0151	0.0151
g__Lactobacillus	s__Lactobacillus_johnsonii	0.0151	0.0151
g__Lactobacillus	s__Lactobacillus_johnsonii	0.0151	0.0151
g__Lactobacillus	s__Lactobacillus_johnsonii	0.0151	0.0151
g__Lactobacillus	s__Lactobacillus_johnsonii	0.0151	0.0151
g__Lactobacillus	s__Lactobacillus_johnsonii	0.0151	0.0151
g__Lactobacillus	s__Lactobacillus_johnsonii	0.0151	0.0151
g__Lactobacillus	s__Lactobacillus_johnsonii	0.0442	0.0442
g__Lactobacillus	s__Lactobacillus_johnsonii	0.0151	0.0151
g__Lactobacillus	s__Lactobacillus_johnsonii	0.0151	0.0151
g__Lactobacillus	s__Lactobacillus_johnsonii	0.0151	0.0151
g__Lactobacillus	s__Lactobacillus_johnsonii	0.0151	0.0151
g__Lactobacillus	s__Lactobacillus_johnsonii	0.0151	0.0151
g__Lactobacillus	s__Lactobacillus_johnsonii	0.0151	0.0151
g__Lactobacillus	s__Lactobacillus_johnsonii	0.0151	0.0151
g__Lactobacillus	s__Lactobacillus_johnsonii	0.0151	0.0151

g__Lactobacillus	s__Lactobacillus_johnsonii	0.0151	0.0151
g__Lactobacillus	s__Lactobacillus_johnsonii	0.0151	0.0151
g__Lactobacillus	s__Lactobacillus_johnsonii	0.0442	0.0442
g__Lactobacillus	s__Lactobacillus_johnsonii	0.0442	0.0442
g__Lactobacillus	s__Lactobacillus_johnsonii	0.0151	0.0151
g__Lactobacillus	s__Lactobacillus_johnsonii	0.0442	0.0442
g__Lactobacillus	s__Lactobacillus_johnsonii	0.0442	0.0442
g__Lactobacillus	s__Lactobacillus_johnsonii	0.0151	0.0151
g__Lactobacillus	s__Lactobacillus_johnsonii	0.0151	0.0151
g__Lactobacillus	s__Lactobacillus_johnsonii	0.0442	0.0442
g__Lactobacillus	s__Lactobacillus_johnsonii	0.0151	0.0151
g__Lactobacillus	s__Lactobacillus_johnsonii	0.0151	0.0151
g__Lactobacillus	s__Lactobacillus_johnsonii	0.0151	0.0151
g__Lactobacillus	s__Lactobacillus_johnsonii	0.0151	0.0151
g__Lactobacillus	s__Lactobacillus_johnsonii	0.0151	0.0151
g__Lactobacillus	s__Lactobacillus_johnsonii	0.0151	0.0151
g__Lactobacillus	s__Lactobacillus_johnsonii	0.0151	0.0151
g__Lactobacillus	s__Lactobacillus_johnsonii	0.0151	0.0151
g__Lactobacillus	s__Lactobacillus_johnsonii	0.0442	0.0442
g__Lactobacillus	s__Lactobacillus_paragasseri	0.04	0.04
g__Lactobacillus	s__Lactobacillus_paragasseri	0.0151	0.0151
g__Lactobacillus	s__Lactobacillus_paragasseri	0.0151	0.0151
g__Lactobacillus	s__Lactobacillus_paragasseri	0.0151	0.0151
g__Lactobacillus	s__Lactobacillus_paragasseri	0.0442	0.0442
g__Lactobacillus	s__Lactobacillus_paragasseri	0.0151	0.0151
g__Lactobacillus	s__Lactobacillus_paragasseri	0.0151	0.0151
g__Lactobacillus	s__Lactobacillus_paragasseri	0.0146	0.0146
g__Lactobacillus	s__Lactobacillus_paragasseri	0.0151	0.0151
g__Lactobacillus	s__Lactobacillus_paragasseri	0.0151	0.0151
g__Lactobacillus	s__Lactobacillus_paragasseri	0.0151	0.0151
g__Lactobacillus	s__Lactobacillus_paragasseri	0.0151	0.0151
g__Lactobacillus	s__Lactobacillus_paragasseri	0.0151	0.0151
g__Lactobacillus	s__Lactobacillus_taiwanensis	0.0151	0.0151
g__Lactobacillus	s__Lactobacillus_taiwanensis	0.0151	0.0151
g__Lactobacillus	s__Lactobacillus_taiwanensis	0.0146	0.0146
g__Lactobacillus	s__Lactobacillus_taiwanensis	0.0151	0.0151
g__Lactobacillus	s__Lactobacillus_taiwanensis	0.0151	0.0151
g__Lactobacillus	s__Lactobacillus_taiwanensis	0.0442	0.0442
g__Lactobacillus	s__Lactobacillus_taiwanensis	0.0151	0.0151
g__Lactobacillus	s__Lactobacillus_taiwanensis	0.0151	0.0151
g__Lactobacillus	s__Lactobacillus_taiwanensis	0.0151	0.0151
g__Lactobacillus	s__Lactobacillus_taiwanensis	0.0151	0.0151
g__Lactobacillus	s__Lactobacillus_taiwanensis	0.0151	0.0151

g__Lactobacillus	s__Lactobacillus_taiwanensis	0.0151	0.0151
g__Lactobacillus	s__Lactobacillus_taiwanensis	0.0151	0.0151
g__Lactobacillus	s__Lactobacillus_taiwanensis	0.0151	0.0151
g__Lactobacillus	s__Lactobacillus_taiwanensis	0.0151	0.0151
g__Lactobacillus	s__Lactobacillus_taiwanensis	0.0151	0.0151
g__Lactobacillus	s__Lactobacillus_taiwanensis	0.0151	0.0151
g__Lactobacillus	s__Lactobacillus_taiwanensis	0.0151	0.0151
g__Lactobacillus	s__Lactobacillus_taiwanensis	0.0151	0.0151
g__Lactobacillus	s__Lactobacillus_taiwanensis	0.0151	0.0151
g__Lactobacillus	s__Lactobacillus_taiwanensis	0.0146	0.0146
g__Lactobacillus	s__Lactobacillus_taiwanensis	0.0151	0.0151
g__Lactobacillus	s__Lactobacillus_taiwanensis	0.0151	0.0151
g__Lactobacillus	s__Lactobacillus_taiwanensis	0.0151	0.0151
g__Lactobacillus	s__Lactobacillus_taiwanensis	0.0151	0.0151
g__Lactobacillus	s__Lactobacillus_taiwanensis	0.0151	0.0151
g__Lactobacillus	s__Lactobacillus_taiwanensis	0.0151	0.0151
g__Lactobacillus	s__Lactobacillus_taiwanensis	0.0151	0.0151
g__Lactobacillus	s__Lactobacillus_taiwanensis	0.0151	0.0151
g__Lactobacillus	s__Lactobacillus_taiwanensis	0.0442	0.0442
g__Lactobacillus	s__Lactobacillus_taiwanensis	0.0151	0.0151
g__Lactobacillus	s__Lactobacillus_taiwanensis	0.0442	0.0442
g__Lactobacillus	s__Lactobacillus_taiwanensis	0.0151	0.0151
g__Lactobacillus	s__Lactobacillus_taiwanensis	0.0151	0.0151
g__Lactobacillus	s__Lactobacillus_taiwanensis	0.0151	0.0151
g__Lactobacillus	s__Lactobacillus_taiwanensis	0.0151	0.0151
g__Lactobacillus	s__Lactobacillus_taiwanensis	0.0151	0.0151
g__Lactobacillus	s__Lactobacillus_taiwanensis	0.0442	0.0442
g__Lactobacillus	s__Lactobacillus_taiwanensis	0.0442	0.0442
g__Lactobacillus	s__Lactobacillus_taiwanensis	0.0151	0.0151
g__Lactobacillus	s__Lactobacillus_taiwanensis	0.0151	0.0151
g__Lactobacillus	s__Lactobacillus_taiwanensis	0.0442	0.0442
g__Lactobacillus	s__Lactobacillus_taiwanensis	0.0151	0.0151
g__Lactobacillus	s__Lactobacillus_taiwanensis	0.0151	0.0151
g__Lactobacillus	s__Lactobacillus_taiwanensis	0.0151	0.0151
g__Lactobacillus	s__Lactobacillus_taiwanensis	0.0151	0.0151
g__Lactobacillus	s__Lactobacillus_taiwanensis	0.0442	0.0442
g__Lactobacillus	s__Lactobacillus_taiwanensis	0.0151	0.0151
g__Lactobacillus	s__Lactobacillus_taiwanensis	0.0151	0.0151
g__Lactobacillus	s__Lactobacillus_taiwanensis	0.0151	0.0151
g__Lactobacillus	s__Lactobacillus_taiwanensis	0.0151	0.0151
g__Lawsonibacter	s__Lawsonibacter_asaccharolyticus	0.0442	0.0442
g__Lawsonibacter	s__Lawsonibacter_asaccharolyticus	0.0151	0.0151
g__Marvinbryantia	s__Marvinbryantia_formatexigens	0.0151	0.0151

g__Mucispirillum	s__Mucispirillum_schaedleri	0.0151	0.0151
g__Mucispirillum	s__Mucispirillum_schaedleri	0.0151	0.0151
g__Mucispirillum	s__Mucispirillum_schaedleri	0.0151	0.0151
g__Mucispirillum	s__Mucispirillum_schaedleri	0.0151	0.0151
g__Mucispirillum	s__Mucispirillum_schaedleri	0.0151	0.0151
g__Mucispirillum	s__Mucispirillum_schaedleri	0.0151	0.0151
g__Mucispirillum	s__Mucispirillum_schaedleri	0.0151	0.0151
g__Mucispirillum	s__Mucispirillum_schaedleri	0.0151	0.0151
g__Mucispirillum	s__Mucispirillum_schaedleri	0.0151	0.0151
g__Mucispirillum	s__Mucispirillum_schaedleri	0.0151	0.0151
g__Mucispirillum	s__Mucispirillum_schaedleri	0.0151	0.0151
g__Mucispirillum	s__Mucispirillum_schaedleri	0.0151	0.0151
g__Mucispirillum	s__Mucispirillum_schaedleri	0.0151	0.0151
g__Mucispirillum	s__Mucispirillum_schaedleri	0.0151	0.0151
g__Mucispirillum	s__Mucispirillum_schaedleri	0.0151	0.0151
g__Mucispirillum	s__Mucispirillum_schaedleri	0.0151	0.0151
g__Mucispirillum	s__Mucispirillum_schaedleri	0.0151	0.0151
g__Mucispirillum	s__Mucispirillum_schaedleri	0.0151	0.0151
g__Mucispirillum	s__Mucispirillum_schaedleri	0.0151	0.0151
g__Mucispirillum	s__Mucispirillum_schaedleri	0.0442	0.0442
g__Mucispirillum	s__Mucispirillum_schaedleri	0.0151	0.0151
g__Mucispirillum	s__Mucispirillum_schaedleri	0.0151	0.0151
g__Mucispirillum	s__Mucispirillum_schaedleri	0.0442	0.0442
g__Mucispirillum	s__Mucispirillum_schaedleri	0.0151	0.0151
g__Mucispirillum	s__Mucispirillum_schaedleri	0.0151	0.0151
g__Mucispirillum	s__Mucispirillum_schaedleri	0.0431	0.0431
g__Mucispirillum	s__Mucispirillum_schaedleri	0.0151	0.0151
g__Mucispirillum	s__Mucispirillum_schaedleri	0.0151	0.0151
g__Mucispirillum	s__Mucispirillum_schaedleri	0.0151	0.0151
g__Mucispirillum	s__Mucispirillum_schaedleri	0.0151	0.0151
g__Mucispirillum	s__Mucispirillum_schaedleri	0.0151	0.0151
g__Mucispirillum	s__Mucispirillum_schaedleri	0.0151	0.0151
g__Mucispirillum	s__Mucispirillum_schaedleri	0.0151	0.0151
g__Mucispirillum	s__Mucispirillum_schaedleri	0.0151	0.0151
g__Mucispirillum	s__Mucispirillum_schaedleri	0.0151	0.0151
g__Mucispirillum	s__Mucispirillum_schaedleri	0.0151	0.0151
g__Mucispirillum	s__Mucispirillum_schaedleri	0.0151	0.0151
g__Mucispirillum	s__Mucispirillum_schaedleri	0.0151	0.0151
g__Mucispirillum	s__Mucispirillum_schaedleri	0.0151	0.0151
g__Mucispirillum	s__Mucispirillum_schaedleri	0.0151	0.0151
g__Mucispirillum	s__Mucispirillum_schaedleri	0.0151	0.0151
g__Mucispirillum	s__Mucispirillum_schaedleri	0.0442	0.0442
g__Mucispirillum	s__Mucispirillum_schaedleri	0.0151	0.0151

g__Mucispirillum	s__Mucispirillum_schaedleri	0.0151	0.0151
g__Mucispirillum	s__Mucispirillum_schaedleri	0.0151	0.0151
g__Mucispirillum	s__Mucispirillum_schaedleri	0.0151	0.0151
g__Mucispirillum	s__Mucispirillum_schaedleri	0.0151	0.0151
g__Mucispirillum	s__Mucispirillum_schaedleri	0.0151	0.0151
g__Mucispirillum	s__Mucispirillum_schaedleri	0.0151	0.0151
g__Mucispirillum	s__Mucispirillum_schaedleri	0.0442	0.0442
g__Mucispirillum	s__Mucispirillum_schaedleri	0.0151	0.0151
g__Mucispirillum	s__Mucispirillum_schaedleri	0.0151	0.0151
g__Mucispirillum	s__Mucispirillum_schaedleri	0.0151	0.0151
g__Mucispirillum	s__Mucispirillum_schaedleri	0.0151	0.0151
g__Mucispirillum	s__Mucispirillum_schaedleri	0.0151	0.0151
g__Mucispirillum	s__Mucispirillum_schaedleri	0.0442	0.0442
g__Mucispirillum	s__Mucispirillum_schaedleri	0.0151	0.0151
g__Mucispirillum	s__Mucispirillum_schaedleri	0.0151	0.0151
g__Mucispirillum	s__Mucispirillum_schaedleri	0.0151	0.0151
g__Mucispirillum	s__Mucispirillum_schaedleri	0.0151	0.0151
g__Mucispirillum	s__Mucispirillum_schaedleri	0.0151	0.0151
g__Mucispirillum	s__Mucispirillum_schaedleri	0.0151	0.0151
g__Mucispirillum	s__Mucispirillum_schaedleri	0.0151	0.0151
g__Mucispirillum	s__Mucispirillum_schaedleri	0.0151	0.0151
g__Mucispirillum	s__Mucispirillum_schaedleri	0.0151	0.0151
g__Mucispirillum	s__Mucispirillum_schaedleri	0.0151	0.0151
g__Mucispirillum	s__Mucispirillum_schaedleri	0.0151	0.0151
g__Mucispirillum	s__Mucispirillum_schaedleri	0.0151	0.0151
g__Mucispirillum	s__Mucispirillum_schaedleri	0.0151	0.0151
g__Mucispirillum	s__Mucispirillum_schaedleri	0.0151	0.0151
g__Mucispirillum	s__Mucispirillum_schaedleri	0.0151	0.0151
g__Mucispirillum	s__Mucispirillum_schaedleri	0.0442	0.0442
g__Mucispirillum	s__Mucispirillum_schaedleri	0.0151	0.0151
g__Mucispirillum	s__Mucispirillum_schaedleri	0.0151	0.0151
g__Mucispirillum	s__Mucispirillum_schaedleri	0.0151	0.0151
g__Mucispirillum	s__Mucispirillum_schaedleri	0.0151	0.0151
g__Mucispirillum	s__Mucispirillum_schaedleri	0.0151	0.0151
g__Mucispirillum	s__Mucispirillum_schaedleri	0.0151	0.0151
g__Mucispirillum	s__Mucispirillum_schaedleri	0.0151	0.0151
g__Mucispirillum	s__Mucispirillum_schaedleri	0.0151	0.0151
g__Mucispirillum	s__Mucispirillum_schaedleri	0.0151	0.0151
g__Mucispirillum	s__Mucispirillum_schaedleri	0.0151	0.0151
g__Mucispirillum	s__Mucispirillum_schaedleri	0.0151	0.0151
g__Mucispirillum	s__Mucispirillum_schaedleri	0.0151	0.0151
g__Mucispirillum	s__Mucispirillum_schaedleri	0.0151	0.0151
g__Mucispirillum	s__Mucispirillum_schaedleri	0.0151	0.0151

g__Mucispirillum	s__Mucispirillum_schaedleri	0.0151	0.0151
g__Mucispirillum	s__Mucispirillum_schaedleri	0.0442	0.0442
g__Mucispirillum	s__Mucispirillum_schaedleri	0.0151	0.0151
g__Mucispirillum	s__Mucispirillum_schaedleri	0.0151	0.0151
g__Mucispirillum	s__Mucispirillum_schaedleri	0.0151	0.0151
g__Mucispirillum	s__Mucispirillum_schaedleri	0.0151	0.0151
g__Mucispirillum	s__Mucispirillum_schaedleri	0.0442	0.0442
g__Mucispirillum	s__Mucispirillum_schaedleri	0.0151	0.0151
g__Mucispirillum	s__Mucispirillum_schaedleri	0.0151	0.0151
g__Mucispirillum	s__Mucispirillum_schaedleri	0.0151	0.0151
g__Mucispirillum	s__Mucispirillum_schaedleri	0.0151	0.0151
g__Mucispirillum	s__Mucispirillum_schaedleri	0.0151	0.0151
g__Mucispirillum	s__Mucispirillum_schaedleri	0.0151	0.0151
g__Mucispirillum	s__Mucispirillum_schaedleri	0.0151	0.0151
g__Mucispirillum	s__Mucispirillum_schaedleri	0.0151	0.0151
g__Mucispirillum	s__Mucispirillum_schaedleri	0.0151	0.0151
g__Mucispirillum	s__Mucispirillum_schaedleri	0.0151	0.0151
g__Mucispirillum	s__Mucispirillum_schaedleri	0.0151	0.0151
g__Mucispirillum	s__Mucispirillum_schaedleri	0.0151	0.0151
g__Mucispirillum	s__Mucispirillum_schaedleri	0.0151	0.0151
g__Mucispirillum	s__Mucispirillum_schaedleri	0.0442	0.0442
g__Mucispirillum	s__Mucispirillum_schaedleri	0.0151	0.0151
g__Mucispirillum	s__Mucispirillum_schaedleri	0.0442	0.0442
g__Mucispirillum	s__Mucispirillum_schaedleri	0.0151	0.0151
g__Mucispirillum	s__Mucispirillum_schaedleri	0.0151	0.0151
g__Mucispirillum	s__Mucispirillum_schaedleri	0.0151	0.0151
g__Mucispirillum	s__Mucispirillum_schaedleri	0.0151	0.0151
g__Mucispirillum	s__Mucispirillum_schaedleri	0.0151	0.0151
g__Mucispirillum	s__Mucispirillum_schaedleri	0.0151	0.0151
g__Mucispirillum	s__Mucispirillum_schaedleri	0.0442	0.0442
g__Mucispirillum	s__Mucispirillum_schaedleri	0.0151	0.0151
g__Mucispirillum	s__Mucispirillum_schaedleri	0.0151	0.0151
g__Mucispirillum	s__Mucispirillum_schaedleri	0.0442	0.0442
g__Mucispirillum	s__Mucispirillum_schaedleri	0.0151	0.0151
g__Mucispirillum	s__Mucispirillum_schaedleri	0.0151	0.0151
g__Mucispirillum	s__Mucispirillum_schaedleri	0.0151	0.0151
g__Mucispirillum	s__Mucispirillum_schaedleri	0.0151	0.0151
g__Mucispirillum	s__Mucispirillum_schaedleri	0.0151	0.0151
g__Mucispirillum	s__Mucispirillum_schaedleri	0.0442	0.0442
g__Mucispirillum	s__Mucispirillum_schaedleri	0.0151	0.0151
g__Mucispirillum	s__Mucispirillum_schaedleri	0.0151	0.0151
g__Mucispirillum	s__Mucispirillum_schaedleri	0.0151	0.0151
g__Mucispirillum	s__Mucispirillum_schaedleri	0.0151	0.0151

g__Mucispirillum	s__Mucispirillum_schaedleri	0.0442	0.0442
g__Mucispirillum	s__Mucispirillum_schaedleri	0.0151	0.0151
g__Mucispirillum	s__Mucispirillum_schaedleri	0.0151	0.0151
g__Mucispirillum	s__Mucispirillum_schaedleri	0.0151	0.0151
g__Mucispirillum	s__Mucispirillum_schaedleri	0.0151	0.0151
g__Mucispirillum	s__Mucispirillum_schaedleri	0.0151	0.0151
g__Mucispirillum	s__Mucispirillum_schaedleri	0.0151	0.0151
g__Mucispirillum	s__Mucispirillum_schaedleri	0.0151	0.0151
g__Mucispirillum	s__Mucispirillum_schaedleri	0.0151	0.0151
g__Mucispirillum	s__Mucispirillum_schaedleri	0.0151	0.0151
g__Mucispirillum	s__Mucispirillum_schaedleri	0.0151	0.0151
g__Mucispirillum	s__Mucispirillum_schaedleri	0.0151	0.0151
g__Mucispirillum	s__Mucispirillum_schaedleri	0.0151	0.0151
g__Mucispirillum	s__Mucispirillum_schaedleri	0.0151	0.0151
g__Mucispirillum	s__Mucispirillum_schaedleri	0.0151	0.0151
g__Mucispirillum	s__Mucispirillum_schaedleri	0.0151	0.0151
g__Mucispirillum	s__Mucispirillum_schaedleri	0.0151	0.0151
g__Mucispirillum	s__Mucispirillum_schaedleri	0.0151	0.0151
g__Mucispirillum	s__Mucispirillum_schaedleri	0.0151	0.0151
g__Mucispirillum	s__Mucispirillum_schaedleri	0.0151	0.0151
g__Mucispirillum	s__Mucispirillum_schaedleri	0.0151	0.0151
g__Mucispirillum	s__Mucispirillum_schaedleri	0.0151	0.0151
g__Mucispirillum	s__Mucispirillum_schaedleri	0.0151	0.0151
g__Mucispirillum	s__Mucispirillum_schaedleri	0.0151	0.0151
g__Mucispirillum	s__Mucispirillum_schaedleri	0.0151	0.0151
g__Mucispirillum	s__Mucispirillum_schaedleri	0.0151	0.0151
g__Mucispirillum	s__Mucispirillum_schaedleri	0.0151	0.0151
g__Mucispirillum	s__Mucispirillum_schaedleri	0.0151	0.0151
g__Mucispirillum	s__Mucispirillum_schaedleri	0.0151	0.0151
g__Mucispirillum	s__Mucispirillum_schaedleri	0.0151	0.0151
g__Mucispirillum	s__Mucispirillum_schaedleri	0.0442	0.0442
g__Mucispirillum	s__Mucispirillum_schaedleri	0.0442	0.0442
g__Mucispirillum	s__Mucispirillum_schaedleri	0.0151	0.0151
g__Mucispirillum	s__Mucispirillum_schaedleri	0.0151	0.0151
g__Mucispirillum	s__Mucispirillum_schaedleri	0.0151	0.0151
g__Mucispirillum	s__Mucispirillum_schaedleri	0.0151	0.0151
g__Mucispirillum	s__Mucispirillum_schaedleri	0.0151	0.0151
g__Mucispirillum	s__Mucispirillum_schaedleri	0.0151	0.0151
g__Mucispirillum	s__Mucispirillum_schaedleri	0.0151	0.0151
g__Mucispirillum	s__Mucispirillum_schaedleri	0.0151	0.0151
g__Mucispirillum	s__Mucispirillum_schaedleri	0.0151	0.0151
g__Mucispirillum	s__Mucispirillum_schaedleri	0.0151	0.0151
g__Mucispirillum	s__Mucispirillum_schaedleri	0.0151	0.0151

g__Mucispirillum	s__Mucispirillum_schaedleri	0.0151	0.0151
g__Mucispirillum	s__Mucispirillum_schaedleri	0.0151	0.0151
g__Mucispirillum	s__Mucispirillum_schaedleri	0.0151	0.0151
g__Mucispirillum	s__Mucispirillum_schaedleri	0.0151	0.0151
g__Mucispirillum	s__Mucispirillum_schaedleri	0.0151	0.0151
g__Mucispirillum	s__Mucispirillum_schaedleri	0.0151	0.0151
g__Mucispirillum	s__Mucispirillum_schaedleri	0.0151	0.0151
g__Mucispirillum	s__Mucispirillum_schaedleri	0.0151	0.0151
g__Mucispirillum	s__Mucispirillum_schaedleri	0.0151	0.0151
g__Mucispirillum	s__Mucispirillum_schaedleri	0.0151	0.0151
g__Mucispirillum	s__Mucispirillum_schaedleri	0.0151	0.0151
g__Mucispirillum	s__Mucispirillum_schaedleri	0.0151	0.0151
g__Mucispirillum	s__Mucispirillum_schaedleri	0.0151	0.0151
g__Mucispirillum	s__Mucispirillum_schaedleri	0.0151	0.0151
g__Mucispirillum	s__Mucispirillum_schaedleri	0.0151	0.0151
g__Mucispirillum	s__Mucispirillum_schaedleri	0.0151	0.0151
g__Mucispirillum	s__Mucispirillum_schaedleri	0.0151	0.0151
g__Mucispirillum	s__Mucispirillum_schaedleri	0.0151	0.0151
g__Mucispirillum	s__Mucispirillum_schaedleri	0.0442	0.0442
g__Mucispirillum	s__Mucispirillum_schaedleri	0.0151	0.0151
g__Mucispirillum	s__Mucispirillum_schaedleri	0.0151	0.0151
g__Mucispirillum	s__Mucispirillum_schaedleri	0.0151	0.0151
g__Mucispirillum	s__Mucispirillum_schaedleri	0.0151	0.0151
g__Mucispirillum	s__Mucispirillum_schaedleri	0.0151	0.0151
g__Mucispirillum	s__Mucispirillum_schaedleri	0.0151	0.0151
g__Mucispirillum	s__Mucispirillum_schaedleri	0.0151	0.0151
g__Mucispirillum	s__Mucispirillum_schaedleri	0.0151	0.0151
g__Mucispirillum	s__Mucispirillum_schaedleri	0.0151	0.0151
g__Mucispirillum	s__Mucispirillum_schaedleri	0.0151	0.0151
g__Mucispirillum	s__Mucispirillum_schaedleri	0.0151	0.0151
g__Mucispirillum	s__Mucispirillum_schaedleri	0.0151	0.0151
g__Mucispirillum	s__Mucispirillum_schaedleri	0.0151	0.0151
g__Mucispirillum	s__Mucispirillum_schaedleri	0.0151	0.0151
g__Mucispirillum	s__Mucispirillum_schaedleri	0.0151	0.0151
g__Mucispirillum	s__Mucispirillum_schaedleri	0.0151	0.0151
g__Mucispirillum	s__Mucispirillum_schaedleri	0.0151	0.0151
g__Mucispirillum	s__Mucispirillum_schaedleri	0.0151	0.0151
g__Mucispirillum	s__Mucispirillum_schaedleri	0.0151	0.0151
g__Mucispirillum	s__Mucispirillum_schaedleri	0.0151	0.0151
g__Mucispirillum	s__Mucispirillum_schaedleri	0.0151	0.0151
g__Mucispirillum	s__Mucispirillum_schaedleri	0.0151	0.0151
g__Mucispirillum	s__Mucispirillum_schaedleri	0.0151	0.0151
g__Mucispirillum	s__Mucispirillum_schaedleri	0.0151	0.0151

g__Mucispirillum	s__Mucispirillum_schaedleri	0.0151	0.0151
g__Mucispirillum	s__Mucispirillum_schaedleri	0.0151	0.0151
g__Mucispirillum	s__Mucispirillum_schaedleri	0.0151	0.0151
g__Mucispirillum	s__Mucispirillum_schaedleri	0.0151	0.0151
g__Mucispirillum	s__Mucispirillum_schaedleri	0.0151	0.0151
g__Mucispirillum	s__Mucispirillum_schaedleri	0.0151	0.0151
g__Mucispirillum	s__Mucispirillum_schaedleri	0.0151	0.0151
g__Mucispirillum	s__Mucispirillum_schaedleri	0.0151	0.0151
g__Mucispirillum	s__Mucispirillum_schaedleri	0.0151	0.0151
g__Mucispirillum	s__Mucispirillum_schaedleri	0.0151	0.0151
g__Mucispirillum	s__Mucispirillum_schaedleri	0.0151	0.0151
g__Mucispirillum	s__Mucispirillum_schaedleri	0.0151	0.0151
g__Mucispirillum	s__Mucispirillum_schaedleri	0.0151	0.0151
g__Mucispirillum	s__Mucispirillum_schaedleri	0.0151	0.0151
g__Mucispirillum	s__Mucispirillum_schaedleri	0.0151	0.0151
g__Mucispirillum	s__Mucispirillum_schaedleri	0.0151	0.0151
g__Mucispirillum	s__Mucispirillum_schaedleri	0.0151	0.0151
g__Mucispirillum	s__Mucispirillum_schaedleri	0.0151	0.0151
g__Mucispirillum	s__Mucispirillum_schaedleri	0.0151	0.0151
g__Mucispirillum	s__Mucispirillum_schaedleri	0.0151	0.0151
g__Mucispirillum	s__Mucispirillum_schaedleri	0.0151	0.0151
g__Mucispirillum	s__Mucispirillum_schaedleri	0.0151	0.0151
g__Mucispirillum	s__Mucispirillum_schaedleri	0.0151	0.0151
g__Mucispirillum	s__Mucispirillum_schaedleri	0.0151	0.0151
g__Mucispirillum	s__Mucispirillum_schaedleri	0.0151	0.0151
g__Mucispirillum	s__Mucispirillum_schaedleri	0.0151	0.0151
g__Mucispirillum	s__Mucispirillum_schaedleri	0.0151	0.0151
g__Mucispirillum	s__Mucispirillum_schaedleri	0.0151	0.0151
g__Mucispirillum	s__Mucispirillum_schaedleri	0.0151	0.0151
g__Mucispirillum	s__Mucispirillum_schaedleri	0.0151	0.0151
g__Mucispirillum	s__Mucispirillum_schaedleri	0.0151	0.0151
g__Mucispirillum	s__Mucispirillum_schaedleri	0.0151	0.0151
g__Mucispirillum	s__Mucispirillum_schaedleri	0.0151	0.0151
g__Mucispirillum	s__Mucispirillum_schaedleri	0.0151	0.0151
g__Mucispirillum	s__Mucispirillum_schaedleri	0.0151	0.0151
g__Mucispirillum	s__Mucispirillum_schaedleri	0.0151	0.0151
g__Mucispirillum	s__Mucispirillum_schaedleri	0.0151	0.0151
g__Mucispirillum	s__Mucispirillum_schaedleri	0.0151	0.0151
g__Mucispirillum	s__Mucispirillum_schaedleri	0.0151	0.0151
g__Mucispirillum	s__Mucispirillum_schaedleri	0.0151	0.0151
g__Mucispirillum	s__Mucispirillum_schaedleri	0.0151	0.0151
g__Mucispirillum	s__Mucispirillum_schaedleri	0.0151	0.0151
g__Mucispirillum	s__Mucispirillum_schaedleri	0.0151	0.0151

g__Mucispirillum	s__Mucispirillum_schaedleri	0.0151	0.0151
g__Mucispirillum	s__Mucispirillum_schaedleri	0.0151	0.0151
g__Mucispirillum	s__Mucispirillum_schaedleri	0.0151	0.0151
g__Mucispirillum	s__Mucispirillum_schaedleri	0.0151	0.0151
g__Mucispirillum	s__Mucispirillum_schaedleri	0.0151	0.0151
g__Mucispirillum	s__Mucispirillum_schaedleri	0.0151	0.0151
g__Mucispirillum	s__Mucispirillum_schaedleri	0.0151	0.0151
g__Mucispirillum	s__Mucispirillum_schaedleri	0.0151	0.0151
g__Mucispirillum	s__Mucispirillum_schaedleri	0.0151	0.0151
g__Mucispirillum	s__Mucispirillum_schaedleri	0.0151	0.0151
g__Mucispirillum	s__Mucispirillum_schaedleri	0.0151	0.0151
g__Mucispirillum	s__Mucispirillum_schaedleri	0.0151	0.0151
g__Mucispirillum	s__Mucispirillum_schaedleri	0.0151	0.0151
g__Mucispirillum	s__Mucispirillum_schaedleri	0.0151	0.0151
g__Mucispirillum	s__Mucispirillum_schaedleri	0.0151	0.0151
g__Mucispirillum	s__Mucispirillum_schaedleri	0.0151	0.0151
g__Mucispirillum	s__Mucispirillum_schaedleri	0.0151	0.0151
g__Mucispirillum	s__Mucispirillum_schaedleri	0.0151	0.0151
g__Mucispirillum	s__Mucispirillum_schaedleri	0.0151	0.0151
g__Mucispirillum	s__Mucispirillum_schaedleri	0.0151	0.0151
g__Mucispirillum	s__Mucispirillum_schaedleri	0.0151	0.0151
g__Mucispirillum	s__Mucispirillum_schaedleri	0.0151	0.0151
g__Mucispirillum	s__Mucispirillum_schaedleri	0.0151	0.0151
g__Mucispirillum	s__Mucispirillum_schaedleri	0.0151	0.0151
g__Mucispirillum	s__Mucispirillum_schaedleri	0.0151	0.0151
g__Mucispirillum	s__Mucispirillum_schaedleri	0.0151	0.0151
g__Mucispirillum	s__Mucispirillum_schaedleri	0.0151	0.0151
g__Mucispirillum	s__Mucispirillum_schaedleri	0.0151	0.0151
g__Mucispirillum	s__Mucispirillum_schaedleri	0.0151	0.0151
g__Mucispirillum	s__Mucispirillum_schaedleri	0.0151	0.0151
g__Muribaculum	s__Muribaculum_intestinale	0.0151	0.0151
g__Muribaculum	s__Muribaculum_intestinale	0.0151	0.0151
g__Muribaculum	s__Muribaculum_intestinale	0.0151	0.0151
g__Muribaculum	s__Muribaculum_intestinale	0.0151	0.0151
g__Muribaculum	s__Muribaculum_intestinale	0.0151	0.0151
g__Odoribacter	s__Odoribacter_splanchnicus	0.0151	0.0151
g__Odoribacter	s__Odoribacter_splanchnicus	0.0151	0.0151
g__Ornithobacterium	s__Ornithobacterium_rhinotracheale	0.0151	0.0151
g__Parabacteroides	s__Parabacteroides_distasonis	0.0151	0.0151
g__Parabacteroides	s__Parabacteroides_distasonis	0.0151	0.0151
g__Parabacteroides	s__Parabacteroides_distasonis	0.0151	0.0151
g__Parabacteroides	s__Parabacteroides_distasonis	0.0151	0.0151
g__Parabacteroides	s__Parabacteroides_distasonis	0.0151	0.0151

g__Parabacteroides	s__Parabacteroides_distasonis	0.0151	0.0151
g__Parabacteroides	s__Parabacteroides_distasonis	0.0151	0.0151
g__Parabacteroides	s__Parabacteroides_distasonis	0.0151	0.0151
g__Parabacteroides	s__Parabacteroides_distasonis	0.0151	0.0151
g__Parabacteroides	s__Parabacteroides_distasonis	0.0151	0.0151
g__Parabacteroides	s__Parabacteroides_distasonis	0.0151	0.0151
g__Parabacteroides	s__Parabacteroides_distasonis	0.0151	0.0151
g__Parabacteroides	s__Parabacteroides_distasonis	0.0151	0.0151
g__Parabacteroides	s__Parabacteroides_goldsteinii	0.0442	0.0442
g__Parabacteroides	s__Parabacteroides_goldsteinii	0.0442	0.0442
g__Parabacteroides	s__Parabacteroides_goldsteinii	0.0442	0.0442
g__Parabacteroides	s__Parabacteroides_goldsteinii	0.0151	0.0151
g__Parabacteroides	s__Parabacteroides_goldsteinii	0.0151	0.0151
g__Parabacteroides	s__Parabacteroides_goldsteinii	0.0151	0.0151
g__Parabacteroides	s__Parabacteroides_goldsteinii	0.0151	0.0151
g__Parabacteroides	s__Parabacteroides_goldsteinii	0.0151	0.0151
g__Parabacteroides	s__Parabacteroides_goldsteinii	0.0151	0.0151
g__Parabacteroides	s__Parabacteroides_goldsteinii	0.0442	0.0442
g__Parabacteroides	s__Parabacteroides_goldsteinii	0.0151	0.0151
g__Parabacteroides	s__Parabacteroides_goldsteinii	0.0151	0.0151
g__Parabacteroides	s__Parabacteroides_goldsteinii	0.0442	0.0442
g__Parabacteroides	s__Parabacteroides_goldsteinii	0.0151	0.0151
g__Parabacteroides	s__Parabacteroides_goldsteinii	0.0151	0.0151
g__Parabacteroides	s__Parabacteroides_goldsteinii	0.0151	0.0151
g__Parabacteroides	s__Parabacteroides_goldsteinii	0.0151	0.0151
g__Parabacteroides	s__Parabacteroides_goldsteinii	0.0151	0.0151
g__Parabacteroides	s__Parabacteroides_goldsteinii	0.0151	0.0151
g__Parabacteroides	s__Parabacteroides_goldsteinii	0.0442	0.0442
g__Parabacteroides	s__Parabacteroides_goldsteinii	0.0442	0.0442
g__Parabacteroides	s__Parabacteroides_goldsteinii	0.0442	0.0442
g__Parabacteroides	s__Parabacteroides_goldsteinii	0.0151	0.0151
g__Parabacteroides	s__Parabacteroides_goldsteinii	0.0442	0.0442
g__Parabacteroides	s__Parabacteroides_goldsteinii	0.0442	0.0442
g__Parabacteroides	s__Parabacteroides_goldsteinii	0.0442	0.0442
g__Parabacteroides	s__Parabacteroides_goldsteinii	0.0151	0.0151
g__Parabacteroides	s__Parabacteroides_goldsteinii	0.0151	0.0151
g__Parabacteroides	s__Parabacteroides_goldsteinii	0.0442	0.0442
g__Parabacteroides	s__Parabacteroides_goldsteinii	0.0151	0.0151
g__Parabacteroides	s__Parabacteroides_goldsteinii	0.0151	0.0151
g__Parabacteroides	s__Parabacteroides_goldsteinii	0.0442	0.0442
g__Parabacteroides	s__Parabacteroides_goldsteinii	0.0442	0.0442
g__Parabacteroides	s__Parabacteroides_goldsteinii	0.0442	0.0442
g__Parabacteroides	s__Parabacteroides_goldsteinii	0.0442	0.0442

g__Parabacteroides	s__Parabacteroides_goldsteinii	0.0151	0.0151
g__Parabacteroides	s__Parabacteroides_goldsteinii	0.0442	0.0442
g__Parabacteroides	s__Parabacteroides_goldsteinii	0.0151	0.0151
g__Parabacteroides	s__Parabacteroides_goldsteinii	0.0442	0.0442
g__Parabacteroides	s__Parabacteroides_goldsteinii	0.0151	0.0151
g__Parabacteroides	s__Parabacteroides_goldsteinii	0.0151	0.0151
g__Parabacteroides	s__Parabacteroides_goldsteinii	0.0151	0.0151
g__Parabacteroides	s__Parabacteroides_goldsteinii	0.0151	0.0151
g__Parabacteroides	s__Parabacteroides_goldsteinii	0.0151	0.0151
g__Parabacteroides	s__Parabacteroides_goldsteinii	0.0151	0.0151
g__Parabacteroides	s__Parabacteroides_goldsteinii	0.0442	0.0442
g__Parabacteroides	s__Parabacteroides_goldsteinii	0.0442	0.0442
g__Parabacteroides	s__Parabacteroides_goldsteinii	0.0431	0.0431
g__Parabacteroides	s__Parabacteroides_goldsteinii	0.0442	0.0442
g__Parabacteroides	s__Parabacteroides_goldsteinii	0.0151	0.0151
g__Parabacteroides	s__Parabacteroides_goldsteinii	0.0151	0.0151
g__Parabacteroides	s__Parabacteroides_goldsteinii	0.0151	0.0151
g__Parabacteroides	s__Parabacteroides_goldsteinii	0.0151	0.0151
g__Parabacteroides	s__Parabacteroides_goldsteinii	0.0151	0.0151
g__Parabacteroides	s__Parabacteroides_goldsteinii	0.0442	0.0442
g__Parabacteroides	s__Parabacteroides_goldsteinii	0.0151	0.0151
g__Parabacteroides	s__Parabacteroides_goldsteinii	0.0442	0.0442
g__Parabacteroides	s__Parabacteroides_goldsteinii	0.0151	0.0151
g__Parabacteroides	s__Parabacteroides_goldsteinii	0.0442	0.0442
g__Parabacteroides	s__Parabacteroides_goldsteinii	0.0151	0.0151
g__Parabacteroides	s__Parabacteroides_goldsteinii	0.0442	0.0442
g__Parabacteroides	s__Parabacteroides_goldsteinii	0.0151	0.0151
g__Parabacteroides	s__Parabacteroides_goldsteinii	0.0151	0.0151
g__Parabacteroides	s__Parabacteroides_goldsteinii	0.0151	0.0151
g__Parabacteroides	s__Parabacteroides_goldsteinii	0.0151	0.0151
g__Parabacteroides	s__Parabacteroides_goldsteinii	0.0442	0.0442
g__Parabacteroides	s__Parabacteroides_goldsteinii	0.0151	0.0151
g__Parabacteroides	s__Parabacteroides_goldsteinii	0.0151	0.0151
g__Parabacteroides	s__Parabacteroides_goldsteinii	0.0442	0.0442
g__Parabacteroides	s__Parabacteroides_goldsteinii	0.0151	0.0151
g__Parabacteroides	s__Parabacteroides_goldsteinii	0.0442	0.0442
g__Parabacteroides	s__Parabacteroides_goldsteinii	0.0151	0.0151
g__Parabacteroides	s__Parabacteroides_goldsteinii	0.0151	0.0151
g__Parabacteroides	s__Parabacteroides_goldsteinii	0.0151	0.0151
g__Parabacteroides	s__Parabacteroides_goldsteinii	0.0151	0.0151
g__Parabacteroides	s__Parabacteroides_goldsteinii	0.0442	0.0442
g__Parabacteroides	s__Parabacteroides_goldsteinii	0.0442	0.0442
g__Parabacteroides	s__Parabacteroides_goldsteinii	0.0442	0.0442

g__Parabacteroides	s__Parabacteroides_goldsteinii	0.0151	0.0151
g__Parabacteroides	s__Parabacteroides_goldsteinii	0.0442	0.0442
g__Parabacteroides	s__Parabacteroides_goldsteinii	0.0151	0.0151
g__Parabacteroides	s__Parabacteroides_goldsteinii	0.0151	0.0151
g__Parabacteroides	s__Parabacteroides_goldsteinii	0.0442	0.0442
g__Parabacteroides	s__Parabacteroides_goldsteinii	0.0151	0.0151
g__Parabacteroides	s__Parabacteroides_goldsteinii	0.0431	0.0431
g__Parabacteroides	s__Parabacteroides_goldsteinii	0.0151	0.0151
g__Parabacteroides	s__Parabacteroides_goldsteinii	0.0442	0.0442
g__Parabacteroides	s__Parabacteroides_goldsteinii	0.0151	0.0151
g__Parabacteroides	s__Parabacteroides_goldsteinii	0.0151	0.0151
g__Parabacteroides	s__Parabacteroides_goldsteinii	0.0442	0.0442
g__Parabacteroides	s__Parabacteroides_goldsteinii	0.0151	0.0151
g__Parabacteroides	s__Parabacteroides_goldsteinii	0.0442	0.0442
g__Parabacteroides	s__Parabacteroides_goldsteinii	0.0442	0.0442
g__Parabacteroides	s__Parabacteroides_goldsteinii	0.0151	0.0151
g__Parabacteroides	s__Parabacteroides_goldsteinii	0.0151	0.0151
g__Parabacteroides	s__Parabacteroides_goldsteinii	0.0442	0.0442
g__Parabacteroides	s__Parabacteroides_goldsteinii	0.0151	0.0151
g__Parabacteroides	s__Parabacteroides_goldsteinii	0.0151	0.0151
g__Parabacteroides	s__Parabacteroides_goldsteinii	0.0151	0.0151
g__Parabacteroides	s__Parabacteroides_goldsteinii	0.0442	0.0442
g__Parabacteroides	s__Parabacteroides_goldsteinii	0.0151	0.0151
g__Parabacteroides	s__Parabacteroides_goldsteinii	0.0151	0.0151
g__Parabacteroides	s__Parabacteroides_goldsteinii	0.0151	0.0151
g__Parabacteroides	s__Parabacteroides_goldsteinii	0.0151	0.0151
g__Parabacteroides	s__Parabacteroides_johnsonii	0.0151	0.0151
g__Parabacteroides	s__Parabacteroides_johnsonii	0.0442	0.0442
g__Parabacteroides	s__Parabacteroides_johnsonii	0.0151	0.0151
g__Parabacteroides	s__Parabacteroides_johnsonii	0.0151	0.0151
g__Parabacteroides	s__Parabacteroides_johnsonii	0.0442	0.0442
g__Parabacteroides	s__Parabacteroides_johnsonii	0.0151	0.0151
g__Parabacteroides	s__Parabacteroides_johnsonii	0.0151	0.0151
g__Parabacteroides	s__Parabacteroides_johnsonii	0.0442	0.0442
g__Parabacteroides	s__Parabacteroides_merdae	0.0151	0.0151
g__Parabacteroides	s__Parabacteroides_merdae	0.0151	0.0151
g__Parabacteroides	s__Parabacteroides_merdae	0.0151	0.0151
g__Parabacteroides	s__Parabacteroides_merdae	0.0151	0.0151
g__Parabacteroides	s__Parabacteroides_merdae	0.0151	0.0151
g__Paraprevotella	s__Paraprevotella_clara	0.0151	0.0151
g__Paraprevotella	s__Paraprevotella_xylaniphila	0.0151	0.0151
g__Paraprevotella	s__Paraprevotella_xylaniphila	0.0151	0.0151
g__Pediococcus	s__Pediococcus_acidilactici	0.0442	0.0442

g__Pediococcus	s__Pediococcus_acidilactici	0.0442	0.0442
g__Pediococcus	s__Pediococcus_acidilactici	0.0442	0.0442
g__Porphyromonas	s__Porphyromonas_gingivalis	0.0151	0.0151
g__Porphyromonas	s__Porphyromonas_somerae	0.0151	0.0151
g__Prevotella	s__Prevotella_bivia	0.0151	0.0151
g__Prevotella	s__Prevotella_bivia	0.0151	0.0151
g__Prevotella	s__Prevotella_corporis	0.0151	0.0151
g__Prevotella	s__Prevotella_dentalis	0.0442	0.0442
g__Prevotella	s__Prevotella_denticola	0.0151	0.0151
g__Prevotella	s__Prevotella_denticola	0.0442	0.0442
g__Prevotella	s__Prevotella_fusca	0.0151	0.0151
g__Prevotella	s__Prevotella_histicola	0.0151	0.0151
g__Prevotella	s__Prevotella_intermedia	0.0151	0.0151
g__Prevotella	s__Prevotella_intermedia	0.0151	0.0151
g__Prevotella	s__Prevotella_intermedia	0.0151	0.0151
g__Prevotella	s__Prevotella_melaninogenica	0.0151	0.0151
g__Prevotella	s__Prevotella_melaninogenica	0.0151	0.0151
g__Prevotella	s__Prevotella_multiformis	0.0151	0.0151
g__Prevotella	s__Prevotella_nigrescens	0.0151	0.0151
g__Prevotella	s__Prevotella_scopos	0.0151	0.0151
g__Pseudobutyrivibrio	s__Pseudobutyrivibrio_xylanivorans	0.0151	0.0151
g__Roseburia	s__Roseburia_hominis	0.0442	0.0442
g__Roseburia	s__Roseburia_hominis	0.0151	0.0151
g__Roseburia	s__Roseburia_hominis	0.0151	0.0151
g__Roseburia	s__Roseburia_intestinalis	0.0151	0.0151
g__Sellimonas	s__Sellimonas_intestinalis	0.0442	0.0442
g__Sellimonas	s__Sellimonas_intestinalis	0.0151	0.0151
g__Subdoligranulum	s__Subdoligranulum_variabile	0.0151	0.0151
g__Desulfovibrio	s__Desulfovibrio_desulfuricans	0.0151	0.0151
